# Asymmetric synthesis of propargylamines as amino acid surrogates in peptidomimetics

**DOI:** 10.3762/bjoc.13.240

**Published:** 2017-11-15

**Authors:** Matthias Wünsch, David Schröder, Tanja Fröhr, Lisa Teichmann, Sebastian Hedwig, Nils Janson, Clara Belu, Jasmin Simon, Shari Heidemeyer, Philipp Holtkamp, Jens Rudlof, Lennard Klemme, Alessa Hinzmann, Beate Neumann, Hans-Georg Stammler, Norbert Sewald

**Affiliations:** 1 Organic and Bioorganic Chemistry, Department of Chemistry, Bielefeld University, Universitätsstraße 25, D-33615 Bielefeld, Germany

**Keywords:** amino acid analogous side chains, desilylation, Ellman’s chiral sulfinamide, intramolecular Huisgen reaction, peptidomimetics, propargylamines, rearrangement to α,β-unsaturated imines

## Abstract

The amide moiety of peptides can be replaced for example by a triazole moiety, which is considered to be bioisosteric. Therefore, the carbonyl moiety of an amino acid has to be replaced by an alkyne in order to provide a precursor of such peptidomimetics. As most amino acids have a chiral center at C^α^, such amide bond surrogates need a chiral moiety. Here the asymmetric synthesis of a set of 24 *N*-sulfinyl propargylamines is presented. The condensation of various aldehydes with Ellman’s chiral sulfinamide provides chiral *N-*sulfinylimines, which were reacted with (trimethylsilyl)ethynyllithium to afford diastereomerically pure *N*-sulfinyl propargylamines. Diverse functional groups present in the propargylic position resemble the side chain present at the C^α^ of amino acids. Whereas propargylamines with (cyclo)alkyl substituents can be prepared in a direct manner, residues with polar functional groups require suitable protective groups. The presence of particular functional groups in the side chain in some cases leads to remarkable side reactions of the alkyne moiety. Thus, electron-withdrawing substituents in the C^α^-position facilitate a base induced rearrangement to α,β-unsaturated imines, while azide-substituted propargylamines form triazoles under surprisingly mild conditions. A panel of propargylamines bearing fluoro or chloro substituents, polar functional groups, or basic and acidic functional groups is accessible for the use as precursors of peptidomimetics.

## Introduction

**Figure 1:**
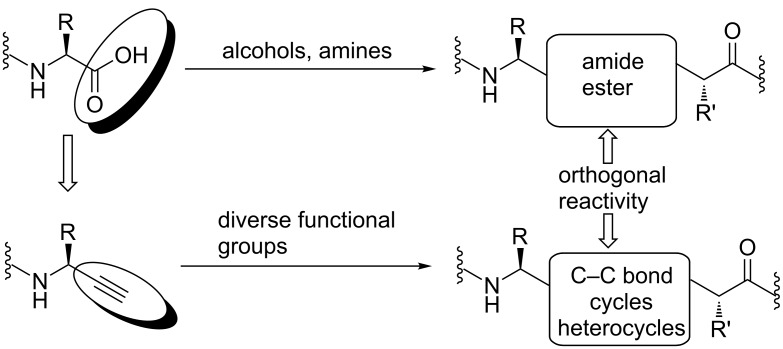
Concept of carboxylic acid or amide bond replacement on the basis of an alkyne moiety.

Terminal alkynes display an intriguing versatility as building blocks in organic and medicinal chemistry, as their reactivity is unique. Their chemistry involves several highly selective reactions, e.g., [3 + 2] cycloadditions with azides and isoelectronic functional groups (among them the copper or ruthenium-catalyzed azide–alkyne cycloaddition, CuAAC and RuAAC), the thiol–yne reaction, Diels–Alder reactions and the Sonogashira cross-coupling. While amino acids with a terminal alkyne in the side chain are well-known, the synthesis of their correlates where the carboxy group is replaced by a terminal alkyne is still tedious. Nevertheless, these propargylamines have been frequently used as precursors for the synthesis of diverse bioactive compounds. Their conversion into triazoles is best investigated, since triazoles as amide bond surrogates are found in several inhibitors of proteases such as cathepsin S [1–6], cysteine proteases [7], cruzain 20 [8–9], caspases [10] and peptidyl aminopeptidases [11]. These protease inhibitors show potential for the treatment of Chagas disease [2,9], Huntington’s disease [10], malaria [11], autoimmune diseases [6] and the imaging of tumor associated macrophages [2]. Whereas the carboxylic acid function of amino acids can be easily converted into amides or esters (Figure 1), propargylamines have been converted into acids, alcohols [12] or olefins in order to obtain natural products like angustureine and cuspareine [13].

**Figure 2:**
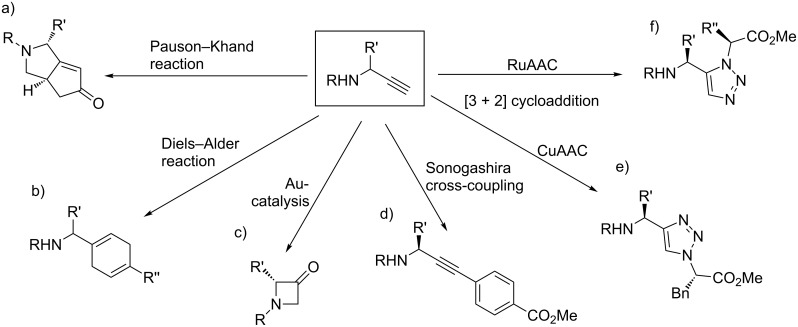
Selection of reactions based on propargylamines as precursors. a) Intramolecular Pauson–Khand reaction, R = (*S*)-*tert-*butylsulfinyl, R’ = CH_2_CH_2_OTBDPS [14]. b) Diels–Alder reaction, R = *p*Ts, R’ = H, R’’ = Me [15]. c) Gold-catalyzed intramolecular reaction to azetidin-3-ones, R = *tert*-butylsulfonyl, R’ = aromatics, aliphatics [16]. d) Sonogashira cross-coupling, R = *tert-*butylsulfinyl, R’ = Me, CHMe_2_, CH_2_CHMe_2_, cyclohexyl [17]. e,f) Cu^I^ or Ru^II^-catalyzed [3 + 2] cycloadditions (CuAAC, RuAAC) [18–21]. e) R = Boc, R’ = Bn [19]. f) R = Boc, R’ = Me, CHMe_2_, CH_2_CHMe_2_, R’’ = Me, CHMe_2_, CH_2_Ph [21].

Intramolecular Pauson–Khand reaction [14], Diels–Alder reaction [15], gold-catalyzed azetidin-3-one formation [16], as well as various transition metal-mediated additions and cross-coupling reactions [17] represent further important reactions of propargylamines, providing the potential to form innovative peptidomimetics (Figure 2).

Our attention has been focused on the synthesis of amino acid analogous propargylamines, furnished with C^α^-substituents imitating various amino acid side chains.

The direct conversion of amino acids into propargylamines by the Corey–Fuchs or the Seyferth–Gilbert homologation has been successfully used for the preparation of several natural amino acid analogues [22–23]. However, epimerization in the α-position frequently occurs under the alkaline reaction conditions of the Seyferth–Gilbert and the Corey–Fuchs reaction. In order to access propargylamines with modified side chains, we chose a de novo synthesis strategy, using Ellman’s chiral sulfinamide auxiliary to produce diastereomerically pure amines [7]. Ellman’s chiral sulfinamide can be readily synthesized on a laboratory scale [24]. Moreover, sulfinamides can be cleaved easily under acidic conditions [1,25–26] and the produced sulfinic acid can even be recycled [25,27]. However, the sulfinamide auxiliary has to be treated with care, as it tends to disproportionate quickly in solution at elevated temperature or in chloroform at room temperature [28]. Furthermore, sulfinamides are unstable upon sonication [28]. Sulfinamides are also reported to decompose in the presence of low concentrations of acid under pressure, typical conditions of HPLC analysis. Consequently, the application of the *tert-*butylsulfinyl as protective group for the amine is restricted to very mild conditions. However, it can be easily cleaved from *N*-sulfinyl propargylamines by acidic methanolysis and subsequent Boc protection [21]. Here we report on the diastereoselective synthesis of chiral *N*-sulfinyl propargylamines with amino acid type “side chains” attached to the propargylic position mediated by Ellman’s auxiliary.

**Figure 3:**
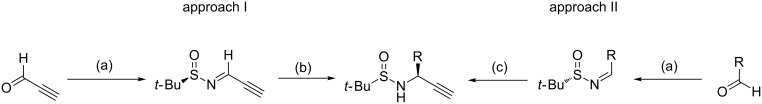
Two different approaches for the stereoselective de novo synthesis of propargylamines using Ellman’s chiral sulfinamide. (a) *tert*-Butyl sulfinamide, Lewis acid, CH_2_Cl_2_. (b) Organometallic compound. (c) (Trimethylsilyl)ethynyllithium.

Enantiomerically pure amines can be obtained by condensation of aldehydes or ketones with Ellman’s chiral sulfinamide, mediated by KHSO_4_ [29], Cs_2_CO_3_ [30], Ti(OEt)_4_ [31–33], or CuSO_4_ [34], followed by either reduction [35–40] or addition of a nucleophile to the imine [41]. In general, there are two options (Figure 3) for the synthesis of enantiomerically pure propargylamines by nucleophilic addition. The synthesis of propargylamines by diastereoselective reductive amination requires alkynyl ketones, which are difficult to prepare and are unstable towards reductive conditions.

In approach I, organometallic nucleophiles are added to *N*-sulfinyl propargylimines, derived from aldehydes. According to approach II, a metallated terminal alkyne is added to an *N-*sulfinylaldimine. In approach I, the organometallic nucleophile is transferring the amino acid side chain, in approach II, the amino acid side chain comes from the aldehyde incorporated in the imine.

## Results and Discussion

### Synthesis of propargylamines, general strategies

**Figure 4:**
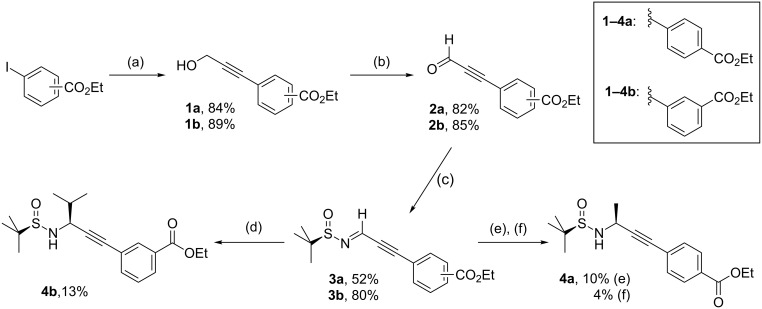
Synthesis of propargylamines **4a** and **4b** by introducing the side chain as nucleophile. (a) HC≡CCH_2_OH, Cl_2_Pd(PPh_3_)_2_ (1 mol %), CuI (2 mol %), THF/piperidine (3:1), rt, 2 h. (b) (COCl)_2_, DMSO, NEt_3_, −78 °C, DCM. (c) (*R*)-*tert-*Butyl sulfinamide ((*R*)**-1**), CuSO_4_, DCM, rt, 3 d (see GP-2). (d) iPrMgBr, THF, −38 °C, AlMe_3_ in *n*-hexane (**4a**, 13%, dr 99:1). (e) MeLi in Et_2_O, toluene, −30 °C, AlMe_3_ in *n*-hexane (**4b**, 4%, dr 52:48). (f) MeMgBr in Et_2_O, toluene, −35 °C (**4b**, 10%, dr 51:49).

To avoid side reactions of the terminal alkyne in approach I, internal alkynes were applied. Benzoate substituents were chosen as they are comparatively inert and convertible to peptidomimetics. At first, iodobenzene derivatives with an ester moiety in *p-* or *m-*position were reacted with propargyl alcohol in a Sonogashira reaction to give the phenylpropargyl alcohols **1a** and **1b** (Figure 4) [42–43], which were transformed in a Swern oxidation to afford the aldehydes **2a** and **2b** [44]. Condensation of the aldehydes **2a** and **2b** with (*R*)-configured *tert*-butyl sulfinamide led to the enantiomerically pure sulfinylimines **3a** and **3b**, which were reacted with a variety of organometallic compounds, including iPrMgBr, MeMgBr and BnMgBr.

The reaction of the enantiomerically pure *N-*sulfinylimines **3a** and **3b** with aliphatic organometallic nucleophiles resulted in low yields and diastereoselectivity. The reaction of sulfinylimine **3a** with isopropylmagnesium bromide provided the sulfinamide **4a** in only 13% yield with a ratio of diastereomers of 99:1. Only 10% of the addition product **4b** were obtained by addition of methylmagnesium bromide to the *N-*sulfinylimine **4b** (dr 51:49). In order to prepare phenylalanine analogoues, *N-*sulfinylimines **3a** and **3b** were reacted with benzylmagnesium bromide. However, only traces of the desired addition products could be detected by LC–MS analysis of the crude products.

Approach II starts with the condensation of an aldehyde with Ellman’s chiral sulfinamide to provide a sulfinylimine [29–34], which was reacted with (trimethylsilyl)ethynyllithium [1,14,45–48]. The terminal TMS group was cleaved off after the addition reaction [49–50] (Table 1).

**Table 1:** Preparation of *N*-sulfinyl propargylamines **7** with aliphatic side chains.^a^

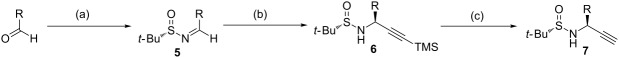					

R	(a)	Yield (**5**)	(b), (c)	Yield (**7**)^b^	dr

Me	GP-1^c^	81% (**5a**)	GP-3, GP-5	47% (**7a**)	100:0
iPr	GP-1	90% (**5b**)	GP-3, GP-5	61% (**7b**)	97:3
CH_2_-iPr	GP-1	92% (**5c**)	GP-3, GP-5	53% (**7c**)	97:3
C_6_H_11_	GP-2	96% (**5d**)	GP-3, GP-5	59% (**7d**)	97:3
*t*-Bu	GP-2	18% (**5e**)	GP-4, GP-5	41% (**7e**)	80:20
adamantyl	GP-2	42% (**5f**)	GP-4, GP-5	15% (**7f**)	100:0
CH_2_CH_2_SMe	GP-2^c^	89% (**5g**)	GP-4, GP-6	34% (**7g**)^d^	96:4

^a^(a) GP-1: Auxiliary (*S*)-**1**, aldehyde, Ti(OiPr)_4_, 70 °C, 40 min. GP-2: Auxiliary (*S*)-**1**, aldehyde, CuSO_4_, DCM, rt, 3 d. (b) GP-3: (Trimethylsilyl)ethynyllithium, Ti(OiPr)_4_, THF, −78 °C to rt. GP-4: (Trimethylsilyl)ethynyllithium, AlMe_3_, toluene, −78 °C to rt. (c) GP-5: TBAF, THF, 0 °C, 3 h. GP-6: KF, 18-crown-6, THF/H_2_O (98:2), 0 °C. ^b^Isolated yields are given, referring to **5** (over two steps (b) and (c)). ^c^GP-1 had to be modified for the synthesis of **5a**: acetaldehyde (5 equiv), MgSO_4_ (5 equiv), 30 °C, 12 h [51]. ^d^(*R*)-configured Ellman’s sulfinamide (*R*)-**1** was applied. Hence, the mirror images of **5–7g** were obtained.

Several conditions for the condensation of aldehydes with Ellman’s chiral sulfinamide (*S*)-**1** have been described. Catalysis by Brønstedt acids has been reported [29], but was not considered here due to the lability of the *tert*-butyl sulfinamide moiety towards acids [1,25–26]. Strongly alkaline conditions [30] were also avoided. Liquid aldehydes were readily condensed with *tert*-butyl sulfinamide (*S*)-**1** in the presence of Ti(OEt)_4_ as Lewis acid and water scavenger [31–33]. However, the removal of precipitated TiO_2_ was tedious and time consuming. Therefore, dry CuSO_4_ as Lewis acid and water scavenger at ambient temperature [34] represented a versatile method, leading to high yields of sulfinylimines **5**. (Trimethylsilyl)ethynylmagnesium bromide has already been successfully added to sulfinylimines to produce various *N-*sulfinyl propargylamines in excellent yields and diastereomeric excesses [13,16]. Several *N-*sulfinyl propargylamines (analogoues of valine, phenylglycine and tyrosine) have been prepared using a large excess (4 equivalents) of [(trimethylsilyl)ethynyl]dimethylaluminum as the nucleophile [14,48,52]. The reaction of the sulfinylimines **5** with (trimethylsilyl)ethynyllithium provided the crude intermediates **6**, which were converted into the *N-*sulfinyl propargylamines **7** in high yields and satisfactory diastereomeric ratios by cleaving off the TMS protecting group with TBAF. In previous investigations, the choice of solvent and Lewis acid were controversially discussed [13–14,45–50,52–55]. Nonpolar solvents (DCM < THF < Et_2_O < toluene) have been described to enhance the stability of the transition state, improving the diastereomeric ratio, as well as the reactivity of the nucleophile, resulting in an improved yield [45]. As aldimines have been reported to be rather unreactive electrophiles [53], hard Lewis acids (AlMe_3_ > AlR_3_ > Ti(OiPr)_4_ [14] > BF_3_ > MgBr_2_ > ZnCl_2_ > ZnEt_2_) have been recommended for activation [45]. However, the Lewis acid BF_3_ was shown to give products with inverted configuration of the newly formed chiral center [47]. While several authors obtained increased yields upon addition of AlMe_3_ [1,45–46], others advised against its use as activation agent of aldimines, because side reactions were observed [13,54–55], which will also be further discussed below. We used THF or toluene as solvents and AlMe_3_ or Ti(OiPr)_4_ as activation agent. According to Ellman et al., the diastereoselectivity of the addition to sulfinylimines is controlled by the formation of a cyclic, six-membered, chair-like transition state, which is formed by precoordination of the organometallic reagent to aldimine **5** [45]. This cyclic transition state accounts for the preferred *re*-face attack of the nucleophile at (*S*)-configured *N-*sulfinylimines, which leads to amines with the same configuration as the proteinogenic (*S*)-configured amino acids. This stereoselection was confirmed by various X-ray crystal structures of propargylamines obtained during our investigations like **7a**, **7c–e**, **7s**, **7i–k**, **7q** and triazole **13w** (see Supporting Information File 1). Independent on the substituent, solvent and Lewis acid used, the direction of the nucleophilic attack of (trimethylsilyl)ethynyllithium was always the same, forming the new centers of chirality of all *N-*sulfinyl propargylamines **7** configured as expected. As already described by Ellman et al. [45], the size of the side chain correlates with the result of the reaction. The diastereomeric excesses increased in a size-dependent manner in the order **6a** (alanine) < **6c** (leucine) < **6d** (cyclohexylglycine) (compare Table 1). The yield of the *tert-*leucine derivative **6e** was reduced due to sterically shielding of the imino moiety by the *tert-*butyl group.

The cleavage of the TMS groups of the addition products **6** could be accomplished with TBAF in THF [49–50,56]. Basic conditions, like K_2_CO_3_ as described in the literature [50,57] did not lead to the desired *N-*sulfinyl propargylamines **7**. We assume that strong bases do not only lead to desilylation, but also induce decomposition of the propargylamine system (see below). Kracker et al. recently demonstrated the substitution of the labile *tert*-butylsulfinyl group of compounds **7a–c** by the more versatile Boc protective group in yields of 56–94% [21].

### Synthesis of propargylamines containing electron-withdrawing substituents

**Figure 5:**
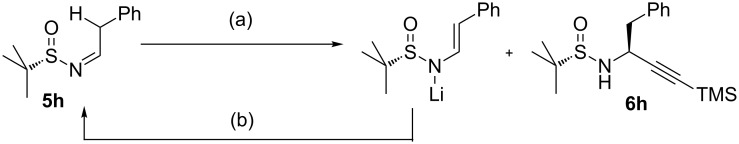
Reaction of *N-*sulfinylimine **5h** with (trimethylsilyl)ethynyllithium. (a) GP-3 or GP-4. (b) Aqueous work-up, H_2_O/H^+^. Deprotonation in benzylic position competes with nucleophilic attack (**5h**/**6h**, 7:3).

Aromatic and carbonyl substituents in the C^β^-position of propargylamines (occurring in analogoues of the amino acids phenylalanine, tyrosine, histidine, tryptophan, aspartic acid and asparagine) increase the acidity of the adjacent protons considerably. In the precursor sulfinylimines **5** of the target propargylamines the acidity of the protons of the methylene moiety is further increased by the electron-withdrawing effect of the adjacent sulfinyl imino moiety. Thus, the nucleophilic addition of (trimethylsilyl)ethynyllithium is competing with deprotonation of the methylene moiety giving rise to the formation of the azaenolate (Figure 5). The resulting anion is reprotonated during aqueous work-up, leading to the starting sulfinylimine **5**.

Benzyl-substituted *N-*sulfinyl propargylamine **6h** was prepared by the addition of (trimethylsilyl)ethynyllithium to *N-*sulfinylimine **5h**. The starting material **5h** and the addition product **6h** were isolated in a 7:3 ratio indicating deprotonation to be the predominant reaction. The phenylalanine analogous *N-*sulfinyl propargylamine **7h** was isolated in only 12% yield (over two steps, referring to imine **5h**) after desilylation with TBAF (Table 2). As the benzylic proton of sulfinylimine **5h** is quite acidic, approach II was not pursued for the synthesis of propargylamines analogous to tyrosine, histidine, tryptophan, and aspartate.

**Table 2:** Synthesis of propargylamines **7** with electron-withdrawing substituents in the side chain.^a^

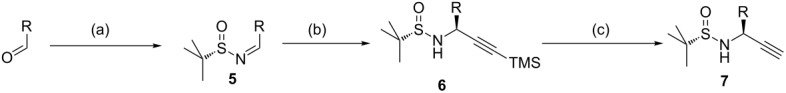							

R	(a)	Yield (**5**)	(b)	Yield (**6**)	(c)	Yield (**7**)	dr

CH_2_Ph	GP-2	64% (**5h**)	GP-4	34% (**6h**)	GP-5	35% (**7h**)	97:3
Ph	GP-1	99% (**5i**)	GP-4	54% (**6i**)	GP-7	66% (**7i**)	95:5
C_6_F_5_	GP-2	88% (**5j**)	GP-4	41% (**6j**)	GP-6	52% (**7j**)	100:0
CF_3_	GP-2^b^	n.i. (**5k**)	GP-4	33%^c^ (**6k**)	GP-6	29% (**7k**)	93:7
CCl_3_	GP-2^b^	87% (**5l**)	GP-4	10% (**6l**)	GP-6	57% (**7l**)	100:0

^a^(a) GP-1: Auxiliary (*S*)-**1**, aldehyde, Ti(OiPr)_4_, 70 °C, 40 min. GP-2: Auxiliary (*S*)-**1**, aldehyde, CuSO_4_, DCM, rt, 3 d. (b) GP-4: (Trimethylsilyl)ethynyllithium, AlMe_3_, toluene, −78 °C to rt. (c) GP-5: TBAF, THF, 0 °C, 3 h. GP-6: KF, 18-crown-6, THF/H_2_O (98:2), 0 °C. GP-7: 1. AgNO_3_, EtOH, 0 °C; 2. KCN, EtOH, HCl. ^b^For the synthesis of **5k**, procedure GP-2 was modified: The aldehyde was distilled under argon atmosphere, condensed onto a mixture of sulfinamide (*S*)-**1** and molecular sieves 4 Å. Toluene was added and the solution was stirred for 48 h. **5k** was directly applied for subsequent reactions and could not be isolated (n.i.). ^c^Yield refers to Ellman’s chiral sulfinamide (*S*)-**1**.

Proteinogenic amino acids do not contain substituents, which additionally increase the acidity of the α-proton. Nevertheless, glycine derivatives with electron-withdrawing substituents like formylglycine [58–59], phenylglycine [60–61] and fluorinated alanine [62–64] have attracted great attention as peptidomimetics, drugs or in the bioorthogonal functionalization of larger peptides.

The trifluoromethyl moiety has been reported to enhance the activity, stability and selectivity of various pharmacologically active compounds [65–66], e.g., trifluridine [67–70], efavirenz [71–73], fluoxetine [74–76] and fluozolate [77]. As the general structure of fluorinated propargylamines occurs in important drugs, like HIV protease inhibitor DPC 961 and its analogues [78–83], particular effort was put on the synthesis of *N-*sulfinyl propargylamine **7k**. Because of the poor electrophilicity of imines **5**, the nucleophilic addition of trifluoromethyl nucleophiles, such as TMS–CF_3_ [84–85] has been described to be inefficient. Still, the asymmetric synthesis of CF_3_-substituted propargylamines has been described, using (*R*)-2-methoxy-1-phenylethan-1-amine as chiral auxiliary [86–88]. However, cleavage of the protective group requires reductive conditions, which also affects the CF_3_ group and the alkyne [86–88].

Various trifluoromethyl-substituted ketones have been converted into *N-*sulfinylimines, which were subsequently transformed into sulfinamides attached to a tertiary C-atom by addition of nucleophiles [89–92]. *N-*Sulfinyl propargylamine **7k** was prepared, following approach II under varying conditions (Table 2). Fluoral hydrate was dehydrated with concentrated sulfuric acid to give trifluoroacetaldehyde by distillation [93].

**Figure 6:**
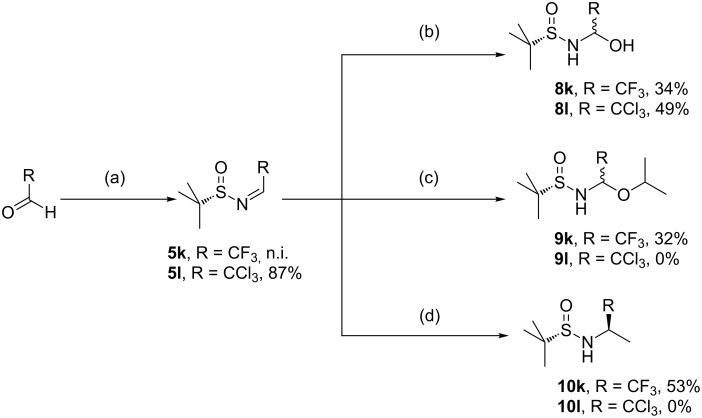
Side reactions observed in the course of the conversion of highly electrophilic sulfinylimines **5**. (a) Sulfinamide (*S*)-**1**, molecular sieves 4 Å, toluene. (b) 1. Dilution with H_2_O, 2. Extraction with DCM. (c) Addition of Ti(OiPr)_4_ prior to the conversion with (trimethylslyl)ethynyllithium [94]. (d) Addition of AlMe_3_ prior to the conversion with (trimethylsilyl)ethynyllithium.

The reaction of trifluoroacetaldehyde with *tert*-butyl sulfinamide (*S*)-**1** in the presence of molecular sieves 4 Å provided the sulfinylimine **5k**, which could not be isolated due to its extremely high electrophilicity. Instead, immediate hydrolysis occurs upon contact with water forming hemiaminal **8k** (Figure 6), as already observed by Truong et al. [94]. This frequently observed hemiaminal was isolated by column chromatography and recrystallized from EtOAc.

**Figure 7:**
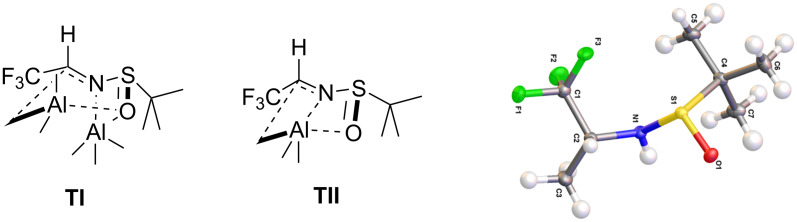
a) Possible transition states **TI** and **TII** for the transfer of the methyl moiety from AlMe_3_ to the imino moiety. b) X-ray crystal structure analysis of the methyl transfer product **10k**.

Distillation of imine **5k** has been reported to provide a very low yield (22% [94]). The reaction of hemiaminal **8k** with (trimethylsilyl)ethynyllithium in the presence of the strong Lewis acid AlMe_3_ has been proposed by Truong et al. [94], but was reported to give very poor yields and low diastereoselectivity. In contrast to the argumentation of Xiao et al., who strongly recommended hard Lewis acids for the reaction of sulfinylimines with various ethynyllithium reagents [95], the crude imine **5k** predominantly reacted with the Lewis acids Ti(OiPr)_4_ and AlMe_3_ and gave only low yields of the desired *N-*sulfinyl propargylamine **6k**. The reaction of crude **5k** in the presence of the Lewis acid Ti(OiPr)_4_ afforded the N/O-acetal **9k**, whereas the attempt of activation with AlMe_3_ provided the methylated sulfinamide **10k**. Both side products were isolated as colorless crystalline solids. It is assumed, that the undesired side products **9k** and **10k** were formed by a ligand transfer from the Lewis acids to imine **5k**. Nucleophilic substitution of N/O-acetal **9k** with two equivalents of (trimethylsilyl)ethynyllithium, in analogy to the conversions reported by Kuduk et al. [96], remained unsuccessful. The (*S*)-configuration of the newly generated chiral center of amine **10k** was determined by X-ray structure analysis (Figure 7), suggesting two possible transition states for the ligand transfer.

Whereas transition state **TII** requires only one equivalent of AlMe_3_, two equivalents of AlMe_3_ are involved in transition state **TI**. As in this case almost quantitative conversion of AlMe_3_ was observed, transition state **TII** seems to be more probable. Still, more complex coordination geometry (hexagonal or dinuclear complexes) of the Lewis acid are probable as well and hardly depend on the stoichiometry. In the conversion of highly reactive sulfinylimines like **5k**, best results were obtained in the absence of a Lewis acid. Analogous investigations with chloral hydrate instead of fluoral hydrate showed that the trichloromethylimine **5l** also forms a hemiaminal **8l** upon contact with water, but does not undergo comparable side reactions with Ti(OiPr)_4_ or AlMe_3_. Sulfinylimine **5l** appears to be a weaker electrophile, which is attributed to the lower electronegativity of Cl compared to F and to the larger size of the CCl_3_ group compared to the CF_3_ moiety, sterically shielding imine **5** against nucleophilic attack.

Desilylation of *N-*sulfinyl propargylamine **6i** with TBAF (GP-5) resulted in decomposition instead of formation of the free alkyne **7i**. In order to obtain the free terminal alkyne **7i**, a milder desilylation procedure was required. Therefore, AgNO_3_ and KCN (GP-7) were used for the desilylation of **6i** to afford the free alkyne **7i** in 66% yield. *N-S*ulfinyl propargylamines **6k** and **6j**, with perfluorinated side chains, required even milder desilylation conditions. KF in the presence of 18-crown-6 (GP-6) provided the free alkynes **7k** and **7j** in 29% and 52% yield, respectively (results are collected in Table 2).

**Figure 8:**
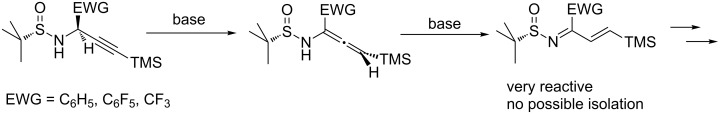
Base-induced rearrangement of propargylamines bearing electron-withdrawing substituents.

Desilylation of alkynes **6k** and **6j** under alkaline conditions with K_2_CO_3_, in analogy to the description of Vasella et al. [57] leads to instant decomposition of the starting material in a base-promoted propargyl–allenamide isomerization. According to our hypothesis, basic fluoride leads to deprotonation in the C^α^-position of **6i**, **6k** and **6j**, inducing an alkyne rearrangement to form an allene, which rearranges further to provide an α,β-unsaturated imine (Figure 8) [97].

**Figure 9:**
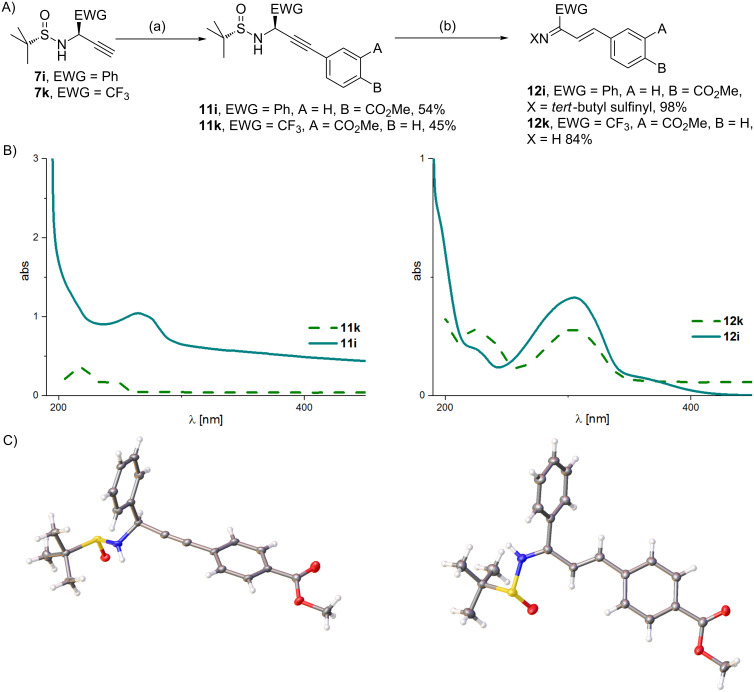
Base-catalyzed rearrangement of propargylamines **11** to α,β-unsaturated imines **12**. A) Reaction scheme: (a) methyl 4-iodobenzoate (for the conversion of **7i** to **11i**, 1.6 equiv) or methyl 3-iodobenzoate (for the conversion of **7k** to **11k**), DIPEA (6 equiv), THF, Cl_2_Pd(PPh_3_)_2_, CuI, room temperature, 2 h (GP-9). (b) Piperidine/THF (1:3), 0 °C, 30 min (conversion of **11i** to **12i**). Or LiOH (3 equiv), MeOH/H_2_O (2:1), 0 °C, 30 min. B) UV–vis spectra of propargylamines **11** and α,β-unsaturated imines **12**. C) X-ray crystal structure analysis of propargylamine **11i** and α,β-unsaturated imine **12i**.

One target application of propargylamines **7** is the Sonogashira cross-coupling with halogenated benzoates, forming the scaffold of versatile peptidomimetics **11**. According to the proposed rearrangement of alkyne **7** (Figure 9), the choice of base is crucial for such cross-coupling reactions. To get a better understanding of the reactivity of propargylamines, the stability and propensity for base induced rearrangement of **11** were investigated (Figure 9).

The ester substituted compounds **11i** and **11k** were obtained by Sonogashira cross-coupling of the terminal alkynes **7i** and **7k** with methyl 4- and 3-iodobenzoate, respectively. As the *tert*-butylsulfinyl group can be cleaved off under mild, acidic conditions [1,25–26], it provides a versatile protective group for the amine during the conversion of the alkyne. Additionally, it’s chirality indicates epimerization and, for example in the Pauson–Khand reaction [14], allows the determination of the stereoselectivity of asymmetric conversions by simple ^1^H NMR experiments. Consequently it was not removed prior to the rearrangement experiments.

Treatment of **11i** and **11k** with the mild base piperidine led to the formation of α,β-unsaturated sulfinylimines **12i** and **12k**. The structures of the rearranged products were proven unequivocally by ^1^H and ^13^C NMR spectroscopy and X-ray crystal structure analysis.

Due to the extended π-system of the α,β-unsaturated imines **12i** and **12k**, the progress of the propargylamide–allenylamide rearrangement that eventually leads to the formation of the α,β-unsaturated imines by tautomerism could be easily monitored by UV–vis spectroscopy. The reaction mixture turned brightly yellow when treated with bases like piperidine. The X-ray crystal structures of **11i** and **12i** confirm unequivocally the structure of the products and thus the postulated two-step rearrangement. Both, the rearrangement of the alkyne to the allene and the subsequent tautomerism to the α,β-unsaturated imine are not reversible. Reversibility of the rearrangement would be fundamental for racemization of propargylamines, which is consequently improbable. However, even in the presence of strong bases like KO*t*-Bu or LDA, the propargylamide–allenylamide rearrangement could never be observed for propargylamines without an acidifying C^α^-substituent.

### Synthesis of propargylamines containing polar or acidic functional groups

The synthesis of propargylamines with polar substituents to mimic polar amino acids such as serine (alcohol), cysteine (thiol) or glutamine (carboxamide) requires special protective groups (Table 3).

**Table 3:** Preparation of *N-*sulfinyl propargylamines **7** with polar and acidic functional groups in the side chains.^a^

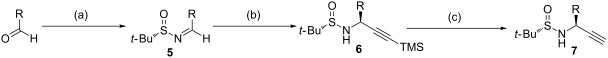					

R	(a)	Yield (**5**)	(b), (c)	Yield (**7**)^b^	dr

CH_2_OCPh_3_	GP-2	47% (**5m**)	GP-4, GP-6	0% (**7m**)^c^	n.d.
CH_2_OBn	GP-2	81% (**5n**)^d^	GP-3, GP-5	4% (**7n**)^e^	95/5
CH_2_OAll	GP-1	24% (**5o**)	GP-4, GP-5	31% (**7o**)	93/7
CH_2_SBn	GP-2	83% (**5p**)^d^	GP-3, GP-5	31% (**7p**)^f^	93/7
(CH_2_)_3_CN	GP-2	80% (**5q**)	GP-4, GP-6	43% (**7q**)	95/5
(CH_2_)_2_CO_2_Bn	GP-2	79% (**5r**)	GP-4, GP-5	0% (**7r**)^c^	n.d.
(CH_2_)_2_CO_2_*t*-Bu	GP-2	64% (**5s**)	GP-4, GP-6	47% (**7s**)	93/7
(CH_2_)_3_OX^f^	GP-1	90% (**5t**)	GP-3, GP-5	27% (**7t**)	93/7
(CH_2_)_3_Cl	GP-1	85% (**5u**)	GP-4, GP-5	0% (**7u**)^c^	n.d.
(CH_2_)_4_N_3_	GP-2	52% (**5v**)	^f^		
(CH_2_)_3_N_3_	GP-2	84% (**5w**)	^f^		

^a^(a) GP-1: Auxiliary (*S*)-**1**, aldehyde, Ti(OiPr)_4_, 70 °C, 40 min. GP-2: Auxiliary (*S*)-**1**, aldehyde, CuSO_4_, DCM, rt, 3 d. (b) GP-4: (Trimethylsilyl)ethynyllithium, AlMe_3_, toluene, −78 °C to rt. (c) GP-5: TBAF, THF, 0 °C, 3 h. GP-6: KF, 18-crown-6, THF/H_2_O (98:2), 0 °C. ^b^Yields of **7** refer to imine **5** (two steps). ^c^Compounds **7m**, **7r** and **7u** could not be isolated and the diastereoselectivity could not be determined (n.d.). ^d^Further reactions in Table 4. ^e^(*R*)-Configured Ellman’s sulfinamide (*R*)-**1** was applied. Hence, the mirror images of **5–7n,p** were obtained. ^f^**5t**, X = TBDMS. **7t**, X = H.

The cyano moiety was used as precursor of the carboxamide moiety of glutamine, since the cyano group is stable in the presence of nucleophiles and strong bases. The synthesis started with the Kolbe nitrile synthesis of 4-iodobutan-1-ol with NaCN. Performing this transformation in DMSO provided the desired 5-hydroxypentanenitrile and THF in the ratio 5:2 (monitored by ^1^H NMR spectroscopy, see Supporting Information File 1). Next, 4-cyanobutan-1-ol was oxidized in a Swern oxidation and the resulting aldehyde was condensed with *tert*-butylsulfinamide (*S*)-**1**, according to GP-2. The reaction of sulfinimine **5q** with (trimethylsilyl)ethynyllithium led to sulfinamide **6q**. In this case, the Lewis acid AlMe_3_, which could also react with the cyano moiety, was omitted. Finally, cleavage of the TMS group was achieved under mild conditions with KF and 18-crown-6 (GP-6), to yield *N-*sulfinyl propargylamine **7q** as an analogue of the non-proteinogenic amino acid 5-cyano-L-norvaline. Formation of a glutamine analogous side chain by hydrolysis of the nitrile function to an amide remained unsuccessful so far.

Propargylamines with diversely protected alcohol functionalities in the side chain were obtained by approach II. The application of an allyl ether, starting from 2-allyloxyacetaldehyde to form **7o** and a benzoate, starting from formylmethyl benzoate to form **7n** (Table 3) turned out to be most convenient. In contrast to labile TMS ethers, the sterically more demanding *tert*-butyl dimethylsilyl ether was successfully applied as protective group to obtain the homologated serine analogous propargylamine **7t** similar to the description by Verrier et al. [47,55], starting from TBDMS-protected oxybutanal. Treatment of alkyne **6t** with TBAF leads to simultaneous cleavage of both silyl groups. Although sterically shielding protective groups have proven convenient, the trityl group turned out to be inefficient to generate a serine-analogous propargylamine. Trityl-protected imine **5m** immediately decomposed, when treated with (trimethylsilyl)ethynyllithium.

The preparation of glutamic acid-analogous propargylamines failed when the acid functionality was protected as a benzyl ester. Fortunately, the sterically more demanding *tert*-butyl ester was stable and gave high yields of **7s**. The synthesis started from the aldehyde *tert-*butyl-4-oxobutanoate, which could be easily obtained from succinic anhydride [44,98–99]. Selective cleavage of the *tert*-butyl group was not yet accomplished without affecting the *tert*-butyl sulfinamide protection group of the amine.

A cysteine-analogous alkyne could be synthesized starting from benzylmercaptan. Unfortunately, only extremely low yields were achieved and *N-*sulfinyl propargylamine **7p** is not stable under the conditions, which are necessary to cleave the thioether [100–101].

### Synthesis of propargylamines with basic functional groups in the side chain

Very often, basic amino acids, like lysine or arginine are found in the catalytic center of enzymes. Therefore, propargylamines mimicking these basic amino acids are of particular interest to be incorporated in peptidomimetics. The exchange of basic amino acids has already been performed to develop potent enzyme inhibitors [102–104]. In order to introduce side chains with basic amino moieties into propargylamines, these have to be protected against nucleophiles, bases and deprotonation.

According to the approach of Ye et al., a 3-bromopropyl side chain was used, which was converted to the azide and further transformed into an aminoalkyl group by Staudinger reduction [16]. In order to follow a more convergent approach and to avoid nucleophilic substitution of the halide at a late stage, we decided to start the synthesis with 4-azidobutanal, which was prepared by opening THF with iodine and NaBH_4_, nucleophilic substitution by sodium azide and Swern oxidation of the alcohol.

4-Azidobutanal was converted with chiral sulfinamide (*S*)**-1** into *N-*sulfinylimine **5w**, which was reacted with (trimethylsilyl)ethynyllithium. However, following the usual procedure GP-4 with warming up the reaction mixture to room temperature before quenching with water led to the formation of triazole **13w**, which was isolated in 56% yield (Table 4). The target propargylamine **6wx** was obtained in only 19% yield. Cleavage of the TMS group with KF and 18-crown-6 at 0 °C provided azide **7wx** in only 22% yield, because triazole **14w** was formed in a side reaction. Compound **7wx** was converted into triazole **14w** even upon standing at room temperature in CDCl_3_ (monitored by ^1^H NMR spectroscopy, see Supporting Information File 1). Formation of triazoles **13w** and **14w** was unexpected, because the uncatalyzed Huisgen reaction usually requires higher temperature or activation by electron-withdrawing substituents at the alkyne or electron-donating substituents at the azide, respectively [105–106]. As none of these requirements are met in the case of **6wx** and **7wx**, it is assumed, that the preorientation of the azide and the alkyne together with the formation of an energetically favored six-membered ring are the driving forces. As hexose scaffolds similar to **13w** and **14w** have been successfully applied as inhibitors of β-glucosidases [107] and hexosamidases [108], this intramolecular Huisgen reaction could be exploited to develop novel enzyme inhibitors.

**Table 4:** Synthesis of lysine, ornithine and arginine-analogous propargylamines **7vy**, **7wy** and **7x** and discovery of an unexpected intramolecular low-temperature *Huisgen* reaction.

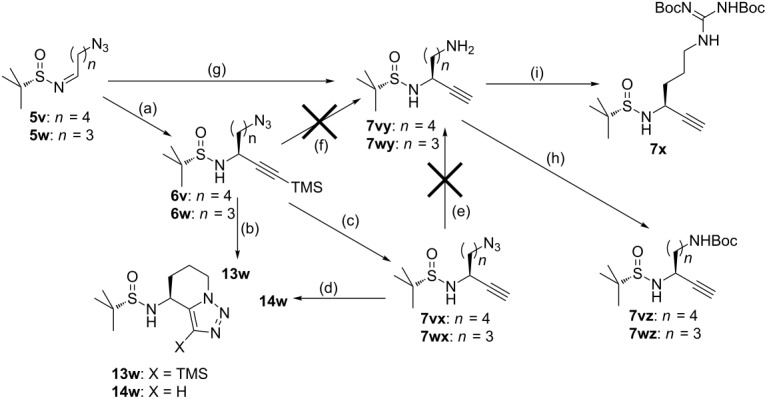				

Starting material	Reaction conditions	Product	Yield	dr

**5v**	(a) (trimethylsilyl)ethynyllithium, Ti(OiPr)_4_, THF, −78 °C (GP-3)	**6v**	58%	100:0
**5w**	(a) (trimethylsilyl)ethynyllithium, Ti(OiPr)_4_, THF, −78 °C. (GP-3)	**6w**	14%	n.d.
**6v**	(b) (trimethylsilyl)ethynyllithium, AlMe_3_, toluene, −78 °C (GP-4)	**13w**	56%	100:0
**6v**	(c) TBAF (2 equiv), THF, 0 °C, 4 h (GP-5)	**7vx**	58%	74:26
**6w**	(c) TBAF (2 equiv), THF, 0 °C, 4 h (GP-5)	**7wx**	19%	96:4
**7wx**	(d) CDCl_3_, 7 d, rt.	**14w**	66%	96:4
**7vx**	(e) 1. PPh_3_, CuSO_4_, THF; 2. H_2_O.	**7vy**	0%	n.d.
**7wx**	(e) 1. PPh_3_, CuSO_4_, THF; 2. H_2_O.	**7wy**	0%	n.d.
**6v**	(f) 1. NaBH_4_, CuSO_4_, THF; 2. H_2_O.	**7vy**	0%	n.d.
**6w**	(f) 1. NaBH_4_, CuSO_4_, THF; 2. H_2_O.	**7wy**	0%	n.d.
**5v**	(g) 1. (trimethylsilyl)ethynyllithium, toluene, −78 °C, 3 h; 2. PPh_3_ (4 equiv), THF, −78 °C; 3. H_2_O, rt, 2 h.	**7vy**	68%	91:9
**5w**	(g) 1. (trimethylsilyl)ethynyllithium, toluene, −78 °C, 3 h; 2. PPh_3_ (4 equiv), THF, −78 °C; 3. H_2_O, rt, 2 h.	**7wy**	86%	80:20
**7vy**	(h) 1. Boc_2_O (2 equiv), THF/H_2_O (1:1), NaHCO_3_ (3 equiv); 2. imidazole, 4 h.	**7vz**	45%	91:9
**7wy**	(h) 1. Boc_2_O (2 equiv), THF/H_2_O (1:1), NaHCO_3_ (3 equiv); 2. imidazole, 4 h.	**7wz**	63%	90:10
**7wy**	(i) BocH*N-*C(=NBoc)SMe (1 equiv), DCM, rt, 3 d.	**7x**	79%	93:7

In order to get access to propargylamines with the side chains of lysine, ornithine and arginine, azide **7wx** should be reduced. However, all attempts to reduce the isolated azide **7wx** with NaBH_4_ or PPh_3_ failed, due to the competing triazole formation. Therefore, instant reduction of in situ prepared **6wy** was envisaged. The nucleophilic addition of (trimethylsilyl)ethynyllithium to *N-*sulfinylimine **5w** was monitored by analytical HPLC. After complete conversion, PPh_3_ was added directly to the reaction mixture at −78 °C. After addition of water and stirring for two hours, the primary amine **7wy** was isolated in 86% yield. This procedure led to the ornithine analogous propargylamine **7wy**, which was reacted with Boc-protected *S*-methylisothiourea to yield the arginine analogous propargylamine **7x** in a yield of 79%. During the decomposition of the azide in the Staudinger reaction, the TMS group at the alkyne was cleaved off simultaneously. Cleavage of TMS groups with PPh_3_ under similar conditions has not been reported so far. Therefore, an intramolecular mechanism is postulated, in which the iminophosphorane, formed by the reaction of PPh_3_ with azide **6wy**, coordinates to the silyl group. This intramolecular coordination facilitates the fast hydrolytic cleavage of the silyl group during aqueous work-up. This PPh_3_-induced TMS cleavage could also be successfully applied in the synthesis of the lysine analogous propargylamine **7vy** from **5v** but could never be reproduced in the formation of other propargylamines, like the *tert*-butyl-substituted compound **7e**, under identical reaction conditions. The amine groups of **7wy** and **7vy** were protected with a Boc group to give propargylamines **7wz** and **7vz**.

## Conclusion

Ellman’s chiral sulfinamide has been successfully used for the asymmetric synthesis of enantiomerically pure propargylamines. An array of 24 diastereomerically pure *N*-sulfinyl propargylamines has been prepared, bearing side chains in α-position, which are analogous or similar to amino acid side chains. In general, various aldehydes are condensed with Ellman’s chiral sulfinamide. Diastereoselective *re*-face addition of (trimethylsilyl)ethynyllithium to the (*S*)-configured sulfinimines **5** gives the corresponding *N-*sulfinyl propargylamines **6**. Cleavage of the TMS group with TBAF or KF × 18-crown-6 provides *N-*sulfinyl propargylamines **7** with a terminal alkyne.

Propargylamines with aliphatic side chains were obtained in good yields, depending on the size of the C^α^ substituent. Various polar and basic substituents in the side chain can be introduced using masked or protected functionalities. Side chains with amino groups were introduced masked as azide. For this purpose, the unprecedented intramolecular Huisgen reaction has to be suppressed. Electron-withdrawing substituents in the C^β^-position could not be used by this approach. Electron-withdrawing substituents in the C^α^-position induced an irreversible alkyne–allene-α,β-unsaturated imine rearrangement under mild basic conditions, which makes an alkaline racemization of propargylamines improbable. Altogether, a large set of propargylamines with various amino acid similar substituents are available for application in peptidomimetics and some knowledge on the reactivity of propargylamines has been contributed.

## Supporting Information

File 1 Details about the experiments, methods and materials, the X-ray crystal structures and NMR spectra.
