# Supplementary material for: Asymmetric synthesis of propargylamines as amino acid surrogates in peptidomimetics
Source: Beilstein J Org Chem. 2017 Nov 15;13:2428–41. doi: 10.3762/bjoc.13.240 (PMC5704752; doi:10.3762/bjoc.13.240)
Supplement: File 1 — Details about the experiments, methods and materials, the X-ray crystal structures and NMR spectra. [file Beilstein_J_Org_Chem-13-2428-s001.pdf]

Supporting Information

for

Asymmetric synthesis of propargylamines as amino acid surrogates in peptidomimetics

Matthias Wünsch, David Schröder, Tanja Fröhr, Lisa Teichmann, Sebastian Hedwig, Nils Janson, Clara Belu, Jasmin Simon, Shari Heidemeyer, Philipp Holtkamp, Jens Rudlof, Lennard Klemme, Alessa Hinzmann, Beate Neumann, Hans-Georg Stammler and Norbert Sewald*

Address: Organic and Bioorganic Chemistry, Department of Chemistry, Bielefeld University, Universitätsstraße 25, D-33615 Bielefeld, Germany

Email: Norbert Sewald - norbert.sewald@uni-bielefeld.de

* Corresponding author

Details about the experiments, methods and materials, the X ray crystal structures and NMR spectra

Table of contents

General methods and materials S3

Abbreviations S6

General Procedures S7

Synthesis S11

Ethyl (3-Hydroxypropynyl)-benzoate derivatives **1** S11

Ethyl (3-Oxopropynyl)-benzoate derivatives **2** S11

Ethyl (*tert*-Butylsulfinyl)imino)propynyl)benzoate derivatives **3** S12

Ethyl benzoate substituted propargylamine derivatives **4** S13

Chiral aldimines **5** S16

TMS protected propargylamines **6** S35

Propargylamines **7** S43

Hydrolysis of imine **5** forms hemiaminal **8** S56

Side-product **9k** of the conversion of **5k** with GP-3 .... S57

Side-product **10k** of the conversion of **5k** with **GP-4** S58

Sonogashira cross-coupling products as peptidomimetics **11** S59

Rearrangementproducts: α,β-unsaturated imines **12** S60

Intramolecular Huisgen reaction of **6w** gives triazole **13w** S61

Intramolecular Huisgen reaction of **7wx** gives triazole **14w** S62

References S63

X-ray structure analysis S65

Spectra S100

General methods and materials

If not mentioned differently, all reagents and solvents were purchased from commercial sources and applied without further purification. THF was kept over KOH before being dried with sodium/benzophenone under reflux and was freshly distilled before use. Toluene was predried over $CaCl_2$, then dried over sodium under reflux and distilled freshly before use. DCM used for synthesis was predried over $CaCl_2$, dried over CaH_2 under reflux and distilled freshly before use. DMSO was dried under reflux over CaH_2, distilled and stored over molecular sieves (4 Å) until use. DCM, EtOAc, PE and Et_2O used for aqueous work-ups or column chromatography were purchased in technical grade and distilled prior to application.

Schlenk conditions: If not mentioned differently, the reactions were carried out under exclusion of moisture and oxygen in dried glassware under argon atmosphere. The argon gas was supplied from Linde (quality 4.0) and passed through a column filled with phosphorpentoxide (sicapent®, *Merck*) before use.

Solvents were removed on a rotational evaporator at 40 °C and appropriately reduced pressure. Solvent residues were removed at rt and 0.001–0.1 mbar.

For column chromatography, Silica gel 60, 40–63 μm (*Merck*) was used. The eluents and their proportions are individually noted. Thin layer chromatography (TLC) was executed using silica gel 60 coated aluminium sheets with fluorescence indicator F254 (Merck). Spots were identified using different stains, such as $KMnO_4$, iodine, ninhydrin or UV light with a wavelength of λ = 254 nm or λ = 366 nm.

Preparative HPLC (Thermo Separation Products): Equipment: UV detector: UV1000; pump: Thermo Separation Products P4000; Method: column: Thermo Scientific Hypersil Gold (8 μm), 250 × 21.2 mm cartridge; flow rate: 10.00 mL min^{-1}; injection volume: 1.00 mL; detection at λ = 254 nm; solvents: A: water/acetonitrile/trifluoroacetic acid (94.9:5:0.1); B: water/acetonitrile/trifluoroacetic acid (5:94.9:0.1). gradient elution: (A %): 0–1 min: 100%, 1-30 min: gradient from 100% to 0%, 30–44 min: 0 %, 44–45 min: gradient from 100% to 0%.

Melting points: Melting points were determined using a Büchi 540 melting point apparatus and are uncorrected.

Optical rotation was measured on a DIP-360 (Jasco) polarimeter with a sodium vapour light source at a specific given temperature. A quartz cell with a path length of 10 cm was used. The average of ten single measurements divided by the concentration in units of g/mL and the

pathlength (1 dm). The sample concentration and solvent is given in c = g/100 mL in parentheses.

Nuclear magnetic resonance (NMR) spectra were recorded on Bruker Avance 300 (300.13 MHz for ^{1}H, 282.38 MHz for ^{19}F, 75.48 MHz for ^{13}C) or DRX 500 (499.87 MHz for ^{1}H, 470.43 MHz for ^{19}F, 125.70 MHz for ^{13}C) or Avance 500 (500.01 MHz for ^{1}H, 125.74 MHz for ^{13}C) or Avance 500 HD (500.20 MHz for ^{1}H, 125.79 MHz for ^{13}C) or Avance 600 (600.13 MHz for ^{1}H, 564.63 MHz for ^{19}F, 150.92 MHz for ^{13}C). Chemical shifts (δ), given in the experimental section, are reported in ppm relative to TMS (δ_{TMS} = 0 ppm) and referenced to the solvent residue signals as internal standard: δ_i [ppm]: $CHCl_3$ (δ 7.26 ppm (^{1}H NMR) and δ 77.2 ppm (^{13}C NMR)) and CHD_2OD (δ 3.31 ppm (^{1}H NMR) and δ 49.0 ppm (^{13}C NMR)) and $D_2HCSOCD_3$ (δ 2.50 ppm (^{1}H NMR) and δ 39.5 ppm (^{13}C NMR)). Coupling constants (J) are reported in Hertz (Hz) with 0.05 Hz resolution. Multiplicities are described as singlet (s), doublet (d), triplet (t) quartet (q) or multiplet (m). The assignments of ^{13}C and ^{1}H NMR signals were supported by 2D NMR techniques (COSY, HMQC, HMBC).

MS: Nano-ESI mass spectra were recorded using an Esquire 3000 ion trap mass spectrometer (Bruker Daltonik GmbH, Bremen, Germany) equipped with a standard nano-ESI source. Samples were introduced by static nano-ESI using *in-house* pulled glass emitters. Nitrogen served both as the nebuliser gas and the dry gas. Nitrogen was generated by a Bruker nitrogen generator NGM 11. Helium served as cooling gas for the ion trap and collision gas for MS^n experiments.

ESI mass spectra were recorded using an Agilent 6220 time-of-flight mass spectrometer (Agilent Technologies, Santa Clara, CA, USA) in extended dynamic range mode equipped with a Dual-ESI source, operating with a nitrogen generator NGM 11. Samples were introduced with a 1200 HPLC system consisting of an autosampler, degasser, binary pump, column oven and diode array detector (Agilent Technologies, Santa Clara, CA, USA) using a C18 Hypersil Gold column (length: 50 mm, diameter: 2.1 mm particle size: 1.9 μm) with a short gradient (in 4 min from 0% B to 98% B, back to 0% B in 0.2 min, total run time 7.5 min) at a flow rate of 250 μL min^{-1} and column oven temperature of 40 °C. HPLC solvent A consisted of water, acetonitrile and formic acid (94.9:5:0.1), solvent B of water, acetonitrile and formic acid (5:94.9:0.1). The mass axis was externally calibrated with ESI-L Tuning Mix (Agilent Technologies, Santa Clara, CA, USA) as calibration standard.

Elemental analyses were performed on an Element Analyser EURO EA.

IR spectra were recorded as neat samples on a FT-IR spectrophotometer Nicolet 380 (Thermo Scientific) equipped with ATR technique (smart orbit).

Analytical HPLC (Thermo Scientific Accela): Equipment; UV detector: Thermo Seperation Products UV6000LP; pump: Thermo Separation Products P4000; autosampler: Thermo Separation Products AS100, Method: column: Jupiter 5 C18 Fa. Phenomenex, 250 × 4.60 mm cartridge; flow rate: 1.00 mL/min; injection volume: 0.2 μL; detection at λ = 254 nm; solvents: A: water/acetonitrile/trifluoroacetic acid (95.9:5:0.1); B: water/acetonitrile/trifluoroacetic acid (5:95.9:0.1). Gradient elution: (A, method 1): 0–9 min: gradient from 100% to 0%, 9–12 min: 0%, 12–13 min: gradient from 0% to 100%. (A, method 1): 0–4.5 min: gradient from 100% to 0%, 4.5–7 min: 0%, 7–8 min: gradient from 100% to 0%.

Crystal data were collected on an Agilent SuperNova diffractometer with Cu Kα radiation except for **12k** and **10k**, where Mo Ka radiation was used. The crystals were kept at 100.0(3) K during data collection. Using Olex2 [1] the structures were solved and refined with the ShelX program package [2] using direct methods and least-squares minimization. Details of the X-ray investigation are given in SI. CCDC 1566791 (**7a**), CCDC 1566792 (**7c**), CCDC 1566793 (**7d**), CCDC 1566794 (**e**), CCDC 1566795 (**7i**), CCDC 1566796 (**7j**), CCDC 1566797 (**7k**), CCDC 1566798 (**7q**), CCDC 1566799 (**7s**), CCDC 1566800 (**10k**), CCDC 1566801 (**11i**), CCDC 1566802 (**12i**), CCDC 1566803 (**12k**) and CCDC 1566804 (**13w**) contain the supplementary crystallographic data for this paper. These data can be obtained free of charge from The Cambridge Crystallographic Data Centre via www.ccdc.cam.ac.uk/data_request/cif.

Abbreviations

ACN	Acetonitrile
All	Allyl
ar	Aryl
Bn	Benzyl
Boc	*tert*-Butoxycarbonyle
Bu	Butyl
cy	cyclohexyl
d	doublet
DCM	Dichlormethane
DMSO	Dimethylsulfoxide
dr	Diastereomeric Ratio
ee	Enantiomeric Excess
eq	Equivalents
ESI	Electrospray Ionization
Et	Ethyl
Et_2O	Diethyl Ether
EtOAc	Ethylacetate
FT	Fourier Transform
h	Hours
HPLC	High Performance Liquid Chromatography
IR	Infrared
M	Molar [mol L^{-1}]
Me	Methyl
MeOH	Methanol
MHz	Megahertz
m	Multiplet
mp	Melting Point
MS	Mass Spectrometry
NMR	Nuclear Magnetic Resonance
Ph	Phenyl
q	Quartet
rt	Room Temperature
s	Singlet
TFA	Trifluoracetic Acid
THF	Tetrahydrofurane
TLC	Thin Layer Chromatography
TMS	Trimethylsilyl
t	Triplet
UV	Ultra Violet
Vis	Visible

General Procedures

Condensation of aldehydes with Ellman's chiral sulfinamide to form imines ***5***

GP-1: *tert*-Butylsulfinamide **(*S*)-1** or **(*R*)-1** (1 equiv) was dissolved in freshly distilled aldehyde (1 equiv) and $Ti(OEt)_4$ (2 equiv) was added in one portion. The slightly yellow suspension turned brightly orange upon heating for 40 min (approximately 60 °C) under reflux conditions. After cooling to rt, the suspension was diluted with EtOAc (40 mL) and brine (1 mL) was added dropwise leading to the formation of a colourless precipitate. The solid was filtered through a pad of celite and washed with EtOAc (200 mL). Evaporation of the solvent in vacuo yielded the aldimine (**5b**, **5c**, **5h**, **5i**, **5t**) in pure form to be converted without further purification. Typical reactions were carried out on a scale of 1–3 g of *tert*-Butylsulfinamide **(*S*)-1** or **(*R*)-1**. Analogous reaction conditions have already been described by Ellman et al. and Yus et al. [3–5].

GP-2: *tert*-Butylsulfinamide **(*S*)-1** or **(*R*)-1** (1 equiv) was dissolved in CH_2Cl_2 (1 M solution) and freshly prepared aldehyde (1.2 equiv), as well as dried $CuSO_4$ (1.5-2.0 equiv) was added in one portion. The colourless reaction mixture was stirred for 72 h at rt. After complete conversion of the sulfinamide (monitored by TLC), the suspension was diluted with a $KHSO_4$ solution (5%). The aqueous layer was separated, extracted twice with CH_2Cl_2 and the combined organic layers were dried over Na_2SO_4. Evaporation of the solvent in vacuo yielded the desired sulfinylimines (**5d-g**, **5j-l**, **5n-q**, **5s**, **5v**, **5w**). In some cases, purification by column chromatography was necessary. Typical reactions were carried out on a scale of 0.2–3 g of *tert*-Butylsulfinamide **(*S*)-1** or **(*R*)-1**. Analogous reaction conditions have already been described by Ellman et al. [6].

Diastereoselective nucleophilic addition of trimethylsilylethynyl lithium to chiral sulfinimines ***5*** *to form TMS-protected alkynes* ***6***.

GP-3: At −78 °C, *n*-BuLi (1.6 M in *n*-hexane, 1.6 equiv) was added dropwise to a 0.85 M solution of ethynyltrimethylsilane (1.5 equiv) in THF. After 2 h, a 0.35 M solution of aldimine **5** (1.0 equiv) and $Ti(OiPr)_4$ (0.5 equiv) in THF was added to the reaction mixture over a period of 30 min. After complete conversion (approximately 2 h, monitored by TLC), the reaction mixture was allowed to warm up to rt. Subsequently, a saturated aqueous solution

of NH_4Cl was added until no further precipitate was formed. The colourless solid was filtered through a pad of celite, and the pad was washed with EtOAc (200 mL). The filtrate was dried over Na_2SO_4 and the solvent evaporated under reduced pressure. The crude product was directly applied for desilylation. This method was applied for the synthesis of **6a-d**, **6n** and **6t**. Typical reactions were carried out on a scale of 0.5–3 g of imine **5**. Analogous reaction conditions have already been described by Ellman et al. and Tartakovski et al. [7–9].

GP-4: At −78 °C, *n*-BuLi (1.6 M in *n*-hexane, 1.6 equiv) was added dropwise to a 0.85 M solution of ethynyltrimethylsilane (1.5 equiv) in toluene. After 2 h, a solution of aldimine **5** (1.0 equiv) and $AlMe_3$ (0.5 equiv) in toluene (0.35 M sol.) was added to the reaction mixture over a period of 30 min. After complete conversion (approximately 2 h, monitored by TLC), the reaction mixture was allowed to warm up to rt. The reaction mixture was diluted with a solution of $KHSO_4$ (5%), the organic layer was separated and washed with another portion of $KHSO_4$ solution (5%). The combined aqueous layers were extracted with Et_2O (4 × 50 mL). The combined organic layers were dried over Na_2SO_4 and the solvent was evaporated under reduced pressure. The crude alkyne **6** was directly applied for desilylation. This method was used for the synthesis of **6e-j**, **6p**, **6o**. Typical reactions were carried out on a scale of 0.2–5 g of imine **5**. Analogous reaction conditions have already been described by Ellman et al., Yus et al., Tan et al., Lin et al. and Hou et al. [9–14].

*Desilylation of alkynes to yield propargylamine **7***

GP-5: In this procedure, no *Schlenk*-conditions were applied. TMS protected alkyne **6** (1.0 equiv) was dissolved in THF to give a 0.2 M solution and a 1 M solution of TBAF in THF (2 equiv) was added dropwise at 0 °C. The reaction mixture was stirred for 2 h at 0 °C and for another 2 h at rt. After complete conversion (monitored by TLC), the reaction mixture was diluted with a saturated NH_4Cl solution. The emulsion was extracted with Et_2O (4 × 50 mL) and the combined organic layers were dried over Na_2SO_4. After evaporation of the solvent, the crude product was purified by column chromatography (in most cases EtOAc/PE, 1:2 or 1:1). The diastereomerically pure propargylamines **7** were isolated by recrystallization. This reaction procedure was used for the synthesis of **7a-e**, **7h**, **7n-p** and **7t**. Typical reactions were carried out on a scale of 0.4-5 g of TMS protected alkyne **6**. Analogous reaction conditions have already been described by Du Bois et al., Isobe et al. and Vasella et al. [15–17].

GP-6: In this procedure, no *Schlenk*-conditions were applied. TMS protected alkyne **6** (1.0 equiv) was dissolved in a mixture of THF/H_2O (98:2) to give a 0.1 M solution and a 0.3 M solution of KF (1.1 equiv) and 18-crown-6 (1.1 equiv) in THF/H_2O (98:2) was added dropwise at 0 °C. After complete conversion (monitored by TLC, about 2.5 h), the reaction mixture was diluted with a saturated aqueous solution of NH_4Cl and extracted with Et_2O (3 × 50 mL). The combined organic layers were dried over Na_2SO_4 and the solvent was evaporated. The crude product was purified by column chromatography (in most cases EtOAc/PE, 1:2). The diastereomerically pure propargylamines **7** were isolated by recrystallization. This reaction procedure was applied for the synthesis of **7g**, **7j-l**, **7q**, **7s**, **7vx** and **7wx**. Typical reactions were carried out on a scale of 0.4–5 g of TMS protected alkyne **6**. Analogous reaction conditions have already been described by Vasella et al. [18].

GP-7: In this procedure, no *Schlenk*-conditions were applied. TMS protected alkyne **6** (8.27 mmol, 1.0 equiv) was dissolved in EtOH (80 mL) and a solution of $AgNO_3$ (22.3 mmol, 2.7 equiv) in EtOH/H_2O (60 mL, 58:42) was added dropwise at rt. After 20 min, a 4 M aqueous solution of KCN (99.2 mmol, 12.0 equiv) was added and the reaction mixture was neutralised with hydrochloric acid (1 M). After 2 h the mixture was concentrated up under reduced pressure and afterwards extracted with Et_2O (3 × 40 mL). The combined organic layers were dried over Na_2SO_4 and the solvent was evaporated. The crude product was purified by column chromatography (EtOAc/PE, 1:2). The diastereopure propargylamines **7** were isolated by recrystallization. These reaction conditions were only used for the synthesis of **7i** (typically 0.3–3 g). Analogous reaction conditions have already been described by Vasella et al. [17].

Swern Oxidation

GP-8: DMSO (50 mmol) was added dropwise to a solution of oxalylchloride (25 mmol) in DCM (60 mL) at −78 °C. After 2 min, a solution of the alcohol (23 mmol) in DCM (30 mL) was added over a period of 5 min. The reaction mixture was stirred for 30 min, before NEt_3 (115 mmol) was added. The resulting slurry was stirred for further 30 min at −78 °C and then warmed up to rt. The suspension was washed with water (50 mL) and a solution of $KHSO_4$ (5 %, 30 mL). The aqueous layers were extracted with DCM (2 × 30 mL) and the combined organic layers were washed with brine (20 mL) and dried over Na_2SO_4 before the solvent was evaporated under reduced pressure. The prepared aldehyde was directly converted or purified

by column chromatography or distillation. Typical reactions were carried out on a scale of 0.5–10 g of alcohol. The synthesis was carried out as described by Swern et al. [19].

Sonogashira cross coupling of propargylamine ***7*** *to peptidomimetic* ***11***

GP-9: DIPEA (6 equiv) was added to a solution of propargylamine **7** (1 equiv) and the methyl iodo-benzoate derivative (1.6 equiv) in THF (THF/DIPEA = 3:1). The reaction mixture was degassed by freeze pump thaw method, until no more gas atmosphere could be detected by the manometer. The catalysts, $Cl_2Pd(PPh_3)_2$ (2 mol %) and CuI (1 mol %) were added to the frozen reaction mixture and the solution slowly warmed to room temperature. After 30-120 min, a colourless precipitate formed in the clear solution, indicating the progress of the reaction. At least 2-8 h later, the suspension was diluted with a saturated aqueous NH_4Cl solution and $KHSO_4$ (aq, 5 %) was added, until the organic layer started to turn faintly red (pH 5-6). The emulsion was diluted with Et_2O, the organic layer separated and the organic layer extracted to more times with Et_2O. The combined organic layers were dried over Na_2SO_4 and the solvent was evaporated in vacuum. The crude product was purified by column chromatography. Typical reactions were carried out on a scale of 0.1–0.5 g of propargylamine **7**. Similar reactions have already been described by Hashmi [20], Ishida [21] and Wong et al. [22].

Synthesis

Ethyl (3-hydroxypropynyl)-benzoate derivatives 1

Ethyl 4-(3-hydroxyprop-1-yn-1-yl)benzoate (**1a**)

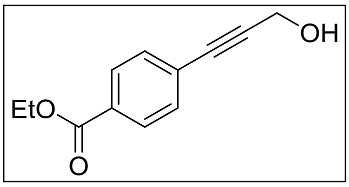

Synthesis: GP-9, reaction scale: 25.2 mmol of prop-2-yn-1-ol. Instead of DIPEA, 6 equivuivalents of piperidine were used. Isolation by column chromatography (PE/EtOAc, 4:1). Compound **1a** has been first described by Soler et al. [23].

Colourless crystals, yield: 4.32 g, 21.2 mmol, 84 %. ^{1}H NMR (300 MHz, Chloroform-*d*) δ = 7.99 (d, ^3J = 8.5 Hz, 2H, ar-2-**H**, ar-6-**H**), 7.49 (d, ^3J = 8.5 Hz, 2H, ar-3-**H**, ar-5-**H**), 4.52 (s, 2H, C**H$_2$**OH), 4.38 (q, ^3J = 7.1 Hz, 1H, OC**H$_2$**CH$_3$), 1.39 (t, ^3J = 7.1 Hz, 2H, OCH$_2$C**H$_3$**). $C_{12}H_{12}O_3$ (204.23 g mol^{-1}). TLC: R_f (EtOAc/PE, 1:4) = 0.28.

Ethyl 3-(3-hydroxyprop-1-yn-1-yl)benzoate (**1b**)

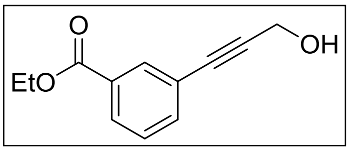

Synthesis: GP-9, reaction scale: 1.153 mmol of prop-2-yn-1-ol. Isolation by column chromatography (PE/EtOAc, 2:1). Compound **1b** has been described first by Chuang, Gallucci and Hart [24].

Colourless crystals, yield: 209.6 mg, 1.026 mmol, 89 %. ^{1}H NMR (500 MHz, Chloroform-*d*) δ = 8.03 (dd, ^4J = 1.8 Hz, ^4J = 1.5 Hz, 1H, ar-2-**H**), 7.92 (dd, ^3J = 7.8 Hz, ^4J = 1.5 Hz, 1H, ar-6-**H**), 7.51 (dd, ^3J = 7.7 Hz, ^4J = 1.5 Hz, 1H, ar-4-**H**), 7.30 (dd, ^3J = 7.8 Hz, ^3J = 7.8 Hz, 1H, ar-5-**H**), 4.48 (s, 2H, C**H$_2$**OH), 4.32 (q, ^3J = 7.1 Hz, 2H, OC**H$_2$**CH$_3$), 3.18 (s, 1H, CH$_2$O**H**), 1.33 (t, ^3J = 7.2 Hz, 3H, OCH$_2$C**H$_3$**). $C_{12}H_{12}O_3$ (204.23 g mol^{-1}). TLC: R_f (EtOAc/PE, 1:4) = 0.28. Smp = 48.8 °C.

Ethyl (3-oxopropynyl)-benzoate derivatives 2

Ethyl 4-(3-oxoprop-1-yn-1-yl)benzoate (**2a**)

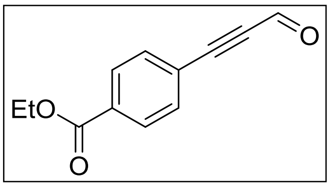

Synthesis: GP-8, reaction scale: 21.10 mmol of alcohol **1a**. Isolation by column chromatography (PE/EtOAc, 10:1).

Compound **2a** has been described by Moser, Lu, Patten, Wang, Kasar, Kaldor and Patterson [25].

Red solid, yield: 3.49 g, 17.3 mmol, 82 %. ^{1}H NMR (600 MHz, Chloroform-*d*) δ = 9.45 (s, 1H, C**H**O), 8.08 (d, ^{3}J = 8.4 Hz, 2H, ar-2-**H**, ar-6-**H**), 7.67 (d, ^{3}J = 8.3 Hz, 2H, ar-3-**H**, ar-5-**H**), 4.40 (q, ^{3}J = 7.1 Hz, 2H, OC**H₂**CH₃), 1.41 (t, ^{3}J = 7.1 Hz, 3H, OCH₂C**H₃**). $C_{12}H_{10}O_3$ (202.21 g mol^{-1}). TLC: R_f (EtOAc/PE, 1:1) = 0.78.

Ethyl 3-(3-oxoprop-1-yn-1-yl)benzoate (**2b**)

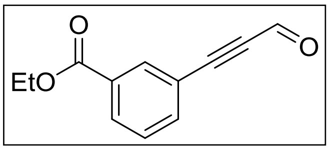

Synthesis: GP-8, reaction scale: 532 μmol of alcohol **1b**. Isolation by column chromatography (PE/EtOAc, 10:1).

Dark red oil, yield: 91.3 mg, 452 μmol, 85 %. ^{1}H NMR (300 MHz, Chloroform-*d*) δ = 9.44 (s, 1H, C**H**O), 8.28 (dd, ^{4}J = 1.7 Hz, ^{4}J = 1.4 Hz, 1H, ar-2-**H**), 8.16 (dd, ^{3}J = 7.8 Hz, ^{4}J = 1.4 Hz, 1H, ar-6-**H**), 7.77 (dd, ^{3}J = 7.7 Hz, ^{4}J = 1.4 Hz, 1H, ar-4-**H**), 7.50 (dd, ^{3}J = 7.8 Hz, ^{3}J = 7.7 Hz, 1H, ar-5-**H**), 4.40 (q, ^{3}J = 7.2 Hz, 2H, OC**H₂**CH₃), 1.41 (t, ^{3}J = 7.1 Hz, 3H, OCH₂C**H₃**). $C_{12}H_{10}O_3$ (202.21 g mol^{-1}). TLC: R_f (EtOAc/PE, 1:1) = 0.77.

Ethyl (*tert*-butylsulfinyl)imino)propynyl)benzoate derivatives 3

Ethyl (*R,E*)-4-(3-((*tert*-butylsulfinyl)imino)prop-1-yn-1-yl)benzoate (**3a**)

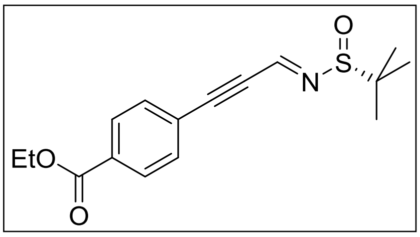

Synthesis: GP-2, reaction scale: 17.1 mmol of alcohol **2a** and 17.0 mmol of **(*R*)-1**. Isolation by column chromatography (PE/EtOAc, 4:1).

Yellow solid, yield: 1.72 g, 8.89 mmol, 52 %. ^{1}H NMR (300 MHz, Chloroform-*d*) δ = 8.06 (d, ^{3}J = 8.6 Hz, 2H, ar-2-**H**, ar-6-**H**), 8.04 (s, 1H, C**H**N), 7.64 (d, ^{3}J = 8.6 Hz, 2H, ar-3-**H**, ar-5-**H**), 4.39 (q, ^{3}J = 7.1 Hz, 2H, OC**H₂**CH₃), 1.40 (t, ^{3}J = 7.1 Hz, 3H, OCH₂C**H₃**), 1.27 (s, 9H, SC(C**H₃**)₃). ^{13}C NMR (126 MHz, Chloroform-*d*) δ = 165.8 (**C**O₂CH₂CH₃), 147.9 (**C**HN), 132.6 (ar-**C**-3, ar-**C**-5), 131.9 (ar-**C**-1), 129.8 (ar-**C**-2, ar-**C**-6), 125.2 (ar-**C**-4), 98.9 (C$^{\alpha}$H**C**≡Car), 87.4 (C$^{\alpha}$HC≡**C**ar), 61.6 (CO₂**C**H₂), 59.0 (S**C**(CH₃)₃), 22.7 (SC(**C**H₃)₃), 14.4 (CO₂CH₂**C**H₃). $C_{16}H_{19}NO_3S$ (305.39 g mol^{-1}). MS(ESI): *m/z* = 328.103 (328.0983 [M+Na]$^{+}$), $[\alpha]_{589}^{22.5}$ = -149.1 (*c* = 0.92; CHCl₃). IR(ATR): ῦ [cm^{-1}] = 2980-2868 (w, NH, C-H), 1717 (CO₂Et), 1220 (S=O). TLC: R_f (EtOAc/PE, 1:4) = 0.38.

Ethyl (*R*,*E*)-3-(3-((*tert*-butylsulfinyl)imino)prop-1-yn-1-yl)benzoate (**3b**)

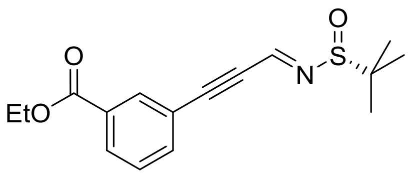

Synthesis: GP-2, reaction scale: 449 µmol of aldehyde **2b** and 450 µmol of **(*R*)-1**. Purification by column chromatography (PE/EtOAc, 2:1).

Red fluid liquid, yield: 110 mg, 359 µmol, 80 %. ^{1}H NMR (300 MHz, Chloroform-*d*) δ = 8.24 (s, 1H, ar-2-**H**), 8.08 (d, ^3J = 7.9 Hz, 1H, ar-6-**H**), 8.01 (s, 1H, C**H**N), 7.73 (d, ^3J = 7.7 Hz, 1H, ar-4-**H**), 7.45 (dd, ^3J = 7.8 Hz, ^3J = 7.8 Hz, 1H, ar-5-**H**), 4.37 (q, ^3J = 7.1 Hz, 2H, OC**H$_2$**CH$_3$), 1.38 (t, ^3J = 7.1 Hz, 3H, OCH$_2$C**H$_3$**), 1.25 (s, 9H, SC(C**H$_3$**)$_3$). $C_{16}H_{19}NO_3S$ (305.39 g mol^{-1}).

Ethyl benzoate substituted propargylamine derivatives 4

Ethyl 4-(3-((*tert*-butylsulfinyl)amido)but-1-yn-1-yl)benzoate (**4a**)

Methyllithium (1.6 M in Et_2O, 3.0 mL, 2.1 g, 2.6 mmol, 3.5 equiv) or MeMgBr (3 M in Et_2O, 0.5 mL, 0.4 g, 1.4 mmol, 1.4 equiv) was added dropwise to a deeply purple solution of imine **3a** (210 mg, 0.69 mmol, 1.0 equiv) and $AlMe_3$ (25 % in *n*-hexane, 0.82 mL, 0.56 g, 1.9 mmol) at -70 °C. The reaction mixture was stirred for 4.5 h at −30 °C to −40 °C. Then, EtOH (15 mL) was added and the crude mixture washed with an aqueous NH_4Cl solution. The aqueous layer was extracted with Et_2O (3 × 20 mL) and the combined organic layers were washed with brine and dried over Na_2SO_4. After evaporation of the solvent, the crude product was purified by preparative HPLC to yield racemic propargylamine in form of a yellow solid.

Yellow solid, yield (nucleophile = MeLi): 8.6 mg, 4 %, *dr* = 52:48. Yield (nucleophile = MeMgBr): 14.5 mg, 10 %, *dr* = 51:49.

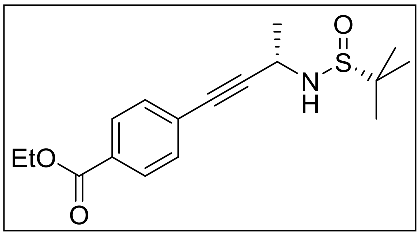

Ethyl 4-((*S*)-3-(((*R*)-*tert*-butylsulfinyl)amido)but-1-yn-1-yl)benzoate: ^{1}H NMR (600 MHz, Chloroform-*d*) δ = 7.96 (d, ^3J = 8.5 Hz, 2H, ar-2-**H**, ar-6-**H**), 7.68 (d, ^3J = 8.6 Hz, 1H, ar-3-**H**, ar-5-**H**), 4.48 (q, ^3J = 6.8 Hz, 1H, C^α**H**), 4.37 (q, ^3J = 7.1 Hz, 2H, OC**H$_2$**CH$_3$), 1.60 (dd, ^3J = 6.8 Hz, ^4J = 1.0 Hz, 3H, C^{α}HC**H$_3$**), 1.39 (t, ^3J = 7.2 Hz, 3H, OCH$_2$C**H$_3$**), 1.25 (s, 9H, SC(C**H$_3$**)$_3$). ^{13}C NMR (151 MHz, Chloroform-*d*) δ = 166.2 (**C**O$_2$Et), 132.8 (ar-**C**-3, ar-**C**-5), 131.7 (ar-**C**-1), 129.5

(ar-**C**-2, ar-**C**-6), 128.5 (ar-**C**-4), 92.5 (C^{α}**C**≡Car), 83.5 (C^{α}C≡**C**ar), 61.3 (O**C**H_2CH_3), 56.2 (S**C**Me_3), 44.2 (**C**$^{\alpha}$), 24.0 (C^{α}**C**H_3), 22.6 (SC(**C**H_3)$_3$), 14.4 (OCH_2**C**H_3). $C_{17}H_{23}NO_3S$ (321.44 g mol^{-1}). MS(ESI): *m/z* = 322.25 (322.44 $[M+H]^+$). IR(ATR): ῦ [cm^{-1}] = 3268- 3195 (NH), 2980- 2866 (CH), 1714 (CO_2Et), 1290 (S=O).

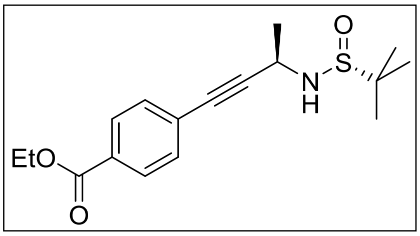

Ethyl 4-((*R*)-3-(((*R*)-*tert*-butylsulfinyl)amido)but-1-yn-1-yl)benzoate: ^{1}H NMR (600 MHz, Chloroform-*d*) δ = 7.96 (d, ^3J = 8.3 Hz, 2H, ar-2-**H**, ar-6-**H**), 7.48 (d, ^3J = 8.5 Hz, 2H, ar-3-**H**, ar-5-**H**), 4.41 (q, ^3J = 6.6 Hz, 1H, C^{α}**H**), 4.37 (q, ^3J = 7.1 Hz, 3H, OC**H$_2$**CH_3), 1.55 (d, ^3J = 6.5 Hz, 3H, C^{α}HC**H$_3$**), 1.39 (t, ^3J = 7.2 Hz, 3H, OCH_2C**H$_3$**), 1.24 (s, 9H, SC(C**H$_3$**)$_3$). ^{13}C NMR (151 MHz, Chloroform-*d*) δ = 147.6 (**C**O_2Et), 131.8 (ar-**C**-3, ar-**C**-5), 130.2 (ar-**C**-1), 129.5 (ar-**C**-2, ar-**C**-6), 127.4 (ar-**C**-4), 92.8 (C^{α}**C**≡Car), 87.8 (C^{α}C≡**C**ar), 58.8 (O**C**H_2CH_3), 56.2 (S**C**(CH_3)$_3$), 43.6 (**C**$^{\alpha}$), 26.9 (C^{α}H**C**H_3), 22.7 (SC(**C**H_3)$_3$), 14.4 (OCH_2**C**H_3). $C_{17}H_{23}NO_3S$ (321.44 g mol^{-1}). MS(ESI) *m/z* = 322.25 (322.44 $[M+H]^+$). IR(ATR): ῦ [cm^{-1}] = 3268- 3195 (NH), 2980- 2866 (CH), 1714 (CO_2Et), 1290 (SO).

Ethyl 3-((*R*)-3-(((*R*)-*tert*-butylsulfinyl)amido)-4-methylpent-1-yn-1-yl)benzoate (**4b**)

Isopropylmagnesium bromide (2 M in THF, 4 equiv) was added dropwise to a solution of imine **3b** and Ti(OiPr)$_4$ in THF at −78 °C. The reaction progress was monitored by analytical HPLC. After complete consumption of the starting material, the reaction was quenched by the addition of a saturated NH_4Cl solution. Et_2O was added, the layers separated and the organic layer was washed with a $KHSO_4$ (5%) solution. The aqueous layers were extracted with Et_2O and the combined organic layers were dried over Na_2SO_4. The solvent was evaporated in vacuo and the crude product purified by preparative HPLC.

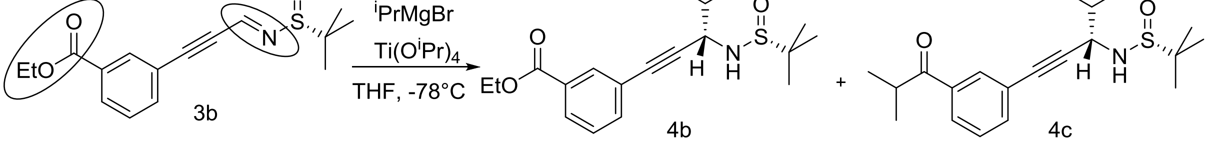

The relation of **4b**/**4c** was 6:4. In **4c**, ester and imine were both substituted. So, the chemoselectivity of imine/ester was 71:29.

Ethyl 3-((*R*)-3-(((*R*)-*tert*-butylsulfinyl)amido)-4-methylpent-1-yn-1-yl)benzoate (**4b**):

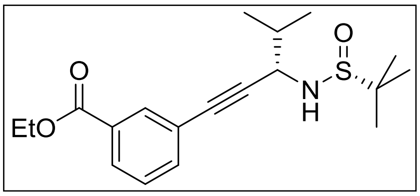

Yellow oil, yield: 8.0 mg, 23 µmol, 13 %, *dr* = 99:1. ^{1}H NMR (600 MHz, Chloroform-*d*) δ = 8.08 (dd, ^4J = 1.5 Hz, ^4J = 1.4 Hz, 1H, ar-2-**H**), 7.98 (ddd, ^3J = 7.9 Hz, ^4J = 1.5 Hz, ^4J = 1.4 Hz, 1H, ar-6-**H**), 7.60 (ddd, ^3J = 7.7 Hz, ^4J = 1.4 Hz, ^4J = 1.4 Hz, 1H, ar-4-**H**), 7.39 (td, ^3J = 7.8 Hz, ^3J = 7.8 Hz, ^4J = 0.6 Hz, 1H, ar-5-**H**), 4.39 (q, ^3J = 7.2 Hz, 2H, OC**H$_2$**CH$_3$), 4.25 (t, ^3J = 4.2 Hz, 1H, C$^\alpha$**H**), 3.39 (m, 1H, C$^\alpha$HN**H**), 2.05 (pd, ^3J = 6.7 Hz, ^3J = 4.9 Hz, 1H, C$^\alpha$C**H**(CH$_3$)$_2$), 1.40 (t, ^3J = 7.1 Hz, 3H, OCH$_2$C**H$_3$**), 1.26 (s, 9H, SC(C**H$_3$**)$_3$), 1.10 (d, ^3J = 6.7 Hz, 3H, C$^\alpha$HCHC**H$_3$**), 1.09 (d, ^3J = 6.7 Hz, 3H, C$^\alpha$HCHC**H$_3$**). ^{13}C NMR (126 MHz, Chloroform-*d*) δ = 166.1 (**C**O$_2$CH$_2$CH$_3$), 136.0 (ar-**C**-2), 132.9 (ar-**C**-6), 130.9 (ar-**C**-1), 129.4 (ar-**C**-4), 128.5 (ar-**C**-5), 123.3 (ar-**C**-3), 87.7 (C$^\alpha$**C**≡C-ar), 85.2 (C$^\alpha$C≡**C**-ar), 61.4 (O**C**H$_2$CH$_3$), 56.3 (S**C**(CH$_3$)$_3$), 54.7 (**C**$^\alpha$), 34.4 (C$^\alpha$**C**H(CH$_3$)$_2$), 22.7 (SC(**C**H$_3$)$_3$), 19.6 (**C**H$_3$CHCH$_3$), 17.4 (CH$_3$CH**C**H$_3$), 14.5 (OCH$_2$**C**H$_3$). $C_{19}H_{27}NO_3S$ (349.49 g mol^{-1}). MS(ESI): *m/z* = 372.3 (372.16 [M+Na]$^+$). $[\alpha]_{589}^{23}$ = 27.7 (*c* = 0.4; CHCl$_3$). IR(ATR): ῡ [cm^{-1}] = 3255 (NH), 2961- 2872 (C-H), 1717 (CO$_2$Et), 1292 (S=O).

Side-product:

(*R*)-*N*-((*S*)-1-(3-isobutyrylphenyl)-4-methylpent-1-yn-3-yl)-*tert*-butylsulfinamide (**4c**):

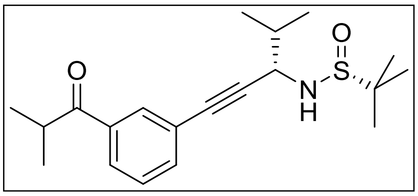

Yellow oil, yield: 5.5 mg, 16 µmol, 9 %. ^{1}H NMR (600 MHz, Chloroform-*d*) δ = 8.07 (dd, ^4J = 1.4 Hz, ^4J = 1.4 Hz, 1H, ar-2-**H**), 7.97 (dd, ^3J = 7.9 Hz, ^4J = 1.4 Hz, 1H, ar-6-**H**), 7.59 (dd, ^3J = 7.7 Hz, ^4J = 1.4 Hz, 1H, ar-4-**H**), 7.38 (dd, ^3J = 8.2 Hz, ^3J = 7.2 Hz, 1H, ar-5-**H**), 5.26 (sept, ^3J = 6.3 Hz, 1H, COC**H**(CH$_3$)$_2$), 4.38 (m, 1H, C$^\alpha$**H**), 4.25 (d, ^3J = 4.2 Hz, 1H, C$^\alpha$HN**H**), 2.05 (m, 1H, C$^\alpha$C**H**(CH$_3$)$_2$), 1.38 (d, ^3J = 6.2 Hz, 3H, COCH(C**H$_3$**)$_2$), 1.36 (d, ^3J = 6.2 Hz, 3H, COCH(C**H$_3$**)$_2$), 1.27 (s, 9H, SC(C**H$_3$**)$_3$), 1.10 (d, ^3J = 6.6 Hz, 3H, C$^\alpha$HCHC**H$_3$**), 1.09 (d, ^3J = 6.7 Hz, 3H, C$^\alpha$HCHC**H$_3$**). $C_{20}H_{29}NO_2S$ (347.52 g mol^{-1}). MS(ESI): *m/z* = 348.41 (348.20 [M+H]$^+$). IR(ATR): ῡ [cm^{-1}] = 3395- 3275 (NH), 2962- 2870 (C-H), 1673 (CO^{i}Pr), 1201 (SO).

Chiral aldimines 5

(*S*,*E*)-*N*-Ethylidene-2-*tert*-butylsulfinamide (**5a**)

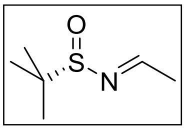

Synthesis: GP-1, reaction scale: 16.4 mmol of sulfinamide **(*S*)-1**. In contrast to other imine condensations, five equivalents of the acetaldehyde are necessary due to its low boiling point. The Lewis acid $Ti(OEt)_4$ was replaced by $MgSO_4$ (5 equiv) and the reaction was carried out at 30 °C overnight. Purification by column chromatography (PE/EtOAc, 4:1 or Et_2O). Compound **5a** has been first described by Ferreira, Audouin and Chemla [26].

Colourless, viscous oil, yield: 1.95 g, 13.3 mmol, 81 %. ^{1}H NMR (500 MHz, Chloroform-*d*) δ = 8.08 (q, ^3J = 5.1 Hz, 1H, C**H**N), 2.23 (d, ^3J = 5.1 Hz, 3H, CNC**H**$_3$), 1.19 (s, 9H, C(C**H$_3$**)$_3$). ^{13}C NMR (126 MHz, Chloroform-*d*) δ = 166.1 (**C**HN), 56.7 (**C**Me$_3$), 22.5 (C(**C**H$_3$)$_3$). $C_6H_{13}NOS$ (147.24 gmol^{-1}). MS(ESI): *m/z* = 170.0 (170.06 $[M+Na]^+$). TLC: R_f (PE/EtOAc, 4:1) = 0.3.

(*S*,*E*)-*N*-(2-Methylpropylidene)-*tert*-butylsulfinamide (**5b**)

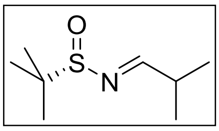

Synthesis: GP-1, reaction scale: 1.7 mmol of sulfinamide **(*S*)-1**. In contrast to other imine condensations, two equivalents of the isobutyraldehyde are necessary due to its low boiling point. Imine **5b** was achieved in pure form and not further purified. Compound **5b** was first described by Tang and Ellman [27].

Yellow viscous oil, yield: 0.27 g, 1.5 mmol, 90 %. ^{1}H NMR (600 MHz, Chloroform-*d*): δ = 7.98 ppm (d, ^3J = 4.4 Hz, 1H, C**H**N), 2.71 (m, 1H, C**H**(CH$_3$)$_2$), 1.18 (s, 9H, C(C**H$_3$**)$_3$), 1.17 (d, ^3J = 6.9 Hz, 3H, CH(C**H$_3$**)$_2$), 1.16 (d, ^3J = 6.8 Hz, 3H, CH(C**H$_3$**)$_2$). ^{13}C NMR (126 MHz, Chloroform-*d*) δ = 173.7 (**C**=N), 56.6 (S**C**(CH$_3$)$_3$), 35.0 (**C**H(CH$_3$)$_2$), 22.4 (SC(**C**H$_3$)$_3$), 19.0 ((CH$_3$)HC(**C**H$_3$)), 19.0 ((**C**H$_3$)HC(CH$_3$)). $C_8H_{17}NOS$ (175.29 gmol^{-1}). $[\alpha]^{20}_{589}$ = 230.2 (*c* = 2.28; $CHCl_3$). TLC: R_f (PE/EtOAc, 2:1) = 0.28.

(*S*,*E*)-*N*-(3-Methylbutylidene)-*tert*-butylsulfinamide (**5c**)

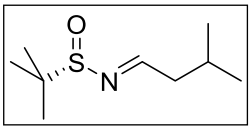

Synthesis: GP-1, reaction scale: 8.36 mmol of sulfinamide **(*S*)-1** and 10.0 mmol of isovaleraldehyde. No further purification of the crude

product was necessary. Compound **5c** was first described by Staas, Savage, Homnick, Tsou and Ball [28], as well as Ye, He and Zhang [29].

Colourless, viscous oil, yield: 1.46 g, 7.69 mmol, 92 %. ^{1}H NMR (300 MHz, Chloroform-*d*): δ = 8.02 (t, ^{3}J = 5.2 Hz, 1H, C**H**N), 2.43-2.36 (m, 2H, CNC**H$_2$**), 2.03 (m, 1H, $(H_3C)_2$C**H**), 1.17 (s, 9H, S(C(C**H$_3$**)$_3$), 0.98 (d, ^{3}J = 6.9 Hz, 3H, (CH$_3$)HC(C**H$_3$**)), 0.95 (d, ^{3}J = 7.3 Hz, 3H, (C**H$_3$**)CH(CH$_3$)). $C_9H_{19}NOS$ (189.32 g mol^{-1}). $[\alpha]_{589}^{20}$ = 276.3 (*c* = 2.09; CHCl$_3$). TLC: R_f (PE/EtOAc, 2:1) = 0.67.

(*S*,*E*)-*N*-(Cyclohexylmethylene)-*tert*-butylsulfinamide (**5d**)

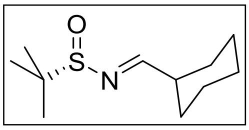

Synthesis: GP-2, reaction scale: 12.66 mmol of sulfinamide **(*S*)-1** and 13.9 mmol of cyclohexanecarbaldehyde. Isolation by column chromatography (PE/EtOAc, 4:1). Compound **5d** has been first described by Prakash, Mandal and Olah [30].

Colourless crystalline solid, yield: 2.616 g, 12.15 mmol 96 %. ^{1}H NMR (300 MHz, Chloroform-*d*) δ = 7.91 (d, ^{3}J = 4.5 Hz, 1H, C**H**N), 2.41 (m, 1H, cy-1-**H**), 1.83 (ddd, ^{2}J = 9.6 Hz, ^{3}J = 4.7 Hz, ^{3}J = 4.2 Hz, 2H, cy-2-**H**, cy-6-**H**), 1.78-1.69 (m, 2H, cy-3-**H**, cy-5-**H**), 1.62 (m, 1H, cy-4-**H**), 1.37-1.17 (m, 5H, cy-5-**H**, cy-3-**H**, cy-4-**H**, cy-6-**H**, cy-2-**H**), 1.13 (s, 9H, C(C**H$_3$**)$_3$). ^{13}C NMR (75 MHz, Chloroform-*d*) δ = 172.7 (**C**HN), 56.5 (**C**(CH$_3$)$_3$), 44.1 (cy-**C**-1), 29.4 (cy-**C**-2, cy-**C**-6), 25.9 (cy-**C**-4), 25.4 (cy-**C**-3), 25.4 (cy-**C**-5), 22.4 (C(**C**H$_3$)$_3$). $C_{11}H_{21}NOS$ (215.36 gmol^{-1}). MS(EI): *m/z* = 238.0 (238.12 [M+Na]$^+$). IR(ATR): $\tilde{\nu}$[cm^{-1}] = 2924 (C-H), 2851 (C-H), 1613 (CN), 1606 (SO), 1451, 1359, 1185, 1076 (SC), 967, 584.

(*S*,*E*)-*N*-(2,2-Dimethylpropylidene)-*tert*-butylsulfinamide (**5e**)

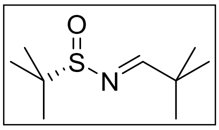

Synthesis: GP-2, reaction scale: 36.5 mmol of sulfinamide **(*S*)-1** and 36.5 mmol of pivalaldehyde. Reaction was monitored by TLC. Reaction time was increased to 7 d, due to the low reactivity of *tert*-butanal. Purification by column chromatography (EtOAc/PE, 1:4). Compound **5e** has been first described by Liu, Cogan, Owens, Tang and Ellman [3].

Colourless oil, yield: 1.245 g, 6.575 mmol, 18 %. ^{1}H NMR (500 MHz, Chloroform-*d*) δ = 7.87 (s, 1H, C**H**N), 1.13 (s, 9H, SC(C**H$_3$**)$_3$), 1.10 (s, 9H, C$^{\alpha}$C(C**H$_3$**)$_3$). ^{13}C NMR (126 MHz,

Chloroform-*d*) δ = 175.7 (**C**HN), 56.6 (S**C**(CH_3)$_3$), 38.0 (C^{α}**C**(CH_3)$_3$), 26.7 (C^{α}C(**C**H_3)$_3$), 22.4 (SC(**C**H_3)$_3$). $C_9H_{19}NOS$ (189.32 g mol^{-1}), MS(ESI): 190.1264 (190.12601 $[M+H]^+$). TLC: R_f (PE/EtOAc, 4:1) = 0.63.

(*S*)-*N*-((*E*)-((3*S*,5*S*,7*S*)-Adamantan-1-yl)methylene)-*tert*-butylsulfinamide (**5f**)

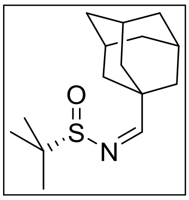

Synthesis: GP-2, reaction scale: 4.20 mmol of sulfinamide **(*S*)-1** and 4.06 mmol of adamantyl-1-carbaldehyde. Purification by column chromatography (PE/EtOAc, 4:1).

Colourless crystalline solid, yield: 456 mg, 1.70 mmol, 42 %. ^{1}H NMR (500 MHz, Chloroform-*d*) δ = 7.64 (s, 1H, C**H**N), 1.90 (d, ^2J = 25.1 Hz, 3H, CNC(CH_2C**H**CH_2)$_3$), 1.64 (d, ^2J = 15.1 Hz, 13H, CNC(C**H$_2$**CHC**H$_2$**)$_3$), 1.57 (d, ^2J = 11.8 Hz, 3H, CNC(C**H$_2$**CHCH_2)$_3$), 1.04 (s, 9H, SC(C**H$_3$**)$_3$). ^{13}C NMR (126 MHz, Chloroform-*d*) δ = 174.9 (**C**HN), 56.2 (S**C**(CH_3)$_3$), 39.9 (CN**C**(CH_2CHCH_2)$_3$), 39.0 (CNC(CH_2CH**C**H_2)$_3$), 36.4 (C^{α}C(**C**H_2CHCH_2)$_3$), 27.7 (C^{α}C(CH_2**C**HCH_2)$_3$), 22.1 (SC(**C**H_3)$_3$). $C_{15}H_{25}NOS$ (267.43 g mol^{-1}), MS(ESI): *m/z* = 268.1733 (268.1730 $[M+H]^+$). TLC: R_f (PE/EtOAc, 10:1) = 0.24.

(*R*,*E*)-*N*-(3-(Methylthio)propylidene)-*tert*-butylsulfinamide (**5g**)

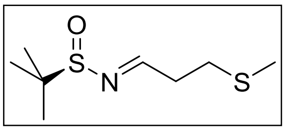

Synthesis: GP-2, reaction scale: 36.5 mmol of sulfinamide **(*R*)-1** and 19.85 mmol of 3-(methylthio)propanal. The crude product was purified by column chromatography (EtOAc/PE, 1:4). Compound **5g** has been first described by Gross, Heuser, Ammer, Heckmann and Bach [31] and Yao and Yuan [32].

Slightly yellow oil, yield: 3.663 g, 17.67 mmol, 89 %, ^{1}H NMR (300 MHz, Chloroform-*d*) δ = 8.03 (t, ^3J = 3.4 Hz, 1H, C**H**N), 2.80-2.73 (m, 4H, SC**H$_2$**C**H$_2$**), 2.07 (s, 3H, SC**H$_3$**), 1.15 (s, 9H, SC(C**H$_3$**)$_3$). ^{13}C NMR (75 MHz, Chloroform-*d*) δ = 167.5 (**C**HN), 56.9 (S**C**(CH_3)$_3$), 35.7 (CN**C**H_2), 29.6 (**C**H_2SCH_3), 22.4 (SC(**C**H_3)$_3$), 15.7 (S**C**H_3). $C_8H_{17}NOS_2$ (207.35 g mol^{-1}).

(*S*)-*N*-(2-Phenylethylidene)-*tert*-butylsulfinamide (**5h**)

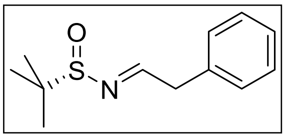

Synthesis: GP-2, reaction scale: 4.16 mmol of sulfinamide **(*S*)-1** and 5.40 mmol of phenylacetaldehyde. Purification of **5h** by column chromatography (DCM). Compound **5h** has been first described by Liu, Cogan, Owens, Tang and Ellman [3].

Yellow oil, yield: 593 mg, 2.66 mmol, 64 %. ^{1}H NMR (500 MHz, Chloroform-*d*): δ = 8.13 (t, ^{3}J = 5.2 Hz, 1H, C**H**N), 7.31 (m, 2H, ar-2-**H**, ar-6-**H**), 7.28-7.20 (m, 3H, ar-3-**H**, ar-4-**H**, ar-5-**H**), 3.85 (dd, ^{2}J = 15.1 Hz, ^{3}J = 5.2 Hz, 1H, CNC**H**$_2$), 3.80 (dd, ^{2}J = 15.1 Hz, ^{3}J = 5.2 Hz, 1H, CNC**H**$_2$), 1.18 (s, 9H, SC(C**H**$_3$)$_3$). $C_{12}H_{17}NOS$ (223.33 g mol^{-1}). TLC: R_f (DCM) = 0.28. $[\alpha]^{20}_{589}$ = 271.0 (*c* = 1.0; CHCl$_3$).

(*S*)-*N*-(Benzylidene)-*tert*-butylsulfinamide (**5i**)

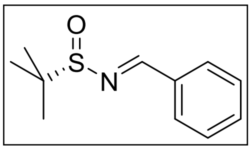

Synthesis: GP-1, reaction scale: 8.31 mmol of sulfinamide **(*S*)-1** and 12.5 mmol of benzaldehyde. The crude product was obtained in pure form. No further purification was necessary for the further conversions. Compound **5i** was first described by Ruano, Fernández, Catalina and Cruz [33].

Yellow oil, yield: 1.72 g, 8.23 mmol, 99 %. ^{1}H NMR (500 MHz, Chloroform-*d*): δ = 8.59 (s, 1H, N=C**H**), 7.86-7.85 (m, 2H, ar-2-**H**, ar-6-**H**), 7.54-7.46 (m, 3H, ar-3-**H**, ar-4-**H**, ar-5-**H**), 1.27 (s, 9H, SC(C**H$_3$**)$_3$). $C_{11}H_{15}NOS$ (209.31 g mol^{-1}). $[\alpha]^{20}_{589}$ = 124.4 (*c* = 1.0; CHCl$_3$).

(*S*,*Z*)-*N*-((Pentafluorophenyl)methylene)-*tert*-butylsulfinamide (**5j**)

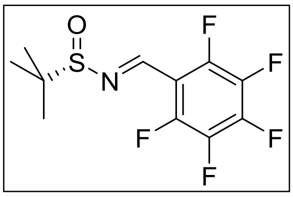

Synthesis: GP-2, reaction scale: 9.0 mmol of sulfinamide **(*S*)-1** and 9.0 mmol of pentafluorobenzaldehyde. Compound **5j** was purified by column chromatography (PE/EtOAc, 4:1).

Colourless highly viscous oil, yield: 2.37 g, 7.92 mmol, 88 %. ^{1}H NMR (300 MHz, Chloroform-*d*) δ = 8.72 (s, 1H, C**H**N), 1.28 (s, 9H, SC(C**H$_3$**)$_3$). ^{19}F NMR (282 MHz, Chloroform-*d*) δ = -139.9 (dt, $^{3}J_{FF}$ = 19.0 Hz, $^{4}J_{FF}$ = 6.3 Hz, 2F, ar-2-**F**, ar-6-**F**), -147.2 (tt, ^{3}J = 20.8 Hz, $^{4}J_{FF}$ = 4.9 Hz, ar-4-**F**), -160.8 (dd, ^{3}J = 20.7 Hz, $^{3}J_{FF}$ = 12.7 Hz, ar-3-**F**, ar-5-**F**). ^{13}C NMR (126 MHz, Chloroform-*d*) δ = 151.4 (d, $^{3}J_{CF}$ = 2.7 Hz, **C**HN), 146.4 (d, $^{1}J_{CF}$ =

260.9 Hz, ar-**C**-3, ar-**C**-5), 143.6 (d, $^{1}J_{CF}$ = 261.2 Hz, ar-**C**-4), 138.0 (d, $^{1}J_{CF}$ = 255.2 Hz, ar-**C**-2, ar-**C**-6), 109.7 (dd, $^{2}J_{CF}$ = 11.0 Hz, ^{3}J = 7.0 Hz, ar-**C**-1), 58.7 (**C**(CH_3)$_3$), 22.7 (SC(**C**H_3)$_3$). $C_{11}H_{10}F_5NOS$ (299.26 g mol^{-1}). ESI: *m/z* = 322.0306 (322.0301 [M+Na]$^+$). TLC: R$_f$(PE/EtOAc, 6:1) = 0.56.

(*S*,*E*)-*N*-(2,2,2-Trifluoroethylidene)-*tert*-butylsulfinamide (**5k**)

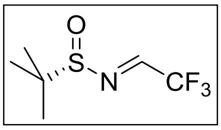

The preparation of 2,2,2-trifluoroacetaldehyde was carried out as described in the dissertation of Gerhard Greier [34]: Fluoral hydrate (75 %, 55.9 mmol) was placed under argon atmosphere in a closed vessel, which is linked by a Claisen condenser to a vessel with molecular sieves (3 Å), cooled to −78 °C. Concentrated sulfuric acid (10 mL) was added dropwise to the fluoral hydrate (6 mL) via dropping funnel and the solution was heated to 70 °C. The developing 2,2,2-trifluoroacetaldehyde was condensed in the cooled flask, where its mass was determined (2.08 g, 21.2 mmol).

To the condensed 2,2,2-trifluoroacetaldehyde, an equimolar amount of (*S*)-*tert*-butyl sulfinamide **(*S*)-1** (2.57 g, 21.2 mmol) was added in one portion. The mixture was diluted with dry toluene (20 mL) and stirred for 48 h at rt. The suspension was filtered under argon atmosphere through a frit and the solution of imine **5k** was stored under argon atmosphere for subsequent reactions.

Imine **5k** is formed as intermediate without isolation, as first described by Truong, Menard and Dion [35].

(*S*,*Z*)-*N*-(2,2,2-Trichloroethylidene)-*tert*-butylsulfinamide (**5l**)

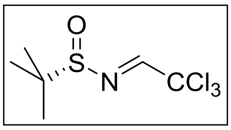

Chloral hydrate was converted to the aldehyde, chloral, following the description of Ullman’s Encyclopedia of Industrial Chemistry [36]: Chloral hydrate (5.00 g, 30.25 mmol, 1 equiv) was placed in a two neck flask with a column, filled with $CaCl_2$ and which is attached to a claisen condenser, leading to another flask, cooled to −78 °C. P_2O_5 (4.29 g, 15.1 mmol, 0.5 equiv) was added to the chloral hydrate in one portion through the second neck. The mixture was melted at 80 °C and then heated up to 120 °C. The developing gas was lead through the $CaCl_2$ and distilled (T =

approximately 100 °C) into the precooled flask, where the chloral was quantified (4.46 g, 30.25 mmol). Molecular sieves (4 Å, 15 g) and an equimolar amount of Ellman's chiral sulfinamide **(*S*)-1** (3.65 g, 30.0 mmol) was added and the reaction mixture was suspended in toluene (30 mL). After full conversion (7 d, monitored by TLC), the solid components were filtered through a pad of silica gel (PE/EtOAc, 10:1) and the solvent was evaporated under reduced pressure to yield imine **5l** in pure form.

Slightly yellow oil, yield: 6.56 g, 26.2 mmol, 87 %. ^{1}H NMR (500 MHz, Chloroform-*d*) δ = 7.98 (s, 1H, C**H**N), 1.25 (s, 9H, SC(C**H$_3$**)$_3$). ^{13}C NMR (126 MHz, Chloroform-*d*) δ = 159.6 (**C**HN), 93.0 (Cl$_3$**C**), 59.3 (S**C**(CH$_3$)$_3$), 22.6 (SC(**C**H$_3$)$_3$). $C_6H_{10}Cl_3NOS$ (250.56 g mol^{-1}). TLC: R$_f$ (PE/EtOAc, 4:1) = 0.72.

(*S,E*)-*N*-(2-(Trityloxy)ethylidene)-*tert*-butylsulfinamide (**5m**)

2-(Trityloxy)ethane-1-ol

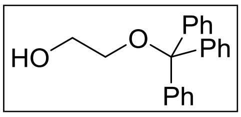

A solution of tritylchloride (5.01 g, 18.0 mmol, 1 equiv) in DCM (20 mL) was added dropwise to a solution of ethane-1,2-diol (3.02 mL, 54.0 mmol, 3 equiv), DMAP (0.22 mL, 1.8 mmol, 0.1 equiv) and Et_3N (5.05 mL, 18.0 mmol, 1 equiv) in DCM (120 mL). The reaction mixture was stirred for 48 h at rt. Then, H_2O (100 mL) was added. The phases were separated and the aqueous layer was extracted with DCM (2 × 100 mL). The combined organic layers were washed with brine, dried over Na_2SO_4 and the solvent was evaporated under reduced pressure. The crude product was purified by column chromatography (PE/EtOAc, 4:1).

Colourless crystalline solid, yield: 4.73 g, 15.5 mmol, 86 %. ^{1}H NMR (500 MHz, Chloroform-*d*) δ = 7.45 (d, ^3J = 7.2 Hz, 6H, ar-2-**H**, ar-6-**H**), 7.31 (t, ^3J = 7.5 Hz, 6H, ar-3-**H**, ar-5-**H**), 7.25 (t, ^3J = 6.7 Hz, 3H, ar-4-**H**), 3.78- 3.72 (m, 2H, C**H$_2$**CH$_2$OH), 3.27 (t, ^3J = 4.8 Hz, 2H, OC**H$_2$**), 1.93 (t, ^3J = 6.2 Hz, 1H, -O**H**). ^{13}C NMR (126 MHz, Chloroform-*d*) δ = 143.9 (ar-**C**-1), 128.7 (ar-**C**-2, ar-**C**-6), 127.9 (ar-**C**-3, ar-**C**-5), 127.1 (ar-**C**-4), 86.7 (Ph$_3$**C**), 64.8 (Ph$_3$CO**C**H$_2$), 62.4 (**C**H$_2$OH). $C_{21}H_{20}O_2$ (304.39 g mol^{-1}), MS(ESI): *m/z* = 327.2 (327.14 [M+Na]$^+$). IR(ATR): $\tilde{\nu}$ [cm^{-1}] = 3383 (OH), 3057 (ar-CH), 2946 (CH$_2$), 1448 (CH$_2$), 1100 (C-O-C). Smp: 99 °C. TLC: R$_f$ (PE/EtOAc, 1:1) = 0.44.

2-(Trityloxy)acetaldehyde

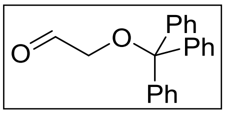

Synthesis: GP-8, reaction scale: 15.7 mmol of 2-(trityloxy)ethan-1-ol. The crude product was purified by filtration through a pad of silica gel (PE/EtOAc, 10:1).

Yellow oil, yield: 3.16 g, 10.5 mmol, 67 %. ^{1}H NMR (500 MHz, Chloroform-*d*) δ = 9.50 (t, ^{3}J = 1.2 Hz, 1H, C**H**O), 7.47 (m, 6H, ar-2-**H**, ar-6-**H**), 7.33 (m, 6H, ar-3-**H**, ar-5-**H**), 7.27 (m, 3H, ar-4-**H**), 3.86 (d, ^{3}J = 1.2 Hz, 2H, C**H₂**). $C_{21}H_{18}O_2$ (302.37 g mol^{-1}). IR(ATR): $\tilde{\nu}$ [cm^{-1}] = 3056 (ar, CH), 2974 (CH_2), 1729 (CHO), 1451 (ar, C=C). TLC: R_f (PE/EtOAc, 10:1) = 0.66.

(*S*,*E*)-*N*-(2-(Trityloxy)ethylidene)-*tert*-butylsulfinamide (**5m**)

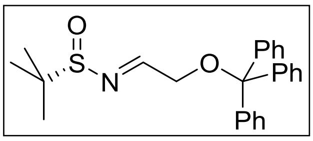

Synthesis: GP-2, reaction scale: 7.2 mmol of sulfinamide **(*S*)-1** and 7.13 mmol of 2-(trityloxy)acetaldehyde. Purification of the crude product by filtration through a short pad of silica gel (PE/EtOAc, 4:1).

Colourless, crystalline solid, yield: 1.36 g, 3.35 mmol, 47 %. ^{1}H NMR (500 MHz, Chloroform-*d*) δ = 8.19 (t, ^{3}J = 3.3 Hz, 1H, C**H**N), 7.58 (d, ^{3}J = 8.0 Hz, 6H, ar-2-**H**, ar-6-**H**), 7.38 (t, ^{3}J = 7.6 Hz, 6H, ar-3-**H**, ar-5-**H**), 7.32 (t, ^{3}J = 7.4 Hz, 3H, ar-4-**H**), 4.17 (t, ^{3}J = 3.7 Hz, 2H, C**H₂**), 1.28 (s, 9H, SC(C**H₃**)₃). ^{13}C NMR (126 MHz, Chloroform-*d*) δ = 166.9 (**C**HN), 143.4 (ar-**C**-1), 128.6 (ar-**C**-2, ar-**C**-6), 128.0 (ar-**C**-3, ar-**C**-5), 127.3 (ar-**C**-4), 87.4 (Ph_3**C**), 66.4 (**C**(CH_3)₃), 60.4 (**C**H_2), 22.4 (SC(**C**H_3)₃). $C_{25}H_{27}NO_2S$ (405.56 g mol^{-1}), MS(ESI): *m/z* = 428.2 (428.17 [M+Na]$^{+}$). TLC: R_f (PE/EtOAc, 4:1) = 0.65.

(*R*,*E*)-*N*-(2-(Benzyloxy)ethylidene)-*tert*-butylsulfinamide (**5n**)

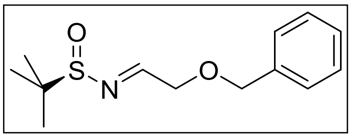

Synthesis: GP-2, reaction scale: 4.23 mmol of sulfinamide **(*R*)-1** and 4.3 mmol of 2-(benzyloxy)acetaldehyde. Purification by column chromatography (EtOAc/PE, 1:4). Decomposition was observed via ^{1}H NMR spectroscopy after 1 d. Compound **5n** has been first described by Tang, Volkman and Ellman [37].

Slightly yellow oil, yield: 869 mg, 3.43 mmol, 81 %. ^{1}H NMR (300 MHz, Chloroform-*d*) δ = 8.12 (t, ^{3}J = 3.2 Hz, 1H, C**H**N), 7.38-7.29 (m, 5H, ar-**H**), 4.63 (s, 2H, PhC**H₂**), 4.40 (d, ^{3}J = 3.2 Hz, 2H, CNC**H₂**), 1.21 (s, 9H, SC(C**H₃**)₃). $C_{13}H_{19}NO_2S$ (253.36 g mol^{-1}). TLC: R_f (EtOAc/PE, 1:4) = 0.34. $[\alpha]_{589}^{20}$ = 180.5 (*c* = 1.43; $CHCl_3$).

(*S*)-*N*-(2-(Allyloxy)ethylidene)-*tert*-butylsulfinamide (**5o**)

2-(Allyloxy)acetaldehyde

Silica gel adsorbed Sodium periodate was prepared according to the description of Roth and Stark [38]: Under stirring, Silica gel (5 g) was added to a solution of $NaIO_4$ (1.29 g, 6.03 mmol) in H_2O (2.5 mL) at 70 °C. The water was evaporated in vacuo and the crude product was dried in an desiccator over P_2O_5.

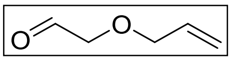

2-(Allyloxy)acetaldehyde was prepared as described by Karmann and Kazmaier [39]: To a suspension of silica gel adsorbed $NaIO_4$ (3 g, 2.88 mmol) in absolute DCM (7.5 mL), a solution of allyloxy-1,2-propanediol (220 mg, 1.64 mmol) in absolute DCM (6 mL) was added dropwise. The reaction mixture was stirred for 2 h at rt and then filtered through a pad of silica gel. The silica gel was washed with DCM and the solvent was evaporated under reduced pressure to yield alloxyacetaldehyde in pure form.

Yellow oil (crude product), yield: quant. ^{1}H NMR (500 MHz, Chloroform-*d*): δ [ppm] = 9.74 (s, 1H, C**H**O), 5.92 (m, 1H, C**H**=CH_2), 5.32 (dddd, ^{4}J = 1.6 Hz, ^{4}J = 1.4 Hz, ^{2}J = 1.4 Hz, ^{3}J = 17.2 Hz, 1H, CH=H_ZC**H**$_E$), 5.26 (dddd, ^{4}J = 1.4 Hz, ^{4}J = 1.3 Hz, ^{2}J = 1.2 Hz, ^{3}J = 10.4 Hz, 1H, CH=H_EC**H**$_Z$), 4.11-4.09 (m, 4H, C**H**$_2$-O-C**H**$_2$). $C_5H_8O_2$ (100.12 g mol^{-1}).

(*S*)-*N*-(2-(Allyloxy)ethylidene)-*tert*-butylsulfinamide (**5o**)

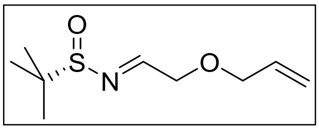

Synthesis: GP-1, reaction scale: 2.7 mmol of sulfinamide **(*S*)-1** and 2.58 mmol of 2-(allyloxy)acetaldehyde. The reaction mixture was dissolved in THF (4 mL) and the reaction time was stretched to 8 h. Purification by column chromatography (PE/EtOAc, 4:1).

Yellow oil, yield: 0.125 g, 0.620 mmol, 24 %. ^{1}H NMR (500 MHz, Chloroform-*d*): δ [ppm] = 8.10 (t, ^{3}J = 3.2 Hz, 1H, C**H**N), 5.91 (m, 1H, C**H**=CH_2), 5.30 (dddd, ^{4}J = 1.6 Hz, ^{4}J = 1.5 Hz, ^{3}J = 17.2 Hz, ^{2}J = 1.6 Hz, 1H, C=H_ZC$\mathbf{H}_E$), 5.23 (dddd, ^{4}J = 1.6 Hz, ^{4}J = 1.5 Hz, ^{3}J = 10.5 Hz, ^{2}J = 1.4 Hz, 1H, C=H_EC$\mathbf{H}_Z$), 4.39 (dd, ^{3}J = 3.1 Hz, ^{2}J = 16.4 Hz, 1H, CHNC$\mathbf{H}_2$O), 4.36 (dd, ^{3}J = 3.2 Hz, ^{2}J = 16.3 Hz, 1H, CHNC$\mathbf{H}_2$O), 4.10-4.08 (m, 2H, C$\mathbf{H_2}$CH=), 1.20 (s, 9H, SC(C$\mathbf{H_3}$)$_3$). ^{13}C NMR (125 MHz, Chloroform-*d*): δ [ppm] = 166.9 (**C**HN), 133.9 (**C**H=CH_2), 118.2 (CH=**C**H_2), 72.4 (**C**H_2CH=), 71.4 (**C**H_2O), 57.1 (S**C**Me_3), 22.5 (SC(**C**H_3)$_3$). $C_9H_{17}NO_2S$ (203.30 g mol^{-1}). MS(ESI): *m/z* = 204.0 (204.31 $[M+H]^+$). TLC: R_f (PE/EtOAc, 4:1) = 0.34. $[\alpha]_{589}^{20}$ = 232.3 (*c* = 1.2; $CHCl_3$).

(*R*,*E*)-*N*-(2-(Benzylthio)ethylidene)-*tert*-butylsulfinamide (**5p**)

The synthesis of all precursors of **5p** was carried out as described by Zhdanko, Gulevich and Nenajdenko [40].

Phenylmethanethiol was synthesised as described by Zhdanko, Gulevich and Nenajdenko

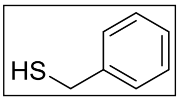

[40]. Brown fluid, yield: 9.106 g, 73 mmol, 56 %. ^{1}H NMR (300 MHz, Chloroform-*d*) δ = 7.45-7.43 (m, 4H, ar-2-**H**, ar-3-**H**, ar-5-**H**, ar-6-**H**), 7.34 (m, 1H, ar-4-**H**), 3.80 (s, 2H, C$\mathbf{H_2}$), 2.00 (s, 1H, S**H**). ^{13}C NMR (75 MHz, Chloroform-*d*) δ = 141.1 (ar-**C**-1), 128.6 (ar-**C**-3, ar-**C**-5), 128.0 (ar-**C**-4), 126.9 (ar-**C**-2, ar-**C**-6), 28.9 (**C**H_2). C_7H_8S (124.20 g mol^{-1}).

Benzyl(2,2-dimethoxyethyl)sulfane

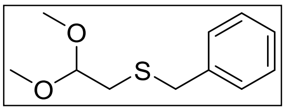

Sodium (1.76 g, 73.3 mmol, 1 equiv) was dissolved in a solution of phenylmethylthiol (9.12 g, 73.3 mmol, 1 equiv) in EtOH (38 mL). Afterwards, KI (372 mg, 2.24 mmol, 3 mol %) and chloroacetaldehyde dimethyl acetal (8.4 mL, 73.3 mmol, 1 equiv) were added and the reaction mixture was heated for 6 h to 80 °C. After cooling down to rt, the suspension was filtered, the residue washed with EtOH and the filtrate concentrated up under vacuum. The residue was diluted with water (45 mL) and washed with DCM (3 × 50 mL). The combined organic layers were washed with brine, dried over Na_2SO_4 and the solvent was evaporated. Purification of the crude product by column chromatography (PE/EtOAc, 10:1) yielded benzyl(2,2-dimethoxyethyl)sulfane in pure

form. The synthesis of the title compound has been first described by Zhdanko, Gulevich and Nenajdenko [40].

Deeply yellow oil, yield: 9.39 g, 44.2 mmol, 60 %. ^{1}H NMR (300 MHz, Chloroform-*d*) δ = 7.38-7.30 (m, 4H, ar-2-**H**, ar-3-**H**, ar-5-**H**, ar-6-**H**), 7.25 (m, 1H, ar-4-**H**), 4.43 (t, ^3J = 5.5 Hz, 1H, C**H**(OCH_3)$_2$), 3.80 (s, 2H, ar-C**H$_2$**), 3.36 (s, 6H, CH(OC**H$_3$**)$_2$), 2.61 (d, ^3J = 5.5 Hz, 2H, SC**H$_2$**CH(OCH_3)$_2$). $C_{11}H_{16}O_2S$ (212.31 g mol^{-1}).

2-(Benzylthio)acetaldehyde

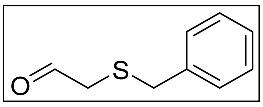

Benzyl(2,2-dimethoxyethyl)sulfane (5.00 g, 23.5 mmol, 1 equiv) was dissolved in H_2SO_4 (0.5 M, 21 mL) and the solution was heated for 5.5 h to 60 °C. After cooling down to rt, a solution of saturated $NaHCO_3$ was added until the pH was neutral. Afterwards, the solution was extracted with DCM (4 × 15 mL). The combined organic layers were dried over Na_2SO_4 and the solvent was evaporated under reduced pressure to yield 2-(benzylthio)acetaldehyde in pure form. The synthesis of the title compound has been first described by Zhdanko, Gulevich and Nenajdenko [40].

Deeply yellow oil, yield: 3.875 g, 23.31 mmol, 99 %. ^{1}H NMR (300 MHz, Chloroform-*d*) δ = 9.43 (t, ^3J = 3.4 Hz, 1H, C**H**O), 7.37-7.27 (m, 5H, C_6**H$_5$**), 3.64 (s, 2H, ar-C**H$_2$**), 3.09 (d, ^3J = 3.4 Hz, 2H, SC**H$_2$**CHO). $C_9H_{10}OS$ (166.24 g mol^{-1}).

(*R*,*E*)-*N*-(2-(Benzylthio)ethylidene)-*tert*-butylsulfinamide (**5p**)

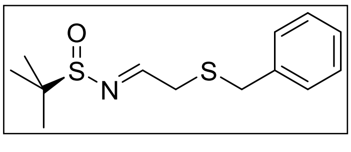

Synthesis: GP-2, reaction scale: 24.0 mmol of sulfinamide **(*R*)-1** and 23.34 mmol of 2-(benzylthio)acetaldehyde. Purification by column chromatography (PE/EtOAc, 4:1). Compound **5p** has been first described by Yao and Yuan [32].

Brown oil, yield: 5.212 g, 19.37 mmol, 83 %. ^{1}H NMR (300 MHz, Chloroform-*d*): *δ* (ppm) = 7.98 (t, ^3J = 5.6 Hz, 1H, C**H**N), 7.38-7.26 (m, 5H, C_6**H$_5$**), 3.70 (s, 2H, Ph-C**H$_2$**), 3.34 (d, ^3J = 2.8 Hz, 2H, SC**H$_2$**), 1.23 (s, 9H, C(C**H$_3$**)$_3$). $C_{13}H_{19}NOS_2$ (269.42 g mol^{-1}).

(S,E)-N-(4-Cyanobutyliden)-*tert*-butylsulfinamide (**5q**)

4-Iodobutan-1-ol

4-Iodobutan-1-ol was prepared as described by Sasano, Nagasawa, Yamazaki, Shibuya, Park, Iwabuchi [41].

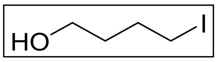

A solution of iodine (7.650 g, 30.14 mmol) in THF (50 mL) was added dropwise at 0 °C to a rigorously stirred suspension of $NaBH_4$ (0.568 g, 15.01 mmol) in THF (80 mL) over a period of 2 h. The reaction mixture was allowed to warm up to rt overnight. The deep purple solution was diluted with an aqueous Na_2SO_3 solution until it turned completely colourless. The organic layer was separated, the aqueous layer extracted with Et_2O (3 × 75 mL) and the combined organic layers dried over Na_2SO_4. Evaporation of the solvent yielded the title compound, which had to be used quickly in further conversions because it was observed to reform THF or polymerise. When the title compound perishes, it starts to turn brown or red.

Colourless oil, yield: 8.141 g, 40.70 mmol, 68 %. ^{1}H NMR (300 MHz, Chloroform-*d*) δ (ppm) = 3.66-3.60 (m, 2H, HOC**H₂**), 3.19 (t, ^3J = 6.9 Hz, 2H, IC$\mathbf{H_2}$), 1.90-1.83 (m, 2H, $HOCH_2C\mathbf{H_2}$), 1.69-1.59 (m, 2H, $ICH_2C\mathbf{H_2}$). C_4H_9IO (200.02 g mol^{-1}).

5-Hydroxypentanenitrile

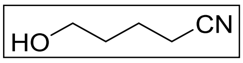

To a solution of 5-iodobutan-1-ol (16.5 g, 82.5 mmol, 1 equiv) in dry DMSO (75 mL), solid NaCN (6.06 g, 124 mmol, 1.5 equiv) was added in small portions at 0 °C. The reaction progress was surveilled by NMR spectroscopy. It is instant and the title compound as well as THF (6 : 5) are formed. After complete conversion, the reaction mixture was diluted with water (75 mL) and extracted with Et_2O (5 × 100 mL). The combined organic layers were washed with brine and dried over Na_2SO_4. Evaporation of the solvent yielded the title compound in pure form.

Slightly yellow oil, yield 3.596 g, 36.3 mmol, 52 %. ^{1}H NMR (600 MHz, DMSO-d_6) δ = 3.35 (t, ^3J = 6.2 Hz, 2H, $HOC\mathbf{H_2}$), 2.47 (t, ^3J = 7.0 Hz, 2H, $N{\equiv}CC\mathbf{H_2}$), 1.56-1.50 (m, 2H, $N{\equiv}CCH_2C\mathbf{H_2}$), 1.50-1.40 (m, 2H, $HOCH_2C\mathbf{H_2}$). ^{13}C NMR (151 MHz, DMSO-d_6) δ = 120.8 (**C**≡N), 59.6 ($HO\mathbf{C}H_2$), 31.3 ($HOCH_2\mathbf{C}H_2$), 21.7 ($HO(CH_2)_2\mathbf{C}H_2$), 16.2 ($N{\equiv}C\mathbf{C}H_2$). C_5H_9NO (99.13 g mol^{-1}).

5-Oxopentanenitrile

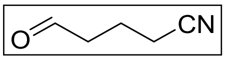

Synthesis: GP-8, reaction scale: 43.2 mmol of 5-hydroxypentanenitrile. Purification by filtration through a pad of silica gel (PE/EtOAc, 10:1).

Red oil, yield 2.434 g, 25.06 mmol, 58 %. ^{1}H NMR (300 MHz, Chloroform-*d*) δ = 9.81 (s, 1H, C**H**O), 2.70 (t, ^{3}J = 6.9 Hz, 2H, HCOC**H₂**), 2.46 (t, ^{3}J = 7.0 Hz, 2H, N≡CC**H₂**), 1.98 (p, ^{3}J = 6.9 Hz, 2H, N≡CCH₂C**H₂**). C_5H_7NO (97.13 g mol^{-1}).

(*S,E*)-*N*-(4-Cyanobutylidene)-*tert*-butylsulfinamide (**5q**)

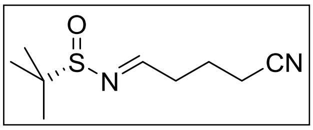

Synthesis: GP-2, reaction scale: 25.1 mmol of sulfinamide **(*S*)-1** and 26.0 mmol of 5-oxopentanenitrile. The crude product was purified by column chromatography.

Orange oil, yield 4.02 g, 20.1 mmol, 80 %. ^{1}H NMR (500 MHz, Chloroform-*d*): δ [ppm] = 8.10 (t, ^{3}J = 3.7 Hz, 1H, C**H**N), 2.71 (td, ^{3}J = 6.8 Hz, ^{3}J = 3.5 Hz, 2H, CHNC**H₂**), 2.51 (ddd, ^{2}J = 17.0 Hz, ^{3}J = 7.3 Hz, ^{3}J = 4.1 Hz, 1H, C**H**₂CH₂CN), 2.48 (ddd, ^{2}J = 17.0 Hz, ^{3}J = 7.1 Hz, ^{3}J = 3.8 Hz. 1H, C**H**₂CH₂CN), 2.09-2.01 (m, 2H, C**H₂**-CN), 1.2 (s, 9H, C(C**H₃**)₃). ^{13}C NMR (126 MHz, Chloroform-*d*): δ [ppm] = 167.3 (**C**HN), 119.1 (**C**N), 56.9 (**C**(CH₃)₃), 34.7 (HC$^{\alpha}$N**C**H₂), 22.8 (C(**C**H₃)₃), 21.4 (**C**H₂CN), 17.1 (**C**H₂CH₂CN). $C_9H_{16}N_2OS$ (200.30 g mol^{-1}). TLC: R_f (PE/EtOAc, 1:1) = 0.3. IR(ATR): ῦ [cm^{-1}] = 3501 (CH), 2962 (CH), 2927 (CH), 2901 (CH), 2243 (CN), 1625 (C=N), 1477 (SO), 1458, 1423, 1363, 1179, 1093 (SC), 102, 584.

Benzyl (*S,E*)-4-((*tert*-butylsulfinyl)imino)butanoate (**5r**)

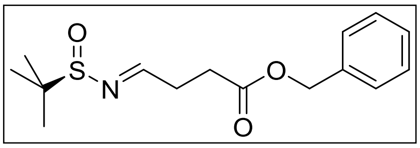

Synthesis: GP-2, reaction scale: 1.3 mmol of sulfinamide **(*S*)-1** and 1.14 mmol of benzyl-5-oxobutanoate. Purification by column chromatography (PE/EtOAc, 4:1).

Yellow oil, yield: 267 mg, 904 µmol, 79 %. ^{1}H NMR (500 MHz, Chloroform-*d*) *δ* = 8.12 (t, ^{3}J = 3.0 Hz, 1H, C**H**N), 7.37-7.31 (m, 5H, C₆**H₅**), 5.12-5.08 (m, 2H, arC**H₂**O), 2.87-2.83 (m, 2H,

$CHNC\mathbf{H_2}$), 2.77 (m, 1H, $C\mathbf{H}_2CO_2$), 2.68 (m, 1H, $C\mathbf{H}_2CO_2$), 1.14 (s, 9H, $SC(C\mathbf{H_3})_3$). ^{13}C NMR (125 MHz, Chloroform-*d*) δ = 172.2 ($\mathbf{C}O_2$), 167.2 (**C**HN), 135.8 (ar-**C**-1), 128.7 (ar-**C**-3, ar-**C**-5), 128.4 (ar-**C**-4), 128.2 (ar-**C**-2, ar-**C**-6), 66.6 (ar-$\mathbf{C}H_2O$), 56.8 ($S\mathbf{C}(CH_3)_3$), 31.0 ($CHN\mathbf{C}H_2$), 29.3 ($\mathbf{C}H_2CO_2$), 22.3 ($SC(\mathbf{C}H_3)_3$). $C_{15}H_{21}NO_3S$ (294.40 g mol^{-1}). MS(ESI): *m/z* = 318.11341 (318.11344 $[M+Na]^+$). $[\alpha]^{20}_{589}$ = 143.3 (*c* = 1.1; $CHCl_3$). TLC: R_f (PE/EtOAc, 4:1) = 0.28.

tert-Butyl (*S,E*)-4-((*tert*-butylsulfinyl)imino)butanoate (**5s**)

4-(*tert*-Butoxy)-4-oxobutanoic Acid

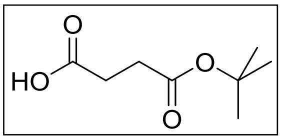

The monoester of succinate was prepared as described by Srinivasan, Uttamchandani and Yao [42]: *Tert*-butanol (10 mL) was added to a solution of succinic anhydrate (6.04 g, 60.40 mmol), *N*-hydroxysuccinimid (2.53g, 22.01 mmol) and DMAP (0.88 g, 7.23 mmol) in toluene (100 mL) and the solution was heated for 48 h under reflux conditions. After cooling down to rt, two layers formed in the reaction vessel (brown oil and clear, colourless solution). The crude solution was diluted with EtOAc (50 mL) and washed with citric acid (10 %, 2 × 50 mL) and brine. The organic layer was dried over Na_2SO_4, the solvent evaporated and the crude product was recrystallized from Et_2O/PE (1:3, 25 mL) to yield the title compound in quantitative yield.

Colourless crystals, yield: 10.52 g, 60.40 mmol, quant. ^1H NMR (500 MHz, Chloroform-*d*) δ = 2.63 (t, ^3J = 6.2 Hz, 2H, $CO_2HC\mathbf{H_2}$), 2.55 (t, ^3J = 6.8 Hz, 2H, $HO_2CCH_2C\mathbf{H_2}$), 1.45 (s, 9H, $CO_2C(C\mathbf{H_3})_3$). ^{13}C NMR (126 MHz, Chloroform-*d*) δ = 177.0 ($\mathbf{C}O_2H$), 171.4 ($\mathbf{C}O_2C(CH_3)_3$), 81.03 ($\mathbf{C}(CH_3)_3$), 30.10 ($\mathbf{C}H_2CO_2C(CH_3)_3$), 29.07 ($\mathbf{C}H_2CO_2H$), 28.03 ($C(\mathbf{C}H_3)_3$). $C_8H_{14}O_4$ (174.20 g mol^{-1}). Smp: 49 °C (44-45 °C, [42]).

tert-Butyl 4-hydroxybutanoate

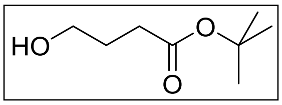

The title compound was prepared as described by Chen, Zhao, Chen, Chen, Kuznetsova, Wong and Ojima [43].

A solution of $BH_3 \times Me_2S$ (2M in THF, 11.4 mL, 22.76 mmol) was added dropwise to a heavily stirred solution of mono *tert*-butyl succinate (3.69 g, 21.17 mmol) in THF (35 mL) and the solution was stirred at ambient temperature for 17 h. The reaction mixture was diluted in EtOAc (150 mL) and the organic layer was washed with H_2O (30 mL) and brine (10 mL).

Drying over Na_2SO_4 and evaporation of the solvent yielded quantitatively *tert*-butyl-4-hydroxybutanoate.

Viscous oil, yield: 3.71 g, 18.5 mmol, 80 %. ^{1}H NMR (500 MHz, Chloroform-*d*) δ = 3.65 (t, ^3J = 6.2 Hz, 2H, C**H₂**OH), 2.37-2.29 (t, ^3J = 7.1 Hz, 2H, C**H₂**CO₂), 1.89-1.78 (m, 2H, CH₂-C**H₂**-CH₂), 1.43 (s, 9H, CO₂C(C**H₃**)₃). ^{13}C NMR (126 MHz, Chloroform-*d*) δ = 173.4 (**C**O₂), 80.6 (**C**(CH₃)₃), 62.3 (HO**C**H₂), 32.6 (**C**H₂CO₂), 28.2 (C(**C**H₃)₃), 28.0 (CH₂-**C**H₂-CH₂). $C_8H_{14}O_3$ (158.20 g mol^{-1}).

tert-Butyl 4-oxobutanoate

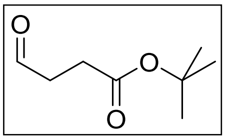

Synthesis: GP-8, reaction scale: 18.1 mmol of *tert*-butyl 4-hydroxybutanoate. The crude product was filtered through a pad of silica gel to yield the title compound in pure form.

Colourless oil, yield: 2.1 g, 13 mmol, 72 %. ^{1}H NMR (500 MHz, Chloroform-*d*) δ = 9.80 (s, 1H, C**H**O), 2.73 (t, ^3J = 6.6 Hz, 2H, CHOCH₂C**H₂**), 2.55 (t, ^3J = 6.6 Hz, 2H, CHOC**H₂**), 1.44 (s, 9H, CO₂C(C**H₃**)₃). $C_8H_{14}O_3$ (158.20 g mol^{-1}) MS(ESI): *m/z* = 181.0 (181.1 [M+Na]$^+$), 339.3 (339.2 [2M+Na]$^+$), 497.3 (497.3 [3M+Na]$^+$). IR(ATR): $\tilde{\nu}$ [cm^{-1}] = 2977 (CH₃), 2930 (CH₂), 1727 (CHO). TLC: R_f (PE/EtOAc, 4:1) = 0.52.

tert-Butyl (*S*,*E*)-4-((*tert*-butylsulfinyl)imino)butanoate (**5s**)

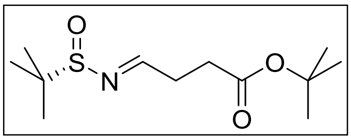

Synthesis: GP-2, reaction scale: 6.31 mmol of sulfinamide **(*S*)-1**. Isolation of title compound by column chromatography (PE/EtOAc, 4:1).

Colourless crystalline solid, yield: 1.06 g, 4.04 mmol, 64 %. ^{1}H NMR (500 MHz, Chloroform-*d*) δ = 8.12 (t, ^3J = 3.3 Hz, 1H, C**H**N), 2.79 (ddd, ^3J = 7.2 Hz, ^3J = 6.7 Hz, ^3J = 3.5 Hz, 2H, CO₂C**H₂**), 2.63 (ddd, ^2J = 16.8 Hz, ^3J = 7.2 Hz, 1H, C**H**₂CHN), 2.55 (dt, ^2J = 16.8 Hz, ^3J = 6.7 Hz, 1H, C**H**₂CHN), 1.44 (s, 9H, CO₂C(C**H₃**)₃), 1.18 (s, 9H, SC(C**H₃**)₃)). ^{13}C NMR (126 MHz, Chloroform-*d*) δ = 171.5 (**C**O₂), 167.6 (**C**HN), 80.7 (CO₂**C**(CH₃)₃), 56.7 (S**C**(CH₃)₃), 31.1 (**C**H₂CHN), 30.5 (CO₂**C**H₂), 28.1 (CO₂C(**C**H₃)₃), 22.3 (SC(**C**H₃)₃). $C_{12}H_{23}NO_3S$ (261.38 g mol^{-1}), MS(ESI): *m/z* = 262.1 (262.1 [M+H]$^+$), 284.1 (284.1 [M+Na]$^+$). TLC: R_f (PE/EtOAc, 4:1) = 0.26. IR(ATR): $\tilde{\nu}$ [cm^{-1}] = 2984 (CH₃), 2863 (CH₂), 1727 (C=O), 1622 (C=N), 1087 (S=O).

(*S*,*E*)-*N*-(4-((*tert*-Butyldimethylsilyl)oxy)butylidene)-*tert*-butylsulfinamide (**5t**)

4-((*tert*-Butyldimethylsilyl)oxy)butanal

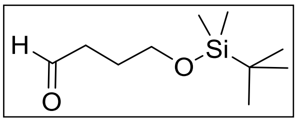

Synthesis: GP-8, reaction scale: 4.127 mmol of 4-((*tert*-butyldimethylsilyl)oxy)butan-1-ol. The title compound was obtained in pure form without further purification. The title compound has been first described by Asano and Matsubara [44].

Brightly yellow oil, yield: quant. ^{1}H NMR (500 MHz, Chloroform-*d*) δ = 9.78 (t, ^3J = 1.6 Hz, 1H, C**H**O), 3.65 (t, ^3J = 6.0 Hz, 2H, SiOC**H$_2$**), 2.50 (td, ^3J = 7.1 Hz, ^3J = 1.6 Hz, 2H, CHOC**H$_2$**), 1.86 (p, ^3J = 6.5 Hz, 2H, CH$_2$C**H$_2$**CH$_2$), 0.88 (s, 9H, C(C**H$_3$**)$_3$), 0.04 (s, 6H, Si(C**H$_3$**)$_2$). $C_{10}H_{22}O_2Si$ (202.37 g mol^{-1}).

(*S*,*E*)-*N*-(4-((*tert*-Butyldimethylsilyl)oxy)butylidene)-*tert*-butylsulfinamide (**5t**)

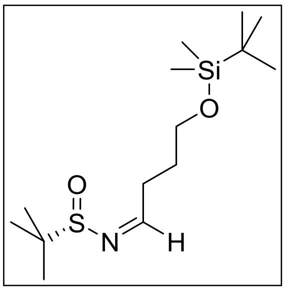

Synthesis: GP-1, reaction scale: 4.13 mmol of 4-((*tert*-butyldimethylsilyl)oxy)butanal and 4.30 mmol of sulfinamide **(*S*)-1**. Reaction for 1 h at 85 °C. The reaction progress was monitored by TLC. Purification by column chromatography. Compound **5t** has already been described by Bauer, DiBlasi and Tan [45].

Colourless oil, yield: 1.13 g, 3.70 mmol, 90 %. ^{1}H NMR (500 MHz, Chloroform-*d*) δ = 8.10 (t, ^3J = 4.5 Hz, 1H, C**H**N), 3.68 (t, ^3J = 6.2 Hz, 2H, OC**H$_2$**), 2.60 (td, ^3J = 7.5 Hz, ^3J = 4.5 Hz, 2H, CHNC**H$_2$**), 1.88-1.82 (m, 2H, OCH$_2$C**H$_2$**), 1.19 (s, 9H, SC(C**H$_3$**)$_3$), 0.89 (s, 9H, SiC(C**H$_3$**)$_3$), 0.05 (s, 6H, Si(C**H$_3$**)$_2$). ^{13}C NMR (126 MHz, Chloroform-*d*) δ = 169.6 (**C**N), 62.3 (Si**C**(CH$_3$)$_3$), 56.7 (S**C**(CH$_3$)$_3$), 32.9 (O**C**H$_2$), 28.7 (CHN**C**H$_2$), 26.1 (SiC(**C**H$_3$)$_3$), 22.5 (SC(**C**H$_3$)$_3$), 18.5 (OCH$_2$**C**H$_2$), -5.2 (Si(**C**H$_3$)$_2$). $C_{14}H_{31}NO_2SSi$ (305.55 g mol^{-1}). MS(ESI): *m/z* = 306.1924 (306.1918 [M+H]$^+$). TLC: R_f (PE/EtOAc, 2:1) = 0.54.

(*S,E*)-*N*-(4-Chlorobutylidene)-*tert*-butylsulfinamide (**5u**)

4-Chlorobutanal

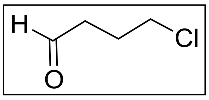

Synthesis: GP-8, reaction scale: 37.0 mmol of 4-chlorobutane-1-ol. No further purification.

Yellow oil, yield: 3.04 g, 28.5 mmol, 77 %. ^{1}H NMR (300 MHz, Chloroform-*d*) δ = 9.81 (t, ^{3}J = 1.1 Hz, 1H, C**H**O), 3.60 (t, ^{3}J = 6.3 Hz, 2H, C$\mathbf{H_2}$Cl), 2.67 (td, ^{3}J = 7.1 Hz, ^{3}J = 1.1 Hz, 2H, CHOC$\mathbf{H_2}$), 2.30-1.91 (m, 2H, CH_2C$\mathbf{H_2}$$CH_2$). C_4H_7ClO (106.55 g mol^{-1}). TLC: R$_f$ (EtOAc) = 0.7.

(*S,E*)-*N*-(4-Chlorobutylidene)-*tert*-butylsulfinamide (**5u**)

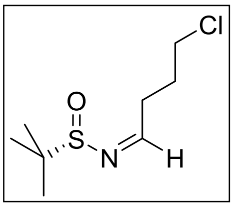

Synthesis: GP-1, reaction scale: 8.24 mmol of sulfinamide **(*S*)-1** and 8.5 mmol of 4-chlorobutanal. Reaction mixture was heated up to 90 °C. No further purification.

Orange oil, yield: 1.57 g, 7.00 mmmol, 85 %. ^{1}H NMR (300 MHz, Chloroform-*d*) δ = 8.10 (t, ^{3}J = 4.0 Hz, 1H, C**H**N), 3.62 (td, ^{3}J = 6.5 Hz, ^{4}J = 1.2 Hz, 2H, ClC$\mathbf{H_2}$), 2.78-2.66 (m, 2H, CHNC$\mathbf{H_2}$), 2.17-2.10 (m, 2H, CHNCH_2C$\mathbf{H_2}$), 1.19 (s, 9H, SC$(CH_3)_3$). ^{13}C NMR (75 MHz, Chloroform-*d*) δ = 168.1 (**C**HN), 56.8 (S**C**$(CH_3)_3$), 44.2 (Cl**C**H_2), 33.3 (CHN**C**H_2), 28.1 (CHNCH_2**C**H_2), 22.5 (SC(**C**$H_3)_3$). $C_8H_{16}ClNOS$ (209.73 g mol^{-1}). TLC: R$_f$ (EtOAc) = 0.64.

(*S,E*)-*N*-(5-Azidopentylidene)-*tert*-butylsulfinamide (**5v**)

5-Iodopentane-1-ol

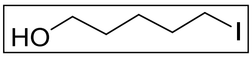

This synthesis was carried out analogue to the conversion of THF as first described by Sasano, Nagasawa, Yamazaki, Shibuya, Park and Iwabuchi [41] with some modifications.

$NaBH_4$ (1,00 g, 27 mmol, 1 equiv) was dissolved in dry Et_2O (25 mL). The suspension was cooled to 0 °C and a solution of tetrahydropyrane (10 mL, 102 mmol, 3.7 equiv) in Et_2O (25 mL) was added in one portion. A solution of iodine (13.4 g, 52.8 mmol, 2 equiv) in Et_2O

(50 mL) was added dropwise to the reaction mixture over a period of more than 3 hours at 0 °C. After each portion, the reaction mixture turned instantly a dark purple colour, which bleached within about 30 seconds. After the addition was complete, the solution was allowed to warm to room temperature overnight. Water was added to the resulting purple solution which immediately developed heat and gas. The organic solvent was evaporated and the aqueous layer was extracted with Et_2O (3 × 70 mL). The combined organic layers were washed with a solution of Na_2SO_3 and brine and dried over Na_2SO_4 to yield the title compound in form of a slightly orange, highly fluid oil.

Colourless oil, yield: 2.36 g, 11.02 mmol, 41 %. ^{1}H NMR (500 MHz, Chloroform-*d*) δ = 3.77 (t, ^3J = 6.4 Hz, 1H, O**H**), 3.66 (t, ^3J = 6.4 Hz, 2H, HOC$\mathbf{H_2}$), 3.20 (t, ^3J = 7.0 Hz, 2H, IC$\mathbf{H_2}$), 1.86 (p, ^3J = 7.0 Hz, 2H, $ICH_2CH_2C\mathbf{H_2}$), 1.63- 1.52 (m, 2H, $HOCH_2C\mathbf{H_2}$), 1.52- 1.42 (m, 1H, $ICH_2C\mathbf{H_2}$). ^{13}C NMR (126 MHz, Chloroform-*d*) δ = 62.8 (HO**C**H_2), 33.4 (I**C**H_2), 31.7 ($ICH_2CH_2\mathbf{C}H_2$), 30.5 ($HOCH_2\mathbf{C}H_2$), 26.9 ($ICH_2\mathbf{C}H_2$). C_5H_9IO (214.05 g mol^{-1}).

5-Azidopentane-1-ol

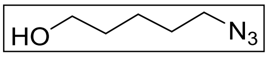

Similar reaction conditions were used as described by Sasano, Nagasawa, Yamazaki, Shibuya, Park and Iwabuchi [41]: An aqueous solution of NaN_3 (1.78 g, 26.5 mmol, 2.4 eq in 25 mL) was added to a solution of freshly prepared 5-iodopentanol (2.36 g, 11.0 mmo, 1 equiv) in THF (25 mL). After 12 hours, the dark yellow reaction mixture was diluted with an aqueous solution of Na_2SO_3, which made the colour vanish instantly. The organic solvent was evaporated and the residue was extracted with Et_2O (3 × 50 mL) and dried over Na_2SO_4. Evaporation of the solvent yielded the title compound in form of a colourless, thin oil (1.313 g, 10.17 mmol, 92 %).

An alternative preparation was executed, starting from 5-aminopentan-1-ol:

An azide transfer reagent was prepared in situ, as proposed by Barner-Kowolik et al. [46] and applied, as described by Días et al. [47]: Trifluoromethanesulfonic anhydride (3.25 mL, 19.4 mmol, 2.0 equiv) was added dropwise at 0 °C to a suspension of NaN_3 (4.53 g, 69.8 mmol, 7.2 equiv) in a mixture of DCM and H_2O (9:17, 25 mL). After 2 h, the aqueous layer was separated, extracted with DCM (15 mL) and the combined organic layers were washed with a saturated solution of Na_2CO_3 and dried over Na_2SO_4. After reduction of the solvent under reduced pressure, the concentrated solution was added to a mixture of 5-

aminopentan-1-ol (1.05 mL, 9.69 mmol, 1.0 equiv), $CuSO_4 \times 5\ H_2O$ (200 mg, 0.8 mmol, 0.1 equiv) and K_2CO_3 (1.6 g, 11.6 mmol, 1.2 equiv) in H_2O/MeOH (2:3, 70 mL). After 12 h, the organic solvent was evaporated and the residue was extracted with Et_2O (3 × 50 mL). The combined organic layers were dried over Na_2SO_4 and the solvent evaporated to yield the title compound (1.05 g, 8.14 mmol, 84 %).

^{1}H NMR (500 MHz, Chloroform-*d*) δ = 3.66 (t, ^3J = 6.4 Hz, 2H, HOC$\mathbf{H_2}$), 3.29 (t, ^3J = 6.9 Hz, 2H, N_3C$\mathbf{H_2}$), 1.68-1.57 (m, 4H, N_3CH_2C$\mathbf{H_2}$, $HOCH_2$C$\mathbf{H_2}$), 1.50-1.43 (m, 2H, $N_3CH_2CH_2$C$\mathbf{H_2}$). ^{13}C NMR (126 MHz, Chloroform-*d*) δ = 62.8 (**C**HO), 51.5 (**C**N_3), 32.3 ($N_3CH_2CH_2$**C**H_2), 28.8 ($HOCH_2$**C**H_2), 23.2 (N_3CH_2**C**H_2). $C_5H_9N_3O$ (127.15 g mol^{-1}).

5-Azidopentanal

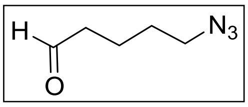

Synthesis: GP-8, reaction scale: 8.17 mmol of 5-azidopentanol. The product was slightly contaminated by DMSO and was applied for the following synthesis without further purification.

Colourless oil, yield: 0.61 g, 4.74 mmol, 58 %. ^{1}H NMR (500 MHz, Chloroform-*d*) δ = 9.79 (t, ^3J = 1.4 Hz, 1H, C**H**O), 3.31 (t, ^3J = 6.6 Hz, 2H, C$\mathbf{H_2}N_3$), 2.50 (td, ^3J = 7.1 Hz, ^4J = 1.5 Hz, 2H, CHOC$\mathbf{H_2}$), 1.77- 1.69 (m, 2H, $N_3CH_2CH_2$C$\mathbf{H_2}$), 1.67- 1.60 (m, 2H, N_3CH_2C$\mathbf{H_2}$). ^{13}C NMR (126 MHz, Chloroform-*d*) δ = 201.8 (**C**HO), 51.3 (N_3**C**H_2), 43.4 (CHO**C**H_2), 28.4 (N_3CH_2**C**H_2), 19.4 ($N_3CH_2CH_2$**C**H_2). $C_5H_9N_3O$ (127.15 g mol^{-1}).

(*S*,*E*)-*N*-(5-Azidopentylidene)-*tert*-butylsulfinamide (**5v**)

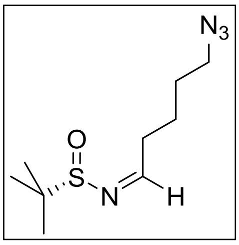

Synthesis: GP-2, reaction scale: 10.08 mmol of sulfinamide **(*S*)-1** and 10.10 mmol of 5-azidopentanal. Purification by column chromatography (PE/EtOAc, 4:1). Compound **5v** has been first described by Ye, He and Zhang [29].

Colourless oil, yield: 1.21 g, 5.24 mmol, 52 % (over two steps, referred to 5-azidopentane-1-ol). ^{1}H NMR (500 MHz, Chloroform-*d*) δ = 8.07 (t, ^3J = 4.4 Hz, 1H, C**H**N), 3.31 (t, ^3J = 6.5 Hz, 2H, N_3C$\mathbf{H_2}$), 2.56 (td, ^3J = 7.1 Hz, ^3J = 4.5 Hz, 2H, CHNC$\mathbf{H_2}$), 1.78-1.64 (m, 2H, N_3CH_2C$\mathbf{H_2}$), 1.19 (s, 9H, C(C$\mathbf{H_3}$)$_3$). ^{13}C NMR (126 MHz, Chloroform-*d*) δ = 168.8 (**C**HN), 56.8 (**C**(CH_3)$_3$), 51.3 (N_3**C**H_2), 35.6 (CHN-**C**H_2), 28.6 (N_3CH_2**C**H_2), 22.7 ($N_3CH_2CH_2$**C**H_2),

22.5 (C(**C**H_3)$_3$). $C_9H_{18}N_4OS$ (230.33 g mol^{-1}). MS(ESI): *m/z* = 253.1 (253.1 [M+Na]$^+$). TLC: R_f (EtOAc/PE, 1:2) = 0.57. IR(ATR): $\tilde{\nu}$ [cm^{-1}] = 2949 (CH), 2930 (CH), 2867 (CH), 2100 (N_3), 1622 (C=N), 1451 (SO), 1359, 1081 (SC).

(*S,E*)-*N*-(4-Azidobutylidene)-*tert*-butylsulfinamide (**5w**)

4-Azidobutane-1-ol

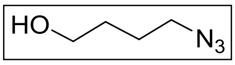

An aqueous solution of NaN_3 (170 mg, 2.57 mmol, 3.0 eq, 5 mL) was added in one portion to a solution of 4-iodobutane-1-ol (170 mg, 0.85 mmol, 1 equiv) in THF (5 mL). The reaction mixture was heated for 19 h to 80 °C. The solution was concentrated under reduced pressure, before it was extracted with DCM (4 × 10 mL). The combined organic layers were washed with brine and dried over Na_2SO_4. The title compound was isolated in pure form. The synthesis of 4-azidobutane-1-ol has been first described by Sasano, Nagasawa, Yamazaki, Shibuya, Park and Iwabuchi [41].

Colourless viscous oil, yield: 3.911 g, 33.97 mmol, 94 %. ^{1}H NMR (300 MHz, Chloroform-*d*): δ [ppm] = 3.60 (t, ^3J = 6.4 Hz, 2H, C**H**$_2$OH), 3.17 (t, ^3J = 6.9 Hz, 2H, C**H**$_2$$N_3$), 1.84-1.76 (m, 2H, C**H**$_2$CH_2), 1.66-1.56 (m, 2H, C**H**$_2$CH_2). $C_5H_{11}N_3O$ (129.16 g mol^{-1}). IR(ATR): $\tilde{\nu}$ [cm^{-1}] = 3600-3200 (O-H), 2926 (CH_2), 2885 (CH_2), 2356-2334 (N_3). $C_4H_9N_3O$ (115.14 g mol^{-1}). TLC: R_f (PE/EtOAc, 2:1) = 0.5.

4-Azidobutanal

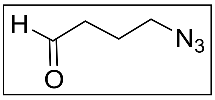

Synthesis: GP-8, reaction scale: 34 mmol of 4-azidobutanol. Purification by filtration through a pad of silica gel (PE/EtOAc, 10:1).

Slightly yellow, fluid oil, yield: quant. ^{1}H NMR (600 MHz, Chloroform-*d*) δ = 9.74 (s, 1H, C**H**O), 3.55 (dd, ^3J = 7.3 Hz, ^3J = 5.4 Hz, 1H, N_3C**H**$_2$), 3.31 (t, ^3J = 6.6 Hz, 1H, N_3C**H**$_2$), 2.62 (td, ^3J = 7.0 Hz, ^4J = 1.1 Hz, 1H, $N_3CH_2CH_2$C**H**$_2$), 2.53 (td, ^3J = 7.0 Hz, ^4J = 1.2 Hz, 1H, $N_3CH_2CH_2$C**H**$_2$), 2.05 (p, ^3J = 6.7 Hz, 1H, N_3CH_2C**H**$_2$), 1.86 (pd, ^3J = 6.8 Hz, ^4J = 1.8 Hz, 1H, N_3CH_2C**H**$_2$). ^{13}C NMR (126 MHz, Chloroform-*d*) δ = 201.0 (**C**HO), 50.7 (N_3**C**H_2), 41.0 (**C**H_2CHO), 21.6 (N_3CH_2**C**H_2). $C_4H_7N_3O$ (113.12 g mol^{-1}).

(*S*,*E*)-*N*-(4-Azidobutylidene)-*tert*-butylsulfinamide (**5w**)

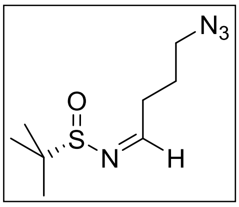

Synthesis: GP-2, reaction scale: 56.0 mmol of sulfinamide **(*S*)-1** and 60.0 mmol of 4-azidobutanal. Purification by column chromatography (PE/EtOAc, 4:1). Compound **5w** has been first described by Shu, Liu, Wang, Li and Ye [48].

Faintly green, thin oil, yield: 10.1 g, 46.9 mmol, 84 % (over two steps, referred to 4-azidobutan-1-ol). ^{1}H NMR (500 MHz, Chloroform-*d*) δ = 8.09 (t, ^{3}J = 4.2 Hz, 1H, C**H**N), 3.38 (t, ^{3}J = 6.8 Hz, 2H, $N_3C\mathbf{H_2}$), 2.62 (td, ^{3}J = 7.2 Hz, ^{3}J = 4.9 Hz, 2H, CHNC**H$_2$**), 1.94 (m, 2H, $N_3CH_2C\mathbf{H_2}$), 1.19 (s, 9H, C(C**H$_3$**)$_3$). ^{13}C NMR (126 MHz, Chloroform-*d*) δ = 168.1 (**C**HN), 56.8 (**C**(CH$_3$)$_3$), 50.8 ($N_3\mathbf{C}H_2$), 33.2 (CHN**C**H$_2$), 24.7 ($N_3CH_2\mathbf{C}H_2$), 22.5 (C(**C**H$_3$)$_3$). $C_8H_{16}N_4OS$ (216.30 g mol^{-1}). MS(ESI): *m/z* = 239.0 (239.1 [M+Na]$^+$). IR(ATR): ṽ [cm^{-1}] = 2955 (CH), 2924 (CH), 2867 (CH), 2091 (N_3), 1625 (C=N), 1458 (SO), 1363, 1255, 1080 (SC). TLC: R_f (PE/EtOAc, 1:1) = 0.62, R_f (EtOAc/PE, 1:2) = 0.57.

TMS protected propargylamines 6

In most cases, compound **6** was not isolated. The crude product of the described synthesis was directly converted to propargylamine **7**, without further purification.

(*S*)-*N*-((*S*)-4-(Trimethylsilyl)but-3-yn-2-yl)-*tert*-butylsulfinamide (**6a**)

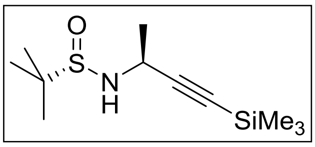

Synthesis: GP-3, reaction scale: 12.7 mmol of imine **5a**. Alkyne **6a** was not purified. The crude product was directly converted to **7a**. ^{1}H NMR (300 MHz, Chloroform-*d*) *δ* [ppm] = 4.20 (qd, ^{3}J = 6.7 Hz, ^{3}J = 4.5 Hz, 1H, C$^{\alpha}$**H**), 3.36 (d, ^{3}J = 4.6 Hz, 1H, N**H**), 1.43 (d, ^{3}J = 6.7 Hz, 3H, C**H$_3$**), 1.22 (s, 9H, C(C**H$_3$**)$_3$), 0.16 (s, 9H, Si(C**H$_3$**)$_3$). $C_{11}H_{23}NOSSi$ (245.46 g mol^{-1}). TLC: R_f (PE/EtOAc, 1:1) = 0.4.

(*S*)-*N*-((*S*)-4-Methyl-1-(trimethylsilyl)pent-1-yn-3-yl)-*tert*-butylsulfinamide (**6b**)

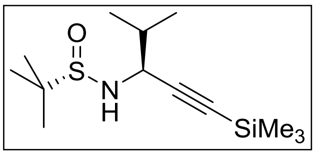

Synthesis: GP-3, reaction scale: 22.5 mmol of imine **5b**. The application of two equivalents of lithiated acetylene lead to a

decreased yield. The crude product was directly converted to form **7b** without further purification.

Brown oil, ^{1}H NMR (600 MHz, Chloroform-*d*): δ = 3.92 (dd, ^3J = 5.3 Hz, ^3J = 6.4 Hz, 1H, NHC$^{\alpha}$**H**), 3.30 (d, ^3J = 5.4 Hz, 1H, N**H**), 1.92 (m, 1H, C**H**($CH_3)_2$), 1.22 (s, 9H, C(C**H$_3$**)$_3$), 1.01 (d, ^3J = 6.3 Hz, 3H, CHC**H$_3$**), 1.00 (d, ^3J = 7.5 Hz, 3H, CHC**H$_3$**), 0.16 (s, 9H, Si(C**H$_3$**)$_3$). $C_{13}H_{27}NOSSi$ (273.51 g mol^{-1}).

(*S*)-*N*-((*S*)-5-Methyl-1-(trimethylsilyl)hex-1-yn-3-yl)-*tert*-butylsulfinamide (**6c**)

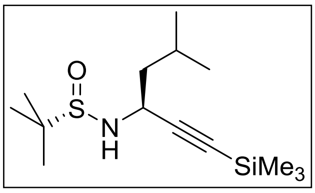

Synthesis: GP-3, reaction scale: 7.61 mmol of imine **5c**. The crude product of alkyne **6c** was directly converted to propargylamine **7c** without further purification.

Dark green oil, yield: 1.07 g, 3.73 mmol, 49 %. ^{1}H NMR (300 MHz, Chloroform-*d*): δ = 4.05 (td, ^3J = 7.6 Hz, ^3J = 6.2 Hz, 1H, C$^{\alpha}$**H**), 3.26 (d, ^3J = 6.0 Hz, 1H, N**H**), 1.83 (m, 1H, C**H**($CH_3)_2$), 1.56 (t, ^3J = 7.3 Hz, 2H, C**H$_2$**), 1.20 (s, 9H, C(C**H$_3$**)$_3$), 0.92 (d, ^3J = 6.6 Hz, 3H, CHC**H$_3$**), 0.91 (d, ^3J = 6.6 Hz, 3H, CHC**H$_3$**), 0.14 (s, 9H, Si(C**H$_3$**)$_3$). $C_{14}H_{29}NOSSi$ (287.54 g mol^{-1}). TLC: R_f (PE/EtOAc, 1:1) = 0.65.

(*S*)-*N*-((*S*)-1-Cyclohexyl-3-(trimethylsilyl)prop-2-yn-1-yl)-*tert*-butylsulfinamide (**6d**)

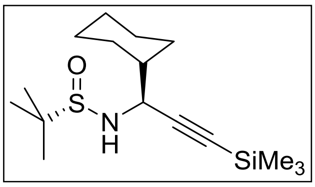

Synthesis: GP-3, reaction scale: 12.12 mmol of imine **5d**. The crude product was applied for the conversion to propargylamine **7d** without further purification.

Dark, brown, viscous oil, yield: 3.496 g, 11.15 mmol, 92 %. ^{1}H NMR (300 MHz, Chloroform-*d*) δ = 3.88 (dd, ^3J = 5.8 Hz, ^3J = 6.1 Hz, 1H, C$^{\alpha}$**H**), 3.24 (d, ^3J = 6.1 Hz, 1H, N**H**), 2.37 (m, 1H, cy-1-**H**), 1.75 (dd, ^2J = 11.9 Hz, ^3J = 6.1 Hz, 6H, cy-2-**H**, cy-6-**H**), 1.66 (d, ^2J = 11.0 Hz, 1H, cy-4-**H**), 1.55 (dd, ^3J = 5.6 Hz, ^3J = 4.4 Hz, 2H, cy-5-**H**, cy-3-**H**), 1.29- 1.23 (m, 2H, cy-6-**H**, cy-2-**H**), 1.22 (s, 9H, C(C**H$_3$**)$_3$), 1.09 (m, 1H, cy-5-**H**), 0.88 (m, 1H, cy-3-**H**), 0.16 (s, 9H, Si(C**H$_3$**)$_3$). $C_{16}H_{31}NOSSi$ (313.57 g mol^{-1}).

(*S*)-*N*-((*S*)-4,4-Dimethyl-1-(trimethylsilyl)pent-1-yn-3-yl)-*tert*-butylsulfinamide (**6e**)

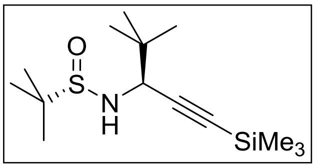

Synthesis: GP-4, reaction scale: 6.62 mmol of imine **5e**. In analogy to the preparation of compounds **7vy** and **7wy**, PPh_3 (2 equiv) was added in one portion to the reaction mixture at −78 °C. After 2 h, water was added and the reaction mixture warmed up to rt. The organic layer was washed with a saturated solution of NH_4Cl and $KHSO_4$ (5 %). The aqueous layers were extracted with Et_2O (2 × 40 mL) and the combined organic layers were washed with brine and dried over Na_2SO_4. The crude product was investigated by LCMS and **6e** was purified by column chromatography (EtOAc/PE, 1:10). Propargylamine **7e** could not be observed by LCMS.

Colourless crystalline solid, yield: 856 mg, 2.98 mmol, 45 %. ^{1}H NMR (500 MHz, Chloroform-*d*) δ = 3.66 (d, ^3J = 8.1 Hz, 1H, C^α**H**), 3.19 (d, ^3J = 8.2 Hz, 1H, N**H**), 1.24 (s, 9H, SC(C**H$_3$**)$_3$), 0.98 (s, 9H, C^{α}C(C**H$_3$**)$_3$), 0.16 (s, 9H, Si(C**H$_3$**)$_3$). ^{13}C NMR (126 MHz, Chloroform-*d*) δ = 104.8 (C^{α}C≡**C**TMS), 90.4 (C^α**C**≡CTMS), 58.7 (C^α**C**(CH$_3$)$_3$), 56.9 (S**C**(CH$_3$)$_3$), 36.4 (**C**^{α}H), 26.2 (C^{α}C(**C**H$_3$)$_3$), 23.0 (SC(**C**H$_3$)$_3$), 0.1 (Si(**C**H$_3$)$_3$). $C_{14}H_{29}NOSSi$ (287.54 g mol^{-1}). TLC: R_f (EtOAc/PE, 1:4) = 0.52.

(*S*)-*N*-((1*S*)-1-((1r,3*R*,5*S*)-Adamantane-1-yl)-3-(trimethylsilyl)prop-2-yn-1-yl)-*tert*-butylsulfinamide (**6f**)

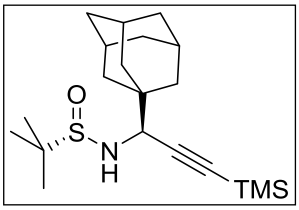

Synthesis: GP-4. Reaction scale: 1.7 mmol of imine **5f**. Compound **6f** was directly converted to the free propargylamine **7f** without further purification or investigation. $C_{20}H_{35}NOSSi$ (365.65 g mol^{-1}).

(*R*)-*N*-((*R*)-5-(Methylthio)-1-(trimethylsilyl)pent-1-yn-3-yl)-*tert*-butylsulfinamide (**6g**)

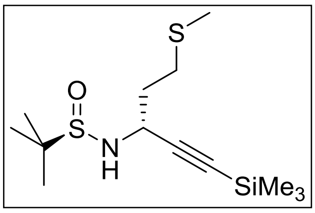

Synthesis: GP-4, reaction scale: 12.6 mmol of imine **5g**. Both stereocenters are (*R*)-configured. The crude product was not further purified after the aqueous workup. Compound **6g** was directly converted to propargylamine **7g** without further purification of the crude product.

Brown oil, yield: 2.07 g, *dr* = 96:4. ^{1}H NMR (500 MHz, Chloroform-*d*) δ = 4.23 (ddd, ^{3}J = 6.1 Hz, ^{3}J = 6.3 Hz, ^{3}J = 6.3 Hz, 1H, C$^{\alpha}$**H**), 3.40 (d, ^{3}J = 5.7 Hz, 1H, N**H**), 2.67-2.59 (m, 2H, S-C**H**$_2$), 2.10 (s, 3H, S-C**H**$_3$), 2.05-1.93 (m, 2H, C$^{\alpha}$C**H**$_2$), 1.21 (s, 9H, SC(C**H**$_3$)$_3$), 0.15 (s, 9H, Si(C**H**$_3$)$_3$). $C_{13}H_{27}NOS_2Si$ (305.57 g mol^{-1}).

(*S*)-*N*-((*S*)-1-Phenyl-4-(trimethylsilyl)but-3-yn-2-yl)-*tert*-butylsulfinamide (**6h**)

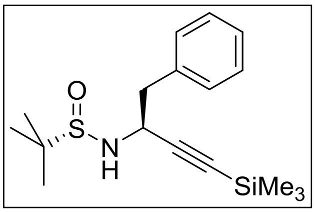

Synthesis: GP-4, reaction scale: 3.12 mmol of imine **5h**. Compound **6h** was not purified. The crude product was purified by filtration through a pad of silica (PE/EtOAc(Net$_3$, 85:14:1).

Yellow oil, yield: 341 mg, 1.06 mmol, 34 %. *dr* = 97:3. ^{1}H NMR (500 MHz, Chloroform-*d*): *δ* = 7.38-7.27 (m, 5H, ar-**H**), 4.26 (m, 1H, C$^{\alpha}$**H**), 3.37 (d, ^{3}J = 6.1 Hz, 1H, N**H**), 3.08-2.95 (m, 2H, C$^{\alpha}$C**H**$_2$), 1.15 (s, 9H, SC(C**H**$_3$)$_3$), 0.15 (s, 9H, Si(C**H**$_3$)$_3$). ^{13}C NMR (125 MHz, Chloroform-*d*): *δ* [ppm] = 136.6 (ar-**C**-1), 130.1 (ar-**C**-3, ar-**C**-5), 128.4 (ar-**C**-2, ar-**C**-6), 127.0 (ar-**C**-4), 104.8 (TMS**C**≡C), 90.8 (TMS-C≡**C**), 56.4 (S**C**(CH$_3$)$_3$), 49.6 (**C**$^{\alpha}$), 43.4 (C$^{\alpha}$**C**H$_2$), 22.6 (SC(**C**H$_3$)$_3$), -0.10 (Si(**C**H$_3$)$_3$). $C_{17}H_{27}NOSSi$ (321.55 g mol^{-1}). TLC: R_f (PE/EtOAc/NEt$_3$, 85:14:1) = 0.17.

(*S*)-*N*-((*R*)-1-Phenyl-3-(trimethylsilyl)prop-2-yn-1-yl)-*tert*-butylsulfinamide (**6i**)

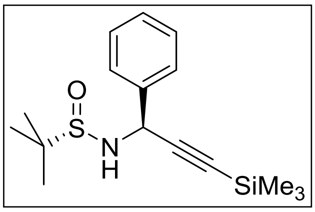

Synthesis: GP-4, reaction scale: 14.0 mmol of imine **5i**. Purification by column chromatography (PE/EtOAc/NEt$_3$, 85:14:1).

Yellow oil, yield: 2.34 g, 7.56 mmol, 54 %. *dr* = 95:5. ^{1}H NMR (500 MHz, Chloroform-*d*): δ = 7.50-7.49 (m, 2H, ar-2-**H**, ar-6-**H**), 7.38-7.31 (m, 3H, ar-3-**H**, ar-4-**H**, ar-5-**H**), 5.24 (d, ^{3}J = 5.2 Hz, 1H, C$^{\alpha}$**H**), 3.68 (d, ^{3}J = 5.2 Hz, 1H, N**H**), 1.20 (s, 9H, SC(C**H**$_3$)$_3$), 0.19 (s, 9H, Si(C**H**$_3$)$_3$). ^{13}C NMR (125 MHz, Chloroform-*d*): δ = 139.0 (ar-**C**-1), 128.7 (ar-**C**-3, ar-**C**-5), 128.4 (ar-**C**-2, ar-**C**-6), 128.0 (ar-**C**-4), 104.0 (C$^{\alpha}$**C**≡CTMS), 91.5 (C$^{\alpha}$C≡**C**TMS), 56.5 (S**C**(CH$_3$)$_3$), 51.1 (**C**$^{\alpha}$), 22.7 (SC(**C**H$_3$)$_3$), -0.1 (Si(**C**H$_3$)$_3$). $C_{16}H_{25}NOSSi$ (307.53 g mol^{-1}). TLC: R_f (PE/EtOAc/NEt$_3$, 85:14:1) = 0.38.

(*S*)-*N*-((*S*)-1-(Pentafluorophenyl)-3-(trimethylsilyl)prop-2-yn-1-yl)-*tert*-butylsulfinamide (**6j**)

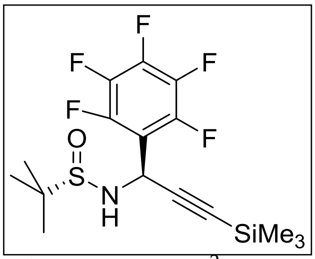

Synthesis: GP-4, reaction scale: 3.12 mmol of imine **5j**. Purification by column chromatography (PE/EtOAc, 2:1).

Colourless crystals, yield: 1.30 g, 3.28 mmol, 41 %, *dr* = 100:0. ^{1}H NMR (500 MHz, Chloroform-*d*) δ = 5.58 (d, $^{3}J_{HH}$ = 5.3 Hz, 1H, C$^{\alpha}$**H**), 3.87 (d, $^{3}J_{HH}$ = 5.4 Hz, 1H, C$^{\alpha}$N**H**), 1.15 (s, 9H, SC(C**H**$_3$)$_3$), 0.14 (s, 9H, Si(C**H**$_3$)$_3$). ^{19}F NMR (282 MHz, Chloroform-*d*) δ = -141.9 (dt, $^{3}J_{FF}$ = 13.9 Hz, $^{4}J_{FF}$ = 1.9 Hz, 2F, ar-2-**F**, ar-6-**F**), -153.4 (tt, $^{3}J_{FF}$ = 21.0 Hz, $^{4}J_{FF}$ = 2.1 Hz, 1F, ar-4-**F**), -161.0 (dd, $^{3}J_{FF}$ = 20.9 Hz, $^{3}J_{FF}$ = 13.8 Hz, 2F, ar-3-**F**, ar-5-**F**). $C_{16}H_{20}F_5NOSSi$ (397.48 g mol^{-1}).

(*S*)-*N*-((*R*)-1,1,1-Trifluoro-4-(trimethylsilyl)but-3-yn-2-yl)-*tert*-butylsulfinamide (**6k**)

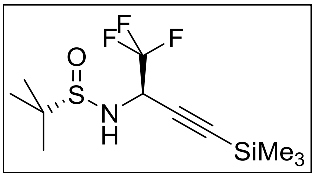

Synthesis: modified GP-4. A solution of *n*-Butyl lithium (1.6 M in *n*-hexane, 20 mL, 32 mmol, 1.5 equiv) was added dropwise to a solution of trimethylsilylethyne (3.152 g, 32.1 mmol, 1.5 equiv) in toluene at −78 °C and the solution was stirred for 2 h at the same temperature. Afterwards, a solution of crude **5k** (21.86 mmol, 1 equiv) in toluene was added dropwise and the reaction mixture was stirred for another 2 h, before warming up to rt. A saturated aqueous solution of NH_4Cl (40 mL) was added, the phases separated and the aqueous layer was extracted with Et_2O (4 × 75 mL). The combined organic layers were washed with brine (15 mL), dried over $MgSO_4$ and the solvent was removed under reduced pressure. The crude product was obtained in the form of a brown oil. It was filtered through a short column of silica gel with EtOAc/PE, 1:2, to yield TMS protected alkyne **6k** in pure form.

Colourless crystals, yield: 2.45 g, 8.18 mmol, 33 % (referred to sulfinamide **(*S*)-1**). ^{1}H NMR (300 MHz, Chloroform-*d*) δ = 4.51 (qd, $^{3}J_{HF}$ = 6.4 Hz, $^{3}J_{HH}$ = 7.2 Hz, 1H, C$^{\alpha}$**H**), 3.82 (d, $^{3}J_{HH}$ = 7.2 Hz, 1H, C$^{\alpha}$N**H**), 1.20 (s, 9H, C(C**H**$_3$)$_3$), 0.14 (s, 9H, Si(C**H**$_3$)$_3$). ^{19}F NMR (282 MHz, Chloroform-*d*) δ = -75.94 (d, $^{3}J_{FH}$ = 6.3 Hz, C**F**$_3$). $C_{11}H_{20}F_3NOSSi$ (299.43 g mol^{-1}). MS(ESI): *m/z* = 322.154 (322.088 [M+Na]$^+$). TLC: R$_f$ (PE/EtOAc, 2:1) = 0.61.

When hemiaminal **8k** was used instead of imine **5k**, the yield was considerably reduced to 24.2 mg, 106 μmol, 5 %. When imine **5k** was used, and $AlMe_3$ was added as a Lewis acid, as described in GP-4, the yield was also notably decreased: 1.384 g, 0.414 mmol, 22 %.

(*S*)-*N*-((*R*)-1,1,1-Trichloro-4-(trimethylsilyl)but-3-yn-2-yl)-*tert*-butylsulfinamide (**6l**)

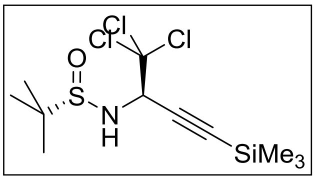

Synthesis: GP-4, reaction scale: 1.50 mmol of imine **5l**. Conversion: 68 % (Isolation of 32 % starting material). When **(*S*)-1** was used, nucleophilic attack from the *si*-face could not be observed at all. The new stereo center only formed in (*R*)-configuration. Compound **5l** was purified by column chromatography (PE/EtOAc, 4:1).

Colourless crystals, yield: 50.6 mg, 0.15 mmol, 10 %. ^{1}H NMR (500 MHz, Chloroform-*d*) δ = 4.69 (d, ^3J = 8.0 Hz, 1H, C^α**H**), 3.97 (d, ^3J = 7.9 Hz, 1H, N**H**), 1.28 (s, 9H, SC(C**H$_3$**)$_3$), 0.18 (s, 9H, Si(C**H$_3$**)$_3$). ^{13}C NMR (126 MHz, Chloroform-*d*) δ = 101.2 (Cl$_3$**C**), 98.5 (C^α**C**≡CTMC), 94.7 (C^{α}C≡**C**TMS), 65.3 (**C**^{α}), 57.9 (S**C**(CH$_3$)$_3$), 22.9 (SC(**C**H$_3$)$_3$), -0.4 (Si(**C**H$_3$)$_3$). $C_{11}H_{20}Cl_3NOSSi$ (348.78 g mol^{-1}). MS(ESI): *m/z* = 243.9888 (243.9877 [$C_7H_{12}Cl_3NSi$]$^+$). TLC: R$_f$ (PE/EtOc, 4:1) = 0.55.

(*R*)-*N*-((*S*)-1-(Benzyloxy)-4-(trimethylsilyl)but-3-yn-2-yl)-*tert*-butylsulfinamide (**6n**)

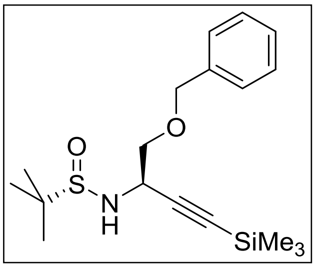

Synthesis: GP-3, reaction scale: 197 μmol of imine **5n**. The crude product **6n** was directly converted into propargylamine **7n**, without further purification or characterization.

(*S*)-*N*-((*R*)-1-(Allyloxy)-4-(trimethylsilyl)but-3-yn-2-yl)-*tert*-butylsulfinamide (**6o**)

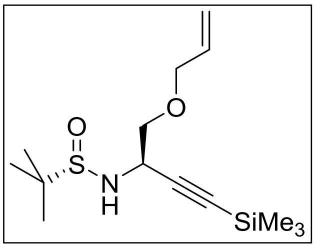

Synthesis GP-4, reaction scale: 1.58 mmol of imine **5o**. The crude product was directly converted to propargylamine **7o** without further purification. The diastereoselectivity of the reaction could be determined by ^{1}H NMR spectroscopy of the crude product.

Dark yellow oil, yield: not determined, *dr* = 89:11, ^{1}H NMR (500 MHz, Chloroform-*d*): δ = 5.87 (m, 1H, C**H**=CH$_2$), 5.27 (dddd, ^4J = 1.3 Hz, ^4J = 1.6 Hz, ^2J = 1.6 Hz, ^3J = 17.2 Hz, 1H, C=H$_Z$C**H**$_E$), 5.17 (dddd, ^4J = 1.0 Hz, ^4J = 1.6 Hz, ^2J = 1.7 Hz, ^3J = 10.4 Hz, 1H, C=**H**$_Z$CH$_E$), 4.22 (m, 1H, C^α**H**), 4.04-4.03 (m, 2H, C**H$_2$**CH=), 3.70 (d, ^3J = 5.2 Hz, 1H, N**H**), 3.59-3.56 (m,

2H, HC$^{\alpha}$C**H$_2$**), 1.20 (s, 9H, S(C(C**H$_3$**)$_3$), 0.14 (s, 9H, Si(C**H$_3$**)$_3$). $C_{14}H_{27}NO_2SSi$ (301.52 g mol^{-1}).

(*R*)-*N*-((*S*)-1-(Benzylthio)-4-(trimethylsilyl)but-3-yn-2-yl)-*tert*-butylsulfinamide (**6p**)

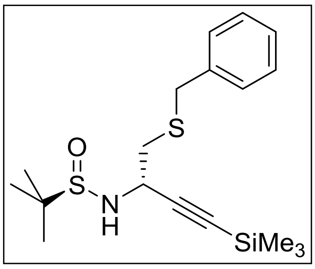

Synthesis: GP-3, reaction scale: 4.45 mmol of imine **5p**. Compound **6p** was directly converted to propargylamine **7p** without further purification or characterization.

(*S*)-*N*-((*S*)-6-Cyano-1-(trimethylsilyl)hex-1-yn-3-yl)-*tert*-butylsulfinamide (**6q**)

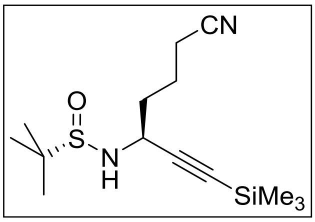

Synthesis: GP-4, reaction scale: 5.02 mmol of imine **5q**. The crude product was directly converted to propargylamine **7q** without further purification or characterization.

Dark yellow oil, yield (crude) 1.0491 g, 3.514 mmol, 70 %. $C_{14}H_{26}N_2OSSi$ (298.52 g mol^{-1}).

tert-Butyl (*S*)-4-(((*S*)-*tert*-butylsulfinyl)amino)-6-(trimethylsilyl)hex-5-ynoate (**6s**)

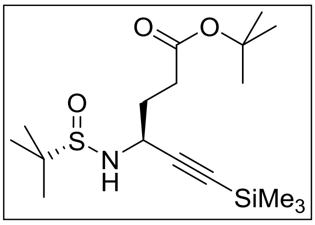

Synthesis: GP-4, reaction scale: 4.06 mmol of imine **5s**. A Lewis Acid (AlMe$_3$) was left out. Isolation by fractionated filtration through a short column with silica gel. (EtOAc/PE, 10:1 – 4:1 – 2:1 - 1:1).

Colourless crystals, yield: 0.773 g, 2.15 mmol, 53 %, *dr* = 93:7. ^{1}H NMR (500 MHz, Chloroform-*d*) δ = 4.14 (ddd, ^3J = 7.4 Hz, ^3J = 5.9 Hz, ^3J = 5.9 Hz, 1H, C$^{\alpha}$**H**), 3.37 (d, ^3J = 5.6 Hz, 1H, N**H**), 2.46-2.34 (m, 2H, C$^{\alpha}$CH$_2$C**H$_2$**), 2.02 (m, 1H, C$^{\alpha}$C**H**$_2$), 1.93 (ddt, ^2J = 13.7 Hz, ^3J = 8.5 Hz, ^3J = 6.7 Hz, 1H, C$^{\alpha}$C**H**$_2$), 1.83 (ddt, ^2J = 13.1 Hz, ^3J = 8.3 Hz, ^3J = 6.8 Hz, 1H, C$^{\alpha}$C**H**$_2$), 1.45 (s, 9H, CO$_2$C(C**H$_3$**)$_3$), 1.22 (s, 9H, SC(C**H$_3$**)$_3$), 0.17 (s, 9H, Si(C**H$_3$**)$_3$). ^{13}C NMR (126 MHz, Chloroform-*d*) δ = 172.7 (**C**O$_2$), 104.1 (CO$_2$**C**Me$_3$), 89.9 (C$^{\alpha}$**C**≡CTMS), 80.5 (C$^{\alpha}$C≡**C**TMS), 56.0 (S**C**(CH$_3$)$_3$), 47.1 (**C**$^{\alpha}$H), 31.6 (C$^{\alpha}$CH$_2$**C**H$_2$), 28.1

($CO_2C(\mathbf{CH_3})_3$), 27.5 ($C^{\alpha}\mathbf{C}H_2$), 22.4 ($SC(\mathbf{C}H_3)_3$), -0.3 ($SiC(\mathbf{C}H_3)_3$). $C_{17}H_{33}NO_3SSi$ (359.60 g mol^{-1}). MS(ESI): *m/z* = 360.479 (360.203 $[M+H]^+$). TLC: R_f (EtOAc/PE, 1:1) = 0.78.

(*S*)-*N*-((*S*)-6-((*tert*-Butyldimethylsilyl)oxy)-1-(trimethylsilyl)hex-1-yn-3-yl)-*tert*-butylsulfinamide (**6t**)

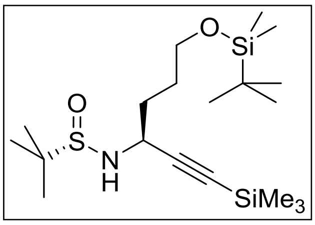

Synthesis: GP-3, reaction scale: 3.67 mmol of imine **5t**. The crude product was directly applied for the following desilylation reaction without further purification. Compound **6t** has already been described by Bauer, DiBlasi and Tan [45].

Brown oil, yield: not determined. ^1H NMR (500 MHz, Chloroform-*d*) δ = 4.11 (t, ^3J = 5.6 Hz, 1H, $C^{\alpha}\mathbf{H}$), 3.64 (t, ^3J = 6.1 Hz, 2H, $SiOC\mathbf{H_2}$), 3.38 (d, ^3J = 5.3 Hz, 1H, $C^{\alpha}N\mathbf{H}$), 1.81 (m, 1H, $C^{\alpha}C\mathbf{H_2}$), 1.74-1.64 (m, 2H, $C^{\alpha}C\mathbf{H_2}$, $C^{\alpha}CH_2C\mathbf{H_2}$), 1.57 (m, 1H, $C^{\alpha}CH_2C\mathbf{H_2}$), 1.21 (s, 9H, $SC(C\mathbf{H_3})_3$), 0.89 (s, 9H, $SiC(C\mathbf{H_3})_3$), 0.16 (s, 9H, $Si(C\mathbf{H_3})_3$), 0.05 (s, 6H, $Si(C\mathbf{H_3})_2$). $C_{19}H_{41}NO_2SSi_2$ (403.77 g mol^{-1}). MS(ESI): *m/z* = 404.141 (404.2469 $[M+H]^+$).

(*R*)-*N*-((*R*)-7-Azido-1-(trimethylsilyl)hept-1-yn-3-yl)-*tert*-butylsulfinamide (**6v**)

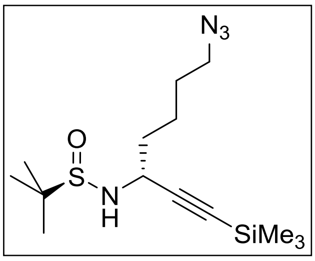

Synthesis: GP-4, reaction scale: 1.16 mmol of imine **5v**. Compound **6v** was directly converted to propargylamine **7vx** without further purification.

Yellow oil, yield not determined. ^1H NMR (300 MHz, Chloroform-*d*) δ = 4.08 (m, 1H, $C^{\alpha}\mathbf{H}$), 3.37 (d, ^3J = 5.2 Hz, 1H, N**H**), 3.34-3.24 (m, 2H, $N_3C\mathbf{H_2}$), 2.55 (m, 1H, $C^{\alpha}C\mathbf{H_2}$), 1.86-1.50 (m, 5H, $C^{\alpha}C\mathbf{H_2}C\mathbf{H_2}C\mathbf{H_2}$), 1.21 (s, 9H, $C(C\mathbf{H_3})_3$), 0.15 (s, 9H, $Si(C\mathbf{H_3})_3$). $C_{14}H_{28}N_4OSSi$ (328.55 g mol^{-1}). MS(ESI): *m/z* = 329.085 (329.185 $[M+H]^+$).

Propargylamines 7

When compound **6** was not isolated, the yield of propargylamine **7** usually refers to imine **5** (2 steps, mentioned in parentheses).

(*S*)-*N*-((*S*)-But-3-yn-2-yl)-*tert*-butylsulfinamide (**7a**)

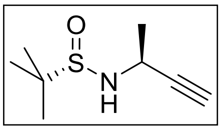

Synthesis: GP-5, starting material was the crude product of **6a**. Reaction scale: 12.7 mmol of imine **5a**. Purification by column chromatography (PE/EtOAc, 2:1 or Et_2O) and recrystallization from DCM or Et_2O. Colourless, crystalline solid. Yield = 1.03 g, 5.96 mmol, 47 % (over two steps, referred to aldimine **5a**). dr: 100:0. ^{1}H NMR (500 MHz, Chloroform-*d*) δ [ppm] = 4.16 (m, 1H, C^{α}**H**), 3.38 (d, ^{3}J = 4.4 Hz, 1H, N**H**), 2.39 (d, ^{4}J = 2.3 Hz, 1H, C≡C**H**), 1.46 (d, ^{3}J = 6.8 Hz, 3H, $C^{\alpha}C\mathbf{H_3}$), 1.21 (s, 9H, $C(C\mathbf{H_3})_3$). ^{13}C NMR (126 MHz, Chloroform-*d*) δ [ppm] = 84.8 (C^{α}C≡**C**H), 72.3 (C^{α}**C**≡CH), 56.1 (**C**$(CH_3)_3$), 42.7 (**C**$^{\alpha}$), 23.3 (**C**H_3), 22.6 (C(**C**$H_3)_3$). $C_8H_{15}NOS$ (173.27 g mol^{-1}). MS(ESI): *m/z* = 196.0757 (196.0767 $[M+Na]^+$). de = 100 %. IR(ATR): $\tilde{\nu}$[cm-1] = 3725 (CH), 3706 (CH), 3623 (CH), 3595 (CH), 3212 (CH), 2946 (CH), 1125 (SO), 1024 (SC), 1014, 846, 699. $[\alpha]_{589}^{22}$ = 55.15 (*c* = 0.65; $CHCl_3$). Smp.: 43-44 °C.

(*S*)-*N*-((*S*)-4-Methylpent-1-yn-3-yl)-*tert*-butylsulfinamide (**7b**)

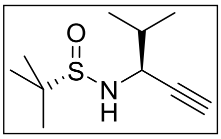

Synthesis: GP-5, starting material was the crude product of **6b**. Reaction scale: 22.5 mmol of imine **5b**. Purification by column chromatography (PE/EtOAc, 2:1) and recrystallization from Et_2O or DCM.

Colourless crystalline solid, yield: 2.77 g, 13.7 mmol, 61 % (over two steps, referred to imine **5b**), *dr* = 97:3. ^{1}H NMR (Chloroform-*d*, 600 MHz): δ = 3.89 ppm (ddd, ^{3}J = 7.4 Hz, ^{3}J = 5.2 Hz, ^{4}J = 2.3 Hz, 1H, C^{α}**H**), 3.31 (d, ^{3}J = 7.1, 1H, N**H**), 2.40 (d, ^{4}J = 2.3 Hz, 1H, C^{α}C≡C**H**), 1.94 (m, 1H, C**H**$(CH_3)_2$), 1.23 (s, 9H, $C(C\mathbf{H_3})_3$), 1.01 (d, ^{3}J = 6.6 Hz, 3H, $CHC\mathbf{H_3}$), 1.00 (d, ^{3}J = 6.6 Hz, 3H, $C^{\alpha}HC\mathbf{H_3}$). ^{13}C NMR (125 MHz, Chloroform-*d*): δ [ppm] = 82.4 (C^{α}**C**≡CH), 73.9 (C^{α}C≡**C**H), 56.4 (**C**$(CH_3)_3$), 53.7 (**C**$^{\alpha}$), 33.6 ($C^{\alpha}\mathbf{C}^{\beta}H(CH_3)_2$), 22.7 (C(**C**$H_3)_3$), 18.9 ($(CH_3)CH\mathbf{C}H_3$), 17.5 ($(CH_3)CH\mathbf{C}H_3$). $C_{10}H_{19}NOS$ (201.33 g mol^{-1}). MS(ESI): *m/z* = 425.22789 (calc. 425.22668 $[2M+Na]^+$). TLC: R_f (PE:EE 2:1) = 0.38. $[\alpha]_{589}^{20}$ = 42.4 (*c* = 2.82; $CHCl_3$). EA: C(61.083%), H(9.618%), N(6.322%), S(14.249%).

(*S*)-*N*-((*S*)-5-Methylhex-1-yn-3-yl)-*tert*-butylsulfinamide (**7c**)

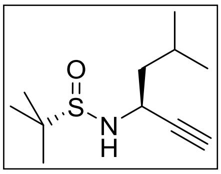

Synthesis: GP-5, starting material was the crude product of **6c**. Reaction scale: 7.81 mmol of imine **5c**. Isolation by column chromatography (EtOAc/PE, 1:2). Recrystallization from Et_2O or DCM. Compound **7c** was first described by Ye, He and Zhang [29], as well as Burke, Cogan, Gao, Heim-Riether, Eugene, Ramsden, Thompson and Xiong [49].

Colourless crystalline solid, yield: 1.03 g, 4.14 mmol, 53 % (over two steps, referred to imine **5c**). *dr* = 97:3. ^{1}H NMR (300 MHz, Chloroform-*d*): δ = 4.03 (m, 1 H, C$^{\alpha}$**H**), 3.26 (d, ^3J = 7.5 Hz, 1 H, N**H**), 2.41 (d, ^4J = 2.3 Hz, 1H, C$^{\alpha}$C≡C**H**), 1.86 (m, 1H, $(CH_3)_2$C**H**), 1.59 (t, ^3J = 7.4 Hz, 2H, C$^{\alpha}$C**H$_2$**), 1.21 (s, 9 H, SC(C**H$_3$**)$_3$), 0.93 (d, ^3J = 6.6 Hz, 3H, CHC**H$_3$**), 0.93 (d, ^3J = 6.6 Hz, 3H, CHC**H**$_3$). ^{13}C NMR (125 MHz, Chloroform-*d*): δ = 84.2 (C$^{\alpha}$**C**≡CH), 73.1 (C$^{\alpha}$C≡**C**H), 56.4 (**C**$(CH_3)_3$), 46.4 (**C**$^{\alpha}$), 46.2 (**C**H_2), 24.9 (H**C**$(CH_3)_2$), 22.7 (C(**C**H_3)$_3$), 22.6 ((CH_3)CH**C**H_3), 22.2 ((CH_3)CH**C**H_3). $C_{11}H_{21}NOS$ (215.36 g mol^{-1}). MS(ESI): *m/z* = 453.25884 (453.25799 $[2M+Na]^+$). $[\alpha]_{589}^{20}$ = 26.1 (*c* = 1.94; $CHCl_3$). TLC: R_f (PE/EtOAc, 2:1) = 0.27. EA: C (60.703%), H (9.790%), N (6.574%), S (14.195%).

(*S*)-*N*-((*S*)-1-Cyclohexylprop-2-yn-1-yl)-*tert*-butylsulfinamide (**7d**)

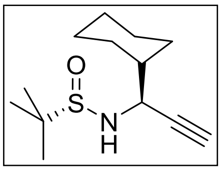

Synthesis: GP-5, starting material was the crude product of **6d**. Reaction scale: 11.1 mmol of imine **5d**. Purification by column chromatography (EtOAc/PE, 1:2). Recrystallization from EtOAc/PE (1:4). Compound **7d** has been first described by Jordan, Starks, Whatley and Turlington [50].

Colourless, crystalline solid, yield: 1.741 g, 7.212 mmol, 65 % (59 % over two steps, referred to imine **5d**). *dr* = 97:3. ^{1}H NMR (500 MHz, Chloroform-*d*) δ = 3.85 (ddd, ^3J = 7.7 Hz, ^3J = 5.7 Hz, ^4J = 2.3 Hz, 1H, C$^{\alpha}$**H**), 3.28 (d, ^3J = 7.5 Hz, 1H, N**H**), 2.41 (d, ^4J = 2.3 Hz, 1H, C$^{\alpha}$C≡C**H**), 1.82 (d, ^2J = 12.2 Hz, 2H, cy-2-**H**, cy-6-**H**), 1.77 (d, ^2J = 11.6 Hz, 2H, cy-3-**H**, cy-5-**H**), 1.67 (d, ^2J = 11.9 Hz, 2H, cy-4-**H**), 1.59 (ddd, ^2J = 11.5 Hz, ^3J = 6.0 Hz, ^4J = 3.2 Hz, 1H, cy-1-**H**), 1.29-1.23 (m, 2H, cy-5-**H**, cy-3-**H**), 1.22 (s, 9H, C(C**H$_3$**)$_3$), 1.15 (ddd, ^2J = 15.8 Hz, ^3J = 7.9 Hz, ^3J = 3.9 Hz, 1H, cy-6-**H**), 1.09 (dd, ^2J = 12.2 Hz, ^3J = 3.4 Hz, 1H, cy-2-**H**). ^{13}C NMR (126 MHz, Chloroform-*d*) δ = 82.9 (C$^{\alpha}$C≡**C**H), 73.9 (C$^{\alpha}$**C**≡CH), 56.5

($C(CH_3)_3$), 53.1 (C^{α}), 43.2 (cy-**C**-1), 29.4 (cy-**C**-3), 28.3 (cy-**C**-5), 26.4 (cy-**C**-6), 26.1 (cy-**C**-2), 26.0 (cy-**C**-4), 22.8 ($C(CH_3)_3$). $C_{13}H_{23}NOS$ (241.39 g mol^{-1}). MS(ESI): *m/z* = 264.1 (264.14 $[M+Na]^+$). IR(ATR): $\tilde{\nu}$[cm^{-1}] = 3217 (NH), 2848 (CH), 1445 (SO), 1059 (SC), 897, 674. Smp. = 59 °C. $[\alpha]^{20}_{589}$ = 32.9 (*c* = 0.82; $CHCl_3$).

(*S*)-*N*-((*S*)-4,4-Dimethylpent-1-yn-3-yl)-*tert*-butylsulfinamide (**7e**)

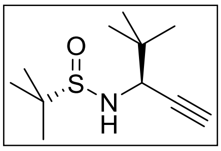

Synthesis: GP-5, starting material was the crude product of **6e**. Reaction scale: 2.98 mmol of imine **5e**. The crude product was purified by column chromatography (PE/EtOAc, 2:1) and recrystallization from EtOAc. Compound **7e** has been first described by Burke, Cogan, Gao, Heim-Riether, Hickey, Ramsden, Thompson and Xiong [49].

Colourless crystals, yield: 577 mg, 2.68 mmol, 90 % (41 % over two steps, referred to imine **5e**), *dr* = 80:20. ^{1}H NMR (500 MHz, Chloroform-*d*) δ = 3.66 (dd, ^3J = 9.3 Hz, ^4J = 1.7 Hz, 1H, C^{α}**H**), 3.24 (d, ^3J = 9.3 Hz, 1H, N**H**), 2.44 (d, ^4J = 2.0 Hz, 1H, $C^{\alpha}C{\equiv}C$**H**), 1.24 (s, 9H, $SC(CH_3)_3$), 1.00 (s, 9H, $C^{\alpha}C(CH_3)_3$). ^{13}C NMR (126 MHz, Chloroform-*d*) δ = 83.0 (C^{α}**C**$\equiv$CH), 74.2 ($C^{\alpha}C{\equiv}$**C**H), 58.4 (C^{α}**C**$(CH_3)_3$), 56.9 (S**C**$(CH_3)_3$), 36.2 (**C**$^{\alpha}$), 26.1 ($C^{\alpha}C$(**C**$H_3)_3$), 22.9 (SC(**C**$H_3)_3$). $C_{11}H_{21}NOS$ (215.36 g mol^{-1}). MS(ESI): *m/z* = 216.1434 (216.1422 $[M+H]^+$). TLC: R_f(EtOAc/PE, 1:4) = 0.08.

(*S*)-*N*-((*S*)-1-((3*S*,5*S*,7*S*)-Adamantane-1-yl)prop-2-yn-1-yl)-*tert*-butylsulfinamide (**7f**)

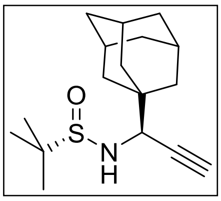

Synthesis: GP-5, starting material was the crude product of **6f**. Reaction scale: 853 μmol of imine **5f**. Purification by column chromatography (PE/EtOAc, 1:1) and recrystallization from *n*-hexane/Et_2O (1:1). The formation of the undesired diastereomer could not be observed in this case (checked by NMR spectroscopy of the crude product).

Colourless, crystalline solid, yield: 37.6 mg, 128 μmol, 15 % (over 2 steps, referred to imine **5f**), *dr* = 100:0. ^{1}H NMR (500 MHz, Chloroform-*d*) δ = 3.51 (dd, ^3J = 9.3, ^4J = 2.2 Hz, 1H,

C^{α}**H**), 3.21 (d, ^{3}J = 9.3 Hz, 1H, $C^{\alpha}N$**H**), 2.43 (d, ^{4}J = 2.3 Hz, 1H, C≡C**H**), 2.01 (s, 3H, $C^{\alpha}C(CH_2$**C****H**$CH_2)_3$), 1.71 (d, ^{2}J = 12.2 Hz, 3H, $C^{\alpha}C(C$**H**$_2CHCH_2)_3$), 1.66 (d, ^{2}J = 12.4 Hz, 3H, $C^{\alpha}C(CH_2CHC$**H**$_2)_3$), 1.61 (d, ^{2}J = 12.0 Hz, 3H, $C^{\alpha}C(C$**H**$_2CHCH_2)_3$), 1.55 (d, ^{2}J = 11.8 Hz, 3H, $C^{\alpha}C(CH_2CHC$**H**$_2)_3$), 1.23 (s, 9H, SC(C**H**$_3)_3$). ^{13}C NMR (126 MHz, Chloroform-*d*) δ = 82.1 (C^{α}**C**≡CH), 74.6 (C^{α}C≡**C**H), 58.6 (**C**$^{\alpha}$), 57.0 (S**C**$(CH_3)_3$), 38.6 ($C^{\alpha}C(CH_2CH$**C**$H_2)_3$), 37.6 (C^{α}**C**$(CH_2CHCH_2)_3$), 37.0 ($C^{\alpha}C($**C**$H_2CHCH_2)_3$), 28.4 ($C^{\alpha}C(CH_2$**C**$HCH_2)_3$), 22.9 (C(**C**$H_3)_3$). $C_{17}H_{27}NOS$ (293.47 g mol^{-1}), MS(ESI): *m/z* = 294.1886 (294.1886 $[M+H]^+$). TLC: R_f (EtOAc/PE, 1:1) = 0.32. $[\alpha]_{589}^{22}$ = 3.45 (*c* = 1.89; $CHCl_3$).

(*R*)-*N*-((*R*)-5-(Methylthio)pent-1-yn-3-yl)-*tert*-butylsulfinamide (**7g**)

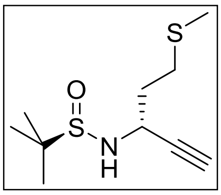

Synthesis: GP-6, starting material was the crude product of **6g**. Reaction scale: 12.6 mmol of imine **5g**. Both stereocenters are (*R*)-configured. Purification by column chromatography (EtOAc/PE, 1:2).

Highly viscous, colourless oil, yield: 999 mg, 4.284 mmol, 34 % (over two steps, referred to imine **5g**) *dr* = 96:4. ^{1}H NMR (500 MHz, Chloroform-*d*) δ = 4.23 (m, 1H, C^{α}**H**), 3.59 (d, ^{3}J = 7.2 Hz, 1H, N**H**), 2.72- 2.62 (m, 2H, $(CH_3)SC$**H**$_2$), 2.46 (d, ^{4}J = 2.3 Hz, 1H, C^{α}C≡C**H**), 2.11 (s, 3H, SC**H**$_3$), 2.04 (dd, ^{2}J = 14.4 Hz, ^{3}J = 3.0 Hz, 2H, $C^{\alpha}C$**H**$_2$), 2.01 (dd, ^{2}J = 14.3 Hz, ^{3}J = 3.2 Hz, 2H, $C^{\alpha}C$**H**$_2$), 1.23 (s, 9H, SC(C**H**$_3)_3$). ^{13}C NMR (126 MHz, Chloroform-*d*) δ = 83.1 (C^{α}**C**≡CH), 73.7 (C^{α}C≡**C**H), 56.4 (S**C**$(CH_3)_3$), 46.6 (**C**$^{\alpha}$), 35.9 ($C^{\alpha}H$**C**H_2), 30.0 ($(CH_3)S$**C**H_2), 22.6 (SC(**C**$H_3)_3$), 15.5 (S**C**H_3). $C_{10}H_{19}NOS_2$ (233.39 g mol^{-1}). MS(ESI): *m/z* = 256.0798 (256.0800 $[M+Na]^+$). IR(ATR): $\tilde{\nu}$[cm^{-1}] = 3208 (NH), 2958 (CH), 2917 (CH), 2863 (CH), 1736 (SO), 1648 (CSC), 1546 (SC), 1477, 1454, 1432, 1363, 1315, 1264, 1223, 1176, 1059, 936, 669, 647. $[\alpha]_{589}^{20}$ = -54.84 (*c* = 0.46; $CHCl_3$). TLC: R_f (PE/EtOAc, 2:1) = 0.14.

(*S*)-*N*-[(*S*)-1-Phenylbut-3-yn-2-yl]-*tert*-butylsulfinamide (**7h**)

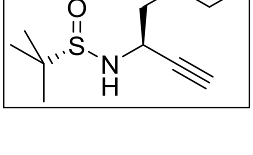

Synthesis: GP-5, starting material was the crude product of **6h**. Reaction scale: 1.52 mmol of imine **5h**. Purification by column chromatography (PE/EtOAc, 2:1). Compound **7h** was first described by Burke, Cogan, Gao, Heim-Riether, Hickey, Ramsden, Thompson, Xiong [49].

Yellow oil, yield: 249 mg, 1.00 mmol, 35 % (when the crude material of **6h** was used in this conversion, the yield was decreased to 234 mg, 938 μmol, 15 % over two steps, ref. to imine **5h**). *dr* = 97:3. ^{1}H NMR (500 MHz, Chloroform-*d*): δ = 7.33-7.29 (m, 2H, ar-2-**H**, ar-6-**H**), 7.27-7.25 (m, 3H, ar-3-**H**, ar-4-**H**, ar-5-**H**), 4.27 (m, 1H, C$^{\alpha}$**H**), 3.36 (d, ^{3}J = 7.2 Hz, 1H, N**H**), 3.07-3.00 (m, 2H, C$^{\alpha}$C**H$_2$**), 2.45 (d, ^{4}J = 2.3 Hz, 1H, C≡C**H**), 1.14 (s, 9H, SC(C**H$_3$**)$_3$). ^{13}C NMR (125 MHz, Chloroform-*d*): δ = 136.4 (ar-**C**-1), 130.0 (ar-**C**-3, ar-**C**-5), 128.5 (ar-**C**-2, ar-**C**-6), 127.2 (ar-**C**-4), 83.2 (C$^{\alpha}$**C**≡CH), 74.3 (C$^{\alpha}$C≡**C**H), 56.5 (S**C**(CH$_3$)$_3$), 49.0 (**C**$^{\alpha}$), 43.3 (C$^{\alpha}$**C**H$_2$), 22.6 (SC(**C**H$_3$)$_3$). $C_{14}H_{19}NOS$ (249.37 g mol^{-1}). MS(ESI): *m/z* = 250.0 (250.13 [M+H]$^{+}$). TLC: R$_f$ (PE/EtOAc, 2:1) = 0.14. $[\alpha]_{589}^{20}$ = 18.6 (*c* = 1.0; CHCl$_3$).

(*S*)-*N*-((*R*)-1-Phenylprop-2-yn-1-yl)-*tert*-butylsulfinamide (**7i**)

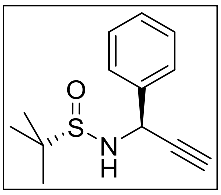

Synthesis: GP-7, starting material was the crude product of **6i**. Reaction scale: 8.30 mmol of imine **5i**. Purification by column chromatography (PE/EtOAc, 2:1). Recrystallization from Et$_2$O. Compound **7i** has been first described by Verrier, Carret, Poisson [51] and Jordan, Starks, Whatley, Turlington [50].

Yellow solid, yield: 1.29 g, 5.48 mmol, 66 % (36 % over two steps, referred to imine **5i**), *dr* = 95:5. ^{1}H NMR (500 MHz, Chloroform-*d*): δ [ppm] = 7.52-7.50 (m, 2H, ar-2-**H**, ar-6-**H**), 7.38-7.33 (m, 3H, ar-3-**H**, ar-4-**H**, ar-5-**H**), 5.22 (dd, ^{3}J = 6.5 Hz, ^{4}J = 2.4 Hz, 1H, C$^{\alpha}$**H**), 3.75 (d, ^{3}J = 6.4 Hz, 1H, N**H**), 2.64 (d, ^{4}J = 2.4 Hz, 1H, C≡C**H**), 1.21 (s, 9H, S(C(C**H$_3$**)$_3$). ^{13}C NMR (125 MHz, Chloroform-*d*): δ [ppm] = 138.5 (ar-**C**-1), 128.8 (ar-**C**-2, ar-**C**-6), 128.6 (ar-**C**-3, ar-**C**-5), 127.8 (ar-**C**-4), 82.6 (H**C**≡CC$^{\alpha}$), 75.1 (HC≡**C**C$^{\alpha}$), 56.6 (S**C**(CH$_3$)$_3$), 50.8 (NH**C**$^{\alpha}$), 22.7 (SC(**C**H$_3$)$_3$). $C_{13}H_{17}NOS$ (235.35 g mol^{-1}). MS(ESI): *m/z* = 493.19639 (493.19539 [2M+Na]$^{+}$), TLC: R$_f$ (PE/EtOAc, 2:1) = 0.19. $[\alpha]_{589}^{20}$ = 17.4 (*c* = 0.5; CHCl$_3$).

(*S*)-*N*-((*R*)-1-(Pentafluorophenyl)prop-2-yn-1-yl)-*tert*-butylsulfinamide (**7j**)

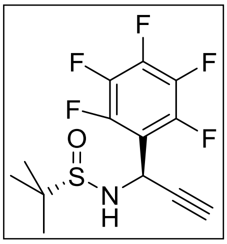

Synthesis: GP-6, starting material was the crude product of **6j**. Reaction scale: 3.12 mmol of imine **5j**. Purification by column chromatography (PE/EtOAc, 2:1). Recrystallization from EtOAc/PE, 1:8.

Colourless crystals, yield: 0.53 g, 1.62 mmol, 52 % (21 % over two steps,

referred to imine **5j**). *dr* = 100:0. ^{1}H NMR (600 MHz, Chloroform-*d*) δ = 5.55 (d, $^{3}J_{HH}$ = 5.3 Hz, 1H, C$^{\alpha}$**H**), 4.01 (d, $^{3}J_{HH}$ = 5.4 Hz, 1H, C$^{\alpha}$N**H**), 2.56 (d, $^{4}J_{HH}$ = 2.5 Hz, 1H, C≡C**H**), 1.13 (s, 9H, SC(C**H$_3$**)$_3$). ^{19}F NMR (565 MHz, Chloroform-*d*) δ = -142.0 (td, $^{3}J_{FF}$ = 21.3 Hz, $^{4}J_{FF}$ = 7.7 Hz, 2F, ar-3-**F**, ar-5-**F**), -153.3 (t, $^{3}J_{FF}$ = 18.9 Hz, 1F, ar-4-**F**), -161.0 (t, $^{3}J_{FF}$ = 7.3 Hz, 2F, ar-2-**F**, ar-6-**F**). ^{13}C NMR (151 MHz, Chloroform-*d*) δ = 144.7 (ddt, $^{1}J_{CF}$ = 251.2 Hz, $^{2}J_{CF}$ = 8.1 Hz, $^{3}J_{CF}$ = 3.9 Hz, ar-**C**-3, ar-**C**-5), 141.5 (d, $^{1}J_{CF}$ = 256.1 Hz, ar-**C**-4), 137.7 (dt, $^{1}J_{CF}$ = 252 Hz, $^{2}J_{CF}$ = 15.8 Hz, ar-**C**-2, ar-**C**-6), 113.4 (td, $^{2}J_{CF}$ = 14.8 Hz, $^{3}J_{CF}$ = 3.6 Hz, ar-**C**-1), 79.2 (C$^{\alpha}$**C**≡CH), 74.5 (C$^{\alpha}$C≡**C**H), 56.7 (**C**(CH$_3$)$_3$), 40.9 (**C**$^{\alpha}$), 22.3 (C(**C**H$_3$)$_3$). $C_{13}H_{12}F_5NOS$ (325.28 g mol^{-1}). MS(ESI): *m/z* = 348.0470 (348.0457 [M+Na]$^{+}$). $[\alpha]_{589}^{23}$ = 38.8 (*c* = 0.53; CHCl$_3$). TLC: R$_f$ (PE/EtOAc, 2:1) = 0.32. IR(ATR): $\tilde{\nu}$ [cm^{-1}] = 3306 (NH), 3208 (≡C-H), 2958 (-CH$_3$), 2356-2331 (-C≡C-), 1524-1498 (ar, C=C), 1074 (S=O).

(*S*)-*N*-((*R*)-1,1,1-Trifluorobut-3-yn-2-yl)-*tert*-butylsulfinamide (**7k**)

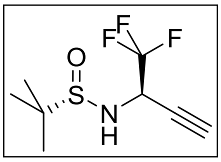

Synthesis: GP-6, starting material was the crude product of **6k**. Reaction scale: 7.21 mmol of imine **5k**. The crude product was washed with pentane and then either purified by column chromatography (EtOAc/PE, 1:2 to 1:1) or by recrystallization from DCM/*n*-hexane (1:1).

Colourless crystals, yield: 474.6 mg, 2.088 mmol, 29 %, (25 % over two steps, referred to sulfinamide **(*S*)-1**) *dr* = 93:7. ^{1}H NMR (600 MHz, Chloroform-*d*) δ = 4.55 (dqd, $^{3}J_{HH}$ = 7.7 Hz, $^{3}J_{HF}$ = 6.3 Hz, $^{4}J_{HH}$ = 2.5 Hz, 1H, C$^{\alpha}$**H**), 3.68 (d, $^{3}J_{HH}$ = 8.0 Hz, 1H, C$^{\alpha}$N**H**), 2.62 (d, $^{4}J_{HH}$ = 2.5 Hz, 1H, C$^{\alpha}$C≡C**H**), 1.26 (s, 9H, C(C**H$_3$**)$_3$). ^{19}F NMR (565 MHz, Chloroform-*d*) δ = -76.51 (d, $^{3}J_{FH}$ = 6.2 Hz, C**F$_3$**). ^{13}C NMR (75 MHz, Chloroform-*d*) δ = 122.9 (q, $^{1}J_{CF}$ = 281.2 Hz, **C**F$_3$), 78.0 (C≡**C**H), 73.7 (q, $^{3}J_{CF}$ = 2.3 Hz, **C**≡CH), 57.0 (**C**(CH$_3$)$_3$), 49.6 (q, $^{2}J_{CF}$ = 35.0 Hz, **C**$^{\alpha}$), 22.4 (C(**C**H$_3$)$_3$). $C_8H_{12}F_3NOS$ (227.25 g mol^{-1}). MS(ESI): *m/z* = 250.0493 (250.0484 [M+Na]$^{+}$). TLC: R$_f$ (EtOAc/PE, 2:7) = 0.27. Smp = 83.7 °C. $[\alpha]_{589}^{22}$ = 52.1 (*c* = 0.97; DCM).

When GP-5 was applied, a far lower yield of propargylamine **7k** was achieved: 24.2 mg, 0.11 mmol, 10 %. When the crude product of **6k** was not purified, but instantly converted by GP-6, the total yield of the whole synthesis could be increased to 1.197 g, 5.267 mmol, 25 % (referred to the chiral sulfinamine **(*S*)-1**, 3 steps).

(*S*)-*N*-((*R*)-1,1,1-Trichlorobut-3-yn-2-yl)-*tert*-butylsulfinamide (**7l**)

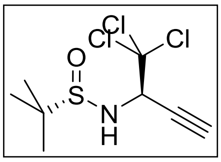

Synthesis: GP-6, starting material was the crude product of **6l**. Reaction scale: 149 µmol of imine **5l**. Reaction was monitored by TLC. Purification by column chromatography (PE/EtOAc, 2:1).

Colourless crystals, yield: 23.5 mg, 85 µmol, 57 % (6 % over two steps, referred to imine **5l**). *dr* = 100:0. ^{1}H NMR (500 MHz, Chloroform-*d*) δ = 4.71 (dd, ^{3}J = 8.6 Hz, ^{4}J = 2.3 Hz, 1H, C^{α}**H**), 4.05 (d, ^{3}J = 8.7 Hz, 1H, N**H**), 2.72 (d, ^{4}J = 2.3 Hz, 1H, C≡C**H**), 1.29 (s, 9H, SC(C**H₃**)₃). ^{13}C NMR (126 MHz, Chloroform-*d*) δ = 100.6 (CCl_3), 77.9 (C^{α}**C**≡CH), 77.4 (C^{α}C≡**C**H), 65.0 (**C**^{α}), 58.0 (**C**(CH_3)₃), 22.8 (C(**C**H_3)₃). $C_8H_{12}Cl_3NOS$ (276.60 g mol^{-1}). MS(ESI): *m/z* = 275.9771 (275.9778 [M+H]$^{+}$). TLC: R_f (EtOAc/PE, 1:2) = 0.27. $[\alpha]_{589}^{22}$ = 15.0 (*c* = 0.59; $CHCl_3$). IR(ATR): $\tilde{\nu}$ [cm^{-1}] = 3281 (NH), 3196 (CH), 3196 (≡C-H), 2360 (-C≡C-), 1068 (S=O), 869, 802, 685.

(*R*)-*N*-((*S*)-1-(Benzyloxy)but-3-yn-2-yl)-*tert*-butylsulfinamide (**7n**)

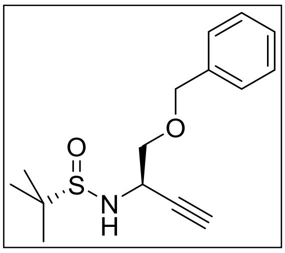

Synthesis: GP-5, starting material was the crude product of **6n**. Reaction scale: 197 µmol of imine **5n**. Purification by column chromatography (EtOAc/PE, 1:4).

Yellow oil, yield: 17 mg, 61 µmol, 31 % (over two steps, referred to imine **5n**), *dr* = 95:5. ^{1}H NMR (500 MHz, Chloroform-*d*) δ = 7.36-7.27 (m, 5H, ar-**H**), 4.64 (d, ^{2}J = 12.0 Hz, 1H, C^{α}C**H**₂), 4.57 (d, ^{2}J = 12.1 Hz, 1H, C^{α}C**H**₂), 4.24 (m, 1H, **C**^{α}), 3.75 (d, ^{3}J = 6.4 Hz, 1H, C^{α}N**H**), 3.65 (dd, ^{3}J = 5.6 Hz, ^{4}J = 1.3 Hz, 2H, PhC**H₂**), 2.45 (d, ^{4}J = 2.4 Hz, 1H, C≡C**H**), 1.21 (s, 9H, C(C**H₃**)₃). ^{13}C NMR (126 MHz, Chloroform-*d*) δ = 137.7 (ar-**C**-1), 128.6 (ar-**C**-3, ar-**C**-5), 128.0 (ar-**C**-4), 127.9 (ar-**C**-2, ar-**C**-6), 81.1 (C^{α}**C**≡CH), 74.2 (C^{α}C≡**C**H), 73.5 (C^{α}**C**H₂), 73.2 (Ph**C**H₂), 56.5 (S**C**(CH_3)₃), 47.5 (**C**^{α}), 22.7 (SC(**C**H_3)₃). $C_{15}H_{21}NO_2S$ (279.40 g mol^{-1}). TLC: R_f (EtOAc/PE, 1:4) = 0.13.

(*S*)-*N*-((*R*)-1-(Allyloxy)but-3-yn-2-yl)-*tert*-butylsulfinamide (**7o**)

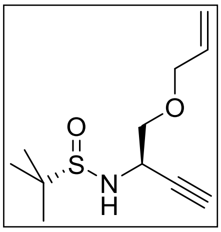

Synthesis: GP-5, starting material was the crude product of **6o**. Reaction scale: 1.58 mmol of imine **5o**. Purification by column chromatography (PE/EtOAC, 2:1 or DCM/MeOH, 98:2).

Yellow oil, yield: 149 mg, 490 µmol, 31 % (over two steps, referred to imine **5o**). *dr* = 93:7. ^{1}H NMR (500 MHz, Chloroform-*d*): δ = 5.86 (m, 1H, C**H**=CH_2), 5.27 (dddd, ^{4}J = 1.4 Hz, ^{4}J = 1.5 Hz, ^{2}J = 1.5 Hz, ^{3}J = 17.2 Hz, 1H, C=H_ZC$\mathbf{H}_E$), 5.18 (dddd, ^{4}J = 1.4 Hz, ^{4}J = 1.4 Hz, ^{2}J = 1.5 Hz, ^{3}J = 10.4 Hz, 1H, C=$\mathbf{H}_Z$CH_E), 4.18 (m, 1H, NHC$^{\alpha}$**H**), 4.04 (m, 2H, C$\mathbf{H_2}$-CH=), 3.72 (d, ^{3}J = 6.4 Hz, 1H, N**H**), 3.58-3.57 (m, 2H, HC$^{\alpha}$C$\mathbf{H_2}$), 2.42 (d, ^{4}J = 2.4 Hz, 1H, C≡C**H**), 1.20 (s, 9H, SC(C$\mathbf{H_3}$)$_3$). ^{13}C NMR (125 MHz, Chloroform-*d*): δ = 134.2 (**C**H=CH_2), 117.6 (CH=**C**H_2), 81.0 (H**C**≡CC$^{\alpha}$), 74.1 (HC≡**C**C$^{\alpha}$), 73.1 (C$^{\alpha}$H**C**H_2), 72.3 (O**C**H_2CH=), 56.4 (S**C**(CH_3)$_3$), 47.4 (**C**$^{\alpha}$), 22.6 (SC(**C**H_3)$_3$). $C_{11}H_{19}NO_2S$ (229.34 g mol^{-1}). MS(ESI): *m/z* = 252.0 (252.33 [M+Na]$^+$). $[\alpha]_{589}^{20}$ = 36.3 (*c* = 0.99; $CHCl_3$). TLC: R_f (DCM/MeOH, 50:1) = 0.17.

(*R*)-*N*-((*S*)-1-(Benzylthio)but-3-yn-2-yl)-*tert*-butylsulfinamide (**7p**)

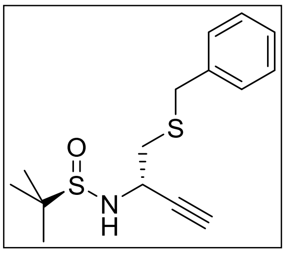

Synthesis: GP-5, starting material was the crude product of **6p**. Reaction scale: 4.45 mmol of imine **5p**. Purification by preparative HPLC.

Brown oil, yield: 57.2 mg, 194 µmol, 4 % (over two steps, referred to imine **5p**). *dr* = 93:7. ^{1}H NMR (300 MHz, Chloroform-*d*) δ = 7.39-7.22 (m, 5H, arC**H**), 4.13 (m, 1H, C$^{\alpha}$**H**), 3.84 (s, 2H, SC$\mathbf{H_2}$Ph), 3.81-3.77 (m, 1H, N**H**), 2.82 (dd, ^{2}J = 13.9 Hz, ^{4}J = 6.3 Hz, 1H, C$^{\alpha}$HC$\mathbf{H}_2$), 2.74 (dd, ^{2}J = 13.8 Hz, ^{3}J = 6.2 Hz, 1H, C$^{\alpha}$HC$\mathbf{H}_2$), 2.50 (d, ^{4}J = 2.3 Hz, 1H, C$^{\alpha}$C≡C**H**), 1.22 (s, 9H, SC(C$\mathbf{H_3}$)$_3$). ^{13}C NMR (151 MHz, Chloroform-*d*) δ = 137.9 (ar-**C**-1), 129.1 (ar-**C**-2, ar-**C**-6), 128.7 (ar-**C**-3, ar-**C**-5), 127.3 (ar-**C**-4), 82.7 (C$^{\alpha}$C≡**C**H), 74.1 (C$^{\alpha}$**C**≡CH), 56.6 (S**C**(CH_3)$_3$), 47.3 (**C**$^{\alpha}$), 38.3 (C$^{\alpha}$H**C**H_2), 36.9 (S**C**H_2Ph), 22.6 (SC(**C**H_3)$_3$). $C_{15}H_{21}NOS_2$ (295.46 g mol^{-1}), MS(ESI): *m/z* = 317.98 (318.10 [M+Na]$^+$). $[\alpha]_{589}^{22}$ = -56.1 (*c* = 0.19; $CHCl_3$). IR(ATR): $\tilde{\upsilon}$ [cm^{-1}] = 3218 (NH), 2958 (CH), 2335 (C≡C).

(*S*)-*N*-((*S*)-6-Cyanohex-1-yn-3-yl)-*tert*-butylsulfinamide (**7q**)

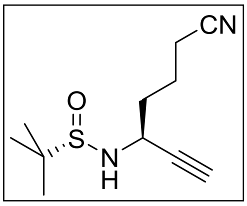

Synthesis: GP-6, starting material was the crude product of **6q**. Reaction scale: 3.53 mmol of imine **5q**. Purification by column chromatography (EtOAc/PE, 1:2).

Yellow crystals, yield: 0.344 g, 1.52 mmol, 43 % (30 % over two steps, referred to imine **5q**). *dr* = 95:5. ^{1}H NMR (500 MHz, Chloroform-*d*): δ = 4.11 (m, 1H, C^α**H**), 3.36 (d, ^3J = 6.1 Hz, 1H, N**H**), 2.46 (d, ^4J = 2.3 Hz, 1H, C≡C**H**), 2.42 (t, ^3J = 6.4 Hz, 2H, C**H$_2$**CN), 1.83-1.95 (m, 4H, CHNC**H$_2$**C**H$_2$**), 1.23 (s, 9H, C(C**H$_3$**)$_3$). ^{13}C NMR (126 MHz, Chloroform-*d*): δ = 77.1 (HC≡**C**C^α), 74.2 (H**C**≡CC^α), 56.5 (**C**(CH$_3$)$_3$), 46.7 (**C**^{α}HNH), 35.5 (CHNCH$_2$**C**H$_2$), 22.7 (C(**C**H$_3$)$_3$), 21.6 (CHN**C**H$_2$CH$_2$), 17.1 (**C**H$_2$CN). $C_{11}H_{18}N_2OS$ (226.34 g mol^{-1}). MS(ESI): *m/z* = 249.1026 (249.1032 [M+Na]$^+$). TLC: R$_f$ (EtOAc/PE, 1:2) = 0.33. $[\alpha]^{20}_{589}$ = 63.4 (*c* = 0.84; CHCl$_3$). IR(ATR): ῡ [cm^{-1}] = 3250 (NH), 2955 (CH), 2930 (CH), 2867 (CH), 2360 (C≡C), 2335 (C≡C), 2240, 1458 (CN), 1423, 1363, 1182, 1059, 878, 647.

tert-Butyl-(*S*)-4-(((*S*)-*tert*-butylsulfinyl)amino)hex-5-ynoate (**7s**)

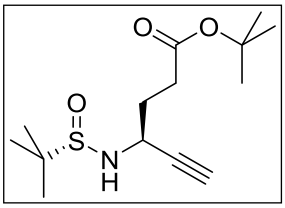

Synthesis: GP-6, starting material was the crude product of **6s**. Reaction scale: 4.04 mmol of imine **5s**. Purification by column chromatography (EtOAc/PE, 1:1).

Colourless crystals, yield: 0.54 g, 1.9 mmol, 47 % (25 % over two steps, referred to imine **5s**). *dr* = 93:7. ^{1}H NMR (500 MHz, Chloroform-*d*) δ = 4.06 (qd, ^3J = 6.9 Hz, ^4J = 2.2 Hz, 1H, C^α**H**), 3.47 (d, ^3J = 7.0 Hz, 1H, N**H**), 2.40 (d, ^4J = 2.4 Hz, 1H, C^{α}C≡C**H**), 2.40- 2.30 (m, 2H, CO$_2$C**H$_2$**), 2.04-1.87 (m, 2H, C^{α}C**H$_2$**), 1.39 (s, 9H, CO$_2$C(C**H$_3$**)$_3$), 1.17 (s, 9H, SC(C**H$_3$**)$_3$). ^{13}C NMR (126 MHz, Chloroform-*d*) δ = 172.1 (**C**O$_2$), 83.0 (C^α**C**≡CH), 80.7 (CO$_2$**C**(CH$_3$)$_3$), 73.7 (C^{α}C≡**C**H), 56.3 (S**C**(CH$_3$)$_3$), 46.8 (**C**^{α}), 31.9 (C^α**C**H$_2$), 31.4 (CO$_2$**C**H$_2$), 28.1 (CO$_2$C(**C**H$_3$)$_3$), 22.6 (SC(**C**H$_3$)$_3$). $C_{14}H_{25}NO_3S$ (287.42 g mol^{-1}), MS(ESI): *m/z* = 288.1635 (288.16279 [M+H]$^+$). $[\alpha]^{22}_{589}$ (*c* = 0.16; CHCl$_3$) = 33.27°. TLC: R$_f$(PE/EtOAc, 1:1) = 0.46.

(*S*)-*N*-((*S*)-6-Hydroxyhex-1-yn-3-yl)-*tert*-butylsulfinamide (**7t**)

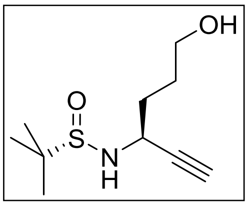

Synthesis: GP-5, starting material was the crude product of **6t**. Reaction scale: 3.67 mmol of imine **5t**. 6 eq of TBAF were used. Reaction was carried out for 2 h at 0 °C. Purification by column chromatography (EtOAc). Compound **7t** has already been described by Bauer, DiBlasi and Tan [45]. X-ray crystal structure analysis has been performed before by Hou et al. [14].

Colourless crystals, yield: 215.1 mg, 989.7 µmol, 27 % (over two steps, referred to aldimine **5t**). *dr* = 93:7. ^{1}H NMR (500 MHz, Chloroform-*d*) δ = 3.99 (m, 1H, C$^{\alpha}$**H**), 3.80 (d, ^{3}J = 6.8 Hz, 1H, C$^{\alpha}$N**H**), 3.56 (t, ^{3}J = 6.2 Hz, 2H, C**H$_2$**OH), 3.37 (s, 1H, O**H**), 2.39 (d, ^{4}J = 2.3 Hz, 1H, C≡C**H**), 1.80 (ddt, ^{2}J = 12.9 Hz, ^{3}J = 8.9 Hz, ^{3}J = 6.4 Hz, 1H, C$^{\alpha}$C**H$_2$**), 1.77-1.68 (m, 1H, C$^{\alpha}$C**H$_2$**), 1.64 (td, ^{2}J = 9.2 Hz, ^{3}J = 8.6 Hz, ^{3}J = 4.1 Hz, 2H, C$^{\alpha}$CH$_2$C**H$_2$**), 1.14 (s, 9H, SC(C**H$_3$**)$_3$). ^{13}C NMR (126 MHz, Chloroform-*d*) δ = 83.6 (C$^{\alpha}$**C**≡CH), 73.2 (C$^{\alpha}$C≡**C**H), 61.6 (**C**H$_2$OH), 56.3 (**C**(CH$_3$)$_3$), 47.3 (**C**$^{\alpha}$), 33.5 (C$^{\alpha}$**C**H$_2$), 28.6 (C$^{\alpha}$CH$_2$**C**H$_2$), 22.6 (SC(**C**H$_3$)$_3$). $C_{10}H_{19}NO_2S$ (217.33 g mol^{-1}). MS(ESI): *m/z* = 218.1213 (218.1209 [M+H]$^{+}$). TLC: R$_f$ (EtOAc) = 0.15. $[\alpha]_{589}^{20}$ = 34.8 (*c* = 1.0; CHCl$_3$). IR(ATR): $\tilde{\nu}$ [cm^{-1}] = 3360 (OH), 3262 (NH), 3132 (CH$_3$), 2948 (CH$_2$), 2898 (CH$_2$), 1027 (SO).

(*S*)-*N*-((*S*)-7-Azidohept-1-yn-3-yl)-*tert*-butylsulfinamide (**7vx**)

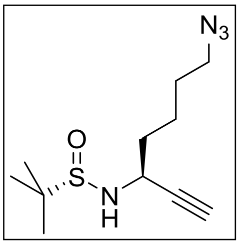

Synthesis: GP-5, starting material was the crude product of **6v**. Reaction scale: 1.16 mmol of imine **5v**. Purification by column chromatography (PE/EtOAc, 2:1). Compound **7vx** has been first described by Ye, He and Zhang [29].

Yellow oil, yield: 170 mg, 0.67 mmol, 58 % (over two steps, referred to imine **5v**). *dr* = 74:26. ^{1}H NMR (500 MHz, Chloroform-*d*) δ = 4.00 (m, 1H, C$^{\alpha}$**H**), 3.42 (d, ^{3}J = 6.7 Hz, 1H, N**H**), 3.26 (t, ^{3}J = 6.6 Hz, 2H, N$_3$C**H$_2$**), 2.41 (d, ^{4}J = 2.4 Hz, 1H, C≡C**H**), 1.72 (ddd, ^{2}J = 15.0 Hz, ^{3}J = 8.1 Hz, ^{3}J = 6.2 Hz, 2H, C$^{\alpha}$C**H$_2$**), 1.63-1.57 (m, 2H, N$_3$CH$_2$C**H$_2$**), 1.56-1.48 (m, 2H, C$^{\alpha}$CH$_2$C**H$_2$**), 1.19 (s, 9H, C(C**H$_3$**)$_3$). ^{13}C NMR (126 MHz, Chloroform-*d*) δ = 83.4 (C$^{\alpha}$**C**≡CH), 73.4 (C$^{\alpha}$C≡**C**H), 56.7 (**C**(CH$_3$)$_3$), 51.2 (N$_3$**C**H$_2$), 47.3 (**C**$^{\alpha}$), 36.2 (C$^{\alpha}$CH$_2$**C**H$_2$), 28.4 (C$^{\alpha}$**C**H$_2$), 22.7 (N$_3$CH$_2$**C**H$_2$), 22.6 (C(**C**H$_3$)$_3$). $C_{11}H_{20}N_4OS$ (256.37 g mol^{-1}), MS(ESI): *m/z* = 279.15 (279.13 [M+Na]$^{+}$). TLC: R$_f$ (EtOAc/PE, 1:2) = 0.25.

(*S*)-*N*-((*S*)-7-Aminohept-1-yn-3-yl)-*tert*-butylsulfinamide (**7vy**)

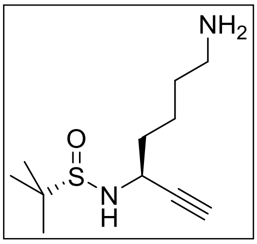

Synthesis: GP-4mod, (compare conversion of **5w** to **7wy**), starting material was the crude product of **6v**. Reaction scale: 5.24 mmol of imine **5v**.

Faintly yellow oil, yield: 0.82 g, 3.559 mmol, 68 %. *dr* = 91:9. ^{1}H NMR (500 MHz, Chloroform-*d*) δ = 3.98 (m, 1H, C$^{\alpha}$**H**), 3.48 (d, ^{3}J = 6.7 Hz, 1H, N**H**), 2.67 (t, ^{3}J = 6.5 Hz, 2H, H_2NC**H₂**), 2.39 (d, ^{4}J = 2.3 Hz, 1H, C≡C**H**), 1.94-1.87 (m, 2H, N$\mathbf{H_2}$), 1.77-1.64 (m, 2H, C$^{\alpha}$C$\mathbf{H_2}$), 1.50-1.40 (m, 4H, C$^{\alpha}$CH$_2$C$\mathbf{H_2}$C$\mathbf{H_2}$), 1.18 (s, 9H, C(C$\mathbf{H_3}$)$_3$). ^{13}C NMR (126 MHz, Chloroform-*d*) δ = 83.8 (C$^{\alpha}$**C**≡CH), 73.1 (C$^{\alpha}$C≡**C**H), 56.2 (**C**(CH$_3$)$_3$), 47.5 (**C**$^{\alpha}$), 41.9 (H$_2$N**C**H$_2$), 36.6 (C$^{\alpha}$**C**H$_2$), 33.0 (C$^{\alpha}$CH$_2$**C**H$_2$), 22.8 (H$_2$NCH$_2$**C**H$_2$), 22.6 (C(**C**H$_3$)$_3$). $C_{11}H_{22}N_2OS$ (230.37 g mol^{-1}). MS(ESI): *m/z* = 253.1352 (253.1345 [M+Na]$^{+}$). $[\alpha]_{589}^{22}$ = 9.3 (*c* = 2.5; CHCl$_3$). TLC: R$_f$ (DCM/MeOH/NEt$_3$, 88:10:2) = 0.18. IR(ATR): ṽ [cm^{-1}] = 3297 (NH), 2927 (NH$_2$), 2863 (CH), 1695, 1521, 1458, 1366, 1253, 1169, 1052, 669.

tert-Butyl ((*S*)-5-(((*S*)-*tert*-Butylsulfinyl)amino)hept-6-yn-1-yl)carbamate (**7vz**)

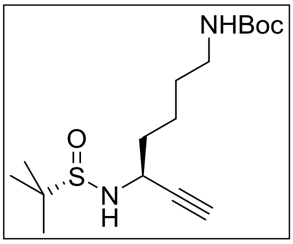

The Boc protection of propargylamine **7vy** was carried out as suggested by Basel and Hassner [52]: Propargylamine **7vy** (820 mg, 3.56 mmol, 1 equiv) was dissolved in a mixture of THF/H$_2$O (1:1, 12 mL). Boc$_2$O (1.55 g, 7.12 mmol, 2 equiv) and NaHCO$_3$ (900 mg, 10.7 mmol, 3 equiv) was added in one portion. The colourless suspension was stirred at rt overnight. When all starting material was consumed (monitored by TLC), imidazole (730 mg, 10.7mmol, 3 equiv) was added and the reaction mixture was stirred for another 4 h at rt. The solution was concentrated under vacuum and then diluted with EtOAc (35 mL). The phases were separated and the organic layer was washed with aqueous HCl (0.1 M, 3 × approximately 10 mL) until the aqueous layer was acidic. The organic layer was washed with brine (5 mL), dried over Na$_2$SO$_4$ and the solvent was evaporated under reduced pressure. The crude product was purified by column chromatography (PE/EtOAc, 2:1). Compound **7vz** has been first described by Ye, He and Zhang [29].

Faintly yellow oil, yield: 531 mg, 1.607 mmol, 45 %. ^{1}H NMR (500 MHz, Chloroform-*d*) δ = 4.56 (s, 1H, N**H**Boc), 4.02 (m, 1H, C$^{\alpha}$**H**), 3.35 (d, ^{3}J = 6.6 Hz, 1H, N**H**), 3.12 (t, ^{3}J = 6.6 Hz, 2H, NHC$\mathbf{H_2}$), 2.41 (d, ^{4}J = 2.2 Hz, 1H, C$^{\alpha}$C≡C**H**), 1.80-1.67 (m, 2H, C$^{\alpha}$CH$_2$C$\mathbf{H_2}$), 1.66-1.59

(m, 2H, C^{α}CH$_2$CH$_2$C**H$_2$**), 1.53-1.47 (m, 2H, C^{α}C**H$_2$**), 1.44 (s, 9H, OC(C**H$_3$**)$_3$), 1.22 (s, 9H, SC(C**H$_3$**)$_3$). ^{1}H NMR (500 MHz, DMSO-*d*$_6$) δ = 6.76 (t, ^3J = 5.8 Hz, 1H, N**H**Boc), 5.62 (d, ^3J = 7.9 Hz, 1H, C^{α}N**H**), 3.82 (q, ^3J = 7.2 Hz, 1H, C^{α}**H**), 3.26 (d, ^4J = 1.6 Hz, 1H, C^{α}C≡C**H**), 2.89 (m, 2H, BocHNC**H$_2$**), 1.63 (m, 2H, C^{α}C**H$_2$**), 1.37 (s, 9H, CO$_2$C(C**H$_3$**)$_3$), 1.29-1.41 (m, 4H, C^{α}CH$_2$C**H$_2$**C**H$_2$**), 1.12 (s, 9H, SC(C**H$_3$**)$_3$). ^{13}C NMR (126 MHz, DMSO) δ = 155.5 (**C**O$_2$), 85.2 (**C**≡CH), 77.3 (CO$_2$**C**(CH$_3$)$_3$), 74.3 (C^{α}C≡**C**H), 55.5 (S**C**(CH$_3$)$_3$), 46.7 (**C**^{α}), 36.4 (BocHN**C**H$_2$), 28.9 (C^{α}**C**H$_2$), 28.3 (SC(**C**H$_3$)$_3$), 22.7 (BocHNCH$_2$**C**H$_2$), 22.6 (CO$_2$C(**C**H$_3$)$_3$), 22.5 (C^{α}CH$_2$**C**H$_2$). $C_{16}H_{30}N_2O_3S$ (330.49 g mol^{-1}). MS(ESI): 331.2045 (331.20499 [M+H]$^+$). $[\alpha]_{589}^{25}$ = 25.0 (*c* = 0.19; CHCl$_3$). TLC: R$_f$(PE/EtOAc, 2:1) = 0.37.

(*S*)-*N*-((*S*)-6-Azidohex-1-yn-3-yl)-*tert*-butylsulfinamide **7wx**

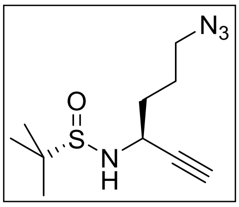

Synthesis: GP-6, starting material was the crude product of **6w**. Reaction scale: 11.53 mmol of imine **5w**. The crude product was separated by column chromatography (elution with EtOAc/PE, 1:1). The ratio of propargylamine **7wx** and triazole **13** was 1:4. Compound **7wx** was observed to be instable. A solution of **7wx** was investigated by ^{1}H NMR spectroscopy. After one week, 66 % of the alkyne had undergone an intramolecular Huisgen reaction and formed compound **14**.

Faintly yellow oil, yield: 0.531 g, 2.19 mmol, 19 % (isolated yield), *dr* = 96:4. ^{1}H NMR (500 MHz, Chloroform-*d*) δ = 4.09 (m, 1H, C^{α}**H**), 3.58 (t, ^3J = 6.3 Hz, 2H, N$_3$C**H$_2$**), 3.35 (d, ^3J = 6.5 Hz, 1H, N**H**), 2.44 (d, ^4J = 2.3 Hz, 1H, C^{α}C≡C**H**), 2.01-1.93 (m, 2H, C^{α}C**H$_2$**), 1.93-1.84 (m, 2H, C^{α}CH$_2$C**H$_2$**), 1.23 (s, 9H, C(C**H$_3$**)$_3$). ^{13}C NMR (126 MHz, Chloroform-*d*) δ = 83.2 (C^{α}**C**≡CH), 73.8 (C^{α}C≡**C**H), 56.4 (**C**(CH$_3$)$_3$), 46.9 (**C**^{α}), 44.5 (H$_2$N**C**H$_2$), 34.1 (C^{α}H**C**H$_2$), 28.6 (H$_2$NCH$_2$**C**H$_2$), 22.7 (C(**C**H$_3$)$_3$). $C_{10}H_{18}N_4OS$ (242.34 g mol^{-1}), MS(ESI): *m/z* = 265.1 (265.34 [M+Na]$^+$). $[\alpha]_{589}^{21.2}$ = 45.0 (*c* = 0.10; CHCl$_3$). IR(ATR): $\tilde{\nu}$ [cm^{-1}] = 3287-3202 (NH), 2955 (CH$_2$), 2369-2331 (N$_3$), 1071 (SO).

(*S*)-*N*-((*S*)-6-Aminohex-1-yn-3-yl)-2-methylpropane-2-sulfinamide (**7wy**)

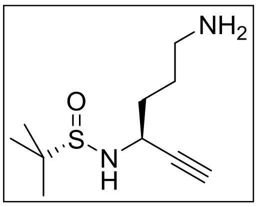

Synthesis: *n*-Butyllithium (1.6 M in *n*-hexane, 35.2 mL, 56.3 mmol, 1.2 equiv) was added dropwise to a solution of TMS-acetylene (7.9 mL,

56.3 mmol, 1.2 equiv) in toluene (90 mL) at −78 °C. The clear solution was stirred for 2 h, before a solution of imine **5w** (10.14 g, 46.9 mmol, 1 equiv) in toluene (90 mL) was dropped very slowly into the mixture. After full conversion (3-4 h, monitored by analytical HPLC), a solution of PPh_3 (49.2 g, 187 mmol, 4.0 equiv) in THF (150 mL) was added to the reaction mixture and the cooling bath was removed, so that the solution could warm up to rt. When no further evolution of gas could be observed (2 h later), water (150 mL) was added to the orange reaction mixture and stirring was continued vigorously for 10 h. Then, the phases were separated. The organic phase was washed with a solution of $NaHCO_3$ (5 %, 50 mL). The combined aqueous layers were extracted with DCM (5 × 40 mL). The combined organic layers were dried over Na_2SO_4 and the solvent was evaporated under reduced pressure. The crude product was purified by column chromatography (DCM/MeOH/NEt_3, 88:10:2) to yield compound **7wy** in pure form.

Yellow oil, yield: 7.330 g, 33.88 mmol, 86 %, *dr* = 80:20. ^{1}H NMR (500 MHz, Chloroform-*d*) δ = 3.99 (dd, ^3J = 5.9 Hz, ^3J = 5.9 Hz, 1H, C^α**H**), 2.70 (t, ^3J = 6.8 Hz, 2H, H_2NC**H₂**), 2.57 (s, 2H, N**H₂**), 2.40 (d, ^4J = 2.0 Hz, 1H, C^{α}C≡C**H**), 1.77 (m, 1H, C^{α}C**H₂**), 1.69 (m, 1H, C^{α}C**H₂**), 1.60 (m, 2H, C^αCH_2C**H₂**), 1.16 (s, 9H, SC(C**H₃**)₃). ^{13}C NMR (126 MHz, Chloroform-*d*) δ = 83.7 (C^α**C**≡CH), 73.2 (C^{α}C≡**C**H), 56.3 (**C**(CH_3)₃), 47.3 (**C**^{α}), 41.3 (NH_2**C**H_2), 34.3 (C^α**C**H_2), 28.8 (C^αCH_2**C**H_2), 22.6 (C(**C**H_3)₃). $C_{10}H_{20}N_2OS$ (216.34 g mol^{-1}). MS(ESI): *m/z* = 217.1357 (217.1369 [M+H]$^+$). $[\alpha]^{22}_{589}$ = 26.2 (*c* = 0.51; $CHCl_3$). TLC: R_f (DCM/MeOH/NEt_3, 88:10:2) = 0.25.

tert-Butyl ((*S*)-4-(((*S*)-*tert*-butylsulfinyl)amino)hex-5-yn-1-yl)carbamate **7wz**

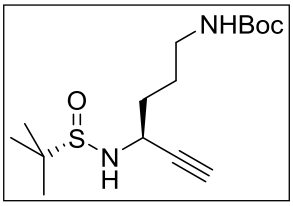

Synthesis: See preparation of **7vz**, starting material was the crude product of **7wy**. Reaction scale: 19.83 mmol of imine **5w**. Purification by column chromatography (PE/EtOAc, 2:1).

Colourless highly viscous oil, yield: 3.9515 g, 12.487 mmol, 63 %, dr = 90:10. ^{1}H NMR (500 MHz, Chloroform-*d*) δ = 4.57 (s, 1H, N**H**Boc), 4.06 (qd, ^3J = 6.5 Hz, ^4J = 2.3 Hz, 1H, C^α**H**), 3.37 (d, ^3J = 6.6 Hz, 1H, N**H**), 3.16 (q, ^3J = 6.8 Hz, 2H, BocHNC**H₂**), 2.42 (d, ^4J = 2.3 Hz, 1H, C^{α}C≡C**H**), 1.82- 1.70 (m, 2H, C^{α}C**H₂**), 1.69- 1.62 (m, 2H, C^αCH_2C**H₂**), 1.44 (s, 9H, CO_2C(C**H₃**)₃), 1.22 (s, 9H, SC(C**H₃**)₃). ^{13}C NMR (126 MHz, Chloroform-*d*) δ = 156.1 (**C**O_2), 83.5 (C^α**C**≡CH), 79.4 (CO_2**C**(CH_3)₃), 73.5 (C^{α}C≡**C**H), 56.4 (S**C**(CH_3)₃), 47.3 (**C**^{α}), 34.1 (BocHN**C**H_2), 28.6 (CO_2C(**C**H_3)₃), 26.3 (C^α**C**H_2), 22.7

(SC(**C**H_3)$_3$), 22.6 (C$^{\alpha}$CH$_2$**C**H$_2$). $C_{15}H_{28}N_2O_3S$ (316.46 g mol^{-1}), MS(ESI): *m/z* = 339.1709 (339.1713 [M+Na]$^+$), $[\alpha]^{25}_{589}$ = 42.3 (*c* = 0.24; $CHCl_3$). TLC: R_f (EtOAc) = 0.56. IR(ATR): ṽ [cm^{-1}] = 3297 (NH), 2971 (CH), 2924 (CH), 2867 (CH), 1689 (CO), 1524 (SO), 1363, 1252, 1169, 1055.

DiBoc protected arginine analogue Propargylamine **7x**

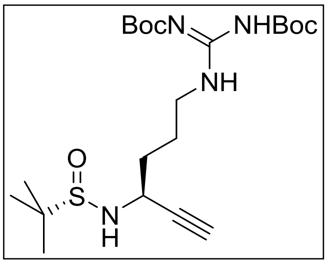

Propargylamine **7wy** (3.378 g, 15.61 mmol, 1 equiv) and DiBocisothiourea (4.5 g, 15.5 mmol, 1.0 equiv) were dissolved under argon atmosphere in dry DCM (20 mL). The reaction mixture was stirred for 3 d at rt. Then the solvent was evaporated under reduced pressure. The residue was diluted with water (30 mL) and the solution was extracted with DCM (5 × 30 mL). The combined organic layers were washed with brine (15 mL) and dried over Na_2SO_4 before the solvent was evaporated in vacuum. The crude product was purified by column chromatography (DCM/MeOH, 10:1).

Faintly yellow, highly viscous oil, yield: 5.625 g, 12.27 mmol, 79 %, dr = 93:7, ^{1}H NMR (500 MHz, Chloroform-*d*) δ = 11.48 (s, 1H, N**H**Boc), 8.37 (s, 1H, N**H**CN$_2$), 4.11-4.04 (m, 1H, C$^{\alpha}$**H**), 3.47 (t, ^3J = 6.2 Hz, 2H, N$_2$CNC**H$_2$**), 3.40 (d, ^3J = 6.5 Hz, 1H, C$^{\alpha}$N**H**), 2.42 (d, ^4J = 2.3 Hz, 1H, C≡C**H**), 1.81-1.72 (m, 4H, C$^{\alpha}$C**H$_2$**C**H$_2$**), 1.49 (s, 9H, C=NCO$_2$C(C**H$_3$**)$_3$), 1.49 (s, 9H, CHNCO$_2$C(C**H$_3$**)$_3$), 1.21 (s, 9H, SC(C**H$_3$**)$_3$). ^{13}C NMR (126 MHz, Chloroform-*d*) δ = 163.7 (N**C**N$_2$), 156.3 (C=N**C**O$_2$^{t}Bu), 153.4 (CHN**C**O$_2$^{t}Bu), 83.4 (C$^{\alpha}$**C**≡CH), 83.3 (C=NCO$_2$**C**(CH$_3$)$_3$), 79.5 (CHNCO$_2$**C**(CH$_3$)$_3$), 73.6 (C$^{\alpha}$C≡**C**H), 56.4 (S**C**(CH$_3$)$_3$), 47.2 (**C**$^{\alpha}$C≡CH), 40.4 (**C**H$_2$NHCN$_2$), 34.0 (C$^{\alpha}$**C**H$_2$), 28.4 (C=NCO$_2$C(**C**H$_3$)$_3$), 28.2 (CHNCO$_2$C(**C**H$_3$)$_3$), 25.2 (C$^{\alpha}$CH$_2$**C**H$_2$), 22.7 (SC(**C**H$_3$)$_3$). $C_{21}H_{38}N_4O_5S$ (458.62 g mol^{-1}), MS(ESI): *m/z* = 459.2719 (459.2636 [M+H]$^+$), 481.2453 (481.2455 [M+Na]$^+$).

Hydrolysis of imine 5 forms hemiaminal 8

(*S*)-*N*-(2,2,2-Trifluoro-1-hydroxyethyl)-*tert*-butylsulfinamide (**8k**)

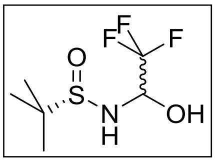

A solution of crude imine **5k** in DCM was diluted with water. The organic layer was evaporated under reduced pressure. Then, the aqueous layer was extracted with DCM (3 × 30 mL). The combined organic layers were dried

over Na_2SO_4 and the solvent was evaporated under vacuum to yield a colourless solid. Recrystallization from EtOAc yielded hemiaminal **8k** in high purity.

Colourless crystals, yield: 1.773 g, 8.810 mmol, 34 % (referred to Ellman's sulfinamide **(*S*)-1**). ^{1}H NMR (300 MHz, DMSO-d_6) δ = 7.41 (d, $^{3}J_{HH}$ = 5.2 Hz, 1H, O**H**), 6.59 (d, $^{3}J_{HH}$ = 8.2 Hz, 1H, N**H**), 4.98 (m, 1H, C$^{\alpha}$**H**), 1.10 (s, 9H, C(C**H**$_3$)$_3$). ^{19}F NMR (282 MHz, DMSO-d_6) δ = -80.0 (d, $^{3}J_{FH}$ = 5.6 Hz, C**F**$_3$). ^{13}C NMR (126 MHz, Chloroform-*d*) δ = 122.5 (q, $^{1}J_{CF}$ = 281.8 Hz, **C**F$_3$), 80.3 (q, $^{2}J_{CF}$ = 34.9 Hz, **C**$^{\alpha}$H), 57.3 (**C**(CH$_3$)$_3$), 22.5 (**C**H$_3$). $C_6H_{12}F_3NO_2S$ (219.22 g mol^{-1}).

(*S*)-*N*-(2,2,2-Trichloro-1-hydroxyethyl)-*tert*-butylsulfinamide (**8l**)

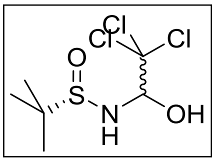

Ellman's chiral sulfinamide **(*S*)-1** (1.00 g, 8.25 mmol, 1 equiv) was added in one portion to freshly dried and distilled chloral (1.21 g, 9.38 mmol, 1 equiv) and the mixture was dissolved in DCM (20 mL). After stirring of the solution for 3 d, conversion of the starting material was complete (monitored by TLC). Water (20 mL) was added and the emulsion was concentrated up under reduced pressure. The residue was stirred for 1.5 h at rt, before it was extracted with DCM (3 × 30 mL). TLC showed complete consumption of the formed intermediate. The combined organic layers were washed with brine (10 mL), dried over $MgSO_4$ and the solvent was evaporated under vacuum. The crude product was obtained in form of a colourless, crystalline solid. Hemiaminal **8l** was isolated by column chromatography.

Colourless crystals, yield: 1.0847 g, 4.0386 mmol, 49 % (referred to Ellman's sulfinamide **(*S*)-1**), ^{1}H NMR (500 MHz, DMSO-d_6) δ = 7.82 (d, ^{3}J = 6.7 Hz, 1H, O**H**), 5.83 (d, ^{3}J = 8.7 Hz, 1H, N**H**), 5.05 (dd, ^{3}J = 8.7 Hz, ^{3}J = 6.6 Hz, 1H, C$^{\alpha}$**H**), 1.17 (s, 9H, SC(C**H**$_3$)$_3$). ^{13}C NMR (126 MHz, DMSO) δ = 103.6 (Cl$_3$**C**), 89.4 (**C**$^{\alpha}$), 56.6 (**C**(CH$_3$)$_3$), 22.8 (C(**C**H$_3$)$_3$). $C_6H_{12}Cl_3NO_2S$ (268.58 g mol^{-1}). TLC: R_f(EtOAc/PE, 1:4) = 0.14.

Side-product 9k of the conversion of 5k with GP-3

(*S*)-*N*-((*R*)-2,2,2-trifluoro-1-isopropoxyethyl)-*tert*-butylsulfinamide (**9k**)

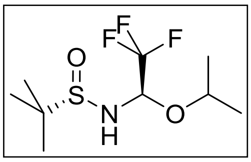

Synthesis: GP-3: Ti(OiPr)$_4$ (2.61 mL, 8.816 mmol, 1 equiv) was added to a solution of crude **5k** (8.816 mmol) and the mixture was dropped

directly into a solution of trimethylsilylethynyl lithium (14.1 mmol, 1.6 equiv) in THF (50 mL) at −78 °C. The reaction mixture was stirred at −78 °C for 2 h, and then warmed up to rt. The solution was diluted with a saturated aqueous solution of NH_4Cl (40 mL) and the colourless precipitate was filtered through a pad of celite. The phases were separated and the aqueous layer was extracted with DCM (2 × 30 mL). The combined organic layers were washed with brine (10 mL) and dried over Na_2SO_4 before the solvent was evaporated under reduced pressure. The crude product was recrystallized from EtOAc/Et_2O, 1:2 to yield aminal **9k** in pure form.

Colourless crystals, yield: 727.9 mg, 2.786 mmol, 32 % (referred to Ellman's sulfinamide **(*S*)-1**). ^{1}H NMR (300 MHz, Chloroform-*d*) δ = 4.72 (dq, $^3J_{HH}$ = 9.6 Hz, $^3J_{HF}$ = 4.7 Hz, 1H, C^{α}**H**), 4.10 (septett, $^3J_{HH}$ = 6.1 Hz, 1H, $(CH_3)_2C$**H**), 3.90 (d, $^3J_{HH}$ = 9.9 Hz, 1H, N**H**), 1.19 (d, $^3J_{HH}$ = 6.3 Hz, 3H, $(CH_3)CHC$**H_3**), 1.17 (s, 9H, $C(C$**H_3**$)_3$), 1.14 (d, $^3J_{HH}$ = 6.1 Hz, 3H, $(C$**H_3**$)CHCH_3$). ^{13}C NMR (126 MHz, Chloroform-*d*) δ = 122.3 (q, $^1J_{CF}$ = 282.4 Hz, **C**F_3), 83.5 (q, $^2J_{CF}$ = 33.7 Hz), 70.7 (O**C**H$(CH_3)_2$), 56.9 (**C**$(CH_3)_3$), 22.8 (C(**C**$H_3)_3$), 22.3 ($(CH_3)CH$**C**H_3), 20.5 ($(CH_3)CH$**C**H_3). ^{19}F NMR (282 MHz, Chloroform-*d*) δ = -80.8 (d, $^3J_{FH}$ = 5.0 Hz, C**F_3**). $C_9H_{18}F_3NO_2S$ (261.30 g mol^{-1}). MS(ESI): *m/z* = 262.1080 (262.10831 $[M+H]^+$). TLC: R_f (PE/EtOAc, 2:1) = 0.42.

Side-product 10k of the conversion of 5k with GP-4

(*S*)-*N*-((*R*)-1,1,1-Trifluoropropane-2-yl)-*tert*-butylsulfinamide (**10k**)

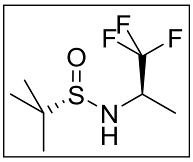

Synthesis: GP-4. A solution of $AlMe_3$ (2.0 M in *n*-hexane, 9.8 mL, 19.6 mmol, 1 equiv) was added to a solution of crude **5k** (19.68 mmol) and the mixture was dropped directly into a solution of trimethylsilylethynyl lithium (31.5 mmol, 1.6 equiv) in toluene (70 mL) at −78 °C. The reaction mixture was stirred at −78 °C for 2 h, and then warmed up to rt. The solution was diluted with a saturated aqueous solution of NH_4Cl (40 mL) and the colourless slurry was filtered through a pad of celite. The phases were separated and the aqueous layer was extracted with DCM (2 × 30 mL). The combined organic layers were washed with brine (10 mL) and dried over Na_2SO_4 before the solvent was evaporated under reduced pressure. The crude product was recrystallised from EtOAc/*n*-hexane, 1:5 to yield aminal **10k** in pure form. Racemic compound **10k** has been first prepared by Packer, Melassis, Wells, Light and Linclau, by adding Ellman's sulfinamide **(*R*)-1** to 1,1,1-trifluoropropan-2-one [53].

Colourless crystals, yield: 2.262 g, 10.41 mmol, 53 % (referred to Ellman's sulfinamide **(*S*)-1**). ^{1}H NMR (300 MHz, Chloroform-*d*) δ = 3.84 (m, 1H, C$^{\alpha}$**H**), 3.13 (d, $^{3}J_{HH}$ = 7.4 Hz, 1H, N**H**), 1.46 (d, $^{3}J_{HH}$ = 6.9 Hz, 3H, C$^{\alpha}$C**H$_3$**), 1.22 (s, 9H, C(C**H$_3$**)$_3$). ^{13}C NMR (75 MHz, Chloroform-*d*) δ = 125.4 (q, $^{1}J_{CF}$ = 280.1 Hz, **C**F$_3$), 56.8 (**C**(CH$_3$)$_3$), 54.6 (q, $^{2}J_{CF}$ = 31.8 Hz, C$^{\alpha}$H), 22.5 (C(C**H$_3$**)$_3$), 16.7 (q, $^{4}J_{CF}$ = 1.9 Hz, C$^{\alpha}$**C**H$_3$). ^{19}F NMR (282 MHz, Chloroform-*d*) δ = -78.3 (d, $^{3}J_{FH}$ = 7.0 Hz, C**F$_3$**). $C_7H_{14}F_3NOS$ (217.25 g mol^{-1}). MS(ESI): *m/z* = 218.0813 (218.0821 [M+H]$^{+}$). $[\alpha]_{589}^{22}$ = -17.2 (*c* = 0.33; MeOH).

Furthermore, TMS-protected alkyne **6k** could be isolated in a low amount of 1.384 g, 0.414 mmol, 22 %.

Sonogashira cross-coupling products as peptidomimetics 11

Methyl 4-((*S*)-3-(((*S*)-*tert*-butylsulfinylamido)-3-phenylprop-1-yn-1-yl)benzoate (**11i**)

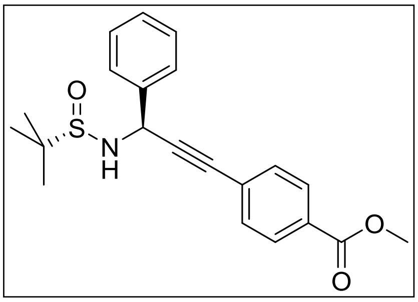

Synthesis: GP-9, reaction scale was 407 µmol of propargylamine **7i**. The crude product was purified by column chromatography (PE/EtOAc, 1:1) and then recrystallized from Et_2O.

Colourless crystalline solid, yield: 83 mg, 225 µmol, 54 %. ^{1}H NMR (500 MHz, Chloroform-*d*) δ = 7.98 (d, ^{3}J = 8.4 Hz, 2H, ar-2-**H**, ar-6-**H**), 7.56 (d, ^{3}J = 6.9 Hz, 1H, Ph-2-**H**, Ph-6-**H**), 7.53 (d, ^{3}J = 8.4 Hz, 2H, ar-3-**H**, ar-5-**H**), 7.41 (t, ^{3}J = 7.3 Hz, 2H, Ph-3-**H**, Ph-5-**H**), 7.37 (m, 1H, Ph-4-**H**), 5.47 (m, 1H, C$^{\alpha}$**H**), 3.92 (s, 3H, CO$_2$C**H$_3$**), 1.27 (s, 9H, SC(C**H$_3$**)$_3$). ^{13}C NMR (126 MHz, Chloroform-*d*) δ = 166.6 (**C**O$_2$CH$_3$), 138.3 (Ph-**C**-1), 131.9 (ar-**C**-3, ar-**C**-5), 130.1 (ar-**C**-1), 129.6 (ar-**C**-2, ar-**C**-6), 129.0 (Ph-**C**-3, Ph-**C**-5), 128.8 (Ph-**C**-1), 127.8 (Ph-**C**-2, Ph-**C**-6), 127.0 (Ph-**C**-4), 90.2 (C$^{\alpha}$**C**≡Car), 86.3 (C$^{\alpha}$C≡**C**ar), 57.3 (S**C**(CH$_3$)$_3$), 52.4 (CO$_2$**C**H$_3$), 52.3 (**C**$^{\alpha}$), 22.7 (SC(**C**H$_3$)$_3$). $C_{21}H_{23}NO_3S$ (369.48 g mol^{-1}), MS(ESI): *m/z* = 392.975 (392.129 [M+Na]$^{+}$). IR(ATR): $\tilde{\nu}$ [cm^{-1}] = 2977 (CH), 2948 (CH), 2920 (CH), 2328 (C≡C), 2353 (C-C), 1720 (C=O), 1543 (ar, C=C), 1280 (C-N). TLC: R_f (EtOAc/PE, 1:2) = 0.44. $[\alpha]_{589}^{19}$ = 10.4 (*c* = 0.72; CHCl$_3$).

Methyl 3-((*R*)-3-(((*S*)-*tert*-butylsulfinyl)amido)-4,4,4-trifluorobut-1-yn-1-yl)benzoate (**11k**)

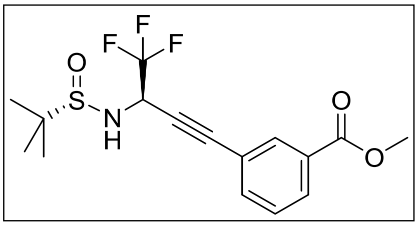

Synthesis: GP-9, reaction scale: 582 µmol of propargylamine **7k**. The crude product was purified by column chromatography (PE/EtOAc, 1:1).

Faintly yellow oil, yield: 94.7 mg, 262 µmol, 45 %. ^{1}H NMR (300 MHz, Chloroform-*d*) δ = 8.10 (t, ^4J = 1.7 Hz, 1H, ar-2-**H**), 8.01 (dt, ^3J = 7.9 Hz, ^4J = 1.5 Hz, 1H, ar-6-**H**), 7.62 (dt, ^3J = 7.7 Hz, ^4J = 1.5 Hz, 1H, ar-4-**H**), 7.39 (t, ^3J = 7.8 Hz, 1H, ar-5-**H**), 4.77 (dq, ^3J = 7.6 Hz, ^3J = 6.3 Hz, 1H, C^α**H**), 4.04 (d, ^3J = 7.6 Hz, 1H, N**H**), 3.90 (s, 3H, $CO_2C\mathbf{H_3}$), 1.25 (s, 9H, $SC(C\mathbf{H_3})_3$). ^{19}F NMR (282 MHz, Chloroform-*d*) δ = -76.3 (d, $^3J_{FH}$ = 6.3 Hz, $C\mathbf{F_3}$). ^{13}C NMR (75 MHz, Chloroform-*d*) δ = 166.2 ($\mathbf{C}O_2(CH_3)$), 136.2 (ar-**C**-4), 133.2 (ar-**C**-2), 130.6 (ar-**C**-1), 130.5 (ar-**C**-6), 128.7 (ar-**C**-5), 121.5 (ar-**C**-3), 121.3 (q, $^1J_{CF}$ = 280.7 Hz, $\mathbf{C}F_3$), 86.7 (C^{α}C≡**C**ar), 80.6 (q, $^3J_{CF}$ = 2.2 Hz, C^α**C**≡Car), 57.6 ($S\mathbf{C}(CH_3)_3$), 52.4 ($CO_2\mathbf{C}H_3$), 51.5 (q, $^2J_{CF}$ = 35.1 Hz, **C**^{α}H), 22.5 ($SC(\mathbf{C}H_3)_3$). $C_{16}H_{18}F_3NO_3S$ (361.38 g mol^{-1}). MS(ESI): *m/z* = 362.1022 (362.1032 $[M+H]^+$). TLC: R_f (EtOAc/PE, 1:1) = 0.63.

Rearrangementproducts: α,β-unsaturated imines 12

Methyl 4-((1*E*,3*Z*)-3-(((*S*)-*tert*-butylsulfinyl)imido)-3-phenylprop-1-en-1-yl)benzoate (**12i**)

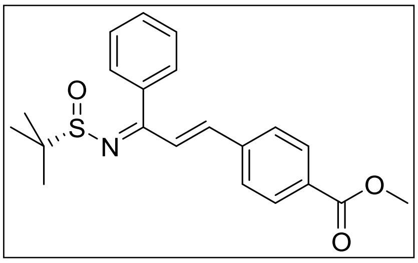

Piperidine (1 mL) was added to a solution of **11i** (151.5 mg, 409 µmol) in THF (3 mL) at 0 °C. The reaction mixture instantly turned brightly yellow. After 30 min, Et_2O (20 mL) was added and the solution was washed with a $KHSO_4$ solution (5 %, 2 × approximately 20 mL) and brine (5 mL). The organic layer was dried over Na_2SO_4 and the solvent was evaporated under reduced pressure. The crude product was purified by column chromatography (PE/EtOAc, 10:1) and recrystallization from Et_2O.

Brightly yellow crystalline solid, yield: 81.8 mg, 221 µmol, 98 % (isolated yield). ^{1}H NMR (600 MHz, Chloroform-*d*) δ = 8.19 (d, ^3J = 16.3 Hz, 1H, C^{α}HC=C**H**ar), 8.01 (d, ^3J = 8.3 Hz, 2H, ar-2-**H**, ar-6-**H**), 7.64 (d, ^3J = 8.2 Hz, 2H, Ph-2-**H**, Ph-6-**H**), 7.56 (d, ^3J = 7.9 Hz, 2H, ar-3-**H**, ar-5-**H**), 7.51 (t, ^3J = 7.4 Hz, 1H, Ph-4-**H**), 7.45 (t, ^3J = 7.5 Hz, 2H, Ph-3-**H**, Ph-5-**H**), 6.90 (d, ^3J = 16.3 Hz, 1H, C^α**H**C=CHar), 3.91 (s, 3H, $CO_2C\mathbf{H_3}$), 1.35 (s, 9H, $SC(C\mathbf{H_3})_3$).

^{13}C NMR (151 MHz, Chloroform-*d*) δ = 174.6 (PhC$^{\alpha}$=N), 166.6 (**C**O$_2$(CH$_3$)), 142.2 (C$^{\alpha}$H**C**=CHar), 139.6 (ar-**C**-1), 138.6 (Ph-**C**-1), 131.1 (ar-**C**-4), 131.0 (Ph-**C**-4), 130.2 (ar-**C**-2, ar-**C**-6), 129.3 (Ph-**C**-2, Ph-**C**-6), 128.5 (Ph-**C**-3, Ph-**C**-5), 128.0 (ar-**C**-3, ar-**C**-5), 124.7 (C$^{\alpha}$HC=**C**Har), 58.8 (**C**(CH$_3$)$_3$), 52.4 (CO$_2$**C**H$_3$), 23.0 (SC(**C**H$_3$)$_3$). $C_{21}H_{23}NO_3S$ (369.48 g mol^{-1}). MS(EI): *m/z* = 370.1 (370.15 [M+H]$^+$), 392.1 (392.13 [M+Na]$^+$). TLC: R$_f$ (EtOAc/PE, 1:1) = 0.6. $[\alpha]_{589}^{20}$ = 246.8 (*c* = 1.46; CHCl$_3$). IR(ATR): ṽ [cm^{-1}] = 2977 (CH), 2948 (CH), 2920 (CH), 2328 (C=C), 2353 (C-C), 1720 (CO), 1543 (ar, C=C), 1280 (C-N).

Methyl (*E*)-3-(4,4,4-trifluoro-3-iminobut-1-en-1-yl)benzoate (**12k**)

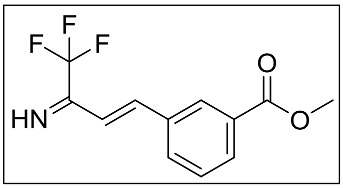

Propargylamine **11k** was dissolved in MeOH and an aqueous solution of LiOH (3 equiv, 1 M) was added dropwise at 0 °C. After 5 min, the solution turned brightly yellow, while the reaction progress was monitored by TLC. After complete conversion, the reaction mixture was acidified with HCl and extracted with Et$_2$O (3 × 30 mL). The combined organic layers were dried over NaSO$_4$, the solvent was evaporated and the crude product directly investigated by NMR spectroscopy.

Crude product, brightly yellow oil, yield: 16.8 mg, 65.4 µmol, 84 %. ^{1}H NMR (500 MHz, Chloroform-*d*) δ = 8.32 (t, ^4J = 1.8 Hz, 1H, ar-2-**H**), 8.16 (dt, ^3J = 7.8 Hz, ^4J = 1.4 Hz, 1H, ar-6-**H**), 7.99 (d, ^3J = 16.0 Hz, 1H, C$^{\alpha}$C=C**H**ar), 7.81 (dt, ^3J = 7.7 Hz, ^4J = 1.5 Hz, 1H, ar-4-**H**), 7.55 (t, ^3J = 7.8 Hz, 1H, ar-5-**H**), 7.09 (dd, ^3J = 16.0 Hz, ^4J = 0.9 Hz, 1H, C$^{\alpha}$C**H**=Car), 3.97 (s, 3H, CO$_2$C**H$_3$**). ^{19}F NMR (470 MHz, Chloroform-*d*) δ = -77.7 (s, C**F$_3$**). ^{13}C NMR (126 MHz, Chloroform-*d*) δ = 180.0 (q, $^2J_{CF}$ = 35.7 Hz, **C**=N), 162.1 (**C**O$_2$(CH$_3$)), 148.6 (C$^{\alpha}$C=**C**ar), 134.2 (ar-**C**-4), 134.0 (ar-**C**-1), 133.6 (ar-**C**-6), 131.2 (ar-**C**-3), 130.7 (ar-**C**-2), 129.8 (ar-**C**-5), 118.2 (C$^{\alpha}$**C**=Car), 117.7 (q, $^1J_{CF}$ = 290.8 Hz, **C**F$_3$). $C_{12}H_{10}F_3NO_2$ (257.21 g mol^{-1}). MS(ESI): *m/z* = 362.1022 (362.1032 [M+H]$^+$). TLC: R$_f$ (EtOAc/PE, 1:2) = 0.74. UV/vis: ε = 12.60 L mol^{-1}cm^{-1} (304 nm).

Intramolecular Huisgen reaction of 6w gives triazole 13w

(*S*)-*N*-((*S*)-3-(Trimethylsilyl)-4,5,6,7-tetrahydro-[1,2,3]triazolo[1,5-a]pyridine-4-yl)-*tert*-butylsulfinamide (**13w**)

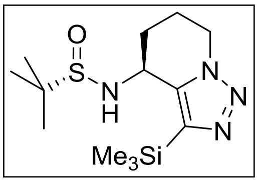

Synthesis: GP-4, reaction scale was 8.96 mmol of imine **5w**. The crude product was separated by column chromatography (elution with EtOAc). The ratio of propargylamine **6w** and triazole **13w** was 1:4. Triazole **13w** was recrystallized from toluene.

Colourless crystals, yield: 1.578 g, 5.017 mmol, 56 %, dr = 100:0. ^{1}H NMR (500 MHz, Chloroform-*d*) δ = 4.88 (m, 1H, C^{α}**H**), 4.66 (dd, ^2J = 13.3 Hz, ^3J = 5.4 Hz, 1H, N_3C**H_2**), 4.13 (dt, ^2J = 13.5 Hz, ^3J = 5.2 Hz, 1H, N_3C**H_2**), 3.07 (s, 1H, N**H**), 2.38-2.23 (m, 2H, $C^{\alpha}C$**H_2**C**H_2**), 1.99 (m, 1H, $C^{\alpha}C$**H_2**), 1.82 (m, 1H, $C^{\alpha}CH_2C$**H_2**), 1.19 (s, 9H, SC(C**H_3**$)_3$), 0.39 (s, 9H, Si(C**H_3**$)_3$). ^{13}C NMR (126 MHz, Chloroform-*d*) δ = 143.5 (TMSC=**C**), 139.3 (TMS**C**=C), 55.7 (S**C**$(CH_3)_3$), 46.3 (**C^{α}**), 43.6 (**C**H_2N_3), 26.1 (C^{α}**C**H_2), 22.5 (SC(**C**$H_3)_3$), 16.8 ($C^{\alpha}CH_2$**C**H_2), -0.8 (Si(**C**$H_3)_3$). $C_{13}H_{26}N_4OSSi$ (314.52 g mol^{-1}). MS(ESI): *m/z* = 315.1668 (315.16693 $[M+H]^+$). Smp.: 167 °C. IR(ATR): ῦ [cm^{-1}] = 3307 (NH), 3234 (CH), 2952 (CH), 2898 (CH), 2860 (CH), 2091, 1477, 1439 (SO), 1363, 1249, 1185, 1154, 1071 (SC), 970, 843, 758. TLC: R_f(EtOAc) = 0.35. EA, C: 49.713%, H: 8.388%, N: 17.643%, S: 9.832%.

Intramolecular Huisgen reaction of 7wx gives triazole 14w

(*S*)-*N*-((*S*)-4,5,6,7-Tetrahydro-[1,2,3]triazolo[1,5-*a*]pyridine-4-yl)-*tert*-butylsulfinamide (**14w**)

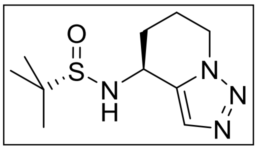

A solution of **7wx** in $CDCl_3$ was monitored for 7 d via NMR spectroscopy at rt. The conversion of the starting material was 66 %.

^{1}H NMR (600 MHz, Chloroform-*d*) δ = 7.86 (s, 1H, C=C**H**), 4.66 (dd, ^3J = 8.1 Hz, ^3J = 5.4 Hz, 1H, C^{α}**H**), 4.40 (dt, ^2J = 13.1 Hz, ^3J = 5.2 Hz, 2H, N_3C**H_2**), 4.30 (ddd, ^2J = 13.5 Hz, ^3J = 8.6 Hz, ^3J = 5.0 Hz, 1H, N_3C**H_2**), 3.59 (d, ^3J = 5.8 Hz, N**H**), 2.29 (ddtd, ^2J = 12.2 Hz, ^3J = 6.8 Hz, ^3J = 4.9 Hz, ^4J = 1.8 Hz, 1H, $C^{\alpha}C$**H_2**), 2.23 (tdd, ^3J = 7.6 Hz, ^3J = 5.6 Hz, ^3J = 3.1 Hz, 1H, N_3CH_2C**H_2**), 2.06 (ddtd, ^2J = 11.6 Hz, ^3J = 8.5 Hz, ^3J = 6.5 Hz, ^3J = 6.0 Hz, ^4J = 2.9 Hz, 1H, N_3CH_2C**H_2**), 1.98 (dddd, ^2J = 13.3 Hz, ^3J = 10.6 Hz, ^3J = 7.9 Hz, ^4J = 2.1 Hz, 1H, C^{α}**H_2**), 1.25 (s, 9H, C(C**H_3**$)_3$). ^{13}C NMR (126 MHz, Chloroform-*d*) δ = 135.8 (**C**=CH), 132.5 (C=**C**H), 56.1 (**C**$(CH_3)_3$), 48.5 (**C^{α}**), 45.7 (N_3**C**H_2), 29.3 (C^{α}**C**H_2), 22.6 (C(**C**$H_3)_3$), 20.3 (N_3CH_2**C**H_2). $C_{10}H_{18}N_4OS$ (242.34 g mol^{-1}). MS(ESI): *m/z* = 243.1268 (243.12741 $[M+H]^+$).

References

1. Dolomanov, O. V.; Bourhis, L. J.; Gildea, R. J.; Howard, J. a. K.; Puschmann, H. *J. Appl. Crystallogr.* **2009**, *42*, 339-341.
2. Sheldrick, G. M. *Acta Crystallogr. A* **2008**, *64*, 112-122.
3. Liu, G.; Cogan, D. A.; Owens, T. D.; Tang, T. P.; Ellman, J. A. *J. Org. Chem.* **1999**, *64*, 1278-1284.
4. Datta, G. K.; Ellman, J. A. *J. Org. Chem.* **2010**, *75*, 6283-6285.
5. Collados, J. F.; Toledano, E.; Guijarro, D.; Yus, M. *J. Org. Chem.* **2012**, *77*, 5744-5750.
6. Liu, G.; Cogan, D. A.; Ellman, J. A. *J. Am. Chem. Soc.* **1997**, *119*, 9913-9914.
7. Robak, M. T.; Herbage, M. A.; Ellman, J. A. *Chem. Rev.* **2010**, *110*, 3600-3740.
8. Levin, V. V.; Dilman, A. D.; Belyakov, P. A.; Struchkova, M. I.; Tartakovsky, V. A. *Eur. J. Org. Chem.* **2008**, *31*, 5226-5230.
9. Cogan, D. A.; Liu, G.; Ellman, J. *Tetrahedron* **1999**, *55*, 8883-8904.
10. Patterson, A. W.; Ellman, J. A. *J. Org. Chem.* **2006**, *71*, 7110-7112.
11. Guijarro, D.; Pablo, Ó.; Yus, M. *Tetrahedron Lett.* **2009**, *50*, 5386-5388.
12. Moura-Letts, G.; DiBlasi, C. M.; Bauer, R. A.; Tan, D. S. *Proc. Natl. Acad. Sci. U. S. A.* **2011**, *108*, 6745-6750.
13. Chen, B.-L.; Wang, B.; Lin, G.-Q. *J. Org. Chem.* **2010**, *75*, 941-944.
14. Ding, C.-H.; Chen, D.-D.; Luo, Z.-B.; Dai, L.-X.; Hou, X.-L. *Synlett* **2006**, *08*, 1272-1274.
15. Carreira, E. M.; Du Bois, J. *J. Am. Chem. Soc.* **1995**, *117*, 8106-8125.
16. Nishikawa, T.; Ino, A.; Isobe, M. *Tetrahedron* **1994**, *50*, 1449-1468.
17. Alzeer, J.; Vasella, A. *Helv. Chim. Acta* **1995**, *78*, 177-193.
18. Ernst, A.; Gobbi, L.; Vasella, A. *Tetrahedron Lett.* **1996**, *37*, 7959-7962.
19. Mancuso, A. J.; Huang, S.-L.; Swern, D. *J. Org. Chem.* **1978**, *43*, 2480-2482.
20. Hashmi, A. S. K.; Haufe, P.; Rivas Nass, A.; Bats, J. W. *Adv. Synth. Catal.* **2004**, *346*, 421-424.
21. Ishida, T.; Kobayashi, R.; Yamada, T. *Org. Lett.* **2014**, *16*, 2430-2433.
22. Wong, V. H. L.; White, A. J. P.; Hor, T. S. A., Hii, K. K. M. *Adv. Synth. Catal.* **2015**, *357*, 3943-3948.
23. Soler, R.; Cacchi, S.; Fabrizi, G.; Forte, G.; Martín, L.; Martínez, S.; Molins, E.; Moreno-Mañas, M.; Petrucci, F.; Roig, A.; Sebastián, R. M.; Vallribera, A. *Synthesis* **2007**, *19*, 3068-3072.
24. Chuang, C. P.; Gallucci, J. C.; Hart, D. J. *J. Org. Chem.* **1988**, *53*, 3210-3218.
25. Moser, H.; Lu, Q.; Patten, P.; Wang, D.; Kasar, R.; Kaldor, S.; Patterson, B. Preparation of Antibacterial Agents, US66766, publ. date **2008**.
26. Ferreira, F.; Audouin, M.; Chemla, F. *Chem. - Eur. J.* **2005**, *11*, 5269-5278.
27. Tang, T. P.; Ellman, J. A. *J. Org. Chem.* **1999**, *64*, 12-13.
28. Staas, D. D.; Savage, K. L.; Homnick, C. F.; Tsou, N. N.; Ball, R. G. *J. Org. Chem.* **2002**, *67*, 8276-8279.
29. Ye, L.; He, W.; Zhang, L. *Angew. Chem. Int. Ed.* **2011**, *50*, 3236–3239.
30. Prakash, G. K. S.; Mandal, M.; Olah, G. A. *Angew. Chem. Int. Ed Engl.* **2001**, *40,* 589-590.
31. Gross, S.; Heuser, S.; Ammer, C.; Heckmann, G.; Bach, T. *Synthesis* **2011**, *2*, 199-206.
32. Yao, Q.; Yuan, C. *J. Org. Chem.* **2013**, *78*, 6962-6974.
33. García Ruano, J.; Fernández, I.; Prado, C. M.; del Cruz, A. A. *Tetrahedron Asymmetry* **1996**, *7*, 3407-3414.

34. Greier, G. Synthesis of Novel Trifluormethyl-Substitued Thienothiazines, Dissertation, Vienna University of Technology, **1983**.
35. Truong, V. L.; Ménard, M. S.; Ménard, M. S.; Dion, I. *Org. Lett.* **2007**, *9*, 683-685.
36. Fenneteau, J.; Vallerotto, S.; Ferrié, L.; Figadère, B. *Tetrahedron Lett.* **2015**, *56*, 3758-3761.
37. Tang, T. P.; Volkman, S. K.; Ellman, J. A. *J. Org. Chem.* **2001**, *66*, 8772-8778.
38. Roth, S.; Stark, C. B. W. *Angew. Chem. Int. Ed.* **2006**, *45*, 6218-6221.
39. Karmann, L.; Kazmaier, U. *Eur. J. Org. Chem.* **2013**, *31*, 7101-7109.
40. Zhdanko, A. G.; Gulevich, A. V.; Nenajdenko, V. G. *Tetrahedron* **2009**, *65*, 4692-4702.
41. Sasano, Y.; Nagasawa, S.; Yamazaki, M.; Shibuya, M.; Park, J.; Iwabuchi, Y. *Angew. Chem. Int. Ed.* **2014**, *53*, 3236-3240.
42. Srinivasan, R.; Uttamchandani, M.; Yao, S. Q. *Org. Lett.* **2006**, *8*, 713-716.
43. Chen, S.; Zhao, X.; Chen, J.; Chen, J.; Kuznetsova, L.; Wong, S. S.; Ojima, I. *Bioconjug. Chem.* **2010**, *21*, 979-987.
44. Asano, K.; Matsubara, S. *J. Am. Chem. Soc.* **2011**, *133*, 16711-16713.
45. Bauer, R. A.; DiBlasi, C. M.; Tan, D. S. *Org. Lett.* **2010**, *12*, 2084-2087.
46. Wang, T.; Wu, Y.; Kuan, S. L.; Dumele, O.; Lamla, M.; Ng, D. Y. W.; Arzt, M.; Thomas, J.; Mueller, J. O.; Barner-Kowollik, C.; Weil, T. *Chem. - Eur. J.* **2015**, *21*, 228-238.
47. Bachl, J.; Mayr, J.; Sayago, F. J.; Cativiela, C.; Díaz, D. D. *Chem. Commun.* **2015**, *51*, 5294-5297.
48. Shu, C.; Liu, M.-Q.; Wang, S.-S.; Li, L.; Ye, L.-W. *J. Org. Chem.* **2013**, *78*, 3292-3299.
49. Burke, M. J.; Cogan, D.; Gao, D. A.; Heim-Riether, A.; Hickey, E. R.; Ramsden, P. D.; Thompson, D. C.; Xiong, Z., Azolyl substituted carbonitrile derivatives as cathepsin S inhibitors and their preparation and use for the treatment of diseases, *PCT Int. Appl.* **2011**.
50. Jordan, S.; Starks, S. A.; Whatley, M. F.; Turlington, M. *Org. Lett.* **2015**, *17*, 4842-4845.
51. Verrier, C.; Carret, S.; Poisson, J.-F. *Org. Biomol. Chem.* **2014**, *12*, 1875-1878.
52. Basel, Y.; Hassner, A. *Synthesis* **2001**, *4*, 0550-0552.
53. Packer, G.; Malassis, J.; Wells, N.; Light, M.; Linclau, B. *Tetrahedron Asymmetry* **2017**, *28*, 539-544.

X-ray structure analysis

Details of crystal and refinement data can be found in Table S1. CCDC 1566791 - CCDC 1566804 contain the supplementary crystallographic data for this paper. These data can be obtained free of charge from The Cambridge Crystallographic Data Centre via www.ccdc.cam.ac.uk/data_request/cif.

Table S1: Crystal and refinement data.

Identification code	**7a**	**7c**	**7d**
Empirical formula	$C_8H_{15}NOS$	$C_{11}H_{21}NOS$	$C_{13}H_{23}NOS$
Formula weight	173.27	215.35	241.38
Crystal system	orthorhombic	orthorhombic	monoclinic
Space group	$P2_12_12_1$	$P2_12_12_1$	I2
a/Å	8.7104(4)	7.42311(6)	15.2100(2)
b/Å	8.8865(3)	8.20918(9)	5.52038(7)
c/Å	12.8249(5)	21.5429(2)	16.9739(2)
β/°			105.2626(14)
Volume/Å^3	992.71(6)	1312.77(2)	1374.95(3)
Z	4	4	4
ρ_{calc}mg/mm^3	1.156	1.090	1.166
μ/mm^{-1}	2.489	1.965	1.929
F(000)	376.0	472.0	528.0
Crystal size/mm^3	0.306 × 0.19 × 0.178	0.3786 × 0.2147 × 0.0328	0.3786 × 0.1633 × 0.0951
2Θ range for data collection	12.116 to 143.96°	8.2 to 152.8854°	6.95 to 143.932°
Index ranges	-10 ≤ h ≤ 10, -10 ≤ k ≤ 10, -15 ≤ l ≤ 15	-9 ≤ h ≤ 9, -9 ≤ k ≤ 10, -26 ≤ l ≤ 26	-18 ≤ h ≤ 18, -6 ≤ k ≤ 6, -20 ≤ l ≤ 20
Reflections collected	35722	52103	24224
Independent reflections	1942[R(int) = 0.0235]	2663[R(int) = 0.0338]	2673[R(int) = 0.0348]
Reflections with [I ≥ 2σ (I)]	1942	2604	2657
Completness / Θ full	0.99 / 72.0°	0.99 / 76.4	0.99 / 72.0
Data/restraints/parameters	1942/0/108	2663/0/136	2673/1/237
Goodness-of-fit on F^2	1.101	1.038	1.037
Final R indexes [I ≥ 2σ (I)]	R_1 = 0.0182, wR_2 = 0.0500	R_1 = 0.0210, wR_2 = 0.0555	R_1 = 0.0237, wR_2 = 0.0632
Final R indexes [all data]	R_1 = 0.0182, wR_2 = 0.0500	R_1 = 0.0216, wR_2 = 0.0561	R_1 = 0.0238, wR_2 = 0.0633
Largest diff. peak/hole / e Å^{-3}	0.16/-0.19	0.18/-0.22	0.17/-0.18
Flack parameter	0.002(3	-0.015(11)	-0.008(7)

CCDC	1566791	1566792	1566793

Identification code	**7e**	**7i**	**7j**
Empirical formula	$C_{11}H_{21}NOS$	$C_{13}H_{17.03}NOS$	$C_{13}H_{12}F_5NOS$
Formula weight	215.35	235.37	325.30
Crystal system	orthorhombic	monoclinic	monoclinic
Space group	$P2_12_12_1$	$P2_1/c$	I2
a/Å	7.51335(10)	19.2823(6)	16.0227(4)
b/Å	9.18409(9)	6.0708(2)	5.58447(14)
c/Å	18.8630(2)	21.7783(7)	17.1518(4)
β/°		93.696(3)	112.519(3)
Volume/Å^3	1301.61(3)	2544.02(14)	1417.70(6)
Z	4	8	4
ρ_{calc}mg/mm^3	1.099	1.229	1.524
μ/mm^{-1}	1.982	2.084	2.571
F(000)	472.0	1008.0	664.0
Crystal size/mm^3	0.359 × 0.094 × 0.041	0.1499 × 0.0586 × 0.02	0.362 × 0.125 × 0.092
2Θ range for data collection	10.7 to 144.6°	8.136 to 134.09	6.424 to 144.044°
Index ranges	-9 ≤ h ≤ 9, -11 ≤ k ≤ 11, -23 ≤ l ≤ 23	-22 ≤ h ≤ 22, -7 ≤ k ≤ 7, -25 ≤ l ≤ 24	-19 ≤ h ≤ 19, -6 ≤ k ≤ 5, -21 ≤ 21
Reflections collected	21947	40018	10869
Independent reflections	2578[R(int) = 0.0389]	4506 [R_{int} = 0.0445]	2574[R(int) = 0.0242]
Reflections with [I ≥ 2σ (I)]	2544	3847	2536
Completness / Θ full	1.00 / 67.7°	0.99 / 67.0	1.00 / 72.0
Data/restraints/parameters	2578/0/137	4506/0/408	2574/1/207
Goodness-of-fit on F^2	1.039	1.051	1.035
Final R indexes [I ≥ 2σ (I)]	R_1 = 0.0226, wR_2 = 0.0589	R_1 = 0.0457, wR_2 = 0.1275	R_1 = 0.0296, wR_2 = 0.0810
Final R indexes [all data]	R_1 = 0.0230, wR_2 = 0.0592	R_1 = 0.0536, wR_2 = 0.1352	R_1 = 0.0300, wR_2 = 0.0815
Largest diff. peak/hole / e Å^{-3}	0.18/-0.20	0.51/-0.46	0.26/-0.21
Flack parameter	-0.017(7)		-0.010(14)
CCDC	1566794	1566795	1566796

Identification code	**7k**	**7q**	**7s**
Empirical formula	$C_8H_{12}F_3NOS$	$C_{11}H_{18}N_2OS$	$C_{14}H_{25}NO_3S$
Formula weight	227.25	226.33	287.41
Crystal system	orthorhombic	orthorhombic	orthorhombic
Space group	$P2_12_12_1$	$P2_12_12_1$	$P2_12_12_1$
a/Å	9.8970(3)	7.61092(13)	5.99613(6)
b/Å	10.5858(4)	8.47774(13)	11.66404(10)

c/Å	31.7578(12)	20.0077(4)	23.4991(2)
Volume/Å^3	3327.2(2)	1290.96(4)	1643.51(3)
Z	12	4	4
ρ_{calc}mg/mm^3	1.361	1.163	1.162
μ/mm^{-1}	2.766	2.051	1.784
F(000)	1416.0	488.0	624.0
Crystal size/mm^3	0.28 × 0.04 × 0.02	0.495 × 0.33 × 0.225	0.656 × 0.284 × 0.082
2Θ range for data collection	5.566 to 143.994°	8.8 to 144.7°	7.5 to 144.7°
Index ranges	-12 ≤ h ≤ 12, -13 ≤ k ≤ 13, -39 ≤ l ≤ 37	-9 ≤ h ≤ 8, -10 ≤ k ≤ 10, -24 ≤ l ≤ 24	-7 ≤ h ≤ 7, -14 ≤ k ≤ 14, -29 ≤ l ≤ 29
Reflections collected	58973	18319	26461
Independent reflections	6525[R(int) = 0.1078]	2547[R(int) = 0.0234]	3249[R(int) = 0.0557]
Data/restraints/parameters	6525/3/387	2547/0/191	3249/0/182
Reflections with [I ≥ 2σ (I)]	5599	2541	3215
Completness / Θ full	1.00 / 77.0	1.00 / 67.7°	1.00 / 67.7°
Goodness-of-fit on F^2	1.045	1.109	1.042
Final R indexes [I ≥ 2σ (I)]	R_1 = 0.0522, wR_2 = 0.1336	R_1 = 0.0204, wR_2 = 0.0550	R_1 = 0.0253, wR_2 = 0.0677
Final R indexes [all data]	R_1 = 0.0627, wR_2 = 0.1419	R_1 = 0.0204, wR_2 = 0.0550	R_1 = 0.0256, wR_2 = 0.0680
Largest diff. peak/hole / e Å^{-3}	0.61/-0.46	0.16/-0.26	0.23/-0.25
Flack parameter	-0.008(10)	0.003(4)	-0.003(7)
CCDC	1566797	1566798	1566799

Identification code	**10k**	**11i**	**12i**
Empirical formula	$C_7H_{14}F_3NOS$	$C_{21}H_{23}NO_3S$	$C_{21}H_{24}NO_3S$
Formula weight	217.25	369.46	370.47
Crystal system	orthorhombic	monoclinic	orthorhombic
Space group	$P2_12_12_1$	$P2_1$	$P2_12_12_1$
a/Å	5.81687(9)	10.3754(9)	6.03357(10)
b/Å	9.36864(17)	5.8828(3)	10.31093(18)
c/Å	18.6951(3)	15.9341(16)	31.0591(6)
β/°		101.162(8)	
Volume/Å^3	1018.81(3)	954.17(14)	1932.24(6)
Z	4	2	4
ρ_{calc}mg/mm^3	1.416	1.288	1.274
μ/mm^{-1}	0.325	1.668	1.648
F(000)	456.0	392.0	788.0
Crystal size/mm^3	0.383 × 0.143 × 0.104	0.358 × 0.071 × 0.02	0.2716 × 0.123 × 0.028
2Θ range for data collection	4.4 to 60.1°	5.654 to 143.896°	9.036 to 143.992°

Index ranges	-8 ≤ h ≤ 8, -13 ≤ k ≤ 13, -26 ≤ l ≤ 26	-12 ≤ h ≤ 11, -7 ≤ k ≤ 7, -19 ≤ l ≤ 19	-7 ≤ h ≤ 7, -12 ≤ k ≤ 12, -38 ≤ l ≤ 38
Reflections collected	24538	19976	47179
Independent reflections	2991[R(int) = 0.0404]	5148[R(int) = 0.0726]	3806[R(int) = 0.0443]
Reflections with [I ≥ 2σ (I)]	2865	4697	3719
Completness / Θ full	1.00 / 25.2°	1.00 / 71.9	1.00 / 72.0
Data/restraints/parameters	2991/0/174	5148/1/244	3806/0/239
Goodness-of-fit on F^2	1.103	1.128	1.039
Final R indexes [I ≥ 2σ (I)]	R_1 = 0.0237, wR_2 = 0.0588	R_1 = 0.0574, wR_2 = 0.1628	R_1 = 0.0382, wR_2 = 0.0996
Final R indexes [all data]	R_1 = 0.0253, wR_2 = 0.0597	R_1 = 0.0613, wR_2 = 0.1651	R_1 = 0.0391, wR_2 = 0.1005
Largest diff. peak/hole / e $Å^{-3}$	0.24/-0.18	0.72/-0.45	0.82/-0.41
Flack parameter	0.00(3)	-0.03(3)	0.004(5)
CCDC	1566800	1566801	1566802

Identification code	**12i**	**13w**
Empirical formula	$C_{11}H_7F_3O_3$	$C_{13}H_{26}N_4OSSi$
Formula weight	244.17	314.53
Crystal system	monoclinic	orthorhombic
Space group	C2/c	$P2_12_12_1$
a/Å	15.9688(11)	8.6570(8)
b/Å	10.3072(7)	11.4855(4)
c/Å	13.6073(13)	16.8678(7)
β/°	104.846(8)	
Volume/$Å^3$	2164.9(3)	1677.17(18)
Z	8	4
ρ_{calc}mg/mm^3	1.498	1.246
μ/mm^{-1}	0.141	2.414
F(000)	992.0	680.0
Crystal size/mm^3	0.393 × 0.307 × 0.193	0.12 × 0.098 × 0.024
2Θ range for data collection	4.8 to 52.0°	9.3 to 152.4°
Index ranges	-17 ≤ h ≤ 19, -12 ≤ k ≤ 7, -16 ≤ l ≤ 13	-10 ≤ h ≤ 10, -14 ≤ k ≤ 14, -21 ≤ l ≤ 20
Reflections collected	4376	64158
Independent reflections	2089[R(int) = 0.0339]	3495[R(int) = 0.0390]
Reflections with [I ≥ 2σ (I)]	1473	3451
Completness / Θ full	0.97 / 26.0°	1.00 / 67.7°
Data/restraints/parameters	2089/0/182	3495/0/191

Goodness-of-fit on F^2	1.060	1.046
Final R indexes [I ≥ 2σ (I)]	R_1 = 0.0509, wR_2 = 0.1105	R_1 = 0.0203, wR_2 = 0.0525
Final R indexes [all data]	R_1 = 0.0798, wR_2 = 0.1307	R_1 = 0.0207, wR_2 = 0.0528
Largest diff. peak/hole / e Å^{-3}	0.28/-0.30	0.19/-0.26
Flack parameter		-0.012(4)
CCDC	1566803	1566804

(*S*)-*N*-((*S*)-But-3-yn-2-yl)-2-methylpropane-2-sulfinamide (**7a**)

Single crystals of $C_8H_{15}NOS$ (**7a**) were achieved out of a saturated solution in *n*-hexane.

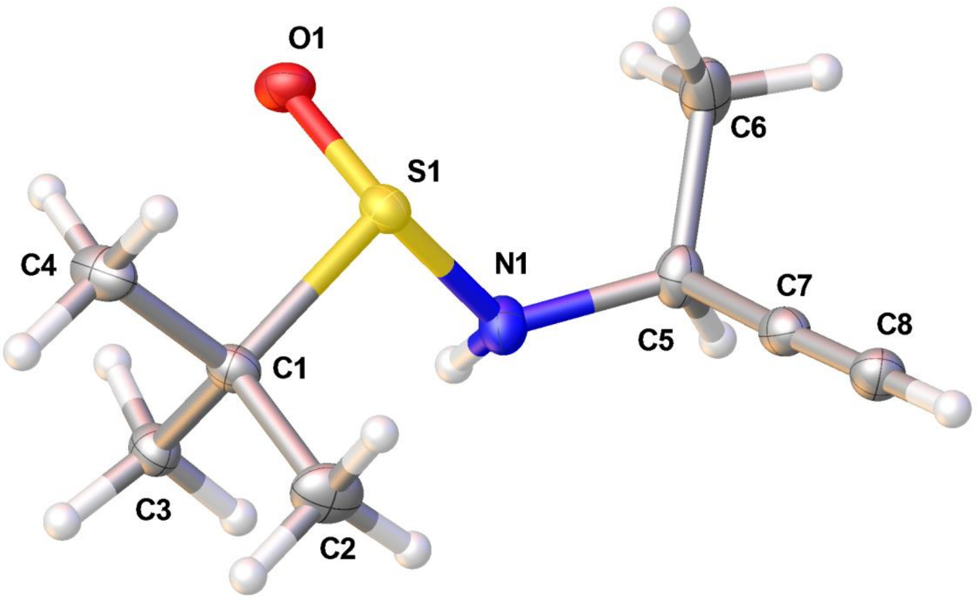

Table S2: Fractional Atomic Coordinates (×10^4) and Equivalent Isotropic Displacement Parameters (Å^2×10^3) for **7a**. U_{eq} is defined as 1/3 of of the trace of the orthogonalised U_{IJ} tensor.

**Atom**	***x***	***y***	***z***	**U(eq)**
S1	4843.9(4)	4082.3(4)	3561.6(3)	17.22(10)
O1	5343.1(12)	3554.0(11)	2502.1(8)	22.0(3)
N1	3965.5(14)	5716.5(15)	3484(1)	20.7(3)
C1	6627.7(17)	4706.0(17)	4201.2(11)	18.1(3)
C2	6204(2)	5205(2)	5303.7(13)	28.6(4)
C3	7377.5(16)	5951.2(17)	3568.0(12)	19.9(3)
C4	7643(2)	3305.6(18)	4228.9(13)	25.7(3)
C5	2269.5(16)	5753.4(19)	3434.2(11)	20.9(3)
C6	1557.4(19)	4628(2)	2681.1(12)	30.8(4)
C7	1596.3(17)	5604.3(16)	4487.0(11)	18.8(3)
C8	974.7(17)	5521.9(17)	5305.8(12)	21.2(3)

(*S*)-2-Methyl-*N*-((*S*)-5-methylhex-1-yn-3-yl)propane-2-sulfinamide (**7c**)

Single crystals of $C_{11}H_{21}NOS$ (**7c**) were achieved out of a saturated solution in Et_2O.

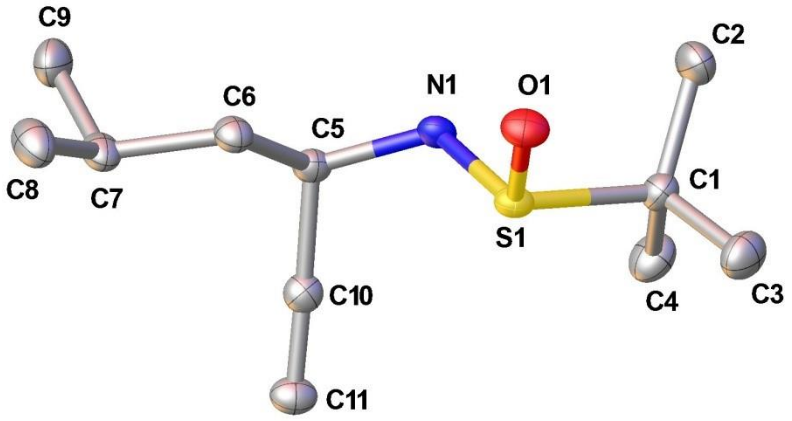

Table S3: Fractional Atomic Coordinates (×10^4) and Equivalent Isotropic Displacement Parameters (Å^2×10^3) for **7c**. U_{eq} is defined as 1/3 of of the trace of the orthogonalised U_{IJ} tensor.

	x	y	z	U(eq)
S(1)	7880(1)	7020(1)	2111(1)	17(1)
O(1)	9617(1)	6189(1)	2274(1)	24(1)
N(1)	7693(1)	8762(1)	2485(1)	19(1)

C(1)	8254(2)	7839(2)	1325(1)	22(1)
C(2)	9956(2)	8868(2)	1310(1)	31(1)
C(3)	8447(2)	6331(2)	915(1)	32(1)
C(4)	6580(2)	8818(2)	1151(1)	30(1)
C(5)	6535(2)	8811(2)	3041(1)	19(1)
C(6)	7264(2)	7802(2)	3587(1)	21(1)
C(7)	6238(2)	8029(2)	4198(1)	24(1)
C(8)	6802(2)	6743(2)	4668(1)	33(1)
C(9)	6522(2)	9737(2)	4466(1)	33(1)
C(10)	4686(2)	8300(1)	2880(1)	20(1)
C(11)	3203(2)	7854(2)	2767(1)	24(1)

(*S*)-*N*-((*S*)-1-Cyclohexylprop-2-yn-1-yl)-2-methylpropane-2-sulfinamide (**7d**)

Single crystals of $C_{13}H_{23}NOS$ (**7d**) were achieved out of a saturated solution in Et_2O.

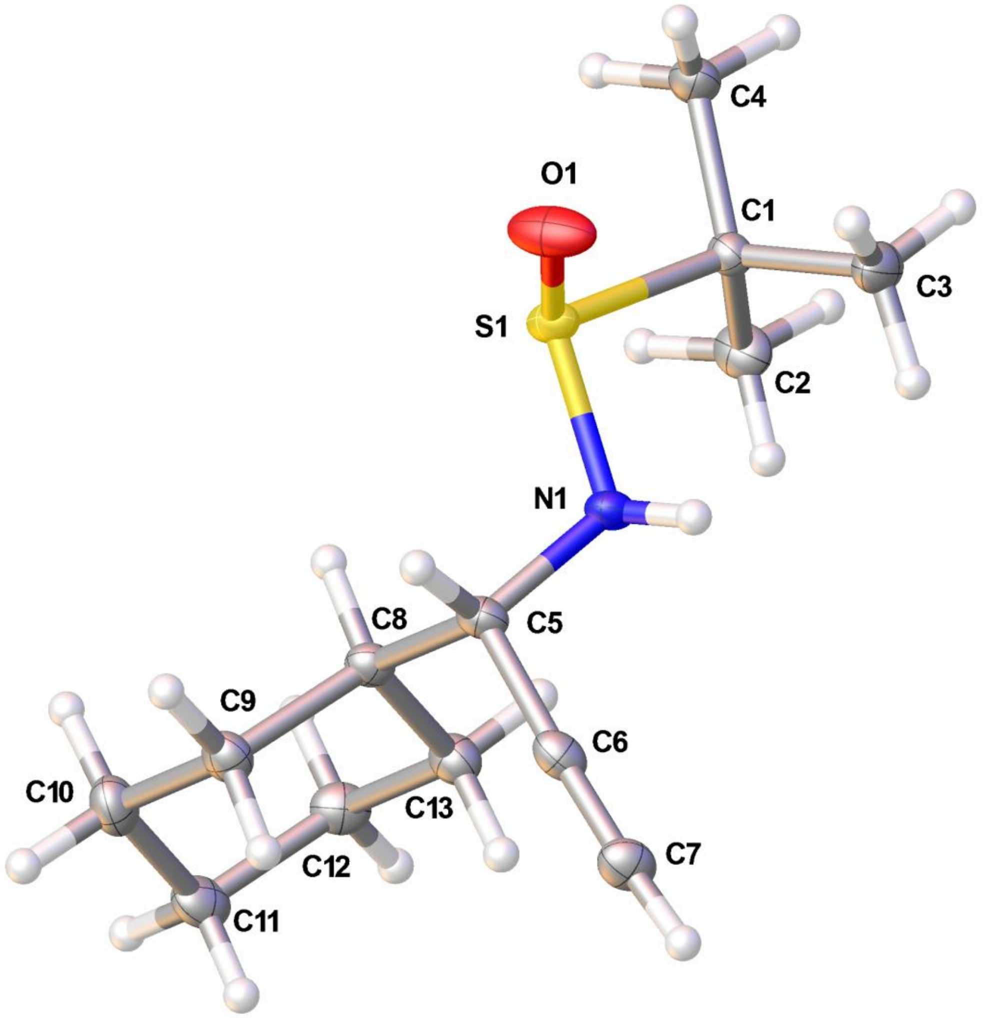

Table S4: Fractional Atomic Coordinates (×10^4) and Equivalent Isotropic Displacement Parameters (Å^2×10^3) for **7d**. U_{eq} is defined as 1/3 of of the trace of the orthogonalised U_{IJ} tensor.

**Atom**	***x***	***y***	***z***	**U(eq)**
S1	4850.7(3)	7186.4(8)	2984.7(2)	18.86(13)
O1	4233.6(11)	5726(3)	3346.1(8)	35.4(4)
N1	5283.8(10)	9543(3)	3577.4(9)	18.8(3)
C1	4100.5(11)	8906(4)	2140(1)	16.4(3)
C2	4697.8(13)	10562(4)	1782.7(12)	23.7(4)

C3	3384.1(13)	10288(4)	2437.4(13)	24.9(4)
C4	3655.5(12)	6968(4)	1522.8(11)	21.7(4)
C5	6080.2(11)	8941(4)	4276.4(10)	17.9(4)
C6	6162.8(12)	10872(4)	4887.4(11)	19.7(4)
C7	6202.8(12)	12455(4)	5365.2(11)	22.5(4)
C8	6950.7(11)	8662(4)	3979.8(10)	17.4(4)
C9	7735.5(13)	7725(3)	4675.3(11)	20.7(4)
C10	8609.8(12)	7436(5)	4397.7(11)	25.1(4)
C11	8868.3(12)	9812(4)	4058.5(12)	24.8(4)
C12	8091.6(13)	10716(4)	3352.5(11)	22.8(4)
C13	7213.5(12)	11016(4)	3621.7(11)	20.7(4)

(*S*)-*N*-((*S*)-4,4-Dimethylpent-1-yn-3-yl)-2-methylpropane-2-sulfinamide (**7e**)

Single crystals of $C_{11}H_{21}NOS$ (**7e**) were achieved out of a saturated solution in EtOAc.

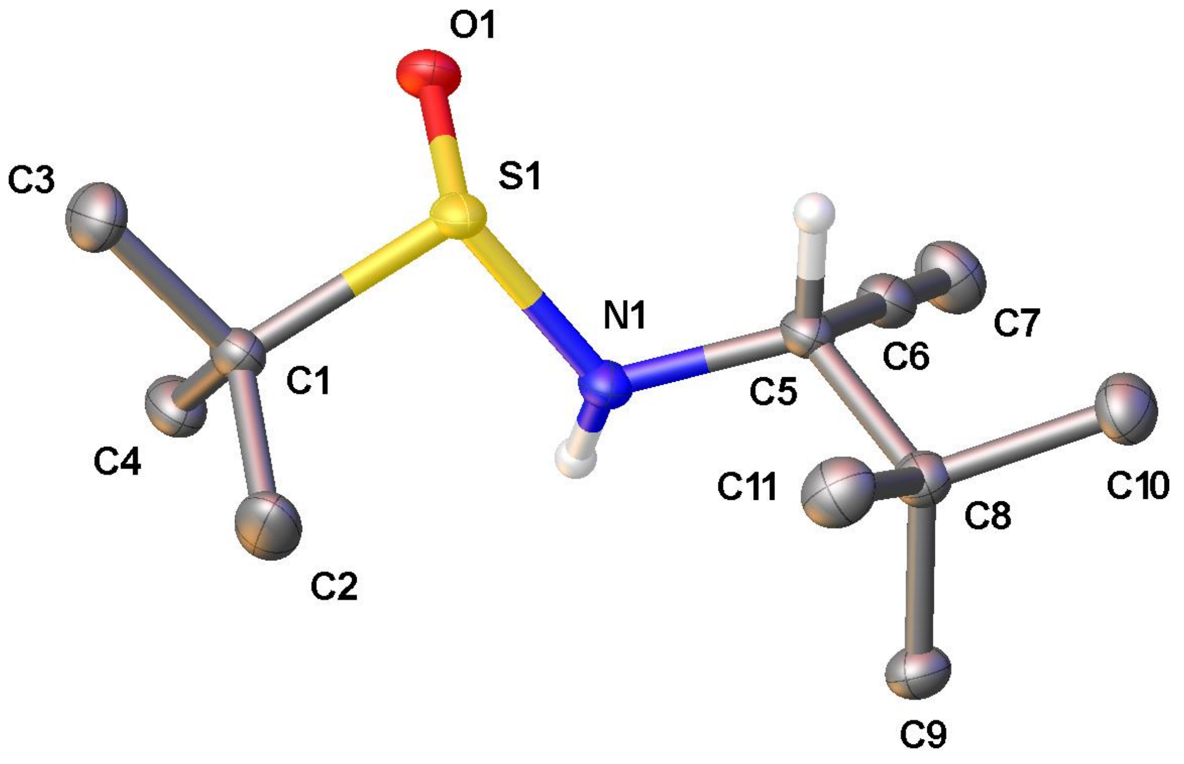

Table S5: Fractional Atomic Coordinates (×10^4) and Equivalent Isotropic Displacement Parameters (Å^2×10^3) for **7e**. U_{eq} is defined as 1/3 of of the trace of the orthogonalised U_{IJ} tensor.

**Atom**	***x***	***y***	***z***	**U(eq)**
S1	8548.3(5)	2785.4(4)	7938.1(2)	17.89(11)
O1	10227.9(17)	1989.8(12)	7769.9(6)	24.0(3)
N1	8117.3(18)	4096.2(14)	7365.9(7)	18.5(3)
C1	9074(2)	3908.6(18)	8723.7(8)	19.9(3)
C2	7561(3)	4982(2)	8850.7(10)	31.9(4)
C3	9181(3)	2790(2)	9326.6(9)	25.9(4)
C4	10861(3)	4658.1(19)	8623.7(9)	25.1(4)
C5	7310(2)	3605.5(17)	6692.9(8)	18.3(3)
C6	8626(2)	3639.6(17)	6112.6(8)	21.5(3)
C7	9685(3)	3684(2)	5646.6(10)	28.2(4)
C8	5586(2)	4460.8(17)	6511.3(8)	19.0(3)
C9	6011(2)	6076.1(18)	6400.0(9)	21.7(3)
C10	4790(2)	3819(2)	5831.1(9)	26.2(4)
C11	4254(2)	4268(2)	7116.5(10)	26.8(4)

(*S*)-*N*-[(*R*)-1-Phenylprop-2-yn-1-yl]-2-methylpropansulfinamide (**7i**)

Single crystals of $C_{13}H_{17}NOS$ (**7i**) were achieved out of a saturated solution in DCM. Nearly the complete molecule is disordered in ratio 93:7. All atoms of minor occupied part were refined isotropically with idealized geometry of the phenyl rings.

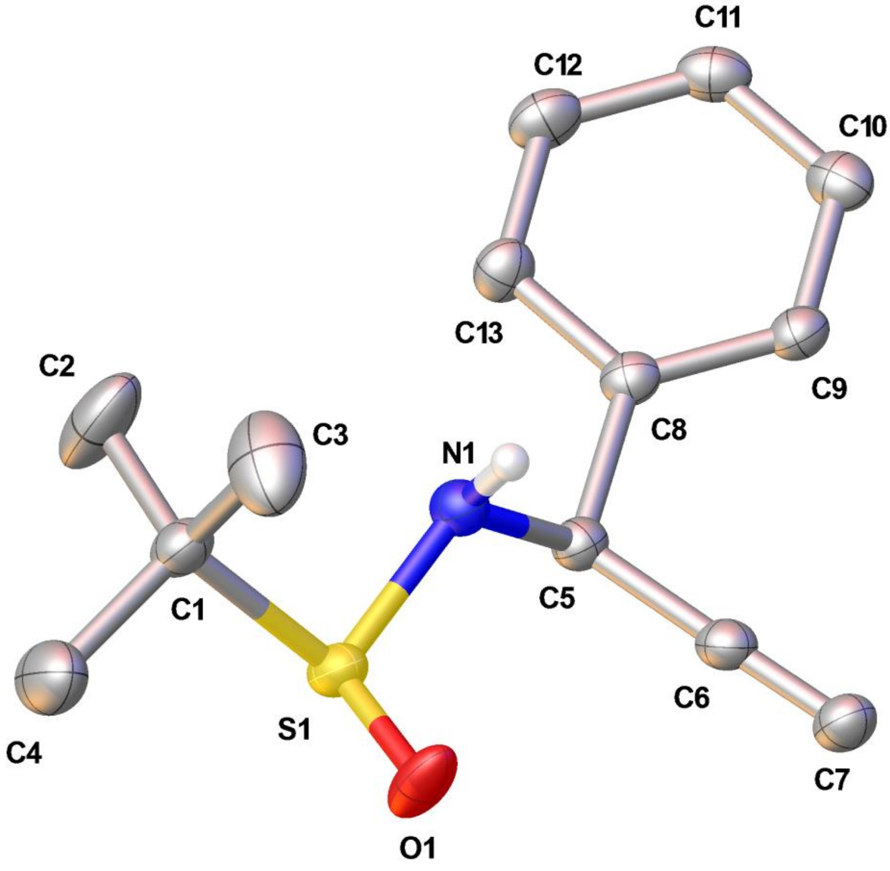

Table S6: Fractional Atomic Coordinates ($\times 10^4$) and Equivalent Isotropic Displacement Parameters ($Å^2 \times 10^3$) for **7i**. U_{eq} is defined as 1/3 of of the trace of the orthogonalised U_{IJ} tensor.

**Atom**	***x***	***y***	***z***	**U(eq)**
S1	7828.1(3)	10703.0(9)	7870.6(2)	25.96(17)
O1	8277.5(8)	9077(3)	8212.5(8)	39.1(4)
N1	7217.5(9)	9490(3)	7406.2(8)	24.1(4)
C1	8360.5(13)	11792(4)	7265.1(10)	28.9(5)
C2	7910.8(15)	13337(6)	6865.1(15)	58.7(9)

C3	8652.2(16)	9920(5)	6900.8(14)	55.5(8)
C4	8946.5(16)	13082(6)	7612.1(13)	47.4(7)
C5	6572.6(10)	8862(3)	7700.9(9)	22.0(4)
C6	6665.8(11)	6879(5)	8089.3(12)	23.9(5)
C7	6744.8(11)	5262(4)	8391.2(10)	27.1(5)
C8	5981.8(10)	8636(3)	7208.2(9)	21.2(4)
C9	5566.9(10)	6754(3)	7153.6(9)	23.2(4)
C10	5026.6(11)	6618(4)	6699.4(10)	26.9(4)
C11	4890.1(10)	8359(4)	6299.9(9)	27.1(5)
C12	5296.2(11)	10252(4)	6356.2(10)	29.4(5)
C13	5836.7(10)	10389(3)	6805.5(10)	25.7(4)
S2	7129.3(2)	5427.9(8)	4568.7(2)	24.50(17)
O2	6689.4(8)	3740(3)	4242.8(8)	39.8(4)
N2	7756.2(9)	4319(3)	5033.0(8)	23.6(4)
C14	6593.9(11)	6506(3)	5172.7(9)	26.1(4)
C15	5967.7(13)	7518(5)	4824.5(11)	46.0(6)
C16	7021.5(16)	8238(6)	5529.8(16)	58.0(8)
C17	6362.3(14)	4672(5)	5582.9(12)	46.0(7)
C18	8411.9(10)	3760(4)	4741(1)	22.1(4)
C19	8354.9(10)	1687(5)	4387.9(10)	24.2(5)
C20	8284.8(10)	47(4)	4096.9(10)	27.6(5)
C21	9005.5(10)	3766(3)	5240.2(9)	22.7(4)
C22	9431.4(11)	1955(4)	5354.5(9)	27.4(5)
C23	9978.2(11)	2055(4)	5803.7(10)	33.2(5)
C24	10104.1(11)	3967(4)	6137.2(10)	34.4(5)
C25	9678.4(12)	5772(4)	6028.2(12)	39.3(6)
C26	9131.4(12)	5677(4)	5583.3(11)	33.5(5)
S1B	7868(3)	4632(12)	7964(3)	25.96(17)
O1B	8322(12)	6280(40)	8270(10)	38(5)

N1B	7236(13)	5700(40)	7495(11)	28(5)
C1B	8388(16)	3400(60)	7352(14)	32(7)
C2B	8050(30)	1960(80)	7080(20)	54(12)
C3B	8611(19)	5280(60)	6937(17)	43(8)
C4B	9095(16)	2190(50)	7657(15)	16(7)
C5B	6598(14)	6320(50)	7843(15)	16(7)
C6B	6646(17)	8020(70)	8146(16)	29(8)
C7B	6742(15)	9980(50)	8444(14)	31(6)
C8B	5979(11)	6390(40)	7266(10)	39(8)
C9B	5570(12)	8270(30)	7192(10)	27(6)
C10B	5020(13)	8310(40)	6746(12)	48(9)
C11B	4881(14)	6490(50)	6374(13)	110(20)
C12B	5290(15)	4610(40)	6448(12)	76(14)
C13B	5840(13)	4560(30)	6894(12)	39(7)
S2B	7165(3)	9458(11)	4677(3)	24.50(17)
O2B	6701(13)	11100(40)	4336(12)	46(6)
N2B	7788(13)	10610(40)	5154(12)	30(5)
C14B	6660(20)	8250(80)	5240(20)	55(10)
C16B	7100(30)	6630(120)	5570(30)	94(18)
C17B	6390(20)	10120(70)	5664(19)	55(10)
C18B	8438(12)	11170(40)	4856(12)	17(5)
C19B	8348(14)	12770(60)	4447(15)	14(6)
C20B	8251(13)	14810(40)	4147(12)	22(5)
C21B	9057(9)	11370(30)	5342(7)	31(7)
C22B	9464(10)	13260(30)	5396(8)	20(5)
C23B	9991(9)	13420(30)	5861(9)	23(6)
C24B	10111(8)	11680(30)	6271(7)	23(5)
C25B	9704(9)	9800(20)	6217(7)	24(6)
C26B	9177(9)	9640(30)	5753(8)	24(6)

(*S*)-2-Methyl-*N*-((*R*)-1-(perfluorophenyl)prop-2-yn-1-yl)propane-2-sulfinamide (**7j**)

Single crystals of $C_{13}H_{12}F_5NOS$ (**7j**) were achieved out of a saturated solution in Et_2O. The NSO unit is disorder at two positions with ratio 73:27. The anisotropic displacement parameters of these three atoms were constrained to be same pairwisely.

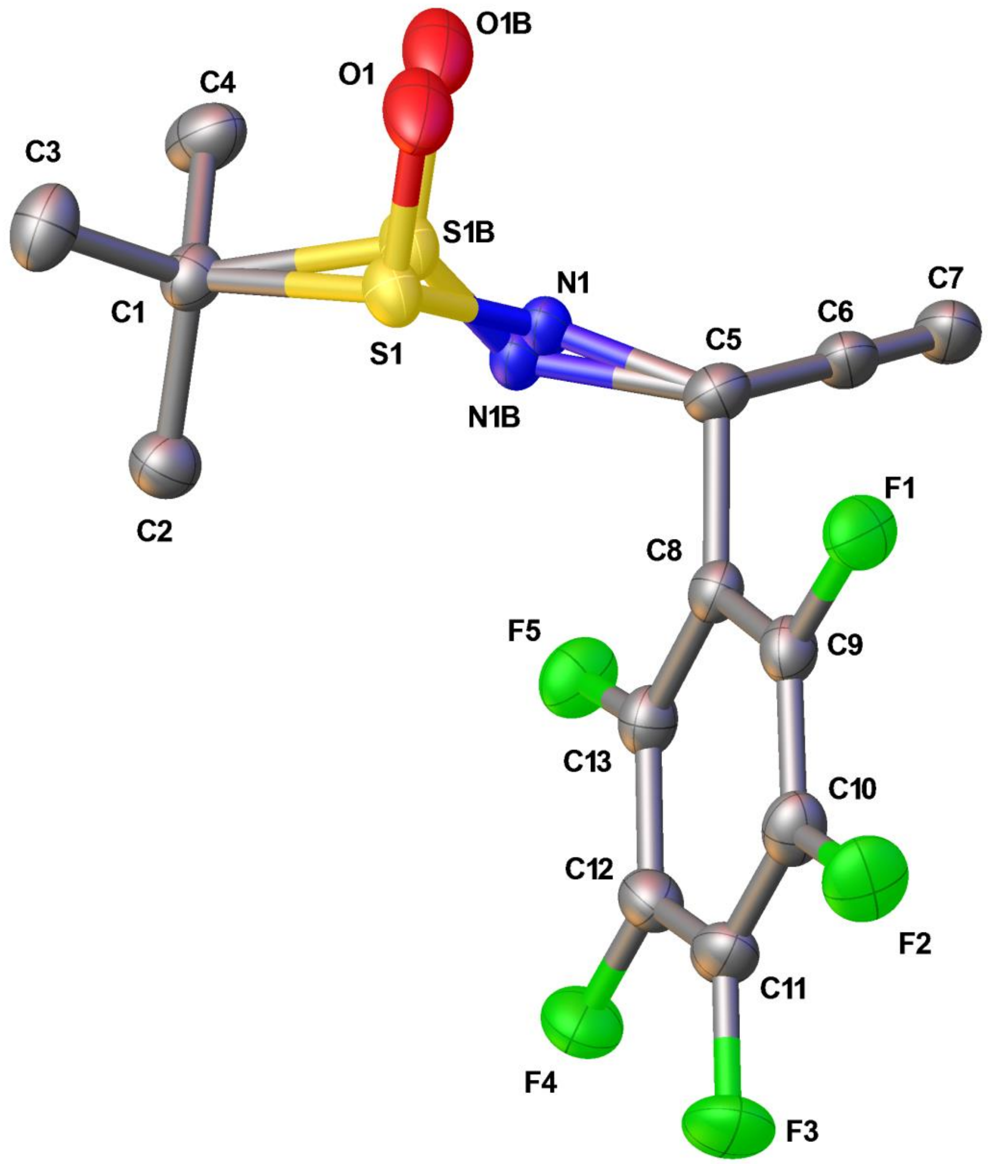

Table S7: Fractional Atomic Coordinates (×10[4]) and Equivalent Isotropic Displacement Parameters (Å[2]×10[3]) for **7j**. U_{eq} is defined as 1/3 of of the trace of the orthogonalised U_{IJ} tensor.

**Atom**	***x***	***y***	***z***	**U(eq)**
S1	5074.3(11)	-2084(2)	7010.6(9)	25.9(3)
S1B	5256(3)	-1807(9)	6885(3)	25.9(3)
F1	2475.2(9)	-3333(3)	5104.1(9)	34.4(4)
F2	982.5(10)	-3091(4)	5448.4(10)	40.8(4)
F3	738.7(10)	630(4)	6361.7(11)	43.5(4)
F4	2025.6(11)	4075(3)	6945(1)	39.9(4)
F5	3513.1(9)	3889(3)	6595.3(10)	34.5(4)
O1	5588(2)	-3759(7)	6691.6(19)	49.5(10)
O1B	5907(7)	-2700(20)	6544(5)	49.5(10)
N1	4736(6)	356(17)	6359(4)	24.6(11)
N1B	4708(18)	580(60)	6524(14)	24.6(11)
C1	5918.5(15)	-521(5)	7928.5(15)	26.0(5)
C2	5368.4(16)	1094(5)	8258.8(16)	31.9(6)
C3	6345.5(18)	-2568(5)	8542.2(16)	35.3(6)
C4	6634.6(17)	841(6)	7723.3(18)	37.6(6)
C5	3899.2(14)	81(5)	5633.4(15)	28.4(5)
C6	3863.1(15)	1923(6)	5011.6(15)	31.2(6)
C7	3850.0(15)	3426(6)	4523.2(16)	35.4(7)
C8	3057.0(14)	229(5)	5849.3(14)	23.8(5)
C9	2390.4(15)	-1476(5)	5567.0(13)	25.9(5)
C10	1607.7(16)	-1376(5)	5730.9(15)	29.3(5)
C11	1489.0(16)	500(6)	6194.7(15)	30.0(5)
C12	2141.5(16)	2255(5)	6488.0(14)	28.8(6)
C13	2914.0(15)	2115(5)	6310.9(14)	26.4(5)

(*S*)-2-Methyl-*N*-((*R*)-1,1,1-trifluorobut-3-yn-2-yl)propane-2-sulfinamide (**7k**)

Single crystals of $C_{13}H_{12}F_5NOS$ (**7k**) were achieved out of a saturated solution in EtOAc. One C(CCH)(CF_3) unit is disordered in ratio 51:49. The anisotropic displacement parameters of these atoms were constrained to be same pairwisely. The N-H distances were restrained to a value of 0.86 Å.

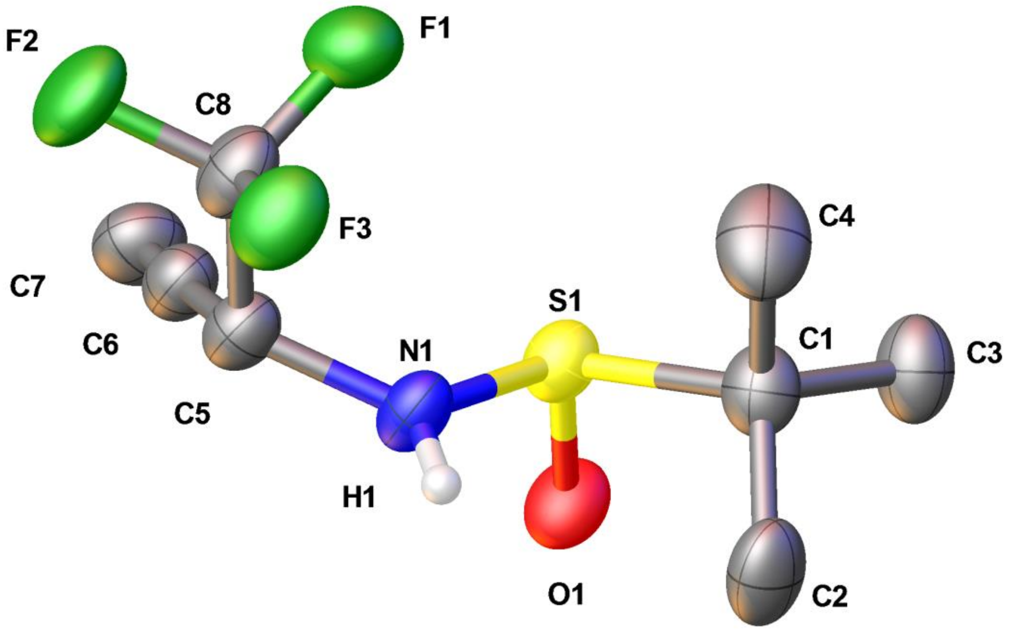

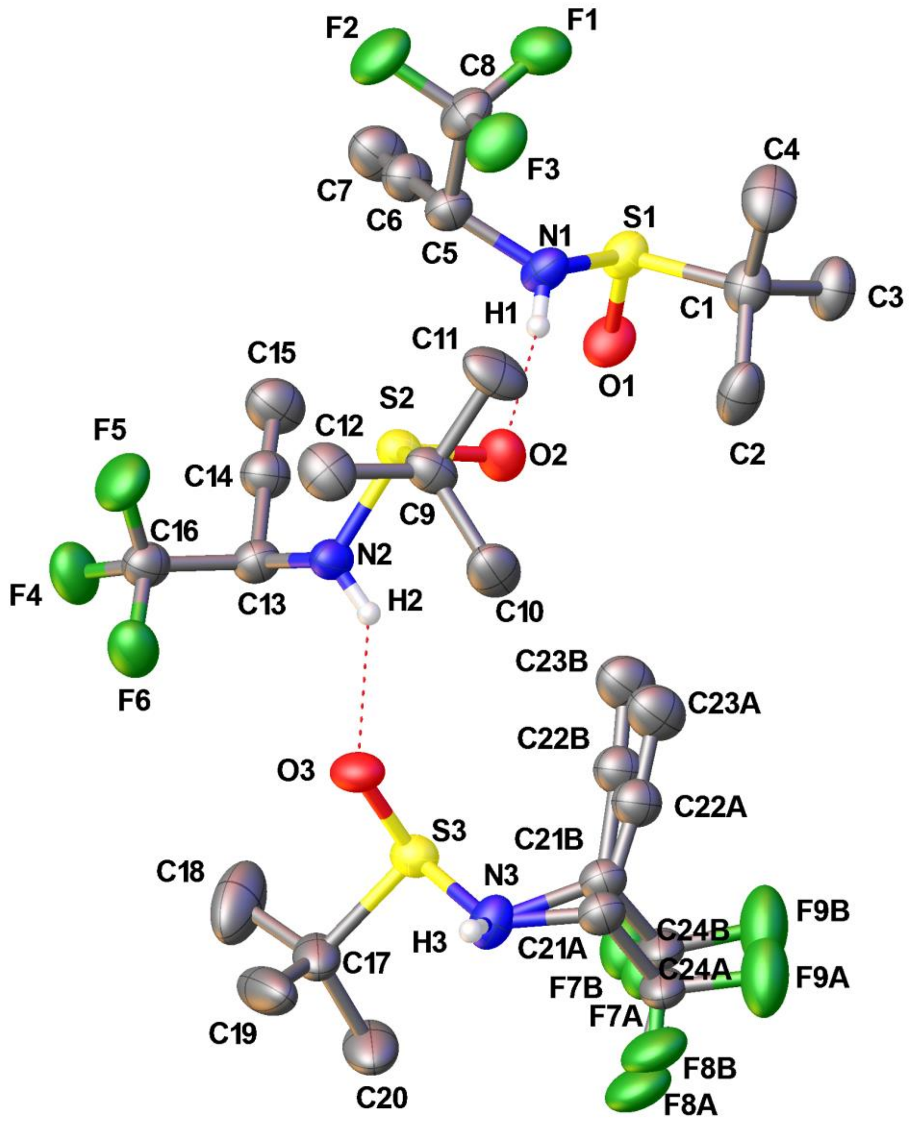

Table S8: Fractional Atomic Coordinates ($\times 10^4$) and Equivalent Isotropic Displacement Parameters (Å$^2\times 10^3$) for **7k**. U_{eq} is defined as 1/3 of of the trace of the orthogonalised U_{IJ} tensor.

**Atom**	***x***	***y***	***z***	**U(eq)**
S1	7760.9(13)	9077.2(11)	3804.3(4)	36.5(3)
F1	7273(4)	9709(3)	4812.8(10)	53.6(8)
F2	5421(4)	9013(3)	5078.7(10)	58.8(9)
F3	7090(4)	7752(3)	4991.4(11)	54.4(9)
O1	7269(4)	8891(3)	3365.0(11)	42.2(8)

N1	7071(5)	8024(4)	4120.7(14)	37.2(9)
C1	9492(5)	8446(5)	3808.4(17)	41.1(11)
C2	9504(6)	7069(6)	3679(2)	54.1(15)
C3	10270(6)	9245(6)	3484.2(18)	50.5(14)
C4	10059(7)	8666(7)	4247.5(19)	63.0(17)
C5	5926(5)	8383(5)	4379.5(17)	37.4(7)
C6	5095(5)	9405(5)	4208.4(18)	42.4(8)
C7	4477(6)	10243(6)	4055(2)	56.3(10)
C8	6423(6)	8719(5)	4819.1(18)	45.0(13)
S2	5700.7(12)	4808.3(11)	4256.3(4)	34.4(3)
F4	1123(3)	4290(3)	3811.1(13)	57.1(9)
F5	2207(4)	4129(4)	4395.7(11)	60.8(9)
F6	2253(3)	2619(3)	3952.6(12)	53.5(8)
O2	6794(4)	5379(3)	3994.1(12)	44.1(9)
N2	4720(4)	3890(4)	3964.4(14)	34.3(9)
C9	6528(5)	3589(5)	4573.9(18)	41.0(12)
C10	7297(6)	2664(5)	4298(2)	51.3(14)
C11	7476(7)	4334(6)	4861(2)	58.5(17)
C12	5423(6)	2932(6)	4835.4(19)	52.9(14)
C13	3502(5)	4435(4)	3775.6(18)	37.4(7)
C14	3433(5)	5810(5)	3788.7(18)	42.4(8)
C15	3405(6)	6922(6)	3791(2)	56.3(10)
C16	2266(5)	3882(5)	3989.2(17)	40.0(11)
S3	5297.5(13)	2044.2(11)	2848.1(4)	36.4(3)
F7A	7850(40)	2060(40)	2116(16)	55(4)
F7B	7580(40)	2290(40)	2157(15)	55(4)
F8A	8070(30)	20(20)	2140(8)	67(4)
F8B	8280(30)	390(20)	2220(8)	67(4)
F9A	9710(20)	1249(17)	2323(7)	85(4)

F9B	9630(20)	1920(16)	2369(7)	85(4)
O3	4824(4)	2244(3)	3290.3(11)	42.2(8)
N3	6475(4)	939(4)	2835.2(15)	42.4(10)
C17	3941(6)	1168(7)	2594.1(19)	52.6(15)
C18	2760(7)	2120(10)	2593(3)	95(3)
C19	3633(8)	-26(7)	2841(3)	83(3)
C20	4381(7)	883(8)	2145(2)	72(2)
C21A	7890(12)	1105(11)	2793(4)	37.4(7)
C21B	7881(12)	1507(11)	2841(4)	37.4(7)
C22A	8324(13)	2333(13)	2994(4)	42.4(8)
C22B	8082(13)	2687(13)	3066(4)	42.4(8)
C23A	8577(16)	3279(14)	3180(5)	56.3(10)
C23B	8163(15)	3629(14)	3236(5)	56.3(10)
C24A	8400(30)	1070(30)	2343(10)	48(4)
C24B	8350(30)	1510(20)	2418(9)	48(4)

(*S*)-2-Methyl-*N*-((*R*)-1,1,1-trichlorobut-3-yn-2-yl)propane-2-sulfinamide (**7l**)

Single crystals of $C_8H_{12}Cl_3NOS$ (**7l**) were achieved out of a saturated solution in DCM/Et_2O, 1:2.

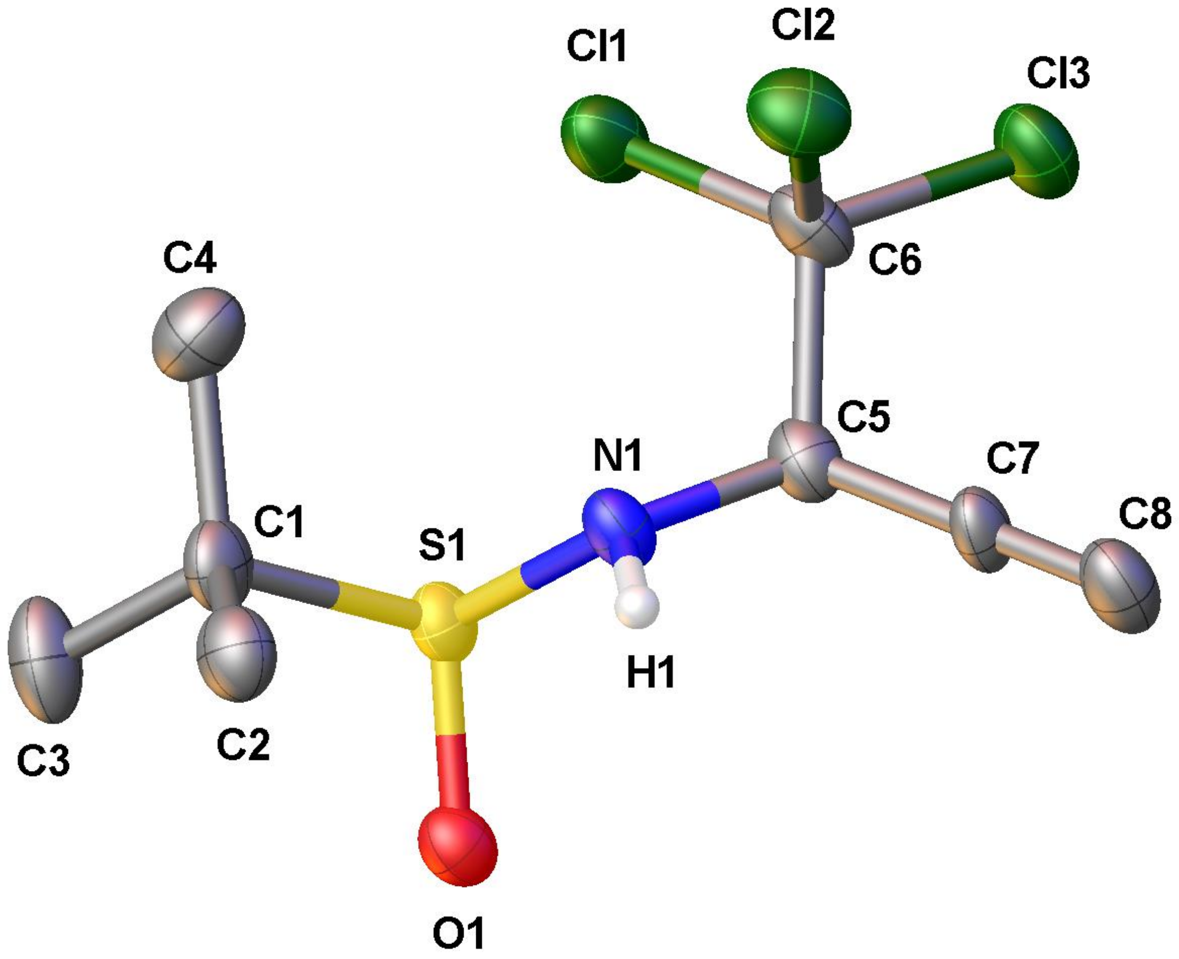

Table S9: Fractional Atomic Coordinates ($\times 10^4$) and Equivalent Isotropic Displacement Parameters ($Å^2 \times 10^3$) for propargylamine **7l**. U_{eq} is defined as 1/3 of of the trace of the orthogonalised U_{IJ} tensor.

**Atom**	***x***	***y***	***z***	**U(eq)**
Cl1	3854(3)	3874.3(16)	6848.8(8)	39.4(5)
Cl2	7752(3)	5568.0(18)	6928.0(8)	41.6(5)
Cl3	3641(3)	6492.0(16)	6324.8(8)	40.1(5)
S1	6445(3)	1954.7(14)	5685.1(7)	26.7(4)
O1	6925(7)	1547(4)	5011(2)	29.8(10)
N1	7652(9)	3412(5)	5844(3)	27.6(11)
C1	8229(12)	902(6)	6208(3)	33.9(14)
C2	10723(12)	1067(7)	6052(3)	35.9(15)
C3	7415(13)	-495(7)	6062(4)	45.9(19)
C4	7715(13)	1292(8)	6909(3)	41.4(17)

C5	6220(12)	4581(6)	5772(3)	30.0(13)
C6	5445(12)	5109(7)	6443(3)	34.0(15)
C7	7421(11)	5629(6)	5430(3)	28.4(13)
C8	8451(13)	6461(7)	5161(4)	38.8(15)

(*S*)-*N*-((*S*)-6-Cyanohex-1-yn-3-yl)-2-methylpropane-2-sulfinamide (**7q**)

Single crystals of $C_{11}H_{18}N_2OS$ (**7q**) were achieved out of a saturated solution in EtOAc.

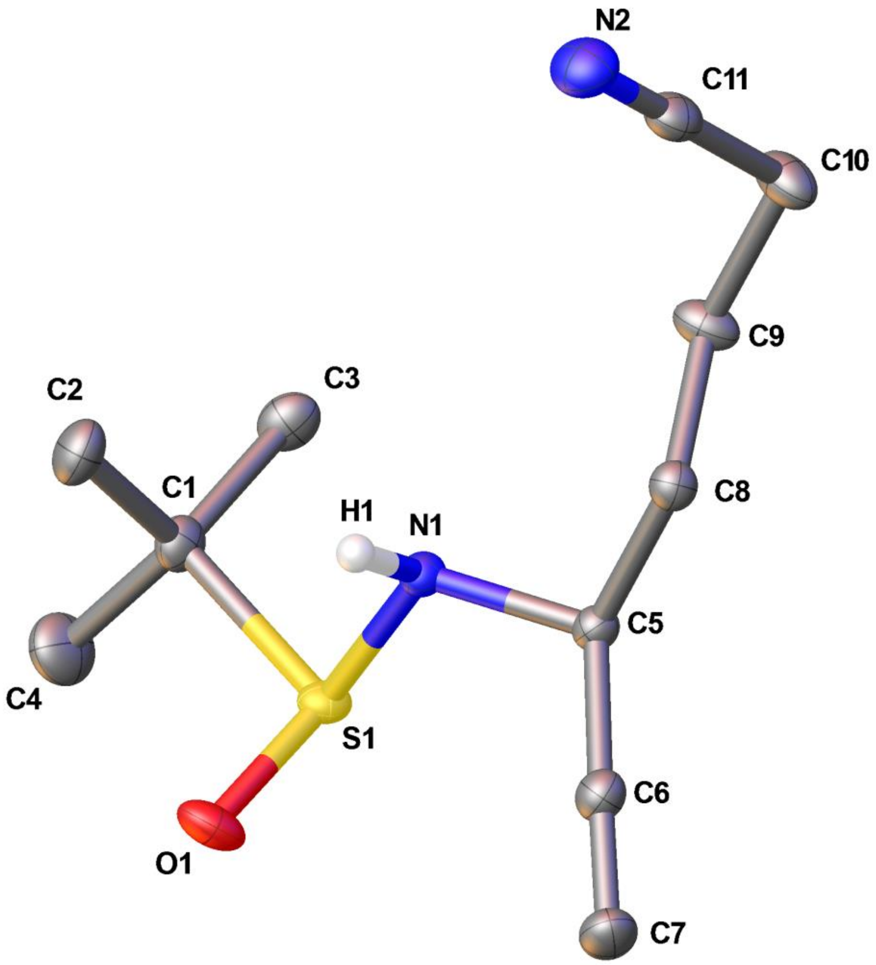

Table S10: Fractional Atomic Coordinates ($\times10^4$) and Equivalent Isotropic Displacement Parameters ($Å^2\times10^3$) for **7q**. U_{eq} is defined as 1/3 of of the trace of the orthogonalised U_{IJ} tensor.

**Atom**	***x***	***y***	***z***	**U(eq)**
S1	2694.2(4)	6821.9(4)	3380.1(2)	15.64(10)
O1	815.5(13)	6379.1(12)	3473.2(6)	24.4(2)
N1	4006.7(16)	5374.0(13)	3643.7(6)	14.2(2)
N2	8058.6(19)	1710.6(16)	5076.5(6)	27.4(3)
C1	3170(2)	8274.2(16)	4040.2(7)	18.8(3)
C2	2906(2)	7528.6(19)	4724.4(7)	24.4(3)
C3	5041(2)	8856.7(18)	3936.1(7)	22.3(3)
C4	1839(3)	9596.2(19)	3921.3(9)	31.5(4)
C5	4616.7(18)	4270.9(16)	3121.2(6)	15.0(3)
C6	3182.5(19)	3405.8(16)	2786.4(7)	18.4(3)
C7	2039(2)	2751.5(18)	2488.9(7)	23.3(3)
C8	5970.1(17)	3153.9(15)	3436.4(6)	15.7(3)
C9	7639(2)	4017.0(17)	3633.3(7)	21.2(3)
C10	9037(2)	2945.1(19)	3941.0(8)	24.1(3)
C11	8491(2)	2250.1(16)	4578.7(7)	20.6(3)

tert-Butyl-(*S*)-4-(((*S*)-*tert*-butylsulfinyl)amino)hex-5-inoate (**7s**)

Single crystals of $C_{14}H_{25}NO_3S$ (**7s**) were achieved out of a saturated solution in EtOAc.

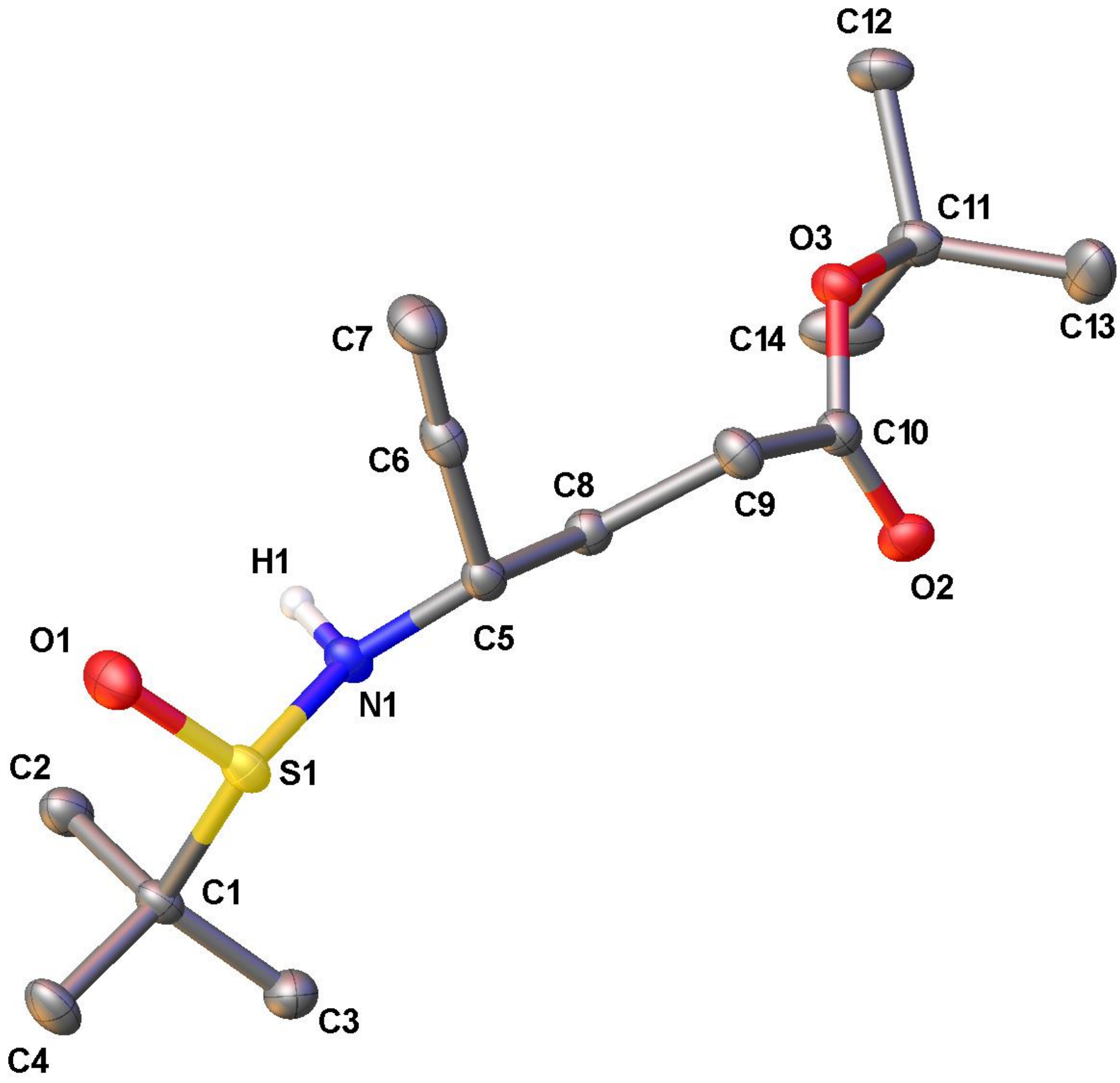

Table S11: Fractional Atomic Coordinates (×10^4) and Equivalent Isotropic Displacement Parameters (Å^2×10^3) for **7s**. U_{eq} is defined as 1/3 of of the trace of the orthogonalised U_{IJ} tensor.

**Atom**	***x***	***y***	***z***	**U(eq)**
S1	7903.9(6)	5097.1(3)	7148.7(2)	18.42(11)
O1	10079(2)	4508.1(11)	7057.4(5)	27.3(3)
O2	-1022(2)	5817.7(11)	5440.9(5)	22.3(3)
O3	686.4(18)	4938.6(10)	4704.4(4)	18.9(2)
N1	6662(2)	5389.8(12)	6531.2(6)	18.2(3)
C1	8608(3)	6584.0(14)	7356.4(7)	18.3(3)

C2	10077(3)	7148.8(15)	6907.8(7)	22.9(3)
C3	6439(3)	7237.1(16)	7446.3(7)	25.2(4)
C4	9888(3)	6454.6(16)	7915.6(7)	26.0(4)
C5	5047(3)	4538.3(13)	6316.0(6)	17.5(3)
C6	6090(3)	3500.7(15)	6067.4(7)	20.9(3)
C7	6853(3)	2681.4(15)	5842.6(8)	28.3(4)
C8	3596(3)	5144.3(14)	5870.8(6)	17.8(3)
C9	1748(3)	4385.8(14)	5631.9(7)	20.3(3)
C10	281(3)	5117.6(14)	5258.8(6)	17.9(3)
C11	-421(3)	5653.3(15)	4265.1(7)	20.8(3)
C12	549(3)	5181.1(16)	3714.1(7)	26.7(4)
C13	-2930(3)	5478(2)	4284.3(8)	33.9(4)
C14	258(4)	6898.2(15)	4343.7(8)	33.0(4)

(*S*)-2-Methyl-*N*-((*R*)-1,1,1-trifluoropropan-2-yl)propane-2-sulfinamide (**10k**)

Single crystals of $C_7H_{14}F_3NOS$ (**10k**) were achieved out of a saturated solution in EtOAc.

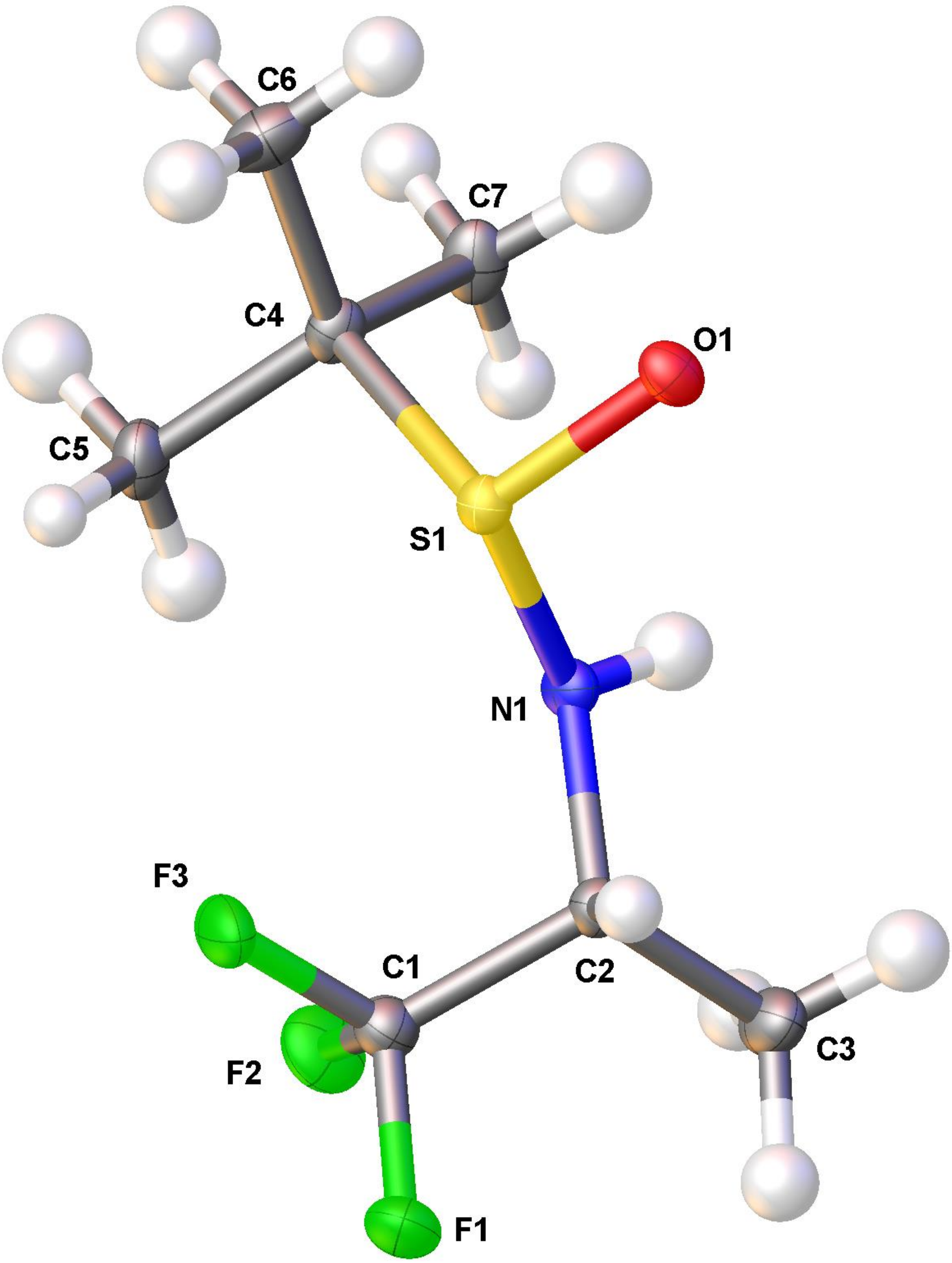
C6
C7
C4
O1
C5
S1
N1
F3
C1
C2
C3
F2
F1

Table S12: Fractional Atomic Coordinates (×10^4) and Equivalent Isotropic Displacement Parameters (Å^2×10^3) for **10k**. U_{eq} is defined as 1/3 of of the trace of the orthogonalised U_{IJ} tensor.

**Atom**	***x***	***y***	***z***	**U(eq)**
S1	8146.3(6)	7780.4(4)	5813.0(2)	12.92(9)
F1	10538(2)	3219.2(11)	6109.6(6)	25.2(2)
F2	7680(2)	3890.0(12)	6758.6(6)	28.5(3)
F3	10563(2)	5288.4(12)	6609.2(6)	26.3(2)
O1	7612.7(19)	8589.2(13)	5142.0(6)	16.8(2)
N1	6760(2)	6224.1(14)	5807.8(7)	14.9(2)
C1	9212(3)	4347.0(18)	6275.4(8)	18.3(3)
C2	8103(3)	4964.2(17)	5609.8(8)	14.8(3)
C3	6546(3)	3856(2)	5265.9(10)	23.7(4)
C4	6511(3)	8669.5(18)	6528.3(8)	15.8(3)
C5	7096(3)	7886(2)	7220.4(9)	21.7(3)
C6	7420(3)	10196(2)	6541.9(11)	25.0(4)
C7	3944(3)	8636(2)	6378.9(10)	21.7(4)

Methyl 4-((*S*)-3-(((*S*)-*tert*-Butylsulfinyl)amino)-3-phenylprop-1-yn-1-yl)benzoate (**11i**)

Single crystals of $C_{21}H_{23}NO_3S$ (**11i**) were achieved out of a saturated solution in $CHCl_3$. The crystal was twinned with ratio 60:40 by a rotation of 180° around 100. Both domains were taken into account during data reduction and refinement.

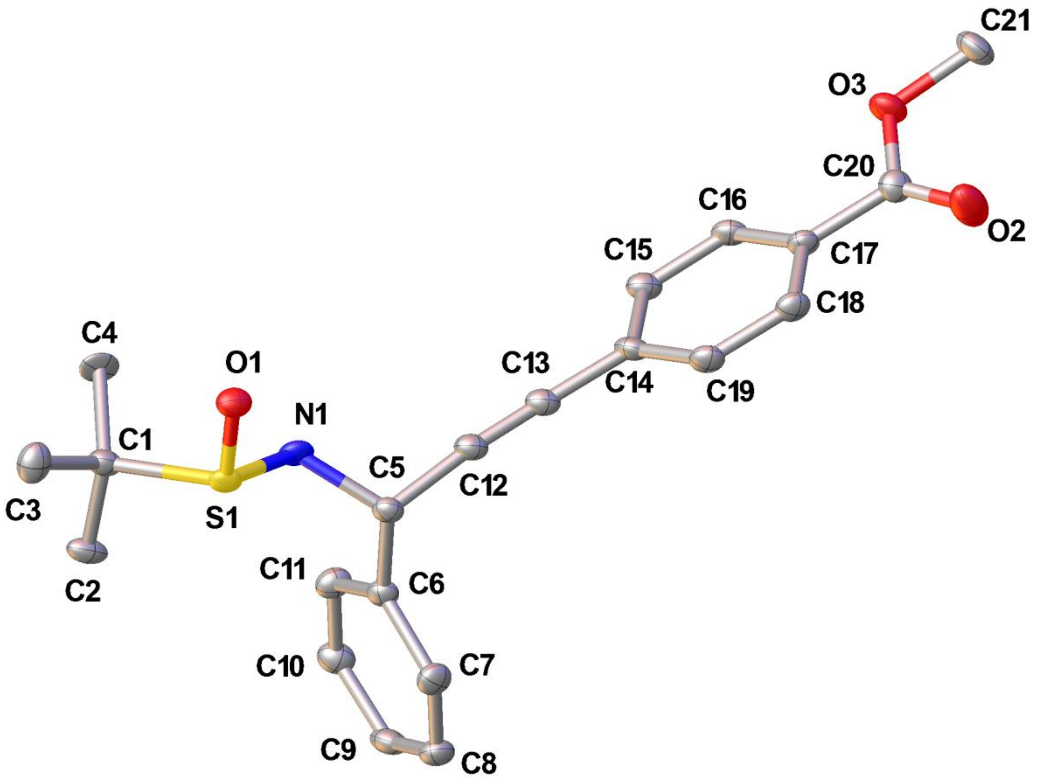

Table S13: Fractional Atomic Coordinates ($\times 10^4$) and Equivalent Isotropic Displacement Parameters (Å$^2\times 10^3$) for **11i**. U_{eq} is defined as 1/3 of of the trace of the orthogonalised U_{IJ} tensor.

**Atom**	***x***	***y***	***z***	**U(eq)**
S1	3625.0(14)	1722(3)	5182.7(8)	17.6(3)
O1	5017(4)	2166(8)	5617(3)	21.3(10)
O2	8272(6)	5050(11)	-390(4)	39.8(14)
O3	7635(5)	8606(9)	-177(3)	25.5(11)
N1	3295(6)	3101(10)	4254(3)	20.3(11)

C1	2594(6)	3384(12)	5789(4)	21.5(13)
C2	1180(6)	2645(13)	5458(4)	24.9(14)
C3	3065(7)	2617(14)	6717(4)	28.3(16)
C4	2786(7)	5923(12)	5694(4)	25.4(14)
C5	3283(6)	1620(13)	3498(3)	18.0(11)
C6	1901(6)	1006(11)	3050(3)	18.7(13)
C7	1676(7)	-1122(13)	2655(4)	23.8(14)
C8	445(7)	-1634(13)	2184(4)	24.6(14)
C9	-573(7)	-43(13)	2103(4)	25.1(14)
C10	-351(6)	2018(14)	2513(4)	24.7(15)
C11	885(7)	2566(12)	2984(4)	22.4(13)
C12	3964(6)	2721(13)	2873(4)	20.1(13)
C13	4477(6)	3494(13)	2324(4)	20.2(13)
C14	5203(6)	4279(12)	1692(4)	20.5(14)
C15	5001(6)	6405(13)	1306(4)	22.0(14)
C16	5779(6)	7106(12)	736(4)	21.1(14)
C17	6741(6)	5669(12)	536(3)	19.0(13)
C18	6922(7)	3527(13)	897(4)	21.6(13)
C19	6160(6)	2839(12)	1477(4)	21.4(14)
C20	7613(6)	6354(13)	-59(4)	22.9(15)
C21	8496(7)	9386(15)	-739(4)	29.8(16)

Methyl 4-((1*E*,3*Z*)-3-(((*S*)-*tert*-Butylsulfinyl)imino)-3-phenylprop-1-en-1-yl)benzoate (**12i**)

Single crystals of $C_{21}H_{23}NO_3S$ (**12i**) were achieved out of a saturated solution in $CHCl_3$.

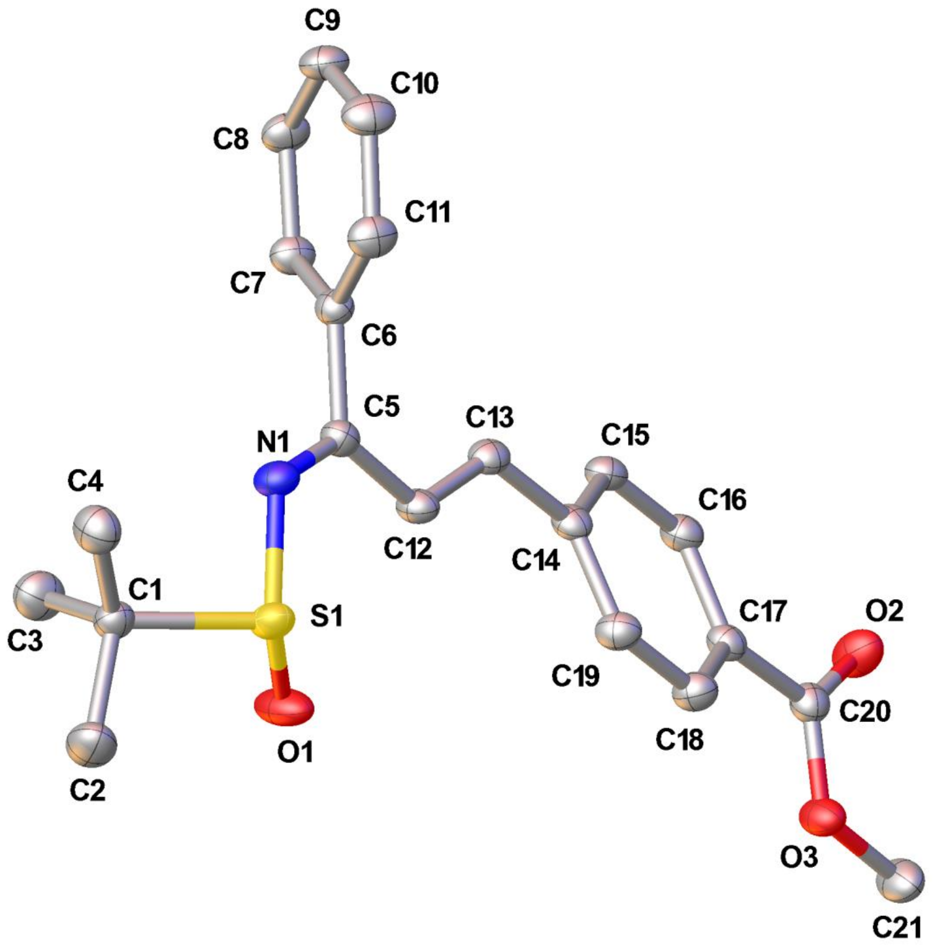

Table S14: Fractional Atomic Coordinates (×10[4]) and Equivalent Isotropic Displacement Parameters (Å[2]×10[3]) for **12i**. U_{eq} is defined as 1/3 of of the trace of the orthogonalised U_{IJ} tensor.

**Atom**	***x***	***y***	***z***	**U(eq)**
S1	5313.5(11)	5191.5(6)	3501.6(2)	26.65(17)
O1	3568(4)	6178(2)	3586.6(8)	40.6(6)
O2	-7437(3)	5126(2)	5629.0(7)	33.0(5)

O3	-5236(3)	6883.7(17)	5585.6(6)	28.8(4)
N1	4352(4)	3670(2)	3444.7(7)	24.7(5)
C1	6103(4)	5394(3)	2929.1(8)	23.5(5)
C2	7134(6)	6752(3)	2910.6(10)	37.5(7)
C3	4073(5)	5320(3)	2643.6(10)	35.3(7)
C4	7818(5)	4369(3)	2822.5(10)	32.6(6)
C5	2672(4)	3189(3)	3652.1(8)	21.8(5)
C6	2266(4)	1784(2)	3565.6(8)	20.8(5)
C7	296(5)	1351(2)	3386.1(8)	24.2(5)
C8	-3(5)	38(3)	3299.2(9)	26.5(5)
C9	1650(5)	-848(3)	3403.3(9)	28.0(6)
C10	3592(5)	-420(3)	3591.3(10)	30.6(6)
C11	3923(5)	892(3)	3668.3(9)	27.3(6)
C12	1280(4)	3866(2)	3967.0(8)	22.5(5)
C13	-305(4)	3275(2)	4199.4(8)	22.0(5)
C14	-1651(4)	3914(2)	4530.8(8)	21.1(5)
C15	-3746(4)	3405(2)	4629.2(8)	22.4(5)
C16	-5087(4)	3993(2)	4932.5(8)	22.9(5)
C17	-4345(4)	5092(2)	5150.5(8)	21.0(5)
C18	-2247(5)	5597(3)	5062.6(9)	25.5(6)
C19	-924(4)	5005(3)	4755.1(9)	24.3(5)

C20	-5837(5)	5680(3)	5480.5(8)	23.9(6)
C21	-6645(6)	7512(3)	5898.5(10)	35.2(7)

Methyl 3-((1*E*,3*Z*)-3-(((*S*)-*tert*-Butylsulfinyl)imino)-4,4,4-trifluorobut-1-en-1-yl)benzoate (**12k**)

Single crystals of $C_{11}H_7F_3O_3$ (**12k**) were achieved out of a saturated solution in $CHCl_3$.

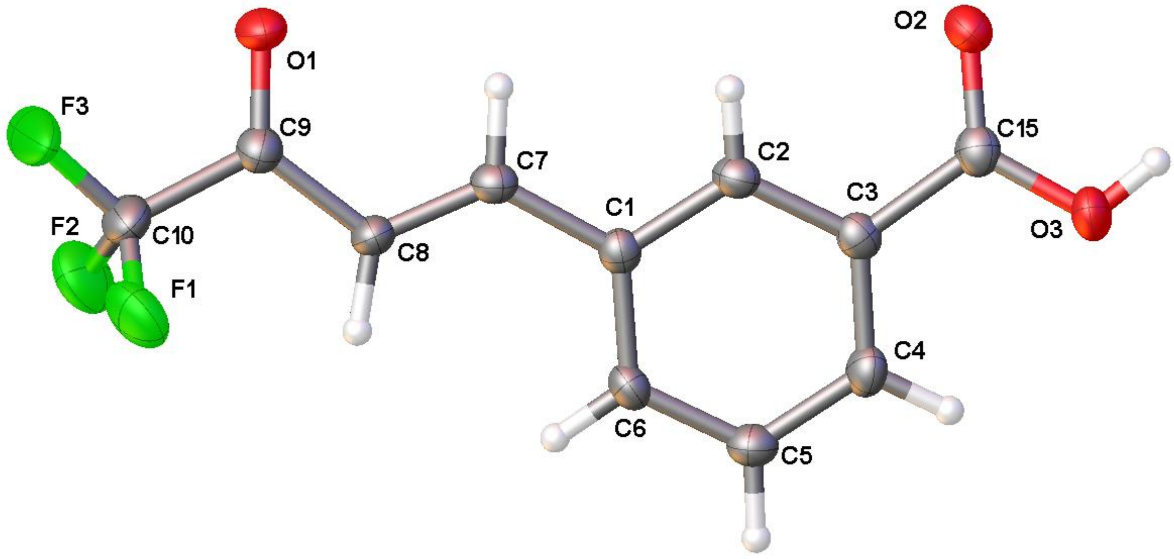

Table S15: Fractional Atomic Coordinates ($\times10^4$) and Equivalent Isotropic Displacement Parameters ($Å^2\times10^3$) for **12k**. U_{eq} is defined as 1/3 of of the trace of the orthogonalised U_{IJ} tensor.

**Atom**	***x***	***y***	***z***	**U(eq)**
F1	1467.9(9)	6458.8(17)	1152.3(11)	49.7(5)
F2	1058(1)	6633.7(18)	2521.6(13)	56.5(5)
F3	583.9(10)	5069.4(19)	1493.9(16)	82.2(8)
O1	1913(1)	3810.2(18)	2611.1(13)	37.3(5)
O2	6460.9(10)	2704.4(18)	4669.3(12)	33.6(5)
O3	7476.2(10)	4218(2)	4694.8(13)	34.1(5)
C1	4484.7(13)	5390(2)	3557.0(15)	21.7(5)

C2	5145.0(14)	4473(3)	3864.5(17)	24.0(6)
C3	6004.6(13)	4855(3)	4202.6(16)	22.9(5)
C4	6213.4(14)	6167(3)	4242.9(17)	26.6(6)
C5	5567.0(15)	7082(3)	3956.6(17)	26.9(6)
C6	4707.5(14)	6706(3)	3608.7(17)	24.8(6)
C7	3593.3(14)	4922(3)	3204.7(17)	24.5(6)
C8	2877.7(13)	5612(3)	2801.6(17)	24.7(6)
C9	2046.3(14)	4945(3)	2468.1(18)	27.8(6)
C10	1276.7(15)	5782(3)	1898(2)	39.0(7)
C15	6668.0(14)	3833(3)	4538.2(16)	25.9(6)

(*S*)-2-Methyl-*N*-((*S*)-3-(Trimethylsilyl)-4,5,6,7-tetrahydro-[1,2,3]triazolo[1,5-a]pyridin-4-yl)propane-2-sulfinamide (**13w**)

Single crystals of $C_{13}H_{26}N_4OSSi$ (**13w**) were achieved out of a saturated solution in toluene.

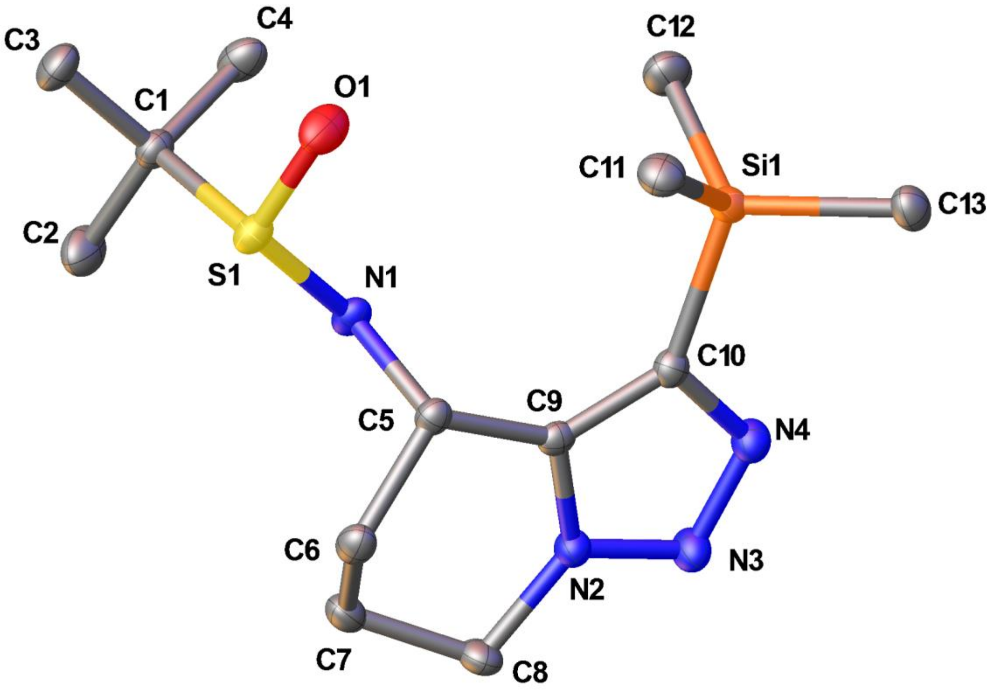

Table S16: Fractional Atomic Coordinates (×10^4) and Equivalent Isotropic Displacement Parameters (Å^2×10^3) for **13w**. U_{eq} is defined as 1/3 of of the trace of the orthogonalised U_{IJ} tensor.

**Atom**	***x***	***y***	***z***	**U(eq)**
S1	7886.8(4)	4926.8(3)	6980.4(2)	17.48(9)
Si1	4242.6(5)	4312.6(4)	4794.9(3)	16.7(1)
O1	7588.4(15)	3706.2(11)	6735.9(8)	28.0(3)
N1	6982.1(15)	5879.4(12)	6404.5(8)	17.0(3)
N2	3991.5(16)	7522.0(11)	5766.3(8)	16.6(3)

N3	3223.0(18)	7664.4(12)	5080.6(9)	20.8(3)
N4	3235.2(16)	6654.8(12)	4714.0(9)	20.0(3)
C1	9879.4(18)	5246.7(14)	6671.3(10)	18.4(3)
C2	10248(2)	6508.0(16)	6874.0(13)	29.8(4)
C3	10861(2)	4409.7(16)	7167.8(11)	25.6(4)
C4	10072.8(19)	4976.3(17)	5795.5(10)	24.8(4)
C5	5319.6(17)	6029.0(14)	6583.2(9)	15.7(3)
C6	5113.7(19)	6918.2(15)	7249.5(10)	20.1(3)
C7	5401.8(19)	8147.2(15)	6934.7(11)	21.8(3)
C8	4167(2)	8474.0(14)	6336.7(10)	21.9(3)
C9	4481.3(17)	6410.7(13)	5848.5(9)	14.7(3)
C10	4005.2(17)	5844.8(13)	5167.9(10)	16.0(3)
C11	4213(2)	3269.7(14)	5643.2(11)	24.1(3)
C12	6144(2)	4190.3(17)	4278.8(11)	27.5(4)
C13	2626.6(19)	4024.2(15)	4095.1(10)	22.7(3)

Spectra

Ethyl (3-hydroxypropynyl)-benzoate derivatives **1**

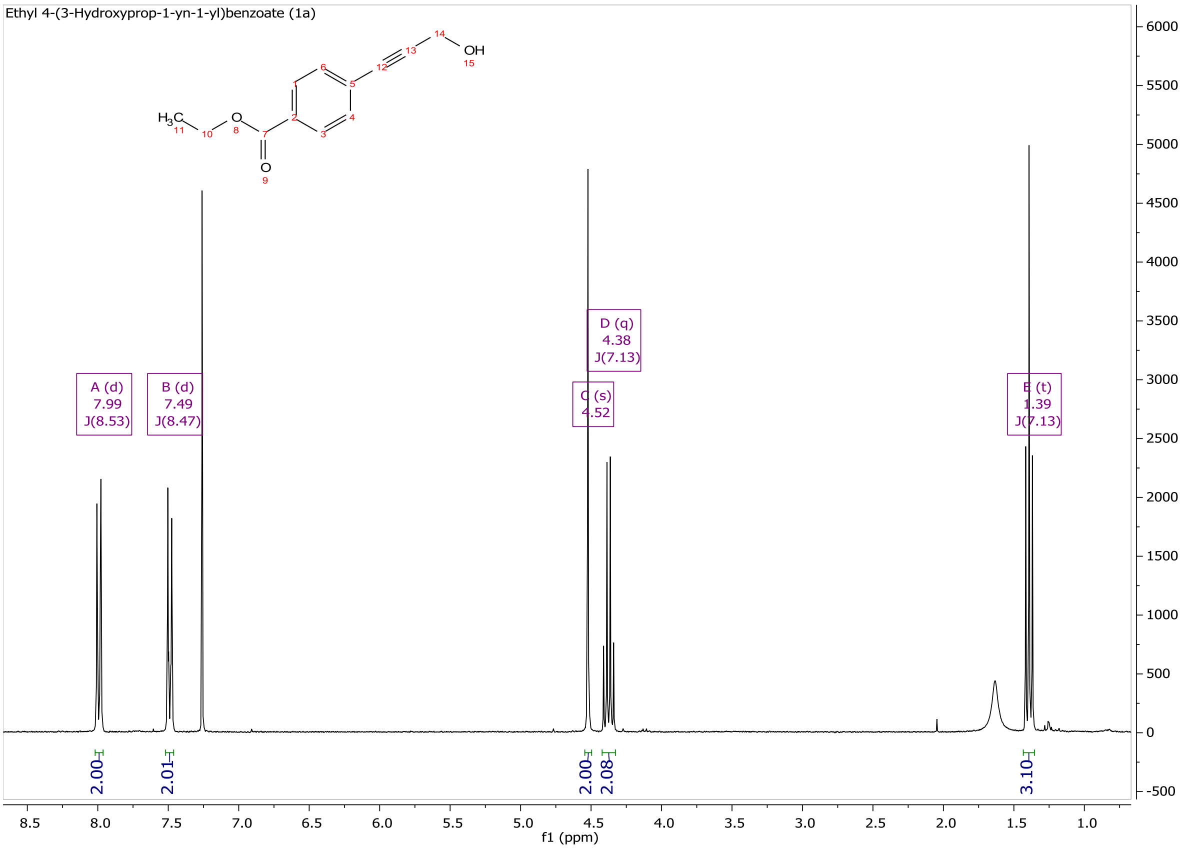

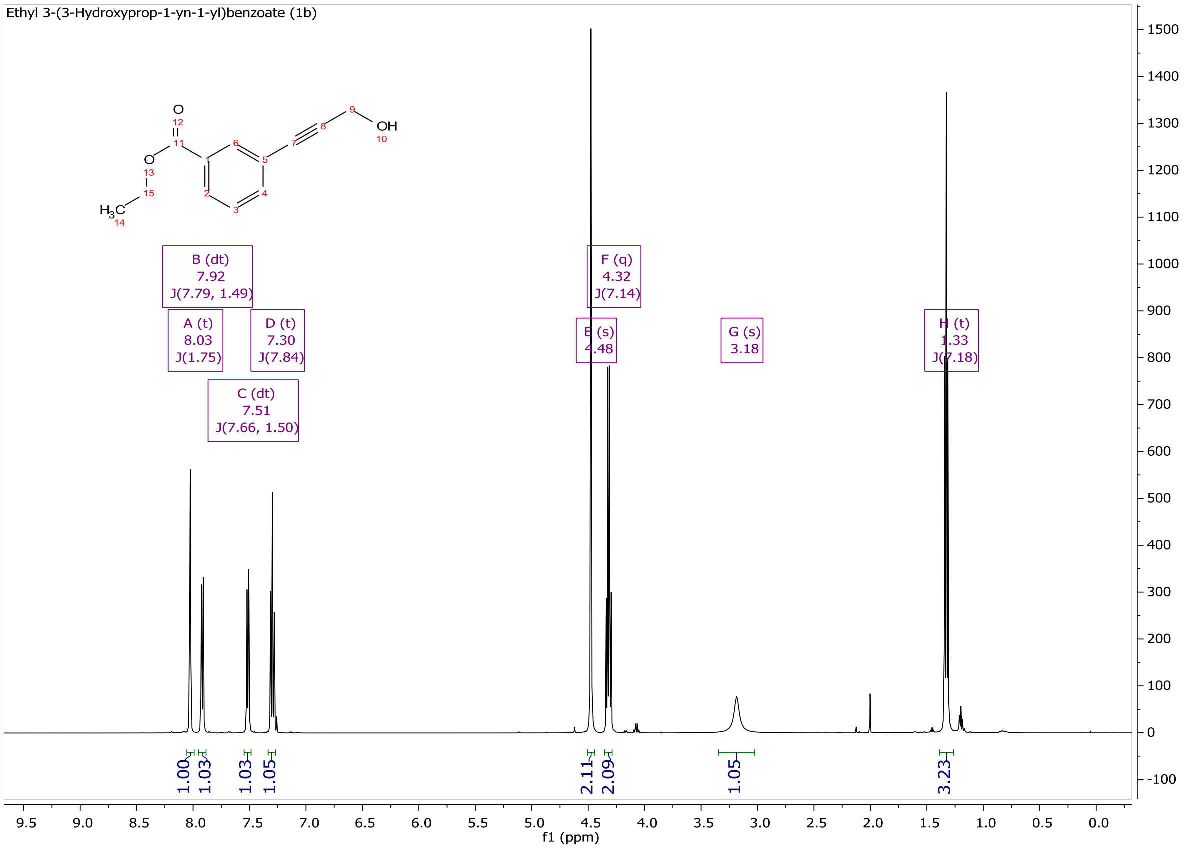

Ethyl (3-Oxopropynyl)-benzoate derivatives **2**

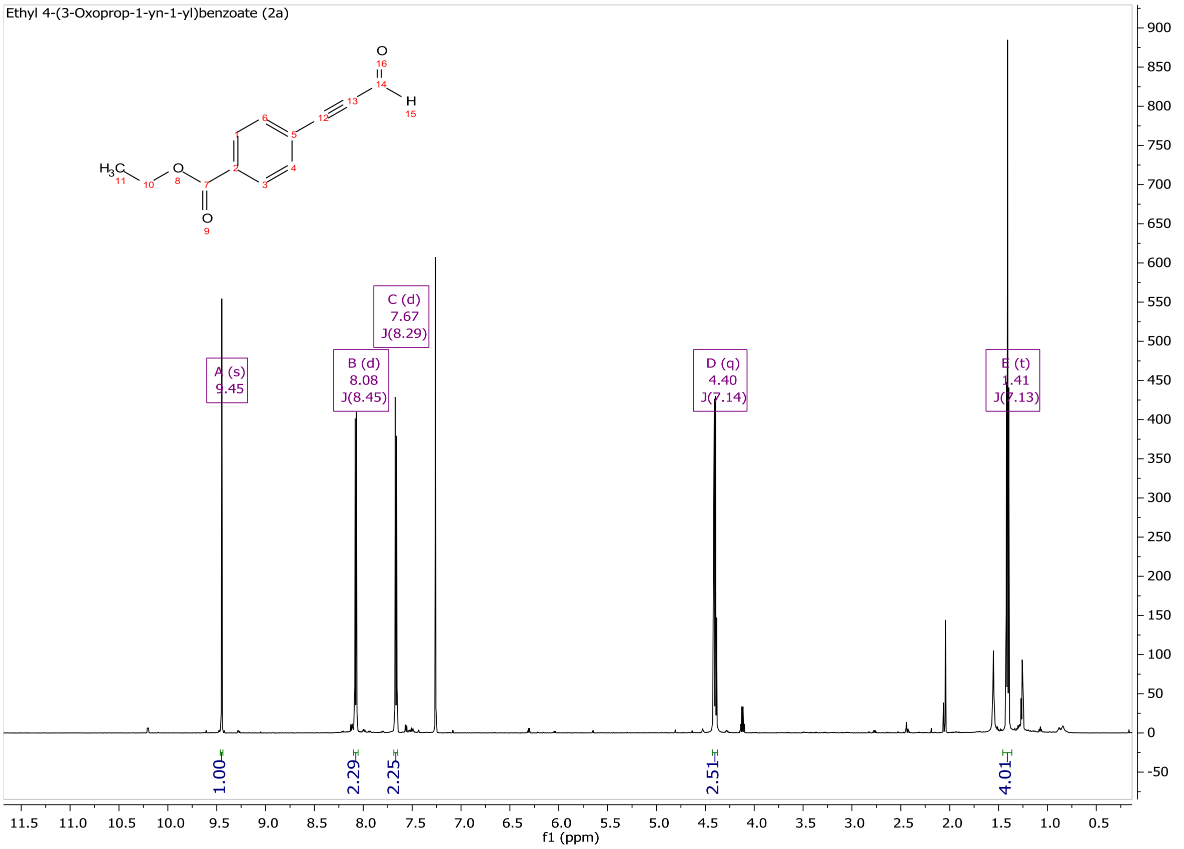

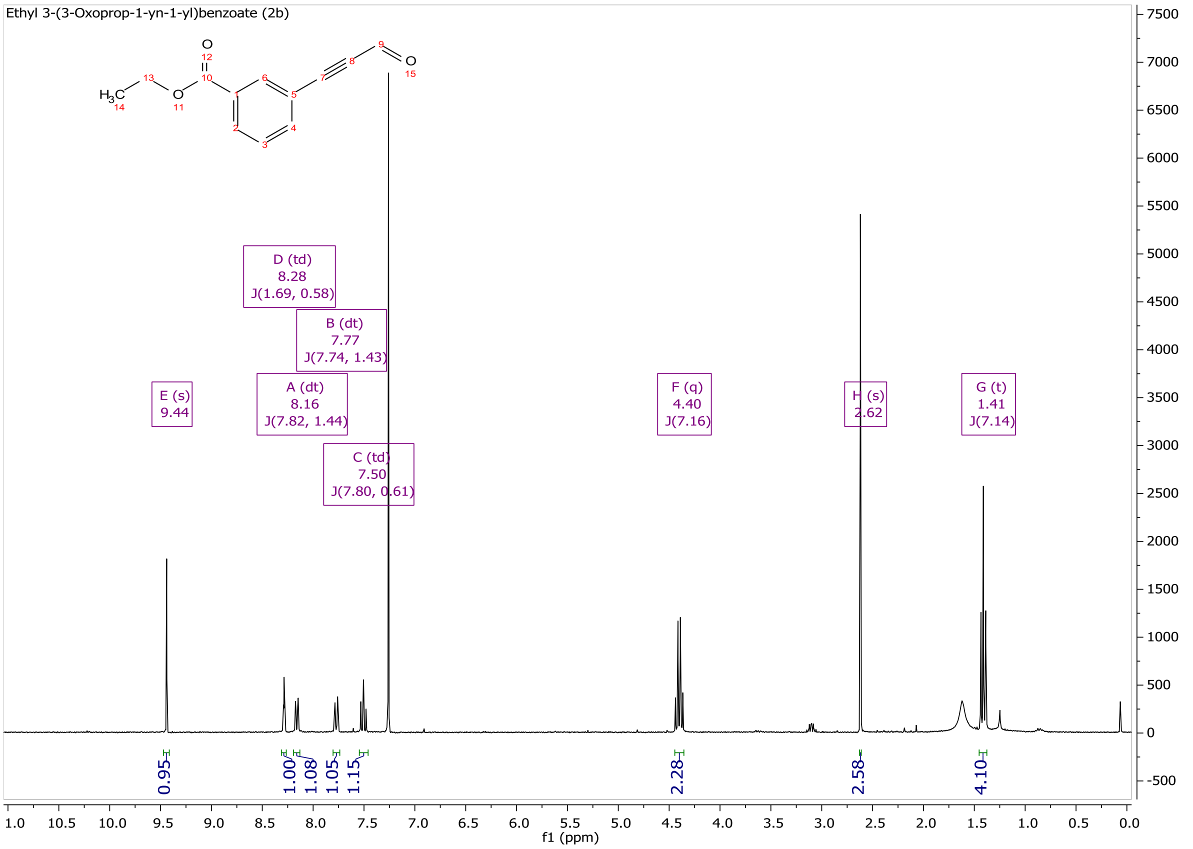

Ethyl (*tert*-Butylsulfinyl)imino)propynyl)benzoate derivatives **3**

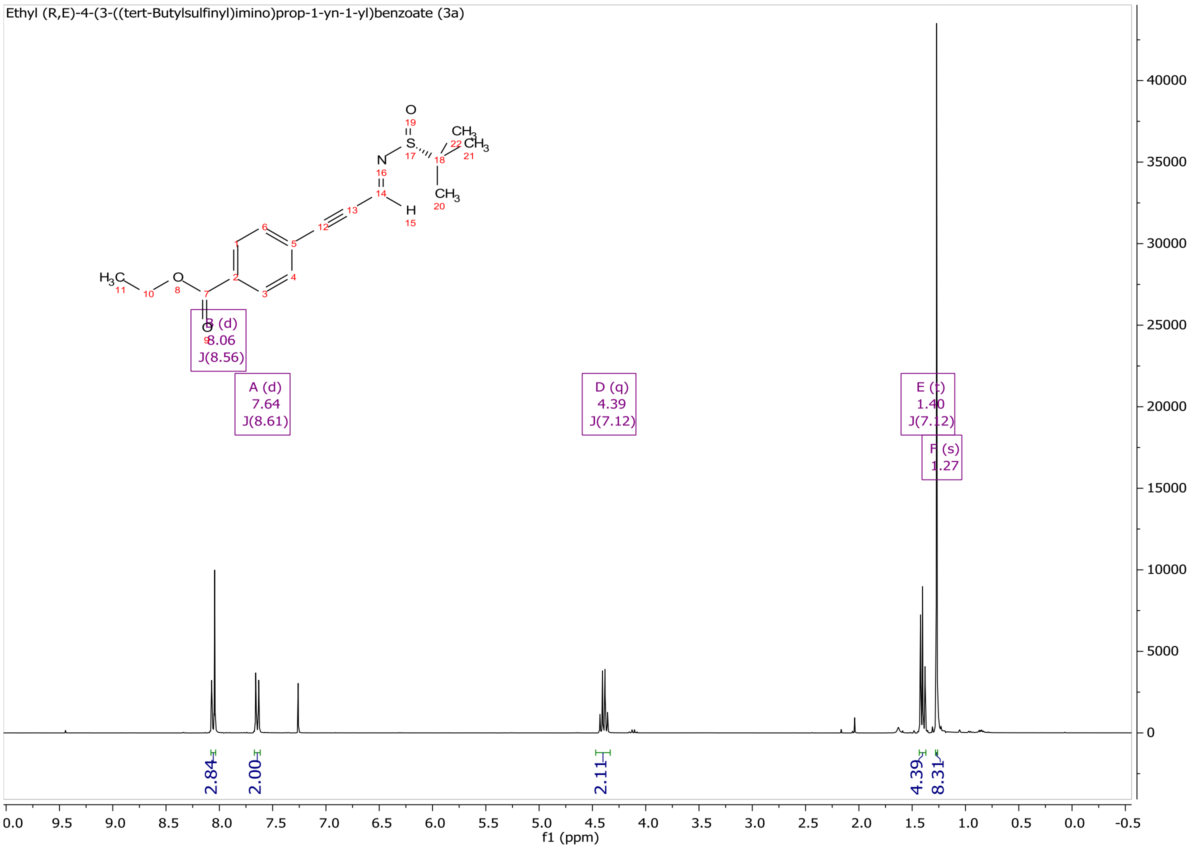

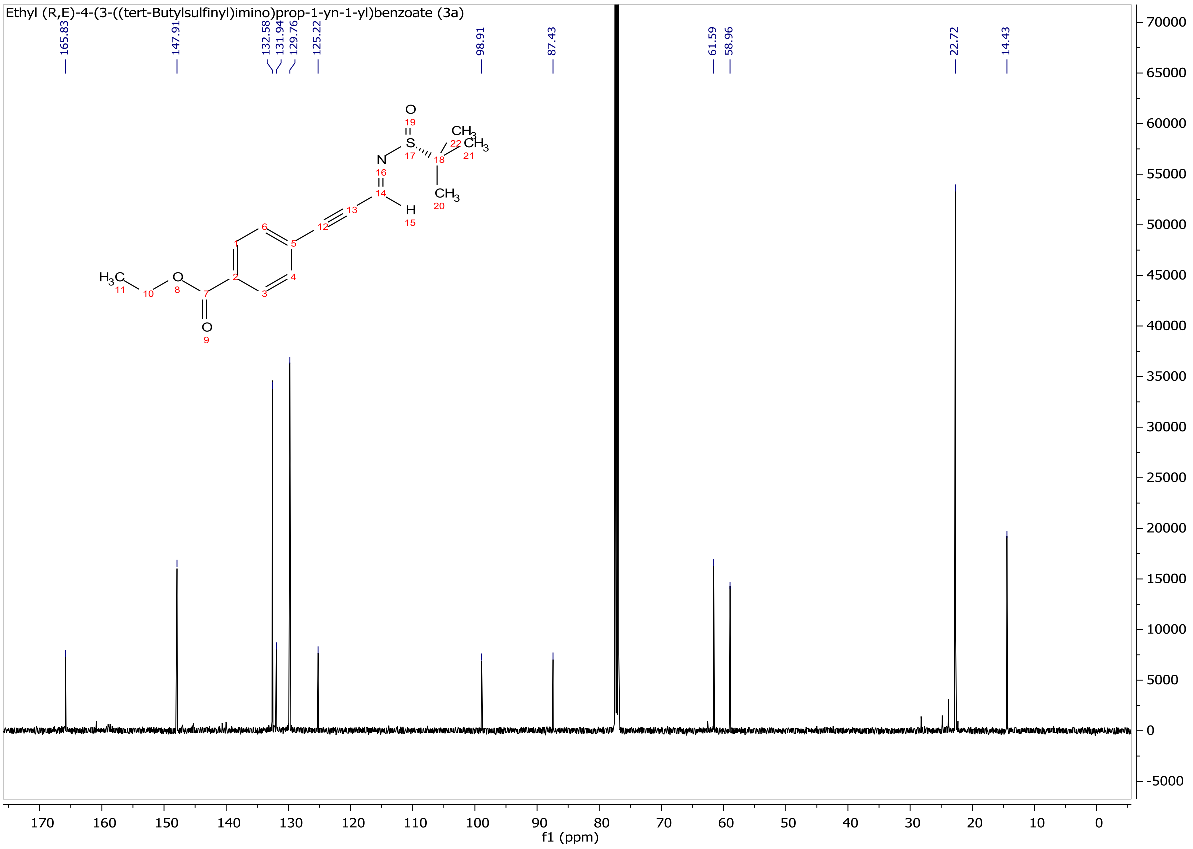
Ethyl (R,E)-4-(3-((tert-Butylsulfinyl)imino)prop-1-yn-1-yl)benzoate (3a)
165.83
147.91
132.58
131.94
129.76
125.22
98.91
87.43
61.59
58.96
22.72
14.43
f1 (ppm)

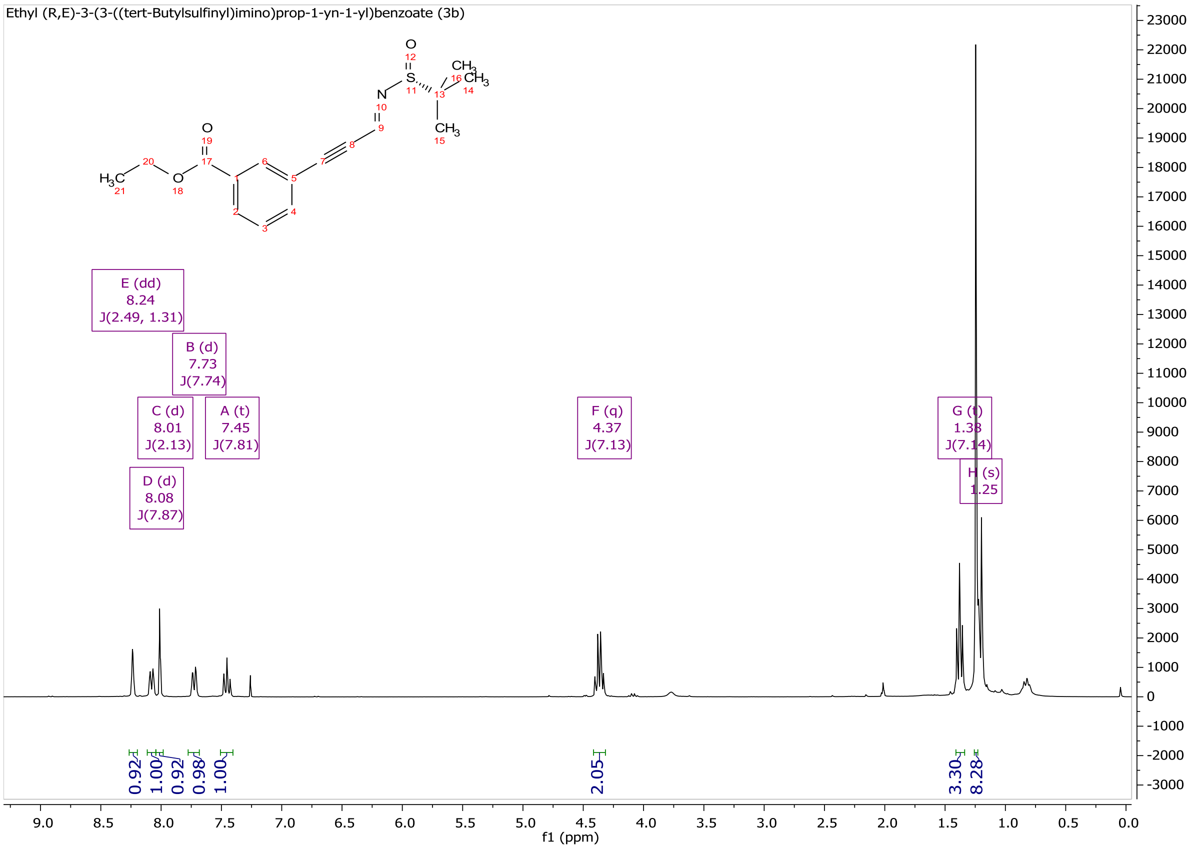
Ethyl (R,E)-3-(3-((tert-Butylsulfinyl)imino)prop-1-yn-1-yl)benzoate (3b)
E (dd) 8.24 J(2.49, 1.31)
B (d) 7.73 J(7.74)
C (d) 8.01 J(2.13)
A (t) 7.45 J(7.81)
D (d) 8.08 J(7.87)
F (q) 4.37 J(7.13)
G (t) 1.38 J(7.14)
H (s) 1.25
0.92
1.00
0.92
0.98
1.00
2.05
3.30
8.28
f1 (ppm)

Ethyl benzoate substituted propargylamine derivatives **4**

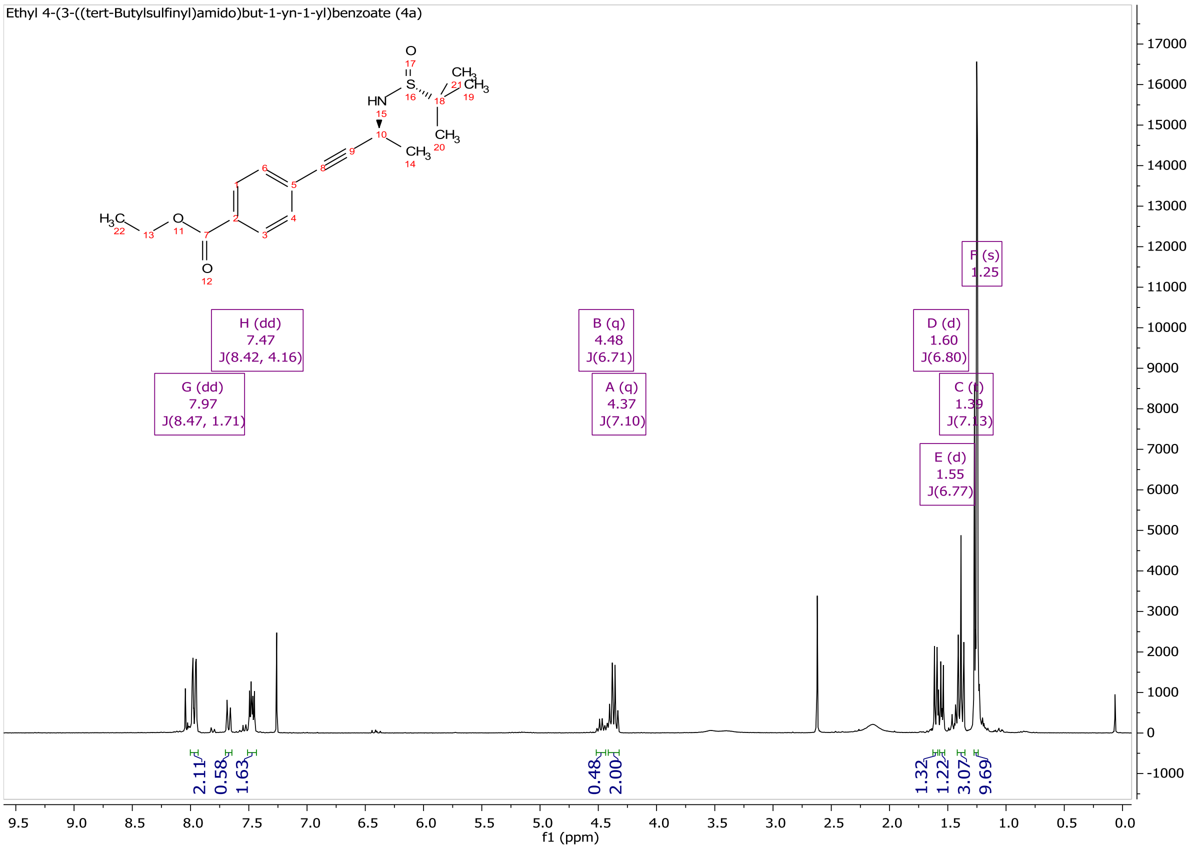

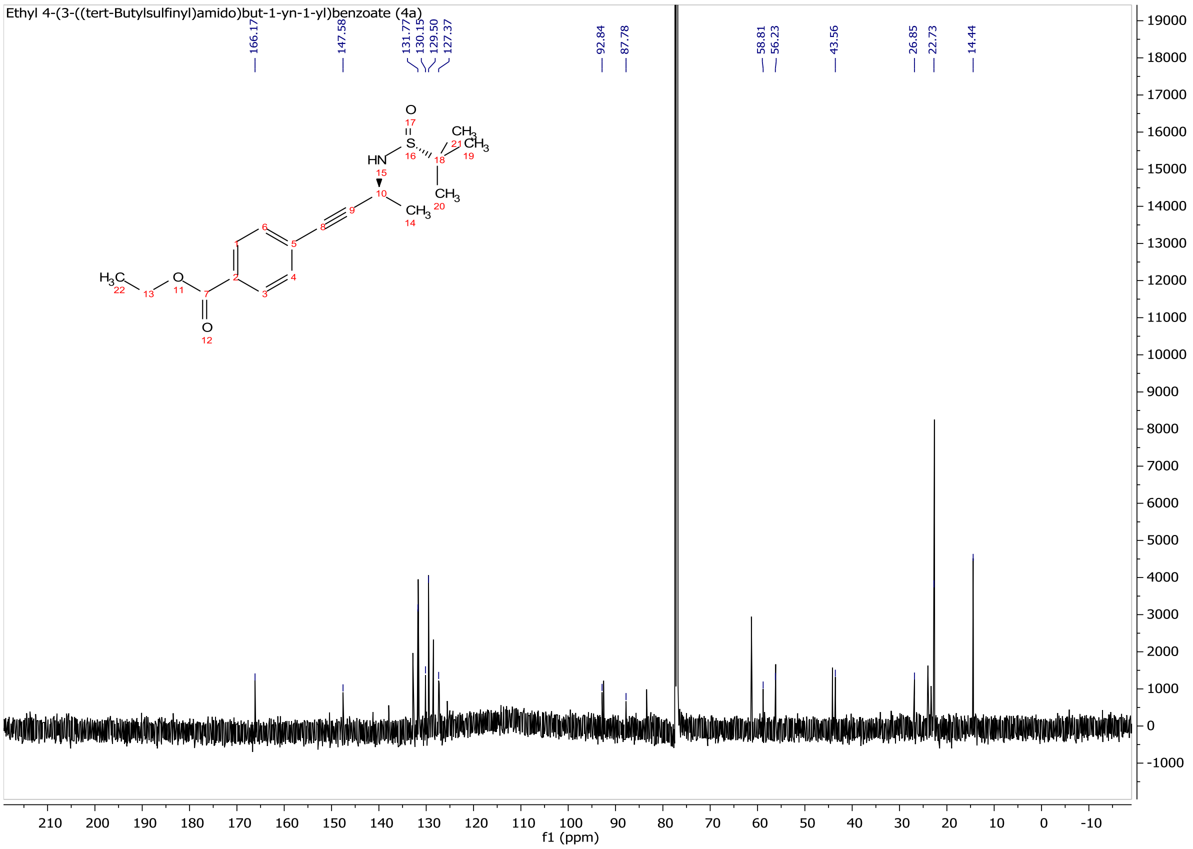

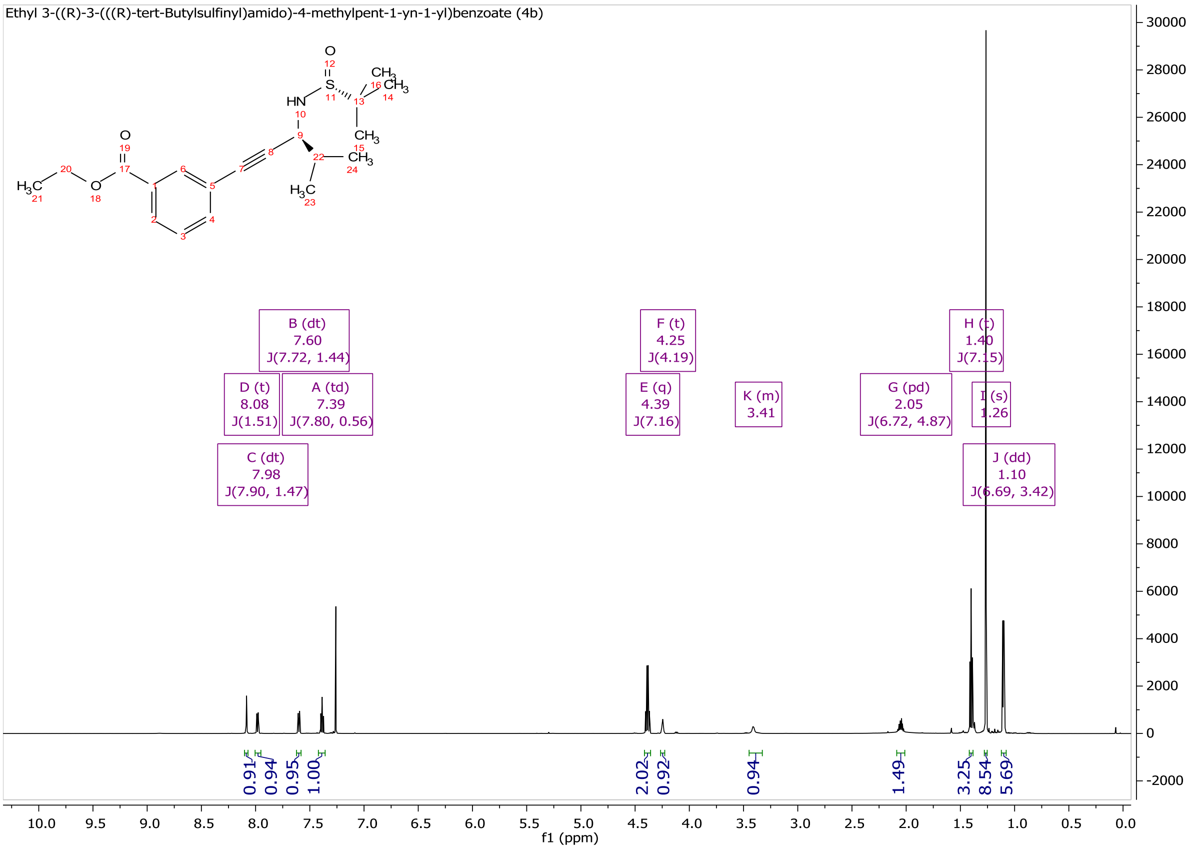
Ethyl 3-((R)-3-(((R)-tert-Butylsulfinyl)amido)-4-methylpent-1-yn-1-yl)benzoate (4b)
B (dt) 7.60 J(7.72, 1.44)
D (t) 8.08 J(1.51)
A (td) 7.39 J(7.80, 0.56)
C (dt) 7.98 J(7.90, 1.47)
F (t) 4.25 J(4.19)
E (q) 4.39 J(7.16)
K (m) 3.41
G (pd) 2.05 J(6.72, 4.87)
H (t) 1.40 J(7.15)
I (s) 1.26
J (dd) 1.10 J(6.69, 3.42)
0.91 0.94 0.95 1.00 2.02 0.92 0.94 1.49 3.25 8.54 5.69
f1 (ppm)

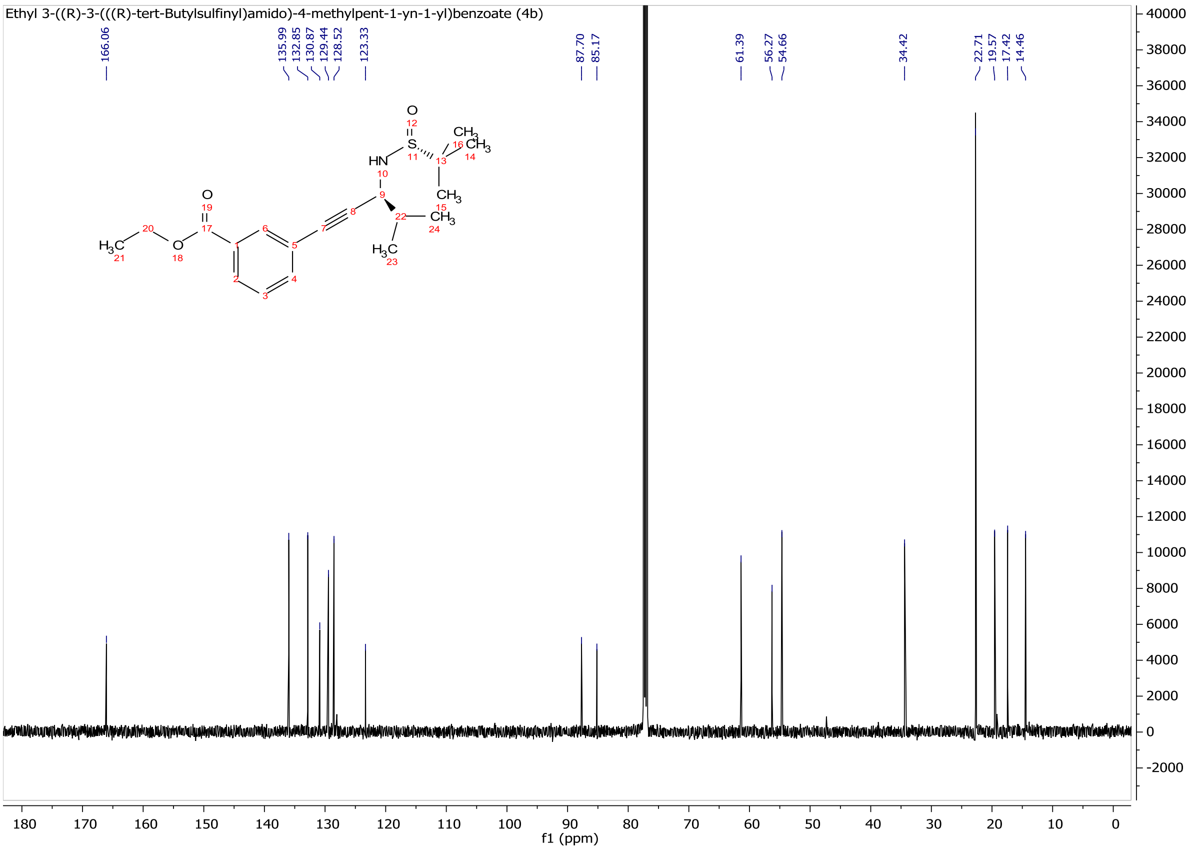
Ethyl 3-((R)-3-(((R)-tert-Butylsulfinyl)amido)-4-methylpent-1-yn-1-yl)benzoate (4b)
166.06
135.99 132.85 130.87 129.44 128.52
123.33
87.70 85.17
61.39 56.27 54.66
34.42
22.71 19.57 17.42 14.46
f1 (ppm)

Chiral aldimines **5**

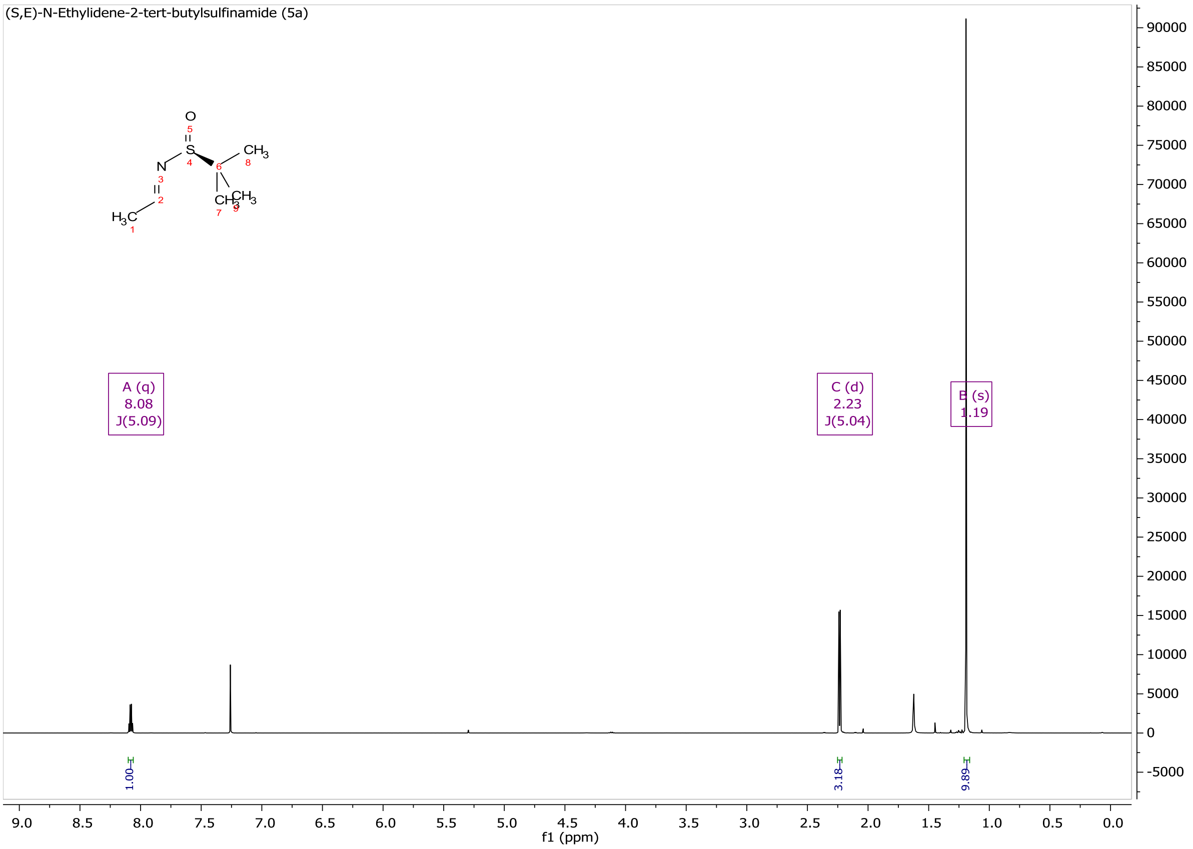

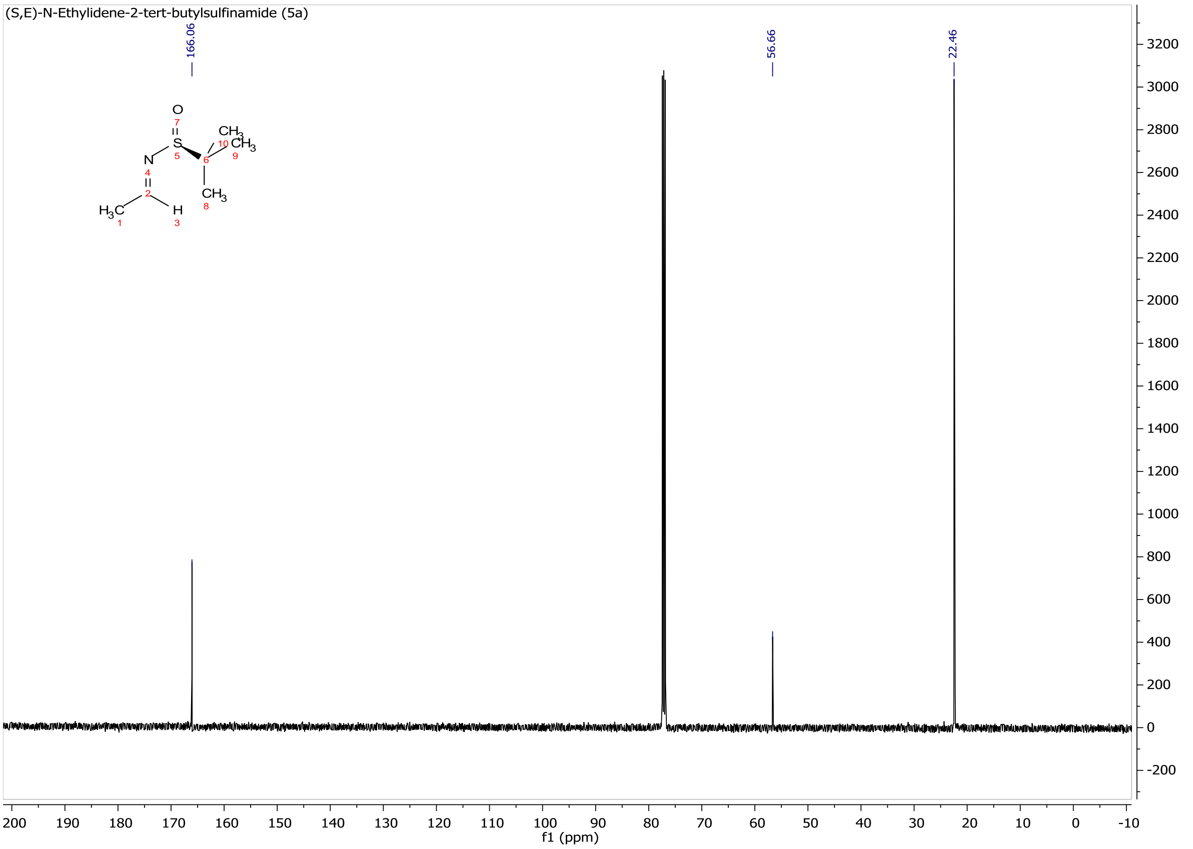

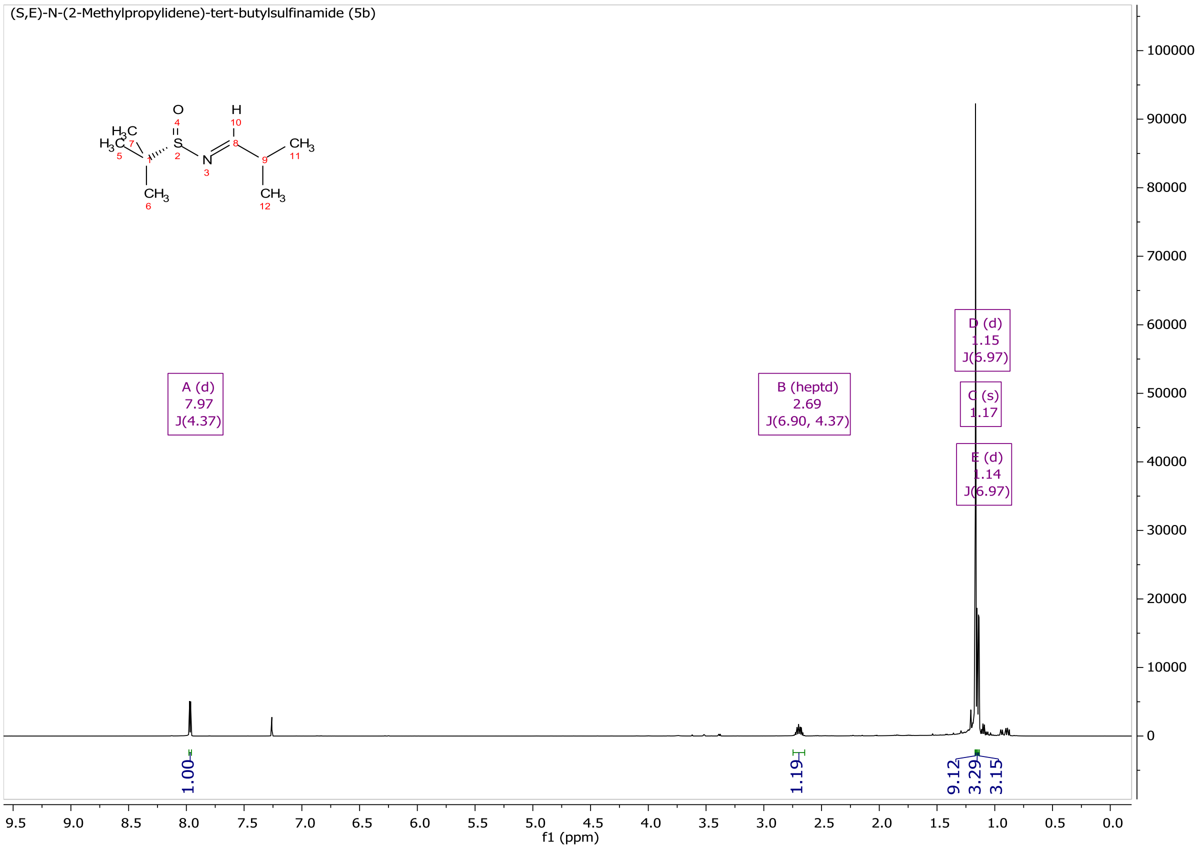
(S,E)-N-(2-Methylpropylidene)-tert-butylsulfinamide (5b)
A (d)
7.97
J(4.37)
B (heptd)
2.69
J(6.90, 4.37)
D (d)
1.15
J(5.97)
C (s)
1.17
E (d)
1.14
J(6.97)
1.00
1.19
9.12
3.29
3.15
f1 (ppm)

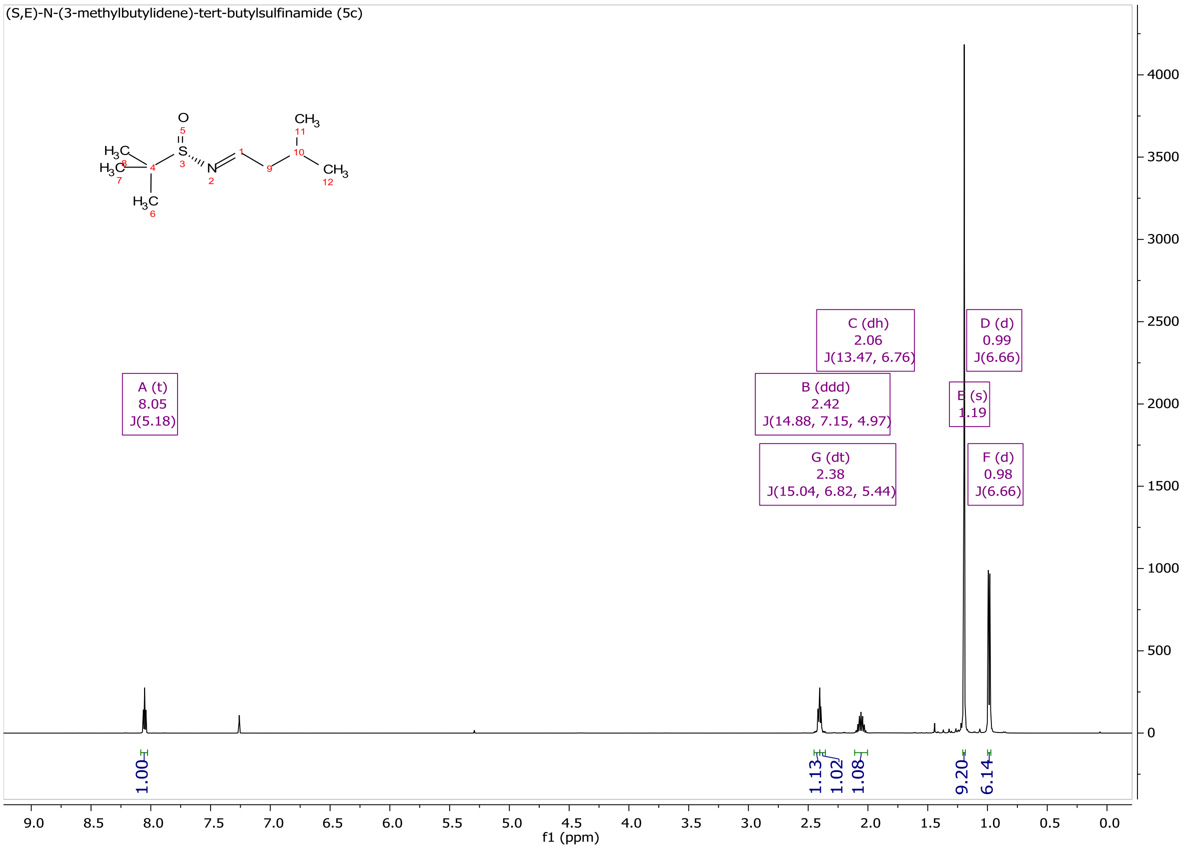
(S,E)-N-(3-methylbutylidene)-tert-butylsulfinamide (5c)
A (t)
8.05
J(5.18)
C (dh)
2.06
J(13.47, 6.76)
B (ddd)
2.42
J(14.88, 7.15, 4.97)
G (dt)
2.38
J(15.04, 6.82, 5.44)
D (d)
0.99
J(6.66)
E (s)
1.19
F (d)
0.98
J(6.66)
1.00
1.13
1.02
1.08
9.20
6.14
f1 (ppm)

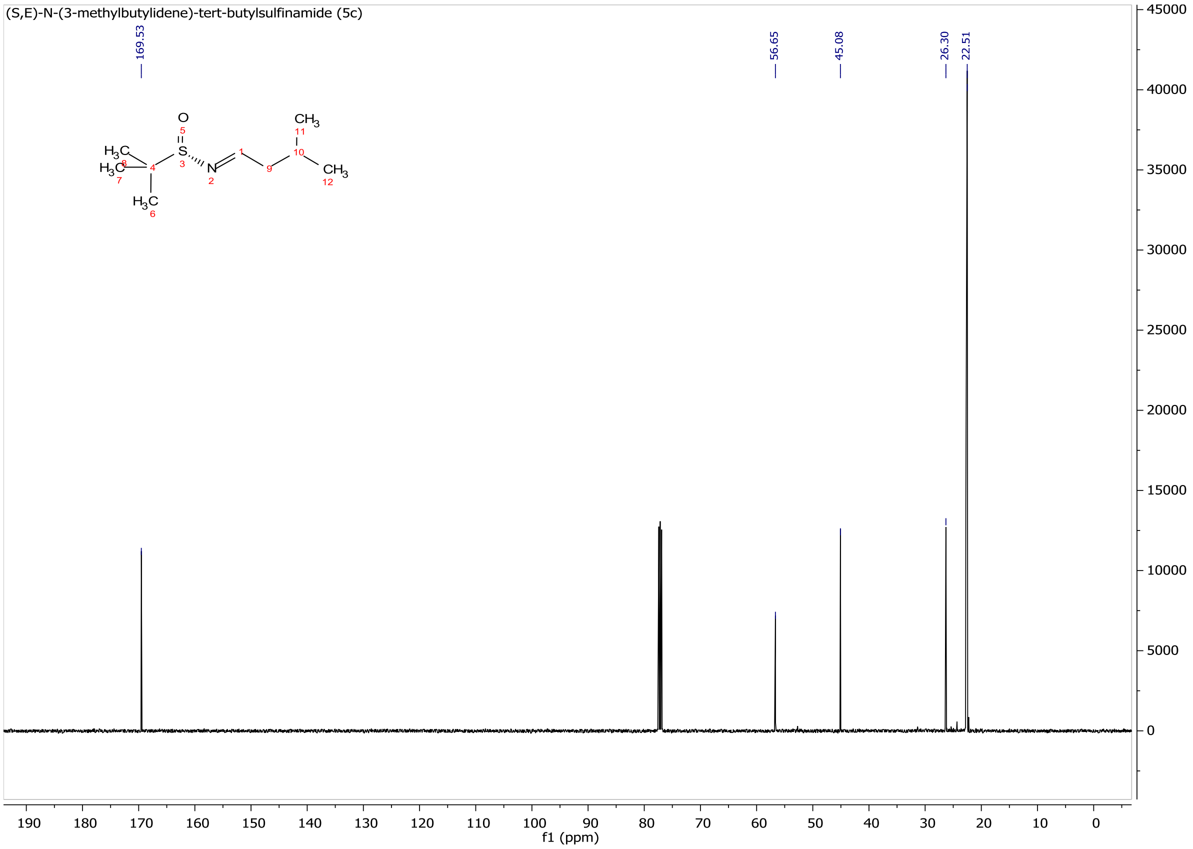
(S,E)-N-(3-methylbutylidene)-tert-butylsulfinamide (5c)
169.53
56.65
45.08
26.30
22.51
f1 (ppm)

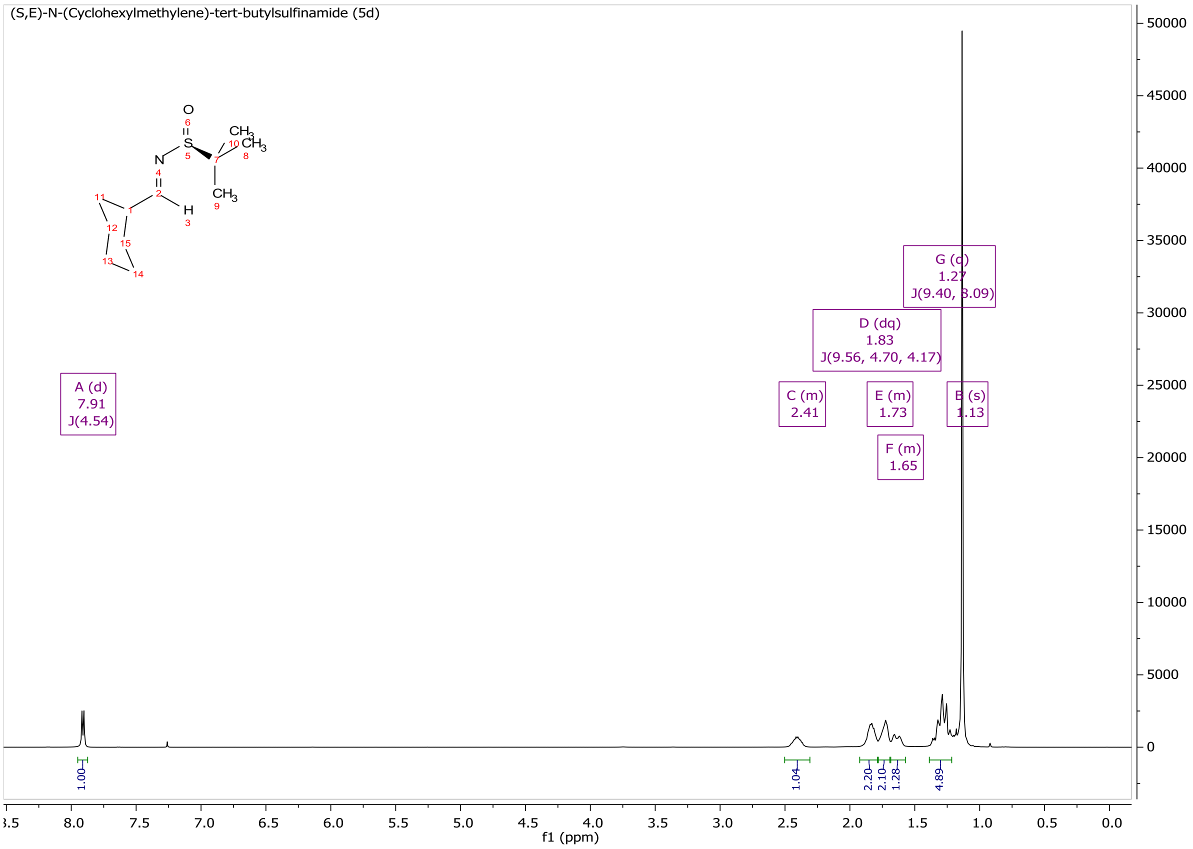
(S,E)-N-(Cyclohexylmethylene)-tert-butylsulfinamide (5d)
A (d) 7.91 J(4.54)
C (m) 2.41
D (dq) 1.83 J(9.56, 4.70, 4.17)
E (m) 1.73
F (m) 1.65
G (d) 1.27 J(9.40, 3.09)
B (s) 1.13
1.00
1.04
2.20
2.10
1.28
4.89
f1 (ppm)

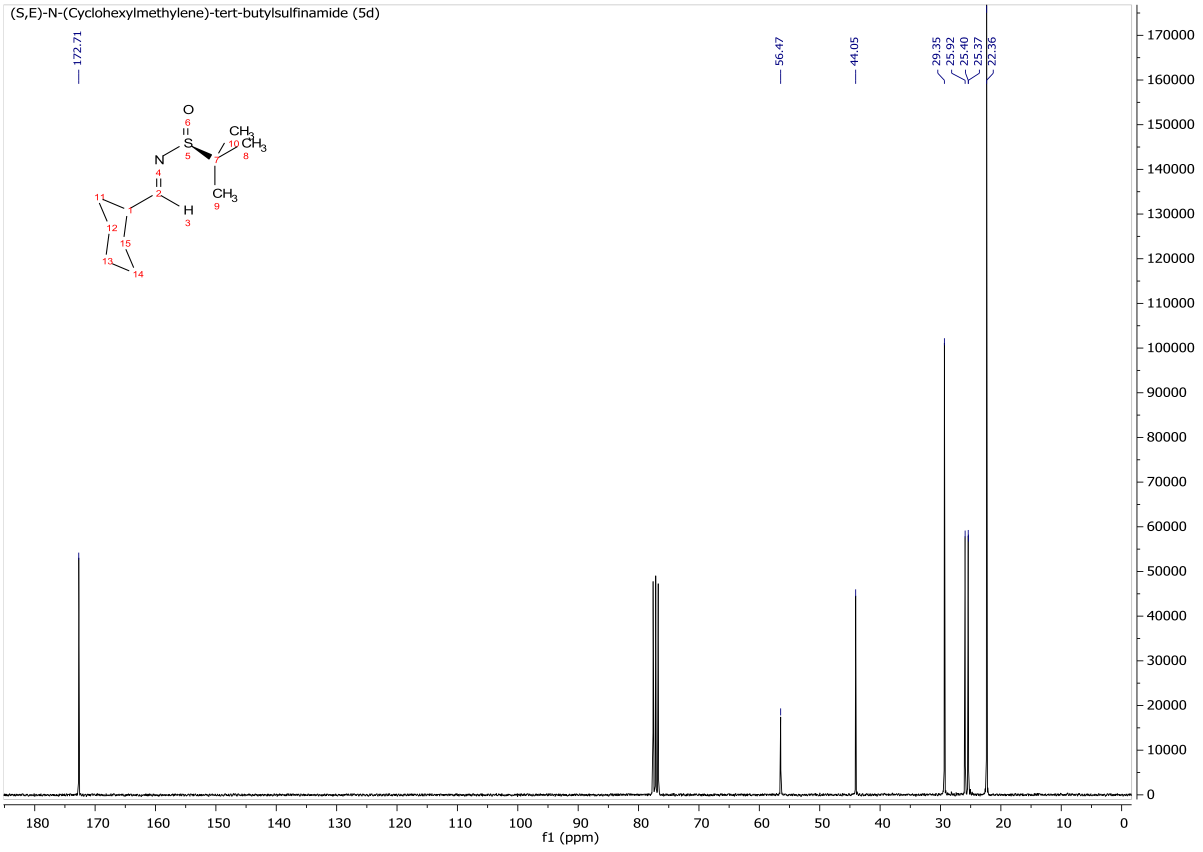
(S,E)-N-(Cyclohexylmethylene)-tert-butylsulfinamide (5d)
172.71
56.47
44.05
29.35
25.92
25.40
25.37
22.36
f1 (ppm)

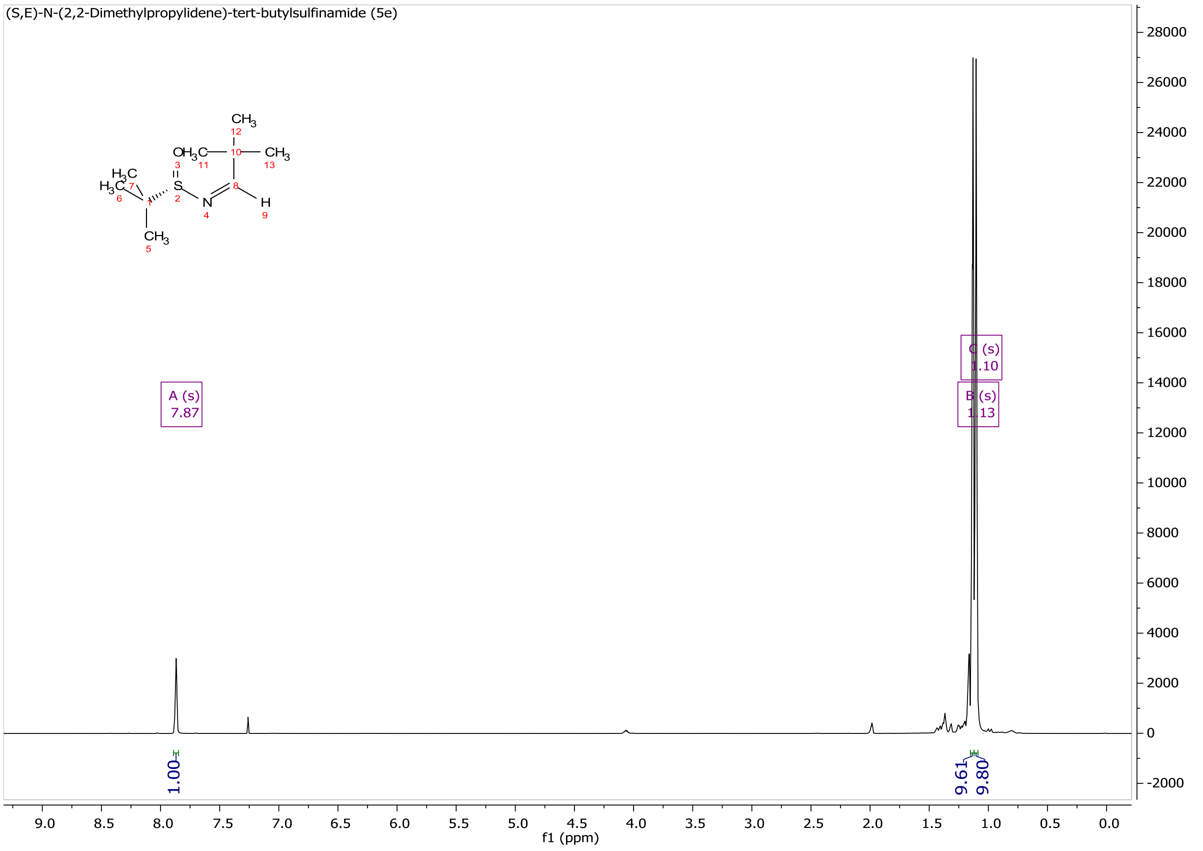
(S,E)-N-(2,2-Dimethylpropylidene)-tert-butylsulfinamide (5e)
A (s)
7.87
B (s)
1.13
C (s)
1.10
1.00
9.61
9.80
f1 (ppm)

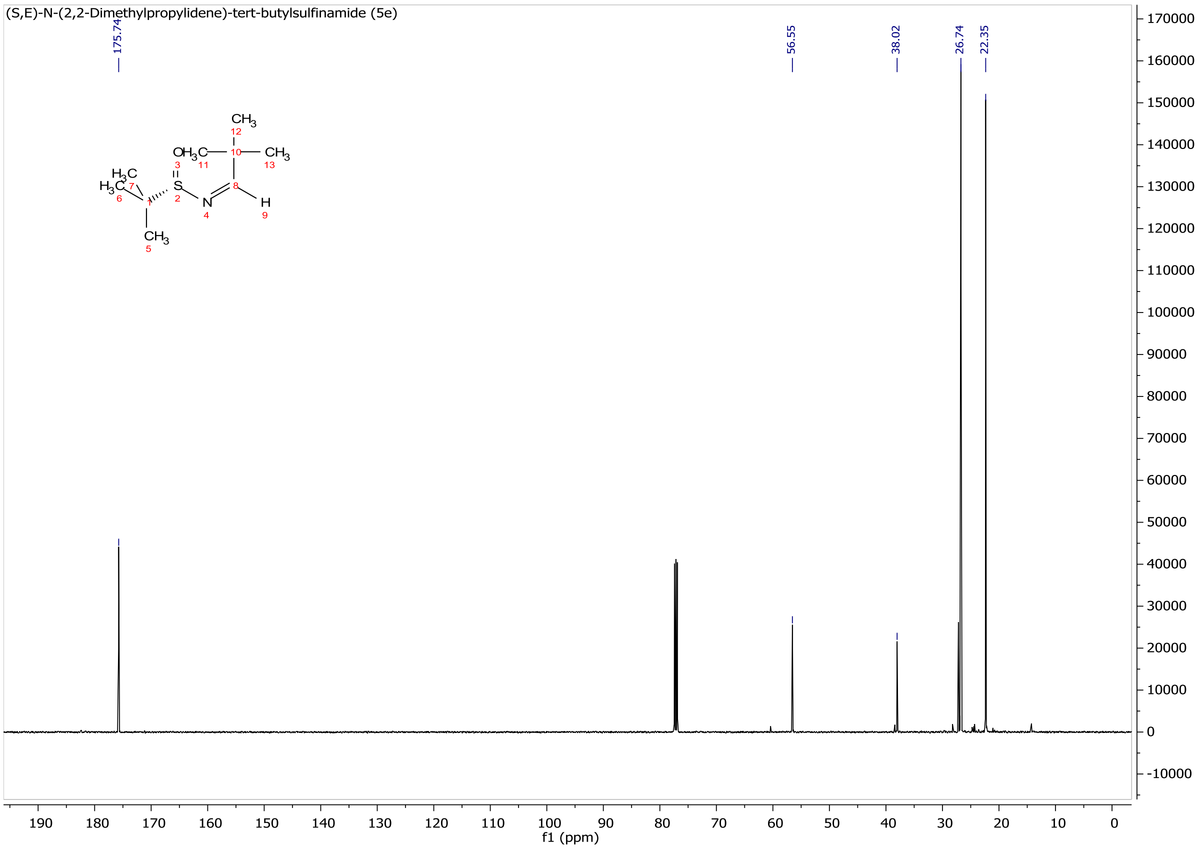
(S,E)-N-(2,2-Dimethylpropylidene)-tert-butylsulfinamide (5e)
175.74
56.55
38.02
26.74
22.35
f1 (ppm)

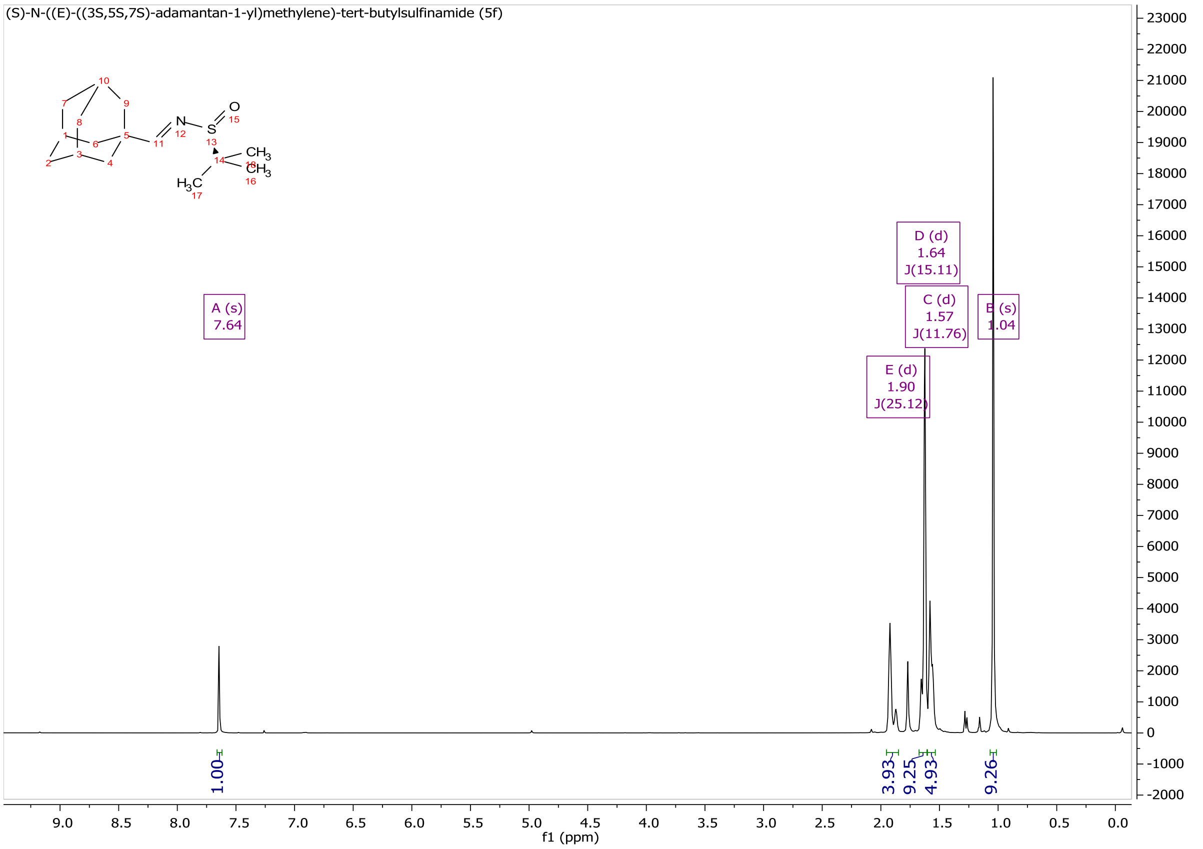
(S)-N-((E)-((3S,5S,7S)-adamantan-1-yl)methylene)-tert-butylsulfinamide (5f)
A (s)
7.64
D (d)
1.64
J(15.11)
C (d)
1.57
J(11.76)
B (s)
1.04
E (d)
1.90
J(25.12)
1.00
3.93
9.25
4.93
9.26
f1 (ppm)

(S)-N-((E)-((3S,5S,7S)-adamantan-1-yl)methylene)-tert-butylsulfinamide (5f)

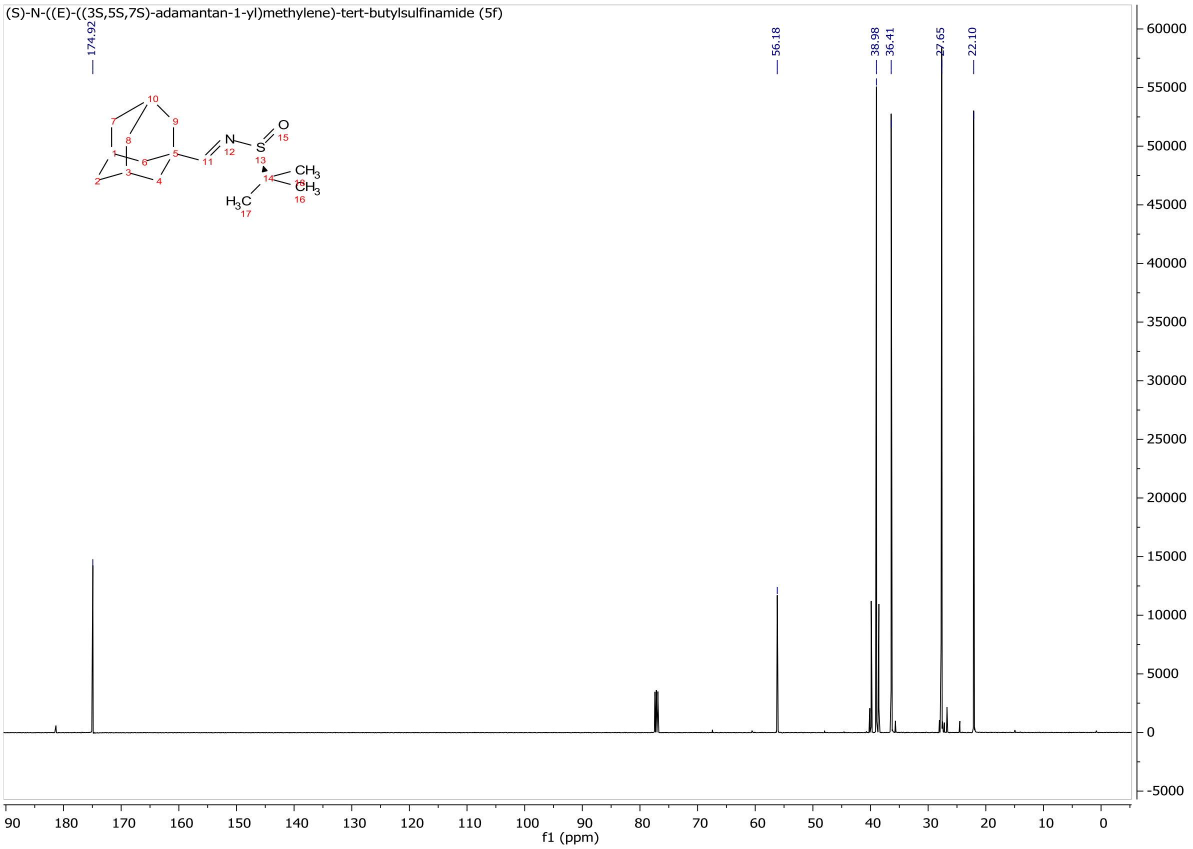

(R,E)-N-(3-(methylthio)propylidene)-tert-butylsulfinamide (5g)

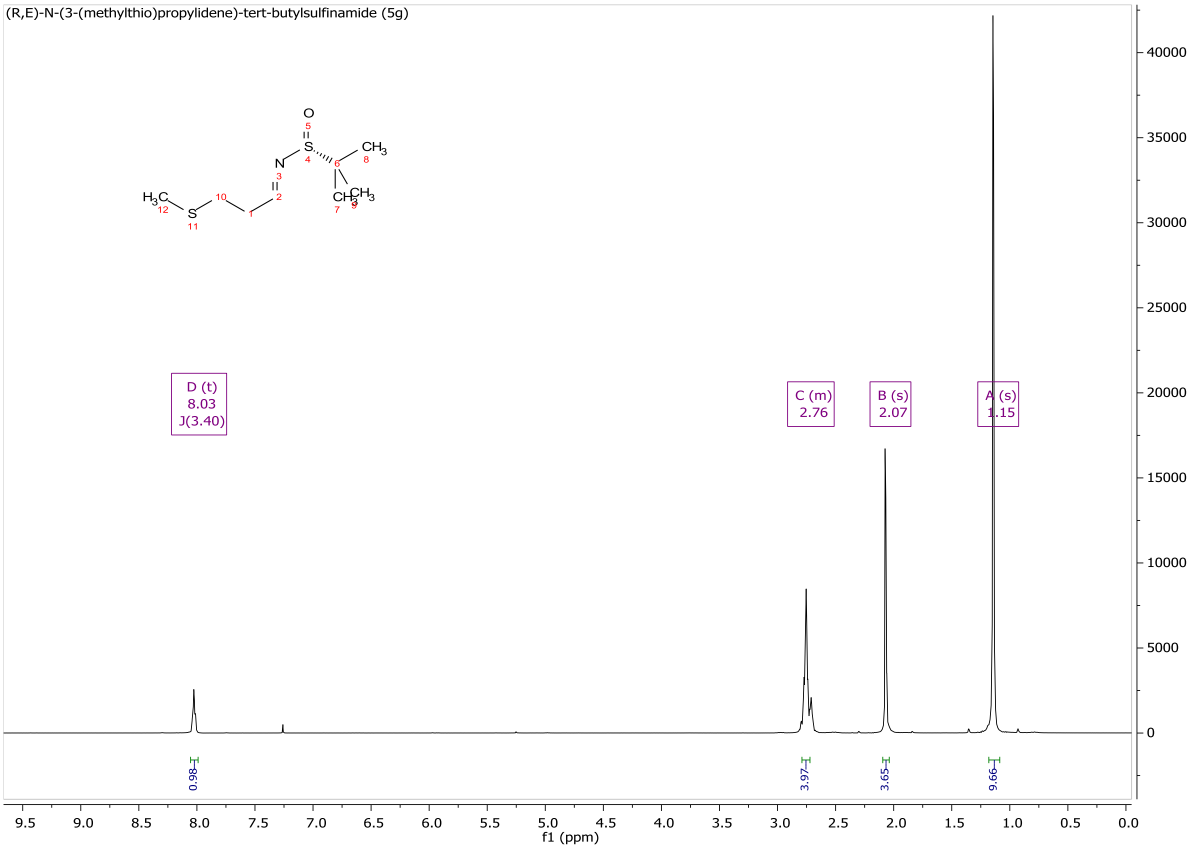

(R,E)-N-(3-(methylthio)propylidene)-tert-butylsulfinamide (5g)

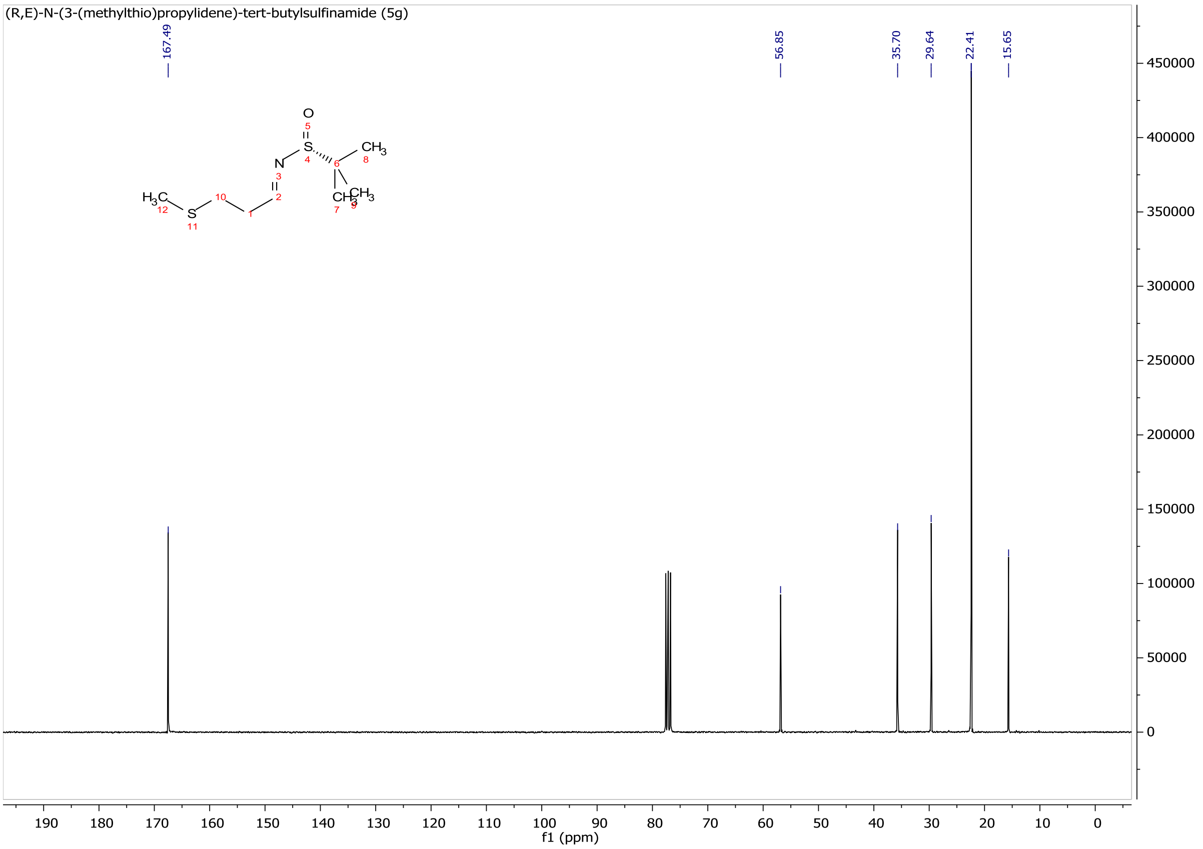

(S)-N-(2-Phenylethylidene)-tert-butylsulfinamide (5h)

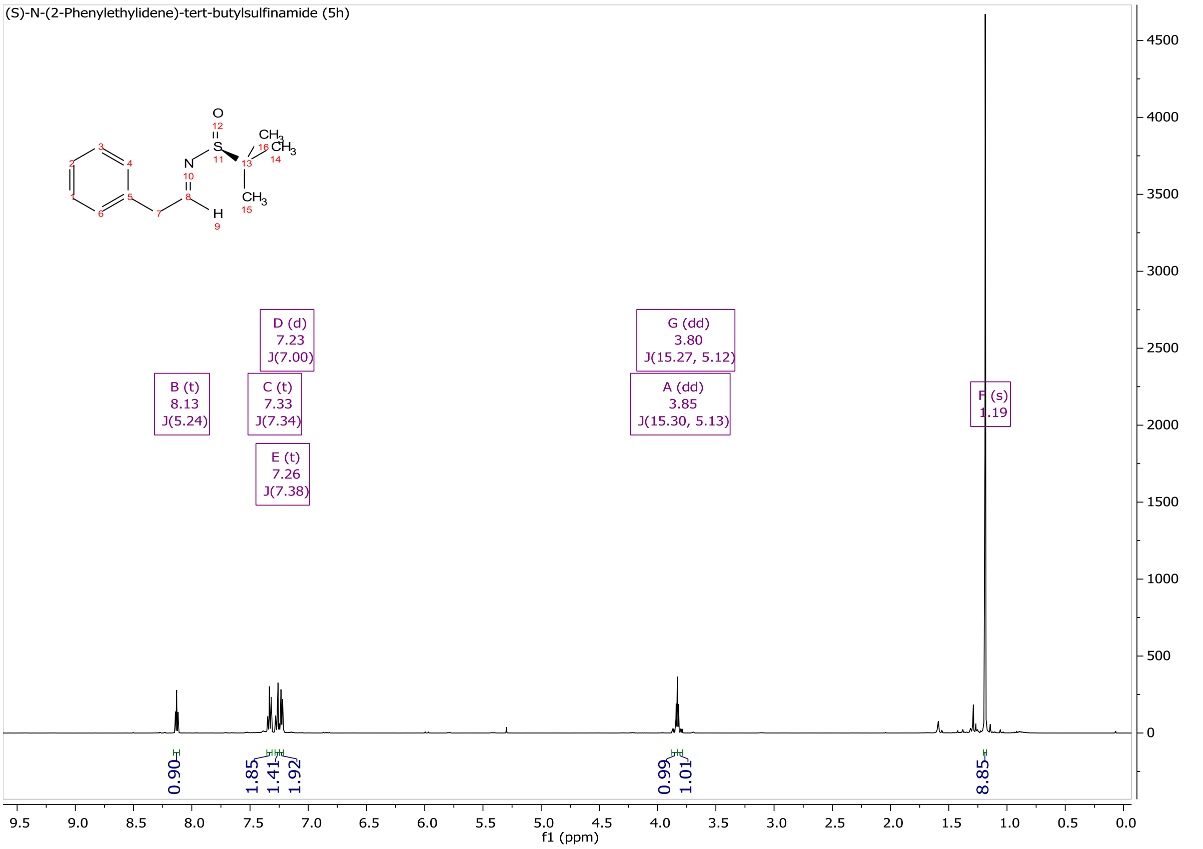

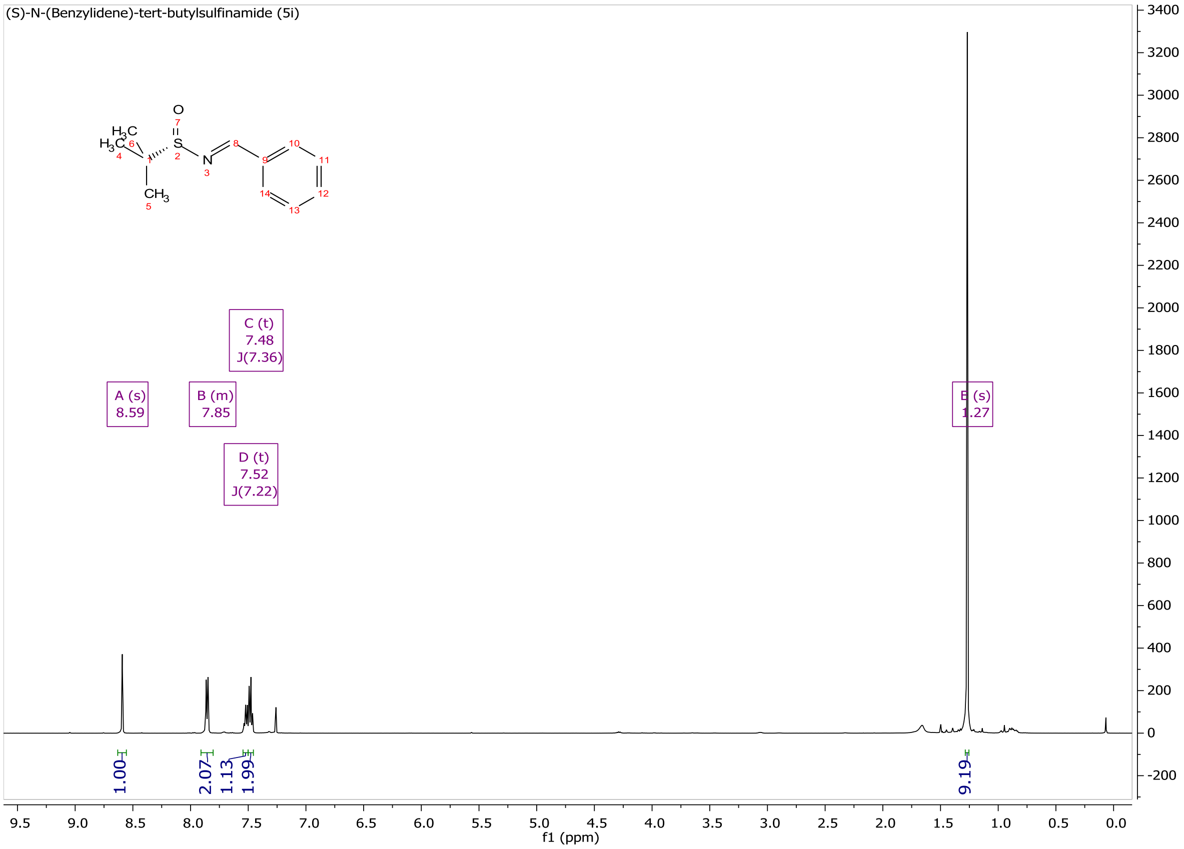
(S)-N-(Benzylidene)-tert-butylsulfinamide (5i)
A (s)
8.59
B (m)
7.85
C (t)
7.48
J(7.36)
D (t)
7.52
J(7.22)
E (s)
1.27
1.00
2.07
1.13
1.99
9.19
f1 (ppm)

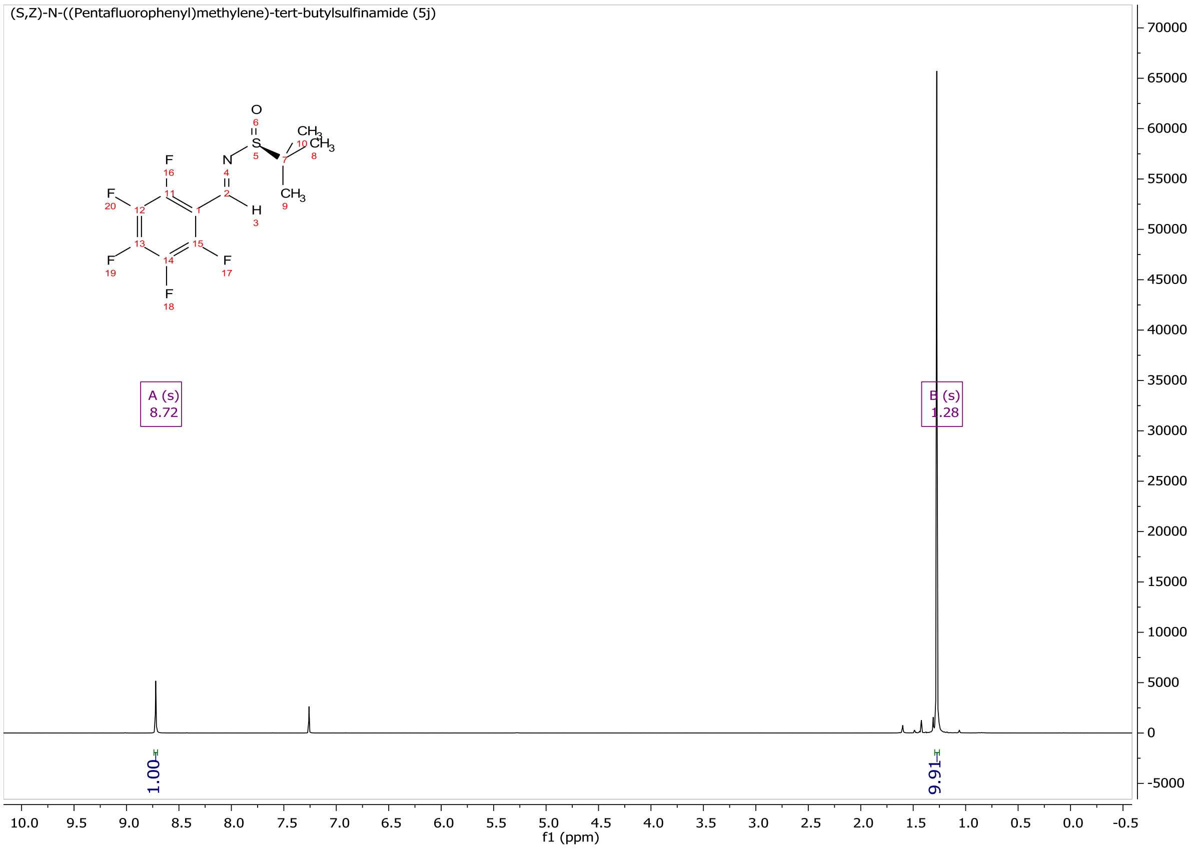
(S,Z)-N-((Pentafluorophenyl)methylene)-tert-butylsulfinamide (5j)
A (s)
8.72
B (s)
1.28
1.00
9.91
f1 (ppm)

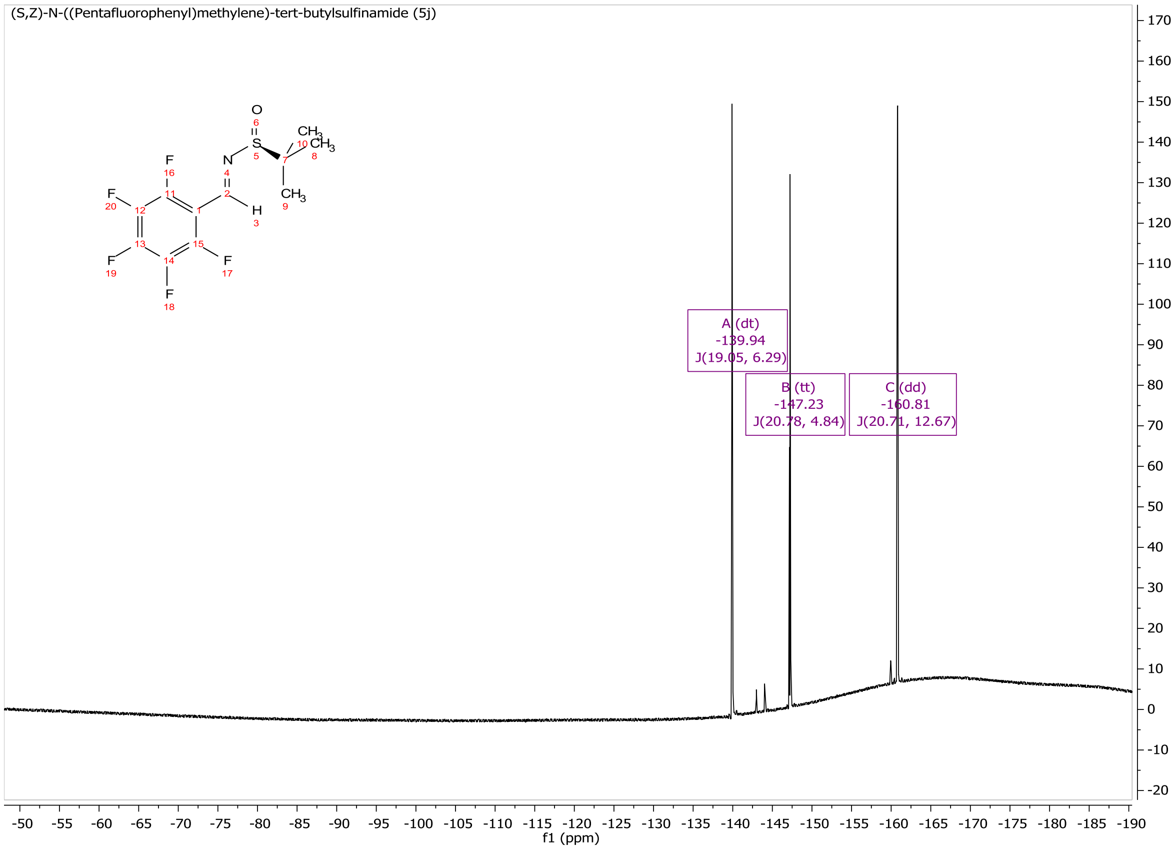

(S,Z)-N-((Pentafluorophenyl)methylene)-tert-butylsulfinamide (5j)
A (dt)
-139.94
J(19.05, 6.29)
B (tt)
-147.23
J(20.78, 4.84)
C (dd)
-160.81
J(20.71, 12.67)
f1 (ppm)

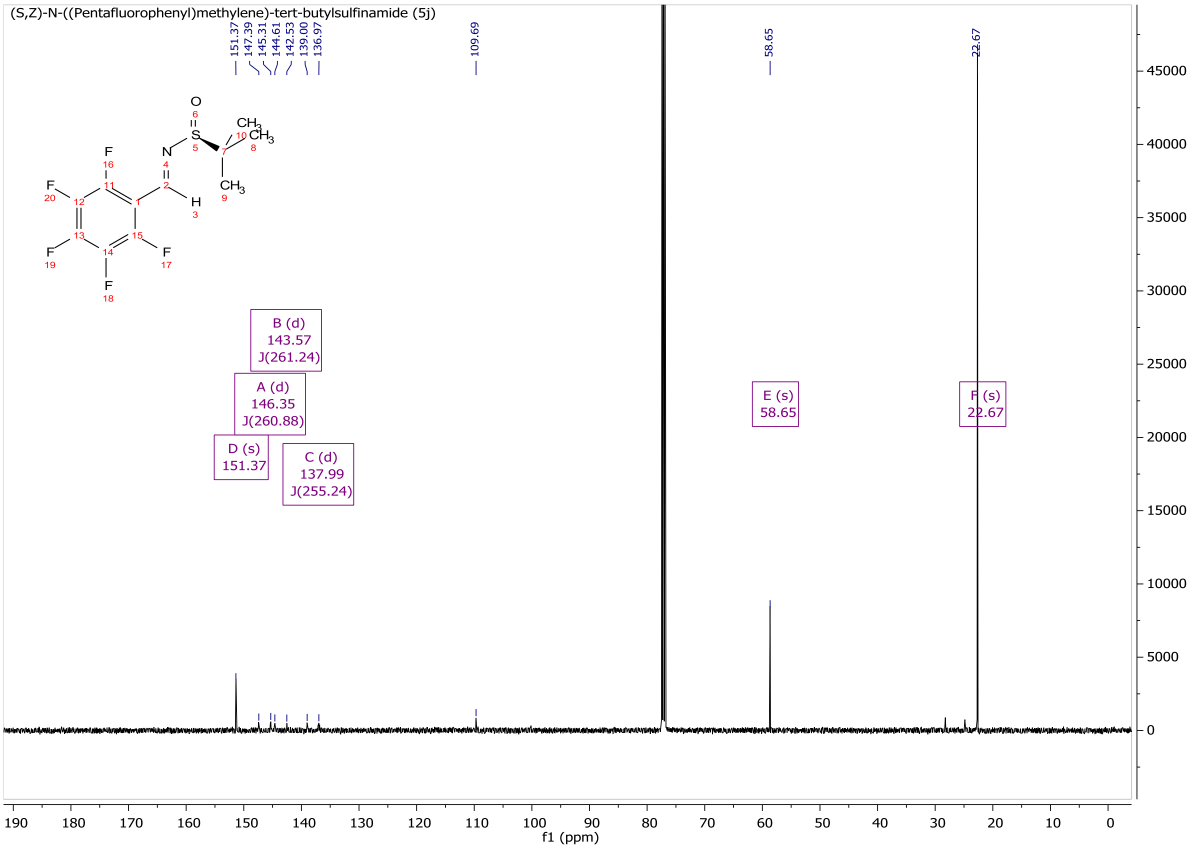

(S,Z)-N-((Pentafluorophenyl)methylene)-tert-butylsulfinamide (5j)
151.37
147.39
145.31
144.61
142.53
139.00
136.97
109.69
58.65
22.67
B (d)
143.57
J(261.24)
A (d)
146.35
J(260.88)
D (s)
151.37
C (d)
137.99
J(255.24)
E (s)
58.65
F (s)
22.67
f1 (ppm)

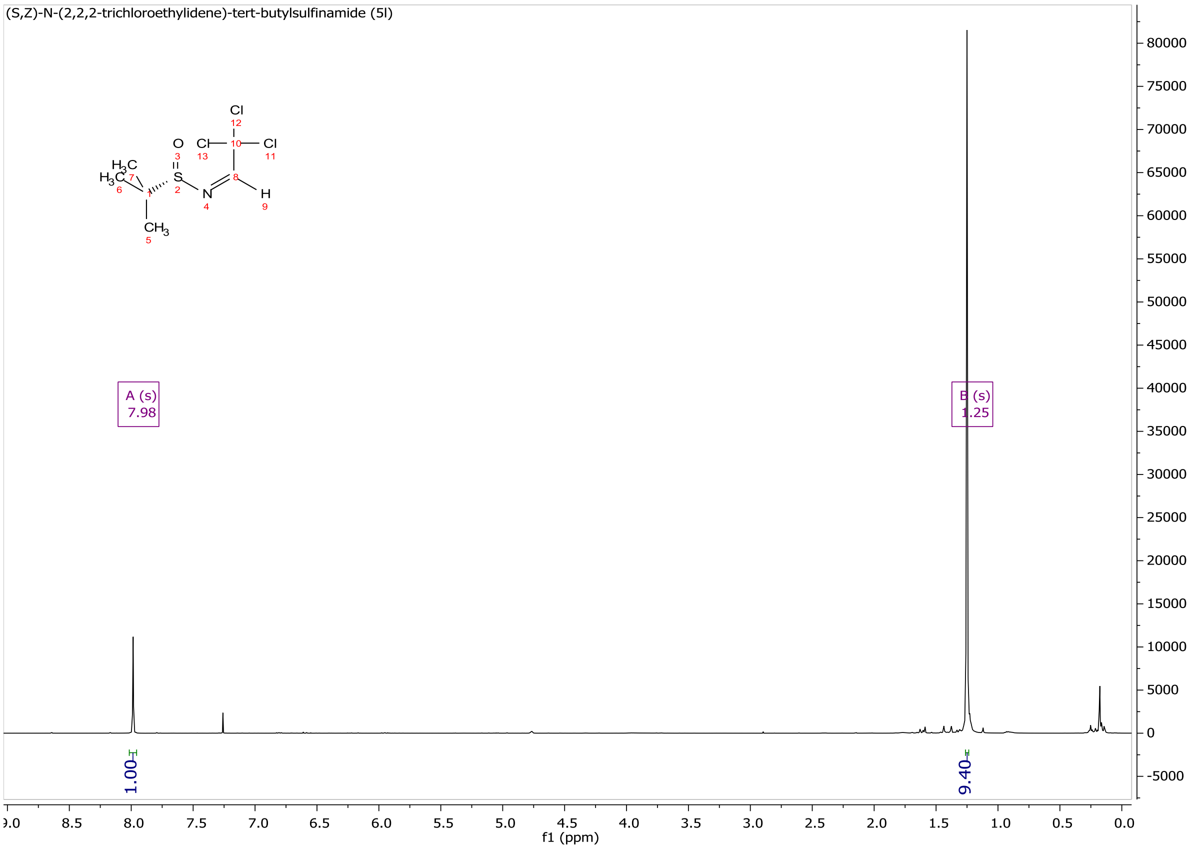

(S,Z)-N-(2,2,2-trichloroethylidene)-tert-butylsulfinamide (5l)
A (s)
7.98
B (s)
1.25
1.00
9.40
f1 (ppm)

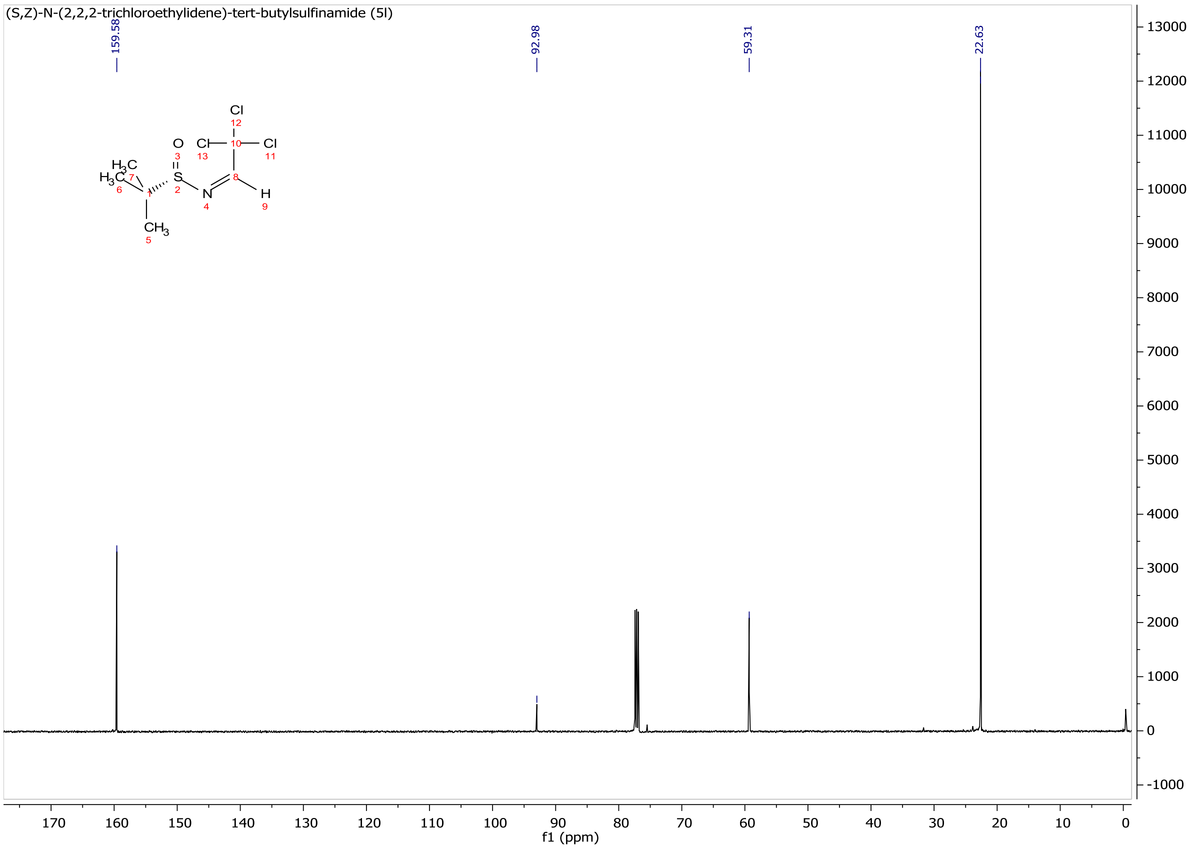

(S,Z)-N-(2,2,2-trichloroethylidene)-tert-butylsulfinamide (5l)
159.58
92.98
59.31
22.63
f1 (ppm)

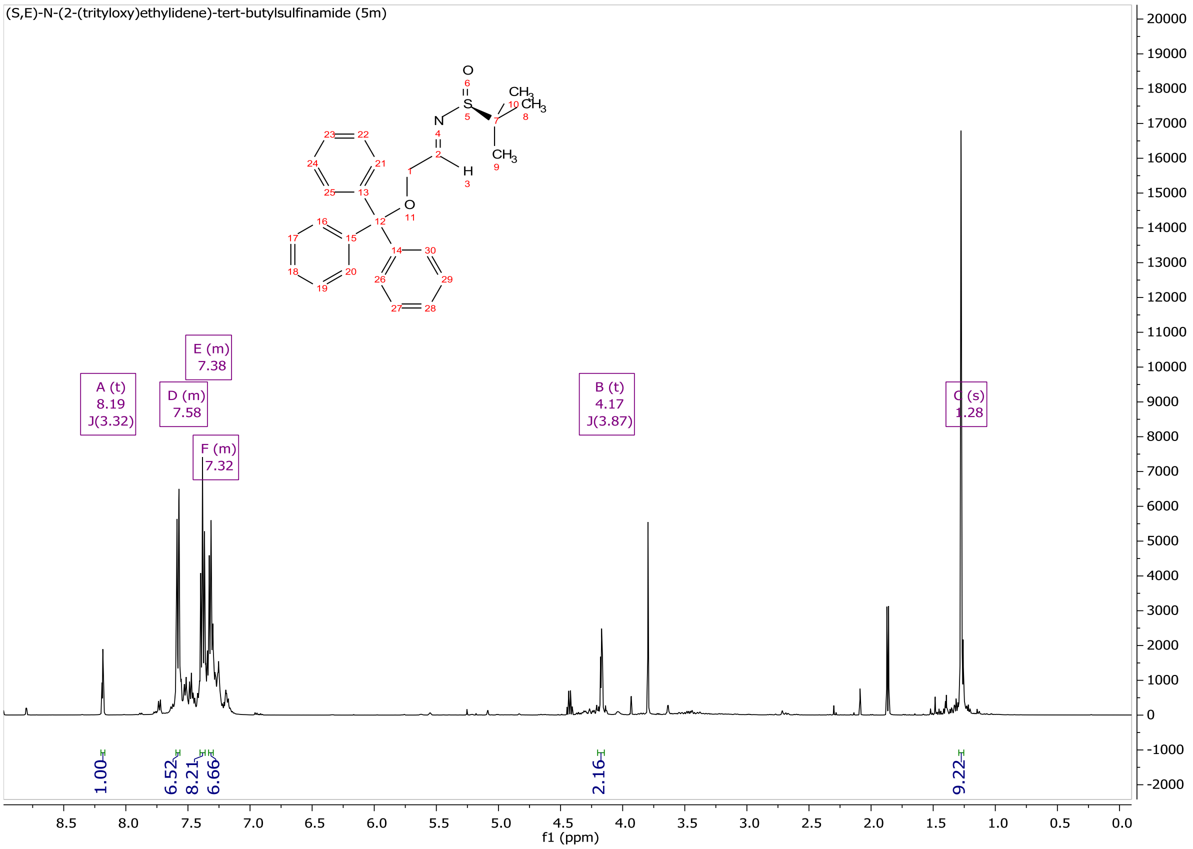
(S,E)-N-(2-(trityloxy)ethylidene)-tert-butylsulfinamide (5m)
A (t)
8.19
J(3.32)
D (m)
7.58
E (m)
7.38
F (m)
7.32
B (t)
4.17
J(3.87)
C (s)
1.28
1.00
6.52
8.21
6.66
2.16
9.22
f1 (ppm)

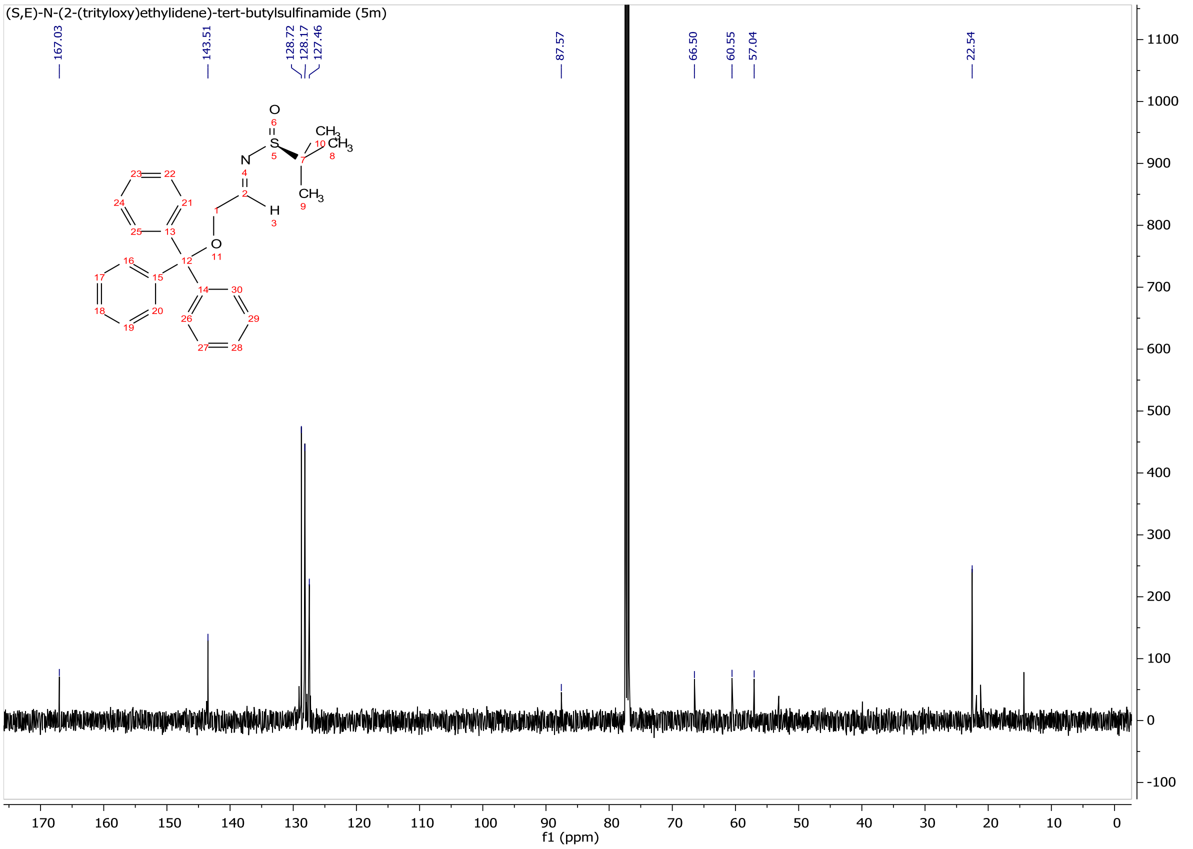
(S,E)-N-(2-(trityloxy)ethylidene)-tert-butylsulfinamide (5m)
167.03
143.51
128.72
128.17
127.46
87.57
66.50
60.55
57.04
22.54
f1 (ppm)

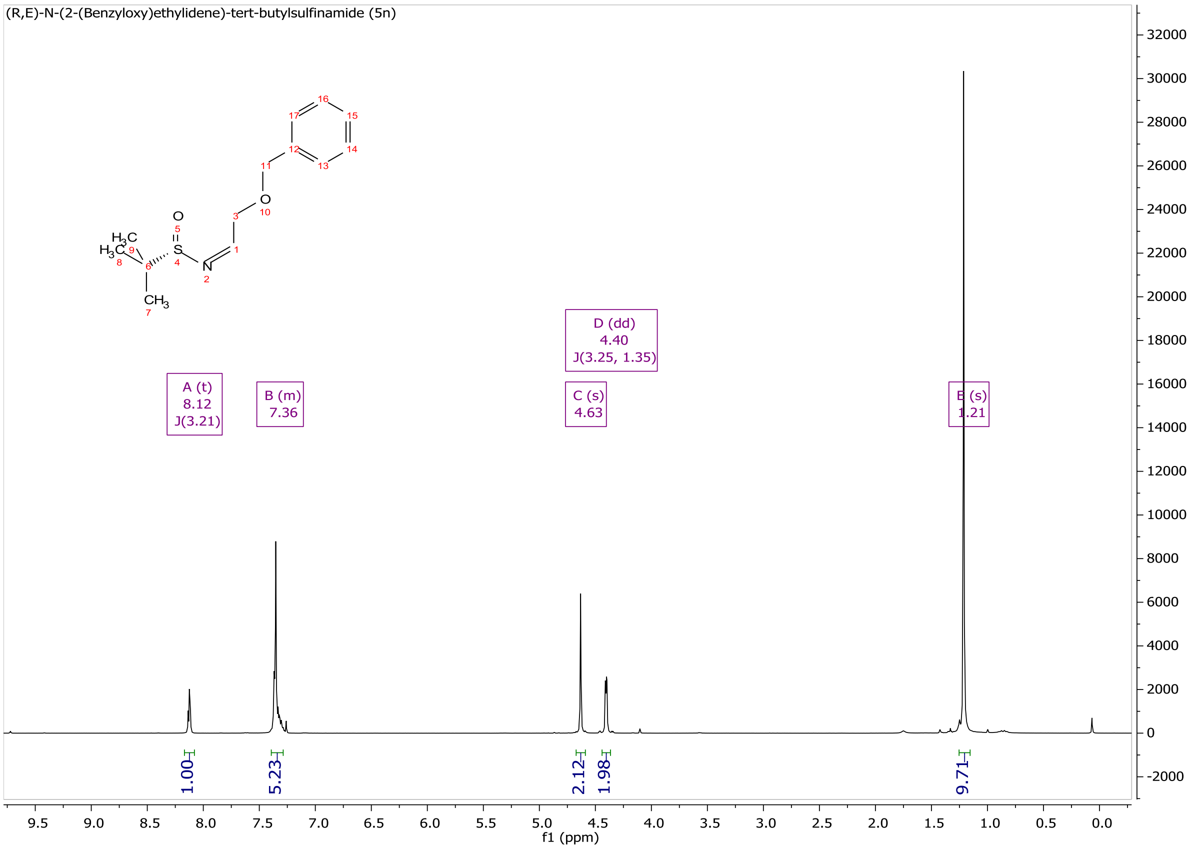
(R,E)-N-(2-(Benzyloxy)ethylidene)-tert-butylsulfinamide (5n)
A (t)
8.12
J(3.21)
B (m)
7.36
C (s)
4.63
D (dd)
4.40
J(3.25, 1.35)
E (s)
1.21
1.00
5.23
2.12
1.98
9.71
f1 (ppm)

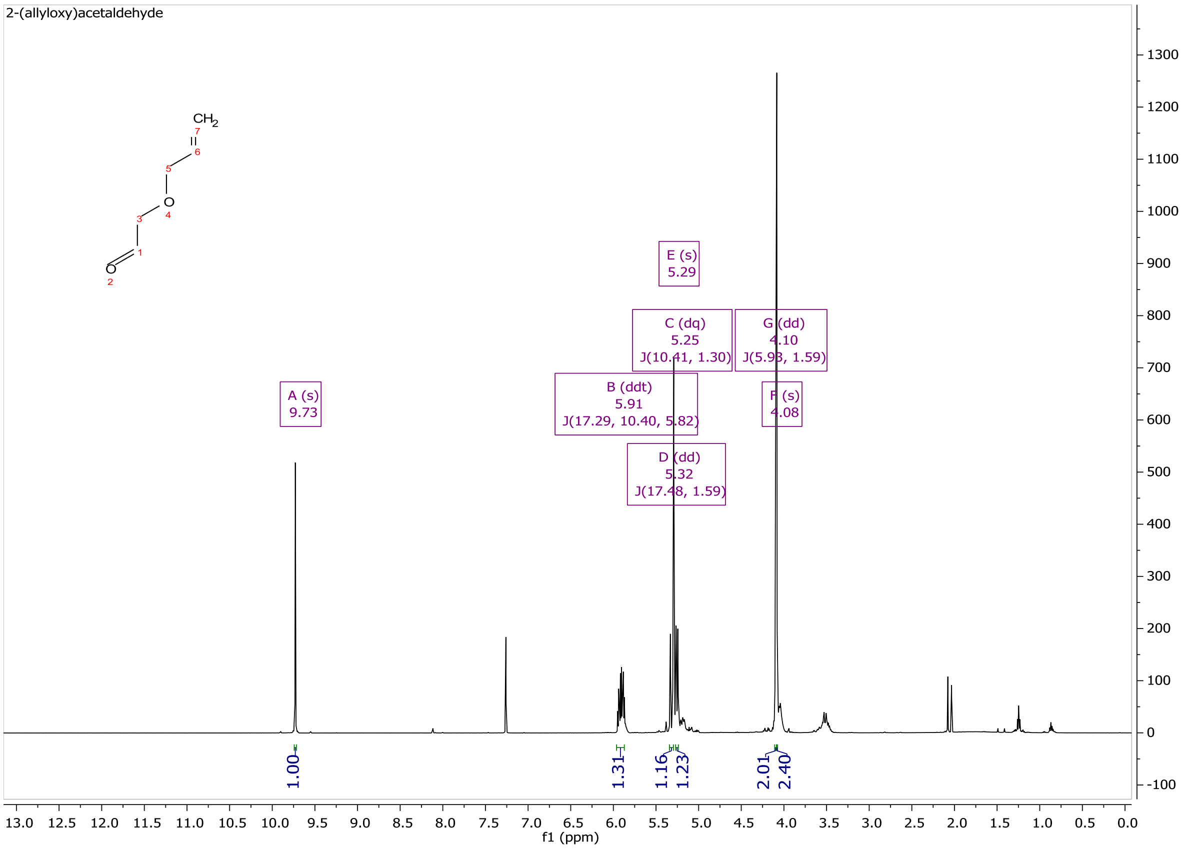
2-(allyloxy)acetaldehyde
A (s)
9.73
B (ddt)
5.91
J(17.29, 10.40, 5.82)
C (dq)
5.25
J(10.41, 1.30)
D (dd)
5.32
J(17.48, 1.59)
E (s)
5.29
F (s)
4.08
G (dd)
4.10
J(5.93, 1.59)
1.00
1.31
1.16
1.23
2.01
2.40
f1 (ppm)

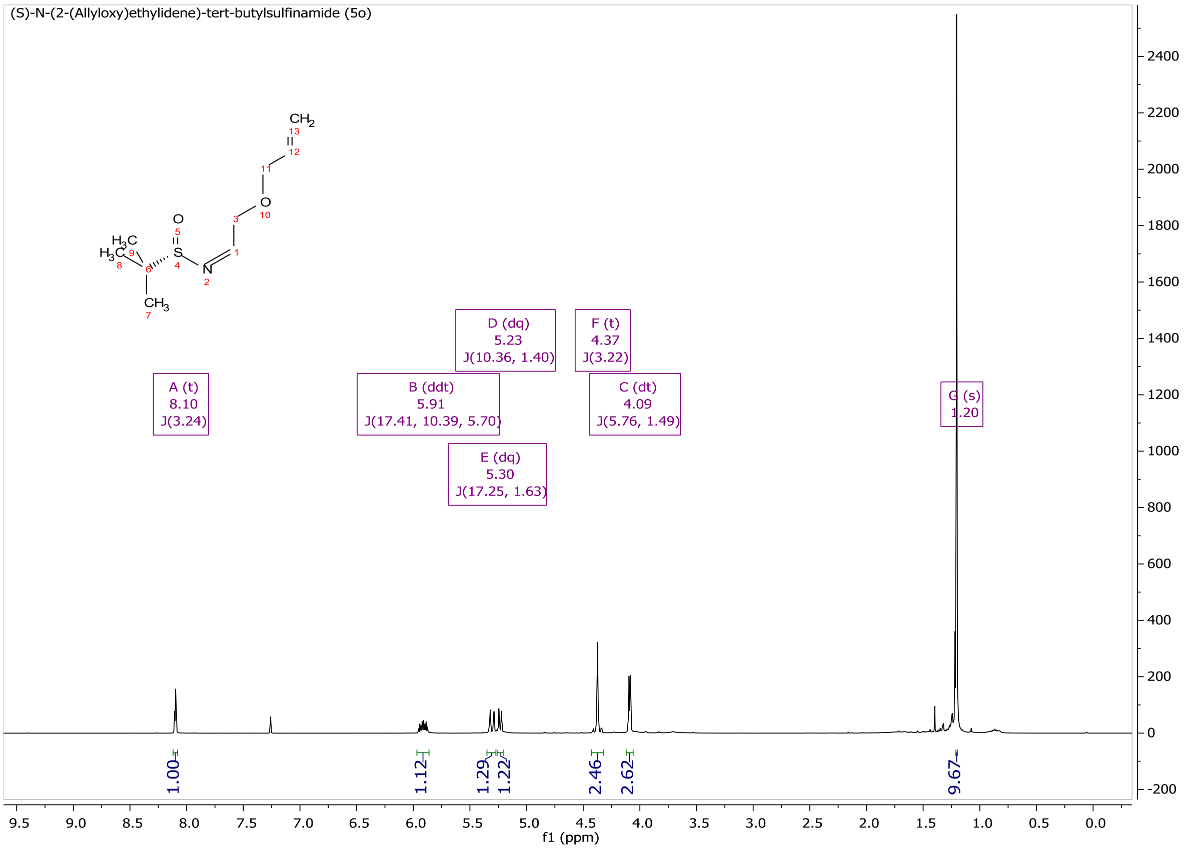
(S)-N-(2-(Allyloxy)ethylidene)-tert-butylsulfinamide (5o)
A (t)
8.10
J(3.24)
B (ddt)
5.91
J(17.41, 10.39, 5.70)
C (dt)
4.09
J(5.76, 1.49)
D (dq)
5.23
J(10.36, 1.40)
E (dq)
5.30
J(17.25, 1.63)
F (t)
4.37
J(3.22)
G (s)
1.20
1.00
1.12
1.29
1.22
2.46
2.62
9.67
f1 (ppm)

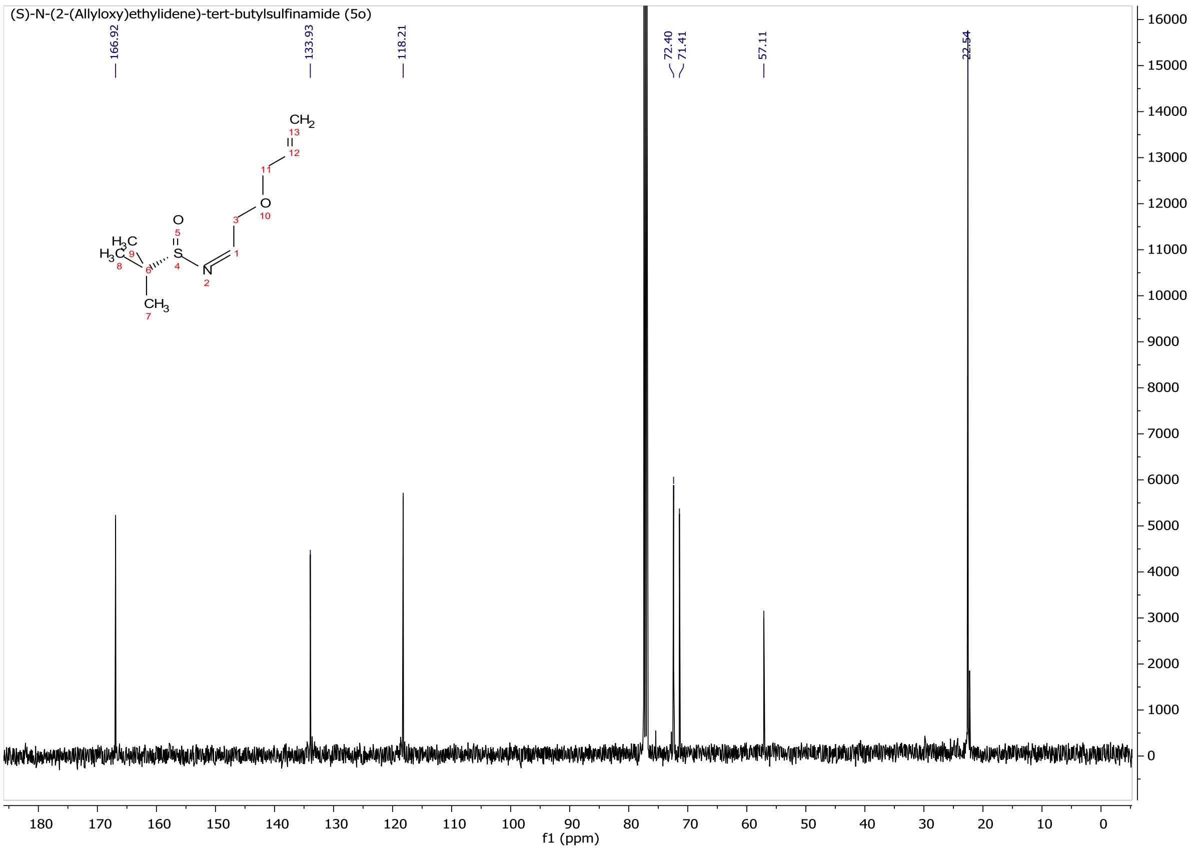
(S)-N-(2-(Allyloxy)ethylidene)-tert-butylsulfinamide (5o)
166.92
133.93
118.21
72.40
71.41
57.11
22.54
f1 (ppm)

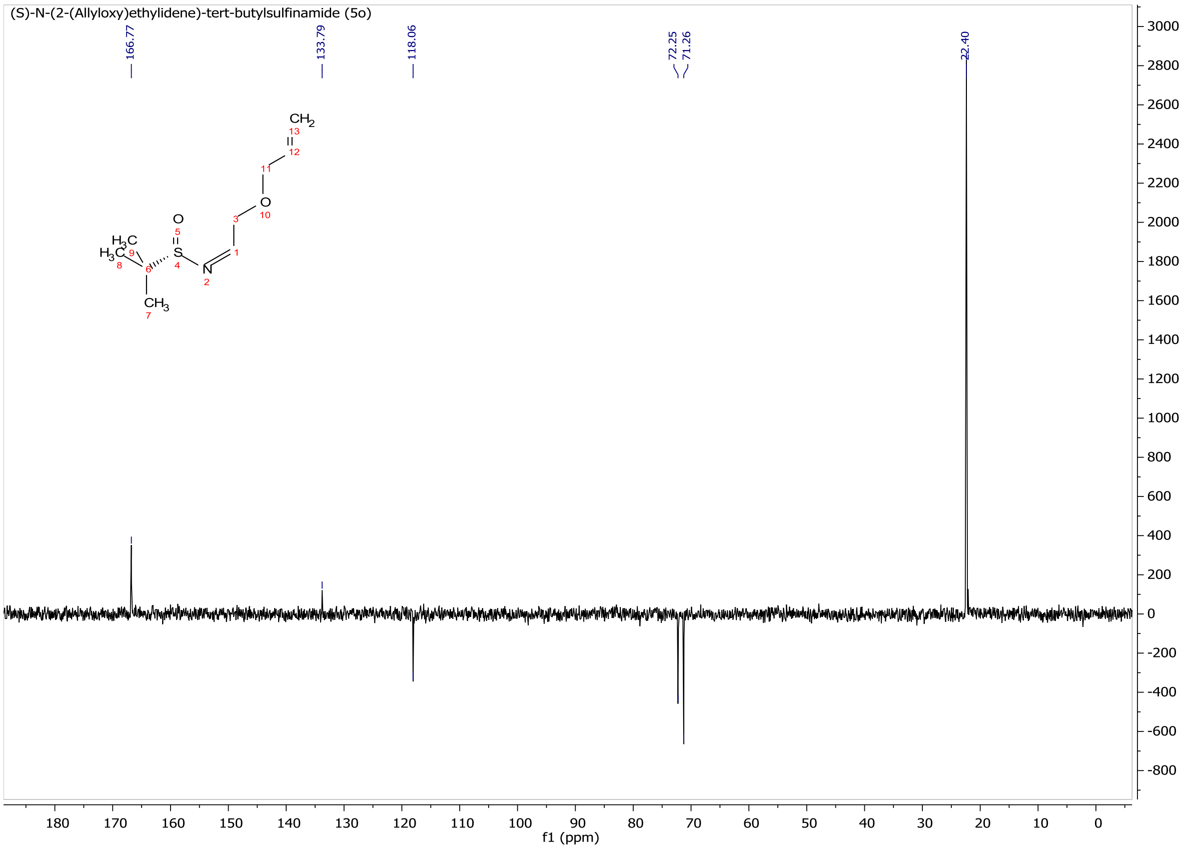
(S)-N-(2-(Allyloxy)ethylidene)-tert-butylsulfinamide (5o)
166.77
133.79
118.06
72.25
71.26
22.40
f1 (ppm)

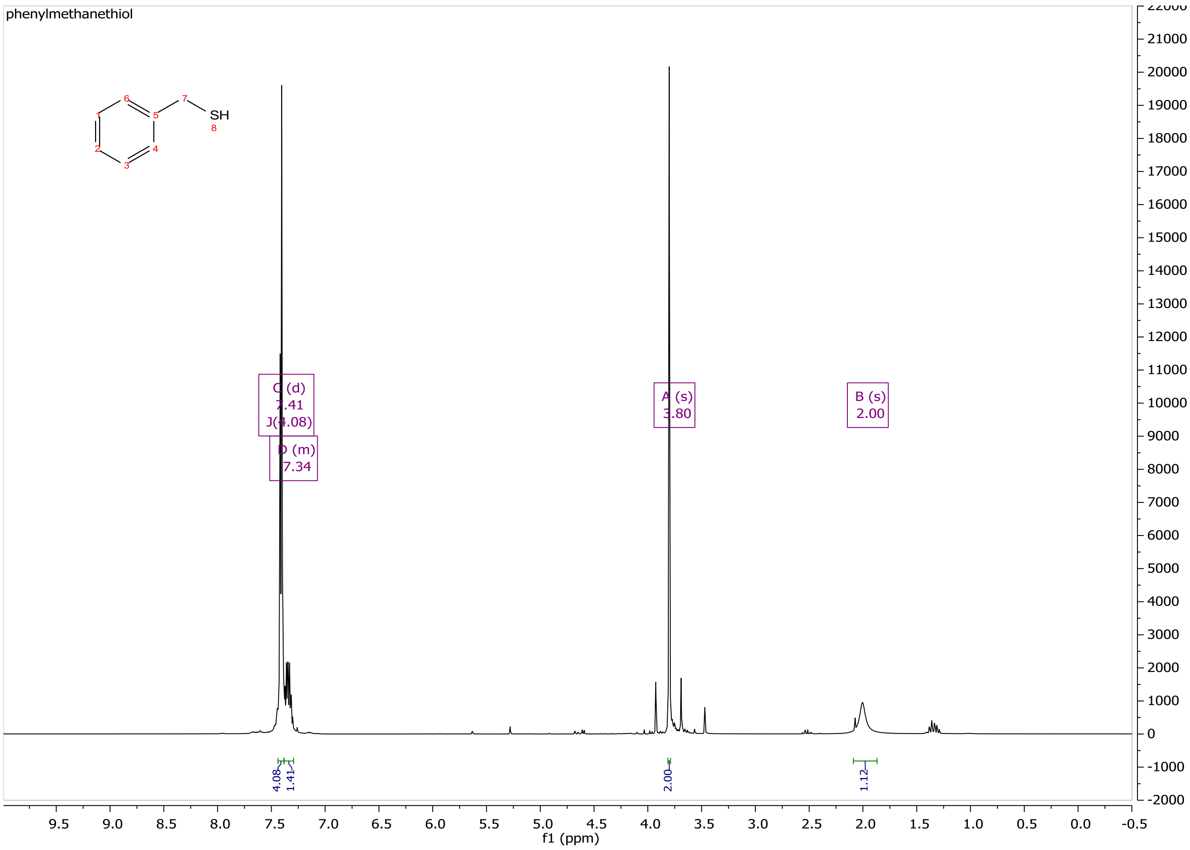
phenylmethanethiol
C (d)
7.41
J(4.08)
D (m)
7.34
A (s)
3.80
B (s)
2.00
4.08
1.41
2.00
1.12
f1 (ppm)

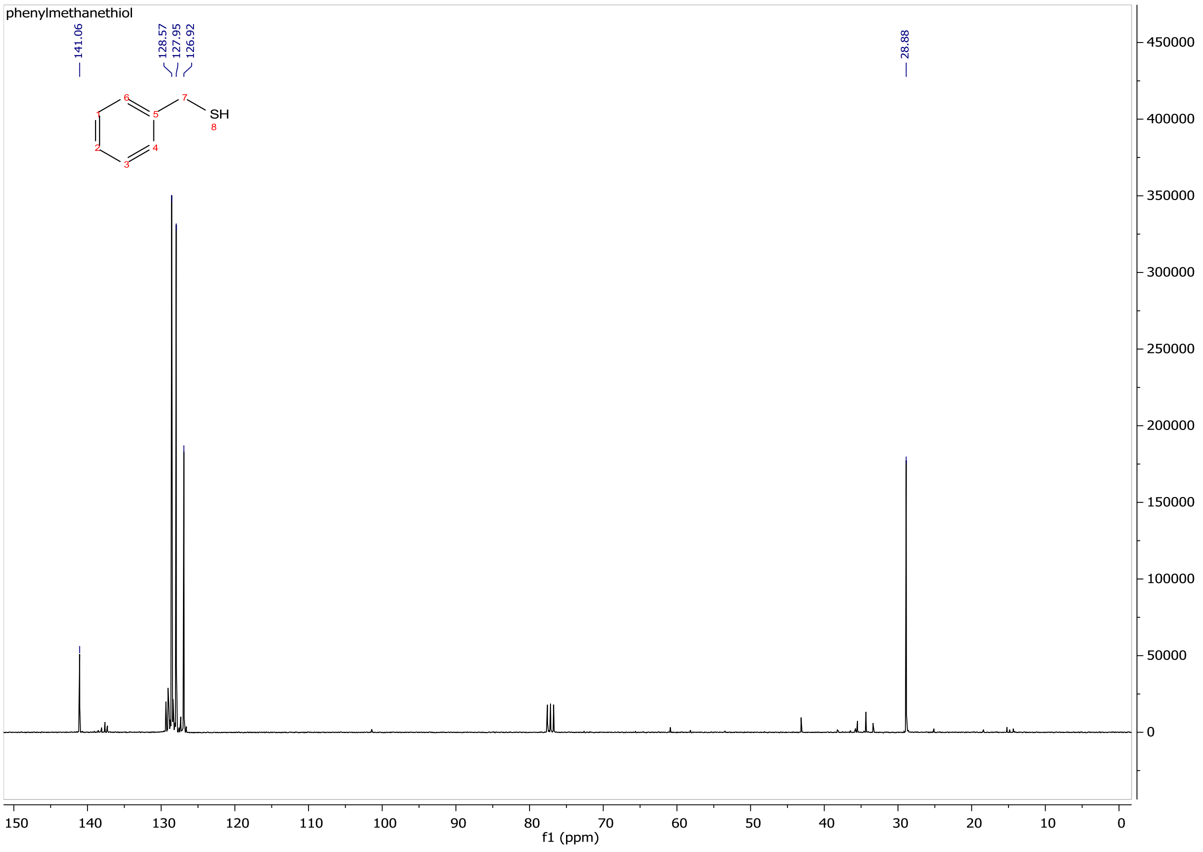
phenylmethanethiol
141.06
128.57
127.95
126.92
28.88
SH
f1 (ppm)

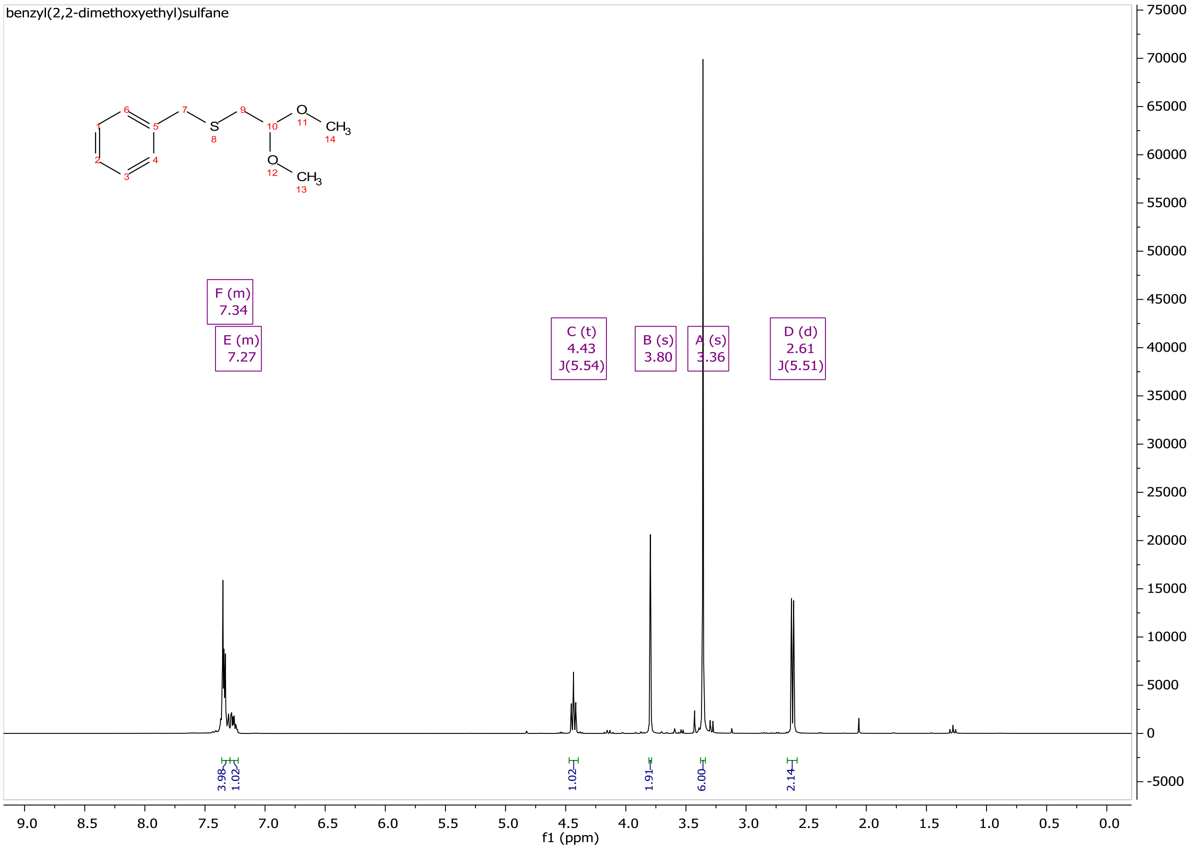
benzyl(2,2-dimethoxyethyl)sulfane
F (m) 7.34
E (m) 7.27
C (t) 4.43 J(5.54)
B (s) 3.80
A (s) 3.36
D (d) 2.61 J(5.51)
3.98
1.02
1.02
1.91
6.00
2.14
f1 (ppm)

2-(benzylthio)acetaldehyde

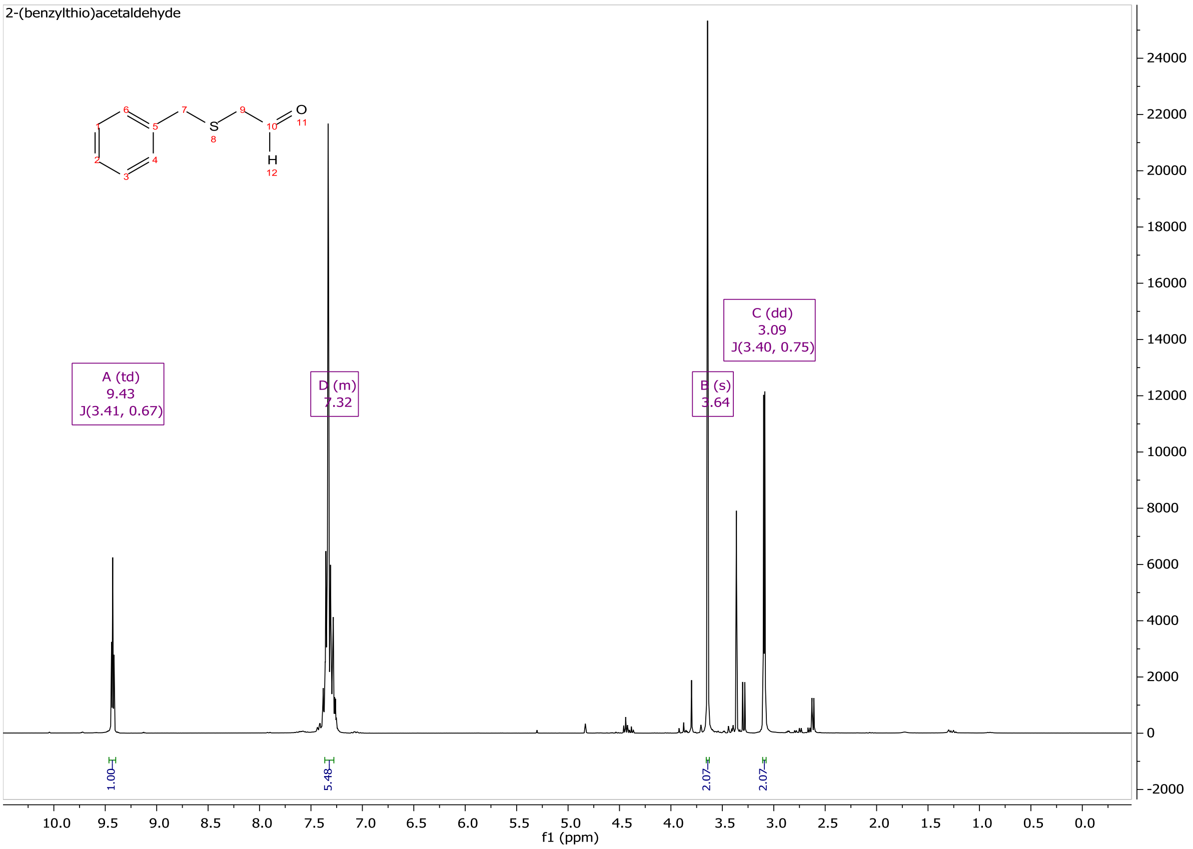

(R,E)-N-(2-(Benzylthio)ethylidene)-tert-butylsulfinamide (5p)

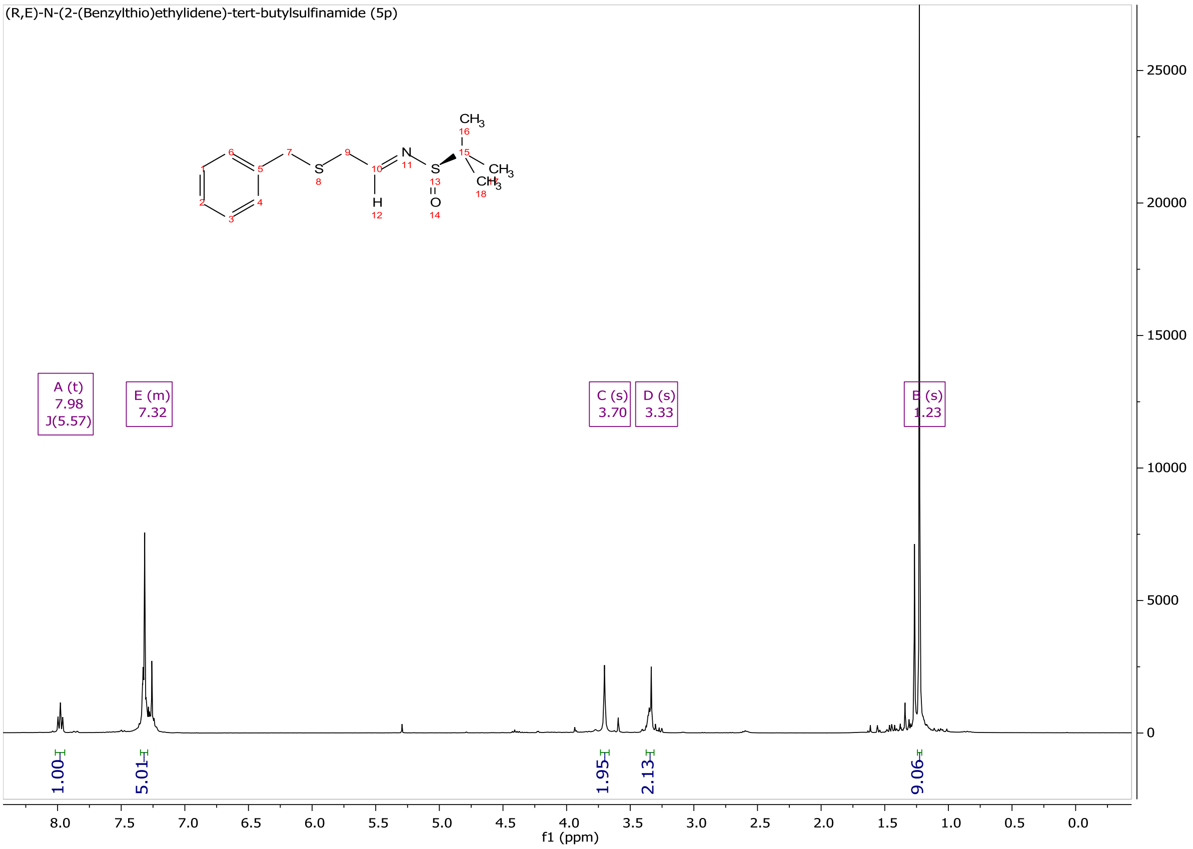

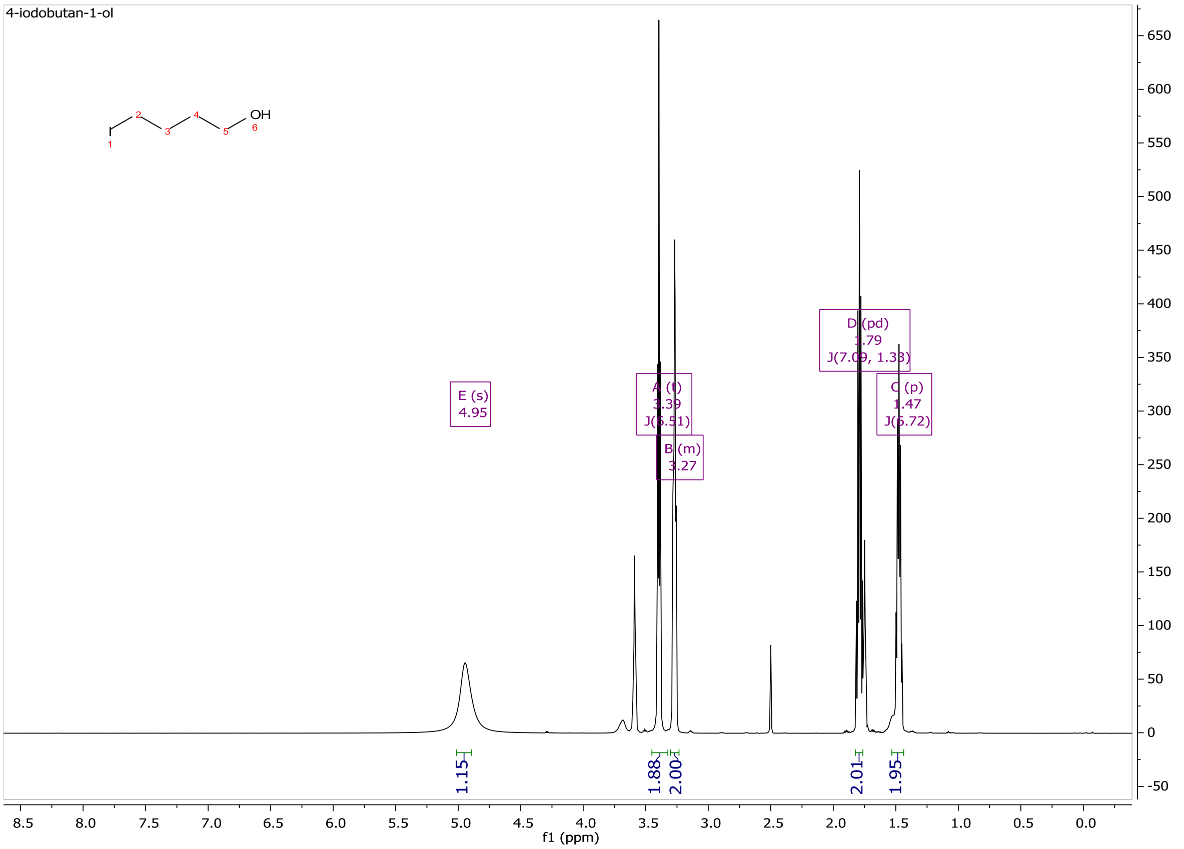
4-iodobutan-1-ol
OH
E (s)
4.95
A (t)
3.39
J(6.51)
B (m)
3.27
D (pd)
1.79
J(7.09, 1.33)
C (p)
1.47
J(6.72)
1.15
1.88
2.00
2.01
1.95
f1 (ppm)

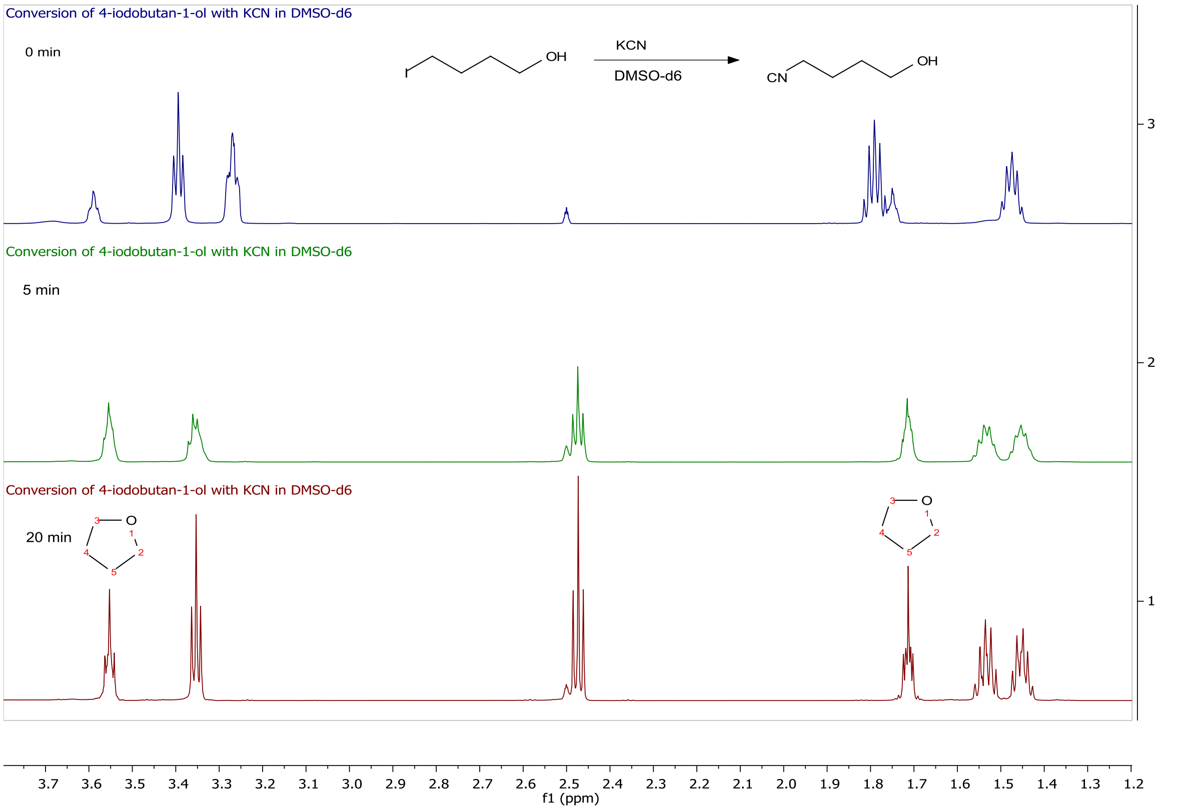
Conversion of 4-iodobutan-1-ol with KCN in DMSO-d6
0 min
OH
KCN
DMSO-d6
CN
OH
Conversion of 4-iodobutan-1-ol with KCN in DMSO-d6
5 min
Conversion of 4-iodobutan-1-ol with KCN in DMSO-d6
20 min
O
f1 (ppm)

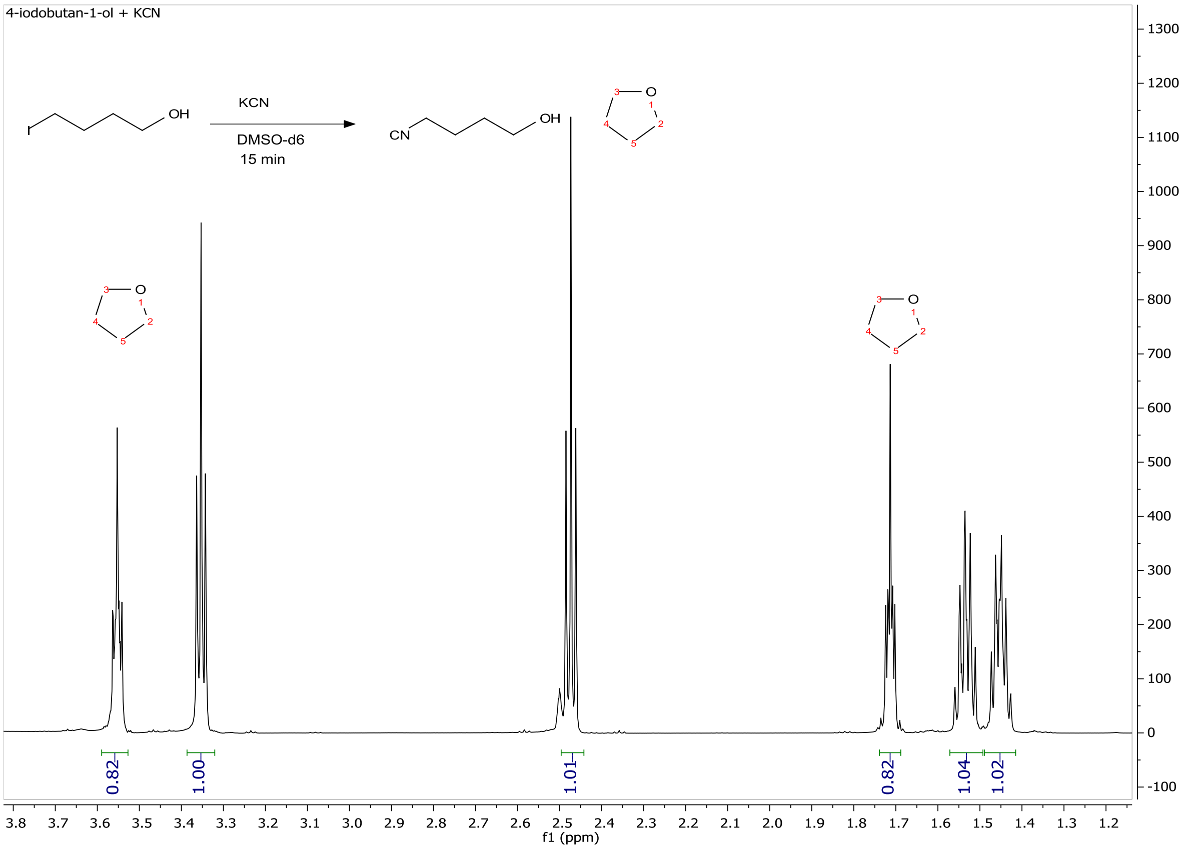
4-iodobutan-1-ol + KCN
KCN
DMSO-d6
15 min
0.82
1.00
1.01
0.82
1.04
1.02
f1 (ppm)

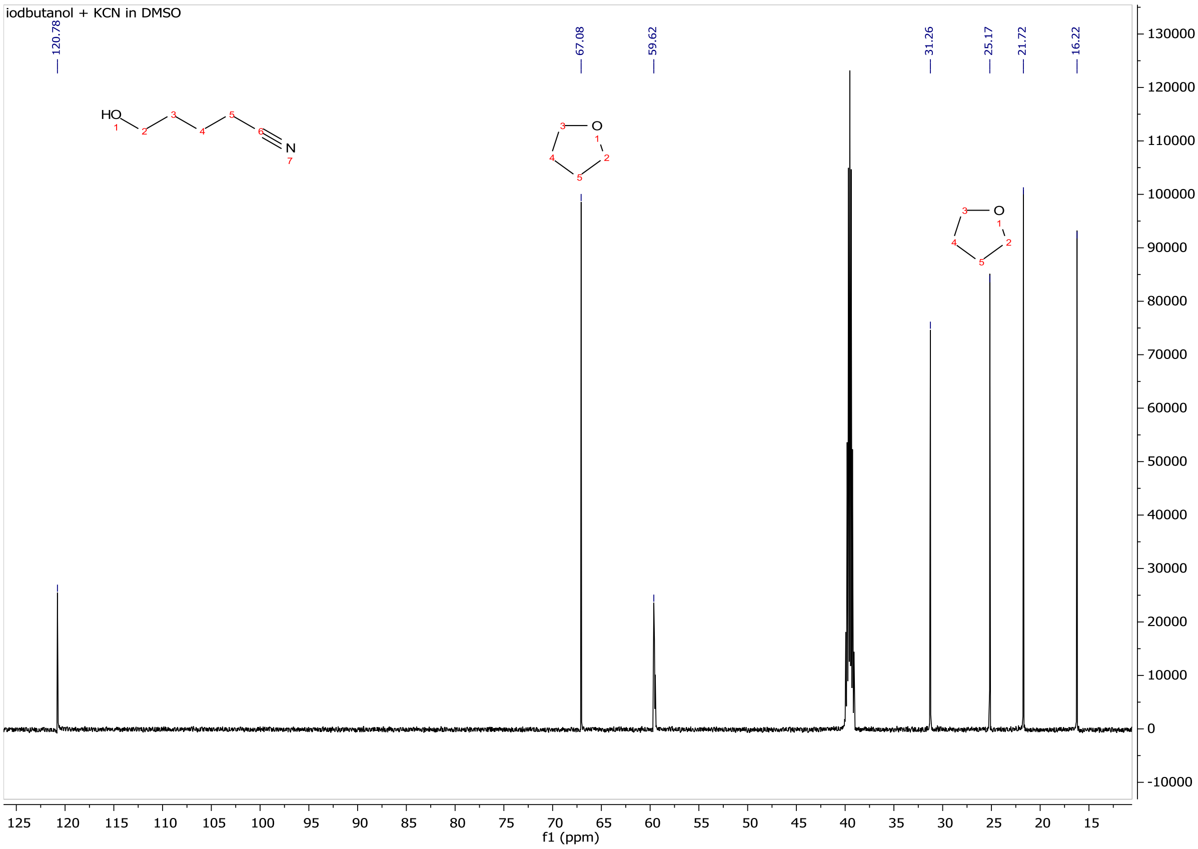
iodbutanol + KCN in DMSO
120.78
67.08
59.62
31.26
25.17
21.72
16.22
f1 (ppm)

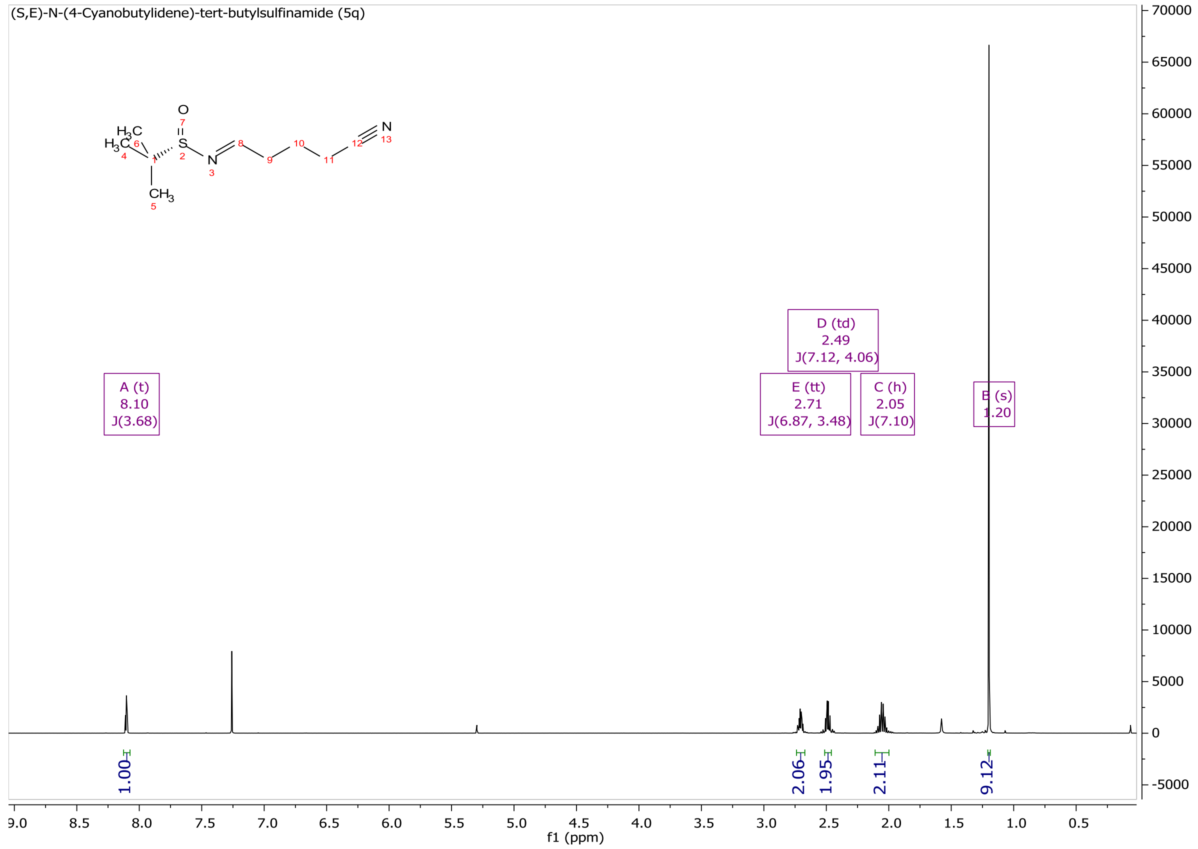
(S,E)-N-(4-Cyanobutylidene)-tert-butylsulfinamide (5q)
A (t)
8.10
J(3.68)
D (td)
2.49
J(7.12, 4.06)
E (tt)
2.71
J(6.87, 3.48)
C (h)
2.05
J(7.10)
B (s)
1.20
1.00
2.06
1.95
2.11
9.12
f1 (ppm)

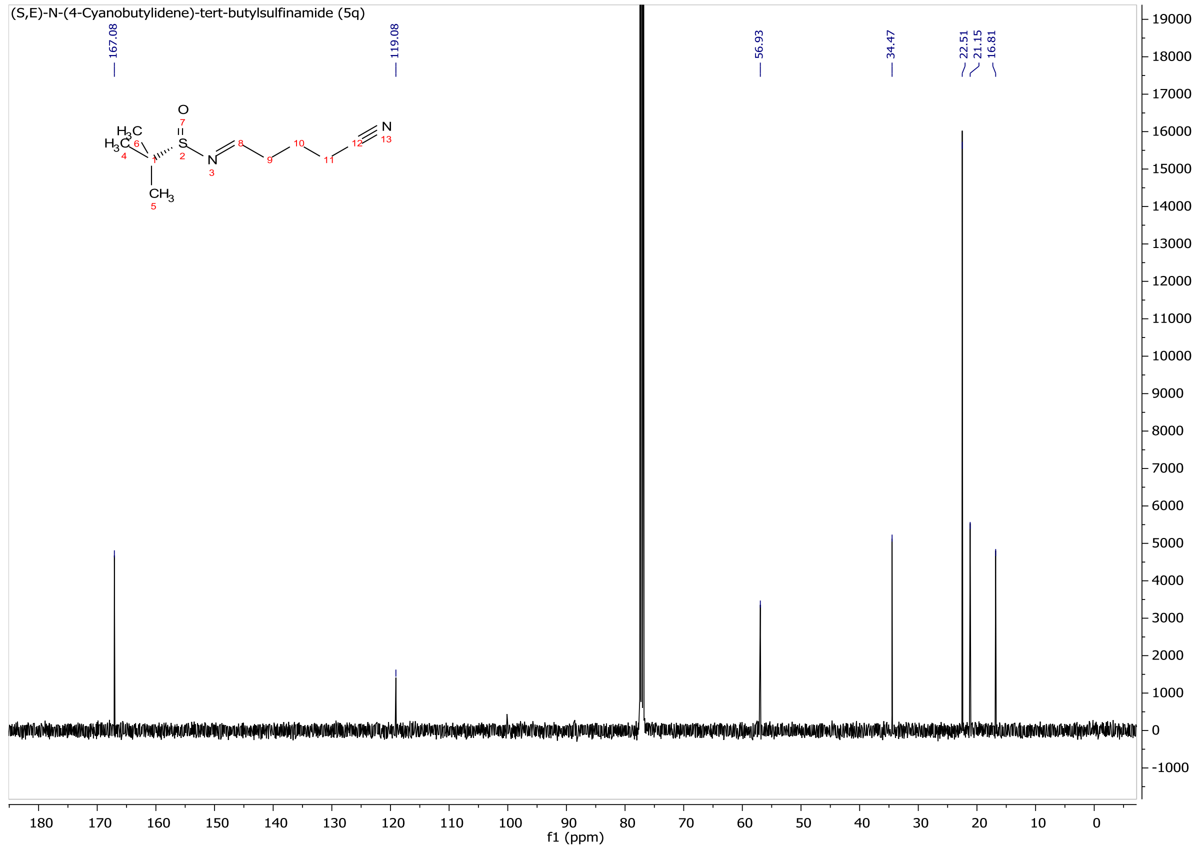
(S,E)-N-(4-Cyanobutylidene)-tert-butylsulfinamide (5q)
167.08
119.08
56.93
34.47
22.51
21.15
16.81
f1 (ppm)

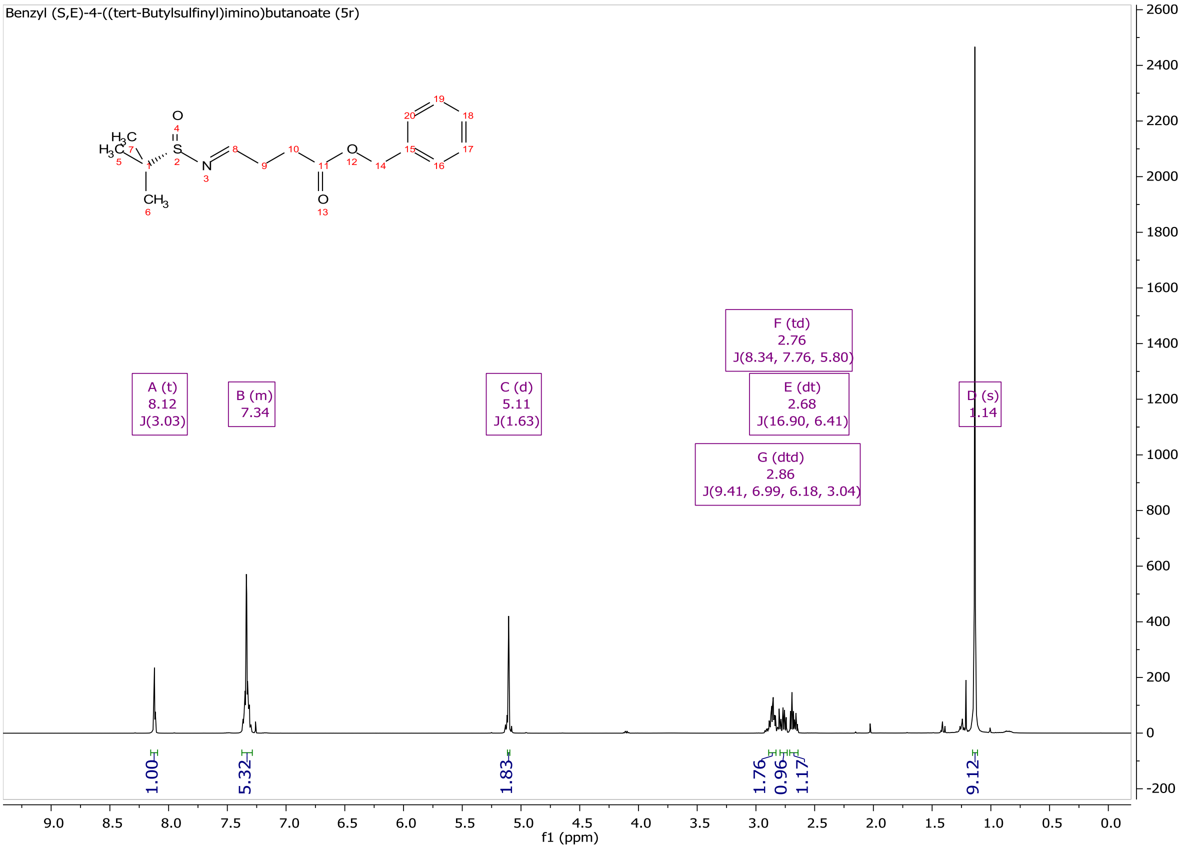
Benzyl (S,E)-4-((tert-Butylsulfinyl)imino)butanoate (5r)
A (t) 8.12 J(3.03)
B (m) 7.34
C (d) 5.11 J(1.63)
F (td) 2.76 J(8.34, 7.76, 5.80)
E (dt) 2.68 J(16.90, 6.41)
G (dtd) 2.86 J(9.41, 6.99, 6.18, 3.04)
D (s) 1.14
1.00
5.32
1.83
1.76
0.96
1.17
9.12
f1 (ppm)

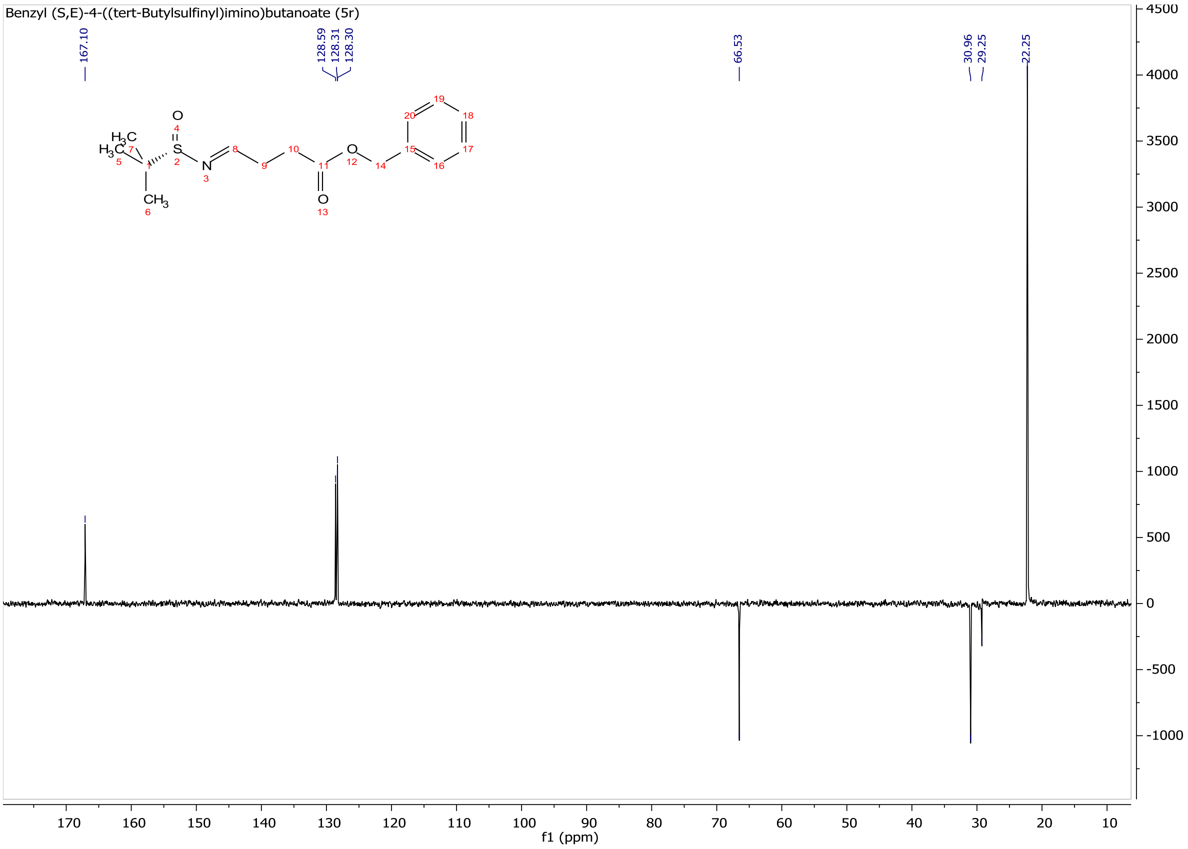
Benzyl (S,E)-4-((tert-Butylsulfinyl)imino)butanoate (5r)
167.10
128.59
128.31
128.30
66.53
30.96
29.25
22.25
f1 (ppm)

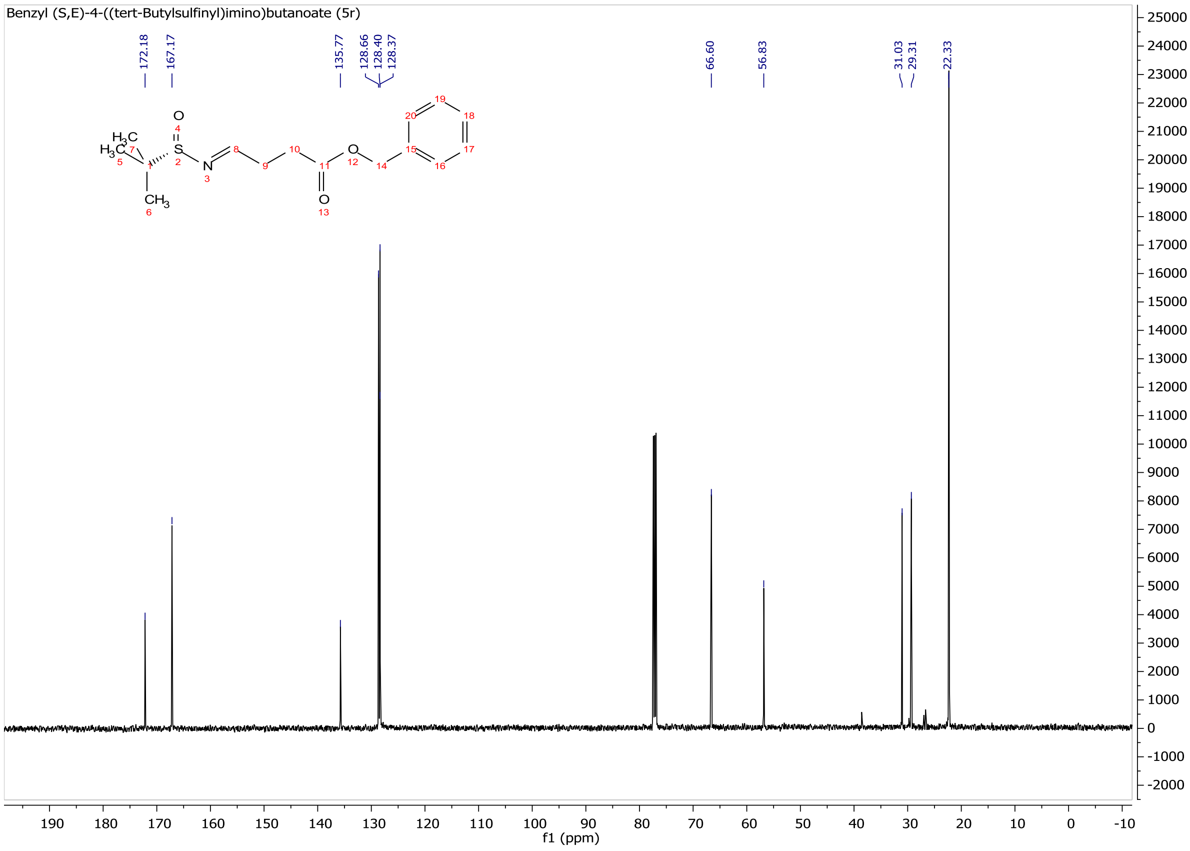
Benzyl (S,E)-4-((tert-Butylsulfinyl)imino)butanoate (5r)
172.18
167.17
135.77
128.66
128.40
128.37
66.60
56.83
31.03
29.31
22.33
f1 (ppm)

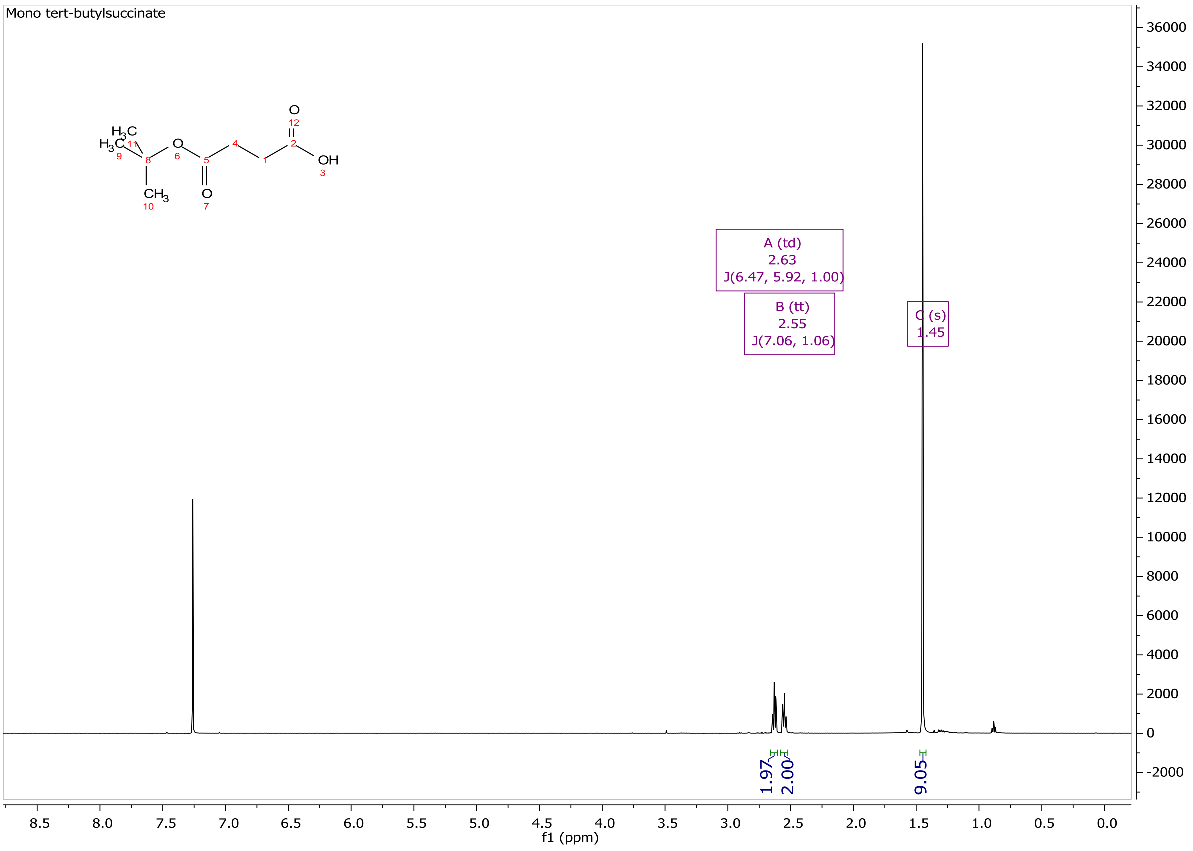
Mono tert-butylsuccinate
A (td)
2.63
J(6.47, 5.92, 1.00)
B (tt)
2.55
J(7.06, 1.06)
C (s)
1.45
1.97
2.00
9.05
f1 (ppm)

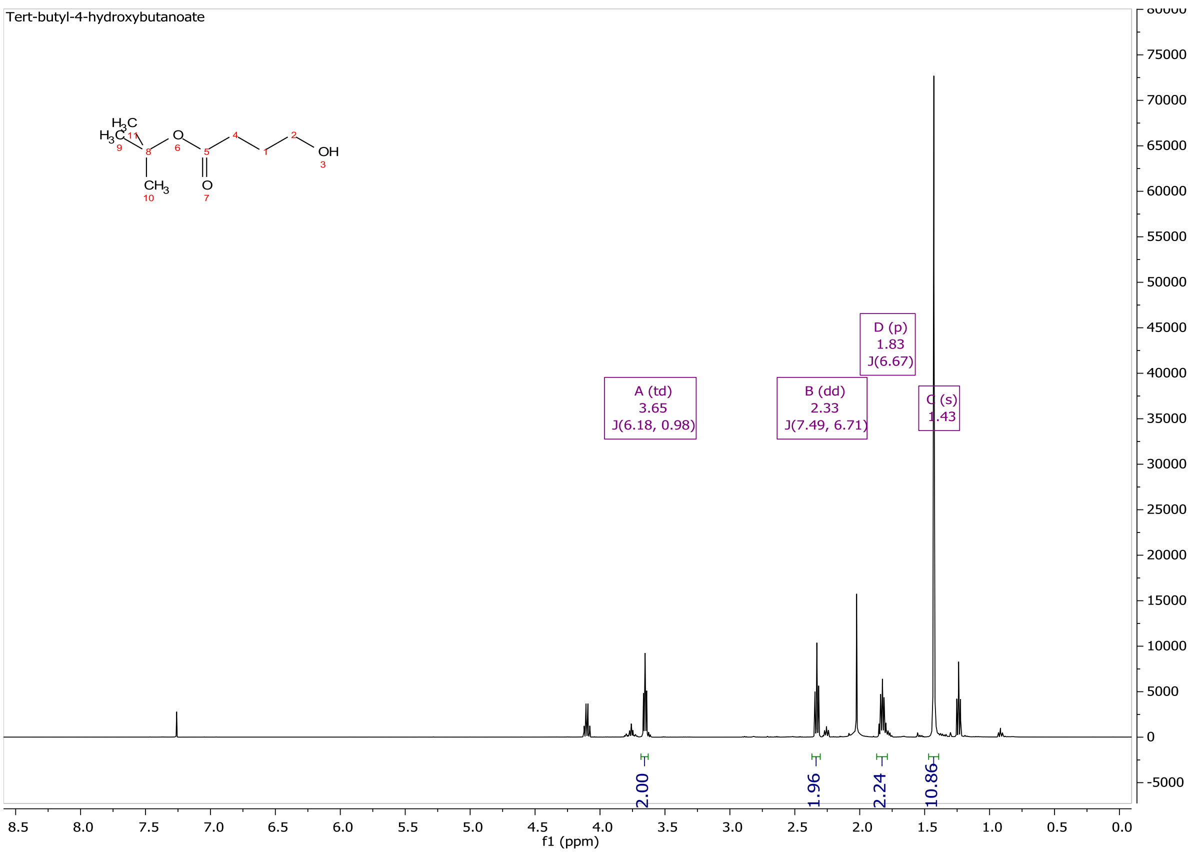
Tert-butyl-4-hydroxybutanoate
A (td)
3.65
J(6.18, 0.98)
B (dd)
2.33
J(7.49, 6.71)
D (p)
1.83
J(6.67)
C (s)
1.43
2.00
1.96
2.24
10.86
f1 (ppm)

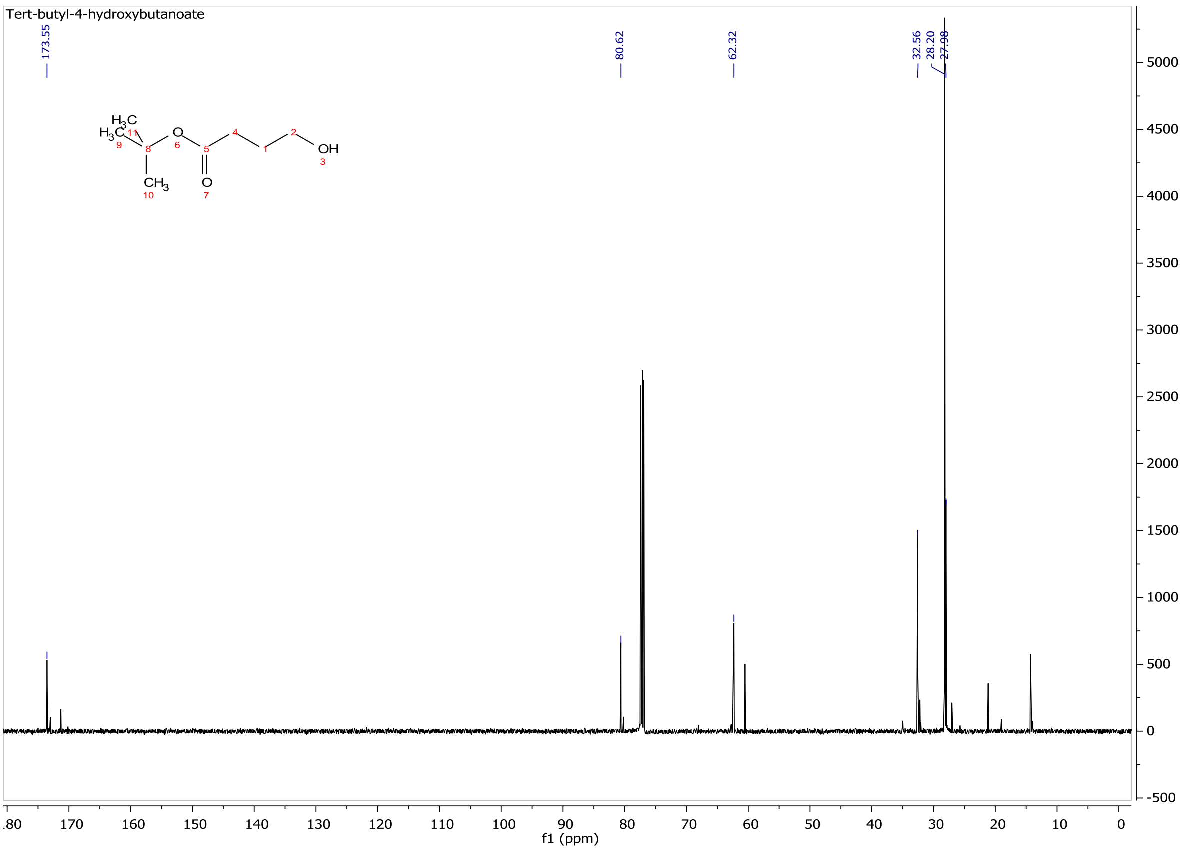
Tert-butyl-4-hydroxybutanoate
173.55
80.62
62.32
32.56
28.20
27.98
f1 (ppm)

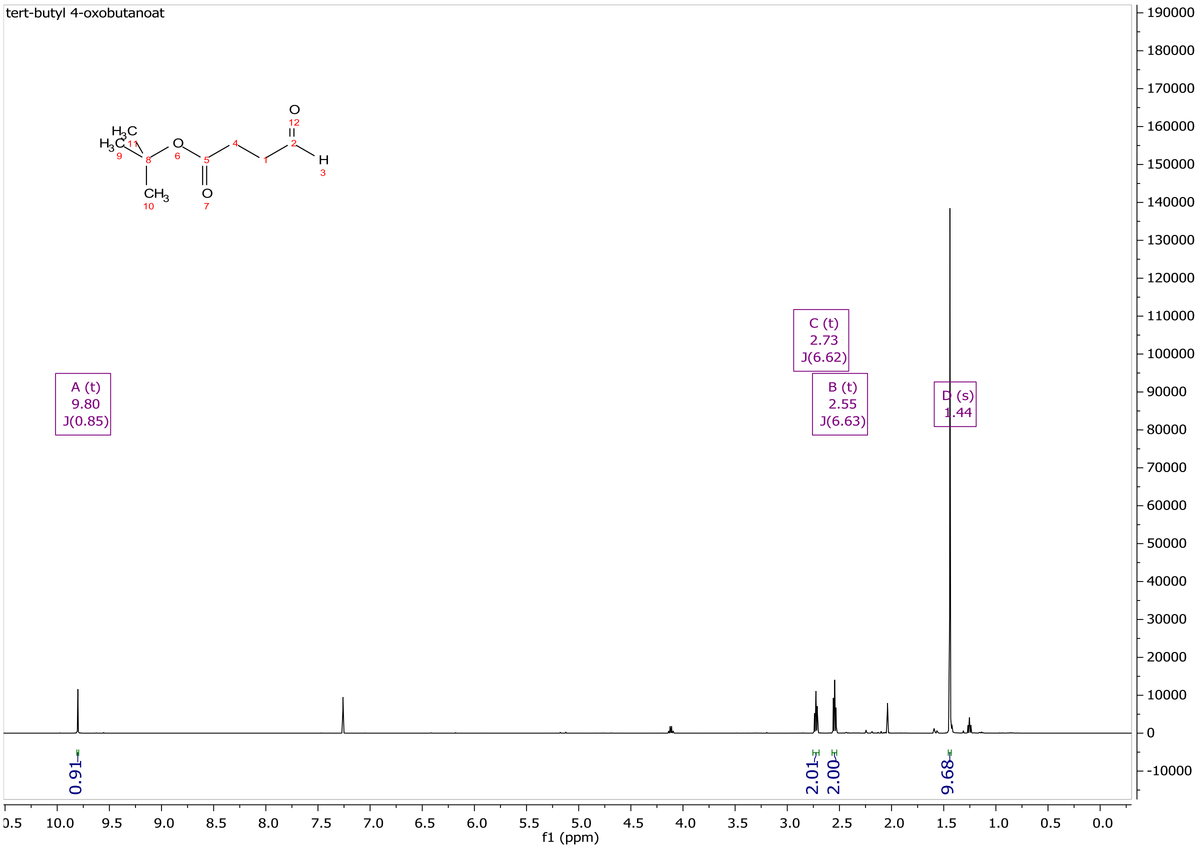
tert-butyl 4-oxobutanoat
A (t)
9.80
J(0.85)
C (t)
2.73
J(6.62)
B (t)
2.55
J(6.63)
D (s)
1.44
0.91
2.01
2.00
9.68
f1 (ppm)

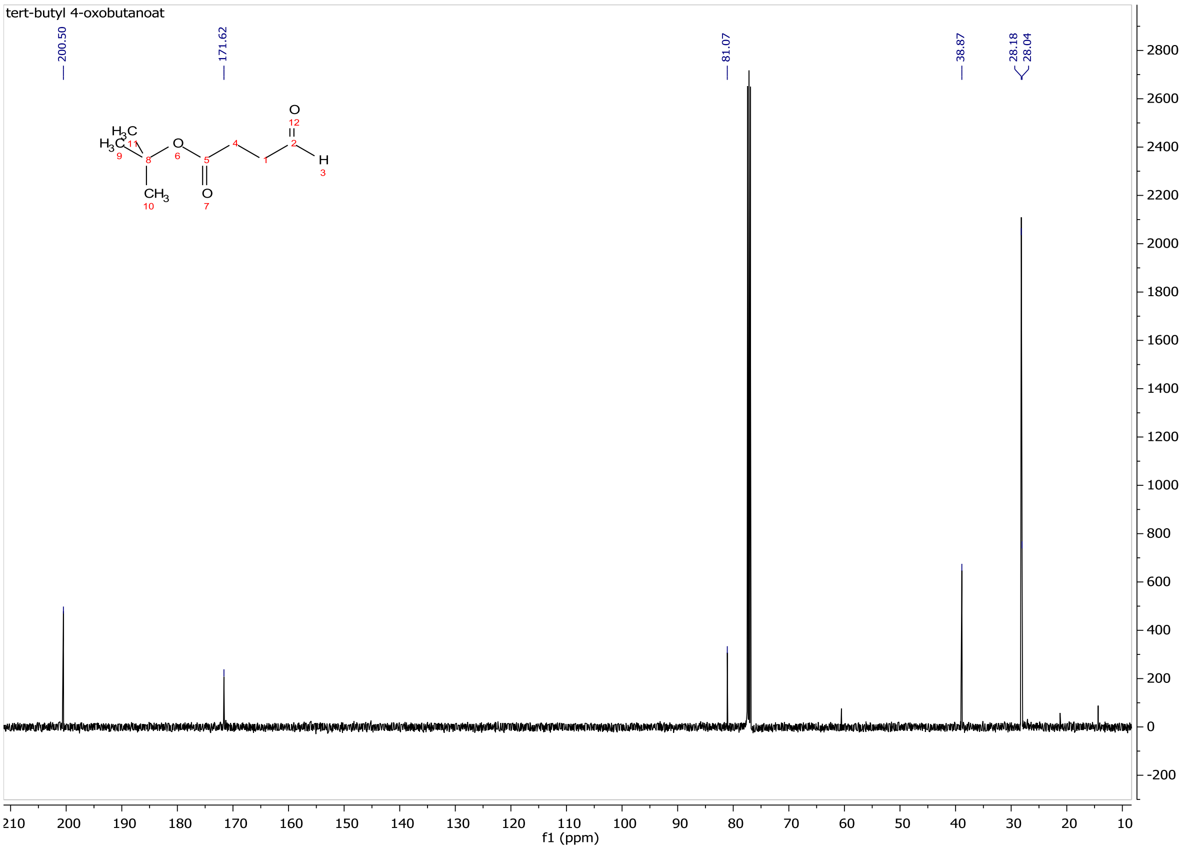
tert-butyl 4-oxobutanoat
200.50
171.62
81.07
38.87
28.18
28.04
f1 (ppm)

tert-Butyl (S,E)-4-((tert-Butylsulfinyl)imino)butanoate (5s)

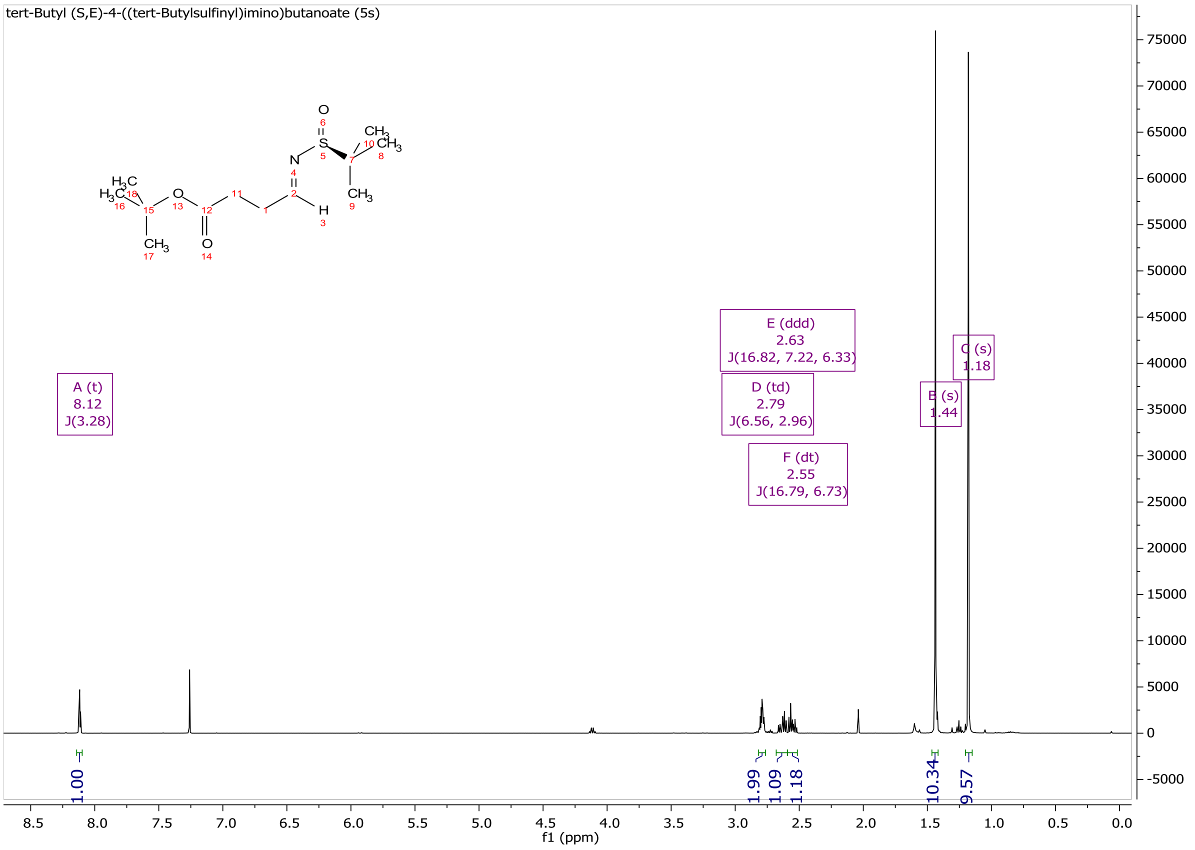

tert-Butyl (S,E)-4-((tert-Butylsulfinyl)imino)butanoate (5s)

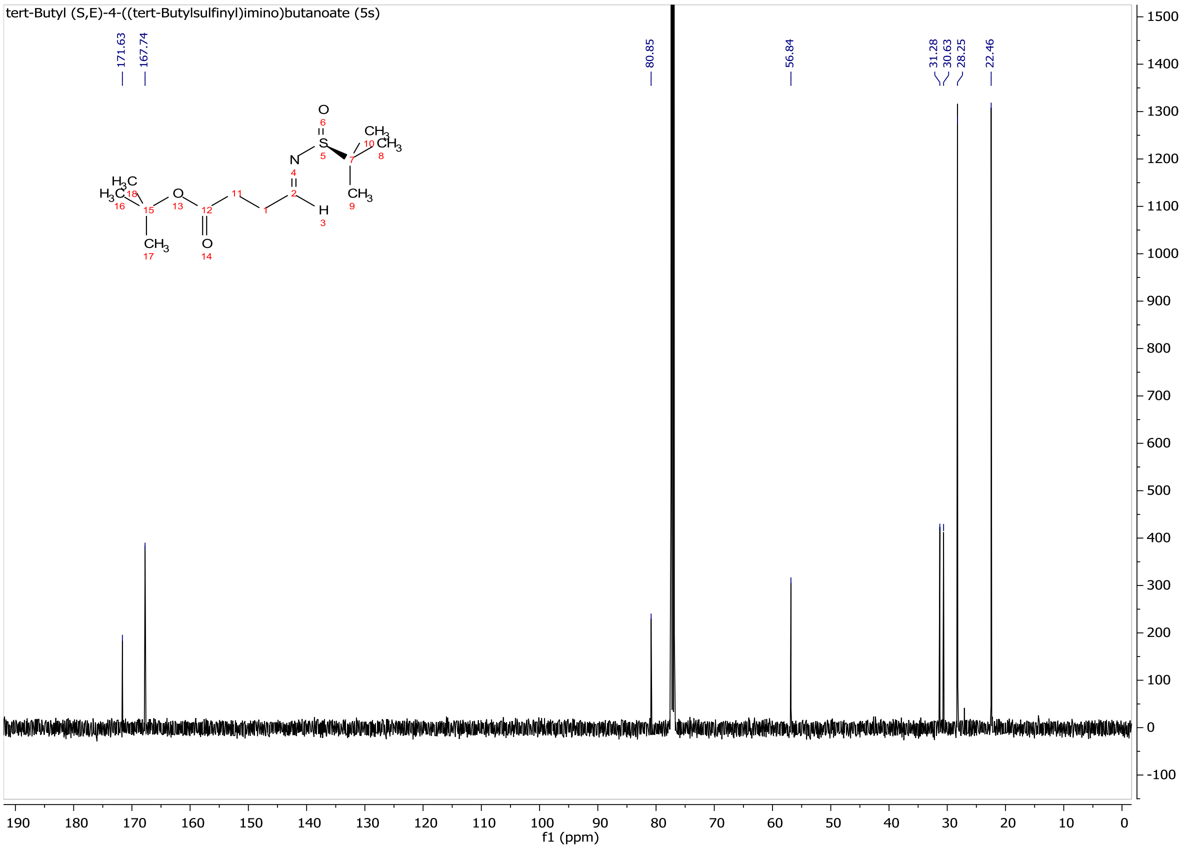

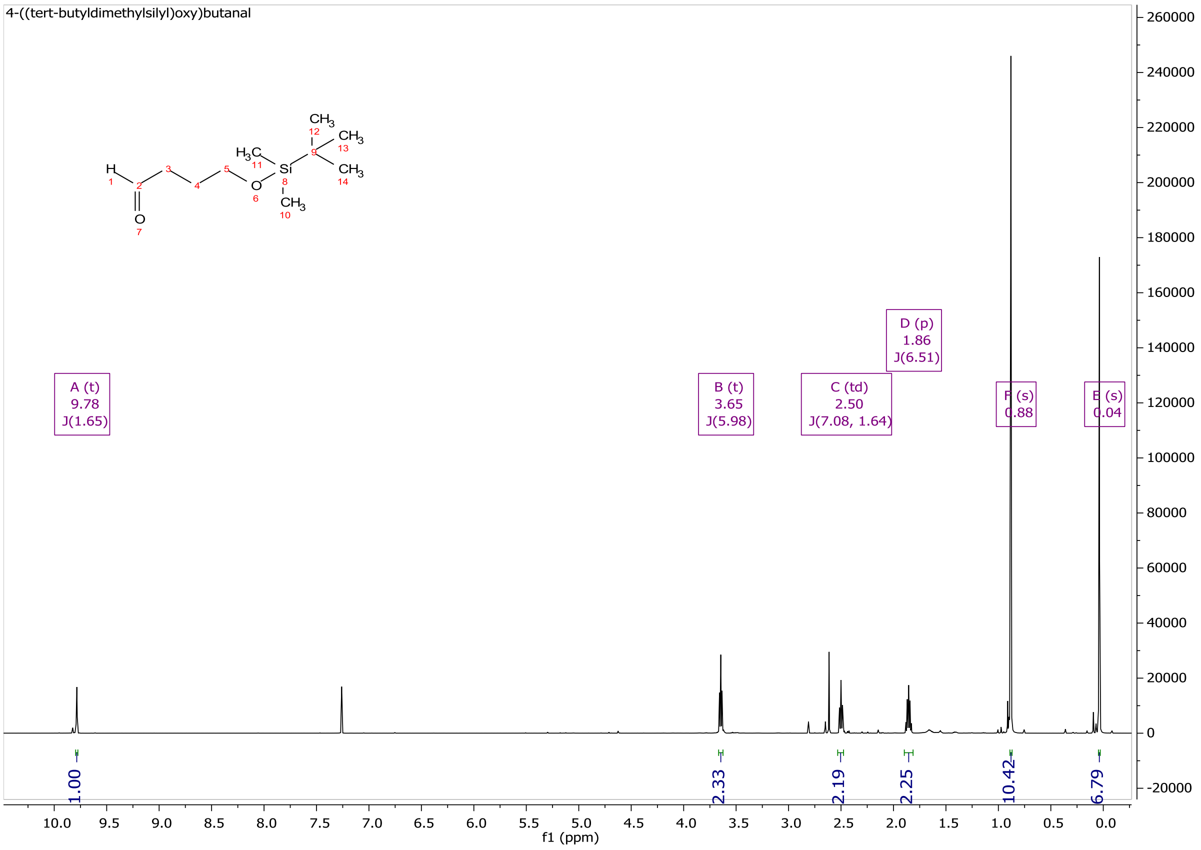
4-((tert-butyldimethylsilyl)oxy)butanal
A (t) 9.78 J(1.65)
B (t) 3.65 J(5.98)
C (td) 2.50 J(7.08, 1.64)
D (p) 1.86 J(6.51)
F (s) 0.88
E (s) 0.04
1.00
2.33
2.19
2.25
10.42
6.79
f1 (ppm)

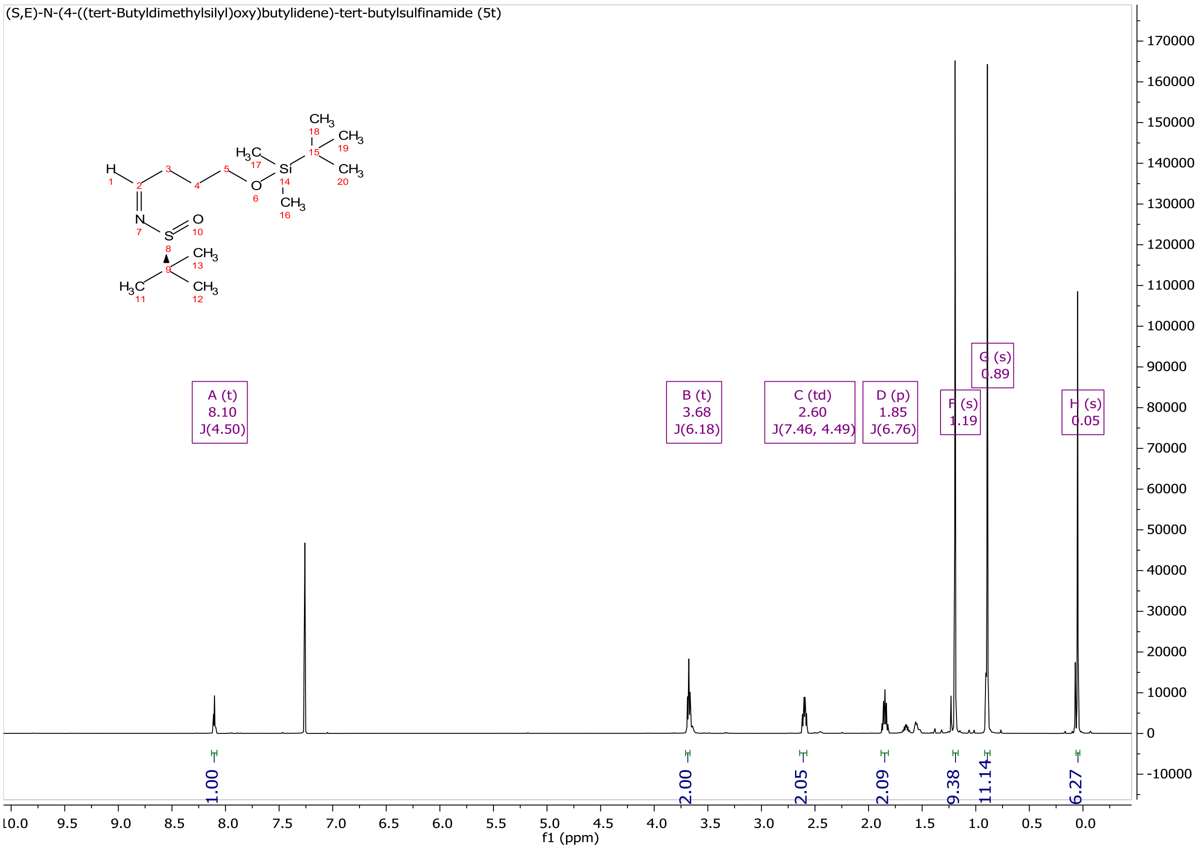
(S,E)-N-(4-((tert-Butyldimethylsilyl)oxy)butylidene)-tert-butylsulfinamide (5t)
A (t) 8.10 J(4.50)
B (t) 3.68 J(6.18)
C (td) 2.60 J(7.46, 4.49)
D (p) 1.85 J(6.76)
F (s) 1.19
G (s) 0.89
H (s) 0.05
1.00
2.00
2.05
2.09
9.38
11.14
6.27
f1 (ppm)

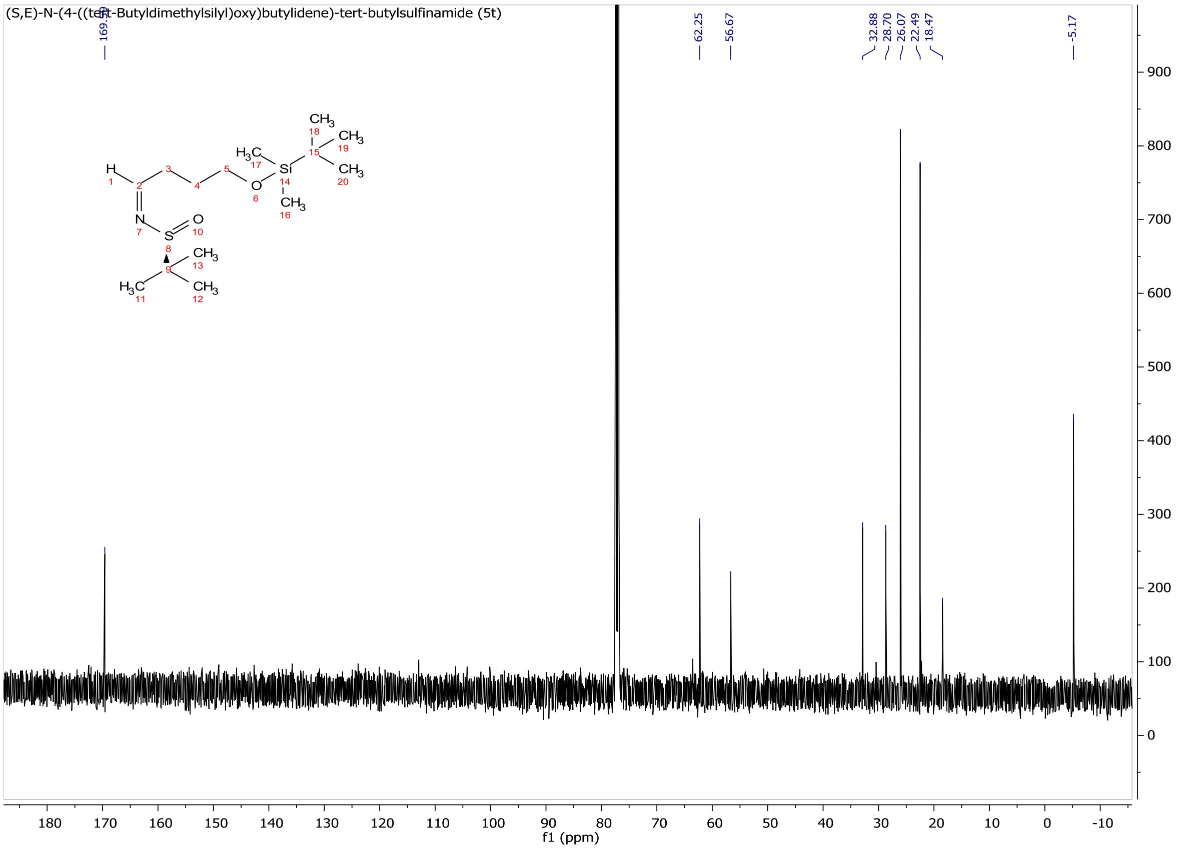
(S,E)-N-(4-((tert-Butyldimethylsilyl)oxy)butylidene)-tert-butylsulfinamide (5t)
169.59
62.25
56.67
32.88
28.70
26.07
22.49
18.47
-5.17
f1 (ppm)

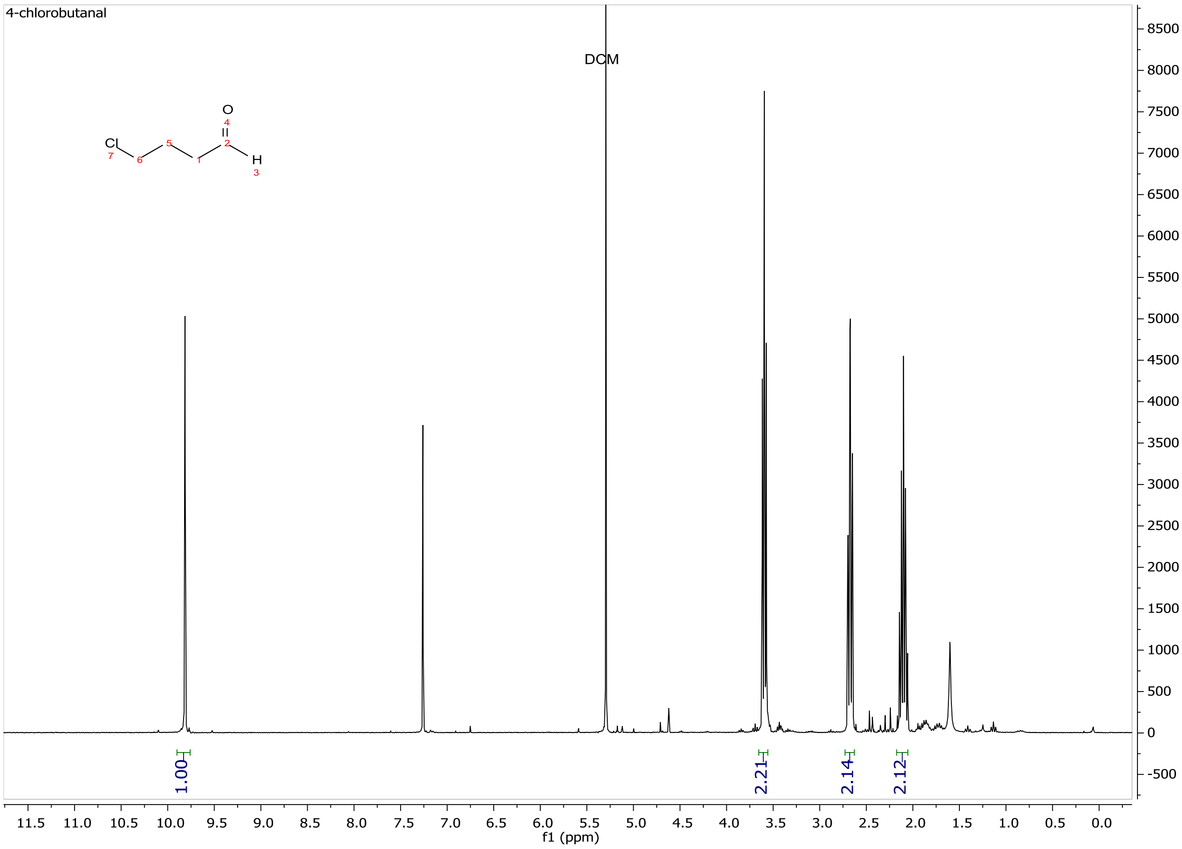
4-chlorobutanal
DCM
1.00
2.21
2.14
2.12
f1 (ppm)

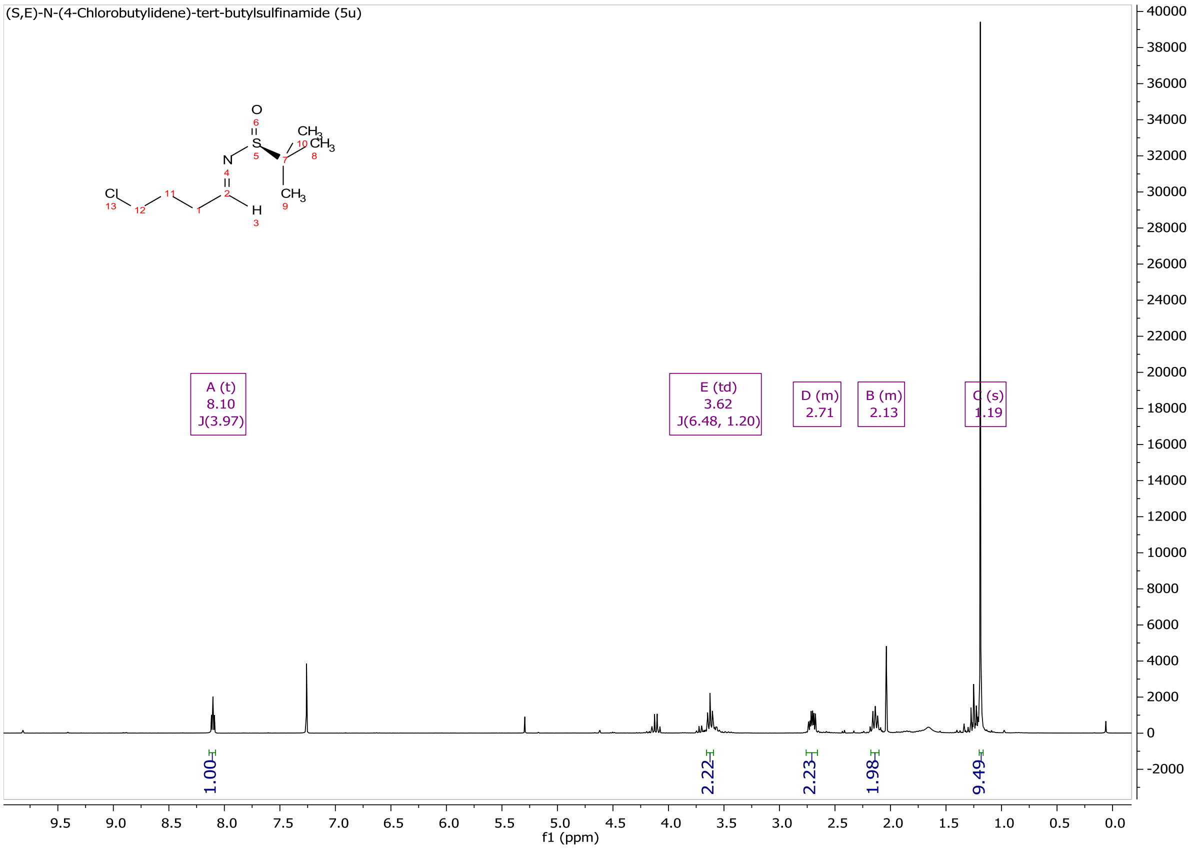
(S,E)-N-(4-Chlorobutylidene)-tert-butylsulfinamide (5u)
A (t)
8.10
J(3.97)
E (td)
3.62
J(6.48, 1.20)
D (m)
2.71
B (m)
2.13
C (s)
1.19
1.00
2.22
2.23
1.98
9.49
f1 (ppm)

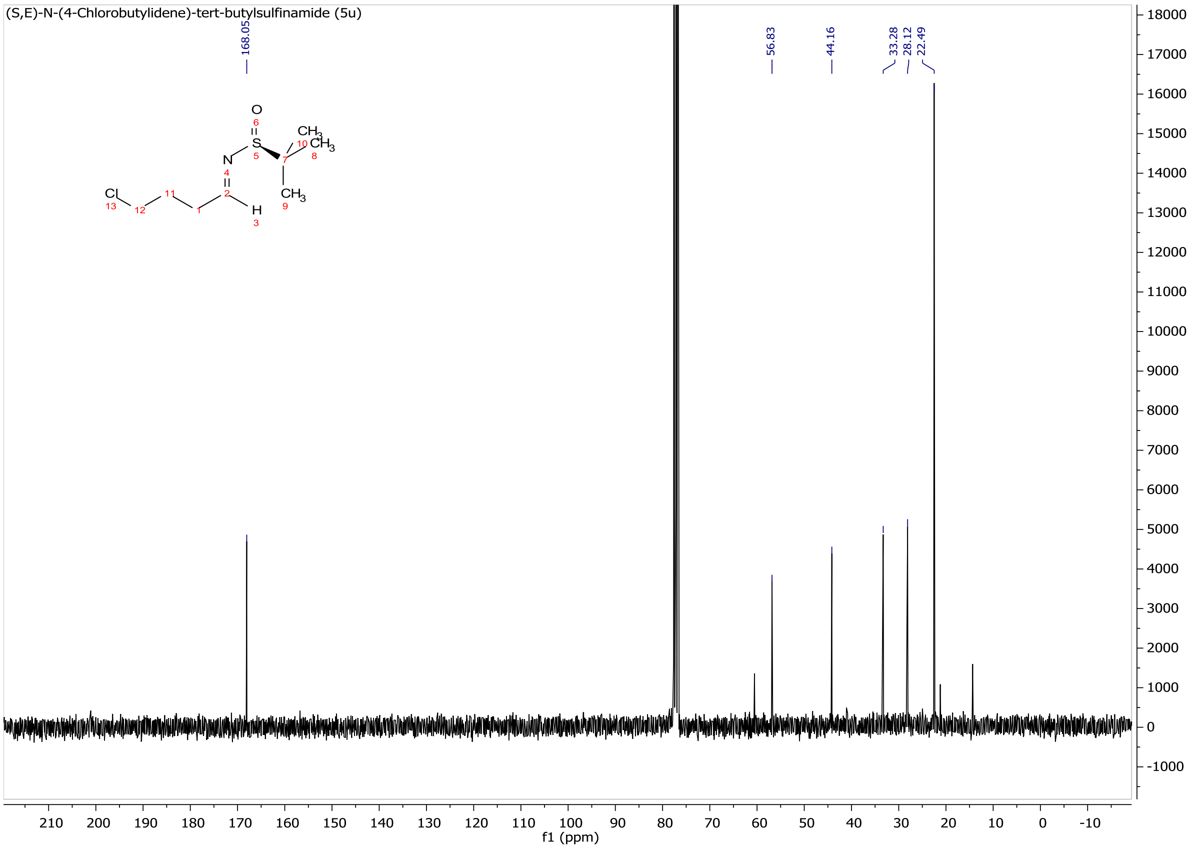
(S,E)-N-(4-Chlorobutylidene)-tert-butylsulfinamide (5u)
168.05
56.83
44.16
33.28
28.12
22.49
f1 (ppm)

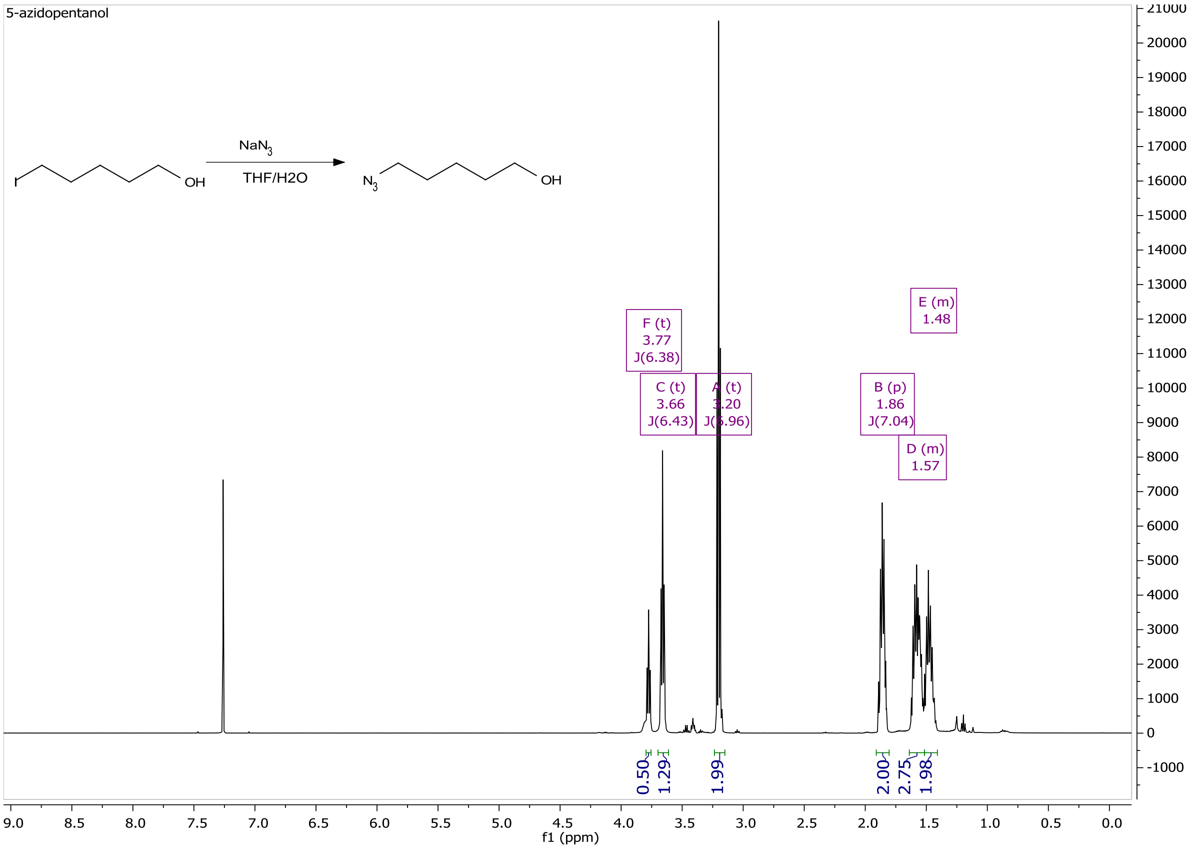
5-azidopentanol
NaN3
THF/H2O
OH
N3
OH
F (t) 3.77 J(6.38)
C (t) 3.66 J(6.43)
A (t) 3.20 J(6.96)
B (p) 1.86 J(7.04)
D (m) 1.57
E (m) 1.48
0.50
1.29
1.99
2.00
2.75
1.98
f1 (ppm)

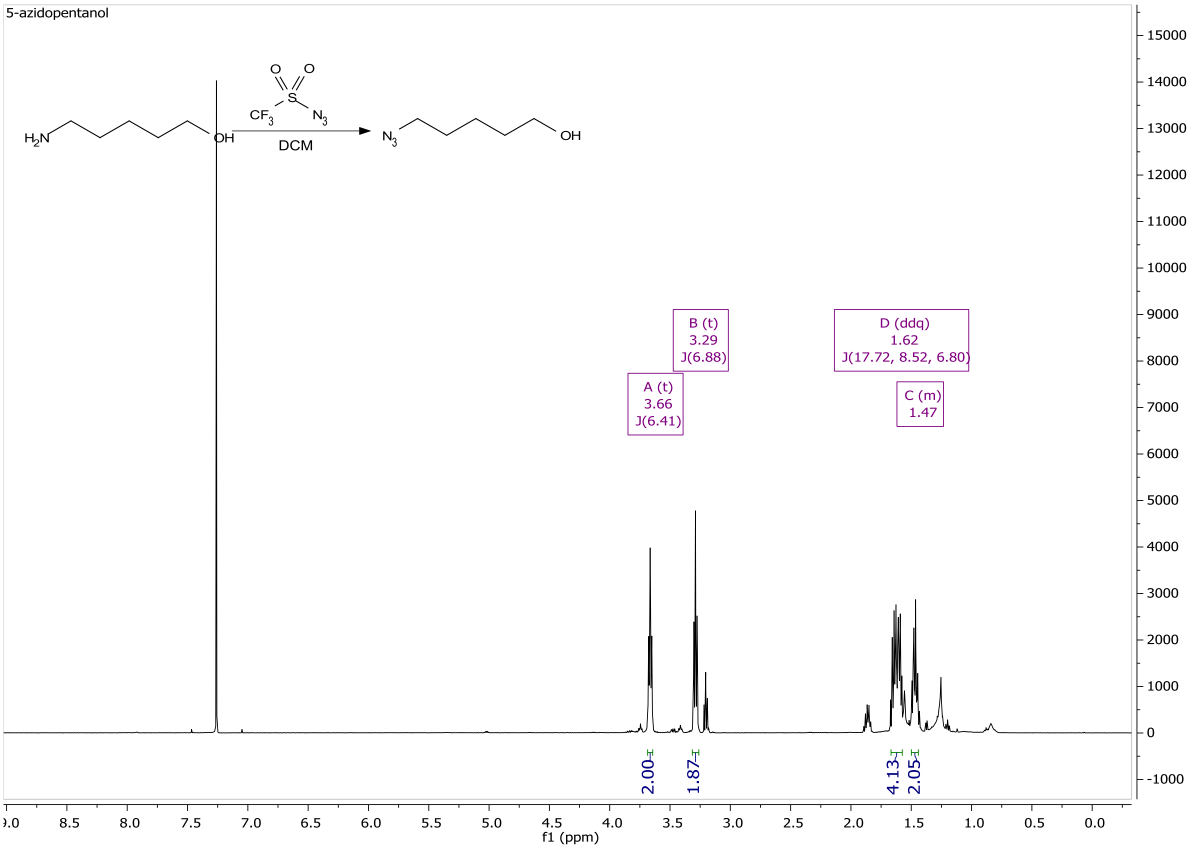
5-azidopentanol
H2N
OH
CF3
N3
DCM
N3
OH
B (t) 3.29 J(6.88)
A (t) 3.66 J(6.41)
D (ddq) 1.62 J(17.72, 8.52, 6.80)
C (m) 1.47
2.00
1.87
4.13
2.05
f1 (ppm)

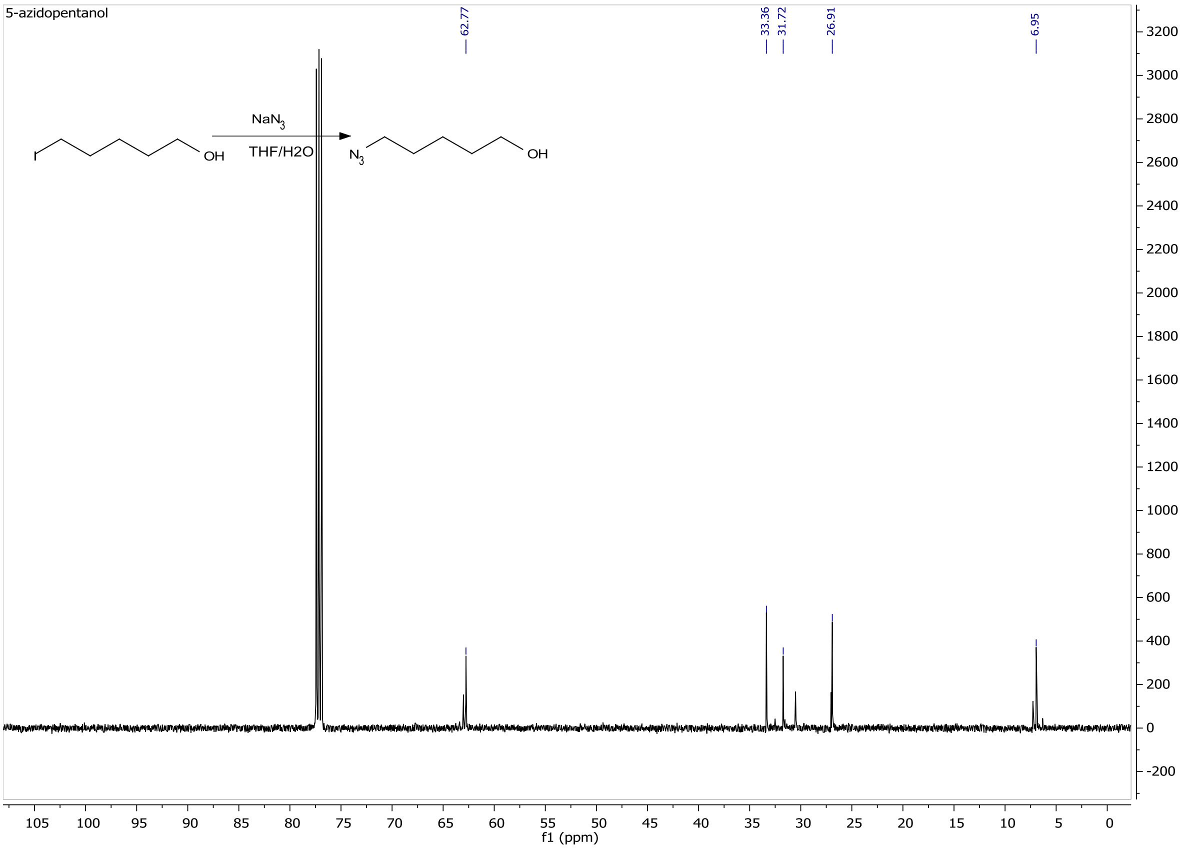
5-azidopentanol
62.77
33.36
31.72
26.91
6.95
NaN3
THF/H2O
I
OH
N3
OH
f1 (ppm)

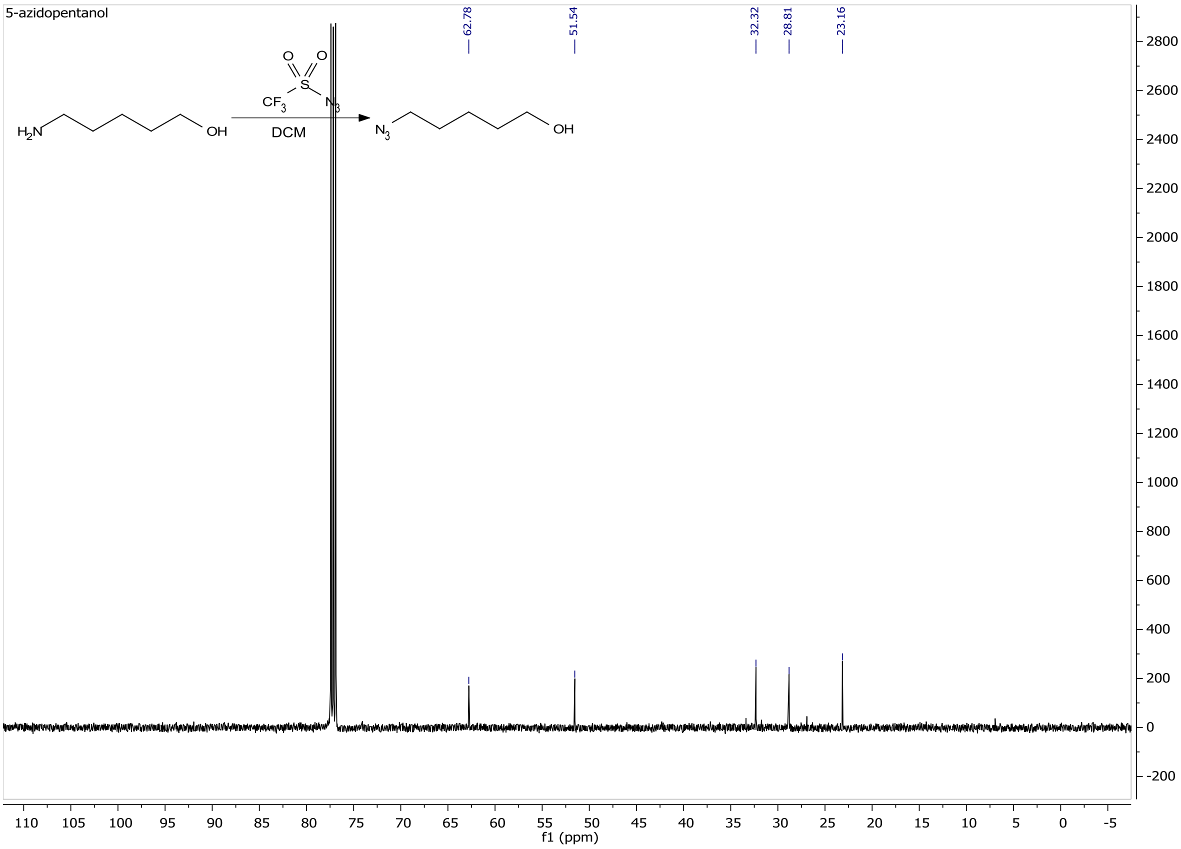
5-azidopentanol
62.78
51.54
32.32
28.81
23.16
H2N
OH
CF3
DCM
N3
OH
f1 (ppm)

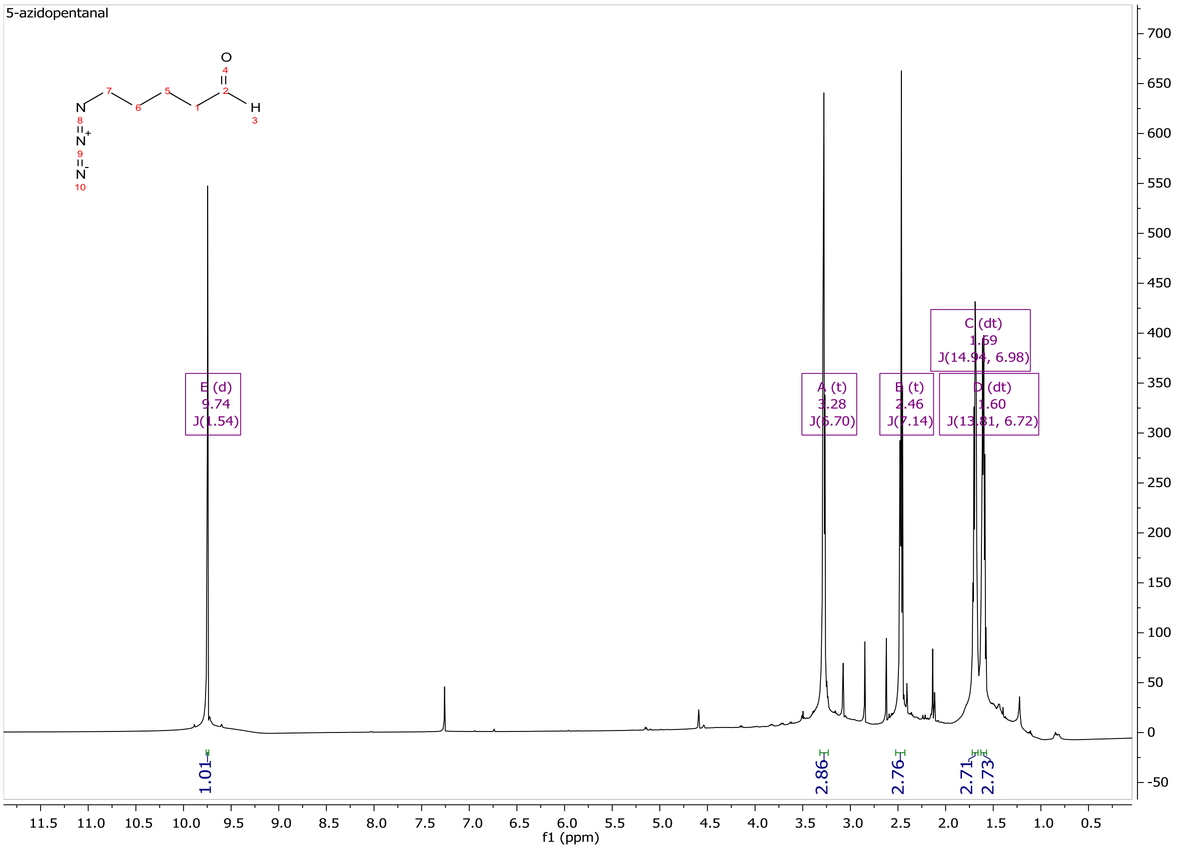
5-azidopentanal
E (d) 9.74 J(1.54)
A (t) 3.28 J(6.70)
B (t) 2.46 J(7.14)
C (dt) 1.69 J(14.94, 6.98)
D (dt) 1.60 J(13.81, 6.72)
1.01
2.86
2.76
2.71
2.73
f1 (ppm)

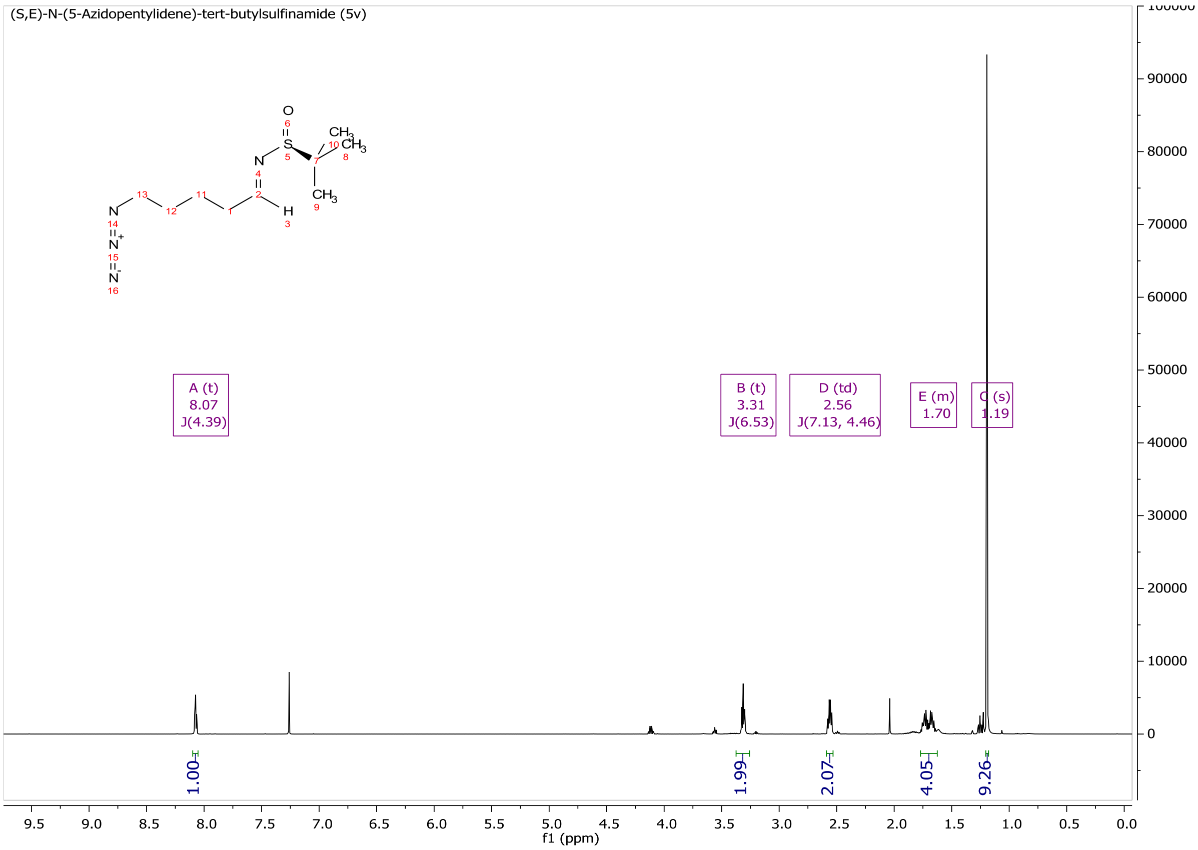
(S,E)-N-(5-Azidopentylidene)-tert-butylsulfinamide (5v)
A (t) 8.07 J(4.39)
B (t) 3.31 J(6.53)
D (td) 2.56 J(7.13, 4.46)
E (m) 1.70
C (s) 1.19
1.00
1.99
2.07
4.05
9.26
f1 (ppm)

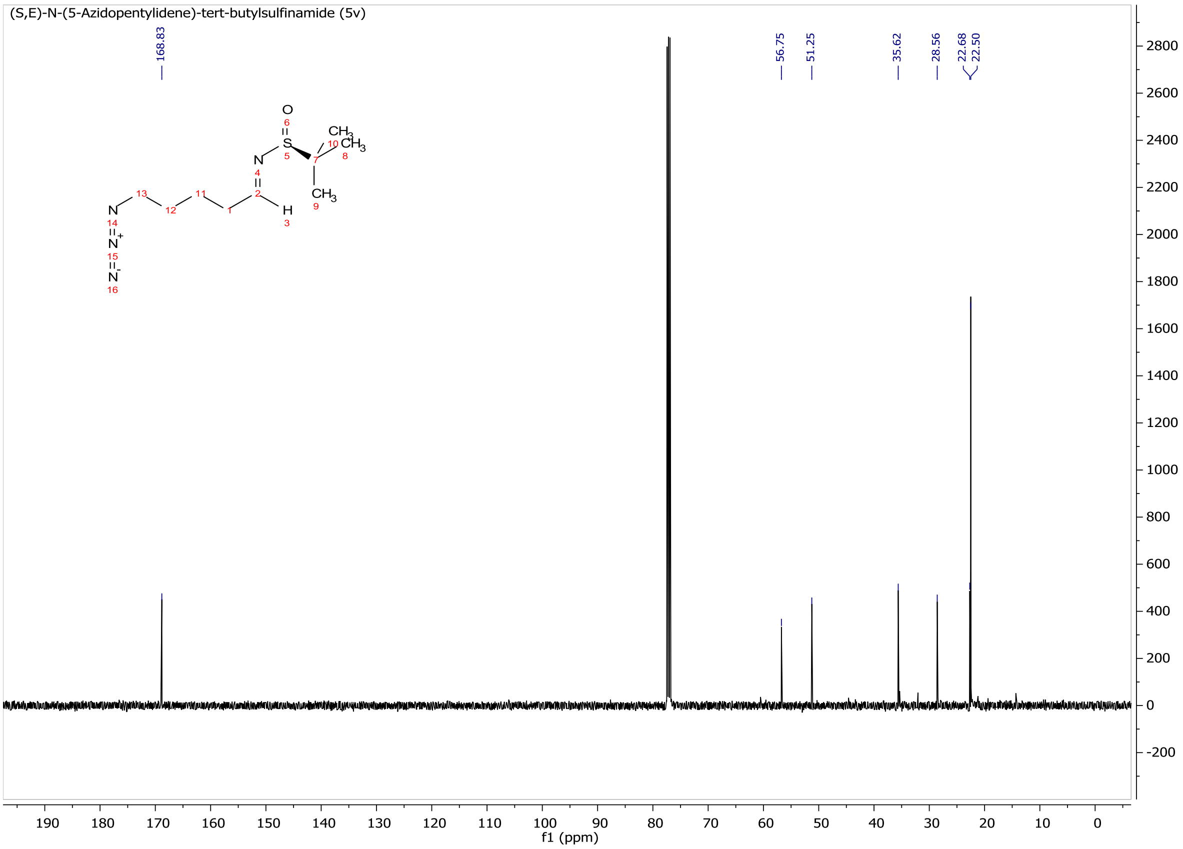
(S,E)-N-(5-Azidopentylidene)-tert-butylsulfinamide (5v)
168.83
56.75
51.25
35.62
28.56
22.68
22.50
f1 (ppm)

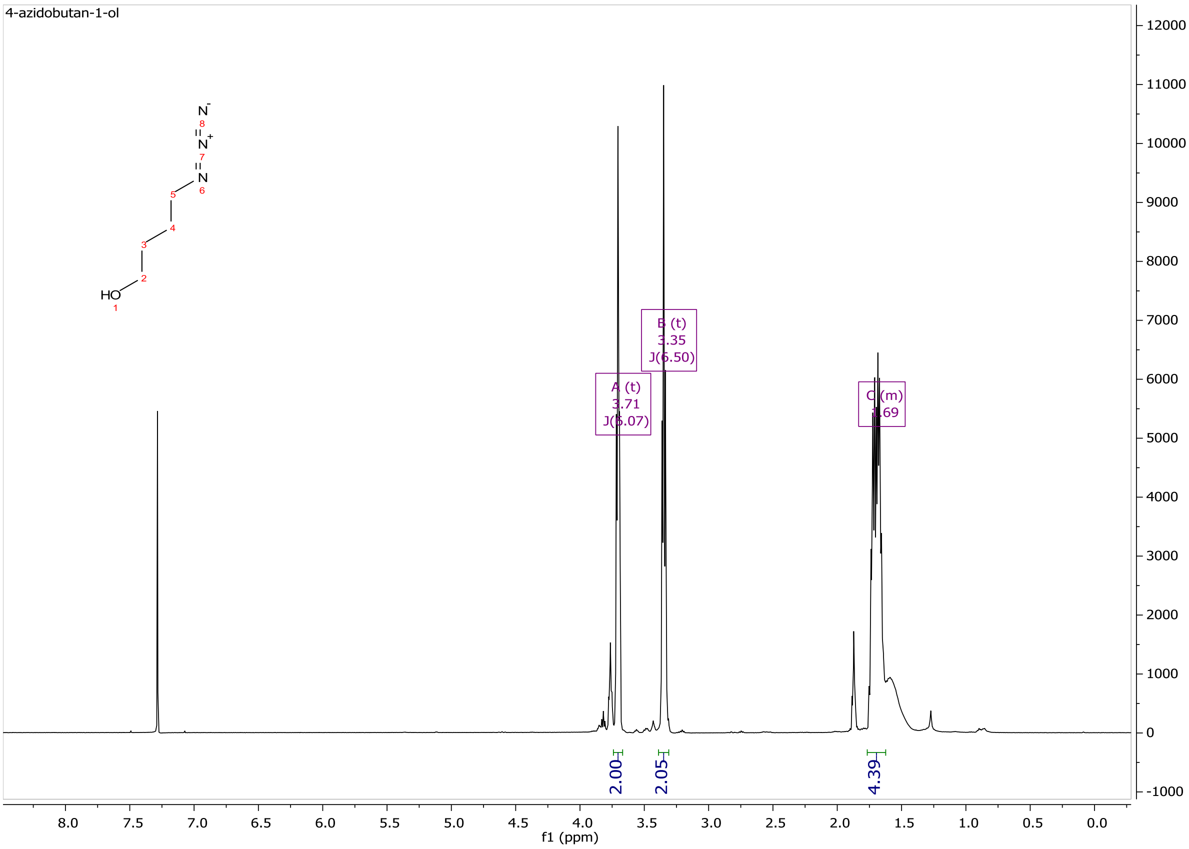
4-azidobutan-1-ol
A (t)
3.71
J(6.07)
B (t)
3.35
J(6.50)
C (m)
1.69
2.00
2.05
4.39
f1 (ppm)

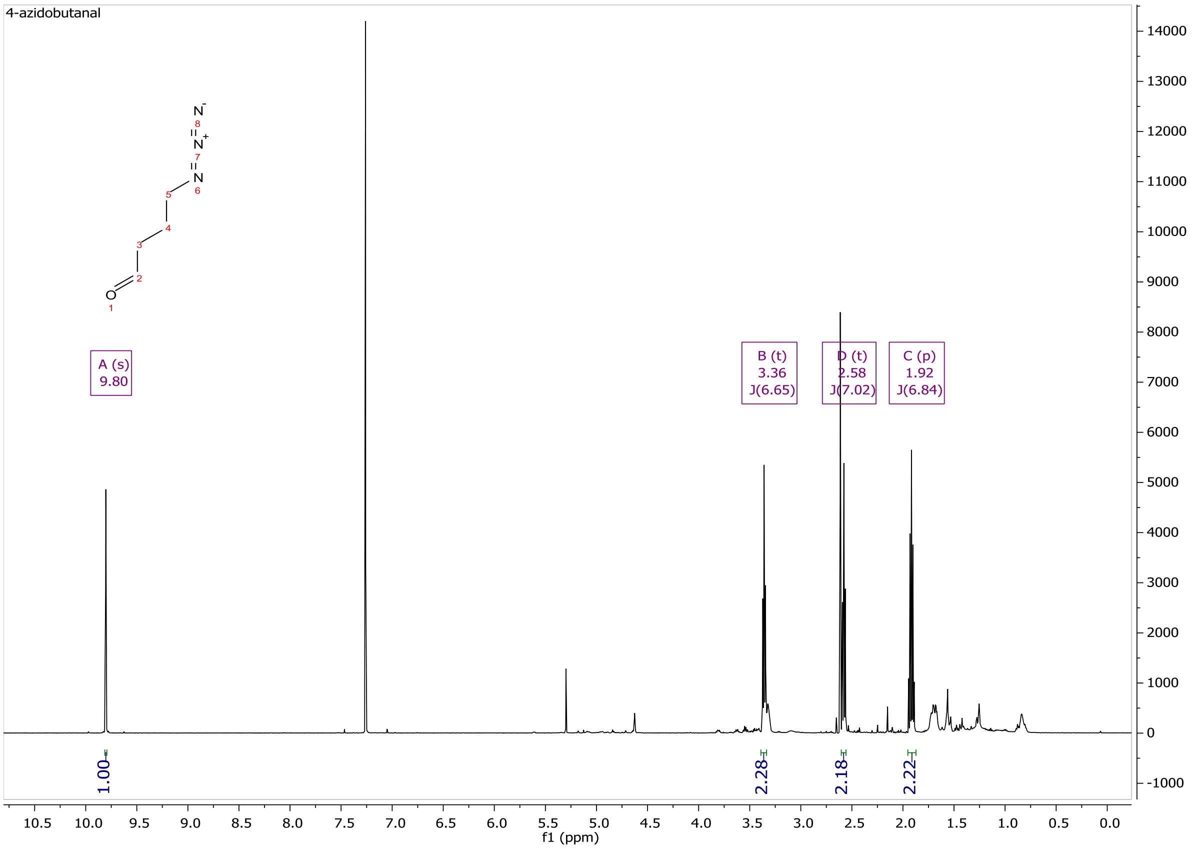
4-azidobutanal
A (s)
9.80
B (t)
3.36
J(6.65)
D (t)
2.58
J(7.02)
C (p)
1.92
J(6.84)
1.00
2.28
2.18
2.22
f1 (ppm)

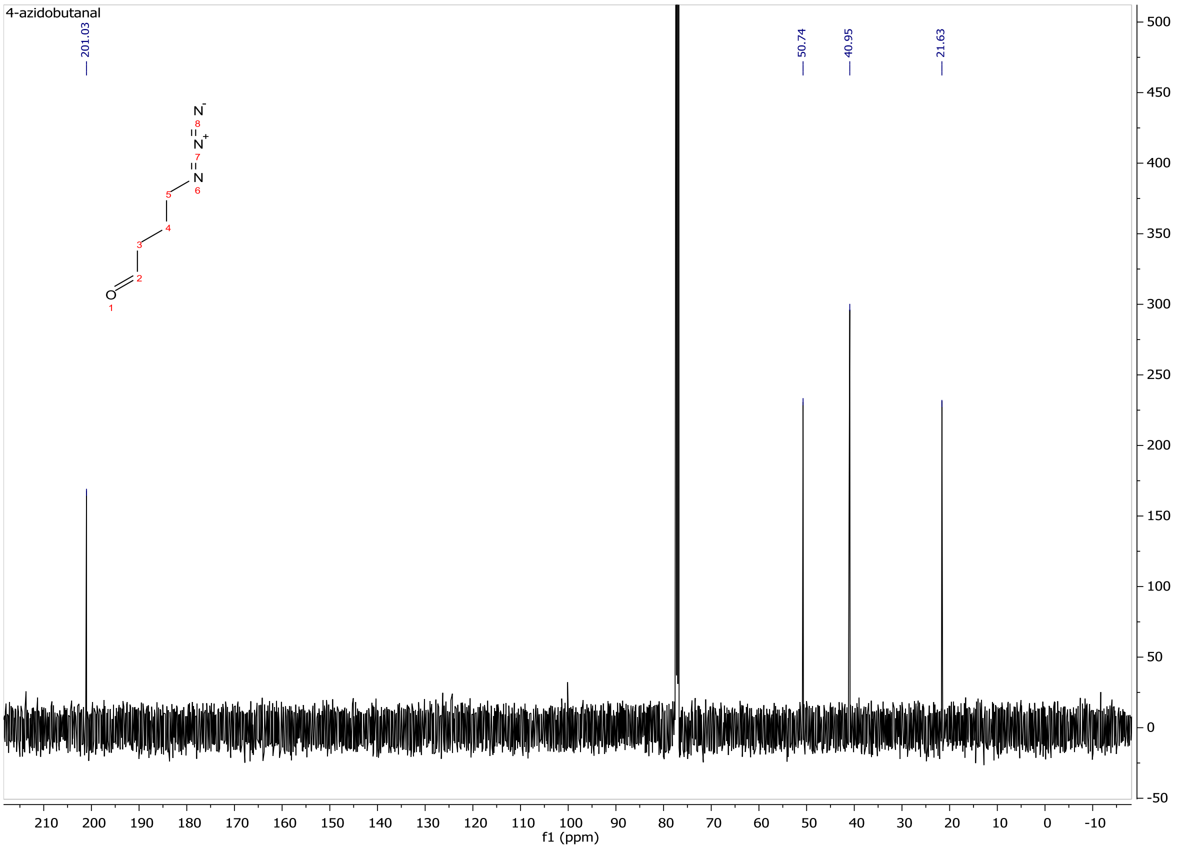
4-azidobutanal
201.03
50.74
40.95
21.63
f1 (ppm)

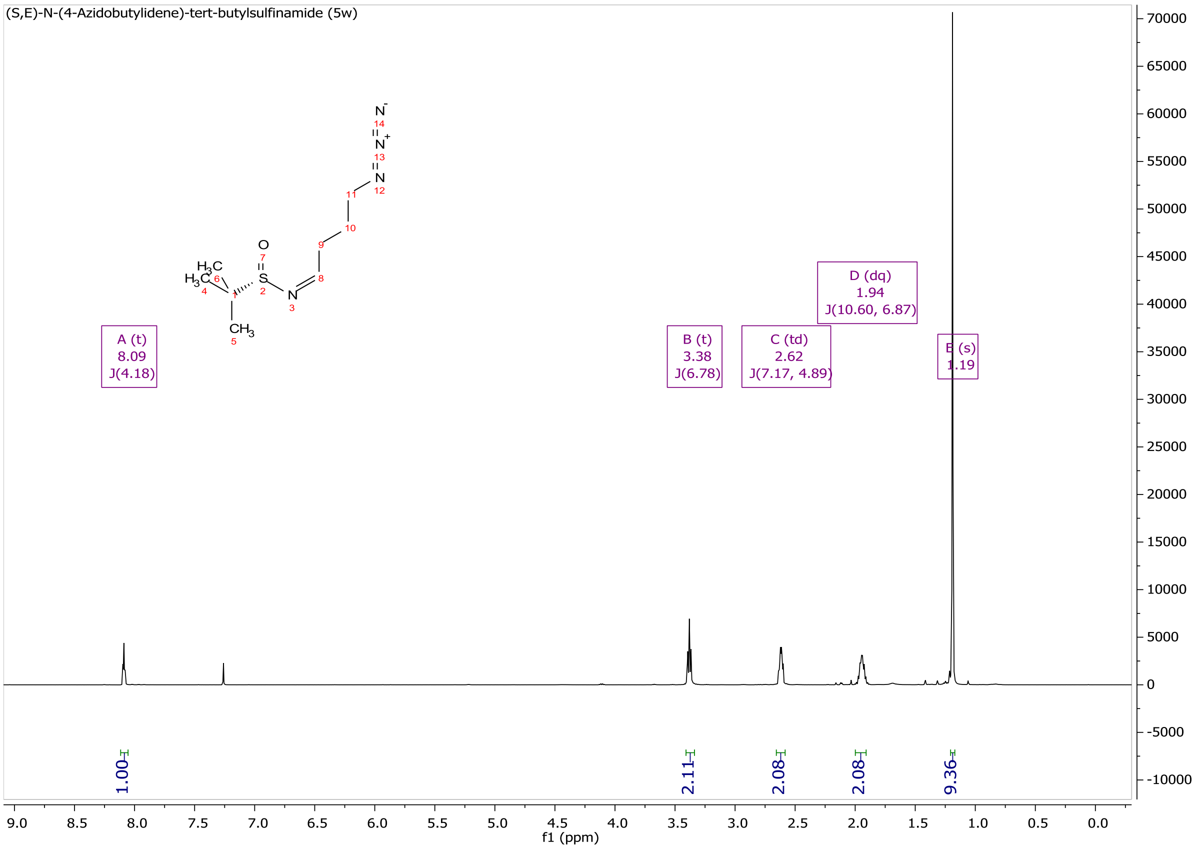
(S,E)-N-(4-Azidobutylidene)-tert-butylsulfinamide (5w)
A (t)
8.09
J(4.18)
B (t)
3.38
J(6.78)
C (td)
2.62
J(7.17, 4.89)
D (dq)
1.94
J(10.60, 6.87)
E (s)
1.19
1.00
2.11
2.08
2.08
9.36
f1 (ppm)

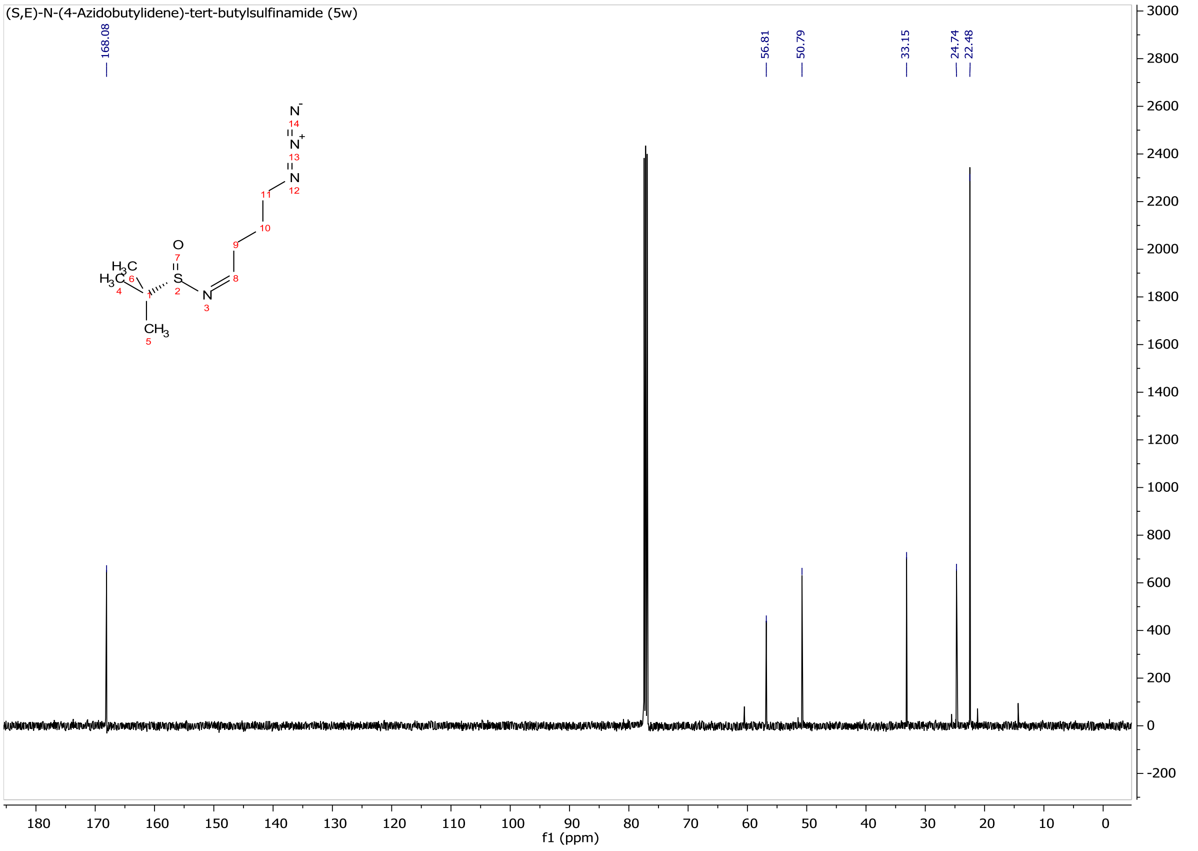
(S,E)-N-(4-Azidobutylidene)-tert-butylsulfinamide (5w)
168.08
56.81
50.79
33.15
24.74
22.48
f1 (ppm)

Trimethylsilyl protected propargylamines **6**

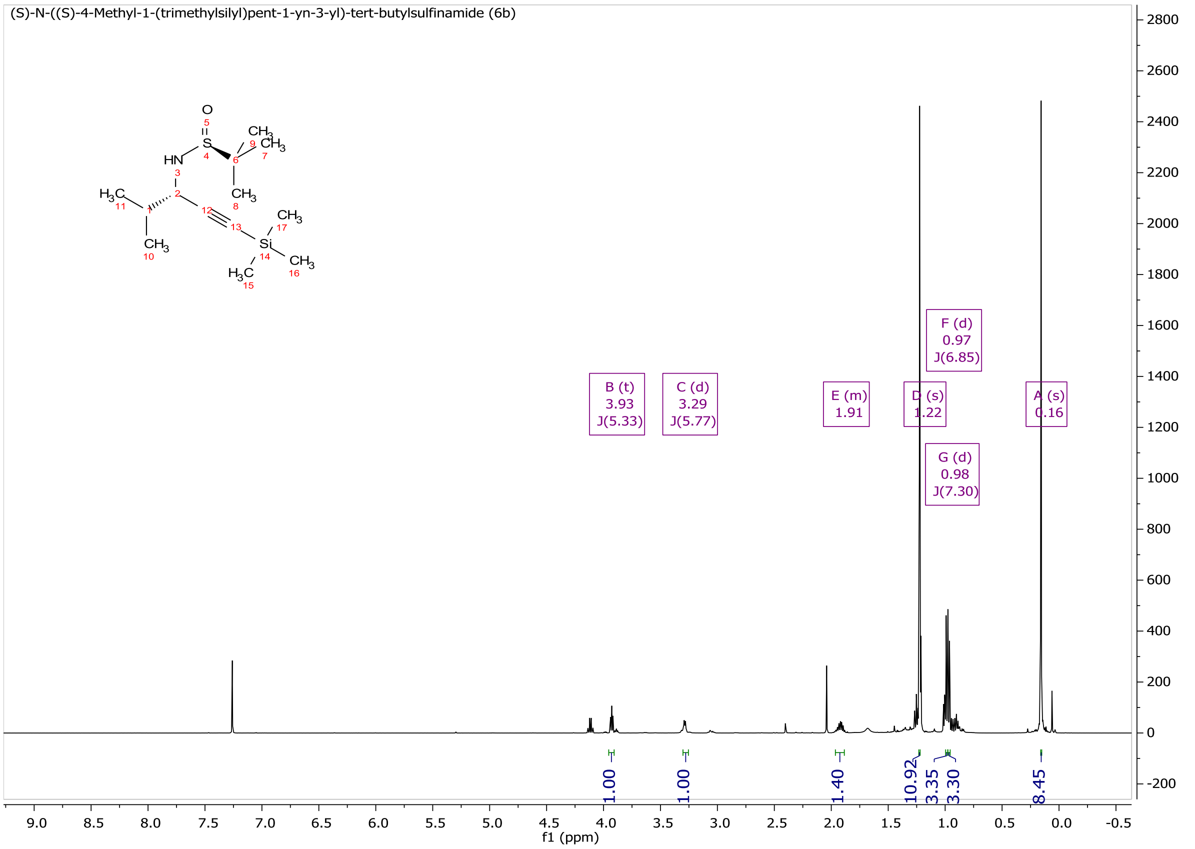

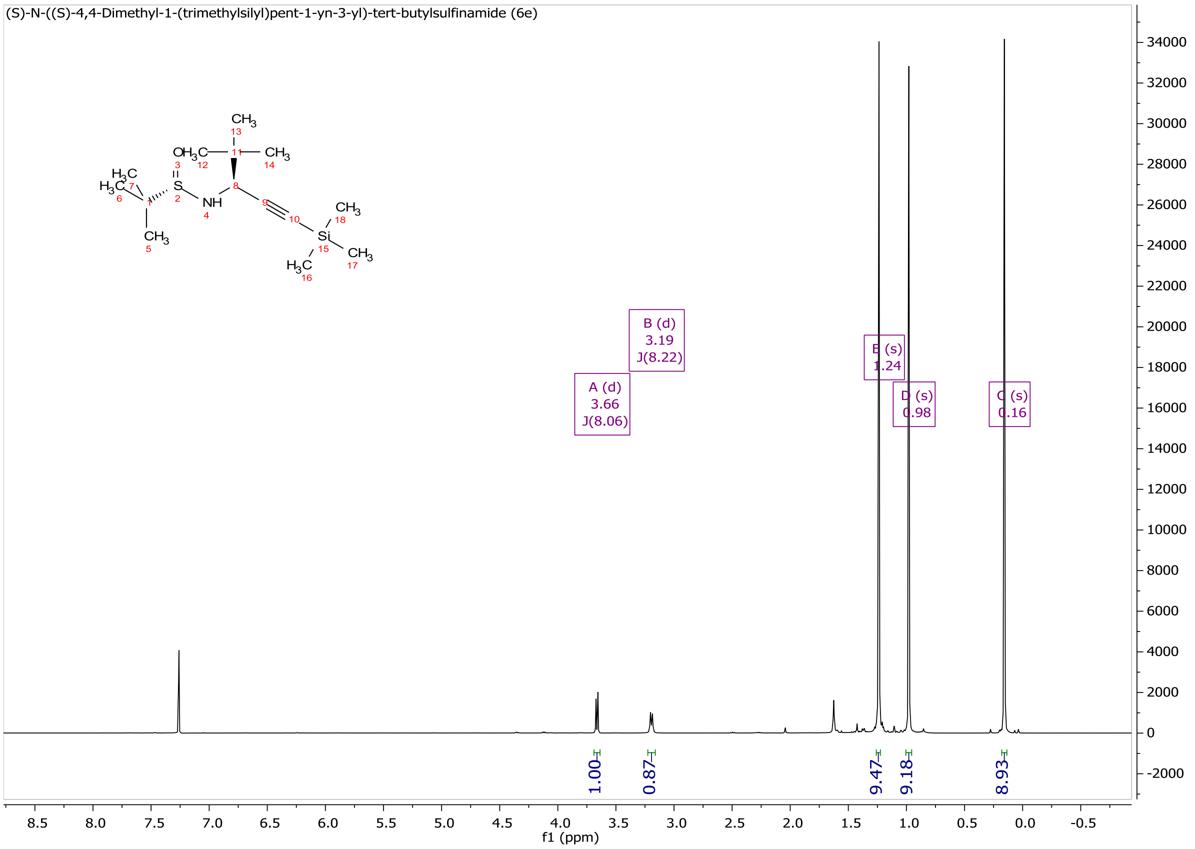

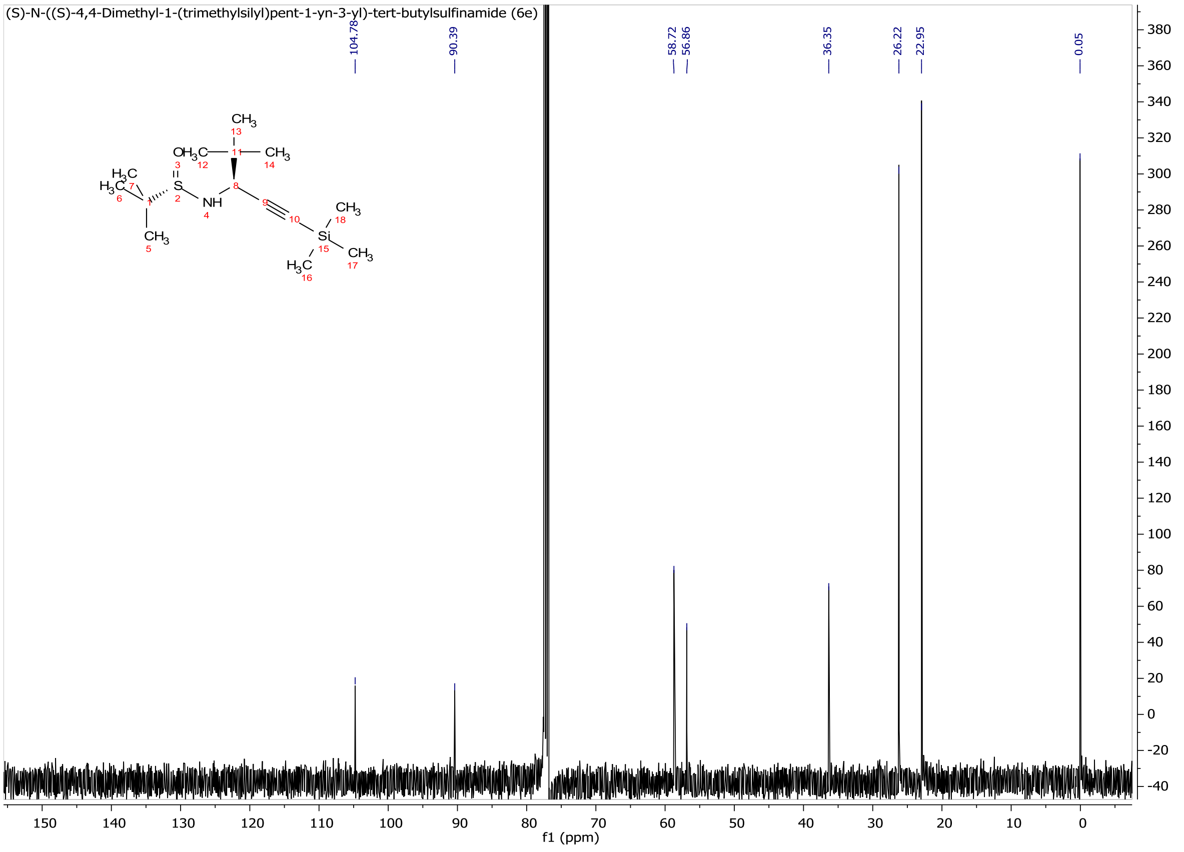
(S)-N-((S)-4,4-Dimethyl-1-(trimethylsilyl)pent-1-yn-3-yl)-tert-butylsulfinamide (6e)
104.78
90.39
58.72
56.86
36.35
26.22
22.95
0.05
f1 (ppm)

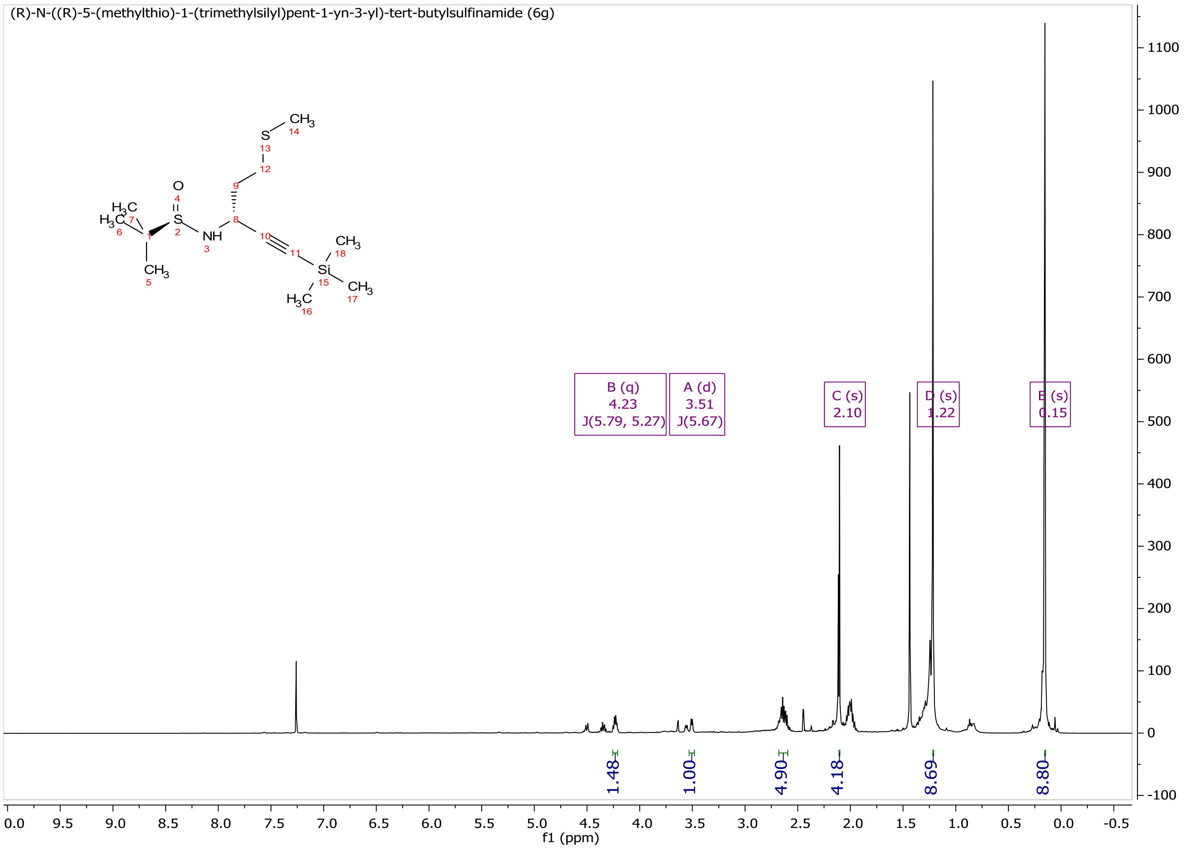
(R)-N-((R)-5-(methylthio)-1-(trimethylsilyl)pent-1-yn-3-yl)-tert-butylsulfinamide (6g)
B (q)
4.23
J(5.79, 5.27)
A (d)
3.51
J(5.67)
C (s)
2.10
D (s)
1.22
E (s)
0.15
1.48
1.00
4.90
4.18
8.69
8.80
f1 (ppm)

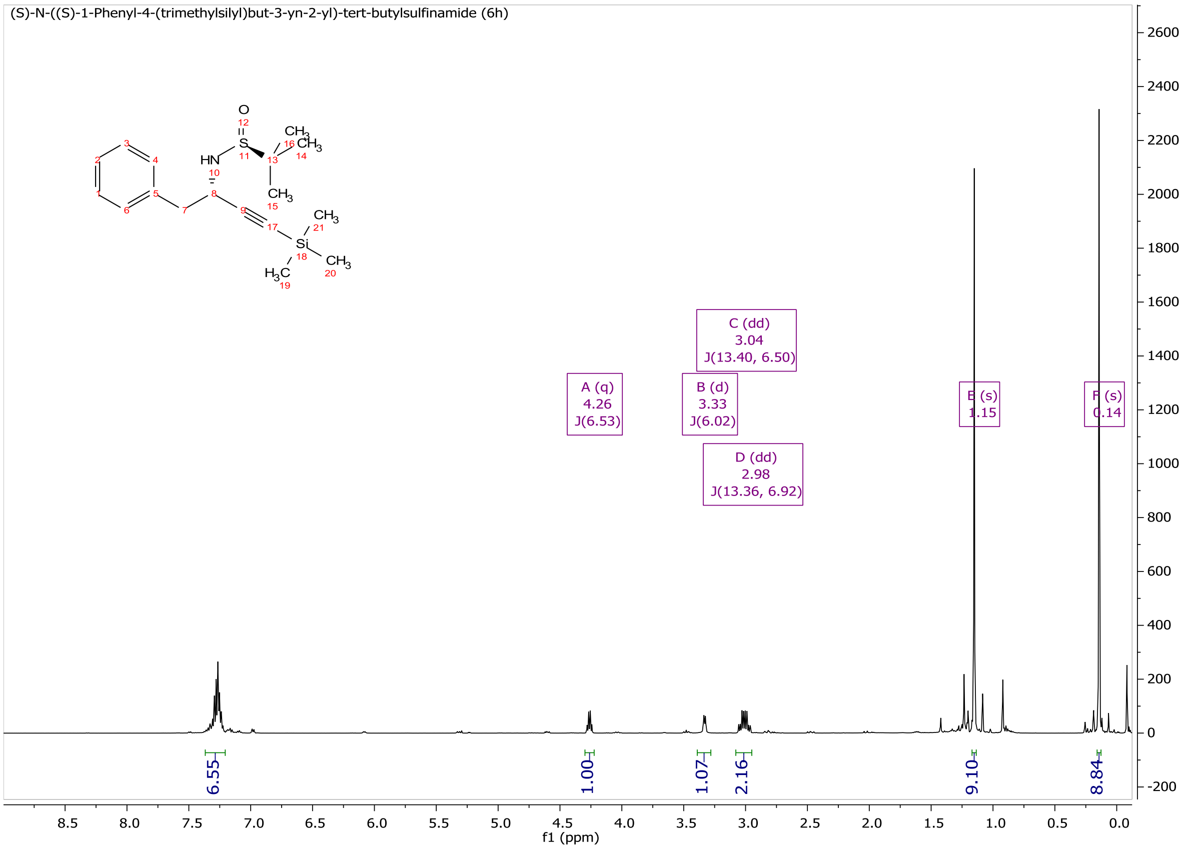
(S)-N-((S)-1-Phenyl-4-(trimethylsilyl)but-3-yn-2-yl)-tert-butylsulfinamide (6h)
A (q)
4.26
J(6.53)
B (d)
3.33
J(6.02)
C (dd)
3.04
J(13.40, 6.50)
D (dd)
2.98
J(13.36, 6.92)
E (s)
1.15
F (s)
0.14
6.55
1.00
1.07
2.16
9.10
8.84
f1 (ppm)

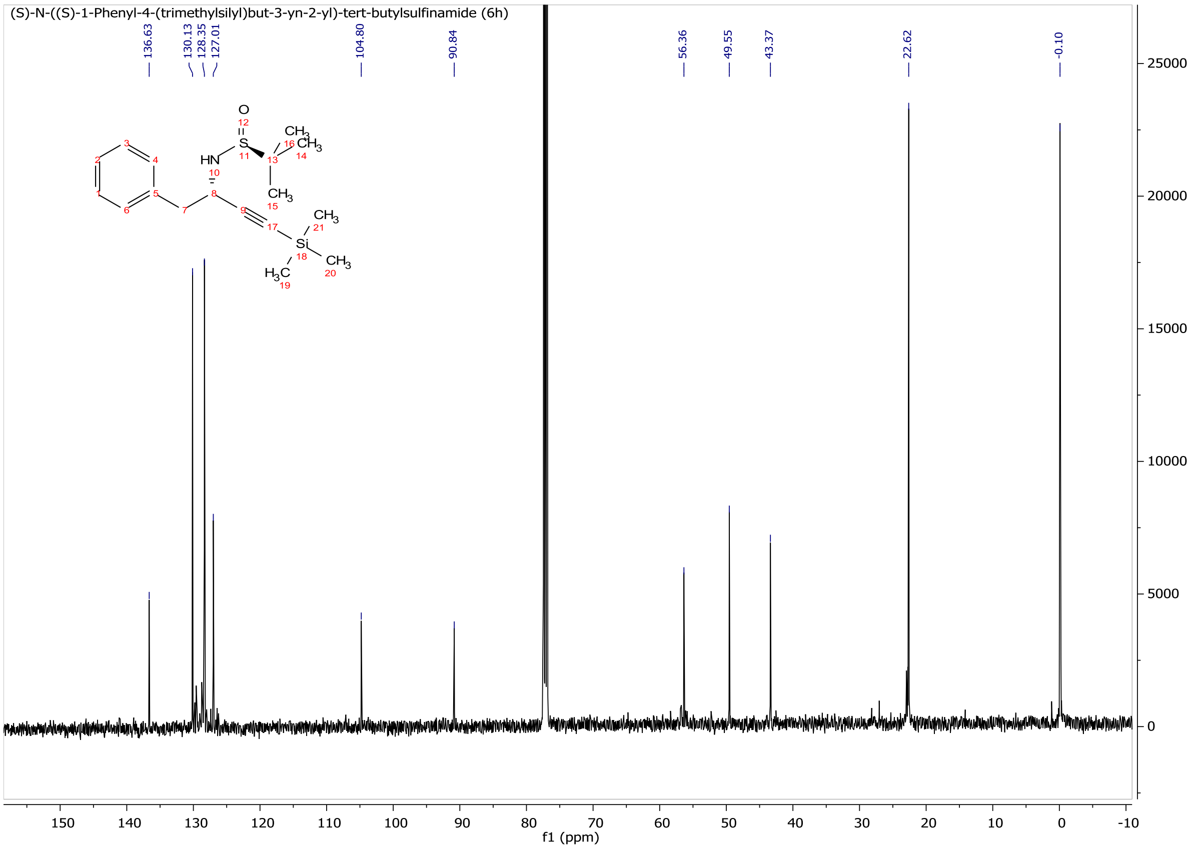
(S)-N-((S)-1-Phenyl-4-(trimethylsilyl)but-3-yn-2-yl)-tert-butylsulfinamide (6h)
136.63
130.13
128.35
127.01
104.80
90.84
56.36
49.55
43.37
22.62
-0.10
f1 (ppm)

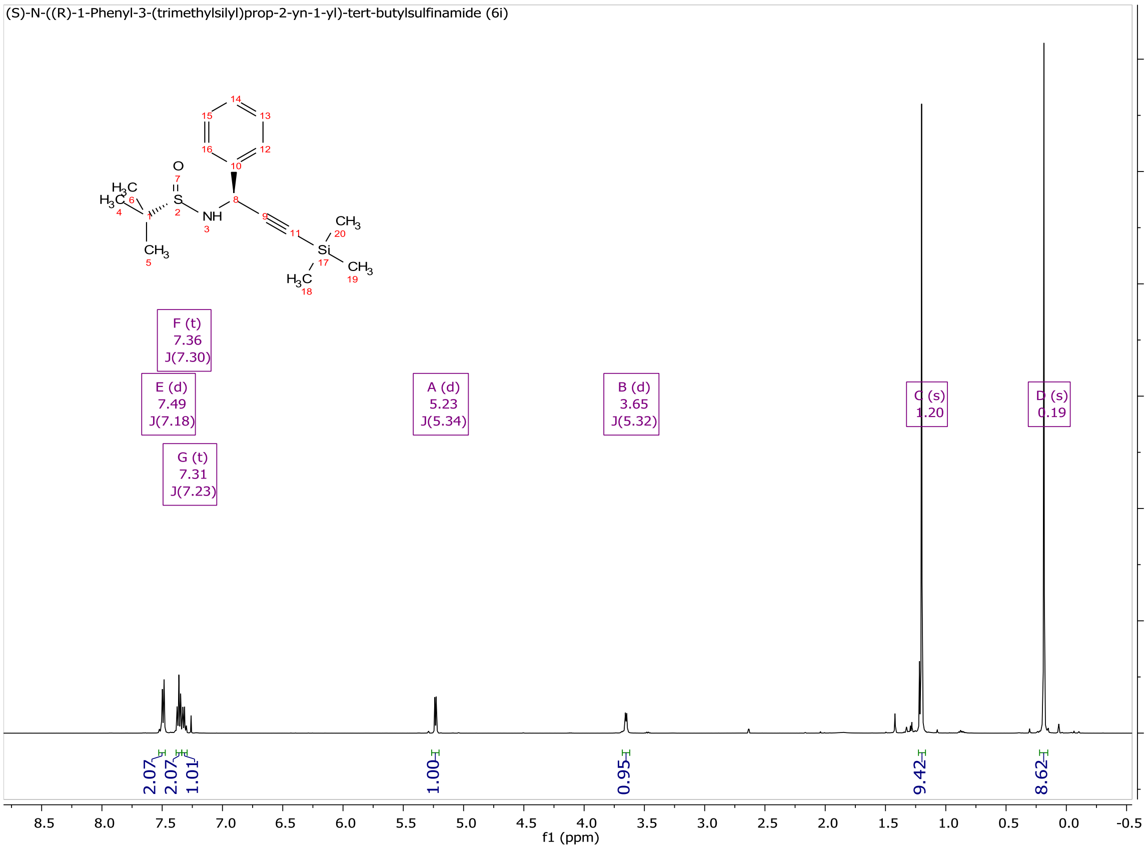
(S)-N-((R)-1-Phenyl-3-(trimethylsilyl)prop-2-yn-1-yl)-tert-butylsulfinamide (6i)
F (t) 7.36 J(7.30)
E (d) 7.49 J(7.18)
G (t) 7.31 J(7.23)
A (d) 5.23 J(5.34)
B (d) 3.65 J(5.32)
C (s) 1.20
D (s) 0.19
2.07 2.07 1.01 1.00 0.95 9.42 8.62
f1 (ppm)

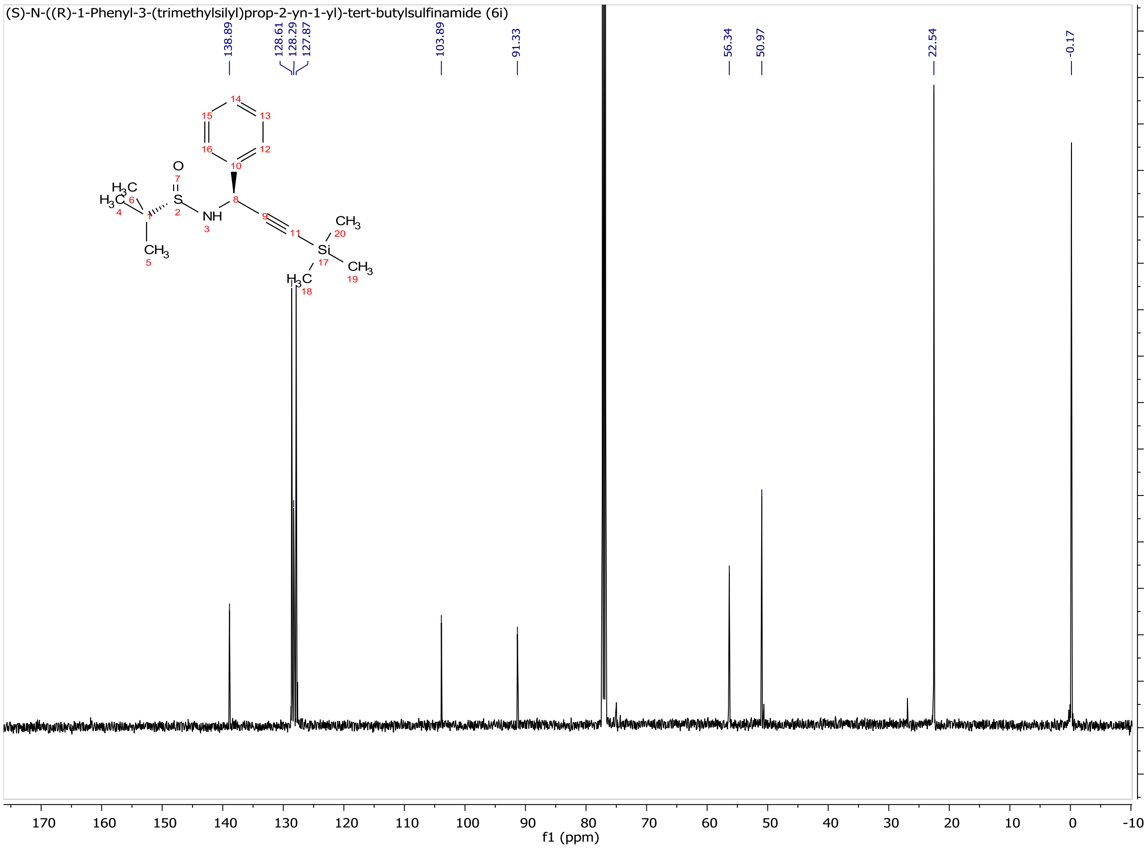
(S)-N-((R)-1-Phenyl-3-(trimethylsilyl)prop-2-yn-1-yl)-tert-butylsulfinamide (6i)
138.89
128.61 128.29 127.87
103.89
91.33
56.34
50.97
22.54
-0.17
f1 (ppm)

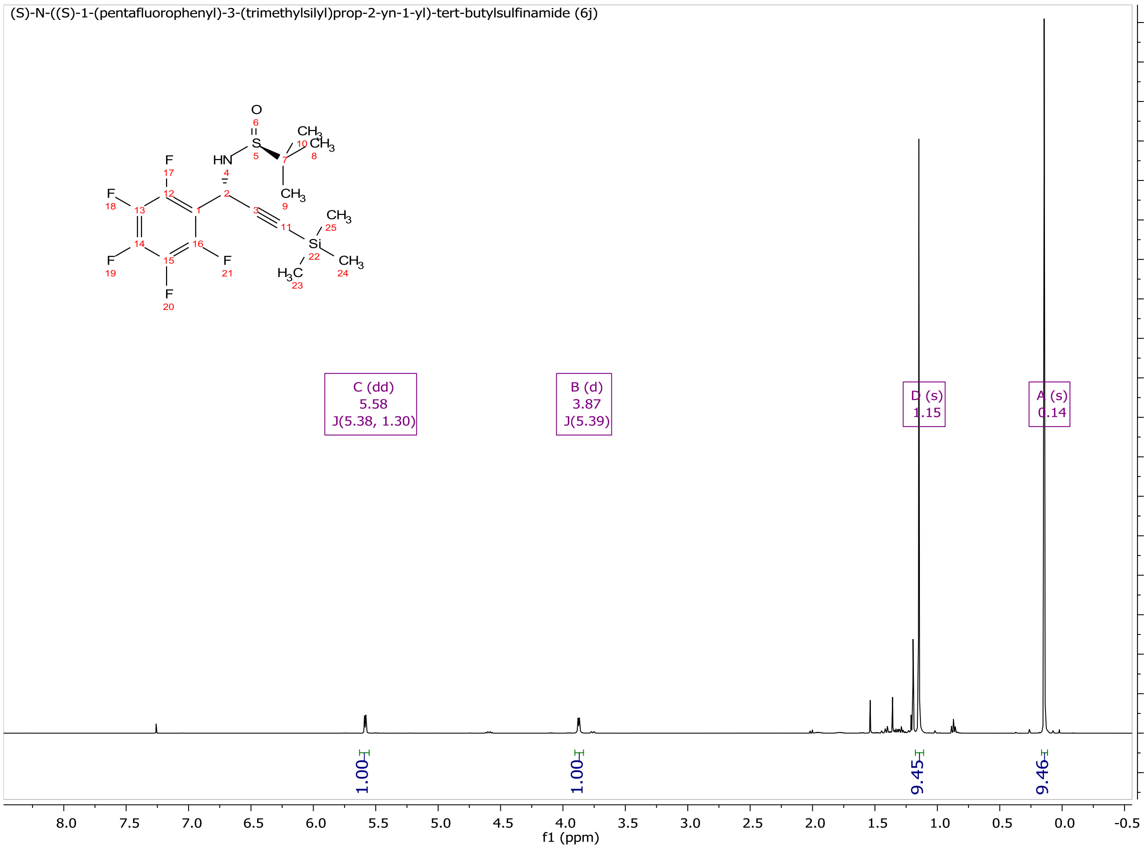
(S)-N-((S)-1-(pentafluorophenyl)-3-(trimethylsilyl)prop-2-yn-1-yl)-tert-butylsulfinamide (6j)
C (dd)
5.58
J(5.38, 1.30)
B (d)
3.87
J(5.39)
D (s)
1.15
A (s)
0.14
1.00
1.00
9.45
9.46
f1 (ppm)

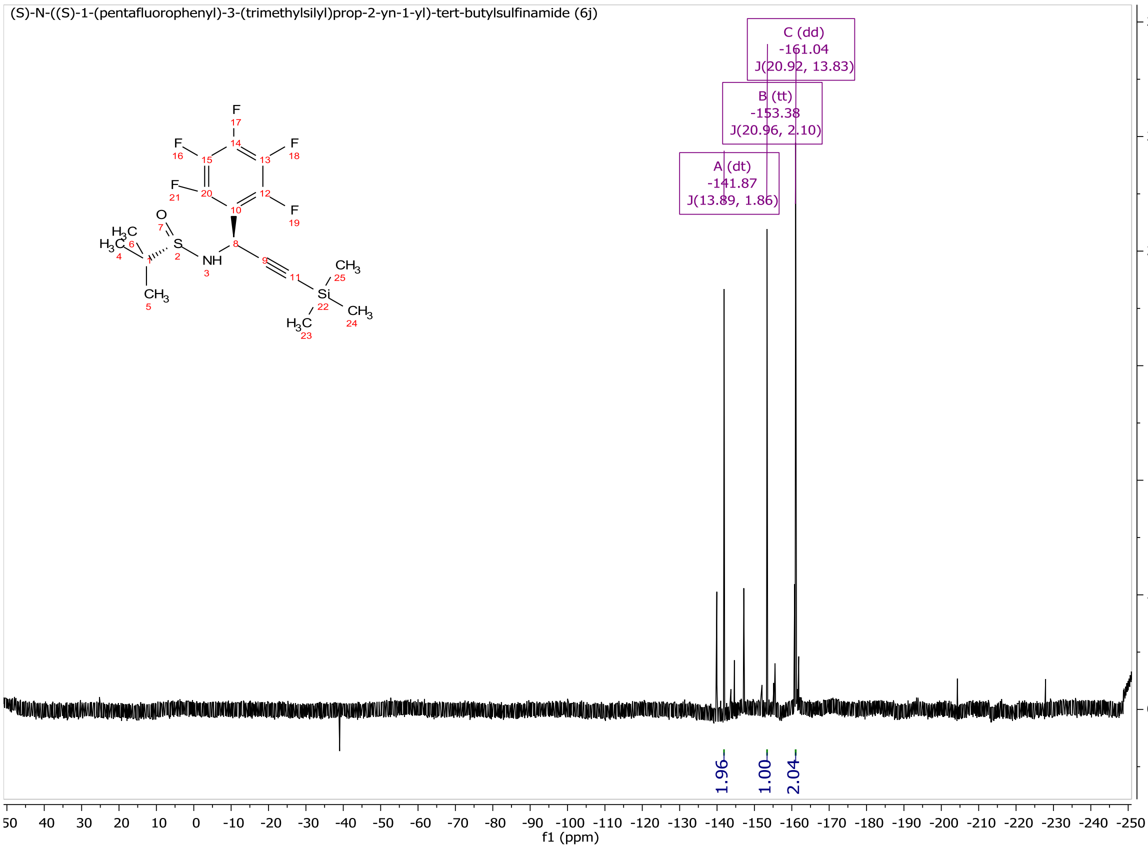
(S)-N-((S)-1-(pentafluorophenyl)-3-(trimethylsilyl)prop-2-yn-1-yl)-tert-butylsulfinamide (6j)
C (dd)
-161.04
J(20.92, 13.83)
B (tt)
-153.38
J(20.96, 2.10)
A (dt)
-141.87
J(13.89, 1.86)
1.96
1.00
2.04
f1 (ppm)

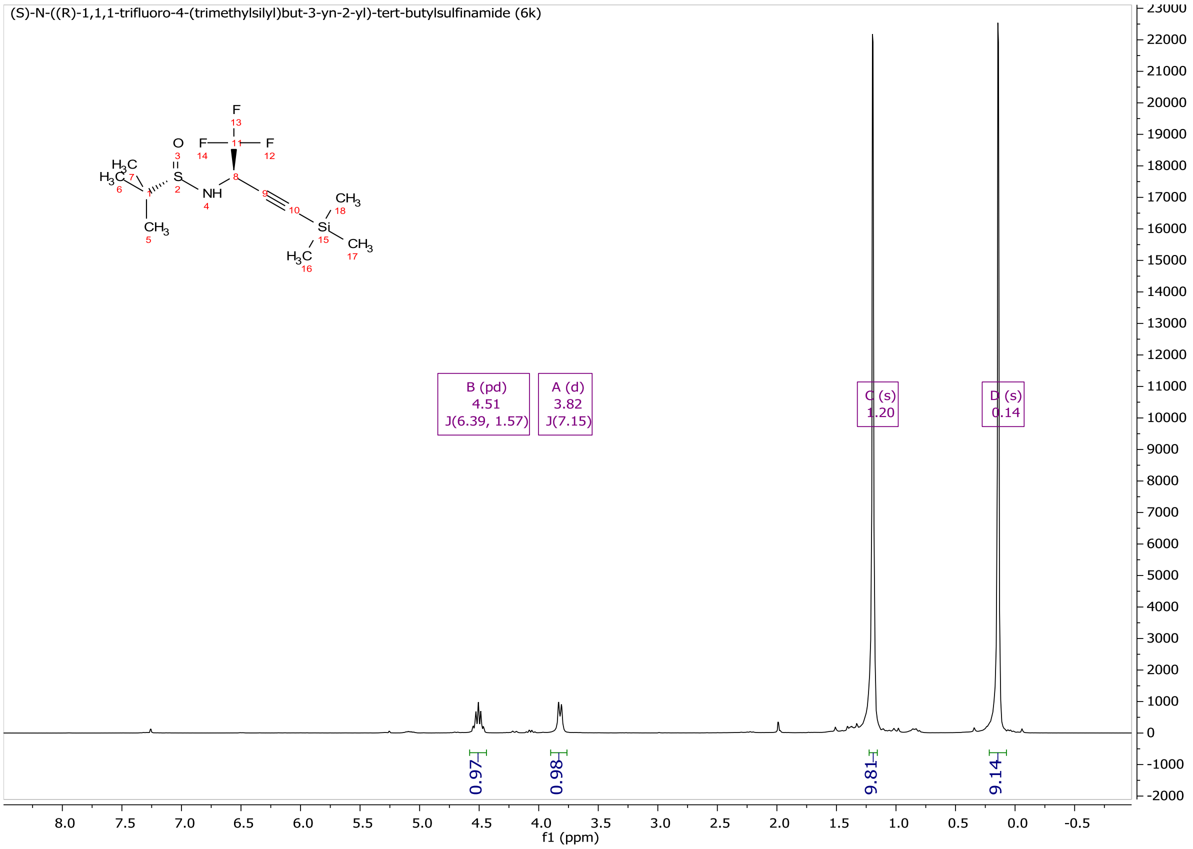
(S)-N-((R)-1,1,1-trifluoro-4-(trimethylsilyl)but-3-yn-2-yl)-tert-butylsulfinamide (6k)
B (pd)
4.51
J(6.39, 1.57)
A (d)
3.82
J(7.15)
C (s)
1.20
D (s)
0.14
0.97
0.98
9.81
9.14
f1 (ppm)

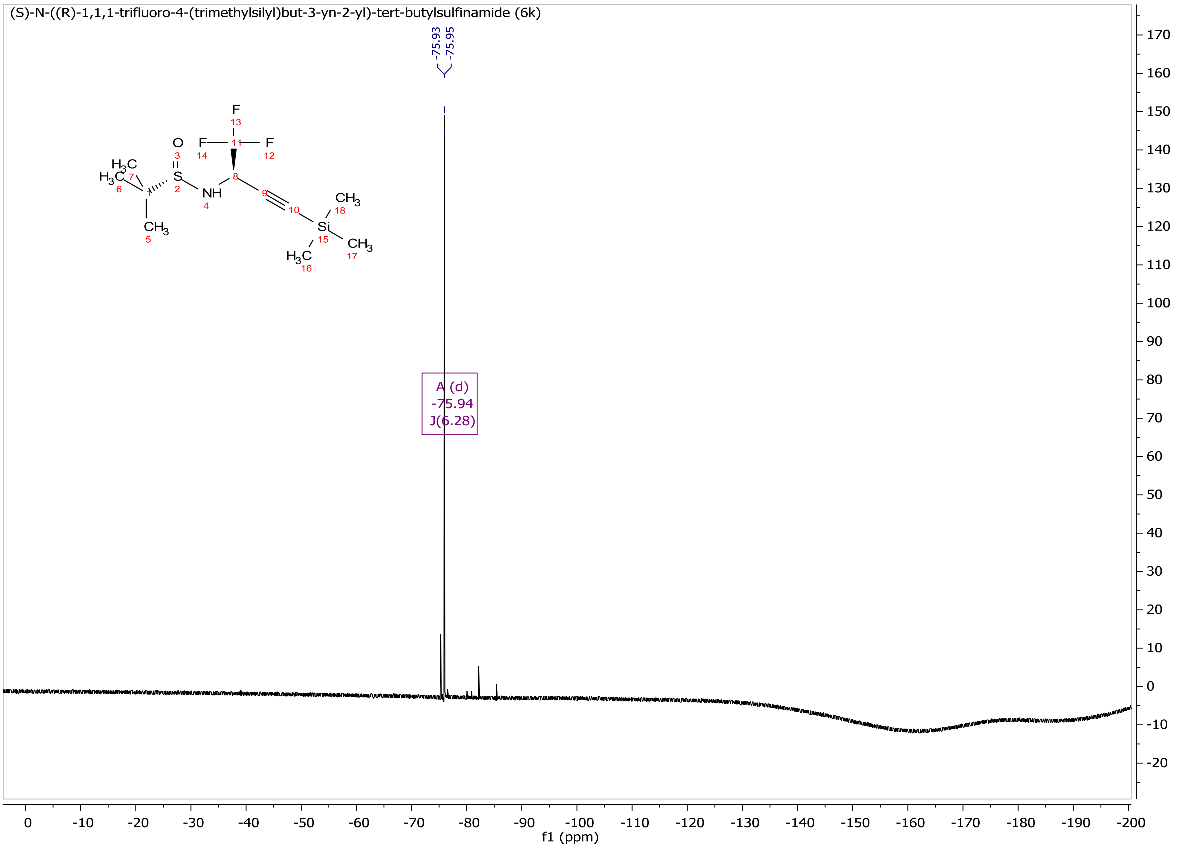
(S)-N-((R)-1,1,1-trifluoro-4-(trimethylsilyl)but-3-yn-2-yl)-tert-butylsulfinamide (6k)
-75.93
-75.95
A (d)
-75.94
J(6.28)
f1 (ppm)

(S)-N-((R)-1,1,1-trichloro-4-(trimethylsilyl)but-3-yn-2-yl)-tert-butylsulfinamide (6l)

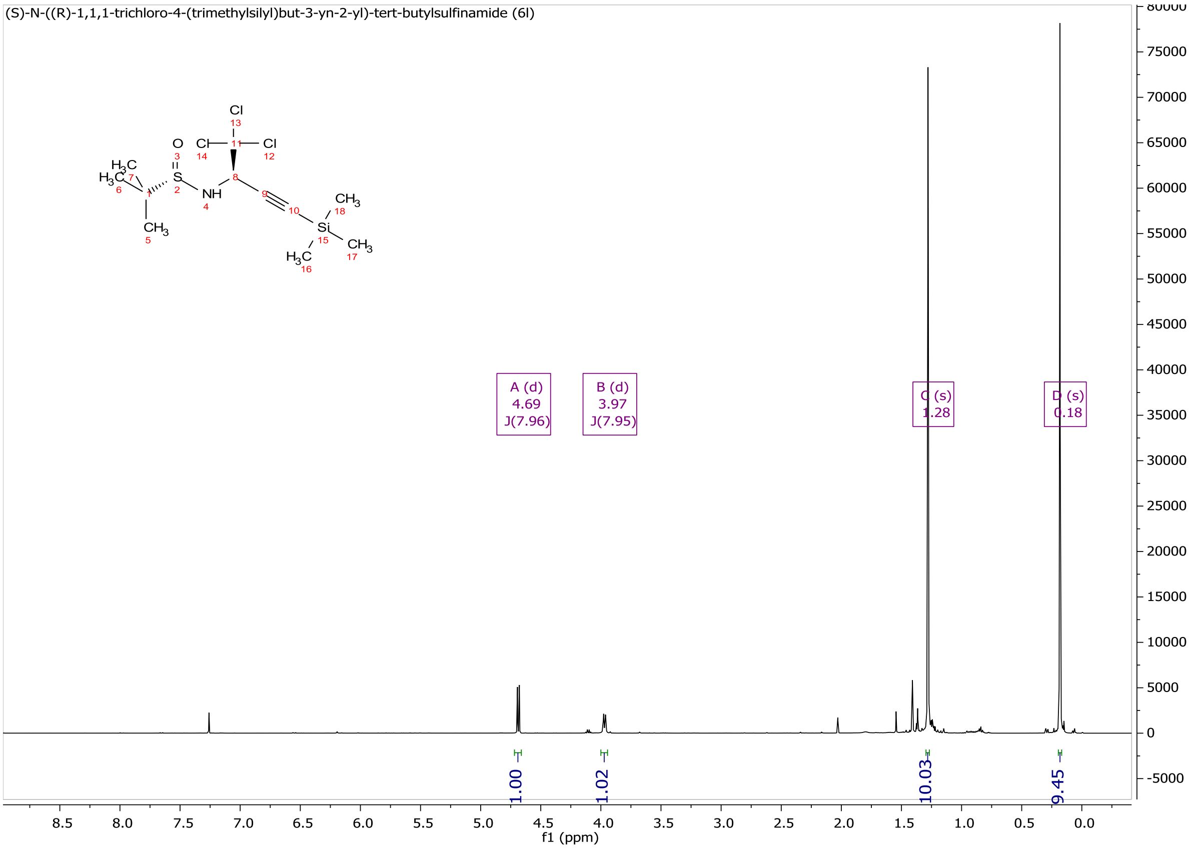

(S)-N-((R)-1,1,1-trichloro-4-(trimethylsilyl)but-3-yn-2-yl)-tert-butylsulfinamide (6l)

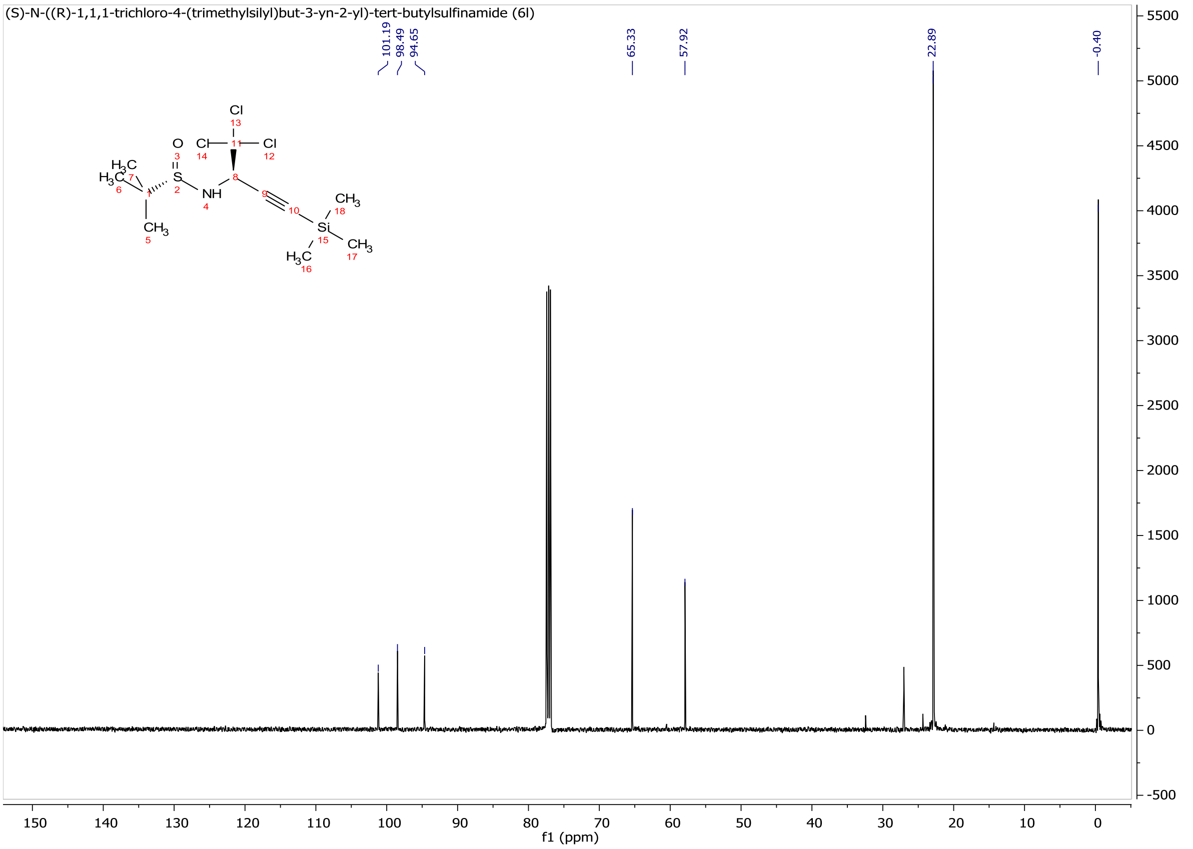

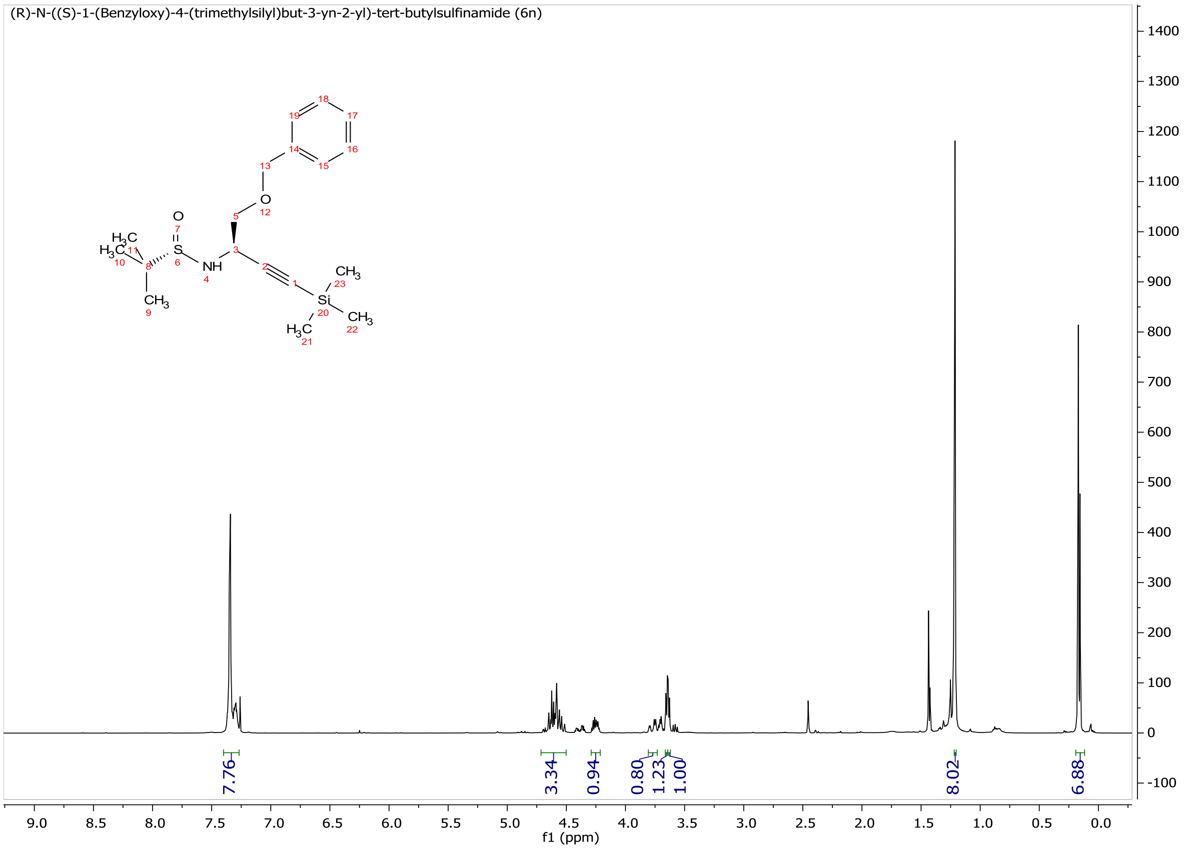
(R)-N-((S)-1-(Benzyloxy)-4-(trimethylsilyl)but-3-yn-2-yl)-tert-butylsulfinamide (6n)
7.76
3.34
0.94
0.80
1.23
1.00
8.02
6.88
f1 (ppm)

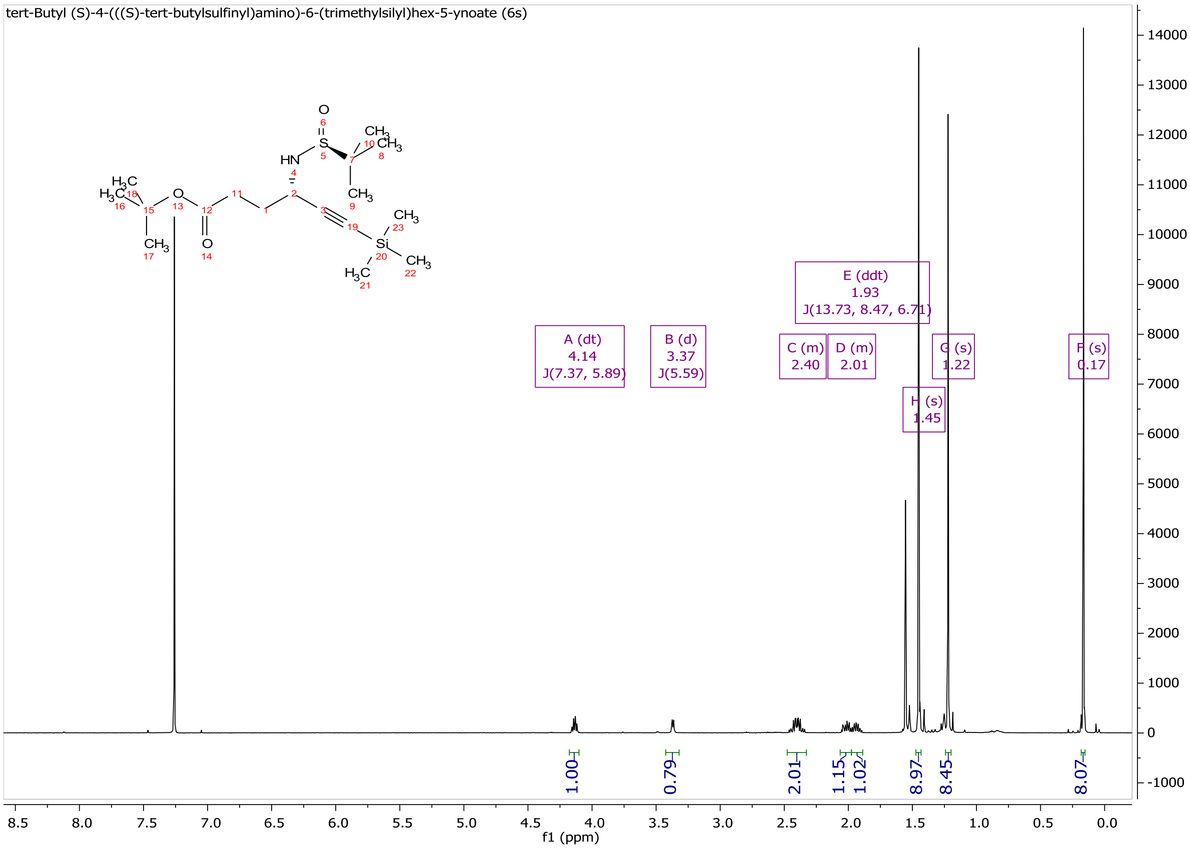
tert-Butyl (S)-4-(((S)-tert-butylsulfinyl)amino)-6-(trimethylsilyl)hex-5-ynoate (6s)
A (dt)
4.14
J(7.37, 5.89)
B (d)
3.37
J(5.59)
C (m)
2.40
D (m)
2.01
E (ddt)
1.93
J(13.73, 8.47, 6.71)
G (s)
1.22
H (s)
1.45
F (s)
0.17
1.00
0.79
2.01
1.15
1.02
8.97
8.45
8.07
f1 (ppm)

tert-Butyl (S)-4-(((S)-tert-butylsulfinyl)amino)-6-(trimethylsilyl)hex-5-ynoate (6s)

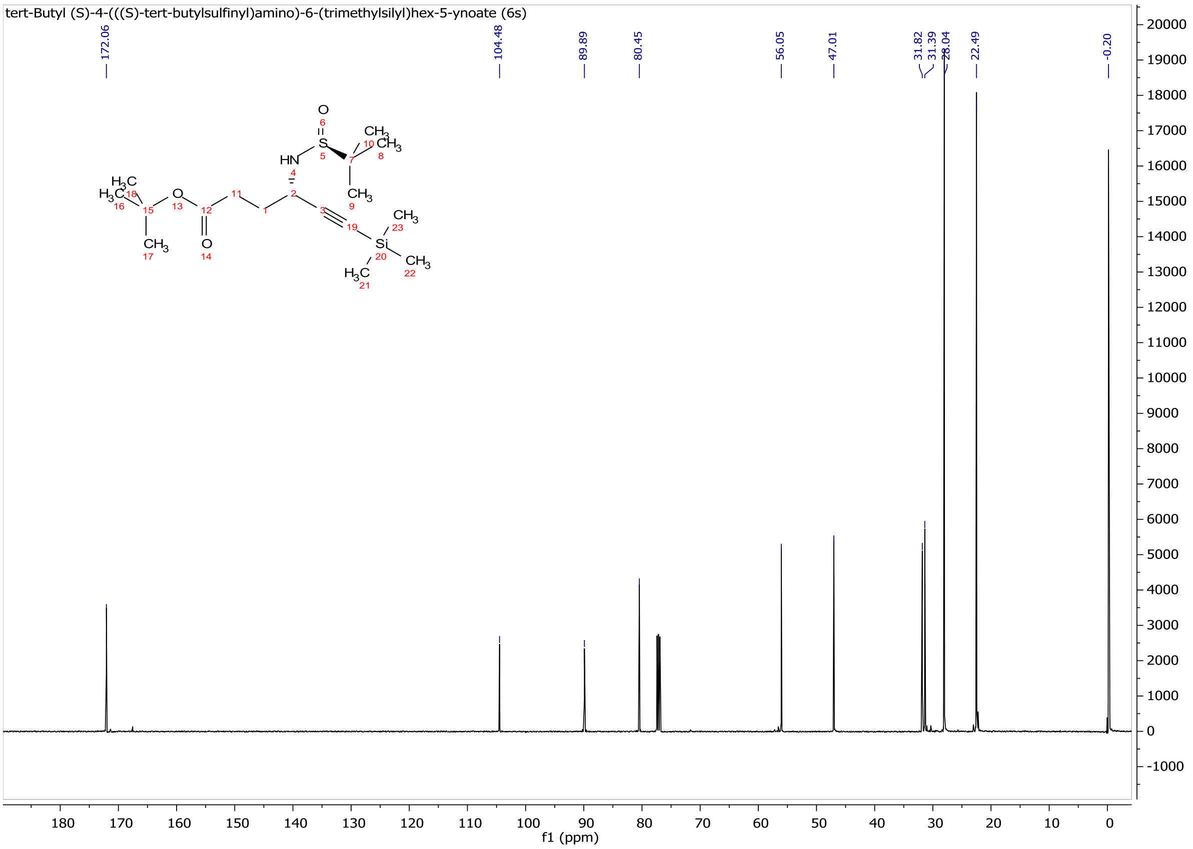

(S)-N-((S)-6-((tert-Butyldimethylsilyl)oxy)-1-(trimethylsilyl)hex-1-yn-3-yl)-tert-butylsulfinamide (6t)

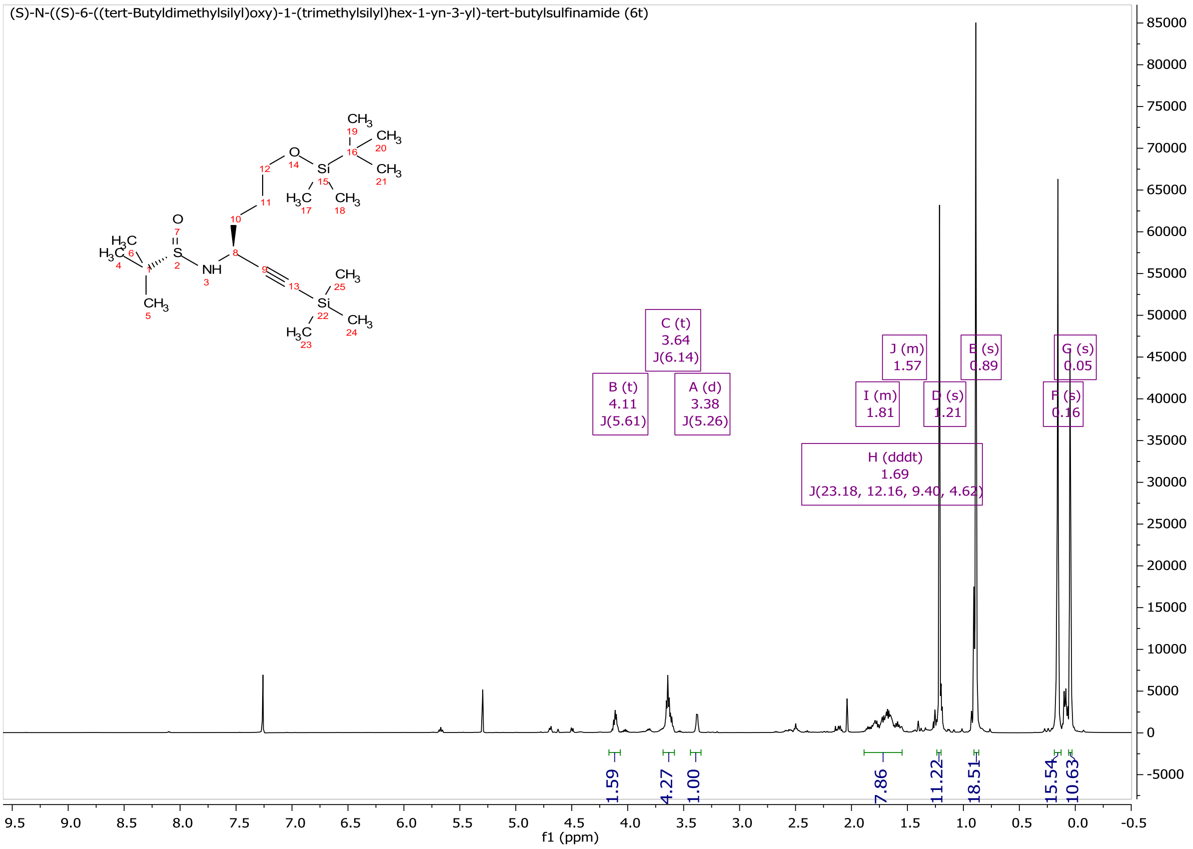

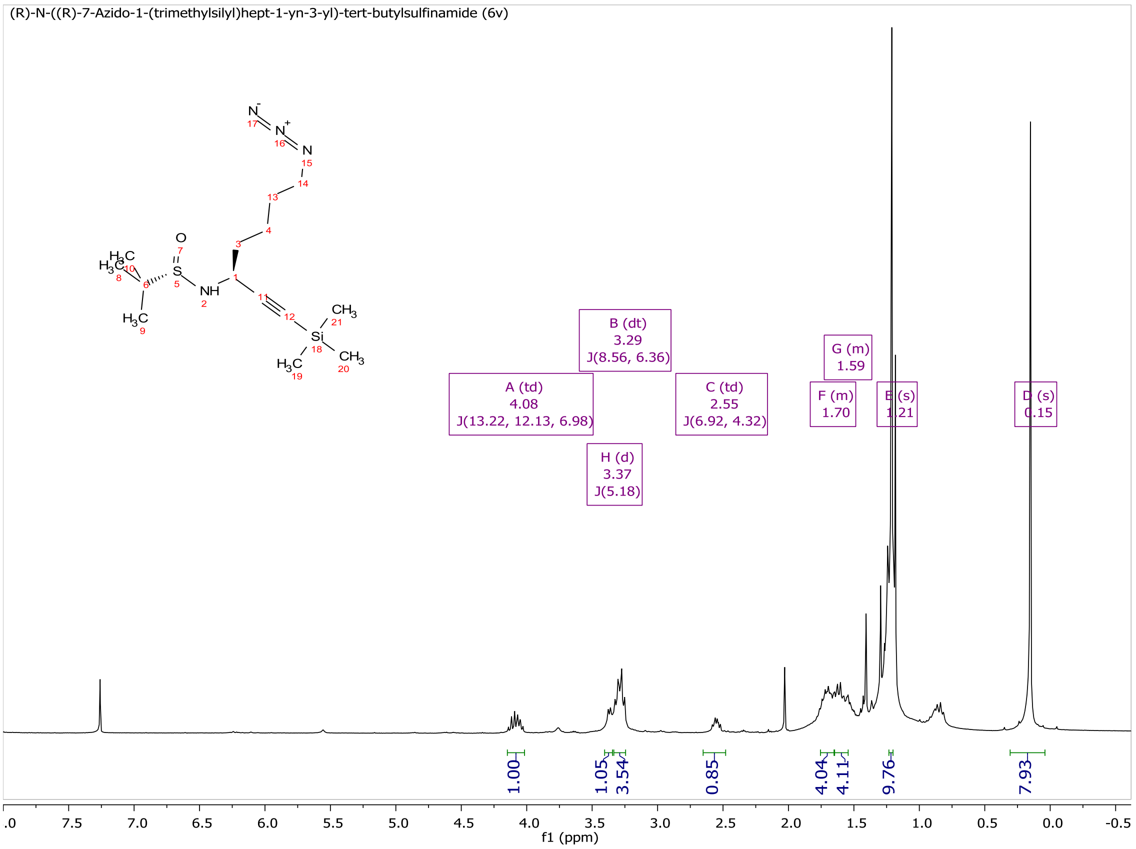

Propargylamines **7**

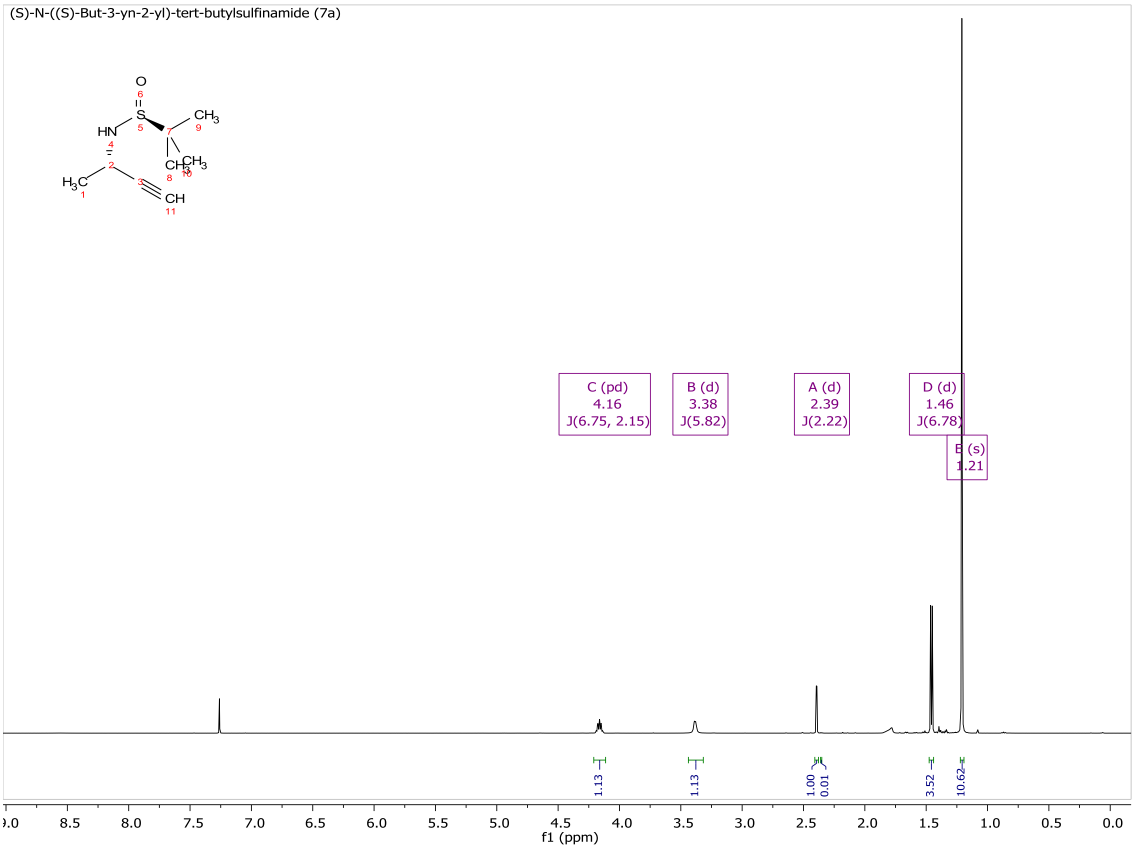

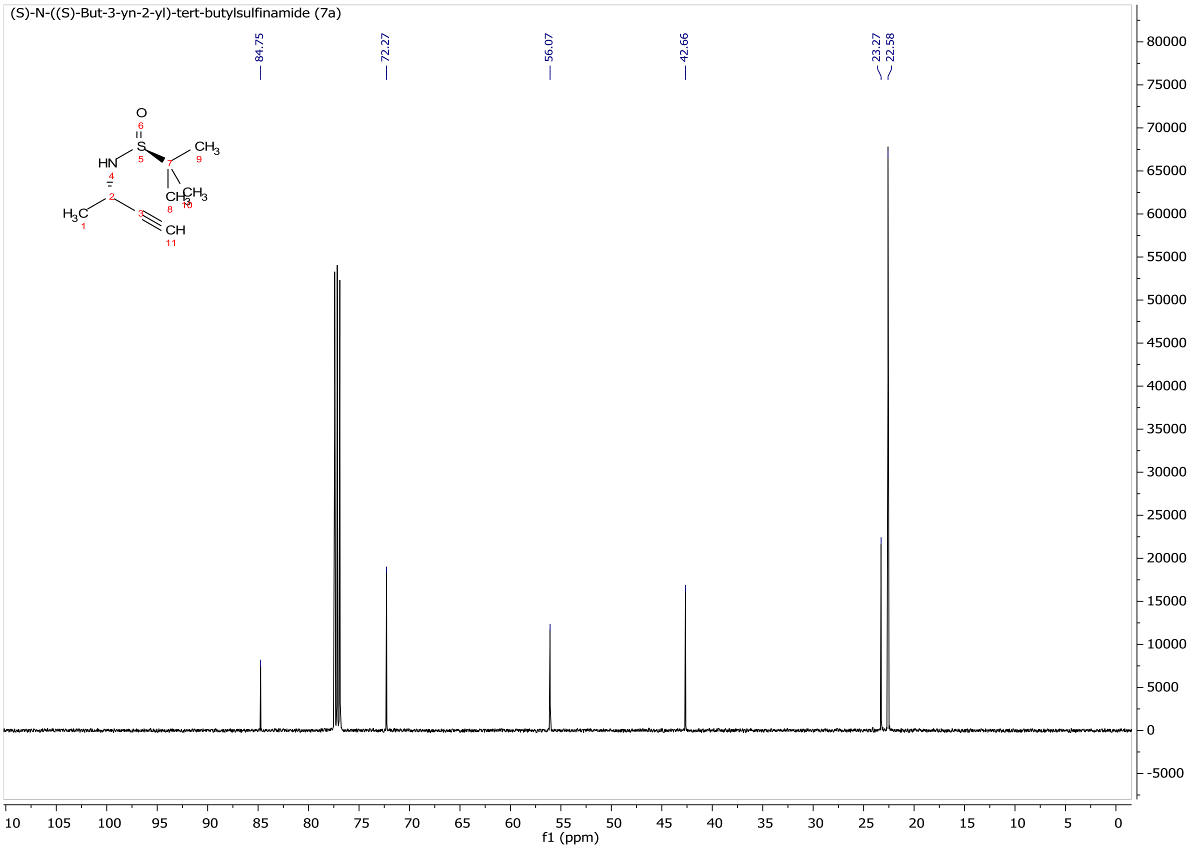
(S)-N-((S)-But-3-yn-2-yl)-tert-butylsulfinamide (7a)
84.75
72.27
56.07
42.66
23.27
22.58
f1 (ppm)

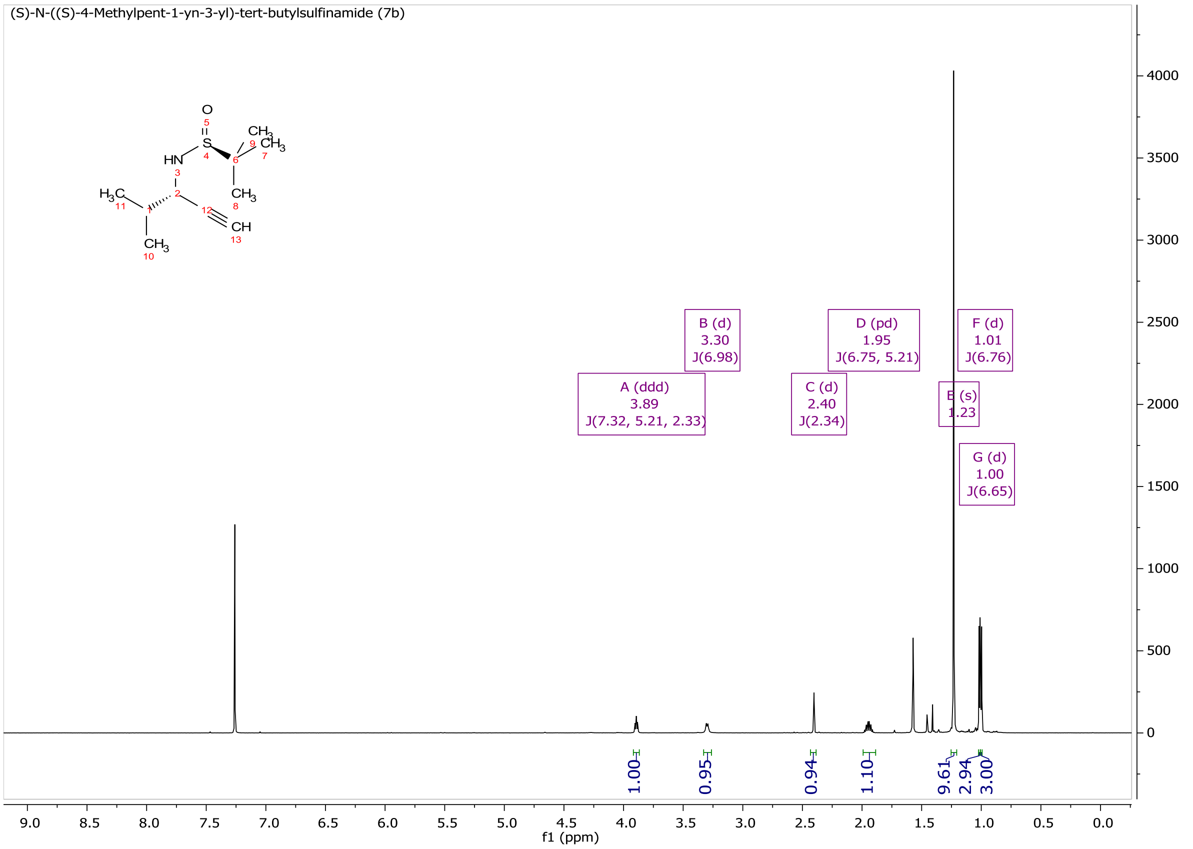
(S)-N-((S)-4-Methylpent-1-yn-3-yl)-tert-butylsulfinamide (7b)
A (ddd) 3.89 J(7.32, 5.21, 2.33)
B (d) 3.30 J(6.98)
C (d) 2.40 J(2.34)
D (pd) 1.95 J(6.75, 5.21)
E (s) 1.23
F (d) 1.01 J(6.76)
G (d) 1.00 J(6.65)
1.00
0.95
0.94
1.10
9.61
2.94
3.00
f1 (ppm)

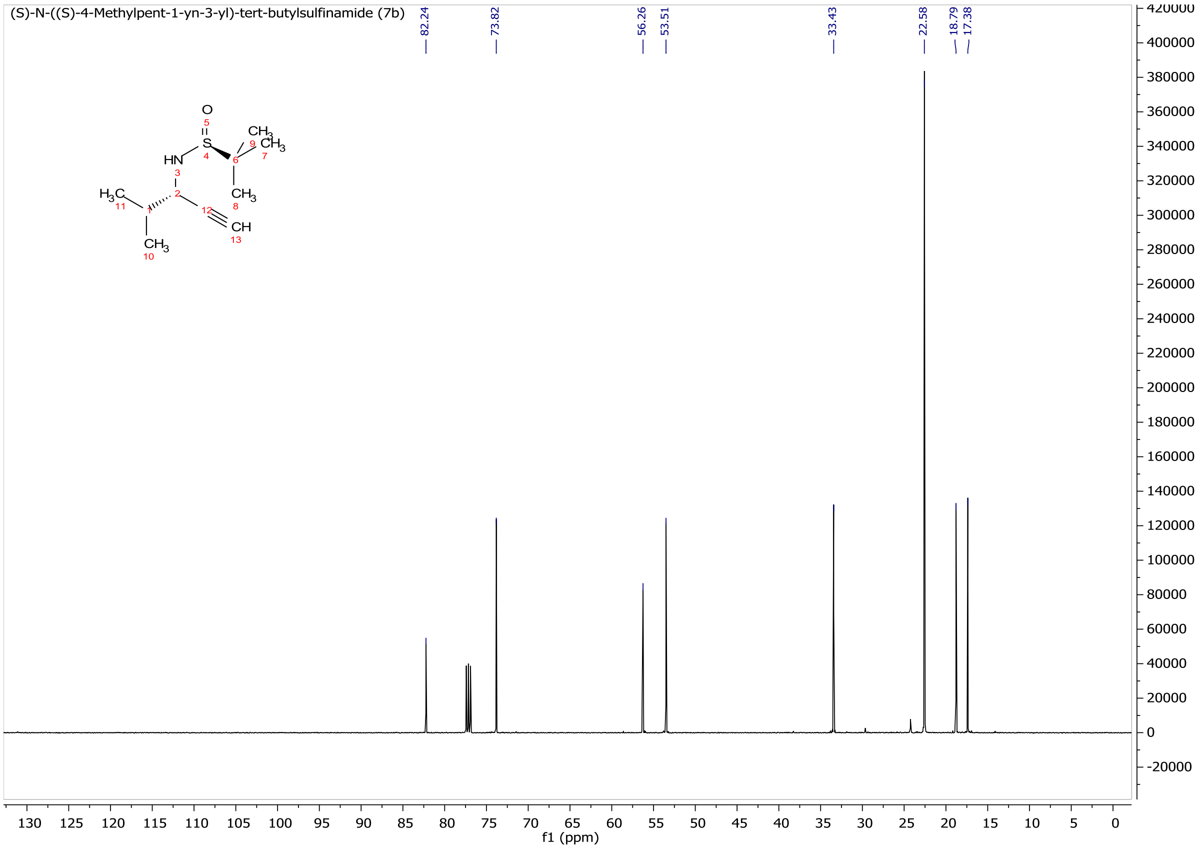

(S)-N-((S)-4-Methylpent-1-yn-3-yl)-tert-butylsulfinamide (7b)
82.24
73.82
56.26
53.51
33.43
22.58
18.79
17.38
f1 (ppm)

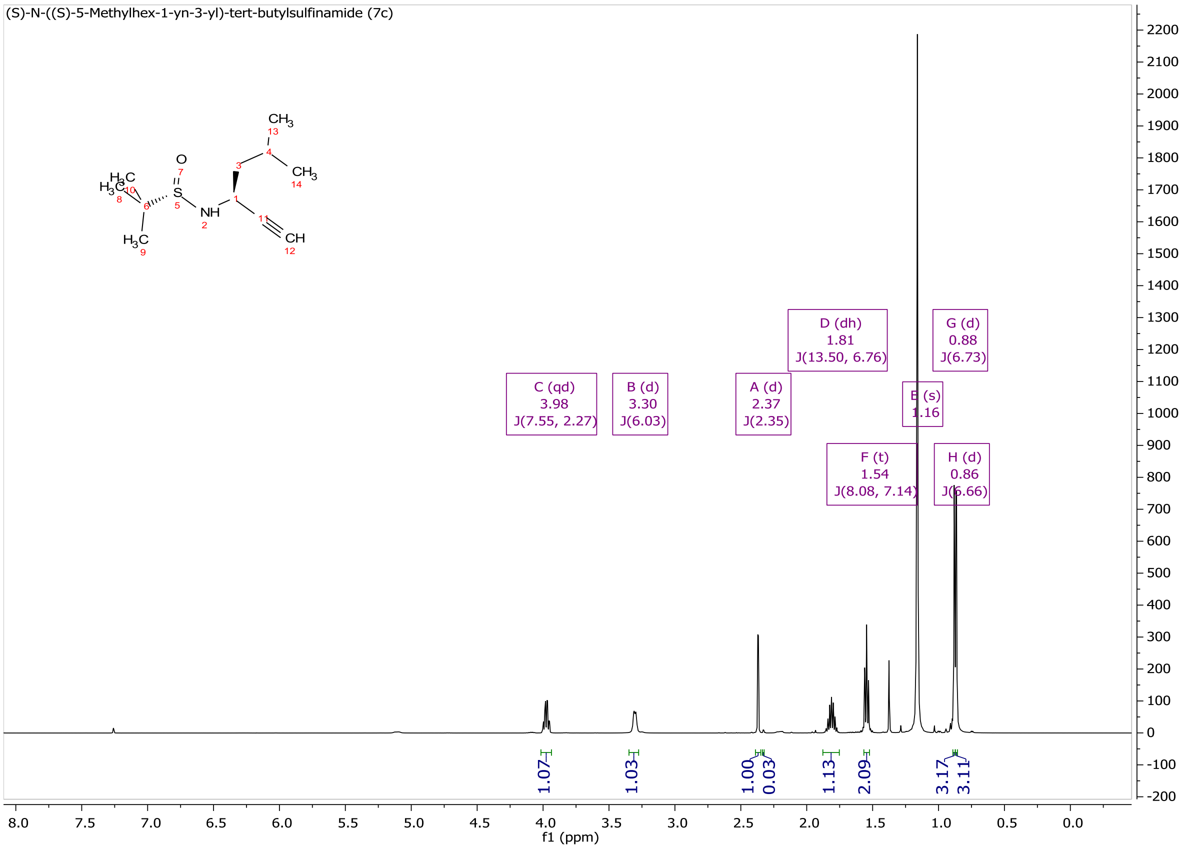

(S)-N-((S)-5-Methylhex-1-yn-3-yl)-tert-butylsulfinamide (7c)
C (qd) 3.98 J(7.55, 2.27)
B (d) 3.30 J(6.03)
A (d) 2.37 J(2.35)
D (dh) 1.81 J(13.50, 6.76)
E (s) 1.16
F (t) 1.54 J(8.08, 7.14)
G (d) 0.88 J(6.73)
H (d) 0.86 J(6.66)
1.07
1.03
1.00
0.03
1.13
2.09
3.17
3.11
f1 (ppm)

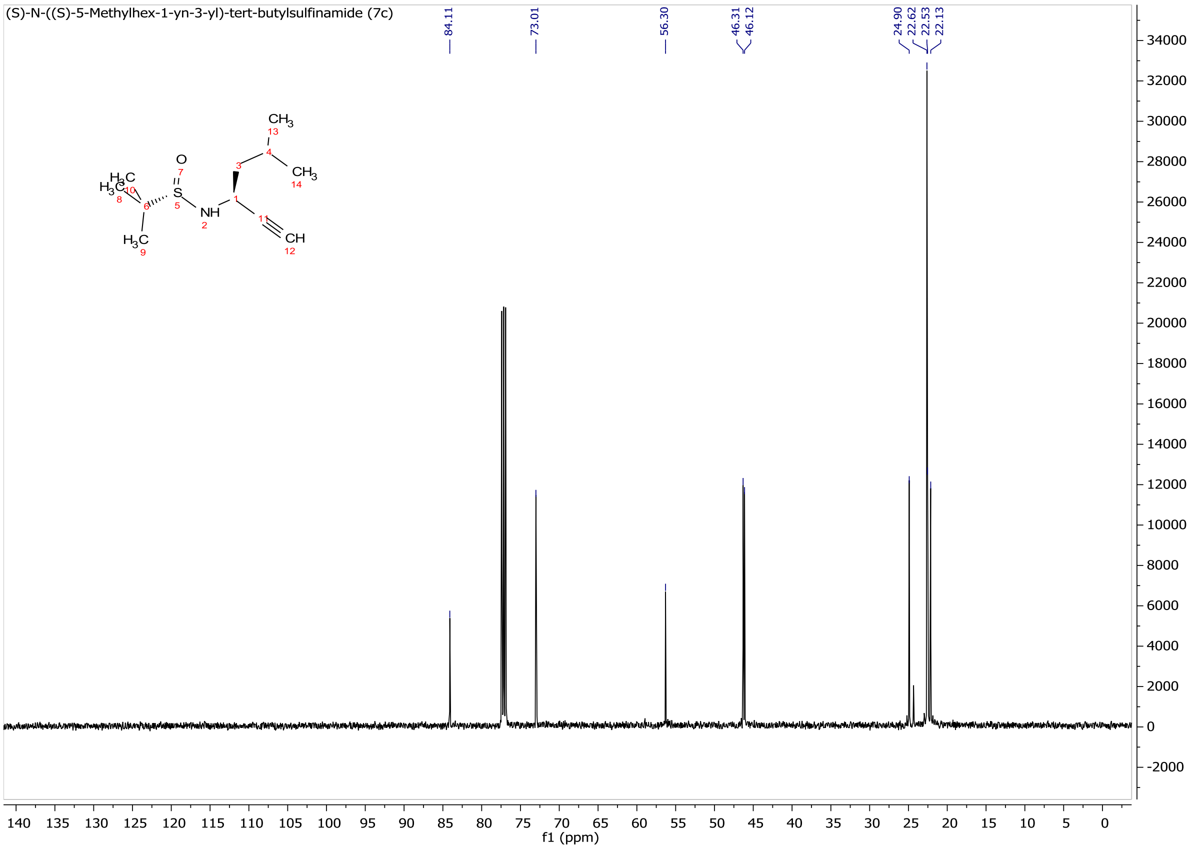
(S)-N-((S)-5-Methylhex-1-yn-3-yl)-tert-butylsulfinamide (7c)
84.11
73.01
56.30
46.31
46.12
24.90
22.62
22.53
22.13
f1 (ppm)

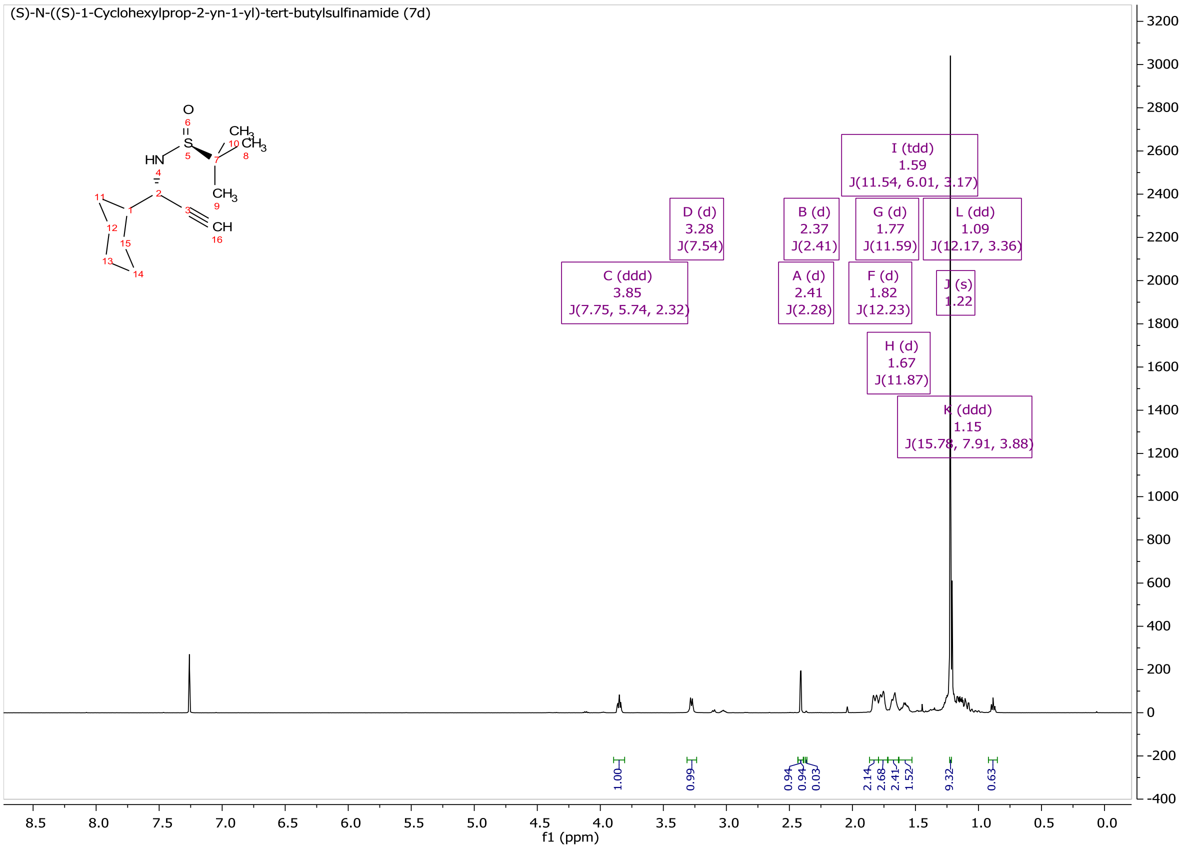
(S)-N-((S)-1-Cyclohexylprop-2-yn-1-yl)-tert-butylsulfinamide (7d)
I (tdd) 1.59 J(11.54, 6.01, 3.17)
D (d) 3.28 J(7.54)
B (d) 2.37 J(2.41)
G (d) 1.77 J(11.59)
L (dd) 1.09 J(12.17, 3.36)
C (ddd) 3.85 J(7.75, 5.74, 2.32)
A (d) 2.41 J(2.28)
F (d) 1.82 J(12.23)
J (s) 1.22
H (d) 1.67 J(11.87)
K (ddd) 1.15 J(15.78, 7.91, 3.88)
f1 (ppm)

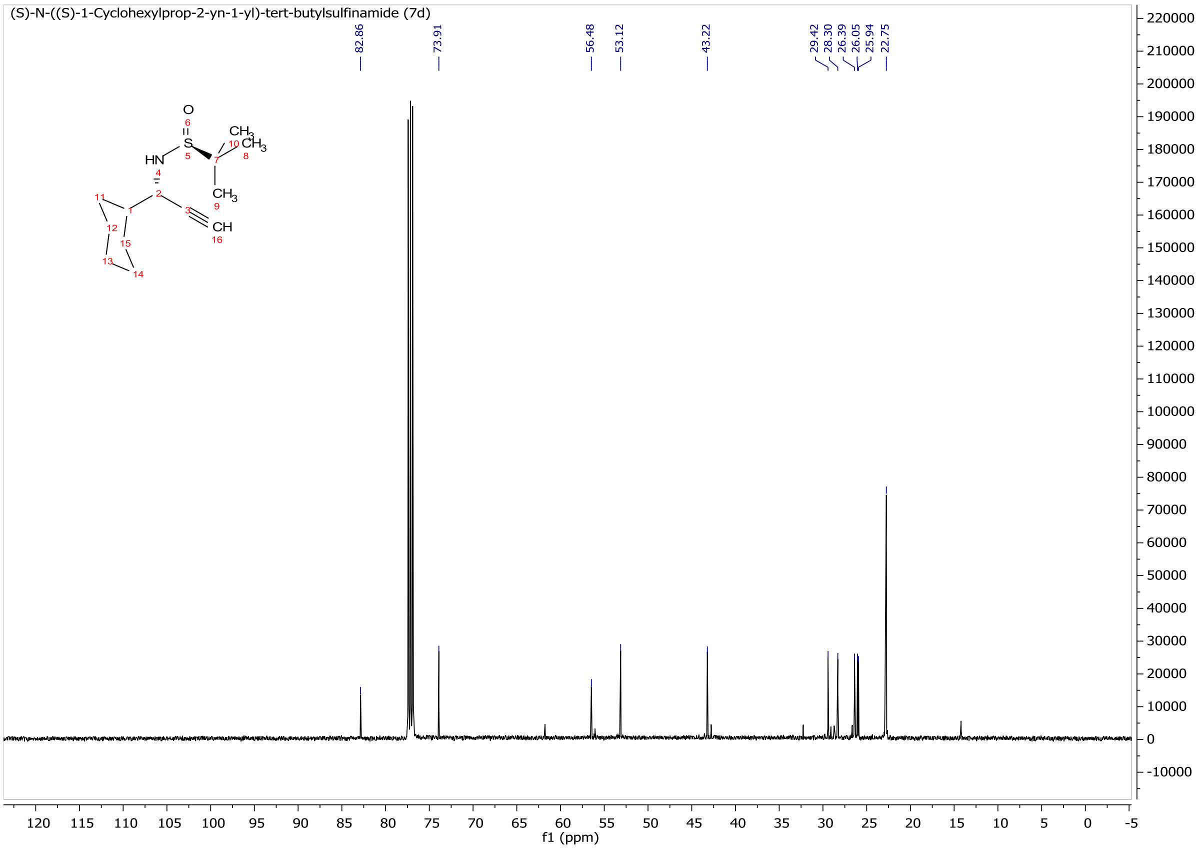
(S)-N-((S)-1-Cyclohexylprop-2-yn-1-yl)-tert-butylsulfinamide (7d)
82.86
73.91
56.48
53.12
43.22
29.42
28.30
26.39
26.05
25.94
22.75
f1 (ppm)

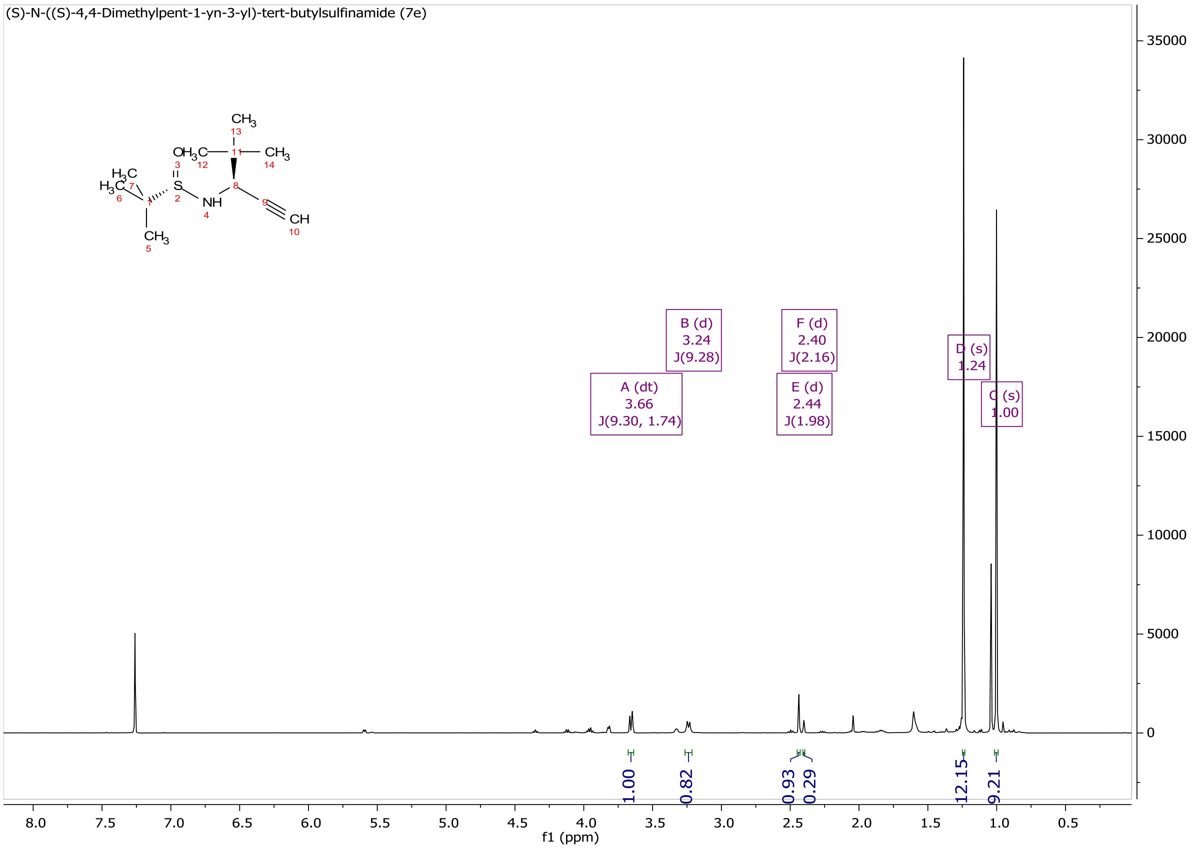
(S)-N-((S)-4,4-Dimethylpent-1-yn-3-yl)-tert-butylsulfinamide (7e)
A (dt)
3.66
J(9.30, 1.74)
B (d)
3.24
J(9.28)
E (d)
2.44
J(1.98)
F (d)
2.40
J(2.16)
D (s)
1.24
C (s)
1.00
1.00
0.82
0.93
0.29
12.15
9.21
f1 (ppm)

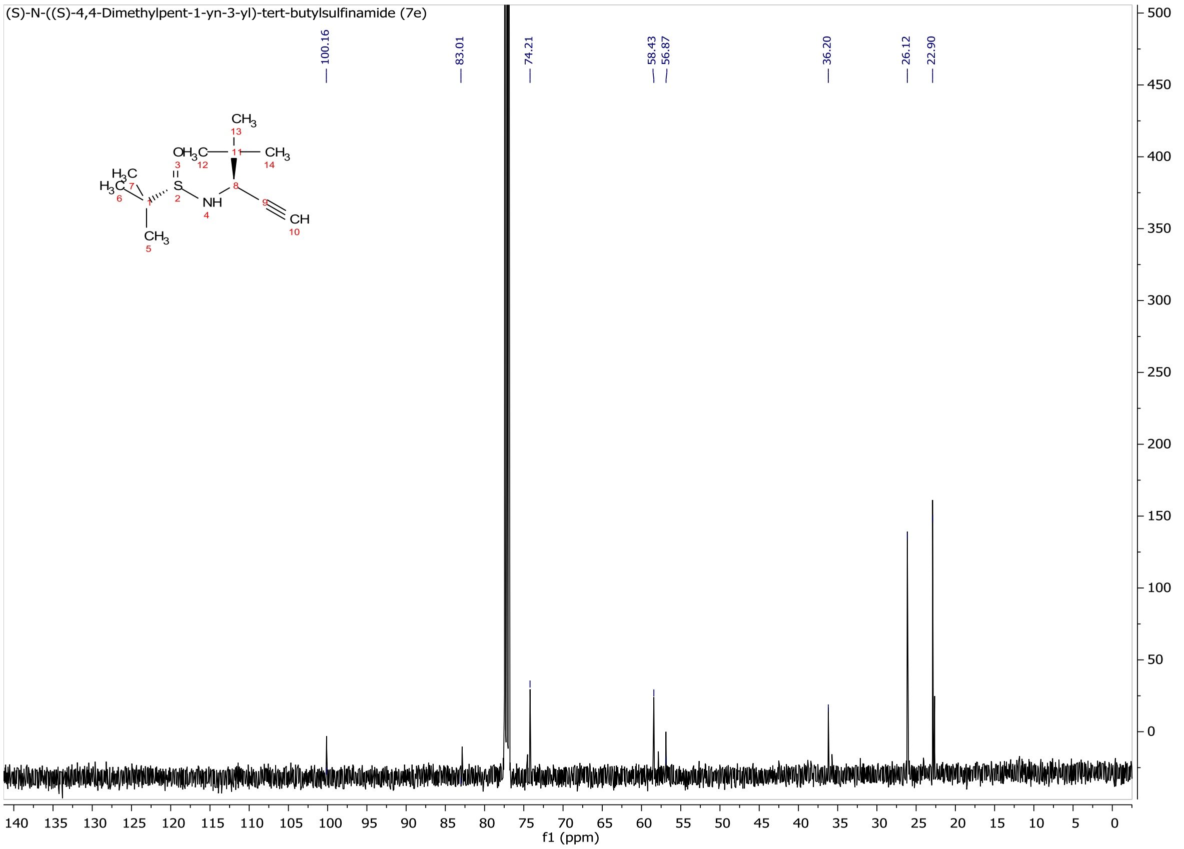
(S)-N-((S)-4,4-Dimethylpent-1-yn-3-yl)-tert-butylsulfinamide (7e)
100.16
83.01
74.21
58.43
56.87
36.20
26.12
22.90
f1 (ppm)

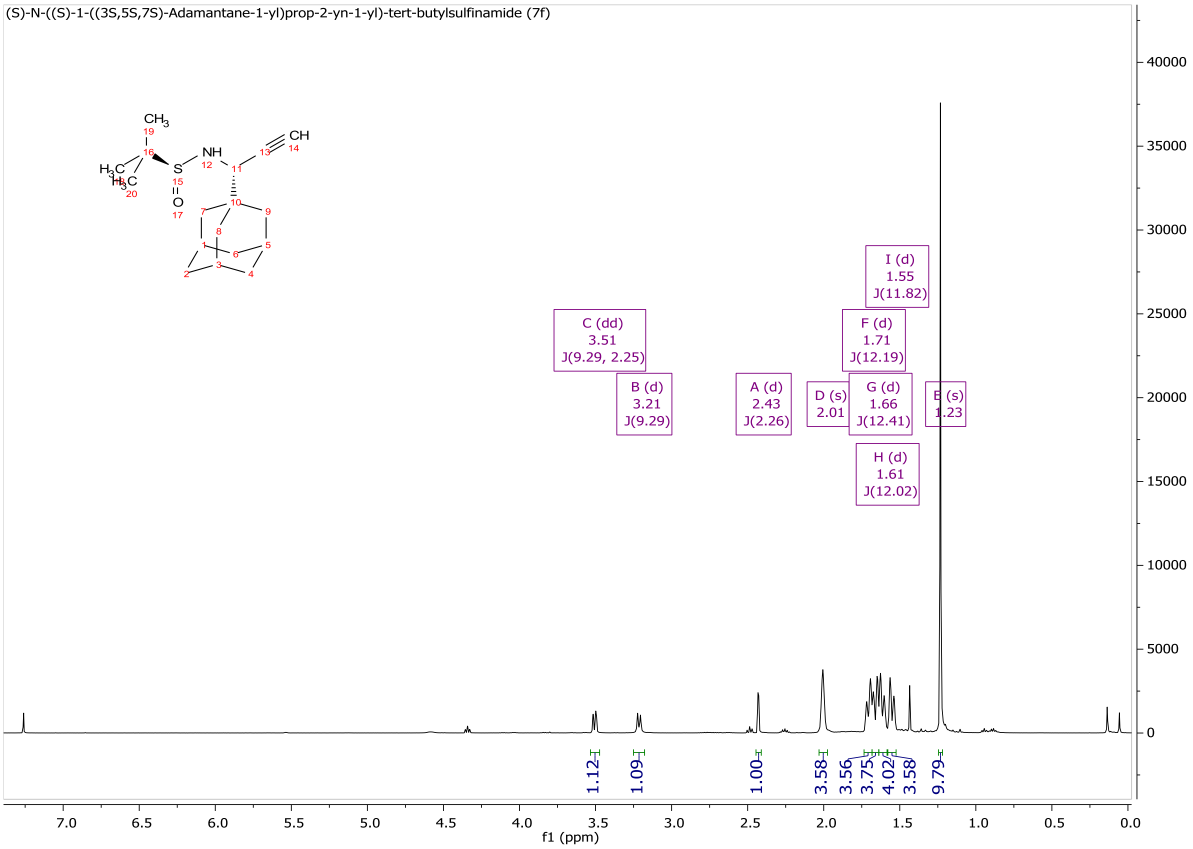
(S)-N-((S)-1-((3S,5S,7S)-Adamantane-1-yl)prop-2-yn-1-yl)-tert-butylsulfinamide (7f)
C (dd) 3.51 J(9.29, 2.25)
B (d) 3.21 J(9.29)
A (d) 2.43 J(2.26)
D (s) 2.01
F (d) 1.71 J(12.19)
G (d) 1.66 J(12.41)
H (d) 1.61 J(12.02)
I (d) 1.55 J(11.82)
E (s) 1.23
1.12
1.09
1.00
3.58
3.56
3.75
4.02
3.58
9.79
f1 (ppm)

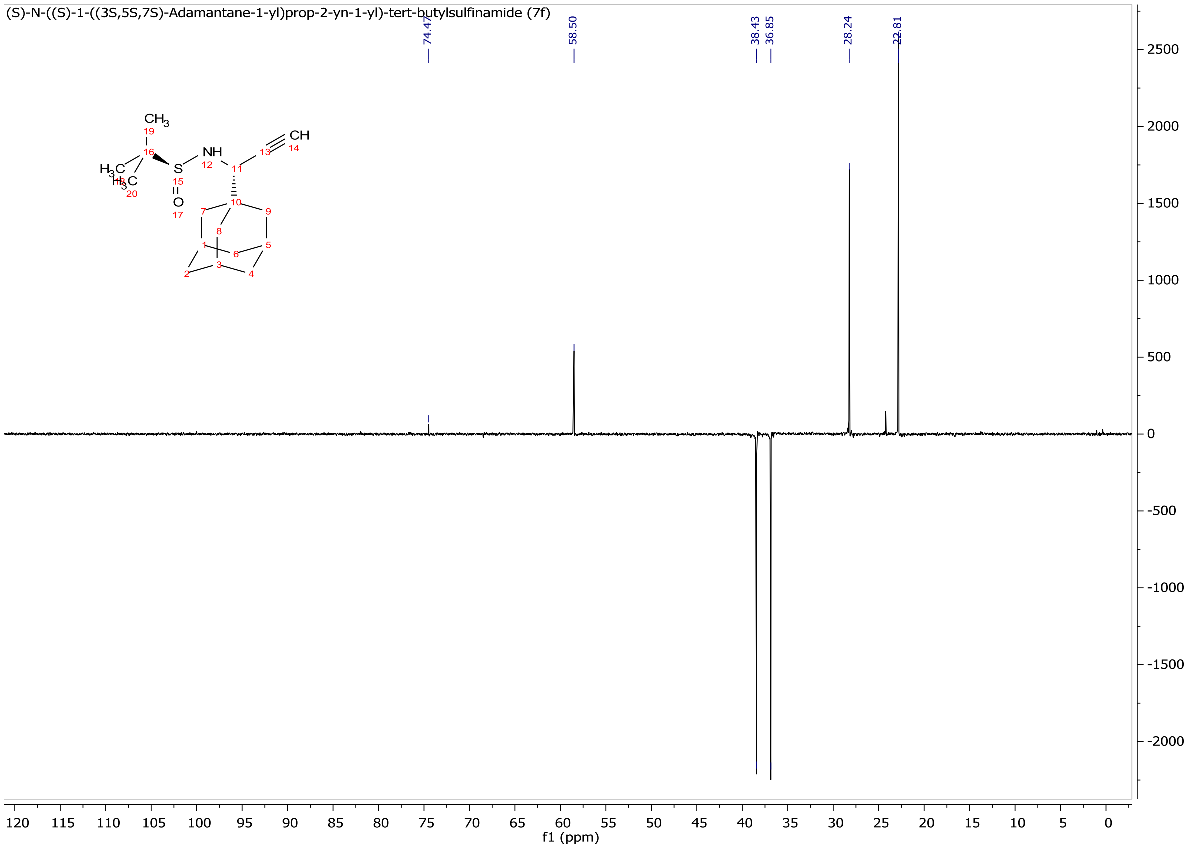
(S)-N-((S)-1-((3S,5S,7S)-Adamantane-1-yl)prop-2-yn-1-yl)-tert-butylsulfinamide (7f)
74.47
58.50
38.43
36.85
28.24
22.81
f1 (ppm)

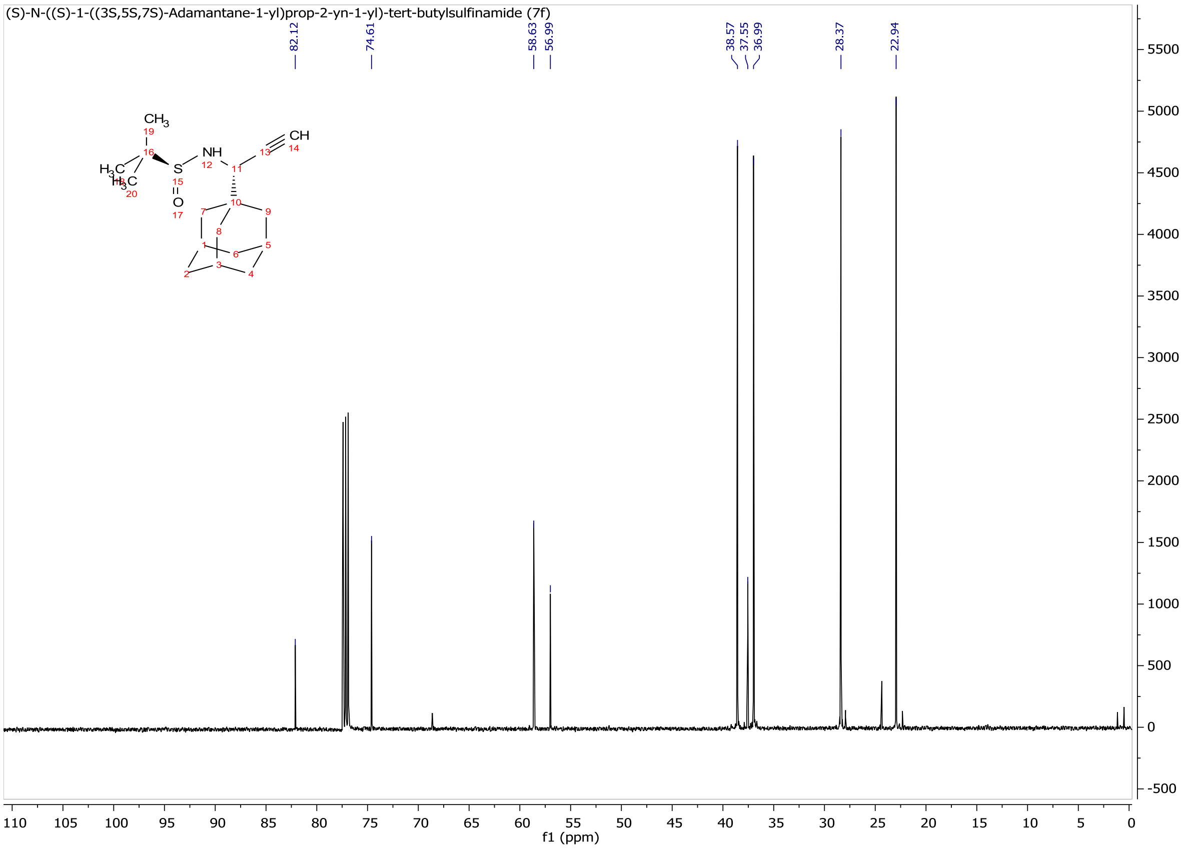
(S)-N-((S)-1-((3S,5S,7S)-Adamantane-1-yl)prop-2-yn-1-yl)-tert-butylsulfinamide (7f)
82.12
74.61
58.63
56.99
38.57
37.55
36.99
28.37
22.94
f1 (ppm)

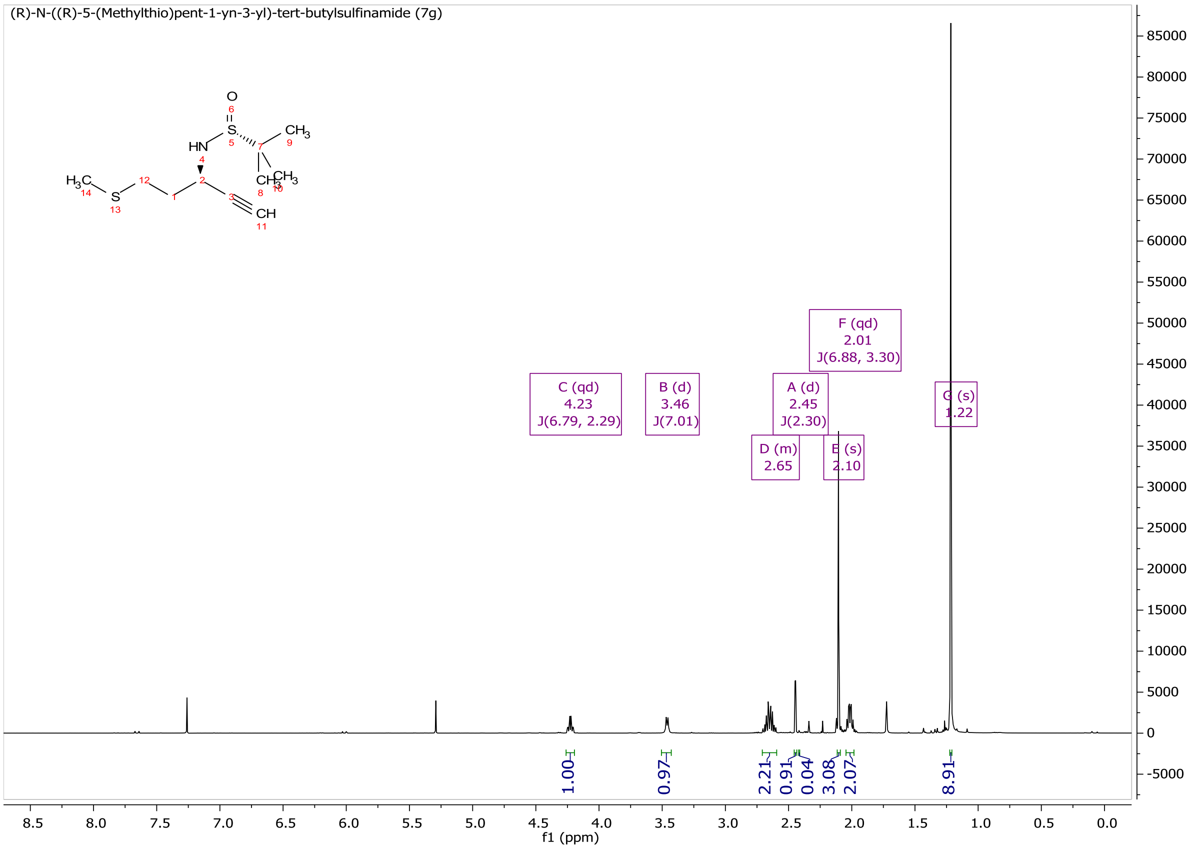
(R)-N-((R)-5-(Methylthio)pent-1-yn-3-yl)-tert-butylsulfinamide (7g)
C (qd)
4.23
J(6.79, 2.29)
B (d)
3.46
J(7.01)
A (d)
2.45
J(2.30)
D (m)
2.65
F (qd)
2.01
J(6.88, 3.30)
E (s)
2.10
G (s)
1.22
1.00
0.97
2.21
0.91
0.04
3.08
2.07
8.91
f1 (ppm)

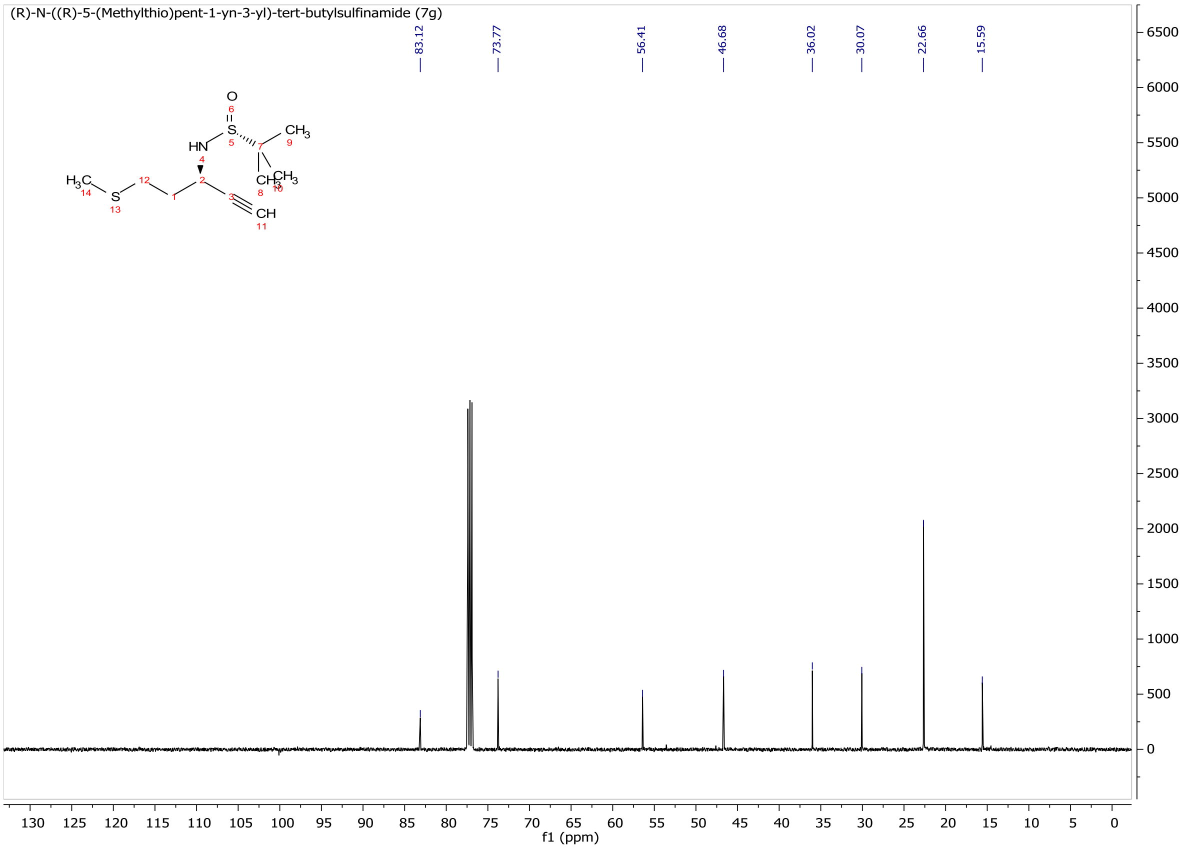
(R)-N-((R)-5-(Methylthio)pent-1-yn-3-yl)-tert-butylsulfinamide (7g)
83.12
73.77
56.41
46.68
36.02
30.07
22.66
15.59
f1 (ppm)

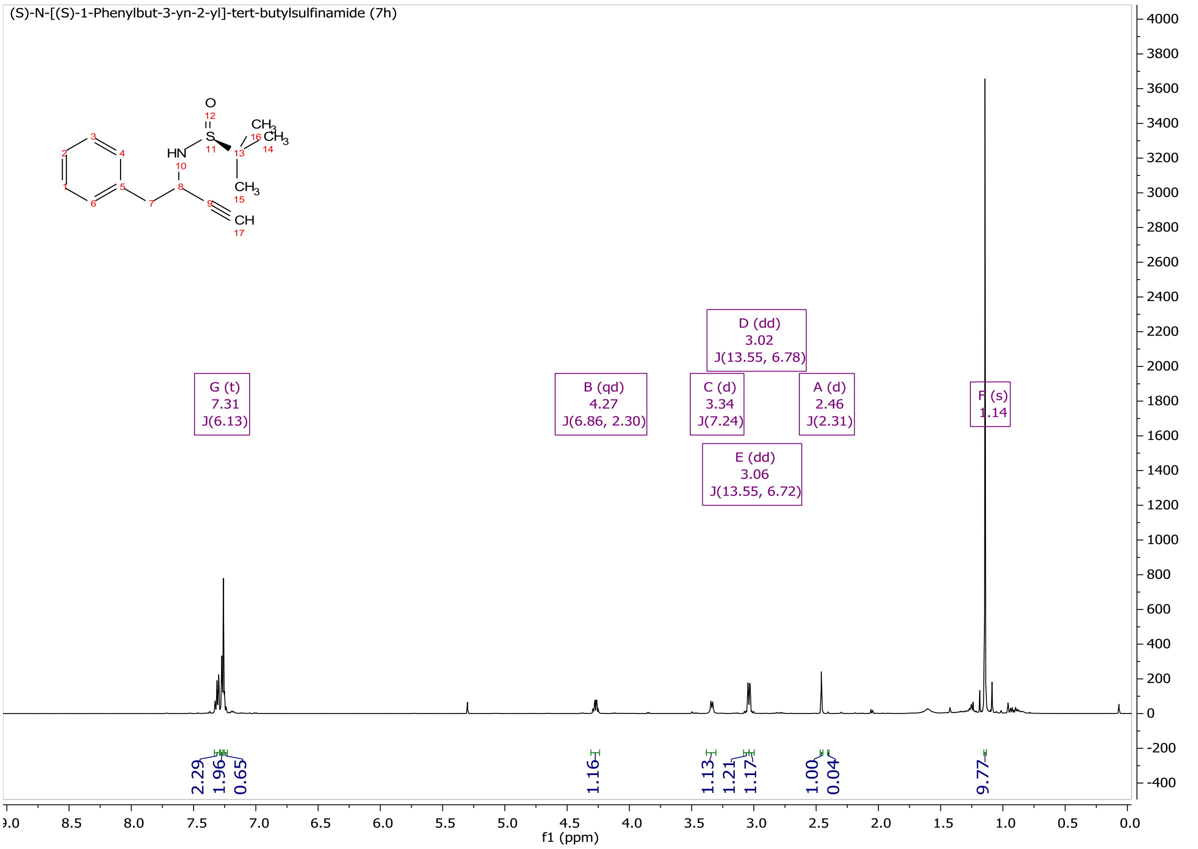
(S)-N-[(S)-1-Phenylbut-3-yn-2-yl]-tert-butylsulfinamide (7h)
G (t) 7.31 J(6.13)
B (qd) 4.27 J(6.86, 2.30)
C (d) 3.34 J(7.24)
D (dd) 3.02 J(13.55, 6.78)
E (dd) 3.06 J(13.55, 6.72)
A (d) 2.46 J(2.31)
F (s) 1.14
2.29 1.96 0.65 1.16 1.13 1.21 1.17 1.00 0.04 9.77
f1 (ppm)

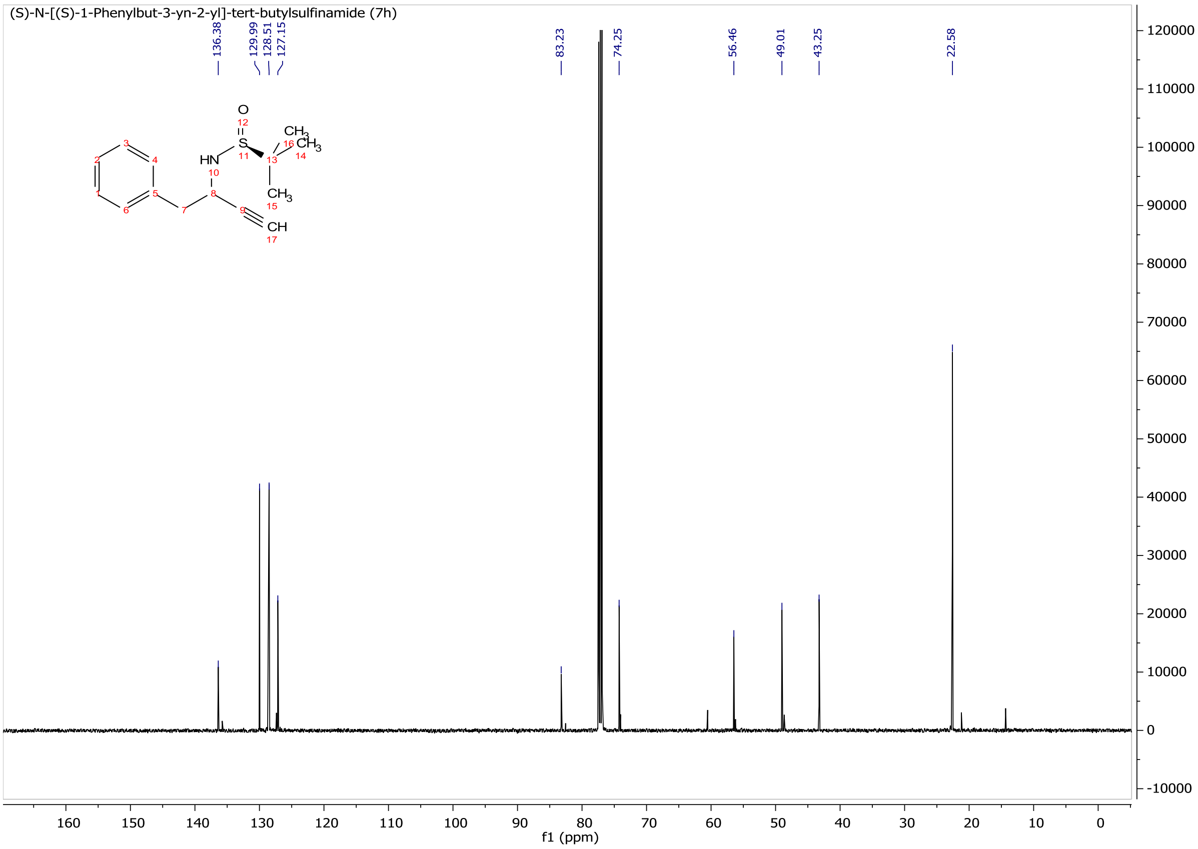
(S)-N-[(S)-1-Phenylbut-3-yn-2-yl]-tert-butylsulfinamide (7h)
136.38 129.99 128.51 127.15 83.23 74.25 56.46 49.01 43.25 22.58
f1 (ppm)

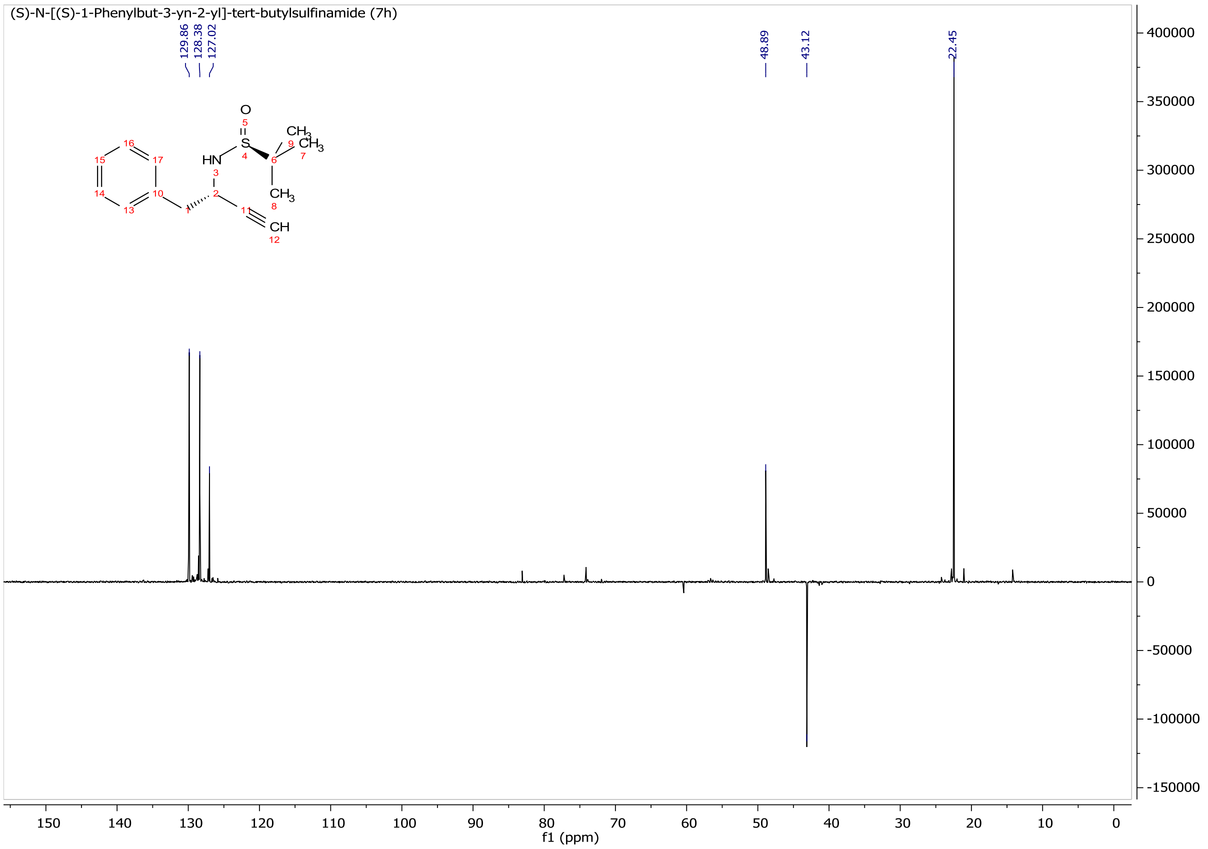
(S)-N-[(S)-1-Phenylbut-3-yn-2-yl]-tert-butylsulfinamide (7h)
129.86
128.38
127.02
48.89
43.12
22.45
f1 (ppm)

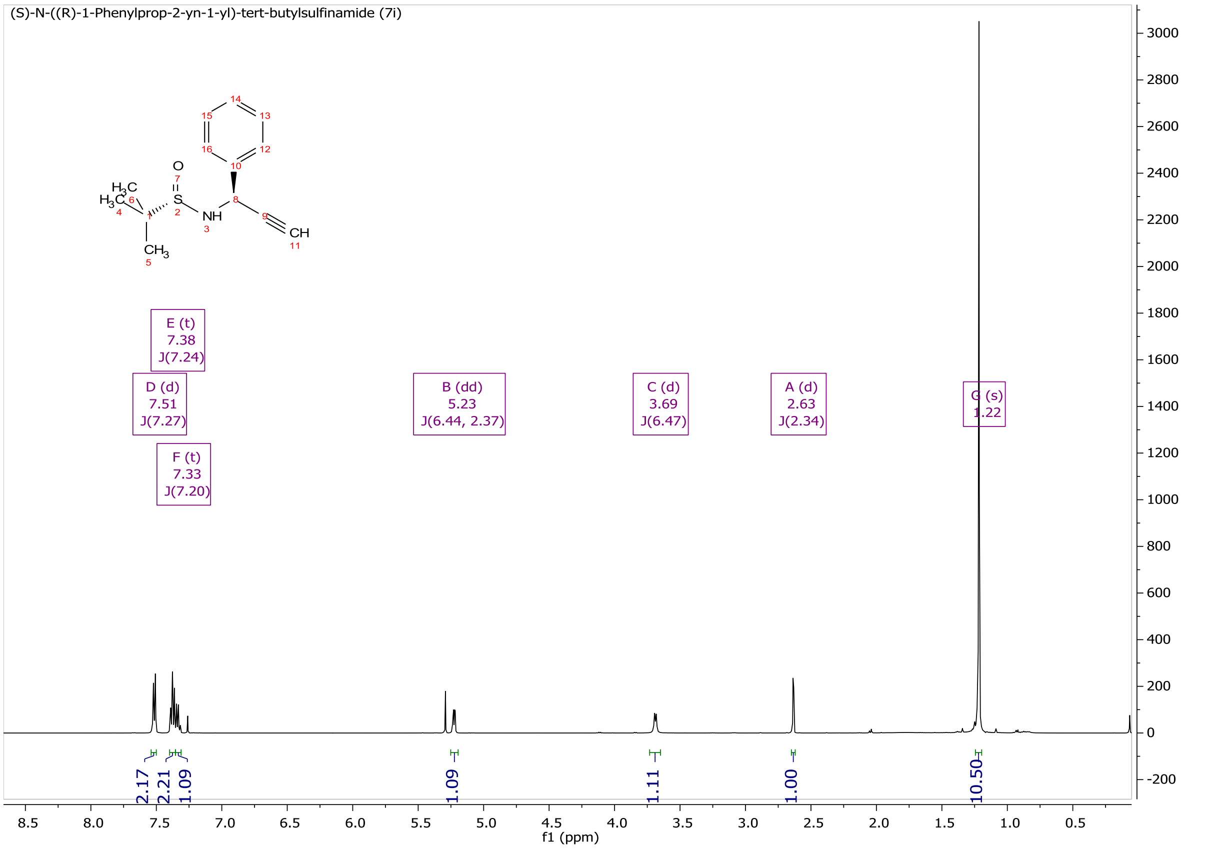
(S)-N-((R)-1-Phenylprop-2-yn-1-yl)-tert-butylsulfinamide (7i)
E (t)
7.38
J(7.24)
D (d)
7.51
J(7.27)
F (t)
7.33
J(7.20)
B (dd)
5.23
J(6.44, 2.37)
C (d)
3.69
J(6.47)
A (d)
2.63
J(2.34)
G (s)
1.22
2.17
2.21
1.09
1.09
1.11
1.00
10.50
f1 (ppm)

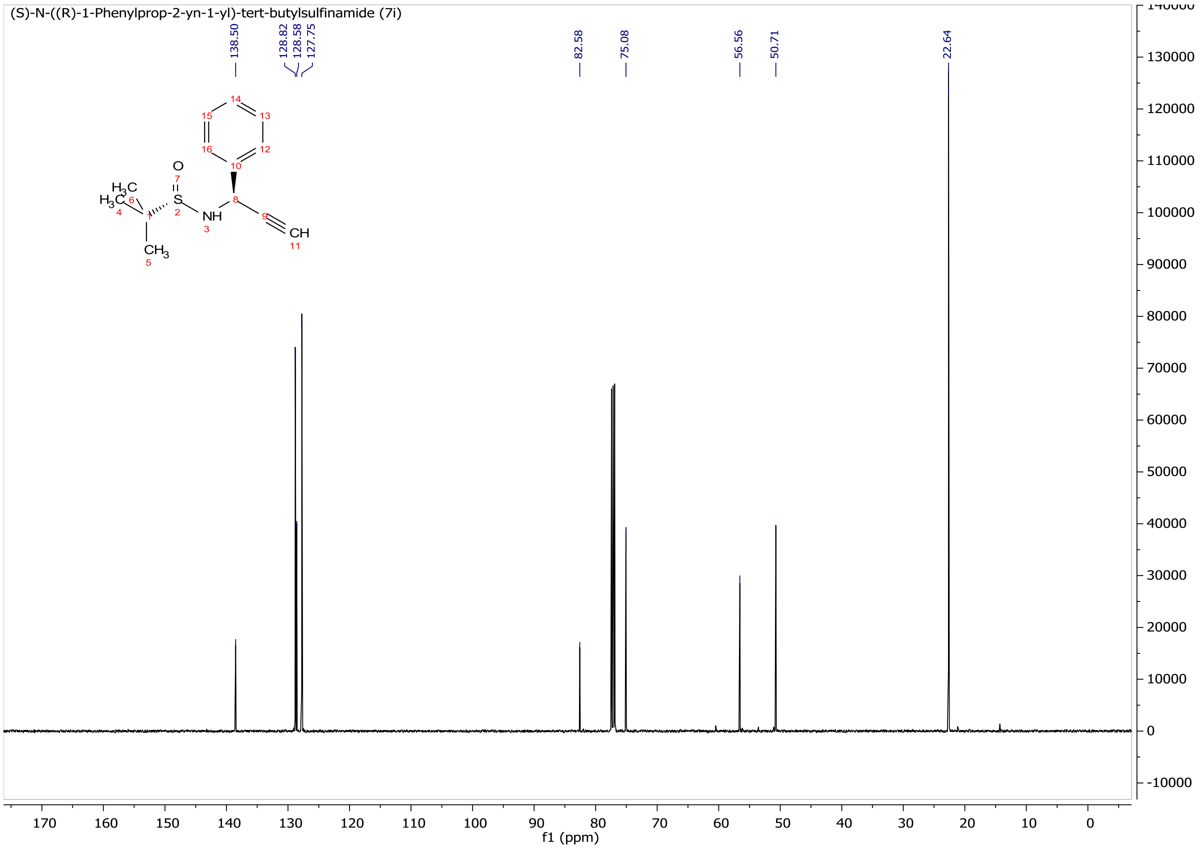

(S)-N-((R)-1-Phenylprop-2-yn-1-yl)-tert-butylsulfinamide (7i)
138.50
128.82
128.58
127.75
82.58
75.08
56.56
50.71
22.64
f1 (ppm)

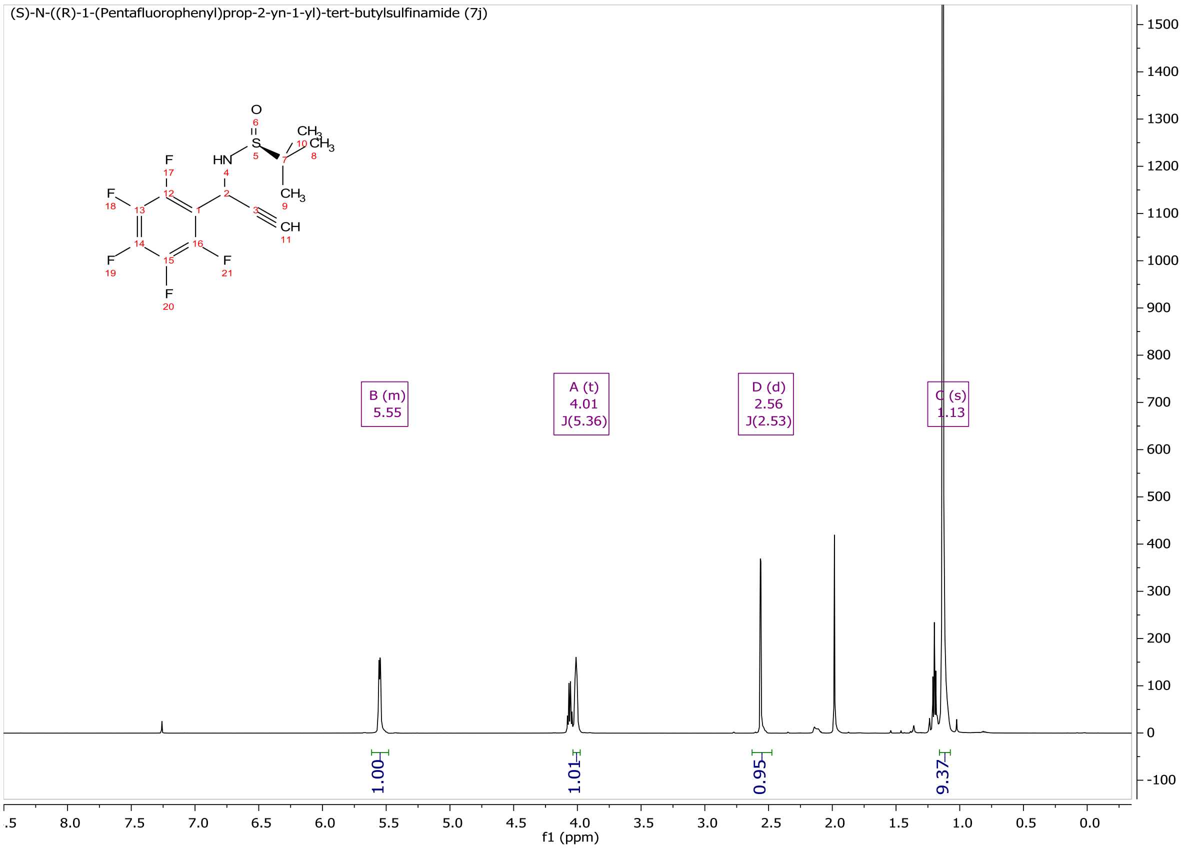

(S)-N-((R)-1-(Pentafluorophenyl)prop-2-yn-1-yl)-tert-butylsulfinamide (7j)
B (m)
5.55
A (t)
4.01
J(5.36)
D (d)
2.56
J(2.53)
C (s)
1.13
1.00
1.01
0.95
9.37
f1 (ppm)

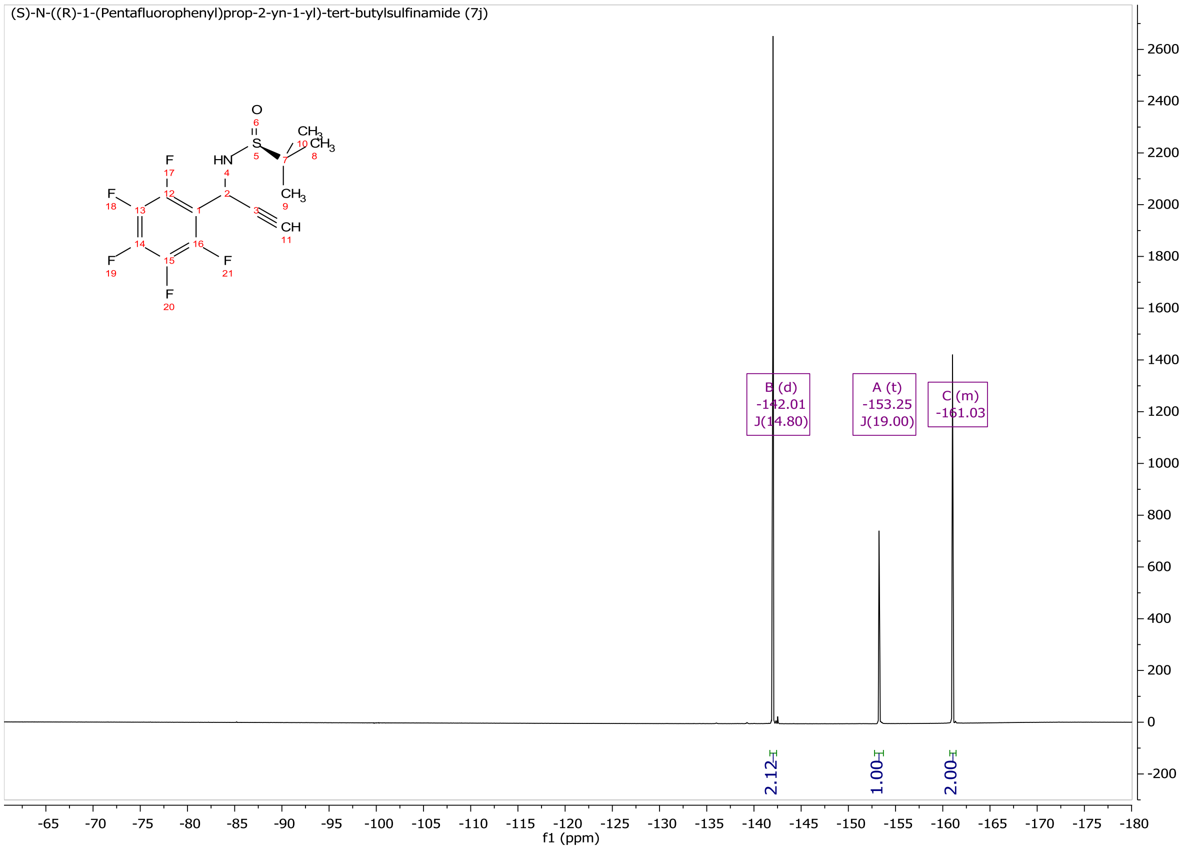
(S)-N-((R)-1-(Pentafluorophenyl)prop-2-yn-1-yl)-tert-butylsulfinamide (7j)
B (d)
-142.01
J(14.80)
A (t)
-153.25
J(19.00)
C (m)
-161.03
2.12
1.00
2.00
f1 (ppm)

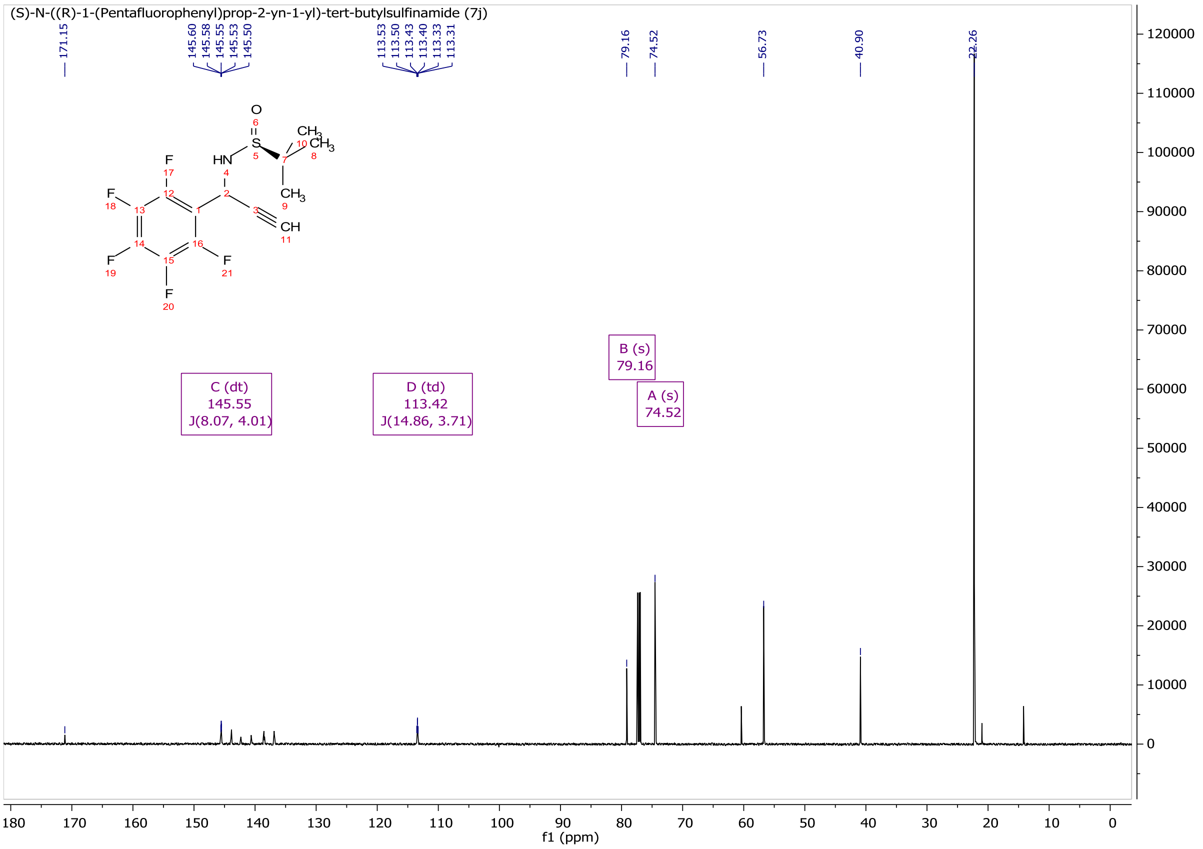
(S)-N-((R)-1-(Pentafluorophenyl)prop-2-yn-1-yl)-tert-butylsulfinamide (7j)
171.15
145.60
145.58
145.55
145.53
145.50
113.53
113.50
113.43
113.40
113.33
113.31
79.16
74.52
56.73
40.90
22.26
C (dt)
145.55
J(8.07, 4.01)
D (td)
113.42
J(14.86, 3.71)
B (s)
79.16
A (s)
74.52
f1 (ppm)

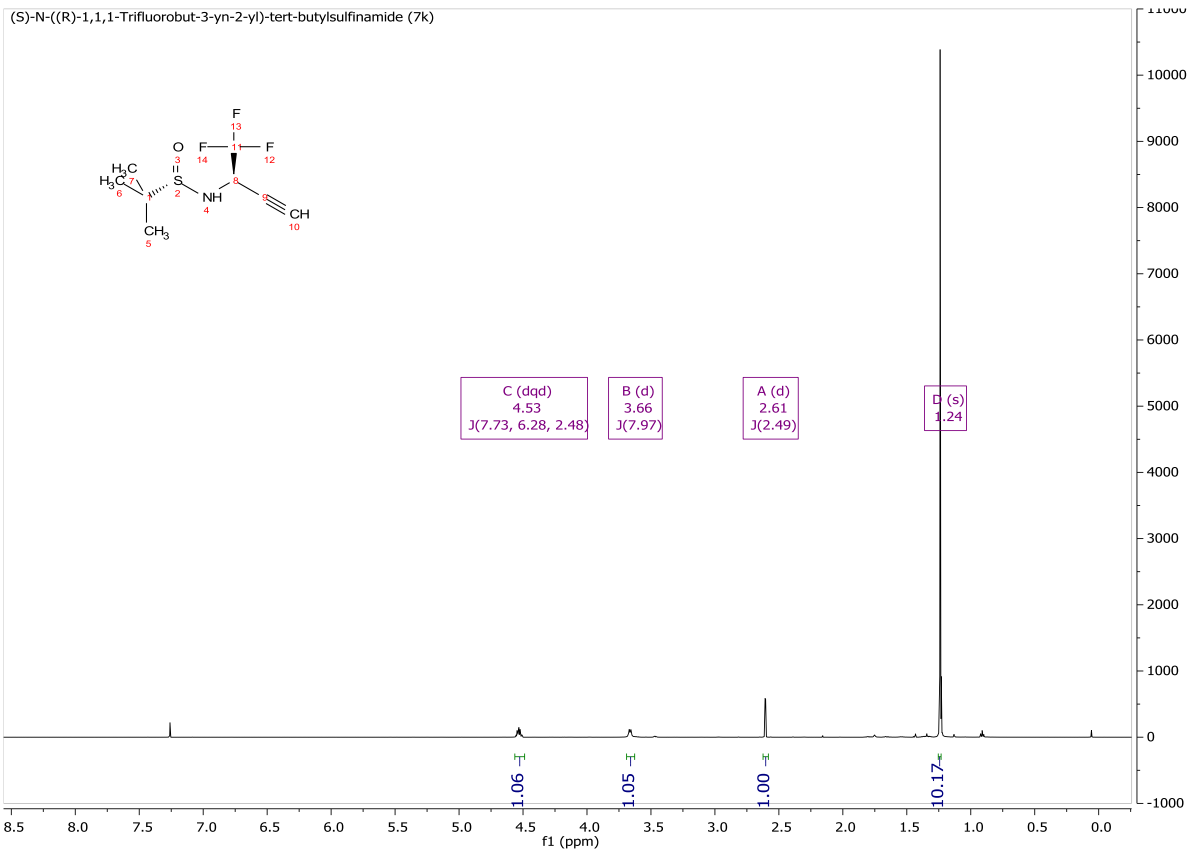
(S)-N-((R)-1,1,1-Trifluorobut-3-yn-2-yl)-tert-butylsulfinamide (7k)
C (dqd)
4.53
J(7.73, 6.28, 2.48)
B (d)
3.66
J(7.97)
A (d)
2.61
J(2.49)
D (s)
1.24
1.06
1.05
1.00
10.17
f1 (ppm)

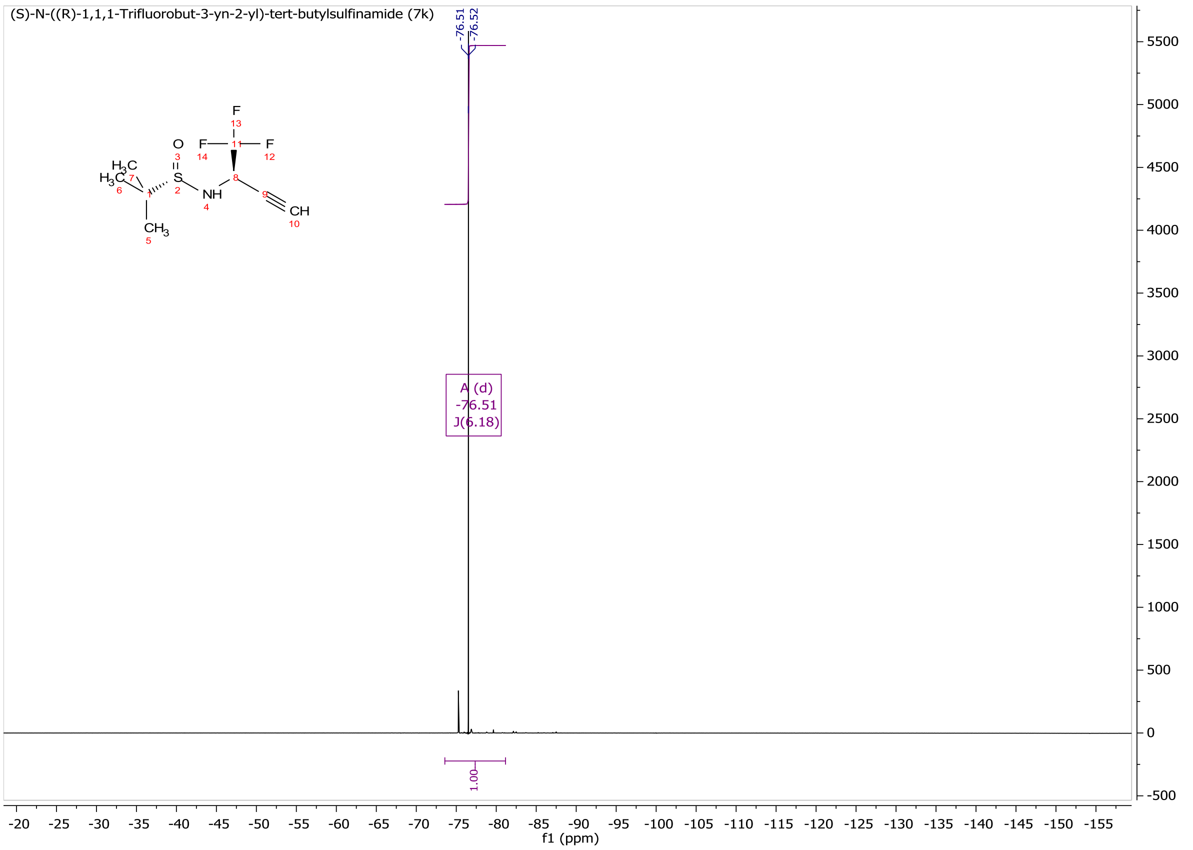
(S)-N-((R)-1,1,1-Trifluorobut-3-yn-2-yl)-tert-butylsulfinamide (7k)
-76.51
-76.52
A (d)
-76.51
J(6.18)
1.00
f1 (ppm)

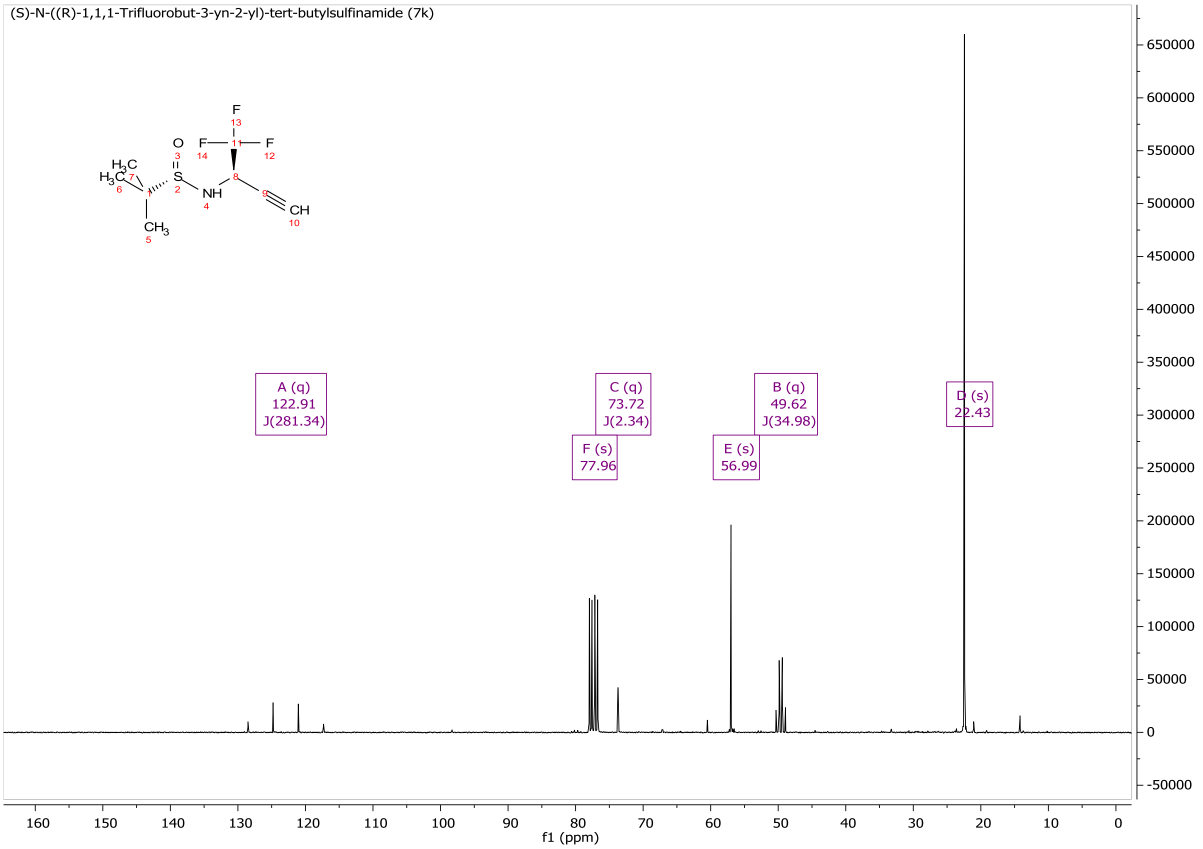
(S)-N-((R)-1,1,1-Trifluorobut-3-yn-2-yl)-tert-butylsulfinamide (7k)
A (q)
122.91
J(281.34)
C (q)
73.72
J(2.34)
F (s)
77.96
E (s)
56.99
B (q)
49.62
J(34.98)
D (s)
22.43
f1 (ppm)

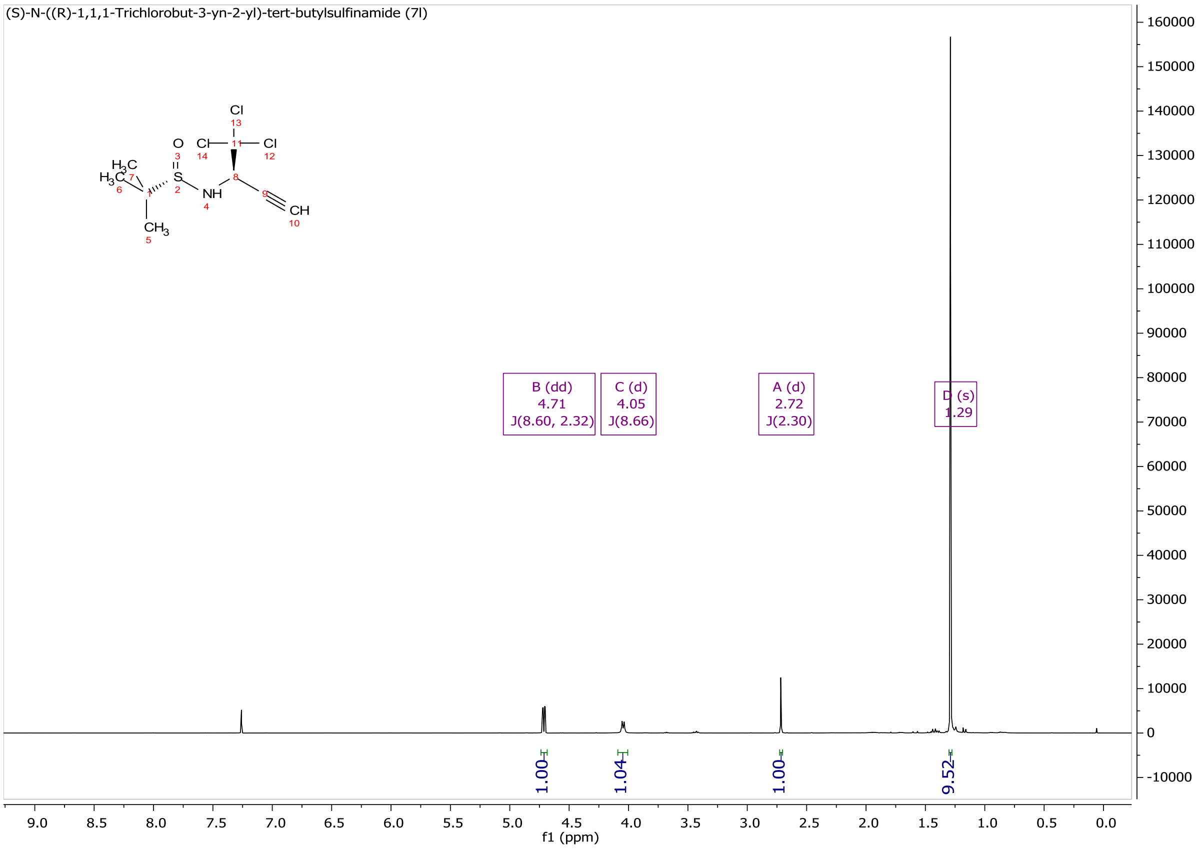
(S)-N-((R)-1,1,1-Trichlorobut-3-yn-2-yl)-tert-butylsulfinamide (7l)
B (dd)
4.71
J(8.60, 2.32)
C (d)
4.05
J(8.66)
A (d)
2.72
J(2.30)
D (s)
1.29
1.00
1.04
1.00
9.52
f1 (ppm)

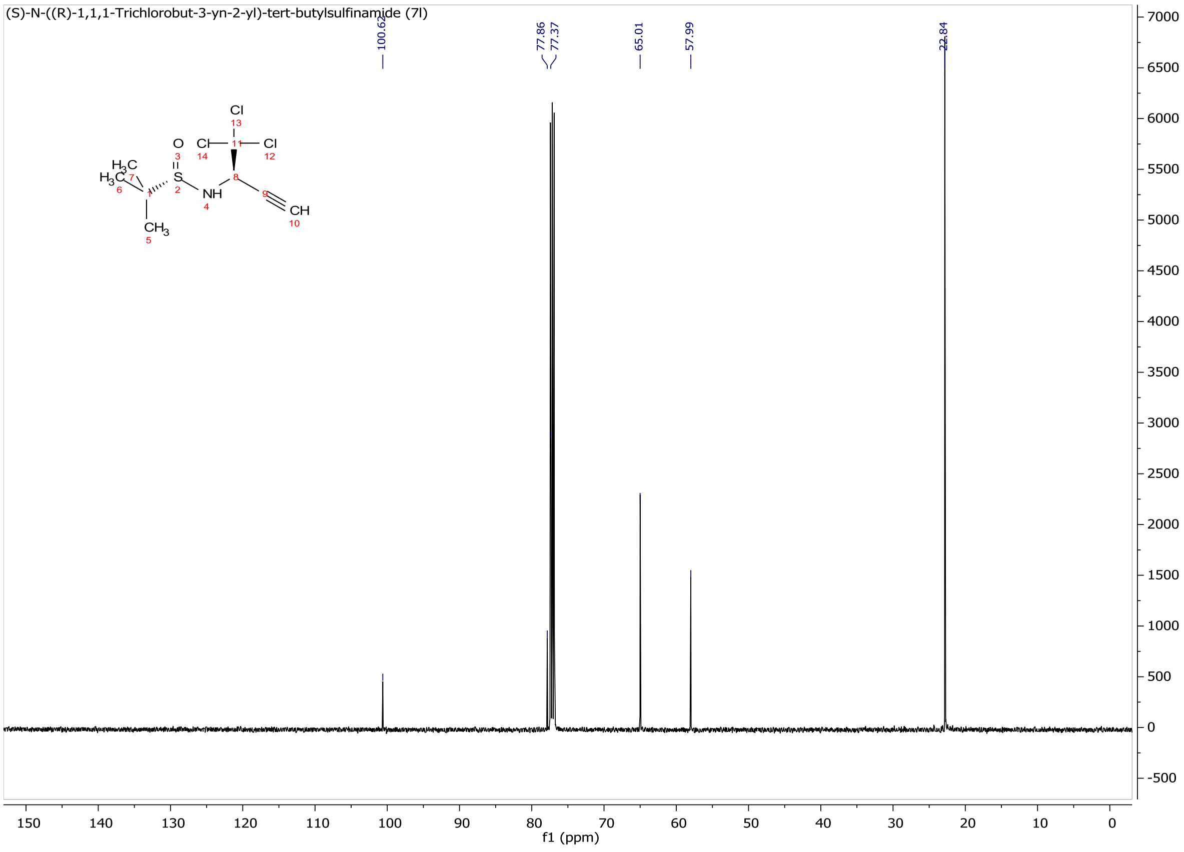
(S)-N-((R)-1,1,1-Trichlorobut-3-yn-2-yl)-tert-butylsulfinamide (7l)
100.62
77.86
77.37
65.01
57.99
22.84
f1 (ppm)

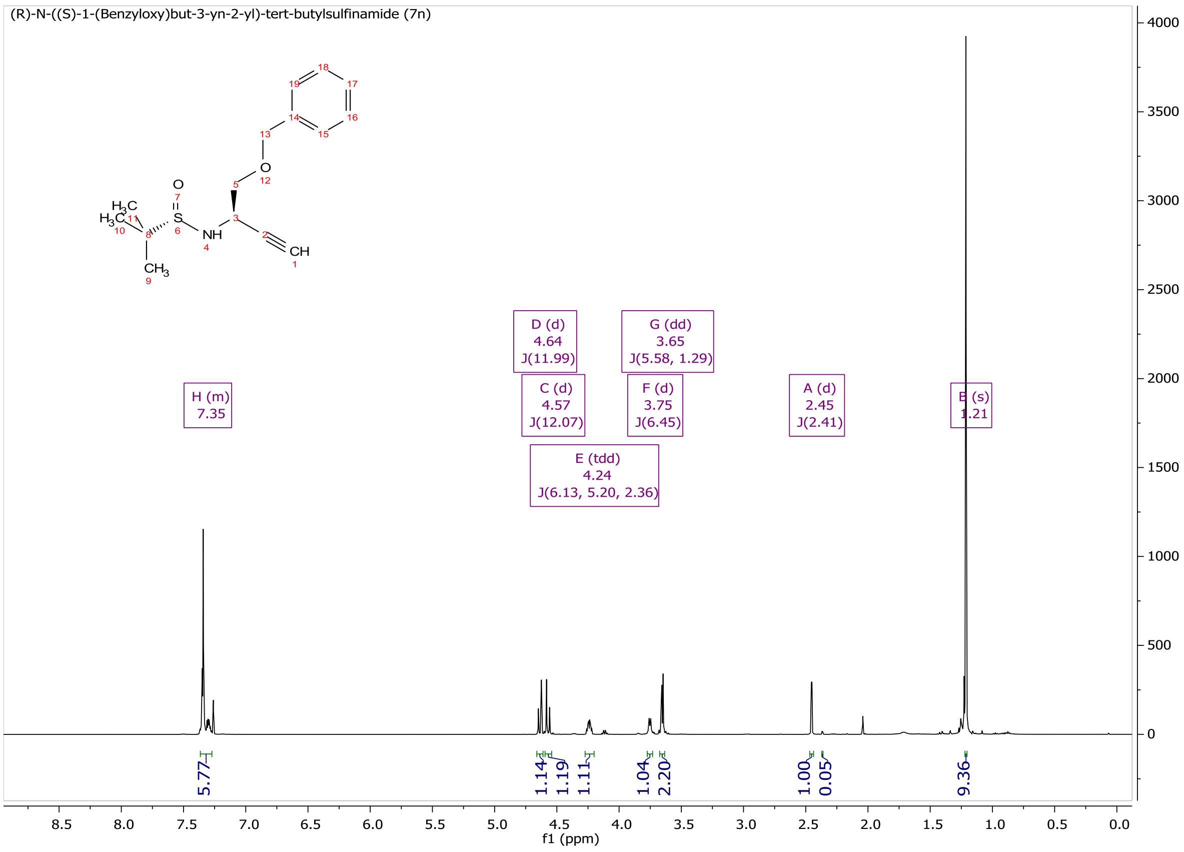
(R)-N-((S)-1-(Benzyloxy)but-3-yn-2-yl)-tert-butylsulfinamide (7n)
D (d) 4.64 J(11.99)
G (dd) 3.65 J(5.58, 1.29)
H (m) 7.35
C (d) 4.57 J(12.07)
F (d) 3.75 J(6.45)
A (d) 2.45 J(2.41)
B (s) 1.21
E (tdd) 4.24 J(6.13, 5.20, 2.36)
5.77
1.14
1.19
1.11
1.04
2.20
1.00
0.05
9.36
f1 (ppm)

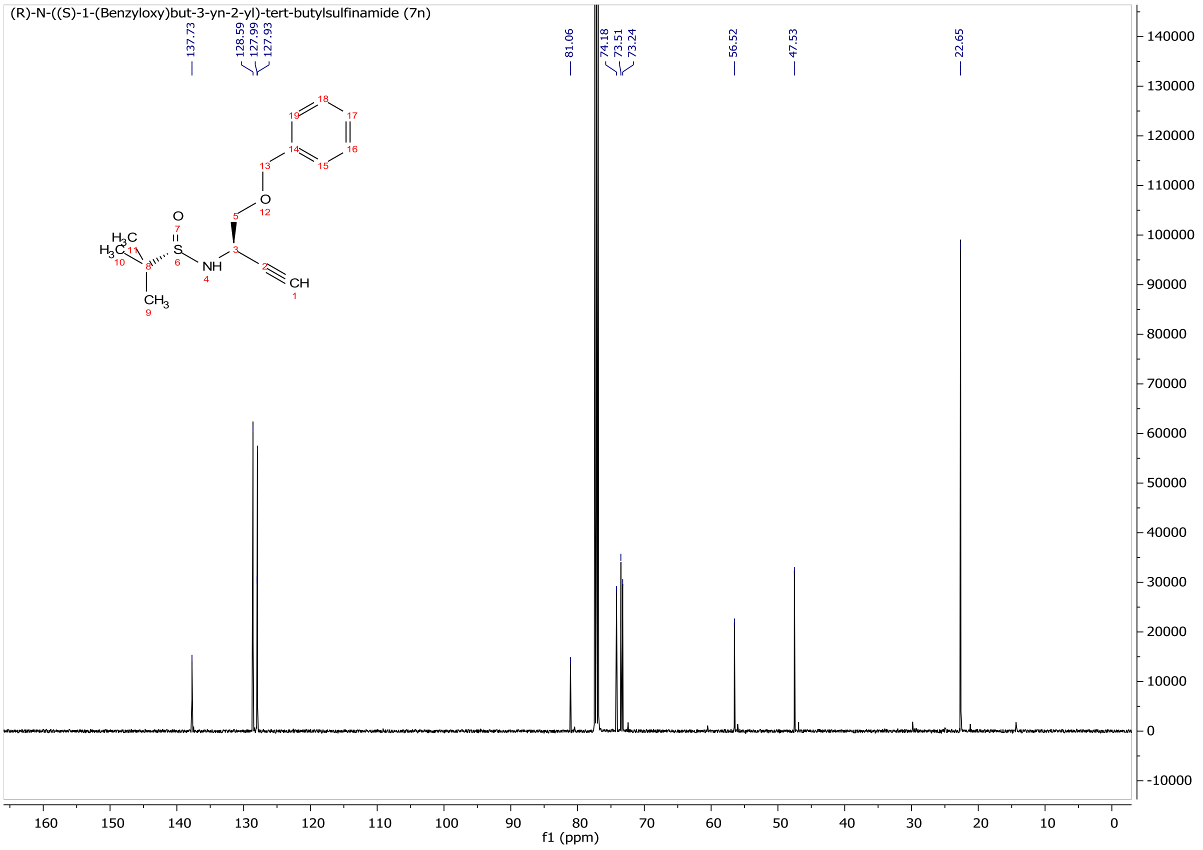
(R)-N-((S)-1-(Benzyloxy)but-3-yn-2-yl)-tert-butylsulfinamide (7n)
137.73
128.59
127.99
127.93
81.06
74.18
73.51
73.24
56.52
47.53
22.65
f1 (ppm)

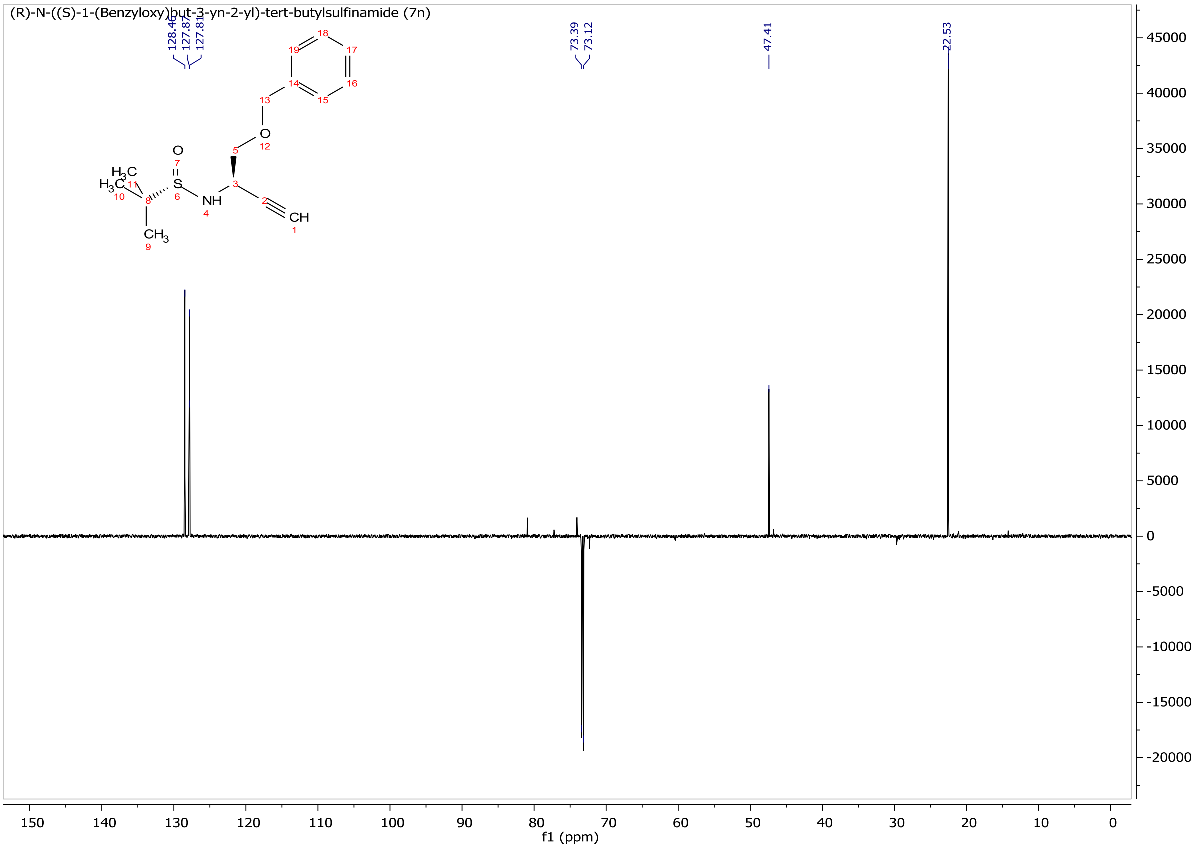
(R)-N-((S)-1-(Benzyloxy)but-3-yn-2-yl)-tert-butylsulfinamide (7n)
73.39
73.12
47.41
22.53
f1 (ppm)

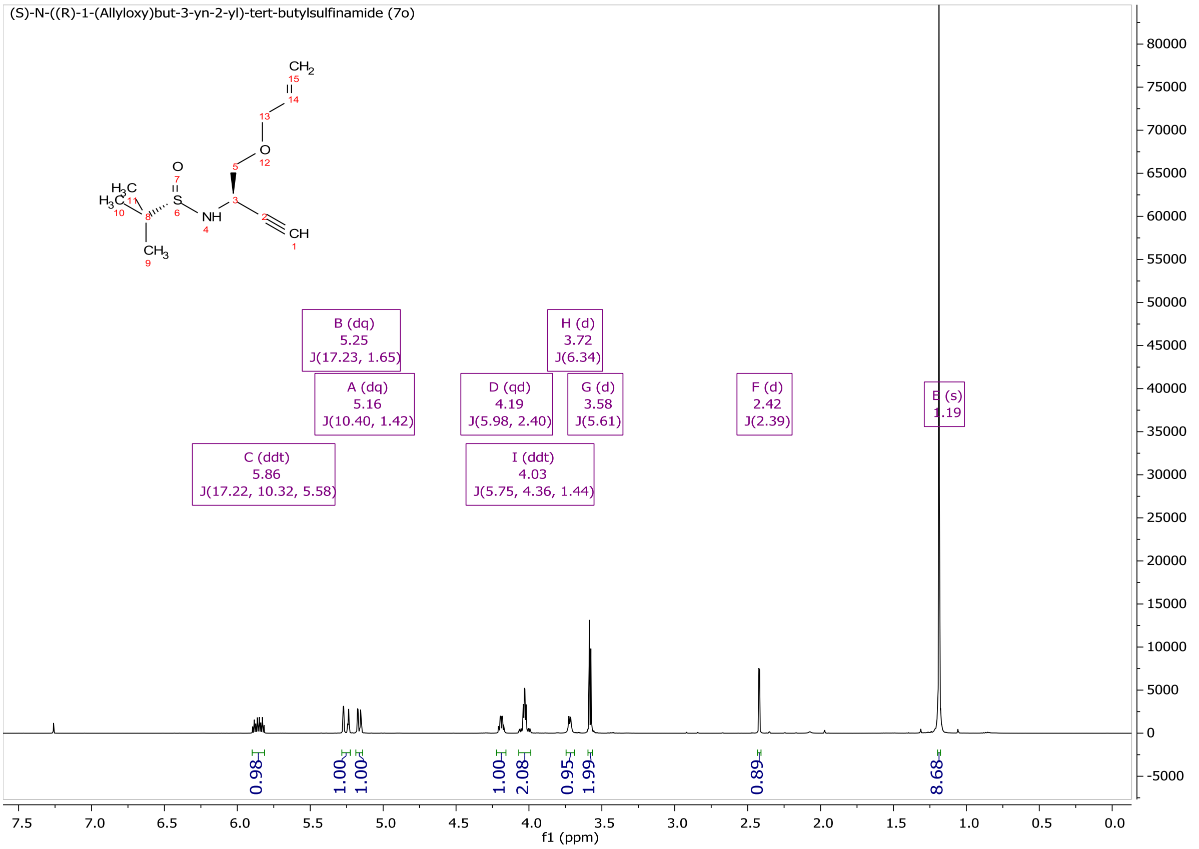
(S)-N-((R)-1-(Allyloxy)but-3-yn-2-yl)-tert-butylsulfinamide (7o)
B (dq) 5.25 J(17.23, 1.65)
H (d) 3.72 J(6.34)
A (dq) 5.16 J(10.40, 1.42)
D (qd) 4.19 J(5.98, 2.40)
G (d) 3.58 J(5.61)
F (d) 2.42 J(2.39)
E (s) 1.19
C (ddt) 5.86 J(17.22, 10.32, 5.58)
I (ddt) 4.03 J(5.75, 4.36, 1.44)
0.98
1.00
1.00
1.00
2.08
0.95
1.99
0.89
8.68
f1 (ppm)

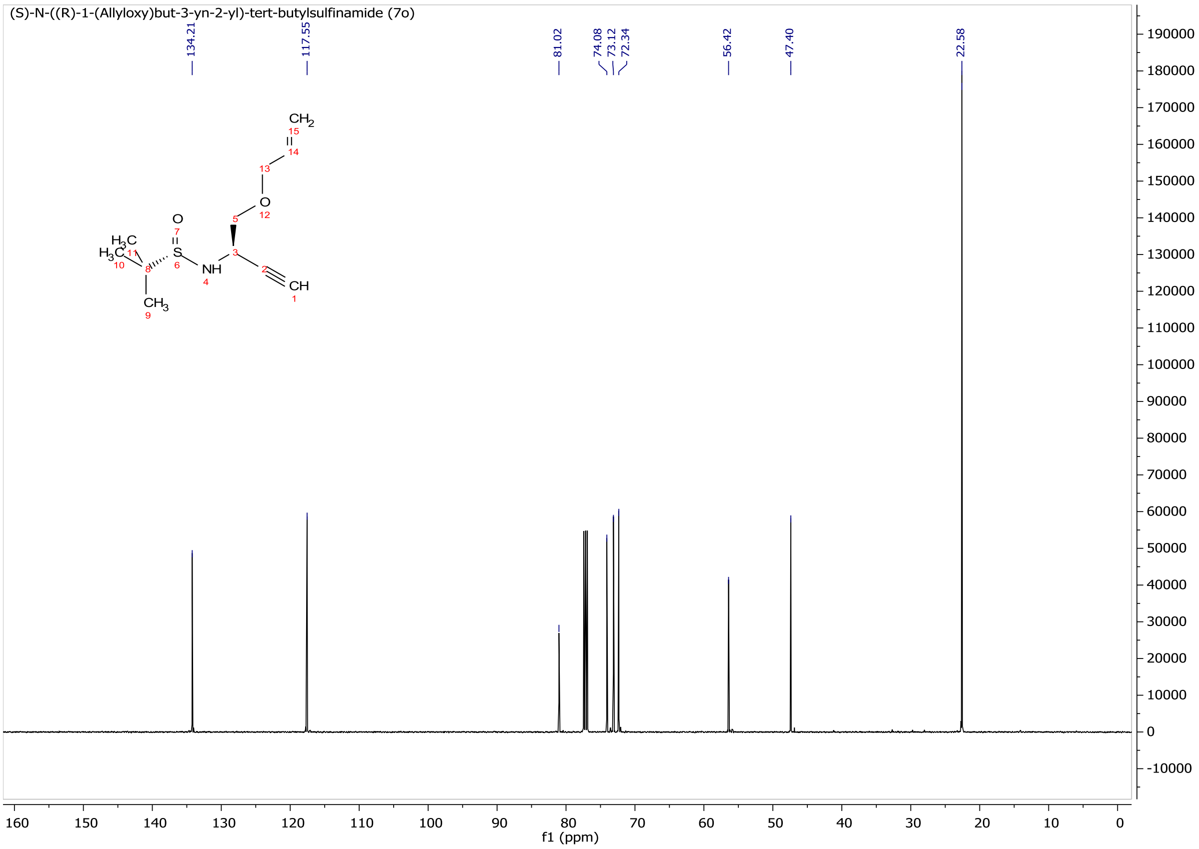
(S)-N-((R)-1-(Allyloxy)but-3-yn-2-yl)-tert-butylsulfinamide (7o)
134.21
117.55
81.02
74.08
73.12
72.34
56.42
47.40
22.58
f1 (ppm)

(S)-N-((R)-1-(Allyloxy)but-3-yn-2-yl)-tert-butylsulfinamide (7o)

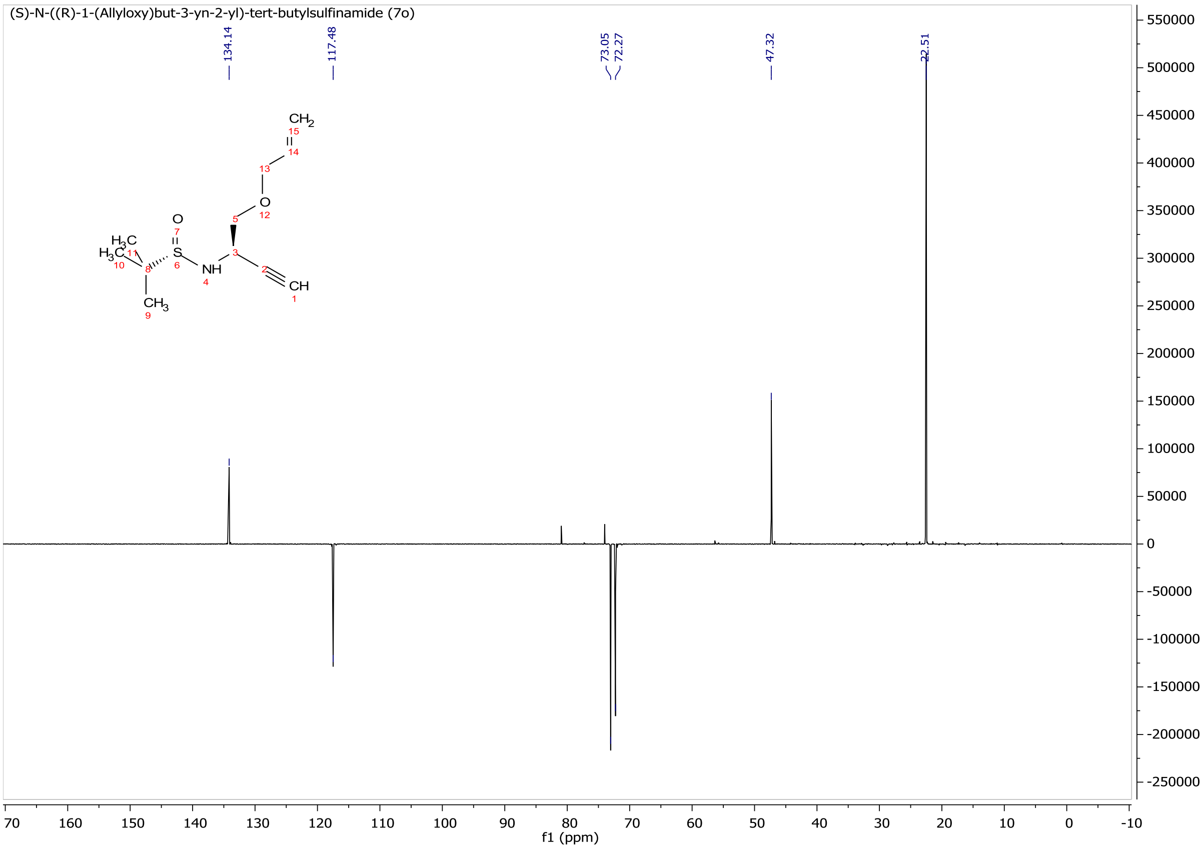

(R)-N-((S)-1-(Benzylthio)but-3-yn-2-yl)-tert-butylsulfinamide (7p)

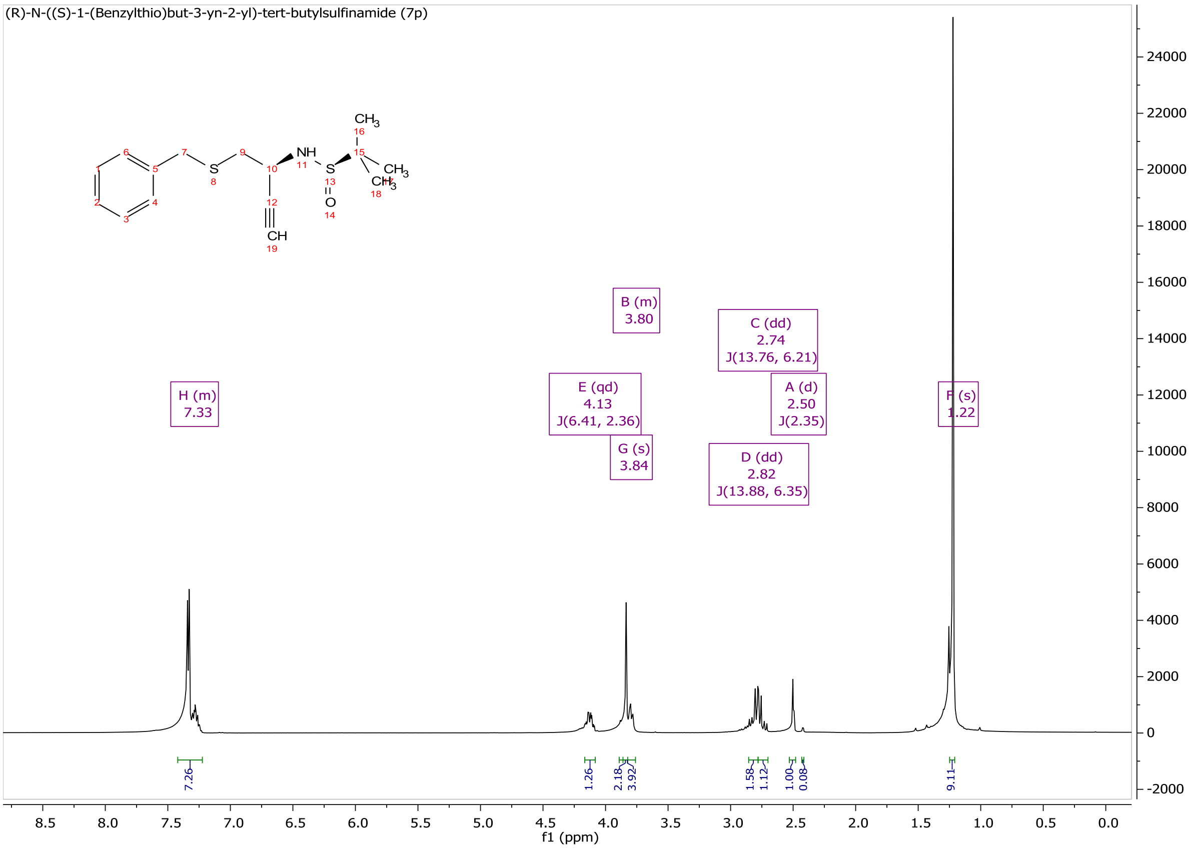

(R)-N-((S)-1-(Benzylthio)but-3-yn-2-yl)-tert-butylsulfinamide (7p)

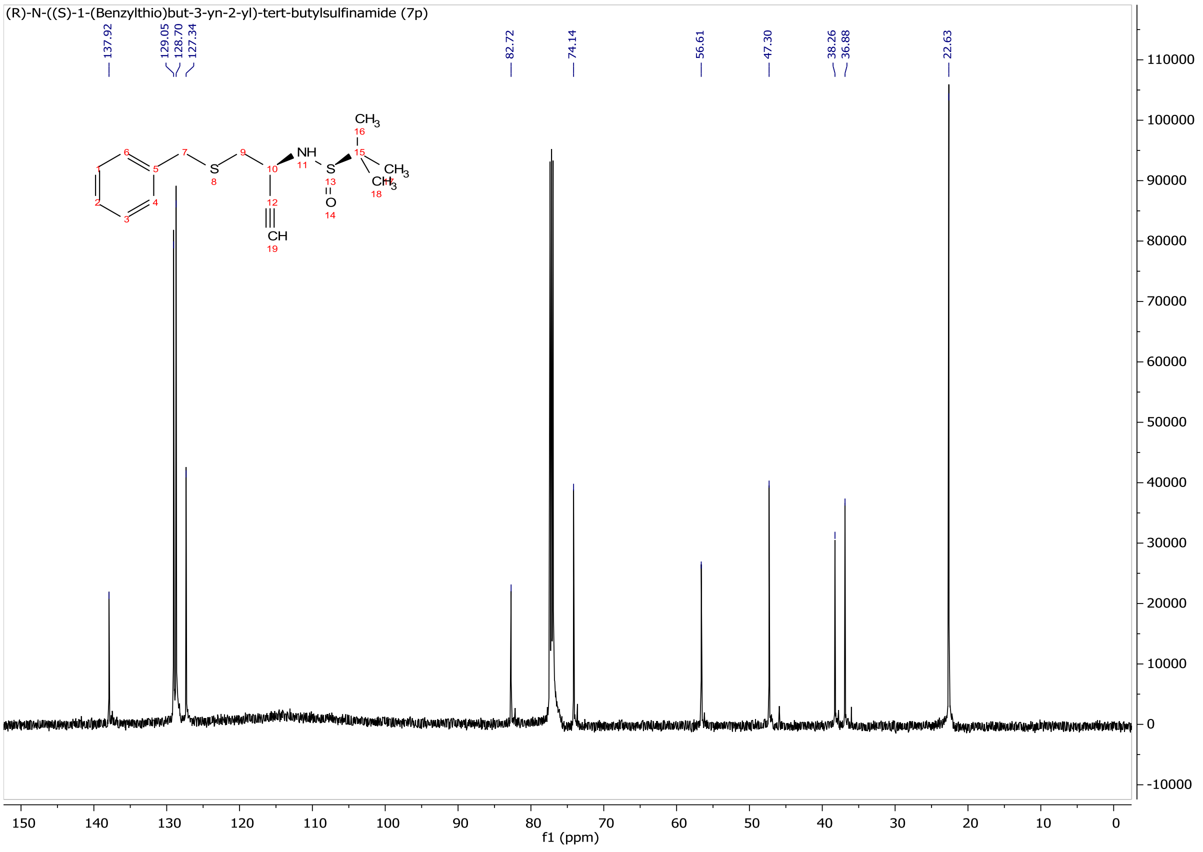

(S)-N-((S)-6-Cyanohex-1-yn-3-yl)-tert-butylsulfinamide (7q)

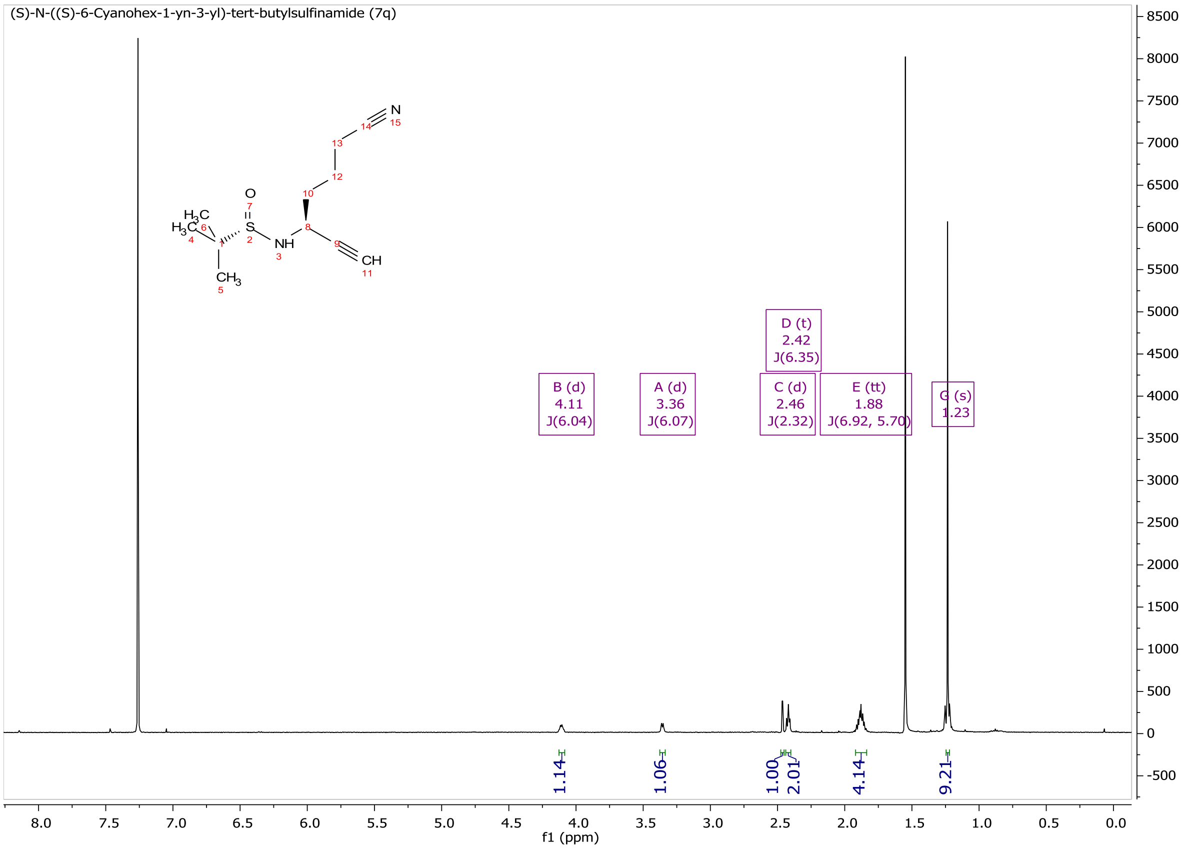

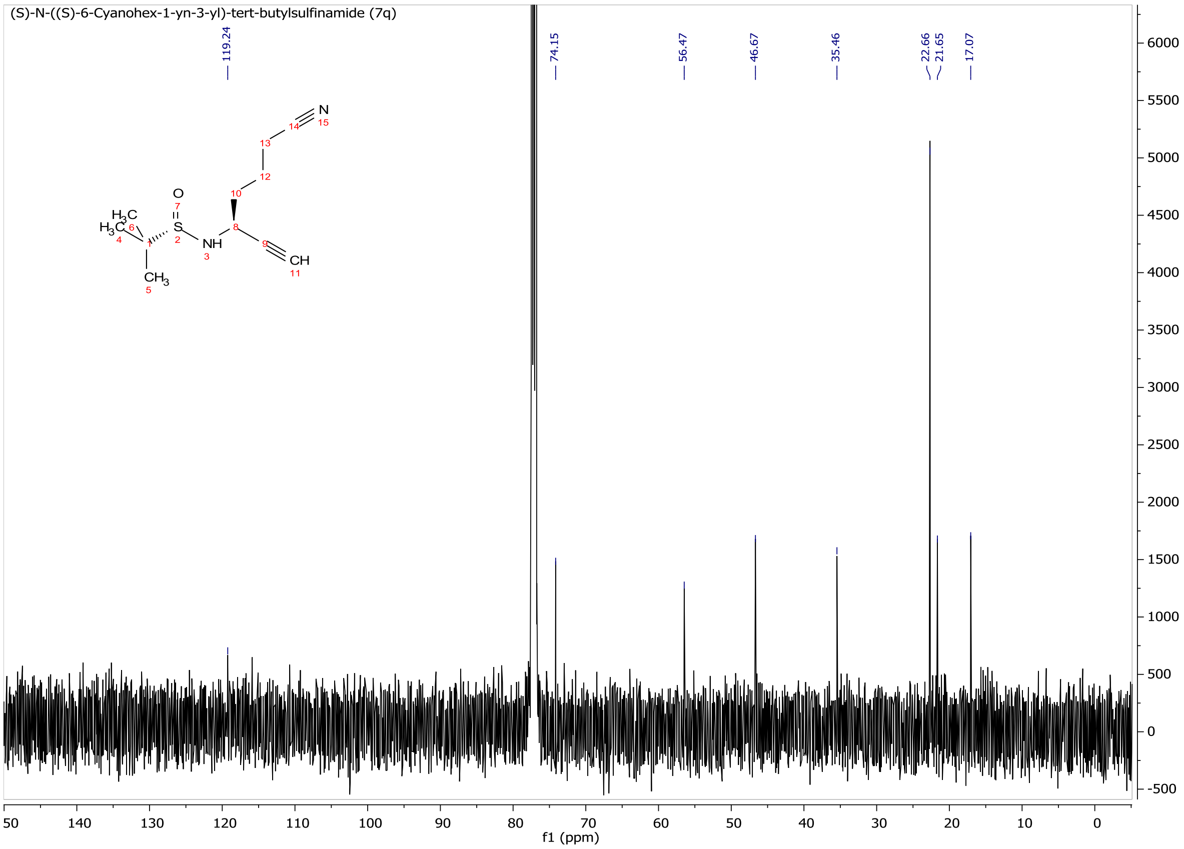
(S)-N-((S)-6-Cyanohex-1-yn-3-yl)-tert-butylsulfinamide (7q)
119.24
74.15
56.47
46.67
35.46
22.66
21.65
17.07
f1 (ppm)

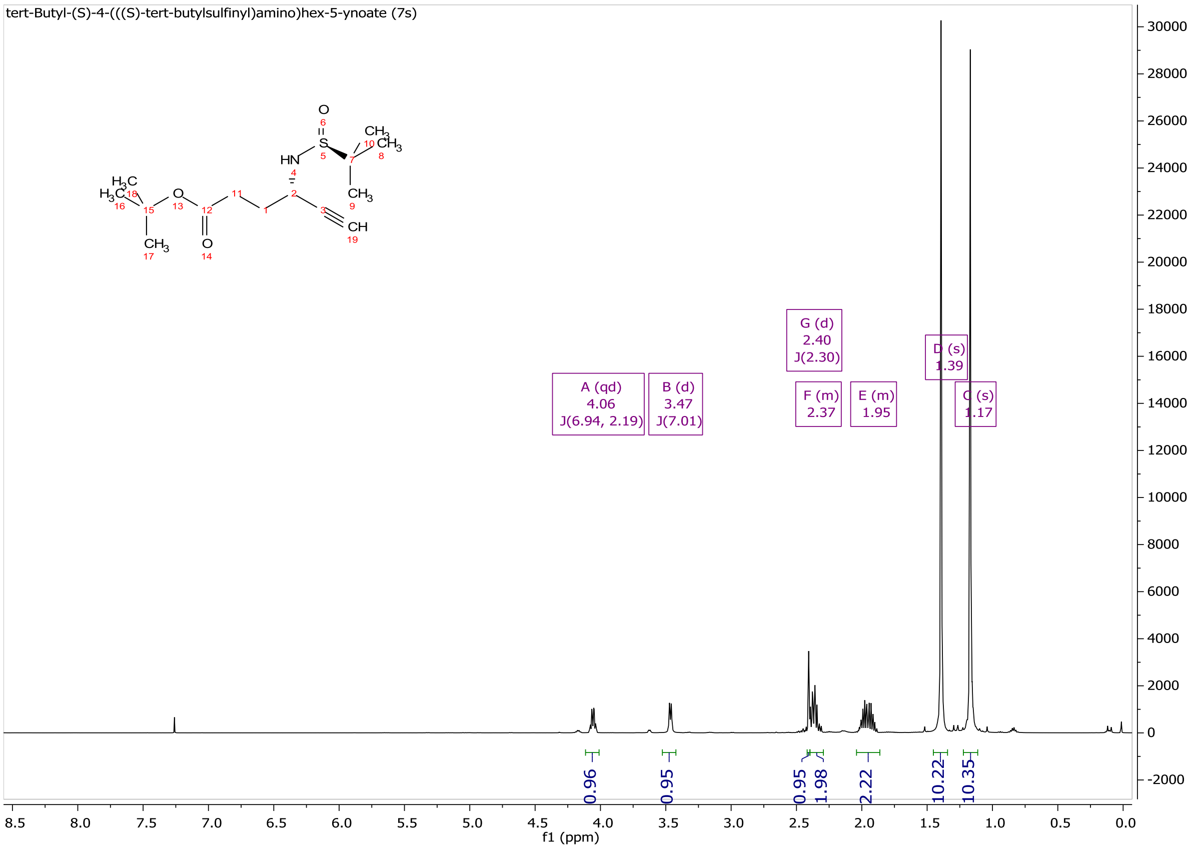
tert-Butyl-(S)-4-(((S)-tert-butylsulfinyl)amino)hex-5-ynoate (7s)
A (qd) 4.06 J(6.94, 2.19)
B (d) 3.47 J(7.01)
G (d) 2.40 J(2.30)
F (m) 2.37
E (m) 1.95
D (s) 1.39
C (s) 1.17
0.96
0.95
0.95
1.98
2.22
10.22
10.35
f1 (ppm)

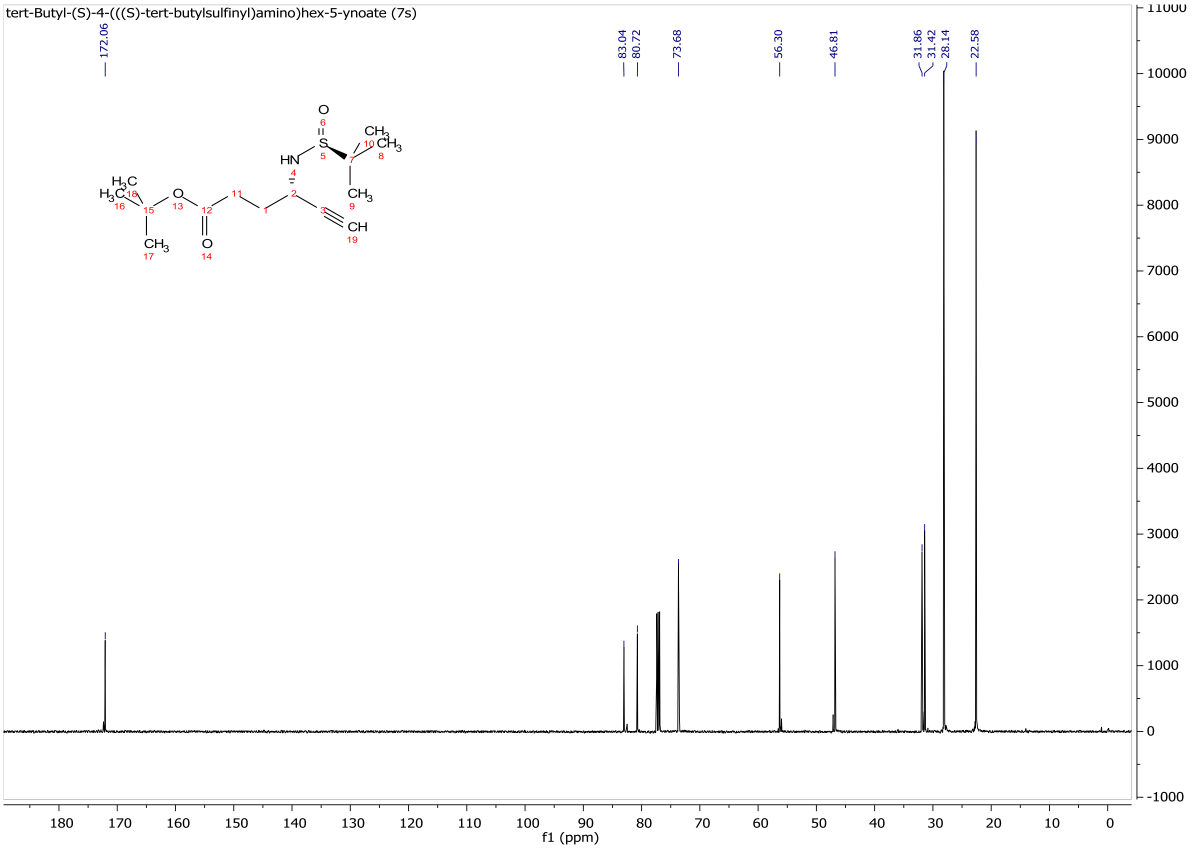
tert-Butyl-(S)-4-(((S)-tert-butylsulfinyl)amino)hex-5-ynoate (7s)
172.06
83.04
80.72
73.68
56.30
46.81
31.86
31.42
28.14
22.58
f1 (ppm)

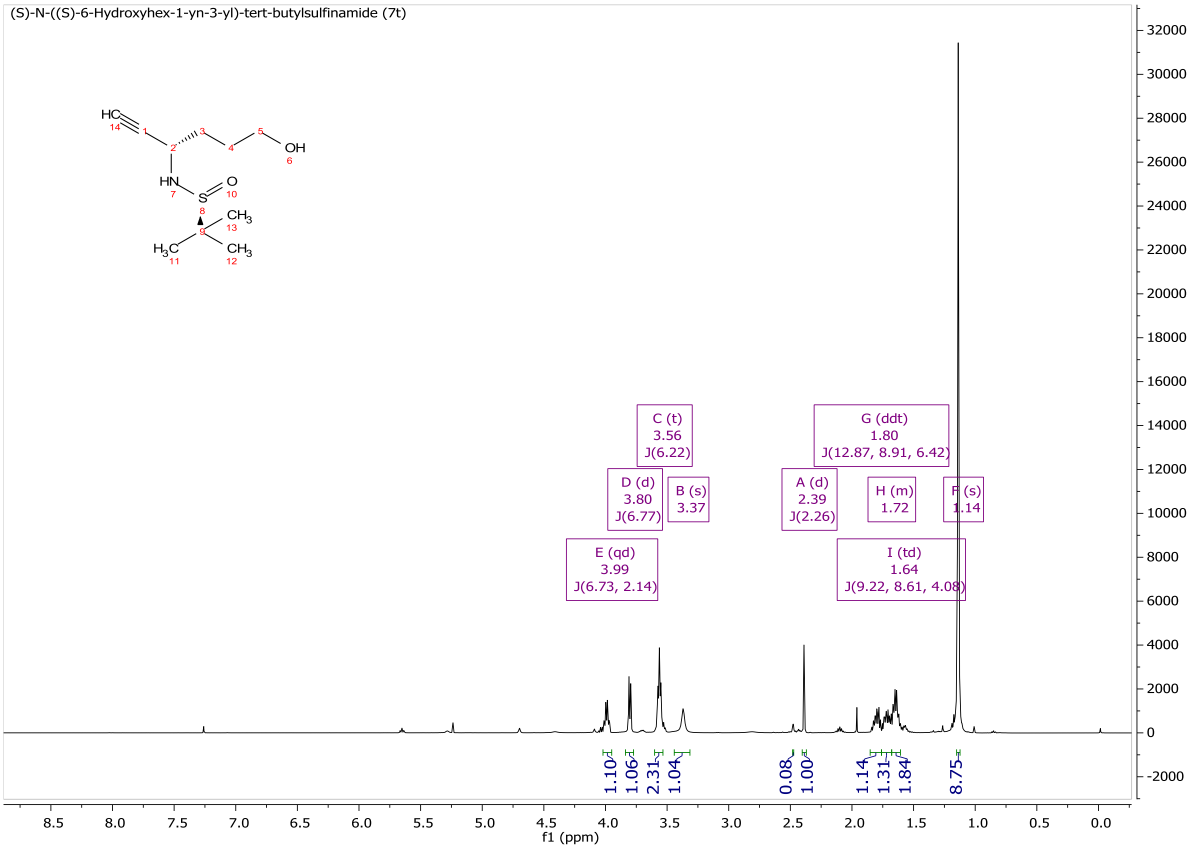
(S)-N-((S)-6-Hydroxyhex-1-yn-3-yl)-tert-butylsulfinamide (7t)
C (t) 3.56 J(6.22)
G (ddt) 1.80 J(12.87, 8.91, 6.42)
D (d) 3.80 J(6.77)
B (s) 3.37
A (d) 2.39 J(2.26)
H (m) 1.72
F (s) 1.14
E (qd) 3.99 J(6.73, 2.14)
I (td) 1.64 J(9.22, 8.61, 4.08)
1.10
1.06
2.31
1.04
0.08
1.00
1.14
1.31
1.84
8.75
f1 (ppm)

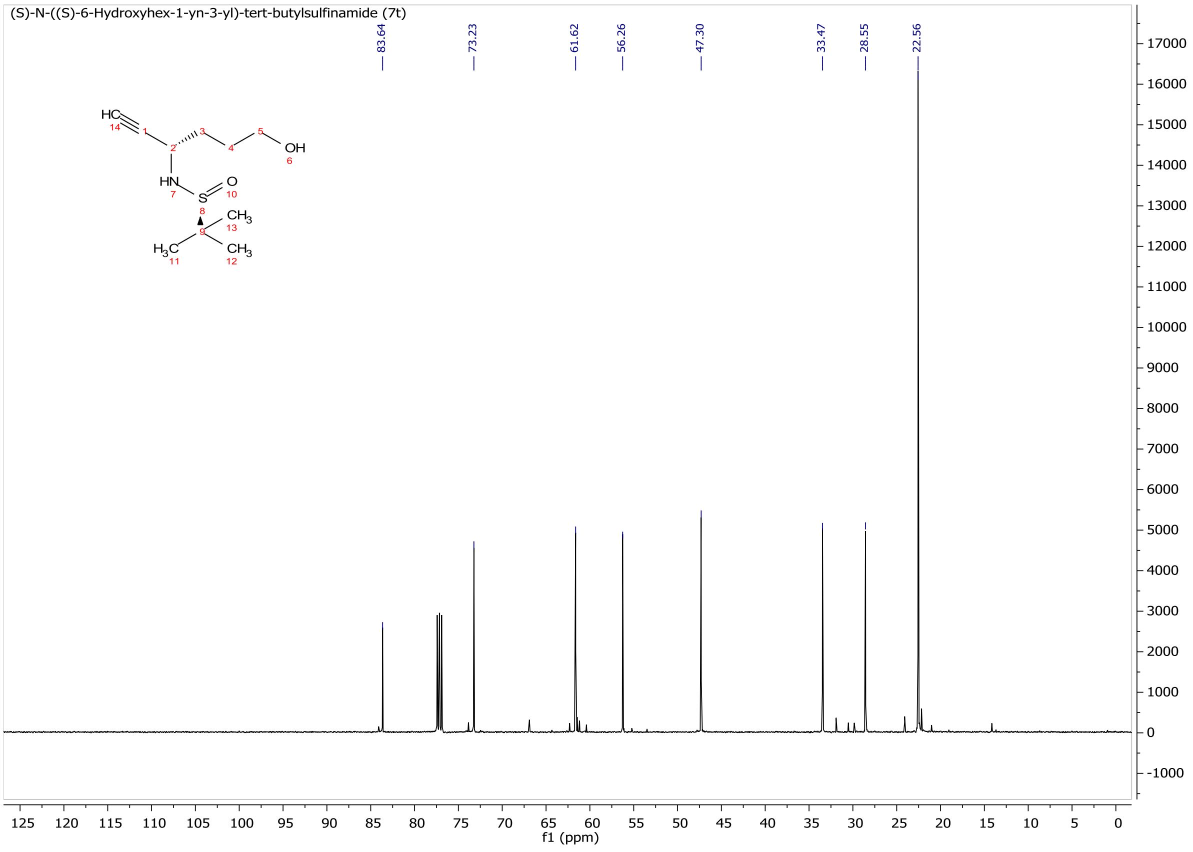
(S)-N-((S)-6-Hydroxyhex-1-yn-3-yl)-tert-butylsulfinamide (7t)
83.64
73.23
61.62
56.26
47.30
33.47
28.55
22.56
f1 (ppm)

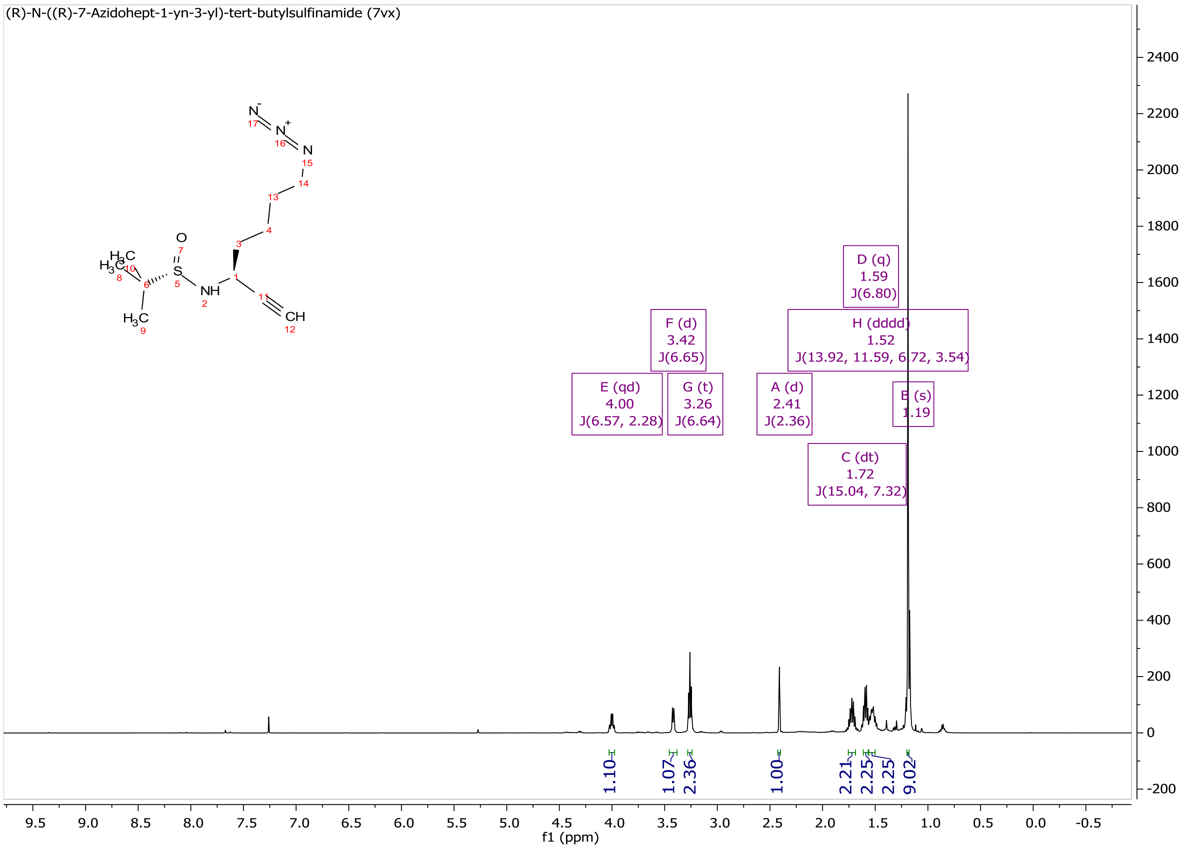
(R)-N-((R)-7-Azidohept-1-yn-3-yl)-tert-butylsulfinamide (7vx)
D (q)
1.59
J(6.80)
F (d)
3.42
J(6.65)
H (dddd)
1.52
J(13.92, 11.59, 6.72, 3.54)
E (qd)
4.00
J(6.57, 2.28)
G (t)
3.26
J(6.64)
A (d)
2.41
J(2.36)
B (s)
1.19
C (dt)
1.72
J(15.04, 7.32)
1.10
1.07
2.36
1.00
2.21
2.25
2.25
9.02
f1 (ppm)

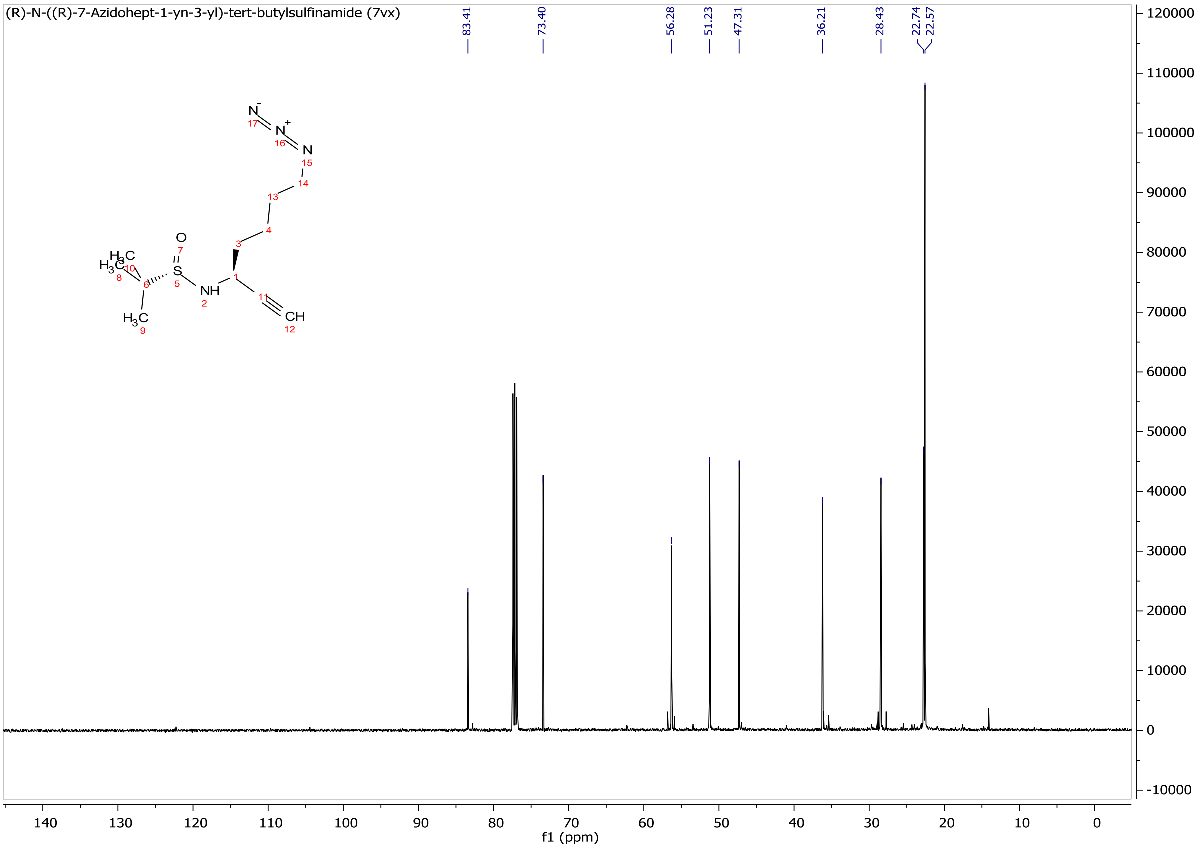
(R)-N-((R)-7-Azidohept-1-yn-3-yl)-tert-butylsulfinamide (7vx)
83.41
73.40
56.28
51.23
47.31
36.21
28.43
22.74
22.57
f1 (ppm)

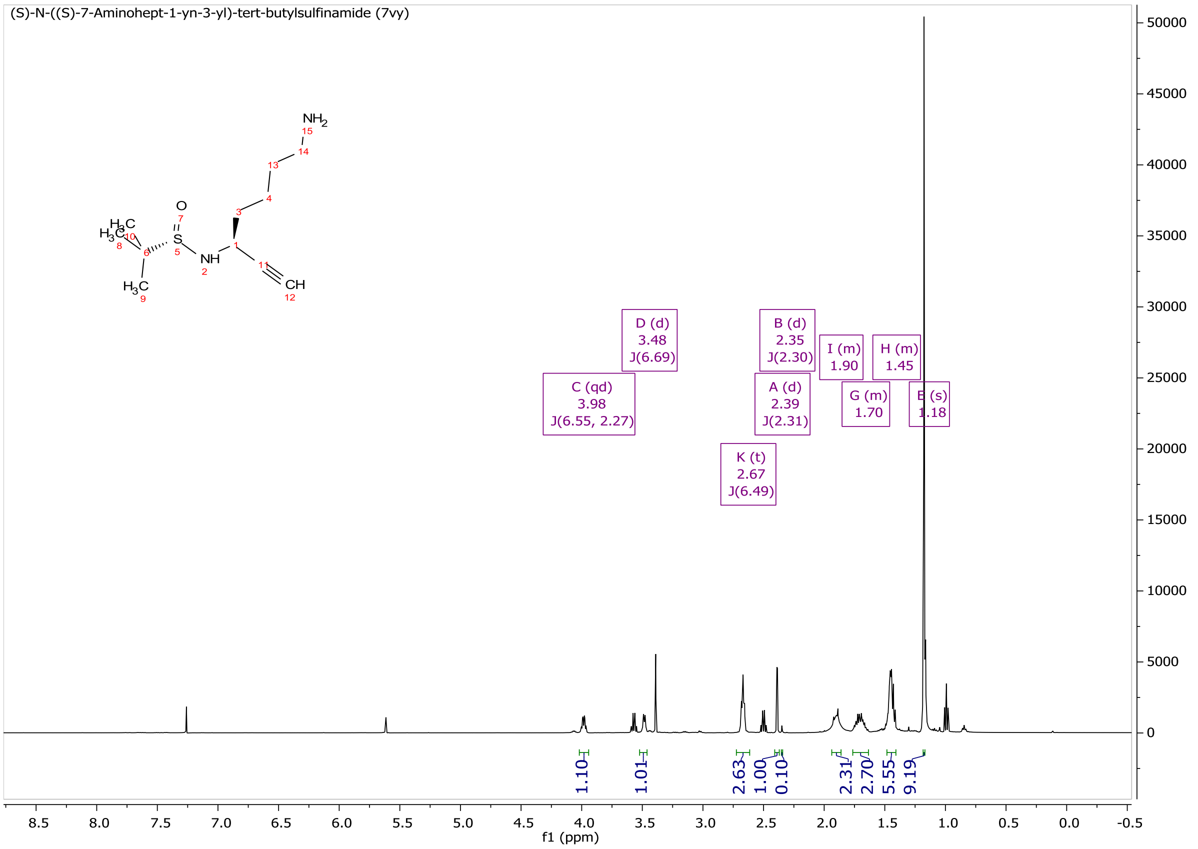
(S)-N-((S)-7-Aminohept-1-yn-3-yl)-tert-butylsulfinamide (7vy)
D (d) 3.48 J(6.69)
B (d) 2.35 J(2.30)
I (m) 1.90
H (m) 1.45
C (qd) 3.98 J(6.55, 2.27)
A (d) 2.39 J(2.31)
G (m) 1.70
E (s) 1.18
K (t) 2.67 J(6.49)
1.10
1.01
2.63
1.00
0.10
2.31
2.70
5.55
9.19
f1 (ppm)

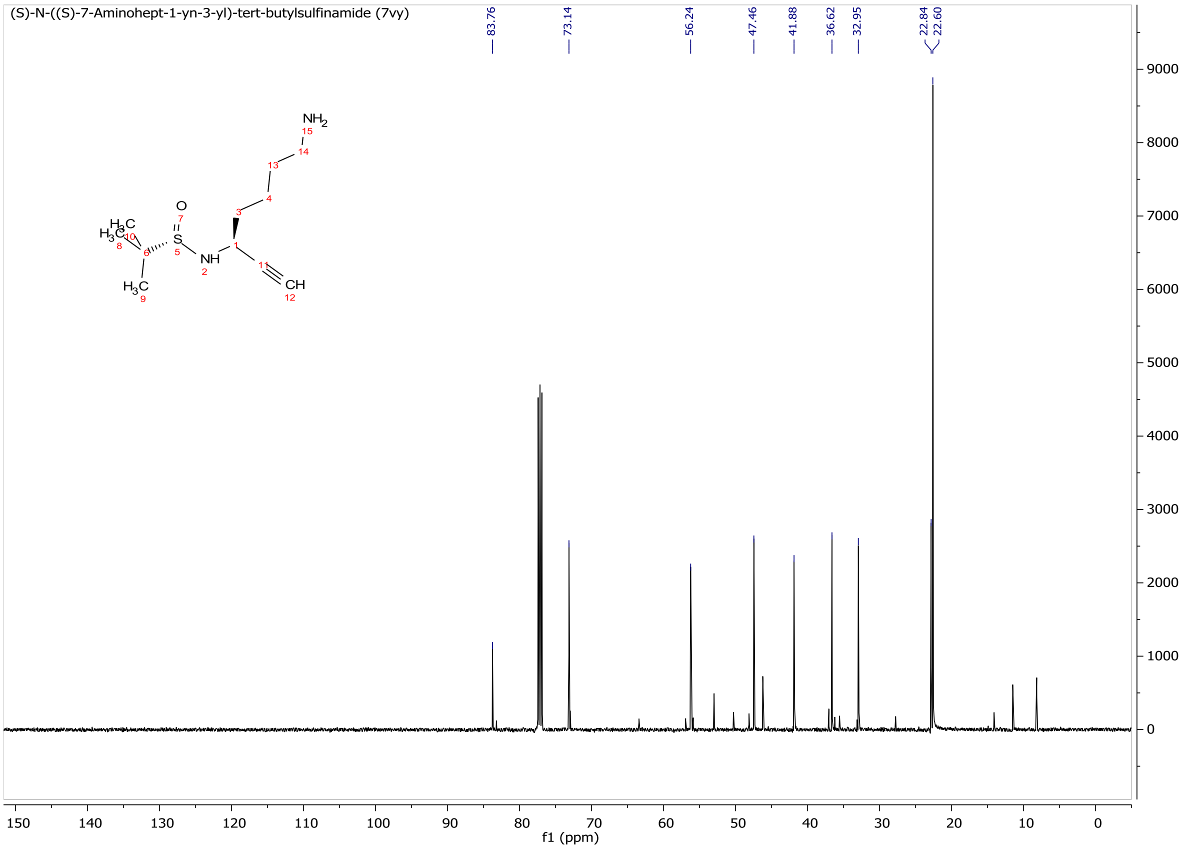
(S)-N-((S)-7-Aminohept-1-yn-3-yl)-tert-butylsulfinamide (7vy)
83.76
73.14
56.24
47.46
41.88
36.62
32.95
22.84
22.60
f1 (ppm)

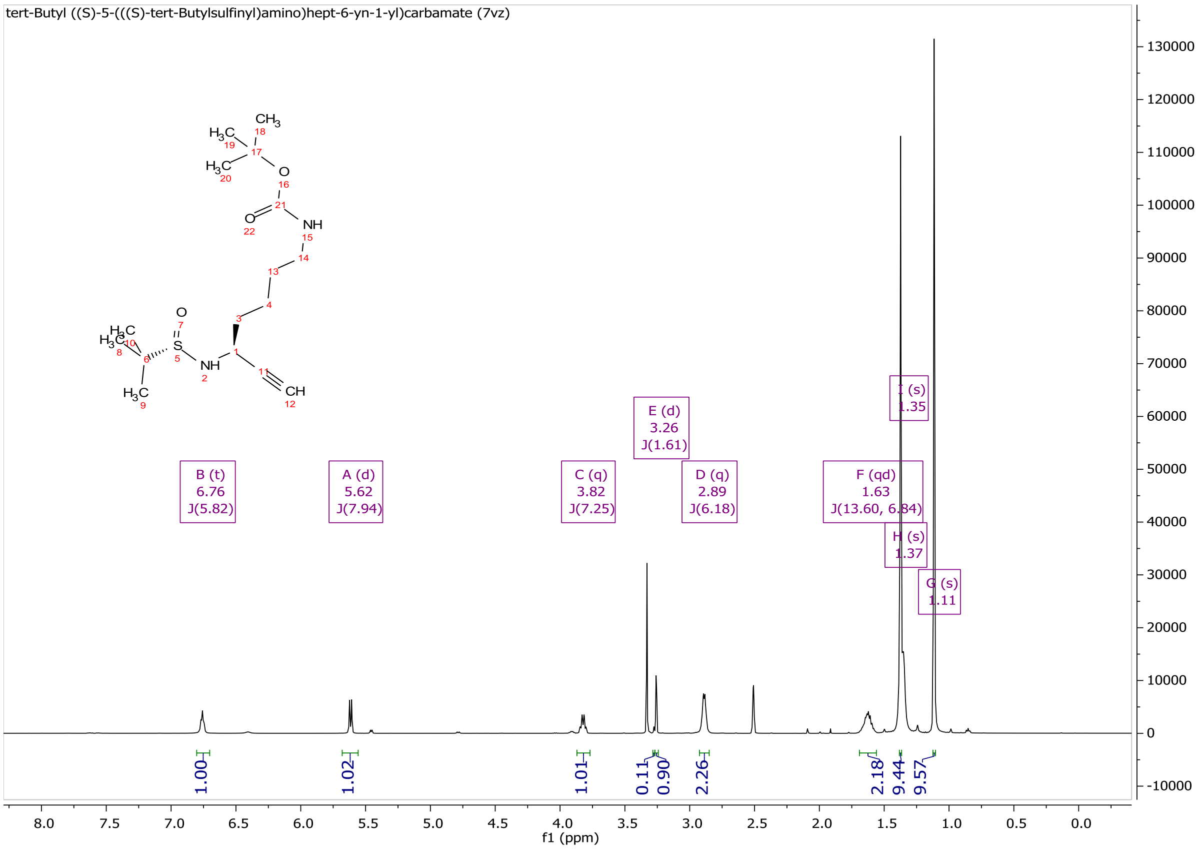
tert-Butyl ((S)-5-(((S)-tert-Butylsulfinyl)amino)hept-6-yn-1-yl)carbamate (7vz)
B (t) 6.76 J(5.82)
A (d) 5.62 J(7.94)
C (q) 3.82 J(7.25)
E (d) 3.26 J(1.61)
D (q) 2.89 J(6.18)
F (qd) 1.63 J(13.60, 6.84)
I (s) 1.35
H (s) 1.37
G (s) 1.11
1.00
1.02
1.01
0.11
0.90
2.26
2.18
9.44
9.57
f1 (ppm)

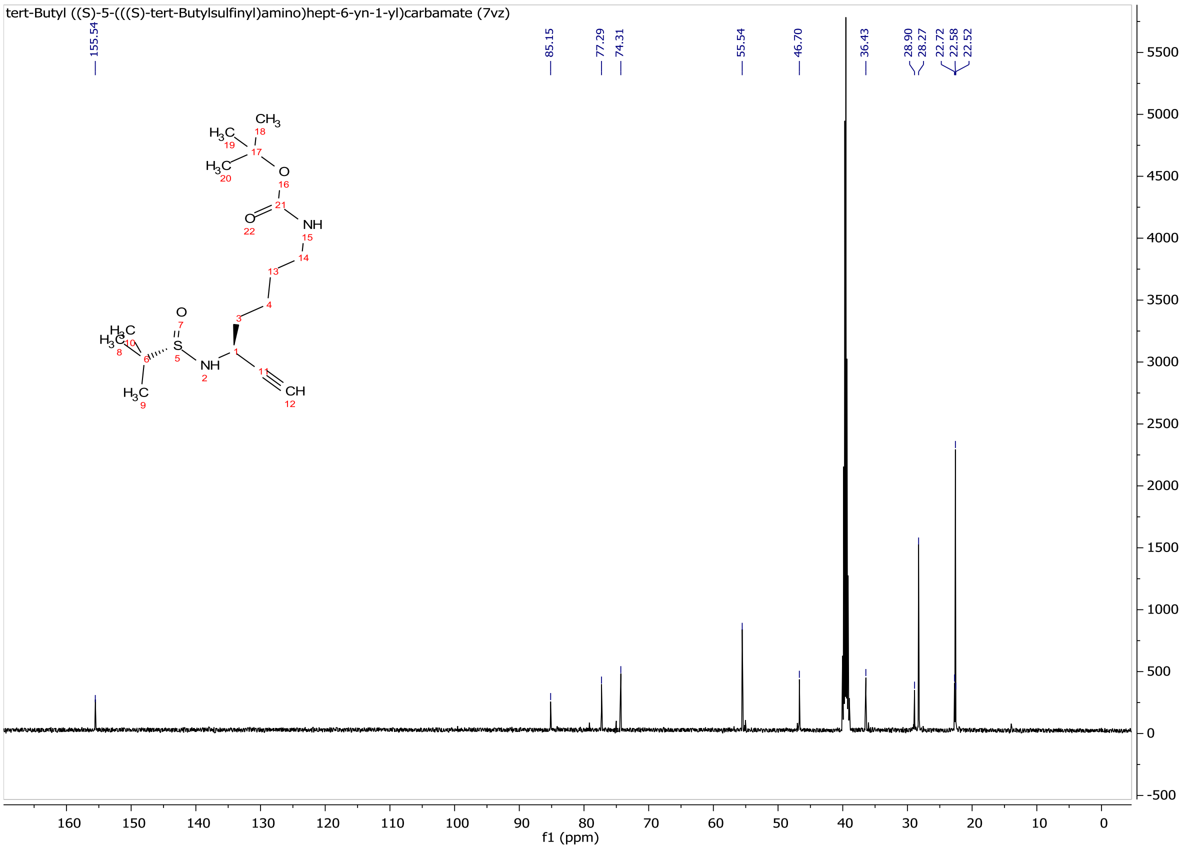
tert-Butyl ((S)-5-(((S)-tert-Butylsulfinyl)amino)hept-6-yn-1-yl)carbamate (7vz)
155.54
85.15
77.29
74.31
55.54
46.70
36.43
28.90
28.27
22.72
22.58
22.52
f1 (ppm)

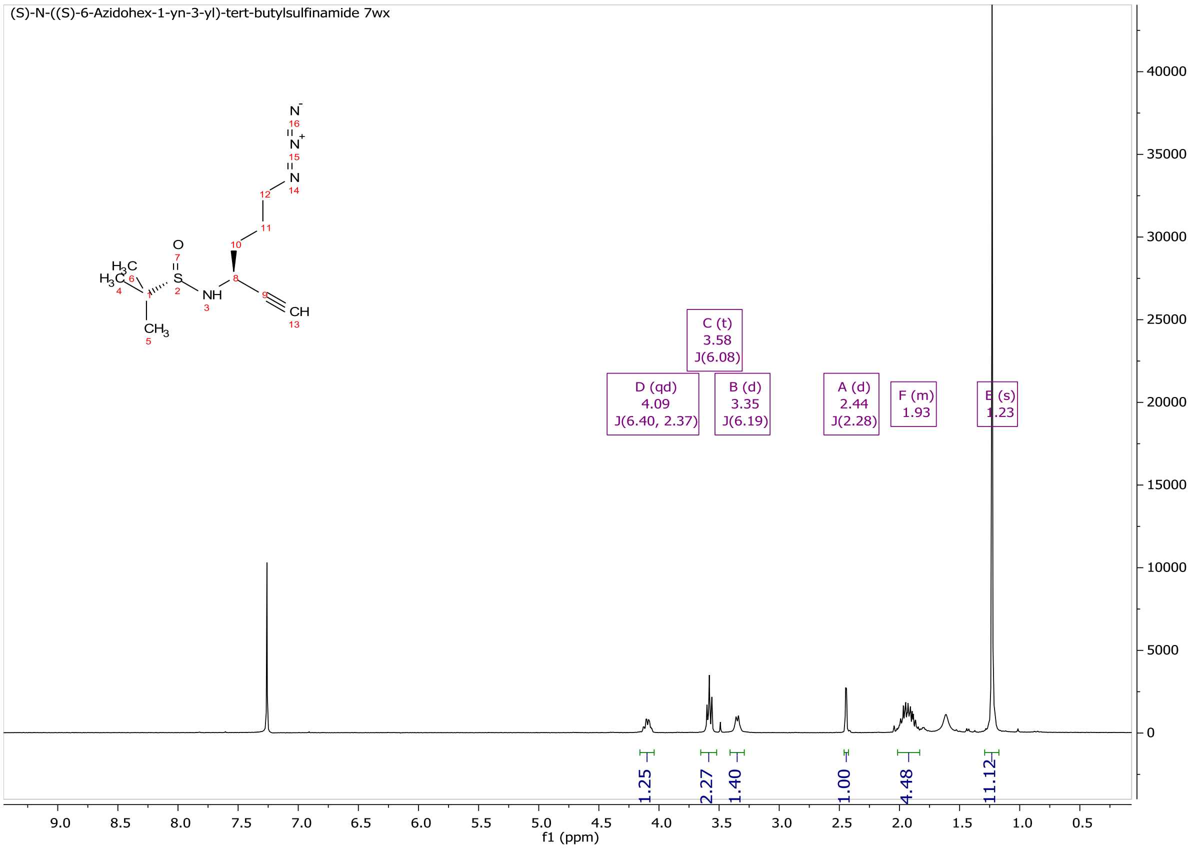
(S)-N-((S)-6-Azidohex-1-yn-3-yl)-tert-butylsulfinamide 7wx
C (t)
3.58
J(6.08)
D (qd)
4.09
J(6.40, 2.37)
B (d)
3.35
J(6.19)
A (d)
2.44
J(2.28)
F (m)
1.93
E (s)
1.23
1.25
2.27
1.40
1.00
4.48
11.12
f1 (ppm)

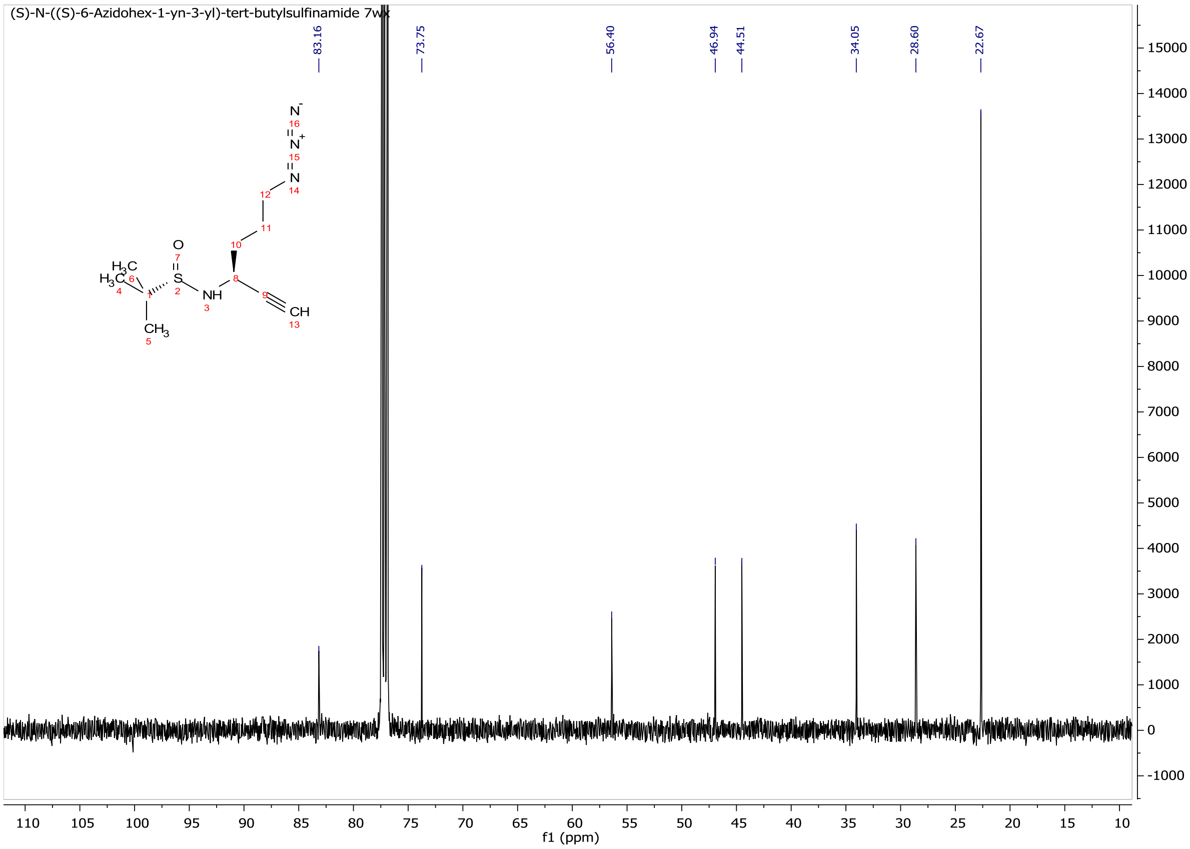
(S)-N-((S)-6-Azidohex-1-yn-3-yl)-tert-butylsulfinamide 7wx
83.16
73.75
56.40
46.94
44.51
34.05
28.60
22.67
f1 (ppm)

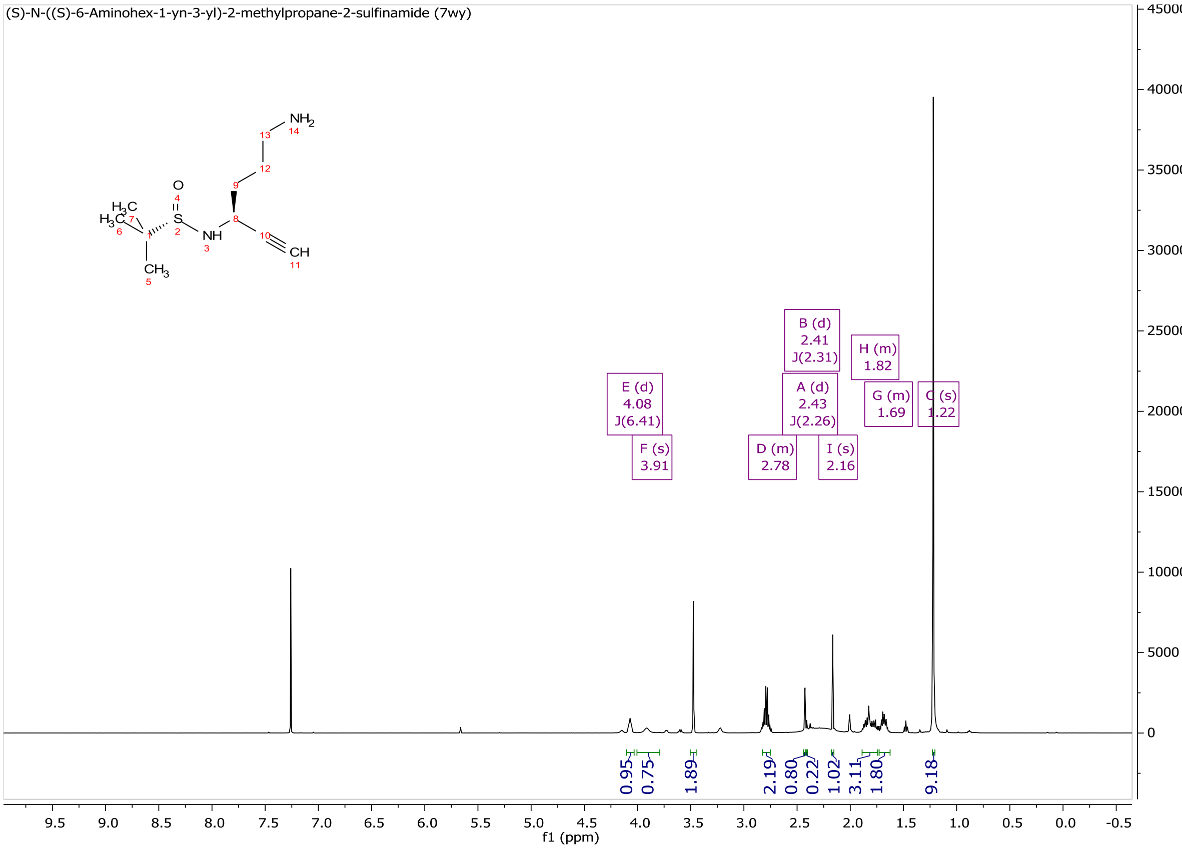
(S)-N-((S)-6-Aminohex-1-yn-3-yl)-2-methylpropane-2-sulfinamide (7wy)
B (d) 2.41 J(2.31)
H (m) 1.82
E (d) 4.08 J(6.41)
A (d) 2.43 J(2.26)
G (m) 1.69
C (s) 1.22
F (s) 3.91
D (m) 2.78
I (s) 2.16
0.95
0.75
1.89
2.19
0.80
0.22
1.02
3.11
1.80
9.18
f1 (ppm)

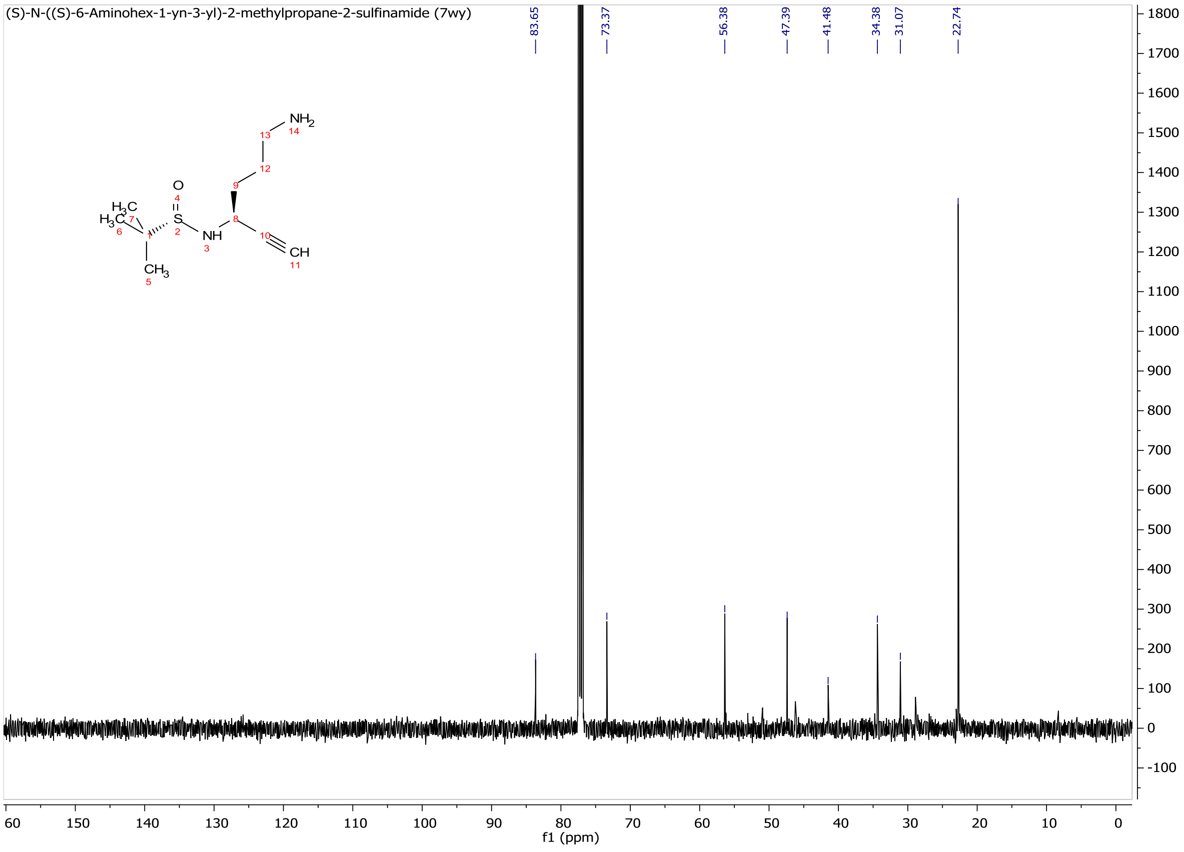
(S)-N-((S)-6-Aminohex-1-yn-3-yl)-2-methylpropane-2-sulfinamide (7wy)
83.65
73.37
56.38
47.39
41.48
34.38
31.07
22.74
f1 (ppm)

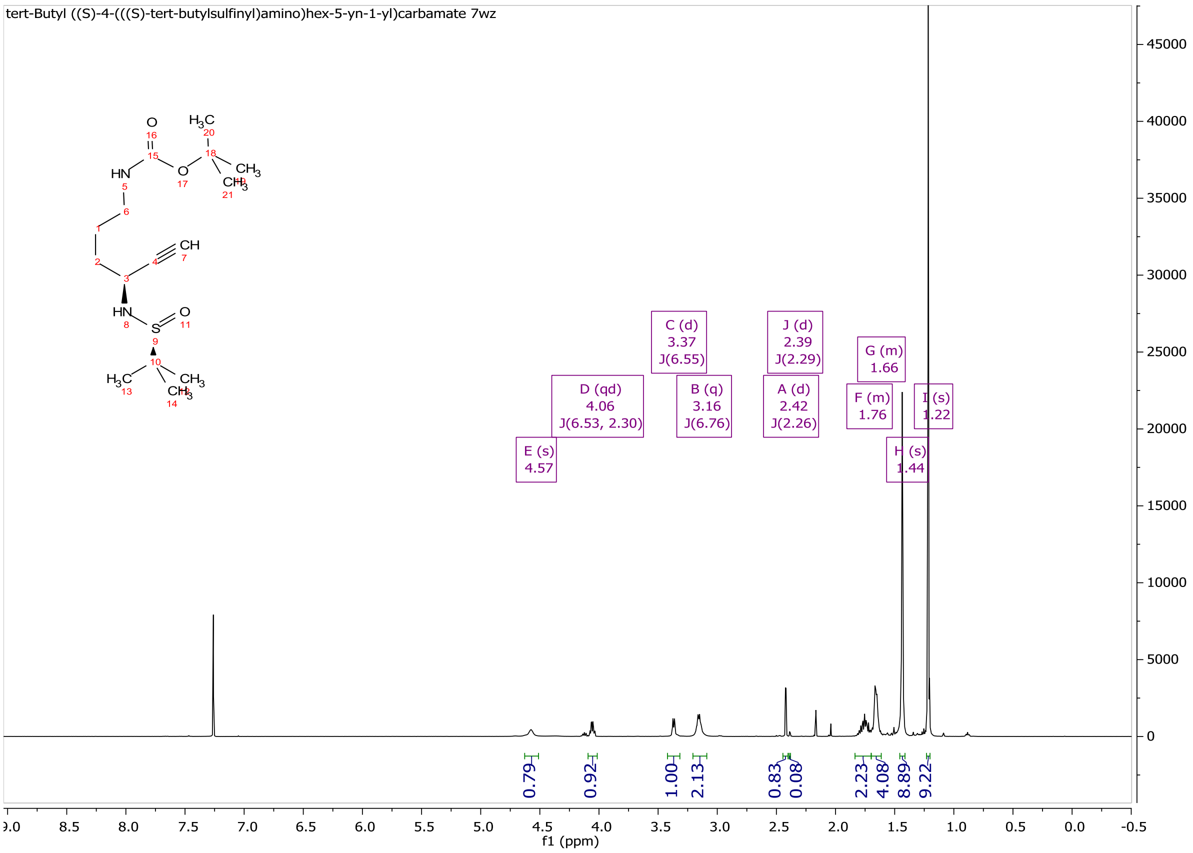
tert-Butyl ((S)-4-(((S)-tert-butylsulfinyl)amino)hex-5-yn-1-yl)carbamate 7wz
C (d) 3.37 J(6.55)
J (d) 2.39 J(2.29)
G (m) 1.66
D (qd) 4.06 J(6.53, 2.30)
B (q) 3.16 J(6.76)
A (d) 2.42 J(2.26)
F (m) 1.76
I (s) 1.22
E (s) 4.57
H (s) 1.44
0.79
0.92
1.00
2.13
0.83
0.08
2.23
4.08
8.89
9.22
f1 (ppm)

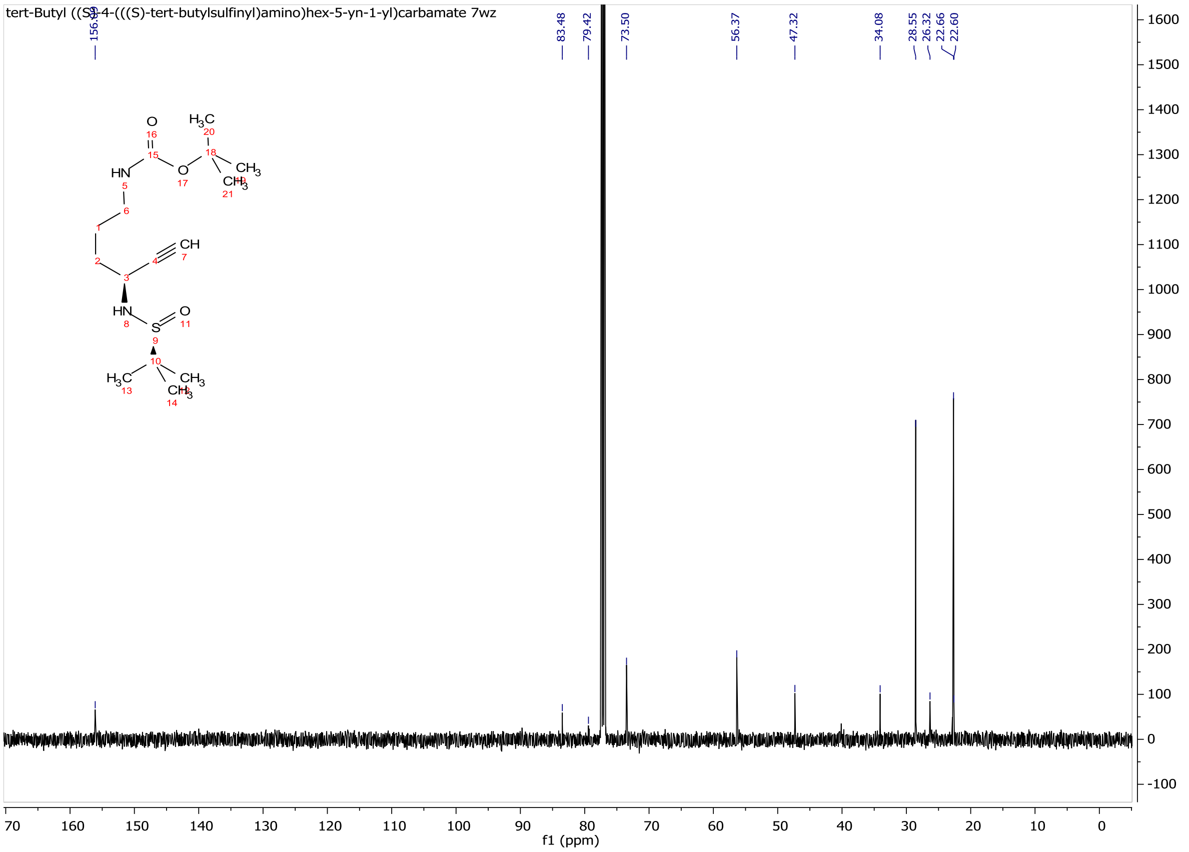
tert-Butyl ((S)-4-(((S)-tert-butylsulfinyl)amino)hex-5-yn-1-yl)carbamate 7wz
83.48
79.42
73.50
56.37
47.32
34.08
28.55
26.32
22.66
22.60
f1 (ppm)

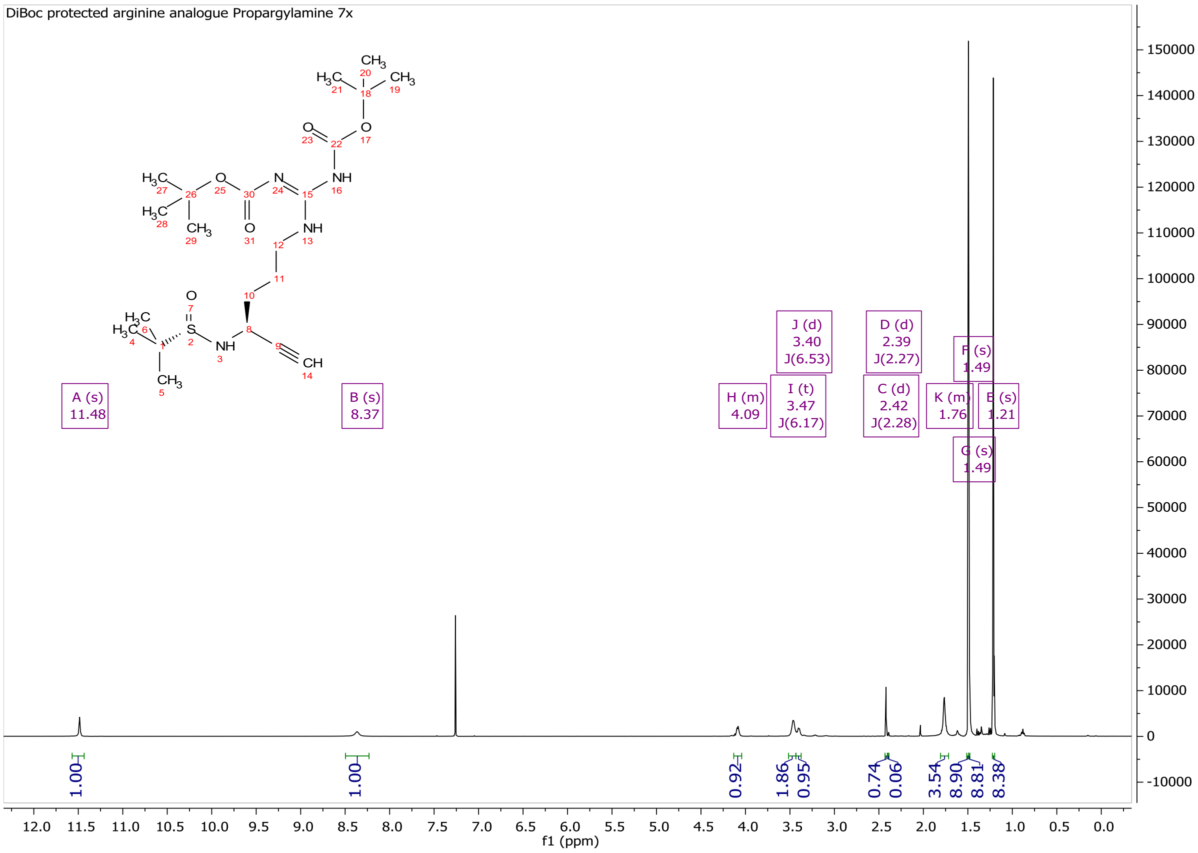
DiBoc protected arginine analogue Propargylamine 7x
A (s) 11.48
B (s) 8.37
H (m) 4.09
J (d) 3.40 J(6.53)
I (t) 3.47 J(6.17)
D (d) 2.39 J(2.27)
C (d) 2.42 J(2.28)
K (m) 1.76
E (s) 1.21
1.00
1.00
0.92
1.86
0.95
0.74
0.06
3.54
8.90
8.81
8.38
f1 (ppm)

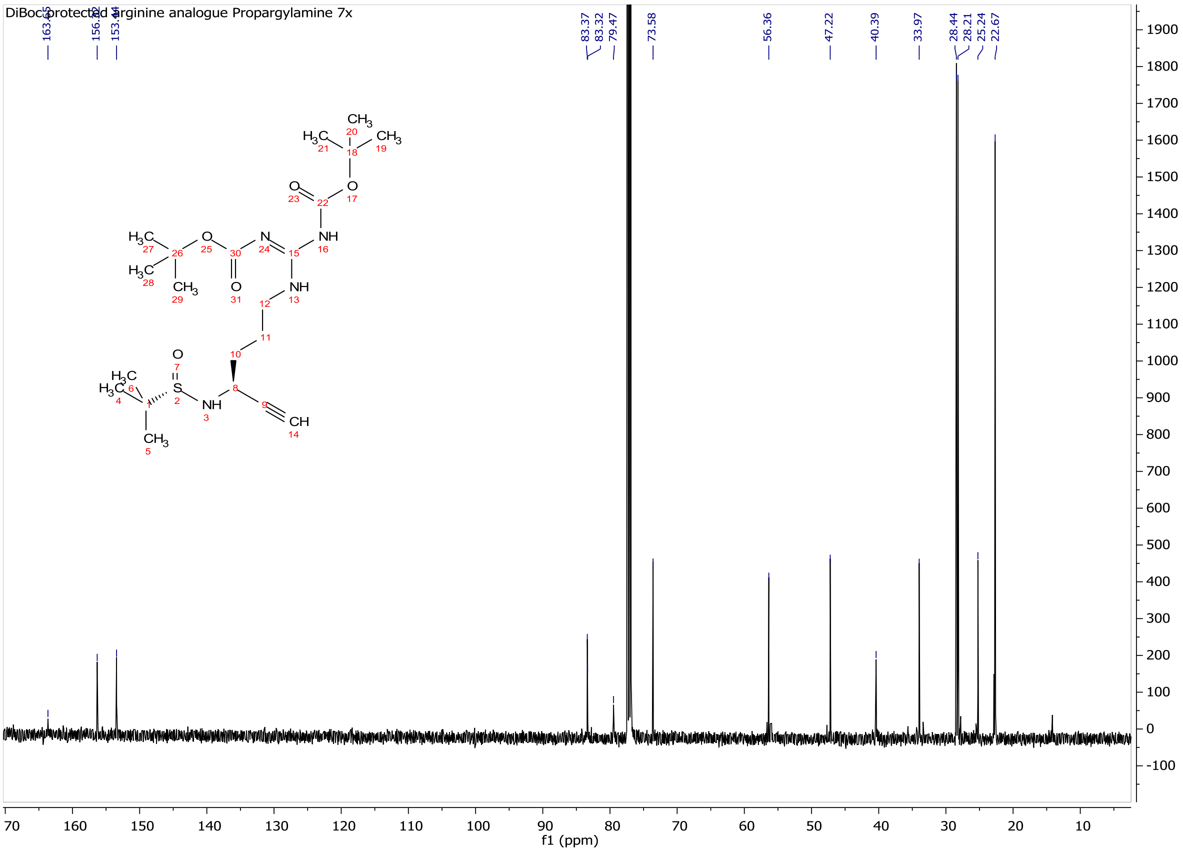

Hydrolysis of imine **5** forms hemiaminal **8**

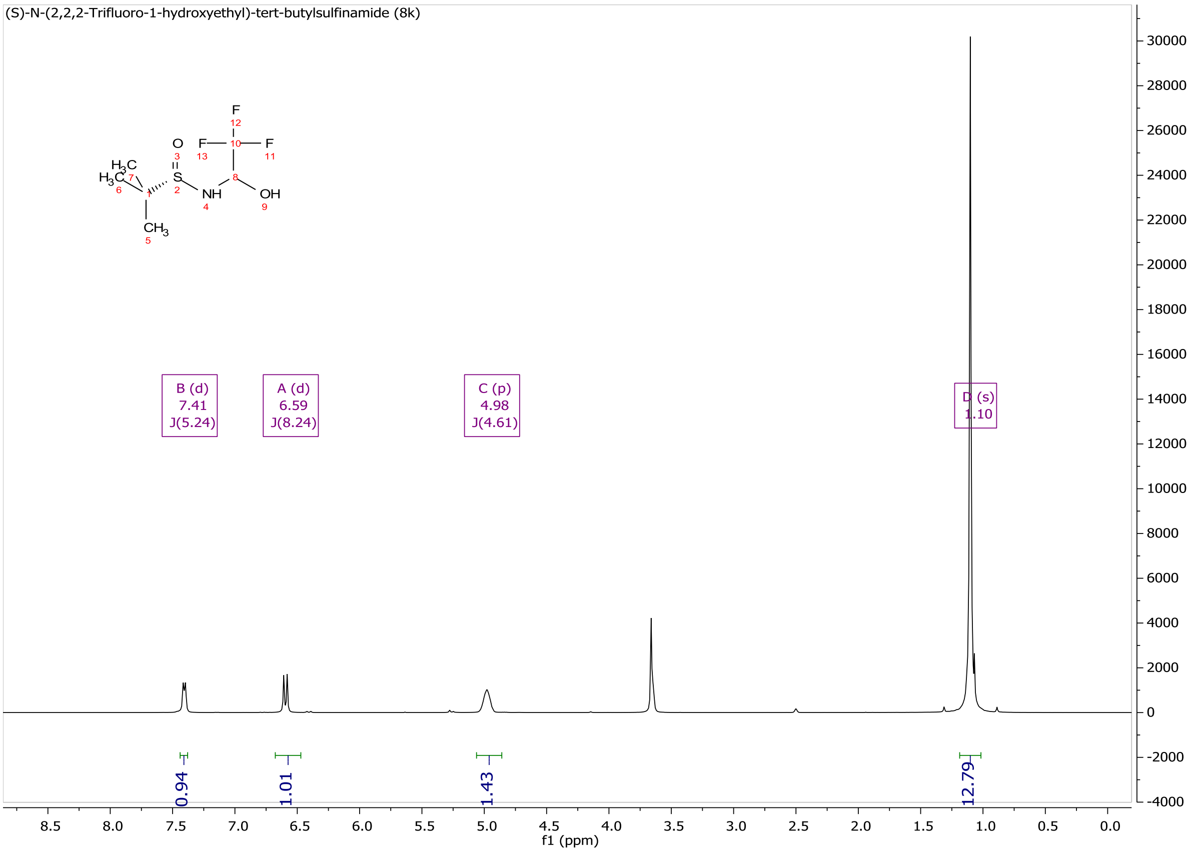

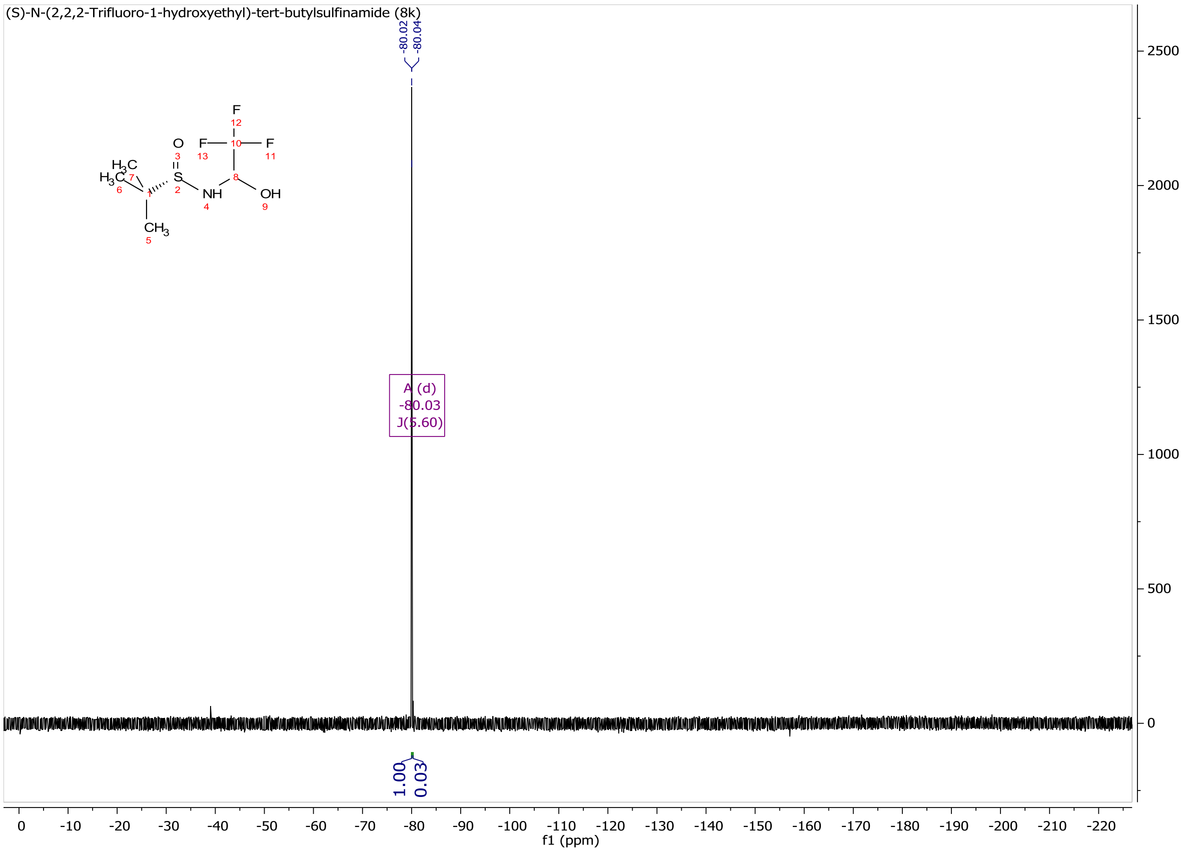
(S)-N-(2,2,2-Trifluoro-1-hydroxyethyl)-tert-butylsulfinamide (8k)
-80.02
-80.04
A (d)
-80.03
J(5.60)
1.00
0.03
f1 (ppm)

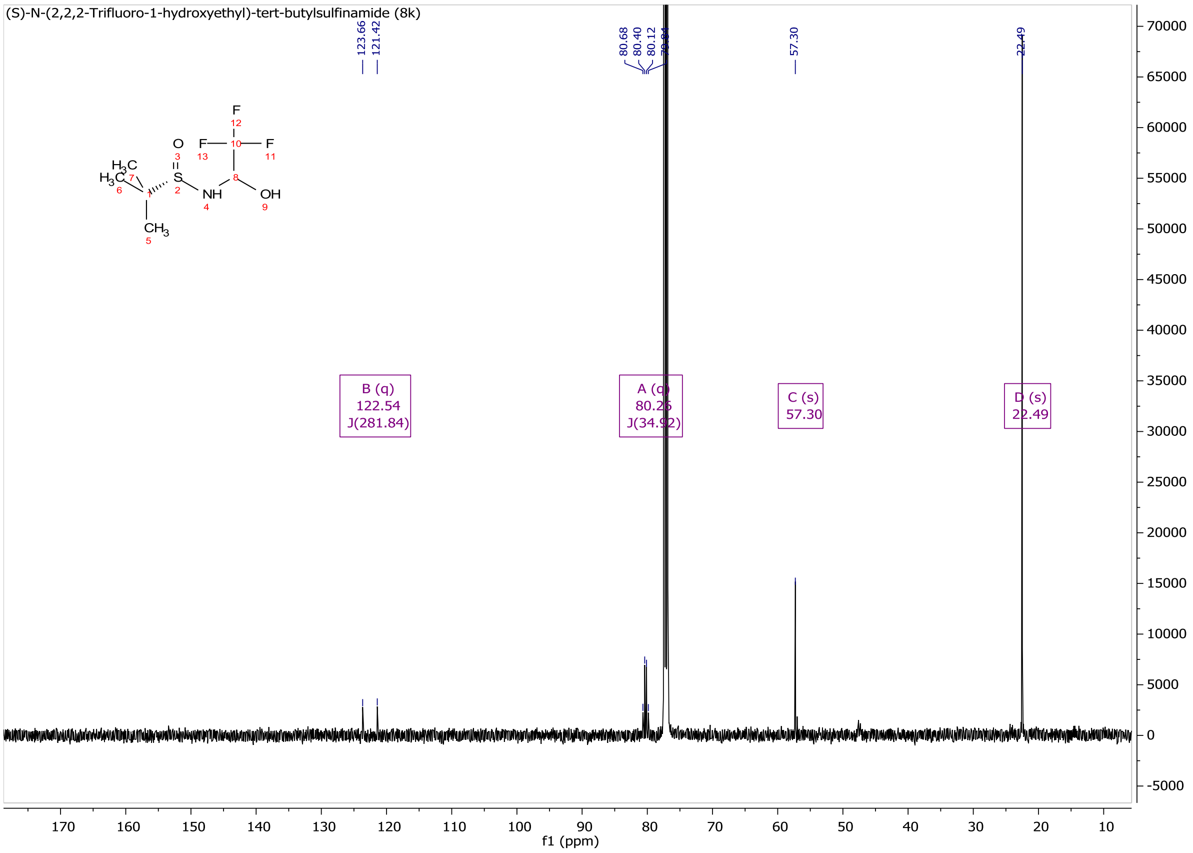
(S)-N-(2,2,2-Trifluoro-1-hydroxyethyl)-tert-butylsulfinamide (8k)
123.66
121.42
80.68
80.40
80.12
57.30
22.49
B (q)
122.54
J(281.84)
A (q)
80.25
J(34.92)
C (s)
57.30
D (s)
22.49
f1 (ppm)

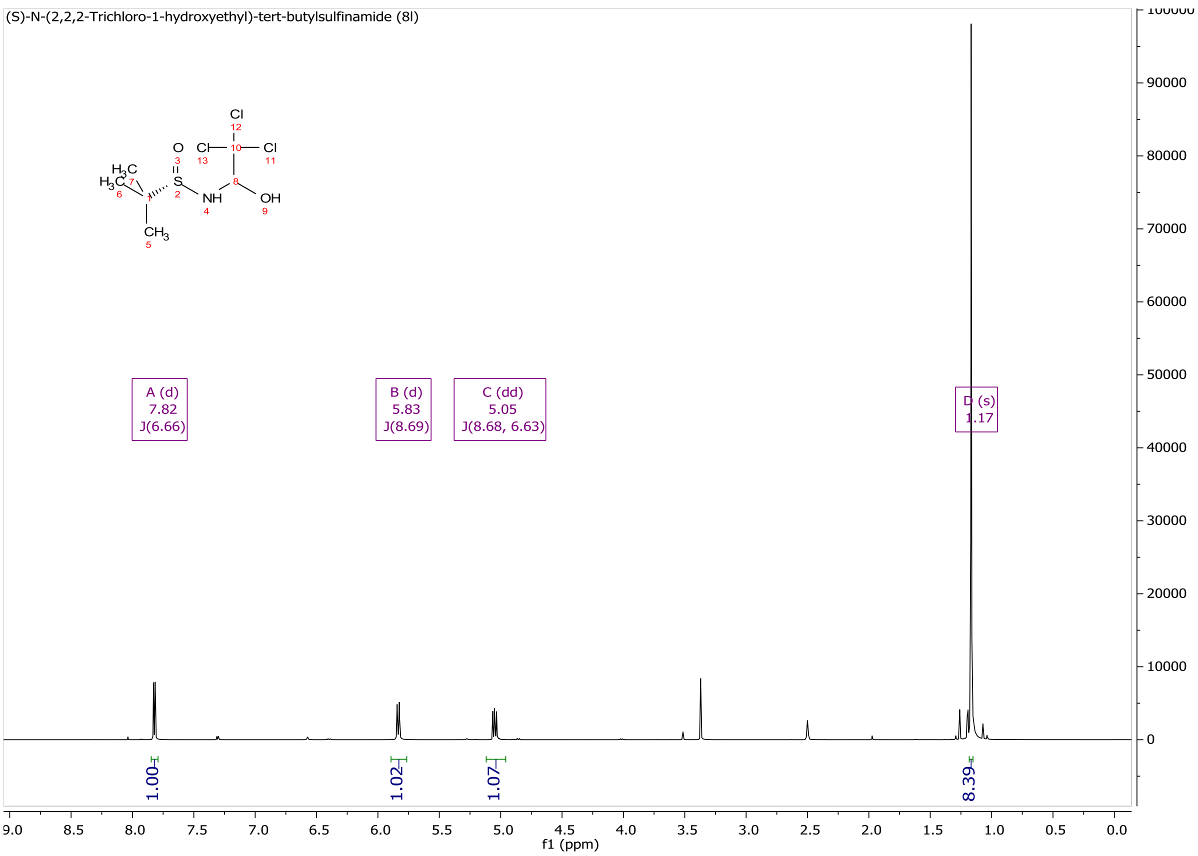
(S)-N-(2,2,2-Trichloro-1-hydroxyethyl)-tert-butylsulfinamide (8l)
A (d)
7.82
J(6.66)
B (d)
5.83
J(8.69)
C (dd)
5.05
J(8.68, 6.63)
D (s)
1.17
1.00
1.02
1.07
8.39
f1 (ppm)

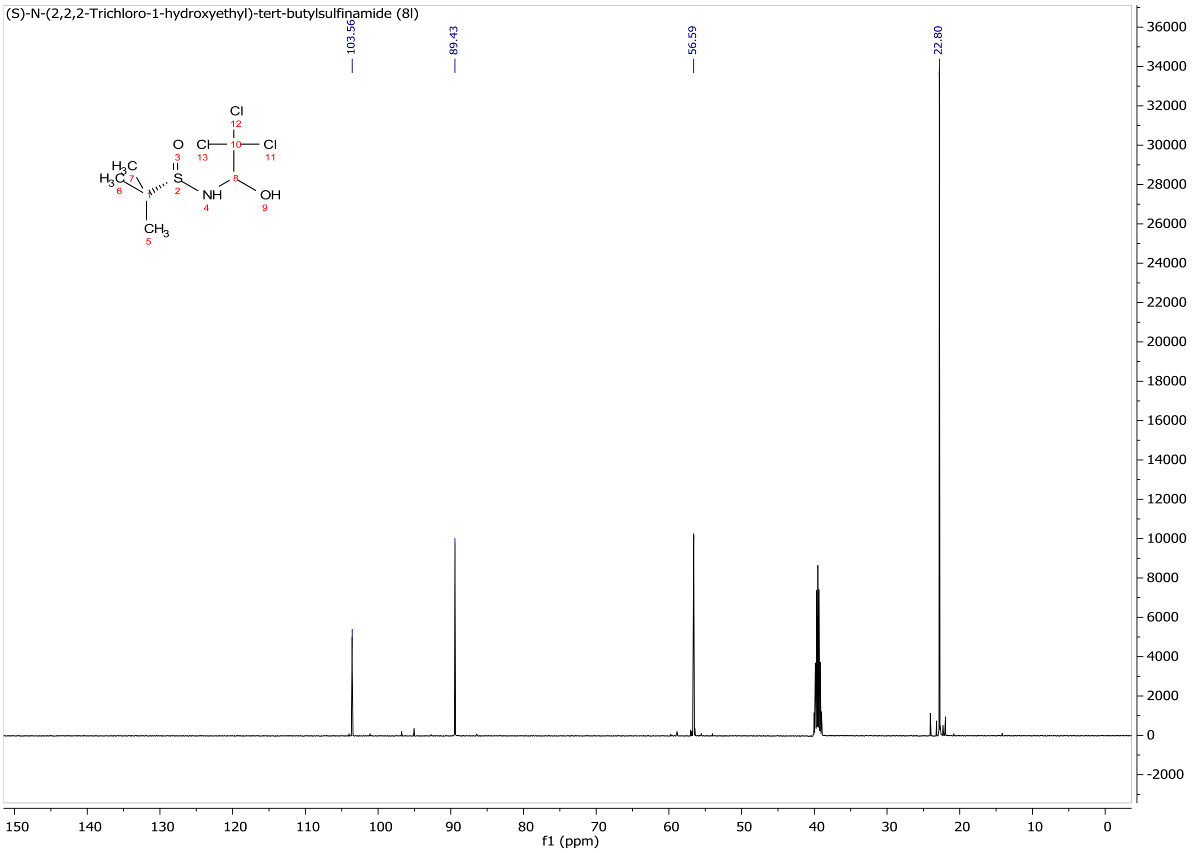
(S)-N-(2,2,2-Trichloro-1-hydroxyethyl)-tert-butylsulfinamide (8l)
103.56
89.43
56.59
22.80
f1 (ppm)

Side-product **9k** of the conversion of **5k** under conditions GP-3

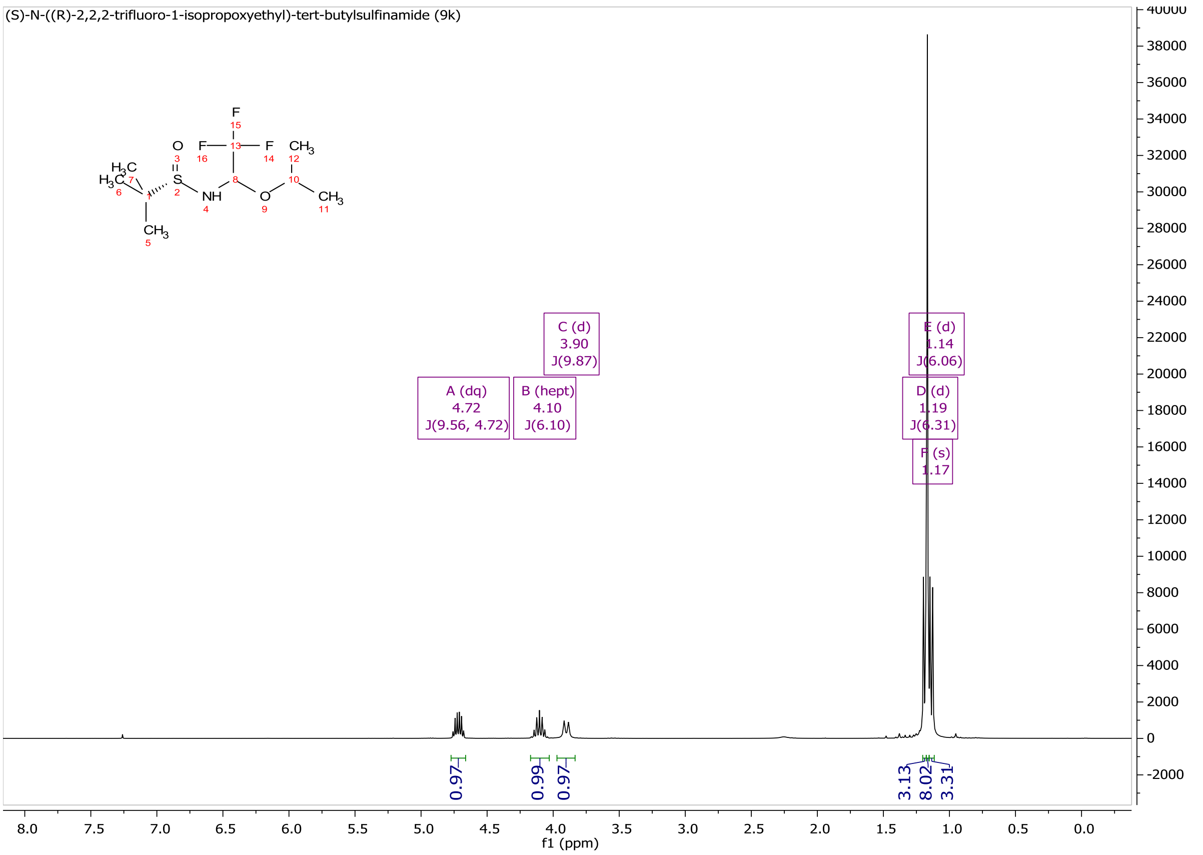

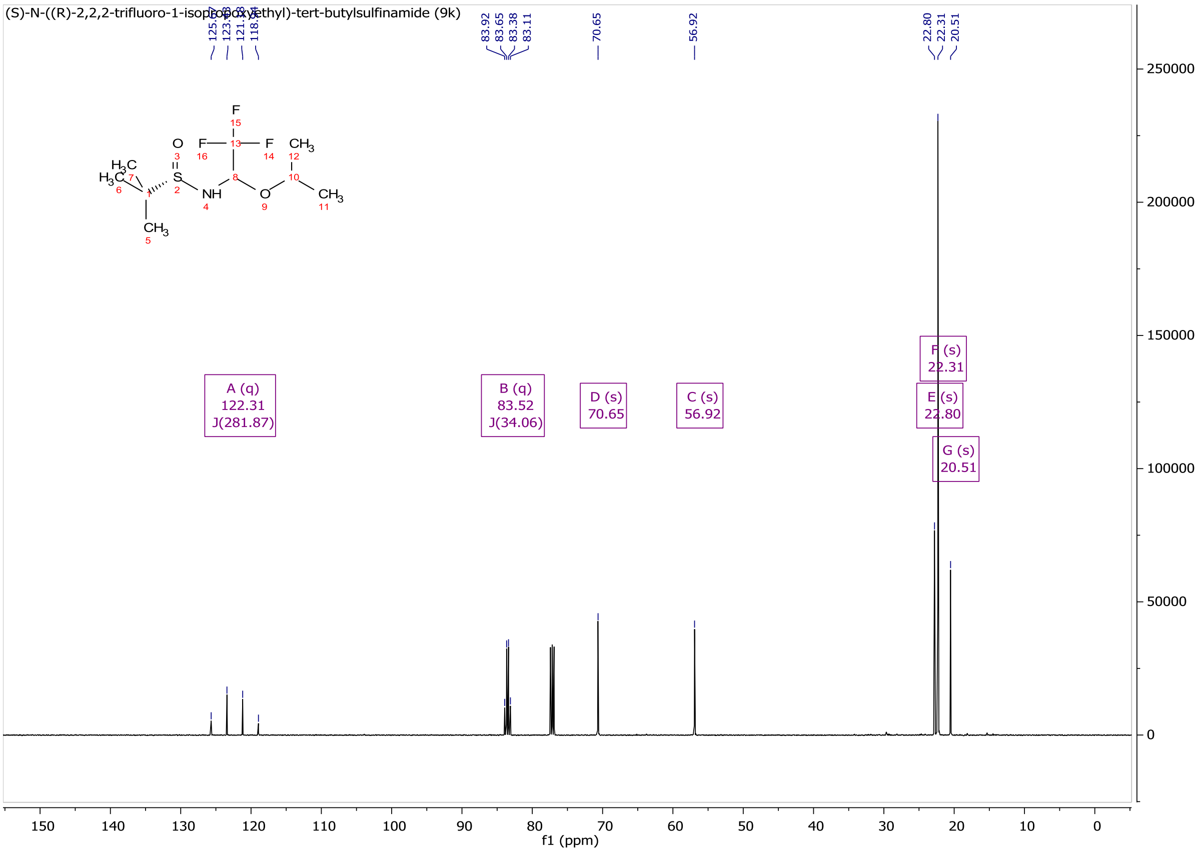

Side-product **10k** of the conversion of **5k** under conditions GP-4

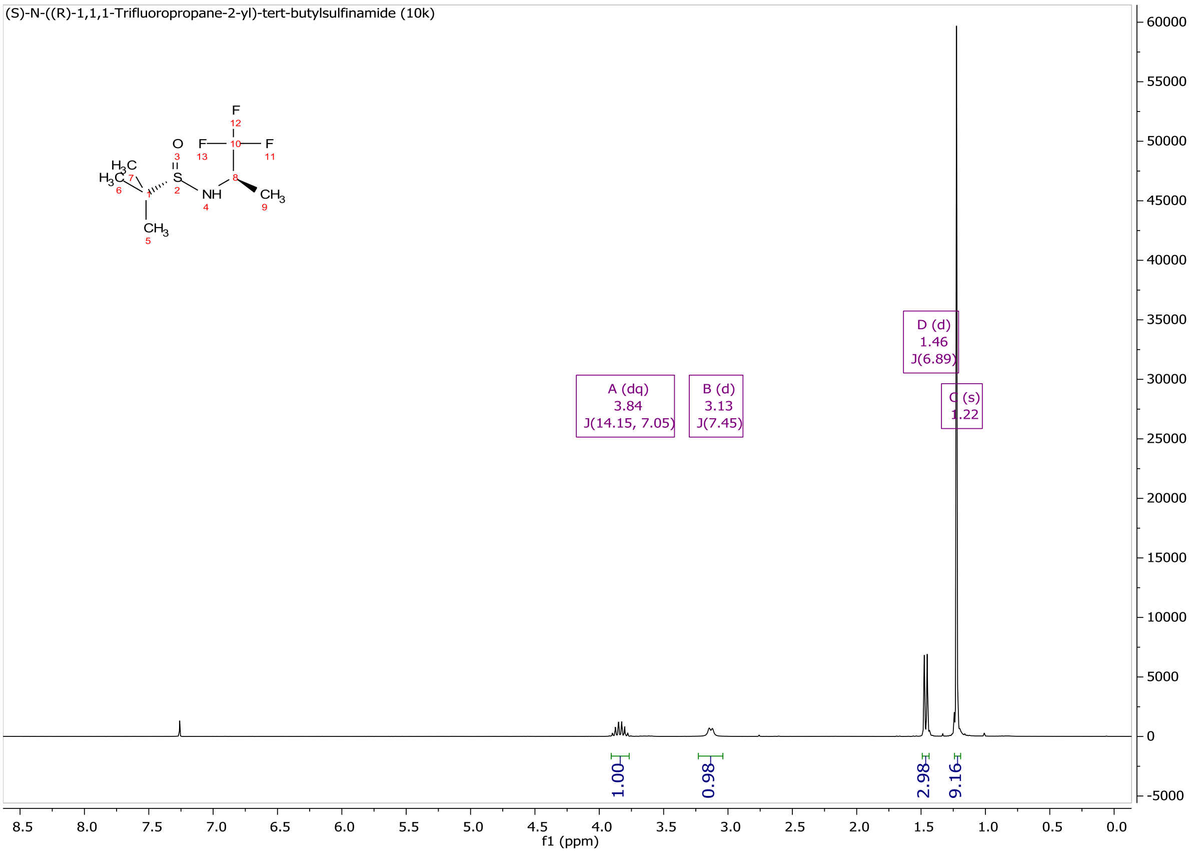

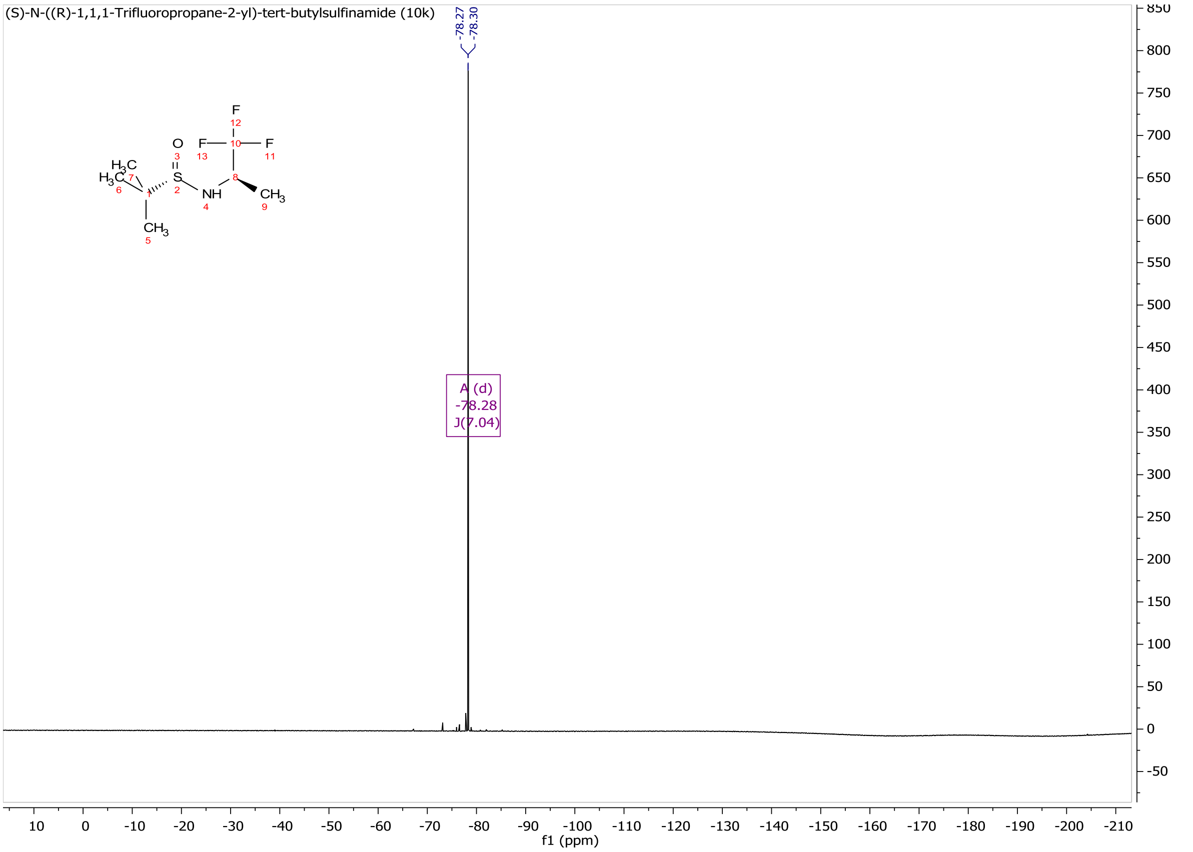

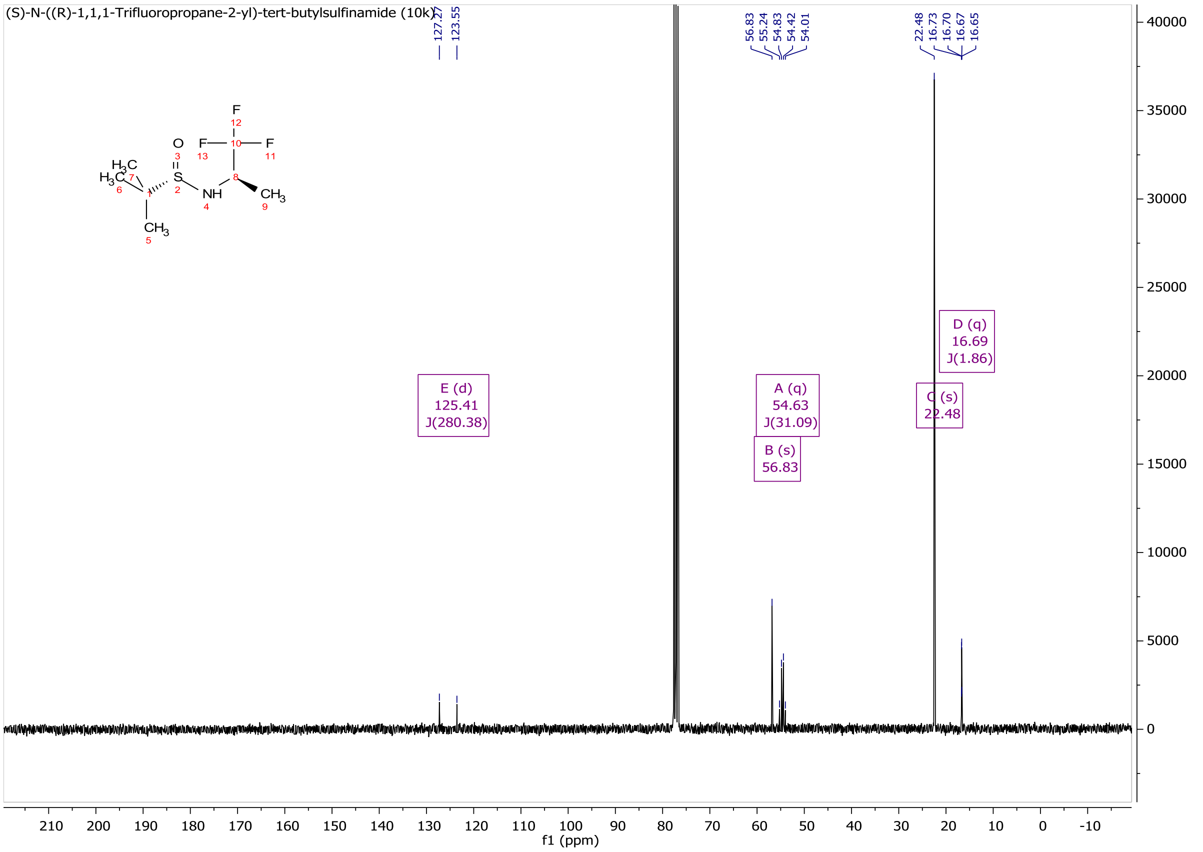

Sonogashira cross-coupling products: Peptidomimetics **11**

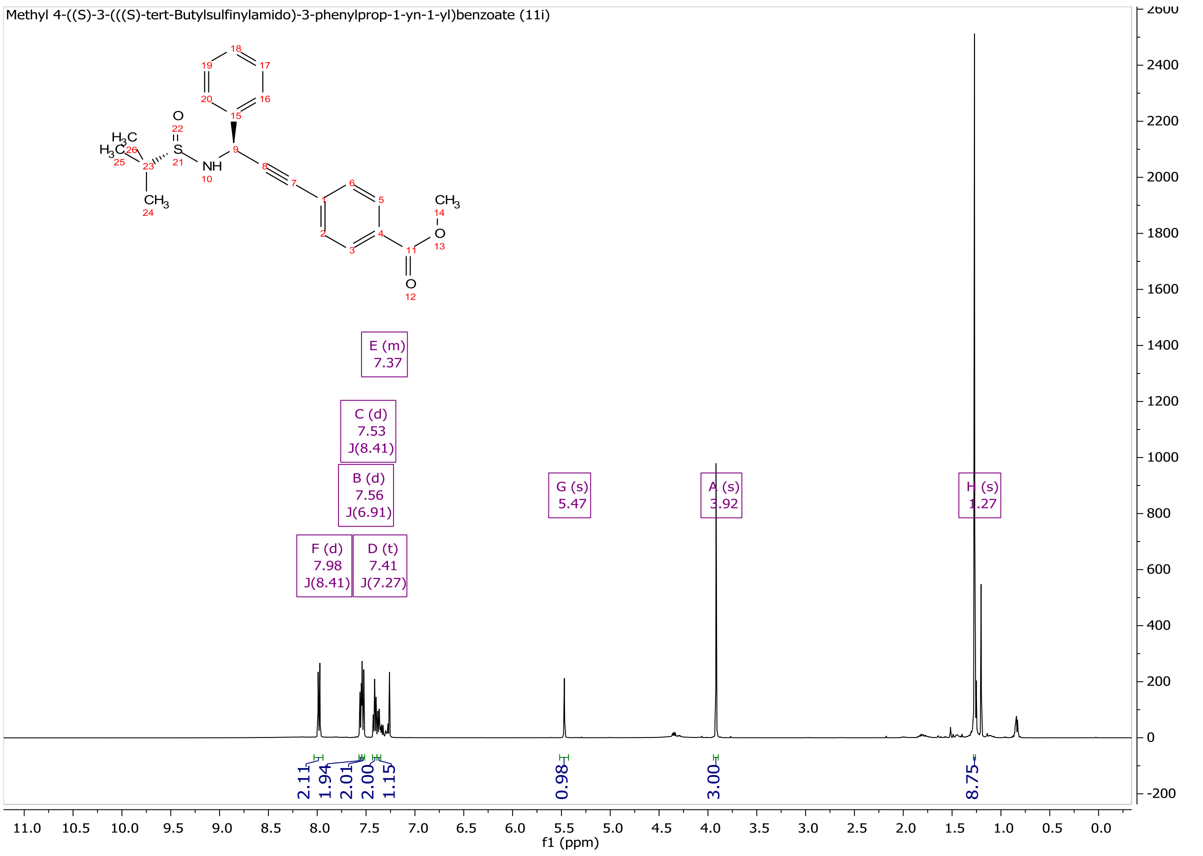

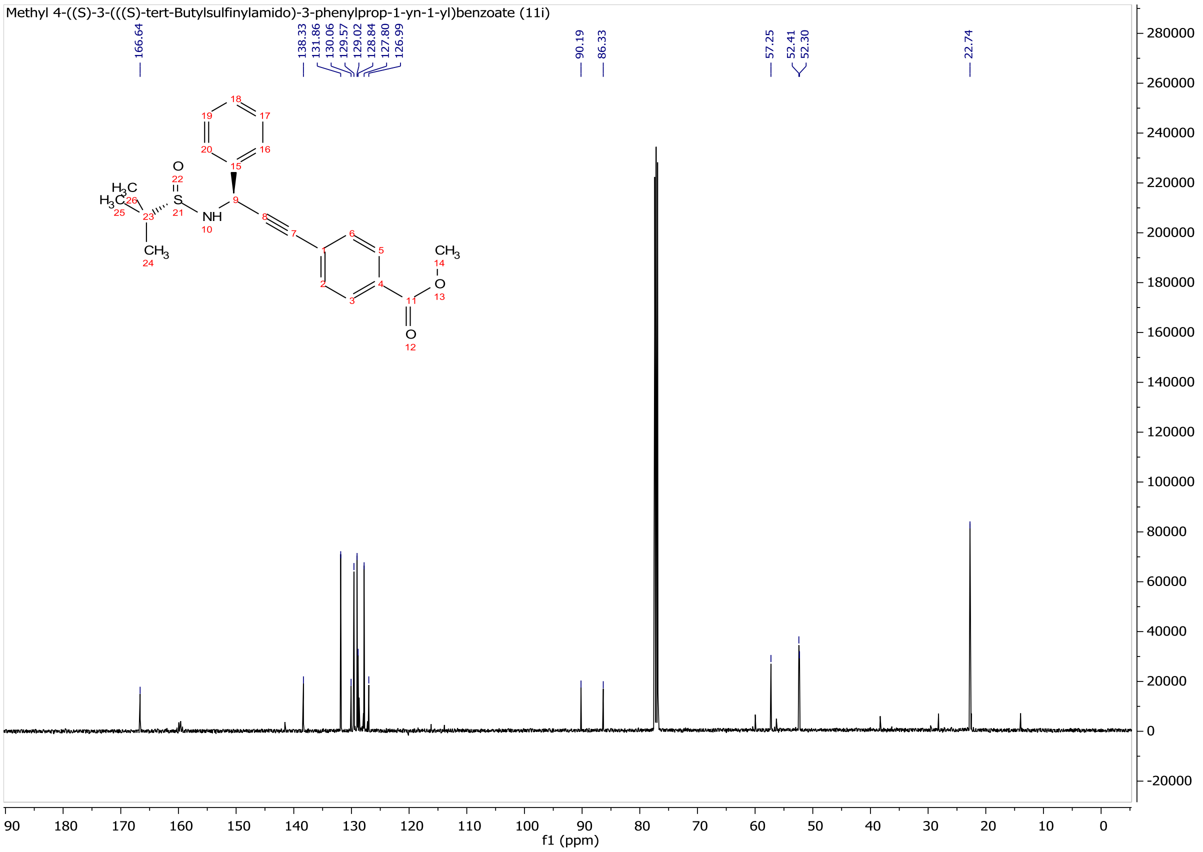

Rearrangementproducts: α,β-unsaturated imines **12**

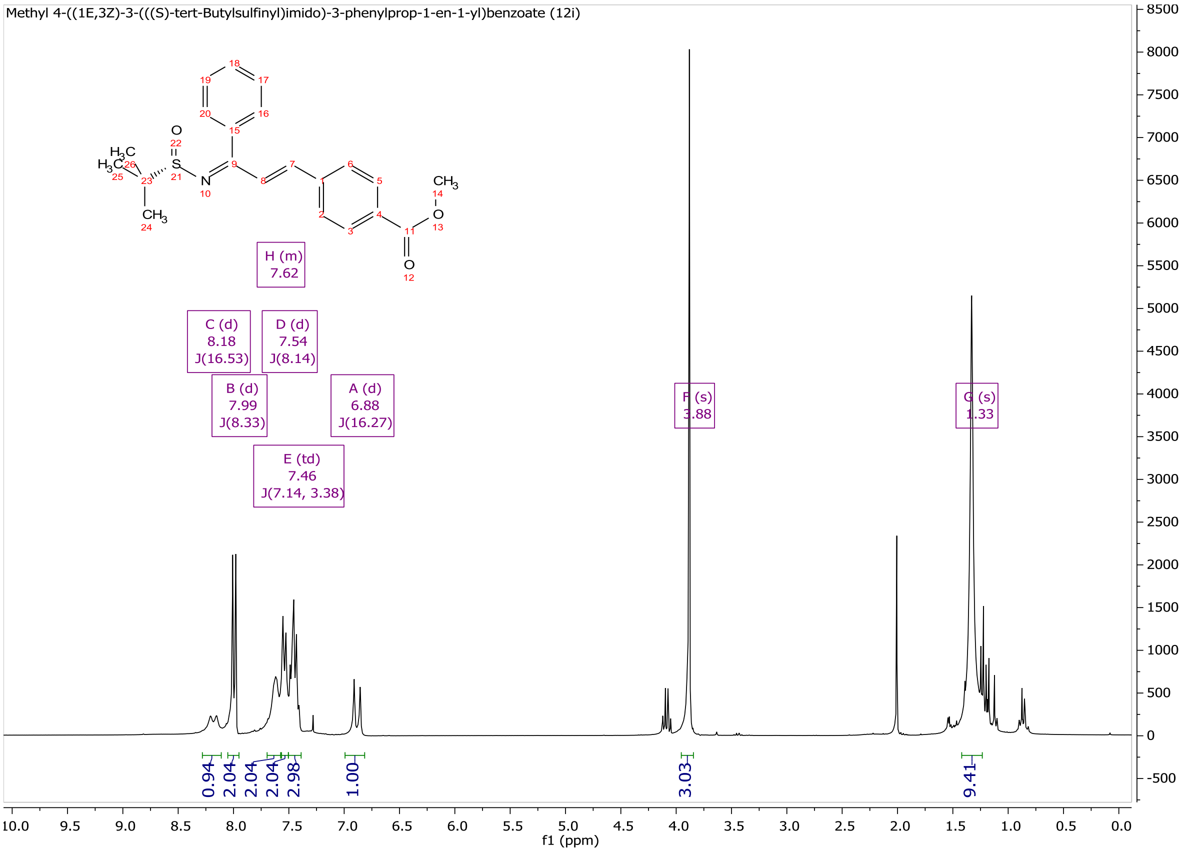

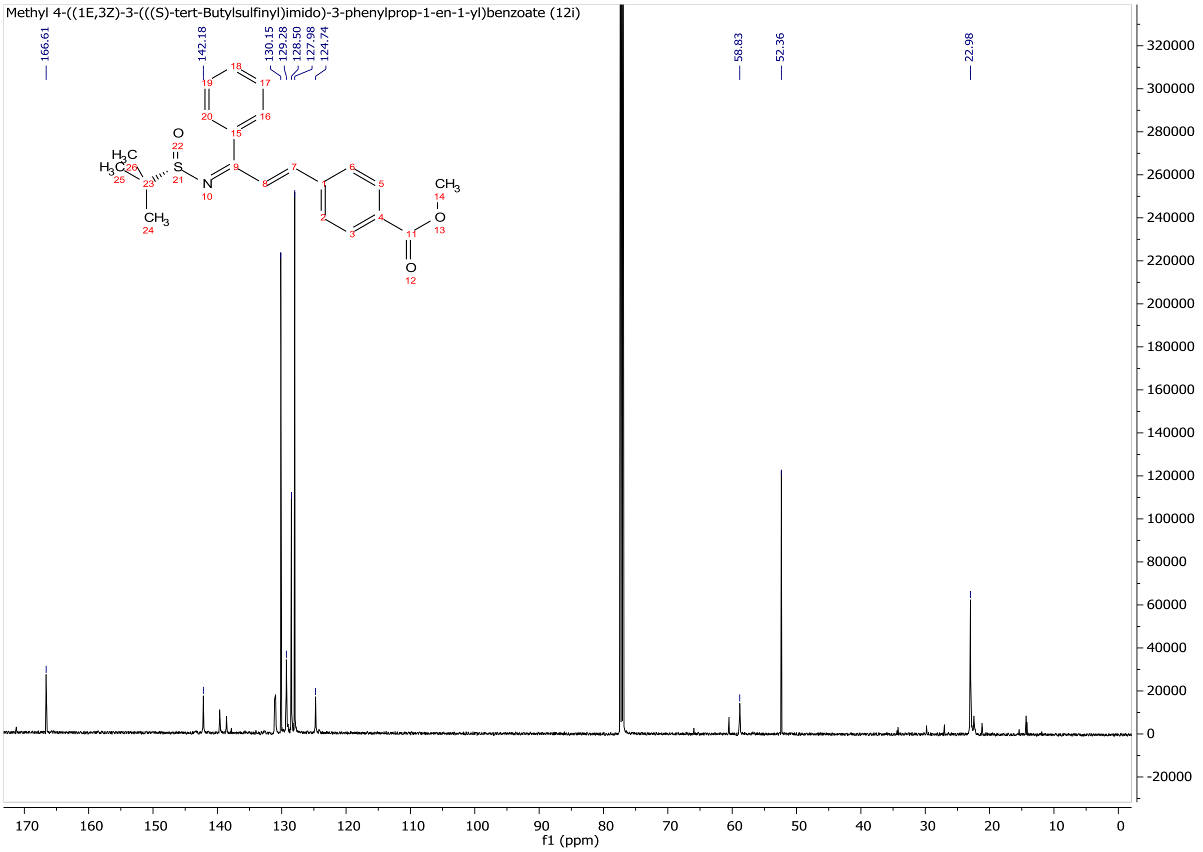

Intramolecular Huisgen reaction of **6w** gives triazole **13w**

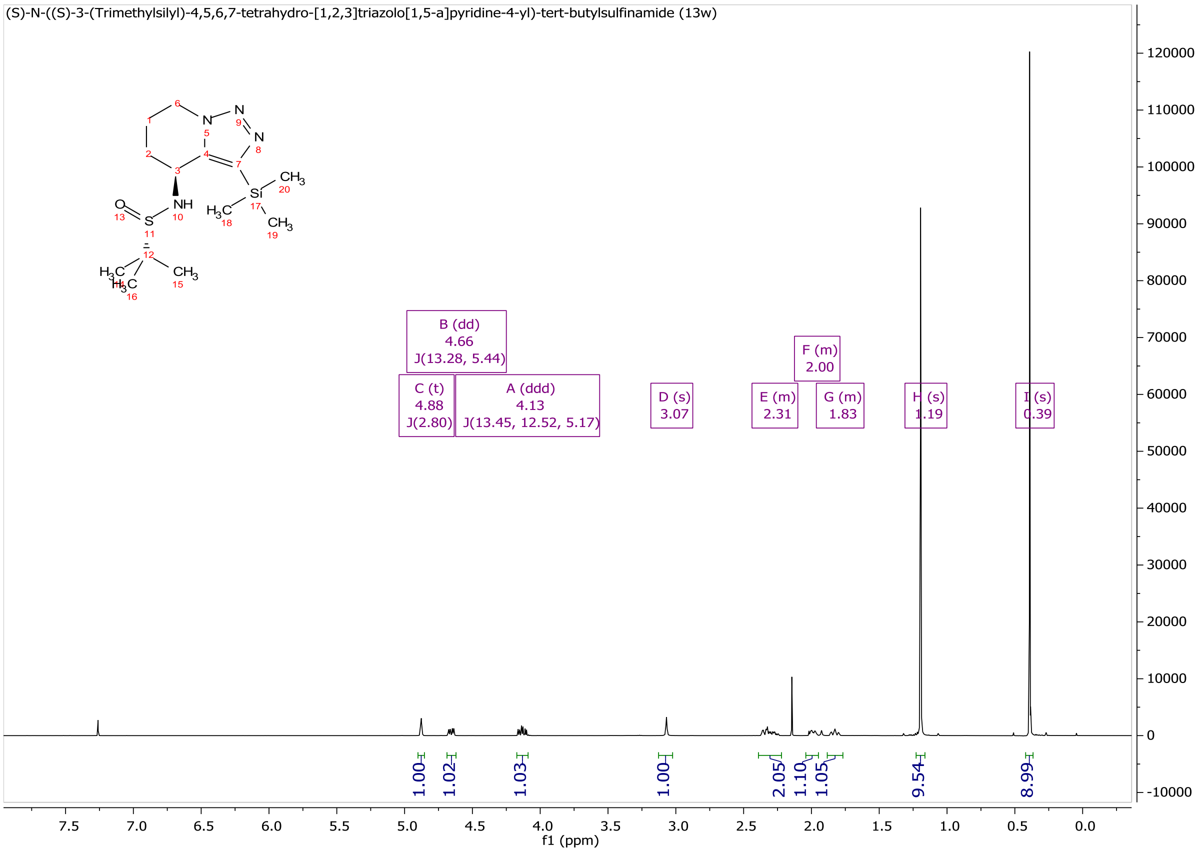

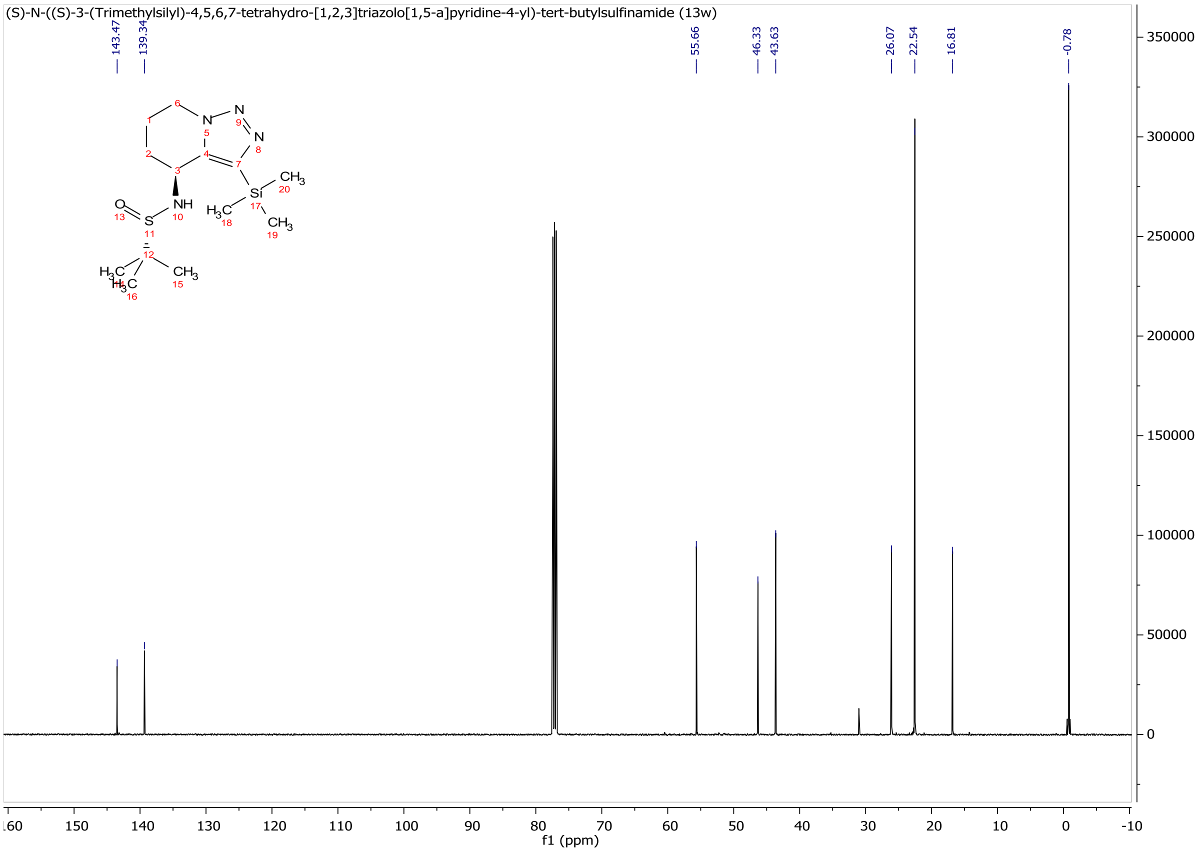

Intramolecular Huisgen reaction of **7wx** gives triazole **14w**

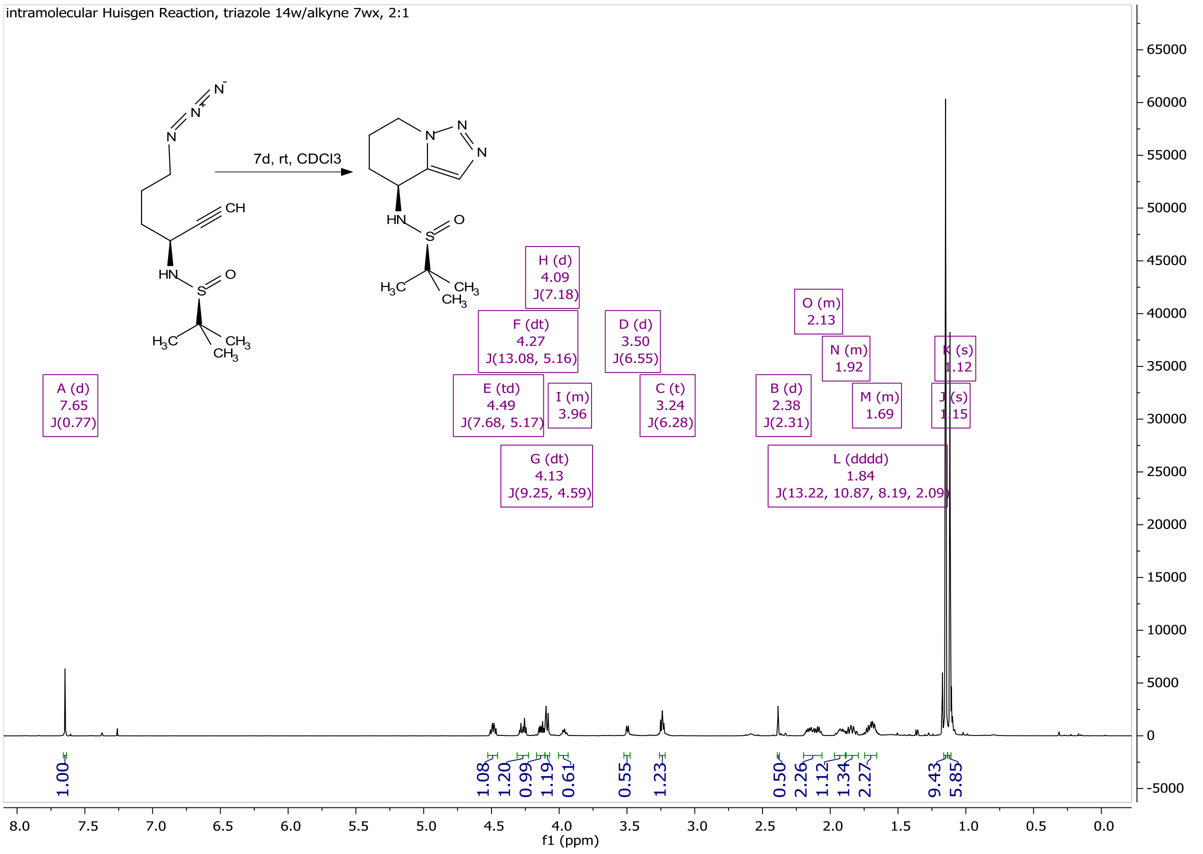

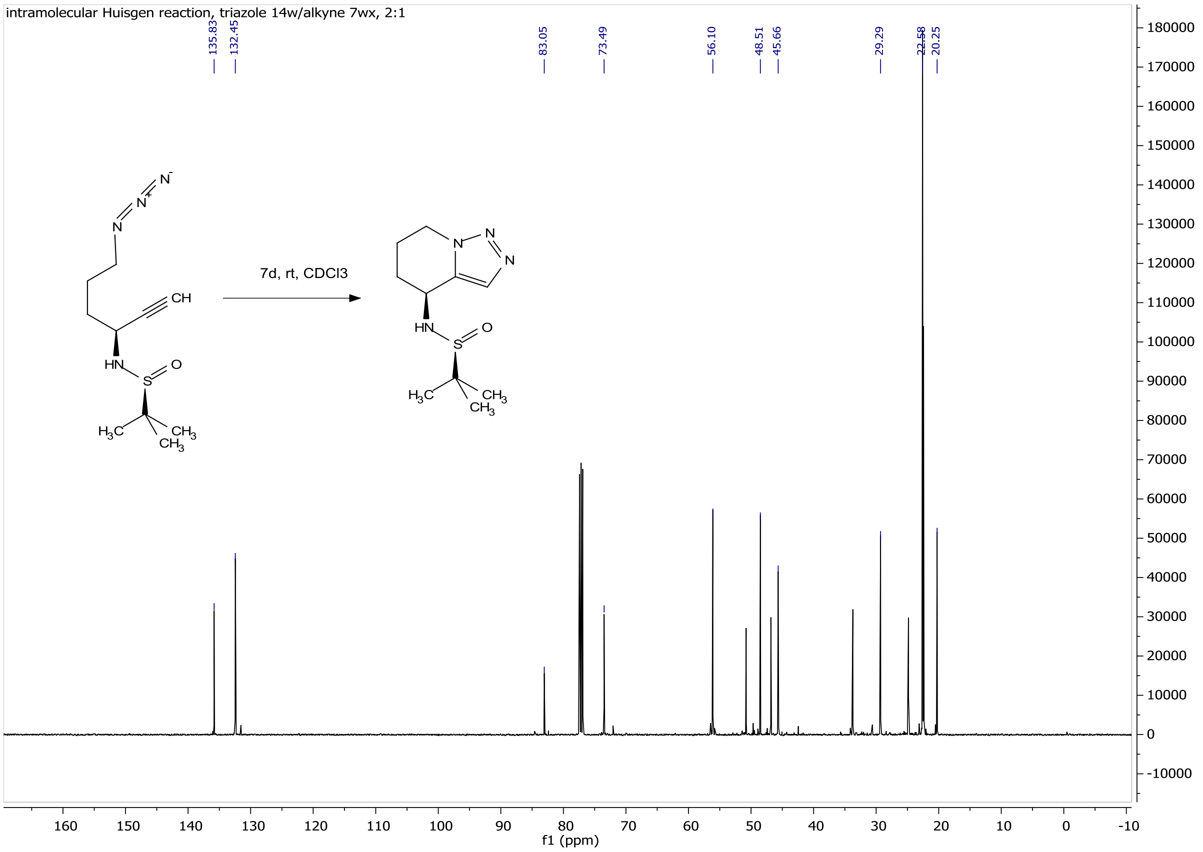
intramolecular Huisgen reaction, triazole 14w/alkyne 7wx, 2:1
135.83
132.45
83.05
73.49
56.10
48.51
45.66
29.29
22.58
20.25
7d, rt, CDCl3
f1 (ppm)

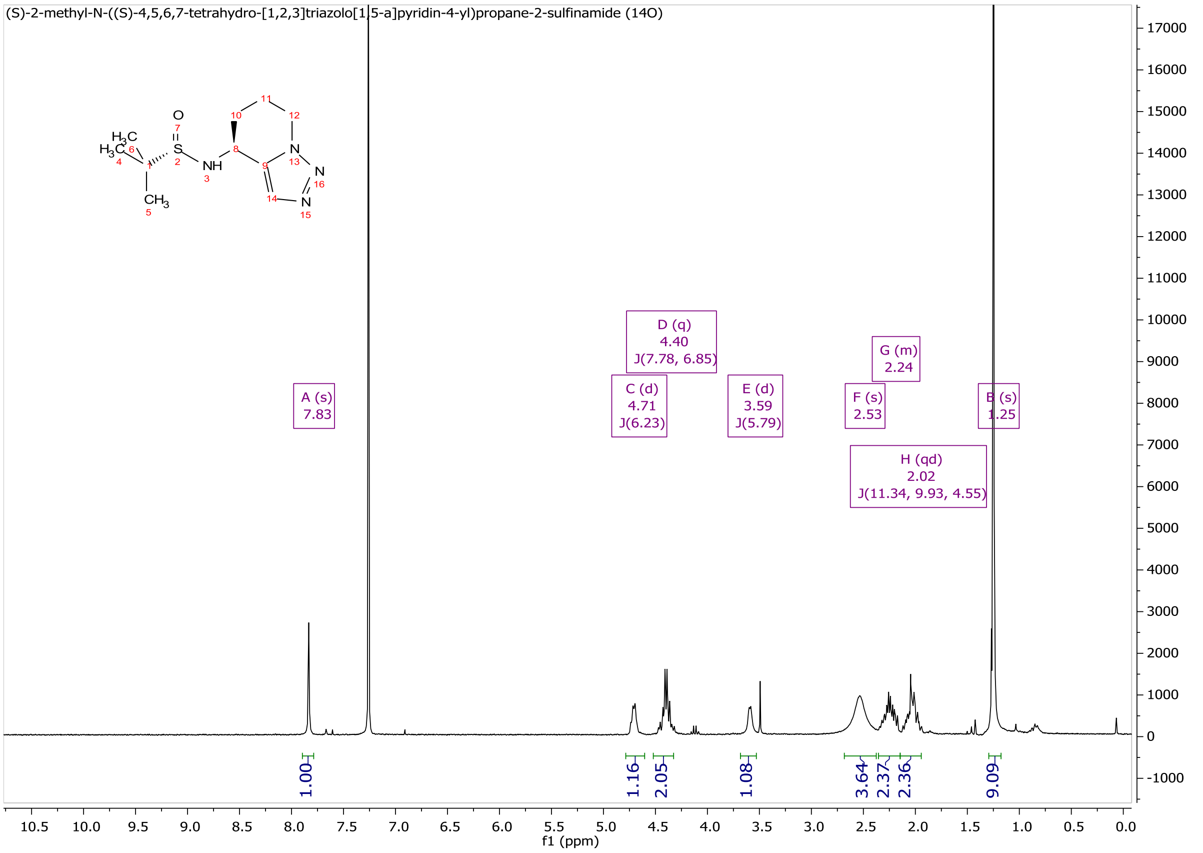
(S)-2-methyl-N-((S)-4,5,6,7-tetrahydro-[1,2,3]triazolo[1,5-a]pyridin-4-yl)propane-2-sulfinamide (14O)
A (s) 7.83
C (d) 4.71 J(6.23)
D (q) 4.40 J(7.78, 6.85)
E (d) 3.59 J(5.79)
F (s) 2.53
G (m) 2.24
H (qd) 2.02 J(11.34, 9.93, 4.55)
B (s) 1.25
1.00
1.16
2.05
1.08
3.64
2.37
2.36
9.09
f1 (ppm)
